# Supplementary material for: Adjunctive Probiotics Alleviates Asthmatic Symptoms via Modulating the Gut Microbiome and Serum Metabolome
Source: Microbiol Spectr. 2021 Oct 6;9(2):e00859-21. doi: 10.1128/Spectrum.00859-21 (PMC8510161; doi:10.1128/Spectrum.00859-21)
Supplement: Supplemental file 1 — Supplemental material. Download SPECTRUM00859-21_Supp_1_seq9.pdf, PDF file, 1.4 MB [file spectrum00859-21_supp_1_seq9.pdf]

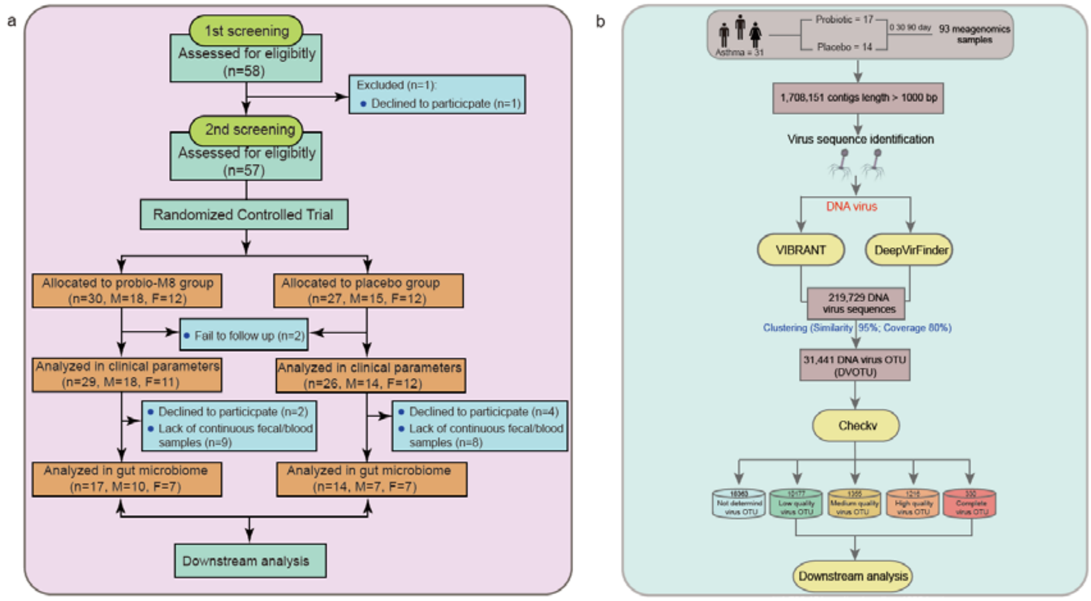

Figure S1. Patient selection process and identification of gut virome. (a) Information of screening 58 asthmatic patients for suitability of participating the clinical trial and samples collection. (b) Flowchart showing the pipeline of identification, annotation, and classification of gut phage features in the metagenome of asthmatic patients.

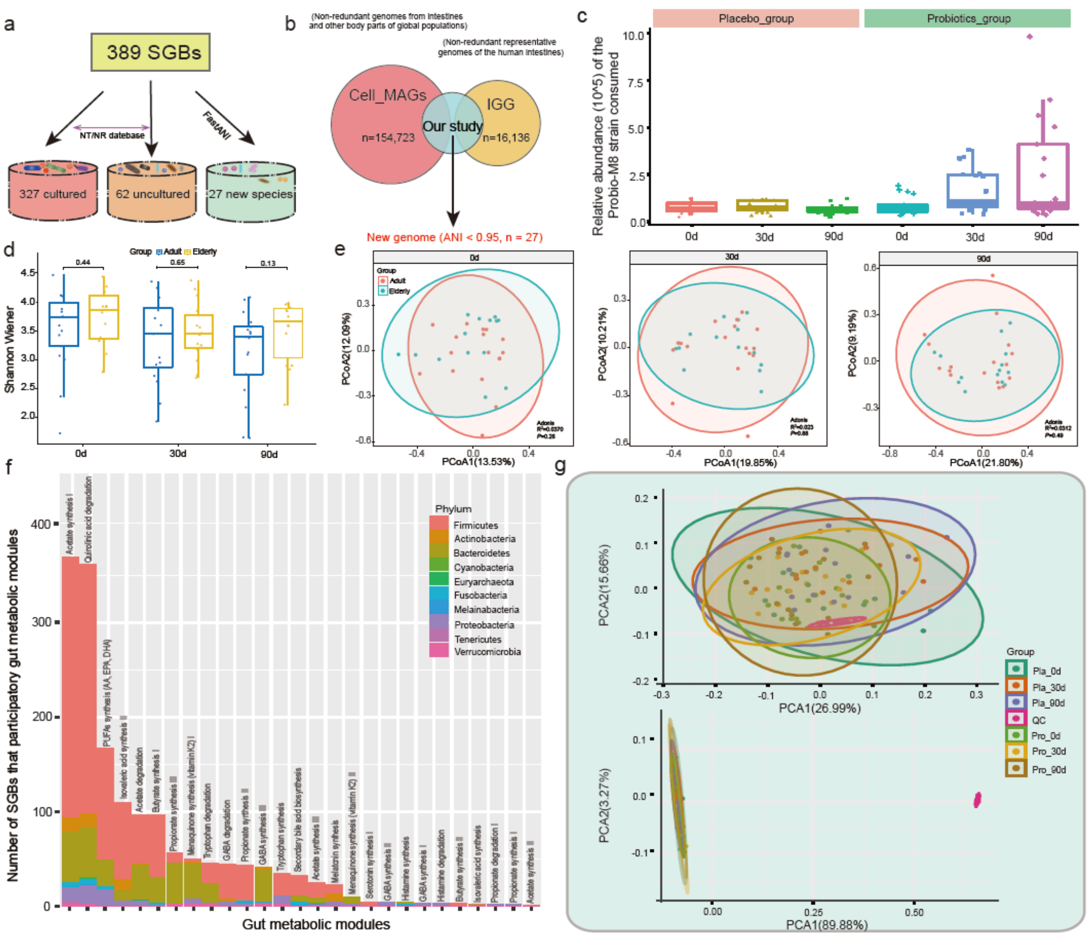

Figure S2. Genomic characteristics, metagenomic potential, and principle component analysis (PCA) of serum metabolomes of probiotic (Pro) and placebo (Pla) groups. (a) Among the 389 identified species-level genome bins (SGBs), 327 and 62 were previously cultured and uncultured, respectively. (b) Twenty-seven SGBs did not match with existing isolates or metagenome-assembled genomes (MAGs) in two major human microbiome datasets (IGG database and MAGs database established in Pasolli et al.). (c) The relative abundance of genomes of Probio-M8 strain (10^5) detected during the course of intervention. (d) Shannon diversity index of the adult and elderly groups at days 0 (0d), 30 (30d), and 90 (90d). (e) Principal coordinates analysis (PCoA) score plots of the the adult and elderly groups at different time points. (f) Distribution of gut metabolic modules across SGBs, grouped by phylum. (g) PCA of serum metabolome of the probiotic (Pro) and placebo (Pla) groups at day 0 (0d), day 30 (30d), and day 90 (90d). The positive (upper) and negative (lower) ion collection mode the quality control (QC) samples clustered

tightly in the score plots, confirming a highly stable chromatographic condition.

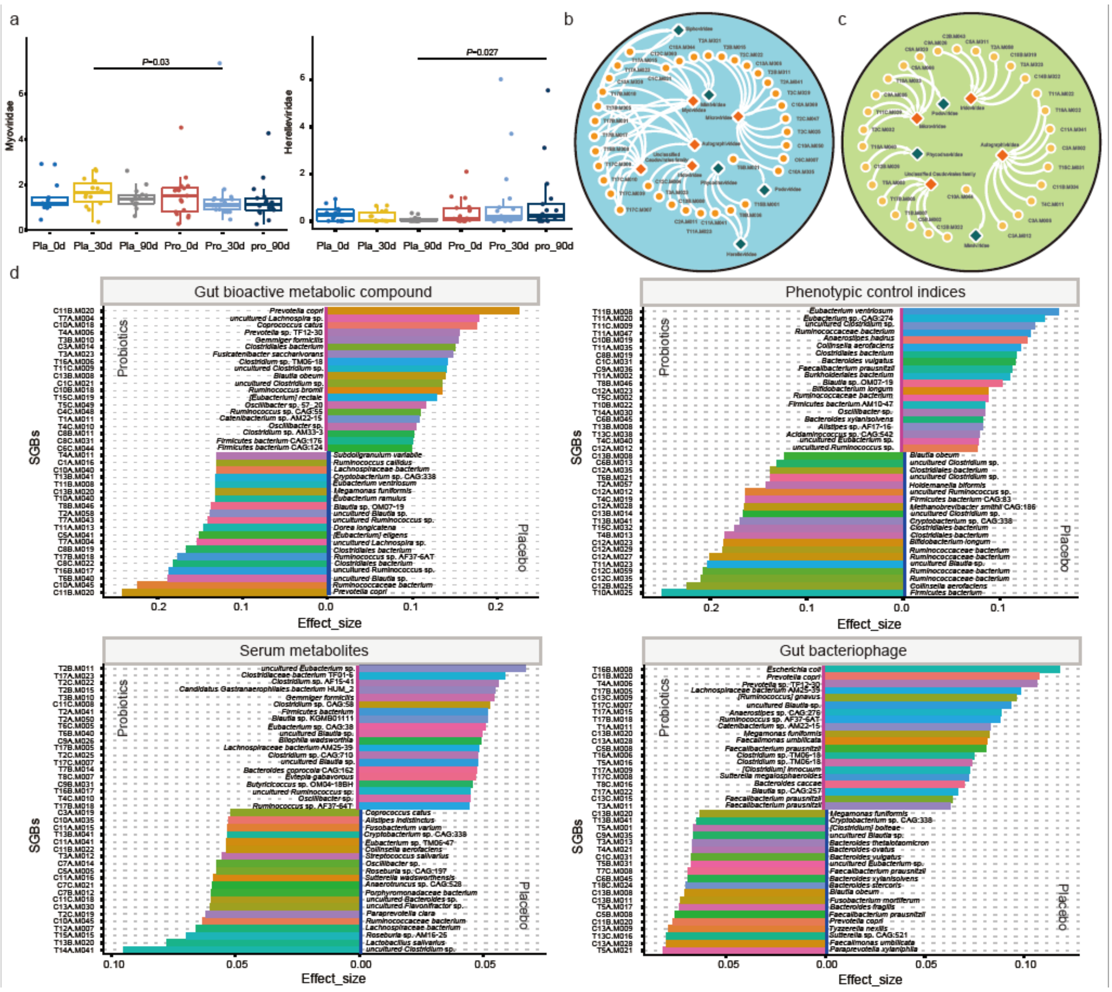

Figure S3. Significant differential gut bacteriophage, interactive correlation networks, effect size of gut microbiota on monitored features. (a) Significant differences in Myovirida and Herellevirida between the probiotic (Pro) and placebo (Pla) groups at day 0 (0d), day 30 (30d), and day 90 (90d). P values of significantly different pairs are stated. Interactive correlation networks between the gut species-level genome bins (SGBs) and virome in the (b) probiotic and (c) placebo groups. (d) Significant effect size of the top 20 specific SGBs on the predicted gut bioactive metabolic compound, clinical indexes, serum metabolites, and, gut bacteriophage.

Table S1. Information of subjects and samples

Subject number	Subject Information										
	Sample_ID	Gender	Age	Group	height (cm)	weight (kg)	History of smoking (year)	Average daily alcohol consumption (mL)	Group_time (T: probiotic; C: placebo; A: d0; B: d30; C: d90)	Fecal sample	Blood sample
	Sample_T1A								TA	√	√
1	Sample_T1B	F	28	Probiotics	174	65	10	80	TB	√	√
	Sample_T1C								TC	√	√
	Sample_T2A								TA	√	√
2	Sample_T2B	F	52	Probiotics	170	70	0	70	TB	√	√
	Sample_T2C								TC	√	√
	Sample_T3A								TA	√	√
3	Sample_T3B	F	62	Probiotics	157	48	0	50	TB	√	√
	Sample_T3C								TC	√	√
	Sample_T4A								TA	√	√
4	Sample_T4B	M	54	Probiotics	162	61	0	0	TB	√	√
	Sample_T4C								TC	√	√
	Sample_T5A								TA	√	√
5	Sample_T5B	F	63	Probiotics	164	73	0	50	TB	√	√
	Sample_T5C								TC	√	√
	Sample_T6A								TA	√	√
6	Sample_T6B	M	58	Probiotics	160	59	0	0	TB	√	√
	Sample_T6C								TC	√	√
	Sample_T7A								TA	√	√
7	Sample_T7B	F	55	Probiotics	170	74	20 (Quit smoking for 10 years)	50	TB	√	√
	Sample_T7C								TC	√	√
	Sample_T8A								TA	√	√
8	Sample_T8B	M	55	Probiotics	168	75.5	0	50	TB	√	√
	Sample_T8C								TC	√	√
	Sample_T9A								TA	–	–
9	Sample_T9B	F	50	Probiotics	155	62.5	0	70	TB	–	–
	Sample_T9C								TC	–	–
	Sample_T10A								TA	√	√
10	Sample_T10B	F	53	Probiotics	165	83	33	50	TB	√	√
	Sample_T10C								TC	√	√

Demographic data of two groups			
	Probiotics group	Placebo group	*P* value
Male	12	11	-
Female	17	15	-
Age	54.62±9.61	57.08±10.46	0.17
BMI	24.41±2.66	25.11±3.82	0.70
Alcohol consumption	28.28±30.25	20.38±27.20	0.39
History of smoking (year)	21.33±9.07	29.00±12.36	0.30

	Sample_T11A								TA	√	√
11	Sample_T11B	M	57	Probiotics	152	58	0	0	TB	√	√
	Sample_T11C								TC	√	√
	Sample_T12A								TA	√	√
12	Sample_T12B	F	49	Probiotics	184	90	0	80	TB	√	√
	Sample_T12C								TC	√	√
	Sample_T13A								TA	√	√
13	Sample_T13B	F	58	Probiotics	159	55	25 (Quit smoking for 6 years)	50	TB	√	√
	Sample_T13C								TC	√	√
	Sample_T14A								TA	√	√
14	Sample_T14B	M	54	Probiotics	150	57	0	0	TB	√	√
	Sample_T14C								TC	√	√
	Sample_T15A								TA	√	√
15	Sample_T15B	M	68	Probiotics	147	52	0	0	TB	√	√
	Sample_T15C								TC	√	√
	Sample_T16A								TA	√	√
16	Sample_T16B	F	59	Probiotics	174	71	28 (Quit smoking for 6 years)	70	TB	√	√
	Sample_T16C								TC	√	√
	Sample_T17A								TA	√	√
17	Sample_T17B	M	59	Probiotics	162	60	0	0	TB	√	√
	Sample_T17C								TC	√	√
	Sample_T18A								TA	√	√
18	Sample_T18B	M	57	Probiotics	162	61	0	0	TB	√	√
	Sample_T18C								TC	√	√
	Sample_T19A								TA	–	–
19	Sample_T19B	M	38	Probiotics	163	51	0	20	TB	–	–
	Sample_T19C								TC	–	–
	Sample_T20A								TA	–	–
20	Sample_T20B	M	72	Probiotics	169	75	0	0	TB	–	–
	Sample_T20C								TC	–	–
	Sample_T21A								TA	–	–
21	Sample_T21B	M	54	Probiotics	154	67	0	0	TB	–	–
	Sample_T21C								TC	–	–
	Sample_T22A								TA	–	–
22	Sample_T22B	M	51	Probiotics	165	59	0	0	TB	–	–
	Sample_T22C								TC	–	–

	Sample_T23A								TA	–	–
23	Sample_T23B	F	63	Probiotics	160	67	0	30	TB	–	–
	Sample_T23C								TC	–	–
	Sample_T24A								TA	–	–
24	Sample_T24B	M	49	Probiotics	167	67	0	0	TB	–	–
	Sample_T24C								TC	–	–
	Sample_T25A								TA	–	–
25	Sample_T25B	M	63	Probiotics	152	64	0	0	TB	–	–
	Sample_T25C								TC	–	–
	Sample_T26A								TA	–	–
26	Sample_T26B	F	50	Probiotics	169	60	12	50	TB	–	–
	Sample_T26C								TC	–	–
	Sample_T27A								TA	–	–
27	Sample_T27B	M	50	Probiotics	150	55	0	0	TB	–	–
	Sample_T27C								TC	–	–
	Sample_T28A								TA	–	–
28	Sample_T28B	M	69	Probiotics	150	50	0	0	TB	–	–
	Sample_T28C								TC	–	–
	Sample_T29A								TA	–	–
29	Sample_T29B	M	34	Probiotics	164	72.5	0	50	TB	–	–
	Sample_T29C								TC	–	–
	Sample_C1A								CA	√	√
30	Sample_C1B	F	63	Placebo	165	58	43	50	CB	√	√
	Sample_C1C								CC	√	√
	Sample_C2A								CA	√	√
31	Sample_C2B	M	61	Placebo	175	61	0	0	CB	√	√
	Sample_C2C								CC	√	√
	Sample_C3A								CA	√	√
32	Sample_C3B	F	65	Placebo	169	85	30 (Quit smoking for 20 years)	30	CB	√	√
	Sample_C3C								CC	√	√
	Sample_C4A								CA	√	√
33	Sample_C4B	M	52	Placebo	163	57	0	0	CB	√	√
	Sample_C4C								CC	√	√
	Sample_C5A								CA	√	√
34	Sample_C5B	M	65	Placebo	160	70	0	0	CB	√	√
	Sample_C5C								CC	√	√

	Sample_C6A								CA	√	√
35	Sample_C6B	M	64	Placebo	158	79	0	0	CB	√	√
	Sample_C6C								CC	√	√
	Sample_C7A								CA	√	√
36	Sample_C7B	M	71	Placebo	156	83	0	0	CB	√	√
	Sample_C7C								CC	√	√
	Sample_C8A								CA	√	√
37	Sample_C8B	M	61	Placebo	164	65	31 (Quit smoking for 7 years)	0	CB	√	√
	Sample_C8C								CC	√	√
	Sample_C9A								CA	√	√
38	Sample_C9B	F	36	Placebo	165	65	0	80	CB	√	√
	Sample_C9C								CC	√	√
	Sample_C10A								CA	√	√
39	Sample_C10B	F	65	Placebo	161	57	0	20	CB	√	√
	Sample_C10C								CC	√	√
	Sample_C11A								CA	√	√
40	Sample_C11B	M	67	Placebo	158	70	0	0	CB	√	√
	Sample_C11C								CC	√	√
	Sample_C12A								CA	√	√
41	Sample_C12B	F	62	Placebo	176	70	0	30	CB	√	√
	Sample_C12C								CC	√	√
	Sample_C13A								CA	√	√
42	Sample_C13B	F	62	Placebo	151	50	40	0	CB	√	√
	Sample_C13C								CC	√	√
	Sample_C14A								CA	√	√
43	Sample_C14B	M	36	Placebo	161	52	0	30	CB	√	√
	Sample_C14C								CC	√	√
	Sample_C15A								CA	–	–
44	Sample_C15B	F	45	Placebo	168	55	10 (Quit smoking for 1 months)	70	CB	–	–
	Sample_C15C								CC	–	–
	Sample_C16A								CA	–	–
45	Sample_C16B	F	68	Placebo	180	84	0	0	CB	–	–
	Sample_C16C								CC	–	–
	Sample_C17A								CA	–	–
46	Sample_C17B	M	40	Placebo	160	60	0	30	CB	–	–
	Sample_C17C								CC	–	–

	Sample_C18A								CA	–	–
47	Sample_C18B	F	46	Placebo	180	79	0	50	CB	–	–
	Sample_C18C								CC	–	–
	Sample_C19A								CA	–	–
48	Sample_C19B	M	64	Placebo	150	68	0	0	CB	–	–
	Sample_C19C								CC	–	–
	Sample_C20A								CA	–	–
49	Sample_C20B	M	61	Placebo	158	65.5	0	0	CB	–	–
	Sample_C20C								CC	–	–
	Sample_C21A								CA	–	–
50	Sample_C21B	M	41	Placebo	161	64	0	10	CB	–	–
	Sample_C21C								CC	–	–
	Sample_C22A								CA	–	–
51	Sample_C22B	F	61	Placebo	170	73	20 (Quit smoking for 12 years)	50	CB	–	–
	Sample_C22C								CC	–	–
	Sample_C23A								CA	–	–
52	Sample_C23B	F	48	Placebo	170	70	0	80	CB	–	–
	Sample_C23C								CC	–	–
	Sample_C24A								CA	–	–
53	Sample_C24B	M	54	Placebo	158	76	0	0	CB	–	–
	Sample_C24C								CC	–	–
	Sample_C25A								CA	–	–
54	Sample_C25B	M	63	Placebo	158	66.5	0	0	CB	–	–
	Sample_C25C								CC	–	–
	Sample_C26A								CA	–	–
55	Sample_C26B	M	63	Placebo	172	70	0	0	CB	–	–
	Sample_C26C								CC	–	–

Table S2. Metagenome sequencing and assembly statistics

Sample ID	Amount of generated data		Assembly results				
	Raw data (Gbp)	Clean data (Gbp)	Number of contigs	Total length (bp)	N50 length (bp)	N90 length (bp)	Max length (bp)
Sample_T1A	5.99	5.90	6050	52670167	17297	3070	346711
Sample_T1B	4.53	4.45	4130	42119467	23186	3347	453789
Sample_T1C	6.86	6.69	6954	46301602	10218	2627	402043
Sample_T2A	6.18	6.10	27518	226016731	16280	2854	646320
Sample_T2B	6.36	6.28	31824	236029712	13369	2705	621851
Sample_T2C	8.54	8.39	22951	208148618	22492	2941	731178
Sample_T3A	7.16	7.06	14937	151105738	24656	3290	550226
Sample_T3B	6.10	6.01	17761	146547381	19518	2739	456946
Sample_T3C	6.28	6.17	12286	146145572	33722	3675	549872
Sample_T4A	6.31	6.20	14100	150765621	36208	3249	527538
Sample_T4B	6.05	5.93	27618	212836547	15689	2700	552032
Sample_T4C	7.97	7.80	14643	163634995	32436	3507	740622
Sample_T5A	7.50	7.32	7759	121596366	49637	4887	590821
Sample_T5B	6.25	6.15	12480	158936823	34732	4083	540098
Sample_T5C	5.84	5.76	22099	189754247	19154	2902	695358
Sample_T6A	6.30	6.18	25546	191811239	13130	2751	588470
Sample_T6B	8.58	8.44	18196	169064696	22442	3015	551712
Sample_T6C	5.83	5.74	22313	186054064	16682	2873	414165
Sample_T7A	7.26	7.14	24162	195851464	15629	2870	692608
Sample_T7B	6.74	6.62	14361	126420843	18185	3016	694320
Sample_T7C	6.76	6.64	12264	99343893	16423	2794	383815
Sample_T8A	6.49	6.39	24904	210097222	16441	2944	462866
Sample_T8B	6.20	6.11	31703	223870896	10979	2718	451461
Sample_T8C	5.87	5.74	17179	171686222	30942	3022	451666
Sample_T10A	8.92	8.71	18630	192772309	25428	3333	445295
Sample_T10B	8.36	8.25	18675	152645564	16394	2867	567911
Sample_T10C	5.44	5.30	7803	69464031	20196	2944	254942
Sample_T11A	7.45	7.32	17242	183303516	28832	3307	754951
Sample_T11B	8.49	8.34	21380	186241197	17732	3045	751021
Sample_T11C	7.53	7.44	19740	182842861	20631	3101	640277
Sample_T12A	6.04	5.94	32456	238349809	12653	2718	344909
Sample_T12B	6.68	6.59	15455	121227734	14515	2812	564984
Sample_T12C	6.27	6.14	17631	134937085	17425	2649	351530

Sample_T13A	7.54	7.46	16976	136063050	15838	2848	595503
Sample_T13B	6.98	6.88	21642	210541158	23017	3198	632246
Sample_T13C	7.58	7.50	16638	156894306	23100	3112	712395
Sample_T14A	7.65	7.58	20823	184413291	21509	2883	688516
Sample_T14B	6.89	6.78	20913	179326000	17684	2980	531669
Sample_T14C	6.09	6.01	13130	124410772	22394	3053	820226
Sample_T15A	7.58	7.51	18874	174222364	28396	2870	473217
Sample_T15B	6.90	6.79	10609	77771877	13890	2630	405107
Sample_T15C	6.77	6.66	14929	153411347	26628	3271	433776
Sample_T16A	6.56	6.46	12312	104376060	17024	2907	612330
Sample_T16B	6.76	6.63	20236	189951316	20295	3160	603995
Sample_T16C	6.59	6.54	16612	165287718	22265	3294	638797
Sample_T17A	8.38	8.31	6528	96876907	50427	4599	472243
Sample_T17B	5.13	5.05	6006	78962194	38961	4054	306980
Sample_T17C	6.67	6.58	3612	61065723	55299	5496	511205
Sample_T18A	6.31	6.27	15674	112990839	13045	2620	678232
Sample_T18B	6.13	6.07	12884	115426869	19920	2999	599982
Sample_T18C	5.64	5.56	14282	118701362	18913	2794	809736
Sample_C1A	6.18	6.05	27217	209761058	14372	2761	648305
Sample_C1B	7.82	7.70	30659	243796716	14641	2853	648307
Sample_C1C	7.49	7.36	21535	207423146	25868	3048	682022
Sample_C2A	6.51	6.39	31414	230997178	12779	2714	450207
Sample_C2B	6.05	5.94	29061	213734904	13326	2680	591300
Sample_C2C	6.46	6.35	25918	182189392	11524	2635	518369
Sample_C3A	9.05	8.89	24100	211087657	18683	2980	607327
Sample_C3B	7.07	6.94	17798	137319044	15395	2702	421149
Sample_C3C	6.65	6.53	20281	170882575	17728	2847	601314
Sample_C4A	7.20	7.09	23574	194266410	17165	2821	670344
Sample_C4B	7.46	7.32	24491	197632278	15838	2835	427886
Sample_C4C	6.76	6.65	25139	203698369	15721	2822	511367
Sample_C5A	8.78	8.63	32301	267168305	15642	2934	537570
Sample_C5B	5.71	5.60	10101	89606192	30814	2812	810636
Sample_C5C	5.76	5.66	13327	94926203	12318	2622	907762
Sample_C6A	6.53	6.43	21187	174182697	18476	2797	398012
Sample_C6B	9.86	9.69	22775	210690124	22369	3002	534576
Sample_C6C	7.12	7.03	23545	209758755	20261	2955	477265
Sample_C7A	6.71	6.59	16150	135237611	16692	2942	513072
Sample_C7B	6.53	6.44	16711	132313563	15682	2795	487303
Sample_C7C	5.82	5.72	9467	85412901	21881	2907	531694

Sample_C8A	6.35	5.39	17358	145086100	17287	2857	589334
Sample_C8B	8.01	7.89	21083	171882388	16589	2835	518489
Sample_C8C	6.02	5.92	19727	188947650	24379	3051	631233
Sample_C9A	7.57	7.43	15364	181347127	39148	3548	759225
Sample_C9B	7.40	7.25	13716	141314666	34928	3075	418923
Sample_C9C	7.85	7.75	11812	114473846	25387	3108	414054
Sample_C10A	8.62	8.44	32037	273916895	19230	2885	607318
Sample_C10B	6.53	6.48	13571	120705521	21144	2938	609179
Sample_C10C	7.60	7.46	22639	161269981	11435	2693	522063
Sample_C11A	7.47	7.40	18492	178557786	23851	3096	705957
Sample_C11B	5.48	5.43	24674	202424270	16860	2832	700562
Sample_C11C	8.19	8.10	17786	152571418	21209	2840	706417
Sample_C12A	6.28	6.23	24335	188292709	13324	2840	516307
Sample_C12B	7.32	7.22	18130	157020811	19211	2900	546119
Sample_C12C	8.57	8.42	24236	213253342	18897	2980	489255
Sample_C13A	6.64	6.56	12623	144980888	31354	3636	489148
Sample_C13B	6.19	6.10	10087	121978809	34466	3796	354745
Sample_C13C	6.10	5.99	11357	113373326	30310	3076	409731
Sample_C14A	6.24	6.09	20914	168934471	15190	2873	492714
Sample_C14B	6.06	5.99	8989	88297145	29615	3036	438640
Sample_C14C	8.33	8.17	18050	151909837	16325	2935	507677

Table S3. Clinical indicators for asthma measured before, during, and after the trail (n=55)

	Day 0					Day 30					Day 90				
	**Result statistics**				**Corrected P-value, T-test**	**Result statistics**				**Corrected P-value, T-test**	**Result statistics**				**Corrected P-value, T-test**
Clinical indicators for asthma	Mean Probiotic group	Mean Placebo group	SD Probiotic group	SD Placebo group	Probiotic vs Placebo	Mean Probiotic group	Mean Placebo group	SD Probiotic group	SD Placebo group	Probiotic vs Placebo	Mean Probiotic group	Mean Placebo group	SD Probiotic group	SD Placebo group	Probiotic vs Placebo
Asthma Control Test score	19.28	19.96	4.85	4.76	1.00	21.89	21.38	3.53	4.29	1.00	23.13	20.81	1.86	4.09	0.02
Alveolar nitric oxide concentration (ppb)	6.19	12.74	3.08	9.05	0.37	3.69	8.66	2.21	9.27	0.04	3.59	10.36	2.36	8.87	0.00
Fractional exhaled nitric oxide (ppb)	50.31	60.42	50.29	59.47	1.00	27.59	40.96	23.07	34.18	0.05	35.03	46.69	21.01	37.59	1.00
Forced vital capacity (%)	111.97	109.31	14.52	21.54	0.89	113.62	109.88	15.09	15.31	1.00	114.03	111.62	16.42	24.14	1.00
Forced expiratory volume in 1 second (%)	88.55	88.65	25.40	23.17	1.00	90.45	88.85	24.44	17.23	1.00	88.41	88.92	25.27	23.82	0.99
FEV1/FVC (%)	76.41	79.08	18.72	13.72	0.99	77.48	79.15	18.88	12.99	1.00	75.59	77.77	19.37	13.70	1.00
Peak expiratory flow (%)	91.66	83.19	32.21	22.67	1.00	94.72	87.92	30.50	21.23	0.99	96.21	88.58	36.65	23.99	0.99
Immunoglobulin E (IU/mL)	229.02	171.10	260.50	181.44	1.00	224.12	225.15	269.02	228.79	1.00	222.58	222.73	253.20	233.62	1.00
Eosinophils (%)	3.65	3.41	3.95	3.15	1.00	3.06	3.72	2.71	2.78	1.00	3.43	4.29	2.50	3.99	1.00
Total Lymphocyte Count (10^9/L)	2.08	1.90	0.52	0.54	1.00	1.84	1.97	0.50	0.52	1.00	1.87	1.98	0.53	0.51	1.00

Table S4. Genomic information of 2601 high-quality metagenome-assembled genomes (MAGs)

MAG ID	Completeness (%)	Contamination (%)	Number of contigs	GC content (%)	Checkm lineage	N50 length (bp)	Genome size (Mbp)
Sample_T5C_bin.26	98.65	0.34	42	39	Clostridiales	39	2.11
Sample_T5C_bin.27	97.95	0.68	230	56.3	Clostridiales	56.3	2.57
Sample_T5C_bin.25	83.18	1.03	389	46.3	Bacteroidales	46.3	3.00
Sample_T5C_bin.22	99.27	0.00	15	41.6	Lachnospiraceae	41.6	2.71
Sample_T5C_bin.28	95.67	0.48	102	60.3	Bacteroidetes	60.3	2.57
Sample_T5C_bin.29	92.41	3.37	34	26.7	Bacteria	26.7	1.31
Sample_C1B_bin.33	80.86	1.01	23	43.4	Clostridiales	43.4	2.27
Sample_C1B_bin.31	99.30	1.17	358	58	Clostridiales	58	3.12
Sample_C1B_bin.30	99.32	0.34	99	36.6	Clostridiales	36.6	2.09
Sample_C1B_bin.35	84.90	0.56	207	24.9	Bacteria	24.9	1.54
Sample_C1B_bin.39	98.91	3.12	69	43.1	Lachnospiraceae	43.1	3.28
Sample_C10B_bin.6	94.92	0.97	87	41.9	Lachnospiraceae	41.9	2.92
Sample_C14A_bin.39	98.44	0.00	37	36.7	Clostridiales	36.7	2.97
Sample_C6B_bin.38	94.17	1.33	61	42.7	Lachnospiraceae	42.7	3.36
Sample_C14A_bin.33	93.84	1.38	77	60.6	Clostridiales	60.6	2.30
Sample_C14A_bin.32	97.04	0.63	57	48	Clostridiales	48	3.10
Sample_C14A_bin.31	90.36	0.89	228	33.1	Clostridiales	33.1	2.34
Sample_C14A_bin.30	99.25	0.12	146	54.2	Actinobacteria	54.2	1.67
Sample_C14A_bin.37	93.24	0.57	170	62.9	Bifidobacteriaceae	62.9	1.92
Sample_C6B_bin.31	90.55	0.22	307	40.5	Bacteroidales	40.5	2.81
Sample_C6B_bin.32	86.74	3.80	47	41.4	Clostridiales	41.4	2.56
Sample_C14A_bin.34	91.49	0.68	219	56.4	Clostridiales	56.4	2.26
Sample_T5A_bin.19	94.17	1.49	97	40.7	Bacteroidales	40.7	3.41
Sample_T5A_bin.18	93.35	0.63	170	49.3	Clostridiales	49.3	2.42
Sample_C8C_bin.28	98.65	0.00	39	41.8	Clostridiales	41.8	2.79
Sample_C8C_bin.26	89.62	2.02	412	64.1	Actinobacteria	64.1	2.09
Sample_C8C_bin.27	97.46	0.32	151	41.8	Clostridiales	41.8	3.03
Sample_C8C_bin.24	96.41	0.95	80	48.5	Clostridiales	48.5	2.76
Sample_C8C_bin.25	85.19	1.01	407	36.4	Clostridiales	36.4	2.67
Sample_C8C_bin.22	99.30	0.93	484	58.2	Clostridiales	58.2	3.00
Sample_C8C_bin.23	81.44	1.51	82	41.5	Clostridiales	41.5	2.37
Sample_T5A_bin.13	99.40	0.59	76	59.4	Deltaproteobacteria	59.4	4.34
Sample_C8C_bin.21	98.65	0.00	182	44.1	Clostridiales	44.1	2.80
Sample_C8A_bin.15	98.65	0.34	165	36.2	Clostridiales	36.2	3.05
Sample_C8A_bin.14	90.49	0.29	260	61	Clostridiales	61	1.72

Sample_C8A_bin.17	99.05	1.27	128	31.3	Firmicutes	31.3	2.40
Sample_C8A_bin.10	93.33	0.67	53	36.1	Clostridiales	36.1	2.42
Sample_C8A_bin.13	81.30	3.48	180	42.6	Bacteria	42.6	4.55
Sample_C8A_bin.12	95.99	0.47	820	44.3	Bacteroidales	44.3	3.25
Sample_T6B_bin.40	92.40	4.11	316	44.8	Clostridiales	44.8	2.62
Sample_C10B_bin.1	95.95	2.53	28	44.2	Clostridiales	44.2	3.13
Sample_C8A_bin.19	87.44	2.04	452	48.5	Proteobacteria	48.5	2.24
Sample_T2B_bin.22	98.31	1.69	81	45.6	Prevotella	45.6	3.72
Sample_T2B_bin.23	99.25	0.23	204	45.6	Bacteroidales	45.6	3.51
Sample_T2B_bin.21	96.97	0.00	100	53.1	Clostridiales	53.1	1.99
Sample_T2B_bin.26	93.53	0.00	14	26.6	Bacteria	26.6	1.15
Sample_T2B_bin.27	96.36	1.79	58	33.5	Bacteria	33.5	2.26
Sample_T2B_bin.24	92.26	1.34	342	33.8	Clostridiales	33.8	1.88
Sample_T2B_bin.28	85.90	0.00	23	44.5	Clostridiales	44.5	2.33
Sample_T10A_bin.29	83.89	0.67	28	59.9	Clostridiales	59.9	1.77
Sample_T10A_bin.28	97.14	0.98	45	46.5	Bacteroidales	46.5	4.13
Sample_T15A_bin.7	99.11	0.00	50	41.8	Lachnospiraceae	41.8	2.60
Sample_T10A_bin.23	94.72	0.49	321	43.5	Bacteroidales	43.5	2.85
Sample_T10A_bin.21	83.49	1.17	391	37.8	Bacteria	37.8	2.31
Sample_T10A_bin.20	99.96	0.58	390	50.7	Enterobacteriaceae	50.7	4.61
Sample_T10A_bin.27	91.46	0.04	301	29.1	Bacteria	29.1	2.51
Sample_T10A_bin.26	81.57	0.48	27	58.6	Bacteroidetes	58.6	2.85
Sample_T10A_bin.25	97.20	0.00	115	51.5	Clostridiales	51.5	2.05
Sample_T11B_bin.15	89.24	0.77	666	45.4	Bacteroidales	45.4	4.04
Sample_T8B_bin.46	98.73	0.63	78	44.8	Clostridiales	44.8	3.08
Sample_T8B_bin.47	98.00	0.84	176	35.8	Clostridiales	35.8	2.70
Sample_T7B_bin.2	80.28	0.79	202	59.4	Clostridiales	59.4	2.14
Sample_T7B_bin.5	98.65	0.06	34	37.8	Clostridiales	37.8	2.04
Sample_T8B_bin.40	92.40	0.70	269	47.5	Clostridiales	47.5	1.49
Sample_T7B_bin.7	96.97	0.00	168	49.7	Clostridiales	49.7	2.79
Sample_T7B_bin.9	98.73	0.02	111	48.5	Firmicutes	48.5	2.04
Sample_T13C_bin.19	84.92	4.50	118	57.7	Clostridiales	57.7	2.39
Sample_T13C_bin.18	95.22	2.05	77	59.5	Bifidobacteriaceae	59.5	2.08
Sample_T13C_bin.13	94.52	0.74	105	60.4	Bacteroidetes	60.4	2.42
Sample_T13C_bin.12	97.31	0.00	90	46.1	Clostridiales	46.1	2.35
Sample_T13C_bin.11	88.81	0.41	108	45.2	Clostridiales	45.2	2.56
Sample_T13C_bin.10	97.31	1.01	243	52.2	Clostridiales	52.2	2.13
Sample_T13C_bin.17	93.28	0.42	118	40.5	Bacteroidales	40.5	2.98
Sample_T13C_bin.16	98.02	1.88	51	47	Proteobacteria	47	1.80

Sample_T13C_bin.15	97.96	0.37	52	49.6	Bacteroidales	49.6	2.70
Sample_T13C_bin.14	97.97	2.11	146	46.8	Lachnospiraceae	46.8	3.35
Sample_T5B_bin.43	92.59	0.09	85	44.5	Bacteroidales	44.5	3.04
Sample_T5B_bin.42	99.05	0.00	79	48.9	Clostridiales	48.9	2.55
Sample_T5B_bin.40	97.76	1.36	61	32.9	Clostridiales	32.9	2.91
Sample_T16B_bin.13	89.45	2.71	76	47	Lachnospiraceae	47	2.57
Sample_T6A_bin.28	93.64	4.46	189	46.9	Lachnospiraceae	46.9	3.08
Sample_T3A_bin.36	99.38	1.50	37	43.7	Selenomonadales	43.7	2.36
Sample_T2C_bin.3	81.87	0.00	234	44.5	Clostridiales	44.5	2.14
Sample_T6A_bin.22	92.12	1.65	36	41.7	Lachnospiraceae	41.7	3.10
Sample_C2A_bin.20	99.03	1.93	80	45.9	Lachnospiraceae	45.9	3.33
Sample_T14C_bin.27	98.63	0.68	247	56.2	Clostridiales	56.2	2.53
Sample_T2C_bin.7	82.85	1.68	302	59.7	Clostridiales	59.7	1.95
Sample_T6A_bin.25	96.37	0.68	260	56.4	Clostridiales	56.4	2.37
Sample_C1C_bin.40	87.12	1.01	296	37.1	Clostridiales	37.1	2.51
Sample_T12A_bin.58	84.34	1.34	292	56.1	Clostridiales	56.1	2.92
Sample_C1C_bin.43	99.32	1.01	38	40.7	Clostridiales	40.7	2.08
Sample_T12A_bin.59	90.69	0.96	34	59.2	Bacteroidetes	59.2	2.68
Sample_C3C_bin.14	91.94	2.11	28	45.7	Prevotella	45.7	2.86
Sample_C3C_bin.15	97.86	0.73	28	37.7	Clostridiales	37.7	2.44
Sample_C3C_bin.16	98.24	0.00	77	41.8	Lachnospiraceae	41.8	2.52
Sample_C5A_bin.66	99.42	0.00	60	45	Bacteroidales	45	4.72
Sample_C3C_bin.10	94.29	3.36	121	60.8	Clostridiales	60.8	2.20
Sample_C3C_bin.11	97.58	1.95	32	46.7	Lachnospiraceae	46.7	2.96
Sample_C3C_bin.12	99.98	1.70	264	43.6	Selenomonadales	43.6	2.58
Sample_C3C_bin.13	84.85	0.11	126	33.2	Clostridiales	33.2	2.21
Sample_C3C_bin.18	94.60	4.11	69	41.1	Lachnospiraceae	41.1	3.10
Sample_C3C_bin.19	94.60	0.34	99	41.9	Clostridiales	41.9	2.66
Sample_C1C_bin.44	92.59	1.34	216	49.4	Clostridiales	49.4	1.93
Sample_C6B_bin.37	90.60	0.78	48	62.5	Clostridiales	62.5	1.70
Sample_C6B_bin.33	98.22	1.45	105	41.5	Lachnospiraceae	41.5	2.82
Sample_T16B_bin.1	90.93	4.03	193	37.2	Clostridiales	37.2	2.26
Sample_T16B_bin.3	95.30	0.00	45	38.7	Clostridiales	38.7	2.69
Sample_T16B_bin.2	85.40	0.66	20	41.9	Clostridiales	41.9	2.85
Sample_T16B_bin.8	99.66	0.07	71	50.9	Enterobacteriaceae	50.9	4.51
Sample_C6A_bin.6	98.59	0.68	231	56.2	Clostridiales	56.2	2.55
Sample_C6A_bin.5	87.63	1.21	166	47.7	Lachnospiraceae	47.7	2.44
Sample_C6A_bin.2	81.73	1.74	110	45.9	Bacteroidales	45.9	3.37
Sample_C6A_bin.1	90.91	1.23	184	59.2	Bacteroidetes	59.2	2.79

Sample_C12B_bin.28	96.90	1.65	142	33.3	Bacteria	33.3	2.57
Sample_C12B_bin.27	88.25	1.74	206	60.3	Clostridiales	60.3	1.90
Sample_C12B_bin.26	98.65	0.00	21	38	Clostridiales	38	1.91
Sample_C12B_bin.25	100.00	0.81	32	59.8	Actinobacteria	59.8	2.22
Sample_C12B_bin.24	99.32	0.00	17	41.3	Clostridiales	41.3	2.66
Sample_C12B_bin.23	96.99	4.43	130	41.9	Bacteroidales	41.9	4.68
Sample_C12B_bin.22	99.21	0.97	50	43.3	Lachnospiraceae	43.3	3.10
Sample_C12B_bin.21	93.95	1.45	132	46.6	Clostridiales	46.6	2.51
Sample_C12B_bin.20	98.07	1.76	48	54.5	Bacteroidetes	54.5	2.36
Sample_C1A_bin.18	82.44	3.92	378	60.3	Bifidobacteriaceae	60.3	1.87
Sample_C1A_bin.14	89.73	2.66	153	46.8	Bacteroidales	46.8	4.49
Sample_T3B_bin.8	92.92	3.87	609	42.1	Bacteria	42.1	3.77
Sample_C1A_bin.16	97.98	0.00	199	50	Clostridiales	50	2.82
Sample_C1A_bin.17	84.52	0.82	34	43.6	Lachnospiraceae	43.6	2.34
Sample_C1A_bin.10	85.22	2.17	66	45.1	Lachnospiraceae	45.1	2.37
Sample_C1A_bin.11	99.98	1.50	172	43.8	Selenomonadales	43.8	2.31
Sample_T3B_bin.9	100.00	0.00	123	55.2	Proteobacteria	55.2	2.86
Sample_T5A_bin.16	98.97	0.00	57	47.1	Clostridiales	47.1	3.02
Sample_T5A_bin.11	93.87	0.80	24	41.4	Bacteroidales	41.4	4.57
Sample_T2A_bin.60	96.26	1.05	138	46.5	Bacteroidales	46.5	4.35
Sample_T5A_bin.10	98.24	0.00	59	43.4	Lachnospiraceae	43.4	2.63
Sample_T18B_bin.22	98.85	2.75	239	41.7	Bacteroides	41.7	6.75
Sample_T18B_bin.23	87.24	1.87	107	44.3	Bacteroidales	44.3	3.12
Sample_T7A_bin.8	92.76	0.00	45	37.4	Clostridiales	37.4	2.63
Sample_T18B_bin.26	94.33	4.02	274	56.5	Clostridiales	56.5	2.34
Sample_T18B_bin.24	93.47	0.53	428	46.3	Bacteroidales	46.3	2.88
Sample_T18B_bin.25	91.05	2.53	399	41	Clostridiales	41	3.15
Sample_T18B_bin.28	95.30	0.00	35	49.4	Clostridiales	49.4	2.28
Sample_T18B_bin.29	99.19	0.00	110	63.3	Actinobacteria	63.3	2.84
Sample_C11A_bin.21	91.33	3.16	227	44	Clostridiales	44	3.00
Sample_C11A_bin.20	91.61	0.00	85	41.6	Lachnospiraceae	41.6	2.80
Sample_C11A_bin.23	97.31	0.00	92	46.4	Clostridiales	46.4	2.44
Sample_C11A_bin.22	92.14	4.08	112	56	Clostridiales	56	3.24
Sample_C11A_bin.25	98.32	0.34	60	38.6	Clostridiales	38.6	2.09
Sample_C11A_bin.24	91.69	3.16	62	42.2	Bacteroidales	42.2	3.24
Sample_C11A_bin.26	99.51	0.96	30	54.3	Bacteroidetes	54.3	2.17
Sample_C11A_bin.29	99.32	1.01	53	40.9	Clostridiales	40.9	2.12
Sample_C11A_bin.28	97.10	0.36	65	42.8	Lachnospiraceae	42.8	3.54
Sample_T3B_bin.2	89.93	0.67	290	61	Clostridiales	61	2.16

Sample_T7A_bin.2	91.66	0.24	46	60	Bacteroidetes	60	2.42
Sample_T3B_bin.3	93.95	0.67	59	34.1	Clostridiales	34.1	2.52
Sample_T7A_bin.7	92.13	1.85	24	27.1	Bacteria	27.1	1.49
Sample_T12A_bin.31	97.09	0.00	38	44.6	Clostridiales	44.6	2.25
Sample_T7A_bin.4	98.10	1.58	94	41	Clostridiales	41	3.59
Sample_T15C_bin.19	99.51	0.00	79	41.5	Lachnospiraceae	41.5	3.05
Sample_C6B_bin.49	95.97	0.00	34	58.5	Clostridiales	58.5	2.07
Sample_T5C_bin.18	91.60	0.38	83	42.1	Bacteroidales	42.1	3.85
Sample_T5C_bin.13	97.55	1.28	271	58.3	Clostridiales	58.3	2.84
Sample_T5C_bin.11	90.19	1.21	267	43.5	Lachnospiraceae	43.5	2.83
Sample_T5C_bin.10	90.93	0.69	144	61.9	Clostridiales	61.9	1.93
Sample_T5C_bin.17	95.69	0.00	73	47.2	Bacteria	47.2	1.72
Sample_T5C_bin.16	97.98	0.00	47	37.8	Clostridiales	37.8	2.07
Sample_T5C_bin.14	90.93	0.67	433	37.6	Clostridiales	37.6	2.29
Sample_T15C_bin.14	83.14	3.29	218	60	Clostridiales	60	1.71
Sample_T15C_bin.17	97.59	1.92	27	54.5	Bacteroidetes	54.5	2.07
Sample_C1B_bin.24	96.20	1.58	251	40.9	Clostridiales	40.9	3.63
Sample_C1B_bin.25	98.25	1.90	86	44.5	Clostridiales	44.5	2.62
Sample_C1B_bin.26	98.26	0.38	95	45	Bacteroidales	45	4.64
Sample_C1B_bin.21	99.32	3.36	29	40.7	Clostridiales	40.7	2.14
Sample_C1B_bin.22	92.69	4.49	28	26.2	Bacteria	26.2	1.31
Sample_C1B_bin.23	100.00	0.23	130	56.3	Bifidobacteriaceae	56.3	2.22
Sample_T15C_bin.11	97.31	0.06	49	58.4	Clostridiales	58.4	2.37
Sample_C3C_bin.5	84.88	0.89	140	59	Bacteria	59	3.09
Sample_C14A_bin.48	94.73	0.67	91	61.1	Clostridiales	61.1	2.39
Sample_C14A_bin.49	95.36	1.20	76	60.3	Bacteroidetes	60.3	2.54
Sample_C14A_bin.46	92.59	1.45	142	47	Lachnospiraceae	47	3.12
Sample_C14A_bin.47	97.38	1.62	74	59.3	Bifidobacteriaceae	59.3	2.16
Sample_C14A_bin.44	87.75	2.50	193	62.3	Clostridiales	62.3	1.49
Sample_C14A_bin.42	81.31	0.49	170	27	Clostridiales	27	2.18
Sample_C14A_bin.40	98.73	0.02	104	48.3	Firmicutes	48.3	2.11
Sample_C14A_bin.41	97.46	0.81	82	44.6	Clostridiales	44.6	3.06
Sample_T14A_bin.4	93.28	1.34	23	57	Clostridiales	57	3.36
Sample_T14A_bin.5	95.19	0.72	54	59.3	Bacteroidetes	59.3	2.65
Sample_C8C_bin.11	95.19	0.62	61	40.8	Bacteroidales	40.8	3.25
Sample_C8C_bin.10	93.79	0.23	85	57	Bifidobacteriaceae	57	2.01
Sample_C8C_bin.17	93.17	3.13	248	38.4	Clostridiales	38.4	2.23
Sample_C8C_bin.16	97.84	0.00	43	51	Bacteria	51	1.93
Sample_C8C_bin.15	94.84	1.63	247	40.9	Clostridiales	40.9	2.30

Sample_C8C_bin.14	94.37	1.84	449	43.4	Bacteroidales	43.4	2.76
Sample_T6B_bin.35	84.49	3.16	384	42.8	Lachnospiraceae	42.8	2.68
Sample_T10C_bin.3	84.35	0.67	411	37.3	Clostridiales	37.3	2.25
Sample_C8C_bin.18	94.94	1.75	138	45.9	Lachnospiraceae	45.9	2.79
Sample_T10C_bin.6	99.21	1.76	46	43.2	Lachnospiraceae	43.2	3.13
Sample_T10C_bin.7	96.71	0.14	66	46	Bacteroidales	46	3.26
Sample_T6B_bin.33	95.63	1.36	106	37.3	Clostridiales	37.3	2.28
Sample_T10C_bin.5	98.65	0.00	41	41.9	Clostridiales	41.9	2.67
Sample_C9A_bin.14	97.58	0.60	56	42.7	Lachnospiraceae	42.7	3.53
Sample_C9A_bin.15	96.61	1.93	52	47	Lachnospiraceae	47	3.06
Sample_C1C_bin.3	97.31	2.01	60	53	Clostridiales	53	2.28
Sample_C9A_bin.16	95.94	0.96	35	59	Bacteroidetes	59	2.80
Sample_T2B_bin.17	99.07	1.27	134	31.4	Firmicutes	31.4	2.40
Sample_T2B_bin.16	93.90	0.89	228	59.3	Clostridiales	59.3	1.99
Sample_T2B_bin.15	88.03	0.85	27	32.7	Bacteria	32.7	1.97
Sample_T2B_bin.14	96.98	0.90	26	43.4	Selenomonadales	43.4	2.47
Sample_T2B_bin.13	85.94	1.27	31	45.3	Clostridiales	45.3	2.06
Sample_C6B_bin.35	99.32	1.01	36	40.5	Clostridiales	40.5	2.28
Sample_T2B_bin.11	97.98	0.00	163	33.1	Clostridiales	33.1	2.51
Sample_T7B_bin.16	98.65	0.00	19	44.3	Clostridiales	44.3	2.68
Sample_T2B_bin.19	92.69	0.39	178	60.5	Bifidobacteriaceae	60.5	1.81
Sample_C12C_bin.48	95.63	4.87	180	59.4	Clostridiales	59.4	1.86
Sample_C12C_bin.49	97.21	1.34	81	36	Clostridiales	36	2.56
Sample_C9A_bin.12	92.61	1.58	129	46.7	Bacteroidales	46.7	4.09
Sample_C12C_bin.43	91.28	1.27	88	42.1	Lachnospiraceae	42.1	2.15
Sample_C12C_bin.40	98.63	0.00	255	43.1	Clostridiales	43.1	2.53
Sample_C9A_bin.13	94.92	0.00	53	44.4	Clostridiales	44.4	2.62
Sample_C12C_bin.47	85.67	0.44	92	46.2	Bacteroidales	46.2	2.78
Sample_C12C_bin.44	98.07	1.03	4	45.2	Bacteroidales	45.2	4.01
Sample_T8C_bin.18	96.59	0.86	71	63.6	Clostridiales	63.6	2.33
Sample_C6B_bin.36	87.08	2.24	122	58.9	Clostridiales	58.9	1.99
Sample_T8C_bin.12	95.78	0.51	369	59.4	Bacteroidetes	59.4	2.76
Sample_T8C_bin.10	94.96	1.34	31	60.3	Clostridiales	60.3	2.55
Sample_T8C_bin.16	92.13	0.90	91	42	Bacteroides	42	4.48
Sample_T8C_bin.17	99.40	0.00	76	61.1	Deltaproteobacteria	61.1	3.59
Sample_T8C_bin.15	98.38	1.08	89	53.1	Clostridia	53.1	2.06
Sample_T17B_bin.6	95.38	0.79	66	44	Clostridiales	44	3.57
Sample_T17B_bin.7	91.42	0.63	31	46.9	Clostridiales	46.9	2.90
Sample_T17B_bin.4	98.83	1.50	162	44	Selenomonadales	44	2.26

Sample_T17B_bin.5	97.53	0.00	65	38.6	Clostridiales	38.6	2.54
Sample_T17B_bin.2	99.55	0.00	35	60.4	Bifidobacteriaceae	60.4	1.94
Sample_T17B_bin.3	97.01	0.19	48	40.6	Bacteroidales	40.6	3.66
Sample_T4C_bin.15	92.69	1.19	61	60.7	Clostridiales	60.7	2.17
Sample_T17B_bin.1	99.60	0.00	109	38.6	Selenomonadales	38.6	2.10
Sample_T17B_bin.8	91.61	0.16	297	44.3	Clostridiales	44.3	2.55
Sample_T17B_bin.9	97.75	0.30	89	57.7	Enterobacteriaceae	57.7	5.14
Sample_C10A_bin.42	91.12	1.61	117	59.6	Actinobacteria	59.6	2.31
Sample_C10A_bin.43	92.41	4.13	64	60.4	Bifidobacteriaceae	60.4	2.05
Sample_C10A_bin.40	89.43	1.20	44	41.9	Lachnospiraceae	41.9	1.97
Sample_T7C_bin.2	96.20	1.27	44	44.3	Clostridiales	44.3	2.91
Sample_C10A_bin.46	90.93	2.42	208	47.3	Bacteroidales	47.3	3.62
Sample_T7C_bin.4	98.65	0.00	21	44.3	Clostridiales	44.3	2.65
Sample_T7C_bin.7	91.79	0.00	98	40.5	Bacteroidales	40.5	2.91
Sample_T15A_bin.18	97.11	0.96	26	54.7	Bacteroidetes	54.7	2.05
Sample_T15A_bin.17	94.85	3.10	90	44.7	Bacteroidales	44.7	3.63
Sample_T15A_bin.16	94.63	0.60	52	44.8	Lachnospiraceae	44.8	2.68
Sample_C10A_bin.48	87.44	3.24	294	56.8	Clostridiales	56.8	1.99
Sample_T15A_bin.14	85.73	0.67	31	60	Clostridiales	60	1.92
Sample_T15A_bin.13	98.65	0.34	64	39	Clostridiales	39	2.36
Sample_T15A_bin.12	99.03	2.13	79	41.7	Lachnospiraceae	41.7	3.50
Sample_T15A_bin.11	84.61	0.68	114	58.9	Clostridiales	58.9	1.75
Sample_T4B_bin.33	98.07	0.00	76	46.6	Bacteroidales	46.6	4.12
Sample_T4B_bin.32	81.91	3.97	305	60	Clostridiales	60	1.80
Sample_T4B_bin.36	97.97	2.03	68	45.4	Prevotella	45.4	3.18
Sample_T15A_bin.6	80.61	1.53	80	58.8	Clostridiales	58.8	1.99
Sample_T4B_bin.39	97.66	2.24	45	43.6	Lachnospiraceae	43.6	2.82
Sample_T15A_bin.4	98.63	0.00	66	42.7	Clostridiales	42.7	2.82
Sample_T15A_bin.3	85.94	1.27	115	48.8	Clostridiales	48.8	2.49
Sample_T1C_bin.10	98.73	0.02	285	48.4	Firmicutes	48.4	2.11
Sample_T12A_bin.61	89.93	1.68	94	59.3	Clostridiales	59.3	2.15
Sample_T12A_bin.60	86.14	0.96	84	60.7	Bacteroidetes	60.7	2.30
Sample_T11B_bin.44	88.11	0.48	55	59.5	Bacteroidetes	59.5	2.39
Sample_T11B_bin.41	97.98	1.98	62	61.5	Clostridiales	61.5	3.59
Sample_T11B_bin.43	81.35	1.70	64	38.5	Clostridiales	38.5	2.34
Sample_T11B_bin.42	95.60	1.49	306	46.3	Bacteroidales	46.3	3.62
Sample_C4A_bin.1	96.37	2.82	20	46.7	Lachnospiraceae	46.7	3.06
Sample_C10A_bin.20	100.00	0.11	18	29.5	Bacteria	29.5	1.54
Sample_C4A_bin.3	84.58	2.34	87	41.7	Bacteroidales	41.7	3.94

Sample_C4A_bin.2	100.00	1.50	81	43.8	Selenomonadales	43.8	2.31
Sample_C4A_bin.5	94.23	0.00	187	49.2	Clostridiales	49.2	2.43
Sample_T10B_bin.16	98.30	0.38	66	49.9	Bacteroidales	49.9	2.66
Sample_T10B_bin.15	95.94	1.69	59	45.2	Prevotella	45.2	3.27
Sample_C5A_bin.2	97.56	1.90	30	44.8	Clostridiales	44.8	1.89
Sample_C4A_bin.8	86.49	0.37	105	47.7	Bacteroidales	47.7	3.44
Sample_T10B_bin.19	99.32	0.67	20	41.2	Clostridiales	41.2	2.61
Sample_C5A_bin.1	93.70	1.39	25	57.4	Clostridiales	57.4	2.14
Sample_C5A_bin.6	99.36	0.63	38	44.5	Clostridiales	44.5	2.71
Sample_C10A_bin.22	91.58	0.17	187	59.3	Clostridiales	59.3	2.51
Sample_C5A_bin.4	97.31	0.00	194	50.1	Clostridiales	50.1	2.79
Sample_C5A_bin.5	94.92	0.00	518	44.9	Lachnospiraceae	44.9	2.56
Sample_T10B_bin.1	94.55	0.70	121	60.8	Clostridiales	60.8	2.19
Sample_C10A_bin.26	80.44	0.00	192	61.4	Clostridiales	61.4	1.80
Sample_C10C_bin.9	97.98	2.01	81	38.2	Clostridiales	38.2	2.74
Sample_C10C_bin.7	84.47	0.32	152	43	Lachnospiraceae	43	1.62
Sample_C10C_bin.5	88.19	1.40	159	58.9	Clostridiales	58.9	1.81
Sample_C10C_bin.4	97.98	0.67	33	36	Clostridiales	36	2.66
Sample_C10C_bin.3	82.22	3.63	91	60.2	Bifidobacteriaceae	60.2	1.77
Sample_C10C_bin.2	96.46	0.58	54	43.5	Lachnospiraceae	43.5	2.62
Sample_C10C_bin.1	99.09	2.08	133	56.2	Clostridiales	56.2	2.50
Sample_C2C_bin.11	97.04	0.67	22	52.4	Clostridiales	52.4	2.35
Sample_T2B_bin.2	97.84	0.07	11	43.4	Bacteria	43.4	3.32
Sample_C2B_bin.53	97.31	0.00	173	37.8	Clostridiales	37.8	1.88
Sample_T8B_bin.43	99.30	1.17	472	57.8	Clostridiales	57.8	3.16
Sample_C2C_bin.17	98.65	0.67	52	37.5	Clostridiales	37.5	2.34
Sample_T8B_bin.41	97.19	1.03	123	54.7	Clostridiales	54.7	2.58
Sample_C2C_bin.19	82.88	0.17	64	61.3	Clostridiales	61.3	1.93
Sample_C11C_bin.41	99.15	4.22	55	44.7	Prevotella	44.7	3.92
Sample_C11C_bin.40	87.11	1.36	152	45.2	Clostridiales	45.2	2.27
Sample_T14A_bin.17	93.19	1.74	80	60.7	Clostridiales	60.7	2.19
Sample_T15A_bin.23	98.01	0.00	18	42.2	Bacteria	42.2	4.34
Sample_T11C_bin.51	91.53	1.27	242	49.1	Clostridiales	49.1	2.44
Sample_T11C_bin.50	93.96	1.04	35	43.9	Lachnospiraceae	43.9	2.57
Sample_T18B_bin.31	97.31	0.00	44	53	Clostridiales	53	1.96
Sample_T18B_bin.30	99.78	1.50	13	44.1	Selenomonadales	44.1	2.23
Sample_T18B_bin.33	92.97	1.54	54	55.3	Bacteroidetes	55.3	2.04
Sample_T18B_bin.32	97.31	0.34	112	36.3	Clostridiales	36.3	2.55
Sample_C3C_bin.42	96.64	0.00	242	53.3	Clostridiales	53.3	1.95

Sample_C11A_bin.14	95.53	0.19	105	48.3	Bacteroidales	48.3	4.01
Sample_C11A_bin.15	97.19	1.12	228	30.3	Bacteria	30.3	2.02
Sample_C11A_bin.16	98.13	0.62	67	62.5	Proteobacteria	62.5	2.37
Sample_C11A_bin.17	87.75	0.00	35	60.2	Clostridiales	60.2	2.25
Sample_C11A_bin.10	89.39	0.02	46	59.9	Deltaproteobacteria	59.9	3.46
Sample_C11A_bin.11	95.30	0.00	50	45.9	Clostridiales	45.9	2.94
Sample_C11A_bin.12	97.98	1.01	42	44.5	Clostridiales	44.5	1.93
Sample_C11A_bin.13	98.70	1.30	38	49.6	Bacteroidales	49.6	2.73
Sample_C11A_bin.19	99.23	0.38	103	45.1	Bacteroidales	45.1	4.55
Sample_T2C_bin.17	97.46	0.63	70	44.1	Clostridiales	44.1	3.37
Sample_C6B_bin.28	98.95	1.17	99	58.1	Clostridiales	58.1	2.94
Sample_C5B_bin.20	93.14	0.12	63	46.4	Bacteroidales	46.4	3.22
Sample_T1B_bin.10	98.73	0.02	34	48.4	Firmicutes	48.4	2.08
Sample_C4C_bin.14	95.67	2.13	70	59.1	Bacteroidetes	59.1	2.80
Sample_T2C_bin.21	88.03	0.85	27	32.7	Bacteria	32.7	1.96
Sample_T2C_bin.20	99.03	2.40	25	54.5	Bacteroidetes	54.5	2.32
Sample_T2C_bin.23	98.65	0.00	180	34	Clostridiales	34	2.02
Sample_T2C_bin.22	99.32	0.00	57	40.9	Clostridiales	40.9	2.69
Sample_T2C_bin.25	93.82	0.00	67	26.8	Bacteria	26.8	1.17
Sample_T2C_bin.27	87.91	3.02	173	57.2	Clostridiales	57.2	2.08
Sample_T2C_bin.26	98.32	0.00	67	45	Clostridiales	45	2.52
Sample_T2C_bin.29	96.64	0.00	33	49.9	Clostridiales	49.9	2.36
Sample_T2C_bin.28	96.42	0.00	58	57.3	Clostridiales	57.3	2.18
Sample_C14A_bin.51	97.98	2.01	143	37.5	Clostridiales	37.5	3.31
Sample_C14A_bin.50	96.84	1.54	272	55	Clostridiales	55	2.49
Sample_C11C_bin.4	100.00	1.20	400	47	Selenomonadales	47	2.26
Sample_T6B_bin.28	95.14	1.90	49	47.7	Clostridiales	47.7	3.13
Sample_T6B_bin.29	97.16	0.00	38	49.9	Bacteroidales	49.9	2.54
Sample_C8A_bin.33	96.51	0.02	35	48.5	Firmicutes	48.5	2.10
Sample_C8A_bin.31	98.51	0.23	128	45.5	Bacteroidales	45.5	3.31
Sample_C8A_bin.30	95.32	0.12	91	41.9	Lachnospiraceae	41.9	2.33
Sample_T6B_bin.22	96.64	1.34	61	58.7	Clostridiales	58.7	2.09
Sample_T6B_bin.23	99.98	1.50	28	43.4	Selenomonadales	43.4	2.42
Sample_T6B_bin.20	97.42	1.01	170	56.4	Clostridiales	56.4	2.72
Sample_T6B_bin.21	99.05	0.00	75	29.8	Bacteria	29.8	2.62
Sample_T11B_bin.13	97.46	0.00	106	41.7	Clostridiales	41.7	3.03
Sample_C12C_bin.59	88.36	0.00	55	56.8	Clostridiales	56.8	2.02
Sample_C12C_bin.58	94.07	2.35	87	57	Clostridiales	57	2.13
Sample_T10A_bin.49	86.91	2.64	233	43.5	Clostridiales	43.5	2.76

Sample_C12C_bin.51	97.90	0.00	67	41.5	Lachnospiraceae	41.5	2.62
Sample_T10A_bin.44	81.81	0.67	51	37.6	Clostridiales	37.6	2.18
Sample_C12C_bin.53	91.96	3.69	73	57.5	Clostridiales	57.5	2.30
Sample_T10A_bin.46	97.30	1.05	343	45.3	Bacteroidales	45.3	4.36
Sample_T10A_bin.41	97.31	1.01	141	44.5	Clostridiales	44.5	1.96
Sample_T10A_bin.40	95.11	1.29	65	43.3	Lachnospiraceae	43.3	2.70
Sample_T10A_bin.43	91.87	0.96	368	60.9	Firmicutes	60.9	2.71
Sample_T10A_bin.42	95.91	0.00	319	58.7	Clostridiales	58.7	3.02
Sample_C13B_bin.9	99.80	1.35	101	43.9	Selenomonadales	43.9	2.43
Sample_T8C_bin.29	99.34	2.06	104	49.2	Clostridiales	49.2	6.46
Sample_T14B_bin.8	84.48	0.32	269	38.4	Clostridiales	38.4	2.37
Sample_T14B_bin.7	99.32	0.31	27	42.6	Clostridiales	42.6	3.09
Sample_T8C_bin.26	91.94	1.01	42	61.5	Clostridiales	61.5	2.20
Sample_T14B_bin.5	90.88	3.47	102	42.1	Bacteroidales	42.1	3.46
Sample_C13B_bin.8	97.46	0.00	146	41.5	Clostridiales	41.5	3.59
Sample_T8C_bin.23	92.61	3.02	108	60.8	Clostridiales	60.8	2.39
Sample_T12C_bin.32	80.38	2.68	96	58.4	Bacteria	58.4	2.02
Sample_T14B_bin.1	97.51	0.62	131	62.7	Proteobacteria	62.7	2.24
Sample_T8C_bin.20	96.49	0.00	468	41.8	Lachnospiraceae	41.8	2.39
Sample_C9A_bin.44	99.03	3.13	549	54.2	Bacteroidetes	54.2	2.53
Sample_C9A_bin.43	93.94	1.53	61	57.8	Clostridiales	57.8	2.11
Sample_C9A_bin.40	99.61	0.38	95	45	Bacteroidales	45	4.11
Sample_T13A_bin.8	99.35	0.48	39	41.6	Lachnospiraceae	41.6	2.66
Sample_T5A_bin.7	99.23	0.00	46	45.3	Bacteroidales	45.3	4.15
Sample_T5A_bin.6	94.87	0.84	238	43.9	Clostridiales	43.9	2.69
Sample_T5A_bin.1	99.25	0.00	102	49.2	Clostridiales	49.2	5.95
Sample_T5A_bin.3	95.80	0.63	111	43.2	Clostridiales	43.2	3.05
Sample_T13A_bin.1	92.98	0.75	86	44.2	Bacteroidales	44.2	3.78
Sample_T13A_bin.5	80.69	1.63	25	46.2	Bacteroidales	46.2	2.52
Sample_T13A_bin.4	95.14	0.67	195	54.1	Clostridiales	54.1	1.78
Sample_T13A_bin.7	82.90	0.30	318	56.8	Selenomonadales	56.8	1.78
Sample_T13A_bin.6	95.67	0.75	75	58.9	Bacteroidetes	58.9	3.10
Sample_C10A_bin.51	88.21	3.41	205	62.1	Clostridiales	62.1	1.50
Sample_C10A_bin.50	98.55	2.17	43	43.9	Lachnospiraceae	43.9	2.76
Sample_C10A_bin.52	96.67	1.01	206	45.5	Prevotella	45.5	3.17
Sample_C10A_bin.55	93.72	2.85	339	41	Clostridiales	41	3.03
Sample_C10A_bin.54	84.22	0.92	55	53.2	Clostridiales	53.2	2.01
Sample_C10A_bin.57	80.41	2.05	233	60	Bifidobacteriaceae	60	1.92
Sample_C10A_bin.59	92.41	3.37	35	25.3	Bacteria	25.3	1.47

Sample_C10A_bin.58	93.38	0.99	29	59	Bacteroidetes	59	2.79
Sample_C11B_bin.52	96.34	0.74	70	47.6	Bacteroidales	47.6	2.67
Sample_T4B_bin.24	99.32	0.00	77	43.9	Clostridiales	43.9	2.92
Sample_T4B_bin.25	91.83	0.67	53	45.3	Clostridiales	45.3	1.64
Sample_T4B_bin.26	96.84	0.82	100	37.6	Clostridiales	37.6	2.55
Sample_T4B_bin.27	93.82	1.12	84	25.1	Bacteria	25.1	1.67
Sample_T4B_bin.21	98.76	0.04	25	56.8	Clostridiales	56.8	2.33
Sample_T10B_bin.2	98.65	0.67	212	33.2	Clostridiales	33.2	2.22
Sample_T4B_bin.28	93.55	0.22	209	33.2	Clostridiales	33.2	2.40
Sample_T4B_bin.29	97.95	0.70	213	56.2	Clostridiales	56.2	2.44
Sample_T18A_bin.8	95.50	1.90	213	41.1	Lachnospiraceae	41.1	3.45
Sample_C3A_bin.48	96.98	0.00	49	43.4	Bacteroidales	43.4	3.08
Sample_T10B_bin.6	97.27	0.72	218	56.3	Clostridiales	56.3	2.37
Sample_T11B_bin.14	84.53	0.87	220	47.3	Bacteroidales	47.3	3.35
Sample_C4C_bin.9	98.65	0.00	35	36.4	Clostridiales	36.4	1.99
Sample_T12A_bin.57	94.63	2.01	68	45.9	Clostridiales	45.9	2.24
Sample_T12A_bin.50	92.61	0.67	180	40.9	Clostridiales	40.9	2.21
Sample_T12A_bin.51	92.95	0.76	555	44.2	Clostridiales	44.2	2.35
Sample_T12A_bin.52	93.96	1.95	102	45.6	Bacteroidales	45.6	4.23
Sample_T12A_bin.53	98.60	1.17	45	58.1	Clostridiales	58.1	3.04
Sample_C4C_bin.3	97.74	3.97	70	42.3	Bacteroidales	42.3	6.01
Sample_C4C_bin.2	86.38	2.05	178	42	Bacteroides	42	3.97
Sample_C4C_bin.7	83.85	4.03	261	55.8	Clostridiales	55.8	2.11
Sample_C4C_bin.6	85.35	3.78	113	59.1	Clostridiales	59.1	1.99
Sample_C2C_bin.2	98.10	0.02	60	48.5	Firmicutes	48.5	2.13
Sample_C2C_bin.4	87.83	0.07	34	61.1	Proteobacteria	61.1	2.19
Sample_C2C_bin.7	93.79	2.85	278	59.2	Bacteroidetes	59.2	2.87
Sample_C2C_bin.6	82.15	2.41	314	45.8	Prevotella	45.8	2.57
Sample_C2C_bin.9	99.19	0.00	123	54.8	Clostridia	54.8	2.72
Sample_C2C_bin.8	85.06	0.84	319	44.3	Clostridiales	44.3	2.01
Sample_T5B_bin.25	99.13	0.00	48	48.6	Bacteroidales	48.6	3.55
Sample_T5B_bin.24	94.81	0.00	159	55.4	Clostridiales	55.4	2.27
Sample_T7A_bin.9	97.96	0.00	50	41.8	Clostridiales	41.8	2.72
Sample_T5B_bin.26	88.32	0.68	61	43.3	Bacteroidales	43.3	4.41
Sample_T5B_bin.21	99.38	0.60	24	45.5	Selenomonadales	45.5	1.92
Sample_T5B_bin.20	92.74	0.00	267	58.5	Clostridiales	58.5	3.04
Sample_T7A_bin.3	96.85	1.17	109	57.9	Clostridiales	57.9	3.09
Sample_C3A_bin.45	94.63	3.36	92	56.2	Clostridiales	56.2	2.57
Sample_T7A_bin.1	84.25	2.80	49	44.1	Clostridiales	44.1	2.27

Sample_T5B_bin.29	96.20	0.85	276	41.8	Clostridiales	41.8	3.21
Sample_T7A_bin.6	99.90	0.00	102	52.4	Firmicutes	52.4	2.55
Sample_T7A_bin.5	83.66	2.01	92	59.7	Clostridiales	59.7	1.77
Sample_C3A_bin.44	100.00	2.42	151	60	Actinobacteria	60	2.14
Sample_T14B_bin.48	97.76	1.34	79	57.3	Clostridiales	57.3	2.21
Sample_T15C_bin.15	95.51	1.76	48	43.6	Lachnospiraceae	43.6	2.78
Sample_T14B_bin.45	94.62	1.27	138	49.3	Clostridiales	49.3	2.25
Sample_T14B_bin.46	89.59	0.17	105	40.6	Clostridiales	40.6	2.44
Sample_T14B_bin.40	93.06	0.34	160	36.3	Clostridiales	36.3	1.89
Sample_T14B_bin.41	91.36	0.77	253	48.7	Lachnospiraceae	48.7	2.73
Sample_T14B_bin.42	95.76	0.96	60	60.5	Bacteroidetes	60.5	2.54
Sample_T12B_bin.7	84.07	1.38	90	40.6	Bacteroidales	40.6	2.96
Sample_C5A_bin.49	99.98	3.39	82	43.7	Selenomonadales	43.7	2.38
Sample_C5A_bin.48	98.11	1.89	76	54.2	Bacteroidales	54.2	2.48
Sample_T15A_bin.9	83.10	0.00	36	46.3	Bacteria	46.3	4.12
Sample_C5A_bin.43	91.62	3.04	35	60.8	Bacteroidetes	60.8	2.38
Sample_C5A_bin.42	96.64	0.17	271	58.9	Clostridiales	58.9	2.00
Sample_C5A_bin.41	98.65	0.02	51	37.3	Clostridiales	37.3	2.85
Sample_C5A_bin.40	80.26	0.32	48	42	Clostridiales	42	2.34
Sample_C5A_bin.47	99.32	1.01	25	40.7	Clostridiales	40.7	2.15
Sample_T15A_bin.2	98.07	0.22	182	45.2	Bacteroidales	45.2	3.95
Sample_C5A_bin.45	83.48	2.04	76	59.4	Clostridiales	59.4	1.88
Sample_C5A_bin.44	99.32	0.34	192	36.5	Clostridiales	36.5	2.13
Sample_C12A_bin.7	98.65	0.34	44	38.7	Clostridiales	38.7	2.11
Sample_C12A_bin.4	98.60	1.17	121	57.6	Clostridiales	57.6	3.11
Sample_C12A_bin.3	99.32	0.00	331	41.4	Clostridiales	41.4	2.57
Sample_C12A_bin.2	86.86	2.87	113	58.3	Clostridiales	58.3	1.77
Sample_C12A_bin.1	98.63	0.00	307	42.8	Clostridiales	42.8	2.64
Sample_C2C_bin.41	99.51	2.34	18	54.3	Bacteroidetes	54.3	2.30
Sample_C12A_bin.9	97.85	1.34	164	38	Clostridiales	38	3.71
Sample_C3A_bin.34	88.25	3.36	59	59.5	Clostridiales	59.5	2.07
Sample_C6A_bin.13	97.98	0.00	59	52.1	Clostridiales	52.1	2.42
Sample_C9B_bin.29	96.16	0.85	135	57.2	Clostridiales	57.2	2.62
Sample_C6C_bin.15	97.82	0.48	44	41.5	Lachnospiraceae	41.5	2.71
Sample_C6C_bin.14	87.75	0.86	270	44.4	Clostridiales	44.4	2.52
Sample_C6C_bin.17	98.55	0.96	58	54.8	Bacteroidetes	54.8	2.17
Sample_C6C_bin.16	96.14	0.40	169	45.5	Bacteroidales	45.5	3.89
Sample_C6C_bin.10	99.30	1.17	82	58.2	Clostridiales	58.2	2.89
Sample_C6C_bin.19	97.31	0.00	43	58.7	Clostridiales	58.7	2.04

Sample_C6A_bin.12	89.16	0.40	23	40.4	Bacteroidales	40.4	2.75
Sample_C13B_bin.6	95.53	0.67	106	51.5	Clostridiales	51.5	2.12
Sample_C13B_bin.4	86.78	0.89	223	59.3	Clostridiales	59.3	1.77
Sample_C13B_bin.3	96.43	1.49	659	48.7	Bacteroidales	48.7	3.60
Sample_C13B_bin.2	84.55	1.46	45	43.9	Lachnospiraceae	43.9	2.11
Sample_C14A_bin.10	97.95	0.68	43	55.2	Bacteria	55.2	2.82
Sample_C1C_bin.16	96.37	1.08	277	56.2	Clostridiales	56.2	2.48
Sample_C1C_bin.17	83.98	0.79	129	42.1	Lachnospiraceae	42.1	2.45
Sample_C1C_bin.14	92.15	2.38	108	59.4	Clostridiales	59.4	2.12
Sample_C1C_bin.12	84.60	0.63	54	43.7	Proteobacteria	43.7	2.06
Sample_C1C_bin.10	86.57	0.67	109	58.8	Clostridiales	58.8	2.22
Sample_C1C_bin.11	97.27	2.04	388	58.1	Clostridiales	58.1	3.50
Sample_C6B_bin.15	98.21	0.00	76	34.2	Clostridiales	34.2	2.41
Sample_C13A_bin.9	96.83	0.95	67	40.3	Clostridiales	40.3	2.59
Sample_C13A_bin.4	98.63	0.00	56	43.1	Clostridiales	43.1	2.75
Sample_C13A_bin.5	89.86	1.86	48	46.6	Bacteroidales	46.6	3.66
Sample_C13A_bin.6	99.10	0.00	116	55.3	Clostridiales	55.3	3.24
Sample_C13A_bin.7	98.99	0.65	40	44	Clostridiales	44	3.26
Sample_C13A_bin.1	80.31	1.68	49	48.3	Clostridiales	48.3	1.70
Sample_C13A_bin.3	89.65	0.67	63	33.5	Clostridiales	33.5	1.99
Sample_C14A_bin.17	94.07	0.34	147	57.4	Clostridiales	57.4	2.16
Sample_C14A_bin.16	91.79	0.00	220	46.6	Clostridiales	46.6	2.76
Sample_C14A_bin.19	86.31	3.79	432	44.9	Bacteroidales	44.9	2.74
Sample_T5C_bin.8	95.97	0.00	52	45	Clostridiales	45	2.61
Sample_T5C_bin.7	97.17	0.93	45	48.4	Bacteroidales	48.4	4.22
Sample_T5C_bin.5	98.95	1.82	49	49.5	Lachnospiraceae	49.5	3.12
Sample_T5C_bin.4	98.63	0.00	37	43	Clostridiales	43	2.52
Sample_T5C_bin.2	97.27	0.00	64	51.2	Clostridiales	51.2	2.19
Sample_T5C_bin.1	84.60	2.11	44	48.6	Proteobacteria	48.6	2.02
Sample_C14C_bin.29	97.31	0.00	11	53.2	Clostridiales	53.2	1.97
Sample_C8B_bin.29	94.34	0.65	50	44.1	Bacteroidales	44.1	3.20
Sample_C8B_bin.25	98.65	0.00	30	36.4	Clostridiales	36.4	2.11
Sample_C14C_bin.23	98.12	0.00	71	57.3	Proteobacteria	57.3	2.42
Sample_C8B_bin.26	98.51	0.23	90	45.4	Bacteroidales	45.4	3.31
Sample_C14C_bin.26	92.90	0.91	206	59.5	Clostridiales	59.5	2.81
Sample_C14C_bin.27	99.10	0.00	64	36	Clostridiales	36	2.58
Sample_C8B_bin.22	92.56	2.06	131	46.5	Bacteroidales	46.5	3.20
Sample_C4A_bin.10	95.97	0.00	64	44.1	Clostridiales	44.1	2.58
Sample_C4A_bin.13	96.41	0.34	294	33	Clostridiales	33	1.89

Sample_C4A_bin.12	98.63	0.00	214	43.1	Clostridiales	43.1	2.88
Sample_C4A_bin.14	89.69	1.58	57	41.2	Clostridiales	41.2	2.98
Sample_C4A_bin.17	97.98	0.00	12	45.6	Clostridiales	45.6	2.12
Sample_C4A_bin.16	98.65	0.67	149	41.1	Clostridiales	41.1	1.99
Sample_C4A_bin.19	84.19	0.67	170	53.6	Clostridiales	53.6	1.84
Sample_C4A_bin.18	94.67	0.84	171	36.6	Clostridiales	36.6	3.13
Sample_C14C_bin.21	91.47	0.13	232	43.7	Bacteroidales	43.7	2.88
Sample_T3C_bin.8	93.67	0.95	210	44	Clostridiales	44	3.12
Sample_C7B_bin.16	93.53	2.31	258	45.4	Lachnospiraceae	45.4	2.17
Sample_T3C_bin.5	89.38	0.73	272	59.5	Clostridiales	59.5	1.74
Sample_T3C_bin.4	99.41	0.00	60	41.7	Lachnospiraceae	41.7	2.51
Sample_T3C_bin.7	87.74	0.58	79	59.6	Bacteroidetes	59.6	2.60
Sample_T3C_bin.6	99.28	0.00	60	45.4	Bacteroidales	45.4	4.07
Sample_T3C_bin.1	98.18	1.99	353	56.6	Bifidobacteriaceae	56.6	2.24
Sample_T3C_bin.3	97.94	0.00	112	43.3	Clostridiales	43.3	2.50
Sample_C2A_bin.56	98.65	0.67	23	37.4	Clostridiales	37.4	2.10
Sample_C6A_bin.16	93.60	1.34	36	49.5	Clostridiales	49.5	2.01
Sample_C14C_bin.25	91.72	0.22	342	50.9	Clostridiales	50.9	2.37
Sample_C7C_bin.21	97.31	0.00	30	43.5	Clostridiales	43.5	2.02
Sample_C7C_bin.23	98.37	1.63	215	42.3	Bacteroidales	42.3	5.04
Sample_C6A_bin.15	97.76	0.34	101	36.6	Clostridiales	36.6	2.01
Sample_C6A_bin.14	92.99	4.58	147	42.6	Lachnospiraceae	42.6	3.36
Sample_T2C_bin.32	98.75	0.47	81	44.3	Bacteroidales	44.3	3.60
Sample_T2C_bin.30	89.64	0.84	79	49.7	Clostridiales	49.7	2.09
Sample_T2C_bin.36	87.52	0.93	450	58.1	Clostridiales	58.1	2.75
Sample_T2C_bin.37	98.30	0.38	139	43.4	Bacteroidales	43.4	3.04
Sample_T4C_bin.18	84.24	0.00	88	41.7	Bacteroides	41.7	5.36
Sample_T4C_bin.19	92.61	0.67	31	60	Clostridiales	60	1.99
Sample_T4C_bin.16	90.24	0.58	258	41	Lachnospiraceae	41	2.44
Sample_T2C_bin.38	85.02	0.50	336	46.2	Bacteroidetes	46.2	2.04
Sample_C7B_bin.18	98.73	0.02	116	48.5	Firmicutes	48.5	2.18
Sample_T4C_bin.12	94.62	1.70	43	59.1	Clostridiales	59.1	2.16
Sample_T4C_bin.13	98.63	0.00	29	43.1	Clostridiales	43.1	2.70
Sample_T4C_bin.10	98.65	0.00	238	57.2	Clostridiales	57.2	2.96
Sample_T4C_bin.11	92.59	2.54	51	62.7	Clostridiales	62.7	1.99
Sample_C4C_bin.39	97.98	0.00	85	46	Clostridiales	46	2.43
Sample_C4C_bin.38	99.32	0.00	30	44.1	Clostridiales	44.1	2.82
Sample_C12C_bin.9	95.24	0.67	192	38.1	Clostridiales	38.1	1.77
Sample_C12C_bin.8	86.24	1.95	48	56.8	Clostridiales	56.8	2.18

Sample_C4C_bin.31	98.73	0.02	58	48.3	Firmicutes	48.3	2.16
Sample_C12C_bin.4	93.25	1.28	34	27.7	Bacteria	27.7	1.25
Sample_C12C_bin.7	98.99	0.00	48	44.9	Clostridiales	44.9	2.59
Sample_C12C_bin.6	90.26	0.67	38	60.3	Clostridiales	60.3	1.93
Sample_C4C_bin.35	98.65	0.00	190	36.3	Clostridiales	36.3	4.17
Sample_C4C_bin.34	97.26	0.00	186	43.1	Clostridiales	43.1	2.54
Sample_C12C_bin.3	95.61	1.60	305	45.4	Bacteroidales	45.4	4.28
Sample_C12C_bin.2	92.45	0.00	85	36.3	Clostridiales	36.3	2.26
Sample_C2A_bin.52	90.24	2.46	89	61.1	Clostridiales	61.1	2.06
Sample_T5A_bin.24	98.79	0.48	38	58.5	Bacteroidetes	58.5	3.14
Sample_T5A_bin.25	92.47	2.35	72	42.9	Bacteroides	42.9	5.49
Sample_T5A_bin.26	97.29	0.09	83	44.4	Bacteroidales	44.4	3.12
Sample_T5A_bin.27	100.00	0.11	113	48.5	Proteobacteria	48.5	2.82
Sample_T5A_bin.20	91.93	0.74	111	46.6	Bacteroidales	46.6	4.07
Sample_T5A_bin.21	99.13	0.00	41	48.6	Bacteroidales	48.6	3.54
Sample_T5A_bin.22	95.35	1.27	105	41.5	Clostridiales	41.5	3.21
Sample_T5A_bin.23	94.83	0.76	89	44.8	Clostridiales	44.8	2.93
Sample_C8A_bin.24	93.70	0.48	275	59.6	Bacteroidetes	59.6	2.66
Sample_C8A_bin.25	96.48	0.74	124	48.6	Bacteroidales	48.6	3.49
Sample_C8A_bin.26	94.02	0.63	123	40.6	Bacteroidales	40.6	3.09
Sample_T5A_bin.28	99.03	1.28	53	44.9	Bacteroidales	44.9	4.72
Sample_C8A_bin.21	98.65	0.34	58	38.7	Clostridiales	38.7	2.30
Sample_C8A_bin.22	98.71	0.13	115	45.3	Bacteroidales	45.3	4.14
Sample_T4A_bin.40	98.42	0.78	86	34.6	Lactobacillus	34.6	1.90
Sample_T14C_bin.26	98.63	0.00	33	42.9	Clostridiales	42.9	2.76
Sample_C7B_bin.31	98.98	2.43	45	44.9	Prevotella	44.9	3.53
Sample_C7B_bin.30	94.68	1.88	91	54.5	Proteobacteria	54.5	2.08
Sample_C7B_bin.33	99.51	1.93	173	41.1	Lachnospiraceae	41.1	3.09
Sample_C7B_bin.32	94.29	1.01	72	61.5	Clostridiales	61.5	1.93
Sample_C5C_bin.4	80.06	1.96	313	50	Clostridiales	50	1.98
Sample_C5C_bin.7	97.31	0.00	6	52.8	Clostridiales	52.8	2.02
Sample_C12C_bin.64	98.30	4.40	42	43.5	Lachnospiraceae	43.5	2.93
Sample_C5C_bin.9	96.86	0.00	78	36.5	Clostridiales	36.5	3.14
Sample_C12C_bin.60	92.00	1.33	43	25.9	Bacteria	25.9	1.35
Sample_T14C_bin.21	89.19	0.81	38	46.8	Bacteroidales	46.8	3.38
Sample_T16C_bin.2	97.78	0.63	15	44.1	Clostridiales	44.1	3.33
Sample_T16C_bin.3	98.55	3.14	51	46.7	Lachnospiraceae	46.7	3.32
Sample_T16C_bin.4	94.09	2.22	70	41	Clostridiales	41	3.48
Sample_T6B_bin.16	99.25	1.16	72	45.5	Bacteroidales	45.5	3.48

Sample_T16C_bin.6	100.00	0.46	34	59.9	Bifidobacteriaceae	59.9	2.43
Sample_T6B_bin.14	99.27	0.48	43	41.8	Lachnospiraceae	41.8	2.66
Sample_T6B_bin.19	98.65	1.01	62	40.5	Clostridiales	40.5	2.19
Sample_C9A_bin.39	94.40	3.57	94	58.4	Clostridiales	58.4	2.23
Sample_C10A_bin.64	97.04	1.34	22	52.9	Clostridiales	52.9	2.36
Sample_C10A_bin.65	93.55	1.26	82	41.2	Lachnospiraceae	41.2	2.91
Sample_C10A_bin.66	98.73	0.00	165	51.7	Firmicutes	51.7	2.44
Sample_C10A_bin.60	90.61	0.90	41	50.3	Bacteroidales	50.3	2.35
Sample_C10A_bin.62	93.82	3.93	32	27.9	Bacteria	27.9	1.23
Sample_C10A_bin.63	91.57	2.25	26	26.3	Bacteria	26.3	1.14
Sample_C9A_bin.32	87.93	2.24	58	57.6	Clostridiales	57.6	2.15
Sample_C10A_bin.68	97.67	1.90	38	48	Clostridiales	48	2.99
Sample_C10A_bin.69	99.51	0.98	29	40.8	Lachnospiraceae	40.8	2.65
Sample_C2C_bin.43	97.98	0.34	405	44.6	Clostridiales	44.6	1.91
Sample_T11A_bin.28	97.17	0.37	38	48.4	Bacteroidales	48.4	3.79
Sample_C9C_bin.9	86.61	1.39	184	41.5	Clostridiales	41.5	3.23
Sample_T11A_bin.22	99.01	0.00	50	43.2	Bacteria	43.2	4.08
Sample_C9C_bin.5	82.73	1.88	59	41.8	Bacteroidales	41.8	4.13
Sample_T11A_bin.20	98.65	1.34	106	33.8	Clostridiales	33.8	2.27
Sample_C9C_bin.7	86.70	0.67	33	61.7	Clostridiales	61.7	2.18
Sample_T11A_bin.27	97.73	0.00	188	43.5	Bacteroidales	43.5	2.91
Sample_T11A_bin.24	99.51	0.96	93	54.4	Bacteroidetes	54.4	2.36
Sample_C9C_bin.3	96.63	1.44	38	54.3	Bacteroidetes	54.3	2.28
Sample_T8C_bin.30	91.54	0.36	44	63.8	Clostridiales	63.8	2.79
Sample_T11C_bin.10	97.26	0.00	163	42.8	Clostridiales	42.8	2.74
Sample_T8C_bin.32	98.27	3.74	52	46.7	Lachnospiraceae	46.7	7.27
Sample_T11C_bin.12	94.40	0.00	20	43.1	Lachnospiraceae	43.1	2.15
Sample_T8C_bin.34	96.60	0.34	719	51.4	Clostridiales	51.4	2.31
Sample_T11C_bin.14	99.32	1.01	29	40.6	Clostridiales	40.6	2.14
Sample_T8C_bin.36	99.51	2.69	37	54.1	Bacteroidetes	54.1	2.38
Sample_T8C_bin.37	92.44	1.01	46	61.8	Clostridiales	61.8	1.92
Sample_T11C_bin.19	86.37	1.30	225	47.2	Bacteroidales	47.2	3.50
Sample_T8C_bin.39	81.60	0.79	350	40.3	Clostridiales	40.3	2.29
Sample_C2A_bin.13	95.14	2.85	39	48.2	Clostridiales	48.2	2.95
Sample_C13B_bin.24	81.21	2.82	139	40	Streptococcus	40	1.60
Sample_C13B_bin.25	99.03	0.97	34	41.5	Lachnospiraceae	41.5	2.69
Sample_C13B_bin.22	94.83	2.18	380	42.5	Bacteroides	42.5	5.85
Sample_C13B_bin.20	100.00	0.63	33	31.3	Firmicutes	31.3	2.47
Sample_C13B_bin.21	89.93	1.68	127	59.5	Clostridiales	59.5	2.18

Sample_T15C_bin.20	95.23	0.04	99	58.7	Clostridiales	58.7	3.20
Sample_T15C_bin.21	89.00	0.02	81	61.3	Clostridiales	61.3	2.12
Sample_T15C_bin.22	100.00	0.06	22	44.5	Clostridiales	44.5	2.90
Sample_T12A_bin.48	85.31	1.78	329	64.5	Deltaproteobacteria	64.5	2.00
Sample_T15C_bin.24	92.61	0.00	274	36.5	Clostridiales	36.5	1.84
Sample_T15C_bin.26	98.10	0.63	28	45.3	Firmicutes	45.3	1.76
Sample_T15C_bin.29	99.32	0.00	42	40.3	Clostridiales	40.3	2.33
Sample_T12A_bin.41	97.11	4.52	40	54.9	Bacteroidetes	54.9	2.22
Sample_T12A_bin.45	96.42	1.34	72	43.8	Clostridiales	43.8	2.83
Sample_T12A_bin.44	84.99	0.74	212	47.8	Bacteroidales	47.8	3.35
Sample_T5B_bin.36	95.32	0.19	64	43.3	Lachnospiraceae	43.3	2.58
Sample_T5B_bin.37	93.82	1.74	172	41	Clostridiales	41	3.18
Sample_T5B_bin.34	80.11	2.27	30	58.3	Clostridiales	58.3	1.59
Sample_C2A_bin.1	98.65	0.67	216	44.2	Clostridiales	44.2	2.77
Sample_T5B_bin.32	97.29	0.00	82	37.5	Clostridiales	37.5	2.78
Sample_T5B_bin.33	98.04	0.00	193	45.4	Bacteroidales	45.4	3.99
Sample_T5B_bin.30	98.79	0.48	165	58.5	Bacteroidetes	58.5	3.15
Sample_T5B_bin.31	98.65	2.80	37	37.6	Clostridiales	37.6	3.28
Sample_T5B_bin.38	95.78	2.85	32	48	Clostridiales	48	2.98
Sample_C5A_bin.61	100.00	0.23	57	56.4	Bifidobacteriaceae	56.4	2.23
Sample_C5A_bin.62	96.81	4.56	84	38.3	Clostridiales	38.3	2.73
Sample_C5A_bin.58	92.69	0.56	103	24.9	Bacteria	24.9	1.44
Sample_C5A_bin.59	98.60	0.93	103	58.1	Clostridiales	58.1	2.96
Sample_C5A_bin.57	95.57	3.86	52	41.7	Lachnospiraceae	41.7	2.81
Sample_C5A_bin.53	85.73	1.28	41	48.8	Bacteroidales	48.8	3.08
Sample_T2A_bin.19	88.03	0.85	40	32.7	Bacteria	32.7	1.95
Sample_C4C_bin.46	89.14	1.01	411	38	Clostridiales	38	2.41
Sample_C4C_bin.47	92.43	1.86	243	47	Bacteroidales	47	3.90
Sample_C5C_bin.13	95.63	3.37	14	44.1	Selenomonadales	44.1	2.20
Sample_C4C_bin.40	92.78	0.96	20	54.6	Bacteroidetes	54.6	1.96
Sample_C1C_bin.23	97.71	0.00	40	43.1	Clostridiales	43.1	2.56
Sample_C1C_bin.22	96.42	0.67	92	54.8	Clostridiales	54.8	2.58
Sample_C1C_bin.21	96.93	0.19	144	48.8	Lachnospiraceae	48.8	2.73
Sample_C1C_bin.20	97.10	0.92	47	42.9	Lachnospiraceae	42.9	3.34
Sample_C1C_bin.27	98.51	4.66	45	46.6	Bacteroidales	46.6	4.29
Sample_C1C_bin.26	98.63	4.70	58	37.4	Clostridiales	37.4	2.82
Sample_C1C_bin.29	83.91	3.57	58	59.2	Clostridiales	59.2	2.04
Sample_T13B_bin.34	97.98	1.06	305	38.3	Clostridiales	38.3	2.85
Sample_C1A_bin.36	96.57	0.96	74	55.1	Bacteroidetes	55.1	1.96

Sample_C2A_bin.39	98.10	0.87	116	48.6	Firmicutes	48.6	2.10
Sample_C2A_bin.38	84.80	1.74	394	46.8	Prevotella	46.8	2.82
Sample_T18B_bin.19	97.98	4.70	23	35.9	Clostridiales	35.9	2.69
Sample_T6A_bin.12	98.06	1.55	11	41.2	Lachnospiraceae	41.2	2.50
Sample_T6A_bin.13	97.31	0.00	225	52.8	Clostridiales	52.8	1.98
Sample_T6A_bin.11	97.98	0.69	119	51.9	Clostridiales	51.9	2.53
Sample_C2A_bin.32	97.90	0.56	156	41.6	Lachnospiraceae	41.6	2.78
Sample_T18B_bin.16	96.85	0.24	49	41.7	Lachnospiraceae	41.7	2.59
Sample_C2A_bin.37	96.13	1.33	76	42.8	Lachnospiraceae	42.8	3.27
Sample_T18B_bin.14	92.19	2.33	69	47.5	Lachnospiraceae	47.5	2.79
Sample_C8B_bin.38	98.46	0.40	127	45.4	Bacteroidales	45.4	4.05
Sample_C14C_bin.31	98.18	0.32	78	60.1	Bacteroidetes	60.1	2.67
Sample_C8B_bin.37	80.38	3.53	183	59.6	Clostridiales	59.6	2.03
Sample_C14C_bin.33	91.43	0.00	45	42	Bacteroidales	42	4.31
Sample_C8B_bin.35	98.32	3.60	127	61.4	Clostridiales	61.4	2.33
Sample_C14C_bin.35	97.98	0.00	124	47	Clostridiales	47	3.01
Sample_C14C_bin.34	98.73	1.21	131	43.5	Lachnospiraceae	43.5	2.84
Sample_C8B_bin.30	99.84	0.23	71	56.6	Bifidobacteriaceae	56.6	2.28
Sample_C8B_bin.31	99.96	0.33	111	50.8	Enterobacteriaceae	50.8	5.01
Sample_C11C_bin.10	98.65	0.67	123	44	Clostridiales	44	2.84
Sample_T13B_bin.47	95.92	0.76	50	46.4	Bacteroidales	46.4	4.30
Sample_T6C_bin.14	98.31	0.71	43	47.8	Clostridiales	47.8	3.06
Sample_T13B_bin.46	89.81	0.37	66	44.3	Bacteroidales	44.3	3.27
Sample_T13B_bin.45	89.55	0.00	109	40.1	Clostridiales	40.1	2.22
Sample_T13B_bin.42	90.10	2.01	39	58.7	Clostridiales	58.7	2.08
Sample_T17C_bin.17	100.00	1.10	21	42.2	Selenomonadales	42.2	1.89
Sample_T17C_bin.16	99.55	0.00	26	60.4	Bifidobacteriaceae	60.4	1.91
Sample_T17C_bin.15	98.65	0.02	18	36	Clostridiales	36	2.72
Sample_T17C_bin.14	89.87	0.63	125	46.8	Clostridiales	46.8	2.94
Sample_T17C_bin.13	98.73	1.44	46	41	Clostridiales	41	3.57
Sample_T17C_bin.12	98.86	0.73	113	40.5	Bacteroidales	40.5	3.74
Sample_T17C_bin.11	98.65	0.67	67	33.2	Clostridiales	33.2	2.20
Sample_T17C_bin.10	98.94	0.00	15	38	Lactobacillus	38	1.99
Sample_T2C_bin.34	98.51	3.07	17	44.9	Bacteroidales	44.9	3.76
Sample_T4C_bin.14	99.09	0.68	71	56.7	Bifidobacteriaceae	56.7	2.20
Sample_C4C_bin.28	97.98	3.36	36	58	Clostridiales	58	2.03
Sample_C4C_bin.29	98.65	0.00	35	36.3	Clostridiales	36.3	2.57
Sample_C4C_bin.22	90.22	0.72	87	59.5	Bacteroidetes	59.5	2.50
Sample_C4C_bin.23	98.86	0.38	214	43.4	Bacteroidales	43.4	3.03

Sample_C4C_bin.20	93.60	0.44	765	49.8	Lachnospiraceae	49.8	2.80
Sample_C4C_bin.21	80.09	1.28	281	45.4	Bacteroidales	45.4	3.43
Sample_C4C_bin.27	97.31	0.00	67	49.6	Clostridiales	49.6	2.25
Sample_C4C_bin.24	98.65	3.02	68	37.3	Clostridiales	37.3	2.43
Sample_C4C_bin.25	97.31	0.00	16	44.1	Clostridiales	44.1	2.72
Sample_C7A_bin.18	87.25	2.05	234	55.3	Bacteroidetes	55.3	1.93
Sample_C7A_bin.19	89.52	2.07	237	41.9	Lachnospiraceae	41.9	2.85
Sample_C7A_bin.12	97.92	0.13	58	49.9	Bacteroidales	49.9	2.51
Sample_C7A_bin.13	96.13	2.53	84	45.6	Lachnospiraceae	45.6	3.41
Sample_C7A_bin.10	85.69	1.27	335	41.9	Clostridiales	41.9	2.75
Sample_C7A_bin.11	95.85	0.38	184	41.7	Bacteroidales	41.7	4.34
Sample_C7A_bin.16	86.97	2.35	347	38.2	Clostridiales	38.2	2.44
Sample_C7A_bin.17	95.59	0.79	94	59	Clostridiales	59	2.51
Sample_C7A_bin.14	89.93	2.07	26	56.9	Clostridiales	56.9	2.03
Sample_T1B_bin.2	98.24	1.75	113	42.5	Bacteria	42.5	4.13
Sample_T1B_bin.3	99.05	0.47	116	33.5	Bacteria	33.5	2.30
Sample_T1B_bin.1	99.27	0.00	109	41.6	Lachnospiraceae	41.6	2.83
Sample_T1B_bin.7	99.32	0.00	17	36	Clostridiales	36	2.66
Sample_T1B_bin.4	81.31	0.68	95	57.8	Clostridiales	57.8	1.94
Sample_T1B_bin.5	100.00	1.27	125	31.2	Firmicutes	31.2	2.50
Sample_T4C_bin.23	98.15	0.06	183	59.8	Bifidobacteriaceae	59.8	2.63
Sample_T4C_bin.21	93.08	0.00	95	43.3	Bacteria	43.3	3.74
Sample_T4C_bin.20	97.27	0.68	257	56.3	Clostridiales	56.3	2.32
Sample_T4C_bin.27	99.32	1.51	24	40.5	Clostridiales	40.5	2.34
Sample_T4C_bin.26	94.05	2.38	80	58.7	Clostridiales	58.7	2.44
Sample_T4C_bin.29	98.65	0.06	133	46.2	Clostridiales	46.2	2.94
Sample_T4C_bin.28	89.86	1.90	589	50.1	Clostridiales	50.1	5.38
Sample_C4C_bin.30	89.93	2.68	108	61.7	Clostridiales	61.7	1.93
Sample_C4C_bin.33	92.58	0.00	49	60.3	Clostridiales	60.3	2.43
Sample_C7B_bin.22	93.28	1.34	49	58.6	Clostridiales	58.6	2.04
Sample_C7B_bin.23	98.65	0.17	24	44	Clostridiales	44	2.88
Sample_T12B_bin.17	99.32	0.34	30	44.1	Clostridiales	44.1	2.82
Sample_C7B_bin.21	96.84	1.15	205	60.1	Bacteroidetes	60.1	2.64
Sample_C7B_bin.27	94.33	2.40	150	43.2	Clostridiales	43.2	2.50
Sample_C7B_bin.24	91.27	4.43	306	58.2	Clostridiales	58.2	2.01
Sample_C7B_bin.25	95.56	0.65	125	49	Clostridiales	49	2.54
Sample_T16A_bin.2	93.45	1.56	314	45	Lachnospiraceae	45	2.54
Sample_C7B_bin.29	98.70	0.28	109	45.7	Bacteroidales	45.7	3.48
Sample_T12B_bin.19	97.98	0.34	65	46	Clostridiales	46	2.40

Sample_T16A_bin.5	85.05	2.44	219	60.5	Clostridiales	60.5	1.67
Sample_T17C_bin.4	97.66	0.00	59	43.1	Lachnospiraceae	43.1	2.82
Sample_C4C_bin.37	92.04	3.10	269	37.7	Clostridiales	37.7	2.38
Sample_T17C_bin.2	96.72	0.33	94	51.1	Enterobacteriaceae	51.1	4.21
Sample_C9A_bin.29	80.95	2.01	383	37.3	Clostridiales	37.3	2.13
Sample_T14B_bin.33	97.98	0.34	15	44.1	Clostridiales	44.1	2.01
Sample_C9A_bin.24	82.61	0.00	37	37.1	Bacteria	37.1	2.63
Sample_C9A_bin.27	97.98	1.34	156	33.2	Clostridiales	33.2	2.14
Sample_C9A_bin.26	99.70	0.00	279	59.8	Deltaproteobacteria	59.8	4.22
Sample_C9A_bin.21	99.98	1.50	25	43.7	Selenomonadales	43.7	2.40
Sample_C9A_bin.20	94.06	0.19	76	42.2	Bacteroidales	42.2	3.36
Sample_C9A_bin.23	97.98	0.00	65	42.3	Clostridiales	42.3	2.56
Sample_T18C_bin.21	91.32	1.25	24	60.4	Clostridiales	60.4	2.42
Sample_T18C_bin.23	99.98	1.50	39	43.6	Selenomonadales	43.6	2.42
Sample_T18C_bin.25	91.57	0.78	136	41.1	Lachnospiraceae	41.1	2.24
Sample_T18C_bin.24	98.14	1.12	43	46.2	Bacteroidales	46.2	3.41
Sample_T18C_bin.27	97.31	0.00	11	53	Clostridiales	53	1.95
Sample_T12A_bin.21	83.91	0.22	212	60.3	Clostridiales	60.3	1.92
Sample_C12A_bin.29	95.30	0.00	23	42.4	Clostridiales	42.4	1.93
Sample_T11A_bin.39	98.11	1.93	52	41.6	Lachnospiraceae	41.6	2.69
Sample_T11A_bin.38	99.42	0.13	63	45	Bacteroidales	45	4.83
Sample_T11A_bin.31	97.98	1.51	120	38.4	Clostridiales	38.4	2.82
Sample_T11A_bin.30	94.30	0.00	54	49.2	Clostridiales	49.2	2.50
Sample_T11A_bin.33	93.31	0.48	66	41.8	Lachnospiraceae	41.8	2.89
Sample_T11A_bin.32	93.09	0.23	108	56.7	Bifidobacteriaceae	56.7	1.94
Sample_T11A_bin.35	100.00	0.81	29	60	Actinobacteria	60	2.14
Sample_T11A_bin.34	94.55	0.00	106	60.6	Clostridiales	60.6	2.40
Sample_T11A_bin.37	93.29	2.01	71	57.9	Clostridiales	57.9	2.02
Sample_T8A_bin.7	96.92	1.25	205	47.2	Selenomonadales	47.2	2.17
Sample_T13C_bin.3	99.32	1.01	88	40.4	Clostridiales	40.4	2.32
Sample_T11C_bin.25	98.47	0.24	46	49.5	Lachnospiraceae	49.5	3.02
Sample_T13C_bin.1	93.95	0.34	10	44.8	Clostridiales	44.8	1.76
Sample_T11C_bin.27	98.65	0.00	61	58.8	Clostridiales	58.8	2.03
Sample_T11C_bin.20	92.69	4.49	20	26	Bacteria	26	1.29
Sample_C12B_bin.29	85.01	2.68	136	56.8	Clostridiales	56.8	1.95
Sample_T11C_bin.22	87.68	0.63	335	42.2	Clostridiales	42.2	2.19
Sample_T14A_bin.46	98.63	0.00	66	42.4	Clostridiales	42.4	3.03
Sample_T14A_bin.48	93.91	1.44	55	54.7	Bacteroidetes	54.7	2.03
Sample_T13C_bin.9	83.27	1.76	74	59.8	Clostridiales	59.8	1.91

Sample_T13C_bin.8	99.36	0.63	125	49.2	Proteobacteria	49.2	2.31
Sample_T11C_bin.28	99.24	0.38	91	43.4	Bacteroidales	43.4	2.98
Sample_T11C_bin.29	89.03	0.13	95	62.7	Clostridiales	62.7	2.77
Sample_T4B_bin.4	99.51	0.96	67	54.3	Bacteroidetes	54.3	2.34
Sample_T11A_bin.9	98.35	2.27	108	44.1	Clostridiales	44.1	3.35
Sample_T11A_bin.8	97.31	0.00	32	34.3	Clostridiales	34.3	2.23
Sample_T4B_bin.1	98.65	0.34	78	44	Clostridiales	44	2.78
Sample_T4B_bin.2	88.59	0.00	99	42.6	Bacteroidales	42.6	3.25
Sample_C13B_bin.34	98.65	0.34	264	38.4	Clostridiales	38.4	2.72
Sample_C13B_bin.31	99.25	0.12	97	49.4	Bacteroidales	49.4	2.76
Sample_T8A_bin.8	92.51	2.95	298	45.8	Clostridiales	45.8	2.79
Sample_T11A_bin.5	94.96	1.34	333	55.2	Clostridiales	55.2	2.27
Sample_C13B_bin.32	85.99	1.27	84	42.6	Clostridiales	42.6	2.02
Sample_T14B_bin.26	98.10	1.50	72	48	Clostridiales	48	3.02
Sample_T14B_bin.27	81.59	2.04	411	56.4	Clostridiales	56.4	2.13
Sample_T14B_bin.24	98.63	0.00	37	42.9	Clostridiales	42.9	2.81
Sample_T14B_bin.25	93.28	1.59	40	59.8	Clostridiales	59.8	2.13
Sample_T14B_bin.22	99.30	1.17	87	58	Clostridiales	58	3.03
Sample_T15C_bin.32	90.60	2.68	55	56.7	Clostridiales	56.7	2.47
Sample_T12A_bin.38	84.83	0.56	65	26.7	Bacteria	26.7	1.04
Sample_T12A_bin.39	96.97	0.73	139	36.6	Clostridiales	36.6	1.70
Sample_T12A_bin.36	99.18	0.00	32	46.7	Lactobacillales	46.7	2.05
Sample_T12A_bin.35	90.90	2.55	298	28.8	Clostridiales	28.8	2.26
Sample_T12A_bin.32	91.94	0.34	44	44.3	Clostridiales	44.3	1.82
Sample_T12A_bin.33	93.95	2.01	422	60.3	Clostridiales	60.3	1.98
Sample_T12A_bin.30	93.58	0.00	25	43.5	Clostridiales	43.5	1.98
Sample_T15C_bin.38	98.65	0.34	107	38.8	Clostridiales	38.8	2.64
Sample_C6A_bin.9	95.43	1.03	93	44.5	Bacteroidales	44.5	3.12
Sample_C6A_bin.8	98.10	0.02	115	48.6	Firmicutes	48.6	2.12
Sample_T10B_bin.21	89.19	0.67	35	38.6	Clostridiales	38.6	2.43
Sample_C11B_bin.32	94.60	0.69	70	45.2	Clostridiales	45.2	2.32
Sample_C11B_bin.35	87.42	0.19	60	41.1	Lachnospiraceae	41.1	2.23
Sample_C11B_bin.34	100.00	0.48	50	54.8	Bacteroidetes	54.8	2.77
Sample_C11B_bin.39	97.30	0.84	91	34.2	Bacteria	34.2	2.10
Sample_C13A_bin.27	84.50	0.67	295	51.6	Clostridiales	51.6	1.88
Sample_C8A_bin.28	85.36	4.67	489	47.6	Lachnospiraceae	47.6	2.47
Sample_C13A_bin.25	93.44	1.24	133	48.9	Bacteroidales	48.9	3.36
Sample_C8A_bin.29	99.60	0.55	134	50.9	Enterobacteriaceae	50.9	4.32
Sample_C6C_bin.38	97.98	0.00	31	37.4	Clostridiales	37.4	2.72

Sample_C6C_bin.33	81.26	1.89	194	33.5	Bacteria	33.5	1.79
Sample_C6C_bin.32	96.72	1.34	253	40.7	Bacteroidales	40.7	3.11
Sample_C14B_bin.23	95.12	0.48	100	41.8	Lachnospiraceae	41.8	3.26
Sample_C14B_bin.22	99.44	0.24	93	49.5	Lachnospiraceae	49.5	2.99
Sample_C6C_bin.36	99.32	0.00	23	41.3	Clostridiales	41.3	2.64
Sample_C6C_bin.35	97.46	1.27	76	41.9	Clostridiales	41.9	2.83
Sample_C13A_bin.22	92.87	0.53	213	47.1	Clostridiales	47.1	2.82
Sample_C11A_bin.2	98.55	1.93	212	41.8	Lachnospiraceae	41.8	2.89
Sample_C11A_bin.3	97.27	1.19	53	47.4	Bacteroidales	47.4	2.76
Sample_C11A_bin.1	97.76	1.45	634	36	Clostridiales	36	2.39
Sample_C11A_bin.6	97.98	2.50	254	38.1	Clostridiales	38.1	3.11
Sample_C11A_bin.5	85.42	0.42	408	47.5	Bacteroidales	47.5	3.47
Sample_C11A_bin.8	100.00	0.76	91	44.4	Clostridiales	44.4	2.80
Sample_C1C_bin.34	98.30	0.00	58	41.9	Clostridiales	41.9	2.76
Sample_C1C_bin.35	99.51	2.72	33	54.3	Bacteroidetes	54.3	2.22
Sample_C1C_bin.36	99.44	0.97	55	49.4	Lachnospiraceae	49.4	3.11
Sample_C1C_bin.37	93.58	3.61	219	43.1	Lachnospiraceae	43.1	2.17
Sample_C1C_bin.30	90.14	1.42	144	60.3	Clostridiales	60.3	2.06
Sample_C1C_bin.31	98.77	0.38	74	42.1	Bacteroidales	42.1	4.55
Sample_C1C_bin.32	88.53	0.00	54	40.2	Bacteroidales	40.2	2.91
Sample_C1C_bin.33	90.15	0.97	48	41.5	Lachnospiraceae	41.5	2.78
Sample_C1C_bin.38	97.84	0.00	44	51	Bacteria	51	1.92
Sample_C3A_bin.17	88.70	3.08	287	57	Clostridiales	57	2.14
Sample_T6A_bin.29	97.57	1.09	417	51	Enterobacteriaceae	51	4.57
Sample_C2A_bin.29	96.83	1.27	85	44.5	Clostridiales	44.5	2.73
Sample_T16B_bin.15	94.29	1.51	211	38.2	Clostridiales	38.2	2.37
Sample_T16B_bin.14	96.64	1.47	169	36.8	Clostridiales	36.8	3.20
Sample_T16B_bin.17	100.00	0.00	81	44.5	Clostridiales	44.5	2.87
Sample_T16B_bin.16	98.65	1.34	38	36.3	Clostridiales	36.3	2.51
Sample_T16B_bin.18	85.14	2.14	215	45.5	Lachnospiraceae	45.5	2.22
Sample_T6A_bin.21	97.43	0.00	225	48.4	Bacteroidales	48.4	3.78
Sample_T6A_bin.20	90.38	0.69	161	62.1	Clostridiales	62.1	1.86
Sample_T6A_bin.26	99.30	1.17	96	57.6	Clostridiales	57.6	3.28
Sample_C2A_bin.24	98.65	1.01	38	40.3	Clostridiales	40.3	2.19
Sample_T6A_bin.24	98.65	0.00	307	36.1	Clostridiales	36.1	2.67
Sample_C4B_bin.10	90.40	2.00	92	48.6	Proteobacteria	48.6	2.39
Sample_C4A_bin.36	98.65	0.00	52	40.5	Clostridiales	40.5	2.75
Sample_C4A_bin.35	81.43	0.78	449	48.8	Proteobacteria	48.8	2.11
Sample_C4A_bin.34	97.95	0.00	91	58.2	Bacteria	58.2	3.00

Sample_C4A_bin.33	98.83	1.17	43	58.1	Clostridiales	58.1	3.01
Sample_C4A_bin.32	98.63	0.00	79	37.3	Clostridiales	37.3	2.68
Sample_C4A_bin.31	92.93	1.45	26	44.3	Clostridiales	44.3	2.91
Sample_C4A_bin.30	95.97	0.89	235	36.2	Clostridiales	36.2	2.36
Sample_T3B_bin.4	99.19	0.81	63	59.8	Actinobacteria	59.8	2.42
Sample_T3B_bin.5	97.47	0.85	98	57.5	Clostridiales	57.5	2.36
Sample_T3B_bin.6	90.44	1.08	74	60.3	Clostridiales	60.3	1.87
Sample_T3B_bin.7	93.28	0.89	76	61.6	Clostridiales	61.6	1.98
Sample_C4A_bin.39	91.19	4.25	72	61.6	Clostridiales	61.6	2.03
Sample_C4A_bin.38	98.11	0.00	26	43.3	Bacteroidales	43.3	3.04
Sample_C4B_bin.29	86.20	2.04	122	59	Clostridiales	59	1.99
Sample_T11B_bin.34	94.64	2.02	114	56.6	Bifidobacteriaceae	56.6	2.05
Sample_T11B_bin.35	94.38	0.22	91	42	Bacteroidales	42	4.19
Sample_C4B_bin.21	84.11	0.67	210	59.3	Clostridiales	59.3	1.68
Sample_C4B_bin.20	97.83	0.53	90	49.4	Lachnospiraceae	49.4	3.10
Sample_C4B_bin.22	90.61	1.36	100	60.8	Clostridiales	60.8	2.21
Sample_C4B_bin.25	99.32	0.67	31	43.9	Clostridiales	43.9	2.92
Sample_C4B_bin.24	97.92	2.85	19	44.7	Clostridiales	44.7	3.09
Sample_C4B_bin.27	90.19	1.95	201	47.3	Bacteroidales	47.3	3.77
Sample_T11B_bin.37	92.00	0.60	338	48.8	Bacteroidales	48.8	3.20
Sample_C8A_bin.4	97.91	2.61	59	43.4	Lachnospiraceae	43.4	2.99
Sample_T11B_bin.30	91.79	0.37	96	40.4	Bacteroidales	40.4	3.03
Sample_C3A_bin.19	81.54	2.47	375	43.6	Clostridiales	43.6	2.28
Sample_C1A_bin.4	97.98	0.67	266	44.5	Clostridiales	44.5	1.91
Sample_C3A_bin.29	86.78	3.79	324	58.6	Clostridia	58.6	2.38
Sample_T2C_bin.18	99.35	0.24	66	41.8	Lachnospiraceae	41.8	2.59
Sample_T2C_bin.19	99.25	0.19	71	48.2	Bacteroidales	48.2	4.40
Sample_T2C_bin.10	89.73	3.99	288	59.6	Clostridiales	59.6	1.84
Sample_C8B_bin.43	94.84	0.54	48	41.5	Lachnospiraceae	41.5	2.93
Sample_T13B_bin.41	98.38	0.19	233	45.8	Actinobacteria	45.8	2.11
Sample_T2C_bin.14	98.90	0.00	47	36.7	Clostridiales	36.7	3.05
Sample_T2C_bin.15	95.48	4.11	54	44.9	Lachnospiraceae	44.9	2.70
Sample_C8B_bin.42	92.72	0.63	82	49.3	Clostridiales	49.3	2.39
Sample_C4C_bin.18	97.28	2.17	46	43.7	Lachnospiraceae	43.7	2.80
Sample_C4C_bin.17	89.04	0.11	55	26.6	Bacteria	26.6	1.07
Sample_C4C_bin.16	96.47	2.10	231	43.9	Selenomonadales	43.9	2.25
Sample_C8B_bin.40	94.21	3.82	150	59.3	Clostridiales	59.3	1.92
Sample_C4C_bin.13	96.30	0.00	62	50.3	Clostridiales	50.3	2.33
Sample_C4C_bin.12	97.32	0.68	205	56.7	Clostridiales	56.7	2.23

Sample_C4C_bin.10	99.30	1.17	86	58	Clostridiales	58	3.21
Sample_T2B_bin.53	97.27	1.36	115	56.4	Clostridiales	56.4	2.50
Sample_T2B_bin.52	91.83	1.53	239	60.5	Clostridiales	60.5	2.01
Sample_T2B_bin.51	86.66	0.98	71	42.9	Lachnospiraceae	42.9	1.84
Sample_C7A_bin.29	97.98	0.00	25	47.8	Clostridiales	47.8	2.16
Sample_C7A_bin.28	97.31	1.01	68	56.7	Clostridiales	56.7	2.79
Sample_C7A_bin.27	84.81	0.00	39	62.4	Clostridiales	62.4	1.69
Sample_C7A_bin.25	100.00	2.35	124	45	Bacteria	45	3.75
Sample_C7A_bin.24	85.29	0.22	37	52.6	Clostridiales	52.6	1.95
Sample_C7A_bin.22	95.89	0.00	278	40.9	Bacteroidales	40.9	3.06
Sample_C7A_bin.21	99.30	1.17	92	57.9	Clostridiales	57.9	3.11
Sample_C3A_bin.49	99.77	0.74	93	50.7	Enterobacteriaceae	50.7	4.75
Sample_T18A_bin.9	100.00	0.00	40	63.5	Actinobacteria	63.5	2.80
Sample_C1A_bin.20	97.27	0.68	219	56.5	Clostridiales	56.5	2.32
Sample_C3A_bin.41	88.71	0.00	23	42.7	Bacteroidales	42.7	4.84
Sample_T18A_bin.1	97.59	0.96	21	54.5	Bacteroidetes	54.5	2.27
Sample_C3A_bin.43	91.27	3.36	84	46.9	Clostridiales	46.9	2.03
Sample_C3A_bin.42	94.40	0.00	149	44.5	Clostridiales	44.5	2.11
Sample_T18A_bin.4	96.82	4.35	34	43.7	Lachnospiraceae	43.7	2.81
Sample_T13A_bin.14	81.03	3.45	99	42.1	Bacteria	42.1	3.69
Sample_C3A_bin.46	85.57	3.02	307	58.4	Clostridiales	58.4	1.78
Sample_T4C_bin.34	83.68	0.95	30	41.2	Clostridiales	41.2	2.83
Sample_T4C_bin.37	98.12	0.00	37	57.1	Proteobacteria	57.1	2.48
Sample_T4C_bin.32	93.95	1.34	468	61.9	Clostridiales	61.9	2.03
Sample_C7B_bin.36	89.93	2.89	31	57	Clostridiales	57	2.25
Sample_T4C_bin.38	98.61	0.72	46	49.4	Lachnospiraceae	49.4	2.99
Sample_T4C_bin.39	94.79	0.87	83	46.6	Bacteroidales	46.6	4.20
Sample_C5C_bin.8	92.17	0.19	192	45.7	Bacteroidales	45.7	3.86
Sample_T12B_bin.28	98.14	0.00	70	41.5	Lachnospiraceae	41.5	2.87
Sample_T12B_bin.29	98.07	0.96	37	54.5	Bacteroidetes	54.5	2.17
Sample_C7B_bin.15	98.99	0.13	190	43.4	Bacteroidales	43.4	3.05
Sample_C7B_bin.12	98.30	0.00	231	49.8	Bacteroidales	49.8	2.52
Sample_C7B_bin.11	96.83	0.63	46	31.4	Firmicutes	31.4	2.55
Sample_C7B_bin.10	80.61	2.53	161	42.4	Clostridiales	42.4	1.98
Sample_T12B_bin.21	98.73	0.02	109	48.6	Firmicutes	48.6	2.04
Sample_T12B_bin.24	97.82	0.04	192	55.6	Proteobacteria	55.6	2.70
Sample_T12B_bin.25	95.16	4.83	51	43.8	Lachnospiraceae	43.8	2.69
Sample_C7B_bin.19	86.70	2.48	277	47	Clostridiales	47	2.31
Sample_T12B_bin.27	96.83	0.63	74	31.7	Firmicutes	31.7	2.23

Sample_T1A_bin.1	83.27	3.03	120	40.8	Bacteroidales	40.8	2.71
Sample_T1A_bin.2	90.75	1.66	201	44.9	Bacteroidales	44.9	3.38
Sample_T1A_bin.4	99.36	1.69	103	43.9	Clostridiales	43.9	3.60
Sample_T1A_bin.7	99.03	0.74	114	41.6	Lachnospiraceae	41.6	2.77
Sample_T1A_bin.6	98.65	1.52	97	57.2	Enterobacteriaceae	57.2	5.50
Sample_T1A_bin.9	100.00	1.27	130	31.3	Firmicutes	31.3	2.38
Sample_C2A_bin.54	98.24	0.00	86	41.8	Lachnospiraceae	41.8	2.71
Sample_T4A_bin.24	86.93	0.19	496	41.1	Lachnospiraceae	41.1	2.31
Sample_T4A_bin.26	98.65	0.00	95	45.9	Clostridiales	45.9	2.58
Sample_T4A_bin.21	97.33	1.43	29	41.8	Bacteroides	41.8	6.10
Sample_T4A_bin.23	93.28	0.00	251	61.9	Clostridiales	61.9	2.04
Sample_T4A_bin.22	97.31	0.00	33	52.9	Clostridiales	52.9	2.09
Sample_C9A_bin.36	93.13	0.17	97	59.8	Clostridiales	59.8	2.00
Sample_C9A_bin.37	99.32	1.01	19	40.9	Clostridiales	40.9	2.11
Sample_C9A_bin.34	95.32	0.00	49	41.8	Lachnospiraceae	41.8	2.36
Sample_C9A_bin.35	84.28	1.27	259	43.4	Clostridiales	43.4	3.04
Sample_T4A_bin.29	91.94	0.00	47	60.7	Clostridiales	60.7	2.30
Sample_T4A_bin.28	97.92	0.92	56	60.1	Bifidobacteriaceae	60.1	2.17
Sample_C9A_bin.30	96.64	0.67	111	60.5	Clostridiales	60.5	3.09
Sample_C9A_bin.31	98.04	0.00	302	48.7	Proteobacteria	48.7	2.41
Sample_T18C_bin.14	99.32	1.01	25	40.5	Clostridiales	40.5	2.23
Sample_T18C_bin.15	95.88	1.58	178	44.2	Clostridiales	44.2	3.14
Sample_T18C_bin.16	83.24	0.88	227	42.5	Bacteroidales	42.5	3.15
Sample_T18C_bin.17	98.65	0.00	19	35.8	Clostridiales	35.8	2.63
Sample_T18C_bin.11	81.03	2.59	129	40.4	Bacteria	40.4	3.31
Sample_T18C_bin.12	91.04	0.16	75	46.2	Bacteroidales	46.2	2.90
Sample_T18C_bin.13	90.43	0.67	144	36.3	Clostridiales	36.3	2.24
Sample_T18C_bin.18	97.04	0.67	236	48.8	Proteobacteria	48.8	2.30
Sample_T18C_bin.19	98.55	0.72	73	41.5	Lachnospiraceae	41.5	3.19
Sample_T13B_bin.32	93.98	1.90	33	49.4	Proteobacteria	49.4	2.02
Sample_T13B_bin.33	86.54	0.00	86	41.6	Lachnospiraceae	41.6	2.21
Sample_T13B_bin.30	96.14	1.44	129	42	Bacteroidales	42	4.66
Sample_T13B_bin.31	84.76	0.45	182	56.8	Bifidobacteriaceae	56.8	1.69
Sample_C5C_bin.16	98.97	1.84	62	43	Lachnospiraceae	43	3.30
Sample_T13B_bin.37	98.94	1.79	49	47.9	Clostridiales	47.9	3.16
Sample_C5C_bin.14	98.65	1.01	57	40.4	Clostridiales	40.4	2.32
Sample_C5C_bin.15	94.21	0.59	44	59	Clostridiales	59	2.18
Sample_C5C_bin.18	99.32	2.01	28	35.9	Clostridiales	35.9	2.72
Sample_C5C_bin.19	98.65	0.67	34	33.3	Clostridiales	33.3	2.29

Sample_T11C_bin.39	99.32	2.68	58	37	Clostridiales	37	2.72
Sample_T11C_bin.38	89.91	1.37	183	60.2	Bifidobacteriaceae	60.2	1.90
Sample_T11C_bin.33	87.83	1.36	170	60	Clostridiales	60	1.94
Sample_T11C_bin.32	97.84	0.00	137	43.3	Bacteria	43.3	4.05
Sample_T11C_bin.30	85.37	2.89	65	57.7	Clostridiales	57.7	1.90
Sample_T11C_bin.37	100.00	2.50	45	56.7	Bifidobacteriaceae	56.7	2.08
Sample_T11C_bin.36	100.00	0.81	90	60.1	Actinobacteria	60.1	2.13
Sample_T14B_bin.35	90.33	0.54	188	43.2	Lachnospiraceae	43.2	2.01
Sample_T14B_bin.37	99.32	0.67	27	40.4	Clostridiales	40.4	2.25
Sample_T14B_bin.36	96.66	0.01	148	43.5	Bacteroidales	43.5	2.98
Sample_T17C_bin.3	97.45	0.87	113	38.5	Clostridiales	38.5	2.69
Sample_T14B_bin.30	95.23	1.59	64	60.7	Clostridiales	60.7	2.28
Sample_T17C_bin.1	85.36	0.00	88	44.3	Clostridiales	44.3	3.06
Sample_T12A_bin.25	83.97	1.72	545	43.6	Bacteria	43.6	2.80
Sample_T12A_bin.24	97.31	0.00	72	41	Clostridiales	41	2.61
Sample_T12A_bin.27	82.20	0.90	320	46.5	Bacteroidales	46.5	2.97
Sample_T12A_bin.26	98.55	1.62	44	46	Lachnospiraceae	46	2.96
Sample_T14B_bin.39	99.51	1.46	71	53.3	Bacteroidetes	53.3	3.07
Sample_T12A_bin.20	94.18	1.34	142	45	Clostridiales	45	2.42
Sample_T17C_bin.9	99.16	0.28	45	44.1	Bacteroidales	44.1	3.77
Sample_T12A_bin.22	95.86	1.84	21	46.3	Bacteroidetes	46.3	2.20
Sample_T12C_bin.17	97.30	0.84	79	33.3	Bacteria	33.3	2.23
Sample_T12C_bin.15	98.65	0.34	59	38.6	Clostridiales	38.6	2.19
Sample_T12C_bin.12	98.65	0.00	72	38	Clostridiales	38	2.00
Sample_T12C_bin.10	97.46	0.02	24	48.6	Firmicutes	48.6	2.03
Sample_C2A_bin.45	89.38	1.70	262	56.6	Clostridiales	56.6	2.24
Sample_T12C_bin.18	99.51	1.44	28	54.1	Bacteroidetes	54.1	2.34
Sample_T16C_bin.5	97.31	0.34	25	44.3	Clostridiales	44.3	2.07
Sample_T5B_bin.19	90.33	0.17	105	59	Clostridiales	59	2.00
Sample_T5B_bin.14	90.24	1.34	175	42.3	Clostridiales	42.3	2.33
Sample_T5B_bin.15	98.05	1.96	309	44.9	Bacteroidales	44.9	4.52
Sample_T5B_bin.16	82.73	0.74	53	46.1	Bacteroidales	46.1	3.05
Sample_T5B_bin.17	99.44	0.24	73	49.4	Lachnospiraceae	49.4	3.12
Sample_T5B_bin.10	97.31	0.00	50	53.4	Clostridiales	53.4	1.92
Sample_T5B_bin.12	95.23	1.90	147	43.3	Clostridiales	43.3	2.91
Sample_T5B_bin.13	99.32	1.01	34	40.6	Clostridiales	40.6	2.24
Sample_C2C_bin.22	92.17	0.67	256	45.3	Clostridiales	45.3	1.77
Sample_C2C_bin.24	90.83	1.68	101	47.2	Clostridiales	47.2	2.24
Sample_T10B_bin.11	97.30	1.18	72	40.7	Bacteroidales	40.7	3.10

Sample_C6C_bin.24	88.59	1.45	26	47.7	Lachnospiraceae	47.7	2.52
Sample_C14B_bin.18	97.12	2.66	51	43.5	Lachnospiraceae	43.5	2.94
Sample_C6C_bin.29	99.03	1.33	70	42.7	Lachnospiraceae	42.7	3.43
Sample_C14B_bin.14	92.13	0.84	34	40.8	Bacteroidales	40.8	3.09
Sample_C6C_bin.25	86.90	1.57	164	62.5	Clostridiales	62.5	1.52
Sample_C14B_bin.16	98.41	0.00	85	44.6	Clostridiales	44.6	2.64
Sample_C14B_bin.10	98.65	0.67	48	36.3	Clostridiales	36.3	2.60
Sample_C6C_bin.21	97.98	0.00	250	52	Clostridiales	52	2.43
Sample_C6C_bin.22	80.80	2.97	198	60.1	Clostridiales	60.1	2.01
Sample_C14B_bin.11	99.31	0.68	268	57.5	Clostridiales	57.5	2.53
Sample_C14B_bin.12	87.85	2.43	168	49.7	Clostridiales	49.7	2.08
Sample_T6C_bin.44	83.86	0.00	368	45.3	Clostridiales	45.3	2.31
Sample_T6C_bin.43	97.92	0.00	70	50	Bacteroidales	50	2.56
Sample_T6C_bin.42	84.41	1.35	52	49	Lachnospiraceae	49	2.44
Sample_T6C_bin.41	90.15	0.34	281	42.4	Clostridiales	42.4	2.40
Sample_T14A_bin.28	98.65	0.34	73	33.7	Clostridiales	33.7	2.14
Sample_T3A_bin.7	95.77	1.05	59	46.1	Bacteroidales	46.1	4.32
Sample_T3A_bin.6	92.92	0.85	100	60.5	Clostridiales	60.5	2.18
Sample_T3A_bin.5	91.79	0.79	223	49.8	Clostridiales	49.8	2.49
Sample_T3A_bin.4	80.27	0.96	62	61.6	Clostridiales	61.6	1.95
Sample_T3A_bin.3	96.03	2.38	41	58.1	Clostridiales	58.1	2.39
Sample_T7C_bin.14	94.89	1.53	265	59.7	Clostridiales	59.7	2.68
Sample_T3A_bin.1	87.53	2.31	100	55.4	Proteobacteria	55.4	2.06
Sample_T16B_bin.23	95.94	2.30	320	44.2	Selenomonadales	44.2	2.12
Sample_T6A_bin.34	96.23	0.00	208	40.7	Clostridiales	40.7	2.57
Sample_T6A_bin.35	97.16	0.00	35	50	Bacteroidales	50	2.54
Sample_T6A_bin.36	93.63	2.82	136	44	Lachnospiraceae	44	2.43
Sample_T16B_bin.28	96.01	0.85	72	43	Lachnospiraceae	43	3.24
Sample_C2A_bin.12	96.00	4.67	163	27.3	Bacteria	27.3	1.20
Sample_T3A_bin.9	95.54	2.04	104	60.2	Clostridiales	60.2	2.01
Sample_T6A_bin.33	98.73	0.02	108	48.5	Firmicutes	48.5	2.04
Sample_C11B_bin.18	95.75	2.09	204	50.6	Enterobacteriaceae	50.6	4.56
Sample_C11B_bin.11	90.51	0.67	121	62.3	Clostridiales	62.3	1.81
Sample_T12C_bin.22	96.72	2.98	77	59.5	Clostridiales	59.5	2.36
Sample_C11B_bin.12	95.68	0.32	199	44.1	Clostridiales	44.1	3.35
Sample_C11B_bin.14	98.13	0.62	198	62.3	Proteobacteria	62.3	2.46
Sample_C11B_bin.17	94.63	0.00	24	38	Clostridiales	38	1.86
Sample_C11B_bin.16	97.59	1.44	33	54.6	Bacteroidetes	54.6	2.14
Sample_C14C_bin.13	92.50	1.15	111	45.6	Bacteroidales	45.6	3.80

Sample_C14C_bin.12	94.20	1.26	317	50.6	Enterobacteriaceae	50.6	4.45
Sample_C14C_bin.11	82.75	0.00	544	27.1	Bacteria	27.1	2.67
Sample_C14C_bin.10	99.30	0.00	220	41.2	Clostridiales	41.2	2.52
Sample_C14C_bin.17	97.67	1.27	54	47.8	Clostridiales	47.8	3.12
Sample_C8B_bin.11	89.88	2.74	279	49.9	Clostridiales	49.9	2.31
Sample_C8B_bin.12	99.32	0.00	21	35.9	Clostridiales	35.9	2.58
Sample_C8B_bin.13	84.34	0.69	295	48.5	Clostridiales	48.5	2.56
Sample_C4A_bin.21	95.70	1.01	188	36.4	Clostridiales	36.4	2.46
Sample_C4A_bin.22	89.59	0.00	61	58.3	Clostridiales	58.3	2.06
Sample_C4A_bin.23	89.26	1.01	30	56.4	Clostridiales	56.4	2.37
Sample_C4A_bin.24	97.27	0.00	29	51.8	Clostridiales	51.8	2.25
Sample_C4A_bin.25	90.38	0.48	23	54.6	Bacteroidetes	54.6	2.06
Sample_T1C_bin.4	98.30	0.53	31	41.6	Lachnospiraceae	41.6	2.76
Sample_C4B_bin.38	95.10	2.42	51	43.6	Lachnospiraceae	43.6	2.71
Sample_T17A_bin.9	91.50	0.00	388	45	Bacteria	45	3.59
Sample_T17A_bin.8	97.88	1.10	71	50.6	Enterobacteriaceae	50.6	4.75
Sample_T17A_bin.5	98.65	0.90	75	45	Bacteroidales	45	4.44
Sample_T17A_bin.4	97.37	0.00	39	44.3	Clostridiales	44.3	2.64
Sample_T17A_bin.6	87.33	1.57	277	33.7	Clostridiales	33.7	1.88
Sample_T17A_bin.1	92.39	2.92	67	43.3	Lachnospiraceae	43.3	2.79
Sample_C4B_bin.37	96.46	2.04	241	56.5	Clostridiales	56.5	2.40
Sample_C7C_bin.18	100.00	0.81	43	59.8	Actinobacteria	59.8	2.25
Sample_C7C_bin.19	99.51	1.45	60	41.2	Lachnospiraceae	41.2	3.14
Sample_C7C_bin.10	96.22	0.02	115	50.1	Bacteroidales	50.1	2.42
Sample_C7C_bin.11	85.24	3.34	30	58.8	Clostridiales	58.8	2.23
Sample_C7C_bin.12	97.98	0.67	167	33.2	Clostridiales	33.2	2.22
Sample_C7C_bin.13	98.73	2.53	57	31.1	Firmicutes	31.1	2.56
Sample_C7C_bin.15	96.30	0.41	136	36.3	Clostridiales	36.3	2.49
Sample_C7C_bin.16	98.64	3.31	91	45	Prevotella	45	3.53
Sample_C13C_bin.22	96.44	0.37	118	46.7	Bacteroidales	46.7	4.02
Sample_C14A_bin.18	96.31	2.50	50	54.5	Bacteroidetes	54.5	2.32
Sample_T11A_bin.23	99.36	0.32	24	44.7	Clostridiales	44.7	2.89
Sample_C9C_bin.6	97.58	2.58	57	46.7	Lachnospiraceae	46.7	3.49
Sample_C2B_bin.17	80.05	1.12	79	26.9	Bacteria	26.9	1.05
Sample_C7A_bin.8	84.35	0.48	492	43.5	Bacteroidales	43.5	2.67
Sample_C7A_bin.9	98.63	0.68	111	42.5	Clostridiales	42.5	3.08
Sample_C7A_bin.2	97.31	0.00	83	43.6	Clostridiales	43.6	2.09
Sample_C7A_bin.3	99.36	1.27	155	43.9	Clostridiales	43.9	3.21
Sample_C7A_bin.4	98.78	2.69	180	43.5	Selenomonadales	43.5	2.47

Sample_C7A_bin.6	100.00	1.58	158	31.2	Firmicutes	31.2	2.47
Sample_C7A_bin.7	81.82	3.51	124	58.4	Clostridiales	58.4	2.06
Sample_T11A_bin.25	97.75	0.22	97	47.8	Clostridiales	47.8	2.09
Sample_T2B_bin.44	88.18	3.06	35	44.2	Clostridiales	44.2	2.82
Sample_T2B_bin.46	95.79	1.22	106	44.9	Lachnospiraceae	44.9	2.59
Sample_T2B_bin.47	97.98	0.00	101	46	Clostridiales	46	2.29
Sample_T2B_bin.40	99.51	1.77	155	41.1	Lachnospiraceae	41.1	2.78
Sample_T2B_bin.41	89.17	2.01	50	49.5	Clostridiales	49.5	1.85
Sample_T2B_bin.43	97.98	0.00	300	36.7	Clostridiales	36.7	3.09
Sample_T14A_bin.50	95.77	1.24	171	46.8	Bacteroidales	46.8	3.97
Sample_C7A_bin.30	93.32	2.35	112	57.9	Clostridiales	57.9	2.12
Sample_C7A_bin.32	97.46	0.02	292	48.4	Firmicutes	48.4	2.13
Sample_C3A_bin.59	90.45	0.87	136	46.9	Bacteroidales	46.9	3.83
Sample_C7A_bin.34	80.99	0.67	115	33.3	Clostridiales	33.3	1.90
Sample_C7A_bin.36	95.10	1.53	295	54.3	Proteobacteria	54.3	2.15
Sample_T13A_bin.24	92.29	0.85	345	56.1	Clostridiales	56.1	2.63
Sample_T13A_bin.25	99.02	0.13	112	36.6	Lactobacillus	36.6	2.09
Sample_T13A_bin.26	99.27	0.00	109	52.2	Firmicutes	52.2	2.60
Sample_C3A_bin.51	98.38	2.04	50	46.2	Lachnospiraceae	46.2	3.08
Sample_C3A_bin.56	85.11	1.23	381	38.4	Clostridiales	38.4	2.11
Sample_C3A_bin.57	97.95	0.68	106	58.1	Clostridiales	58.1	3.40
Sample_C3A_bin.54	92.51	1.79	368	43.7	Lachnospiraceae	43.7	2.47
Sample_T13A_bin.23	99.32	1.01	65	40.8	Clostridiales	40.8	2.16
Sample_T11C_bin.17	94.51	4.28	61	46.1	Bacteroidales	46.1	3.61
Sample_T3A_bin.40	96.37	0.00	86	41.4	Lachnospiraceae	41.4	2.88
Sample_T3A_bin.41	95.55	1.50	315	47.5	Lachnospiraceae	47.5	2.88
Sample_T11C_bin.16	97.95	0.68	224	56.2	Clostridiales	56.2	2.55
Sample_T8C_bin.38	100.00	0.00	45	31.2	Euryarchaeota	31.2	2.16
Sample_T11C_bin.18	93.75	1.02	147	58.5	Clostridiales	58.5	2.44
Sample_T12B_bin.30	98.65	0.00	29	37.7	Clostridiales	37.7	2.22
Sample_C12C_bin.15	83.39	4.46	84	59	Bacteria	59	1.96
Sample_C12C_bin.14	97.98	1.01	35	51.4	Clostridiales	51.4	2.56
Sample_C12C_bin.16	95.96	0.81	20	49.6	Clostridia	49.6	1.93
Sample_C12C_bin.11	94.07	0.84	53	46.5	Bacteroidales	46.5	4.08
Sample_C12C_bin.10	84.28	0.00	143	59.7	Clostridiales	59.7	2.23
Sample_C12C_bin.13	97.97	2.48	95	44.9	Prevotella	44.9	3.66
Sample_C12C_bin.12	94.29	0.13	29	41.2	Clostridiales	41.2	2.64
Sample_T4A_bin.36	96.09	0.34	146	57.8	Clostridiales	57.8	3.49
Sample_T4A_bin.34	98.49	0.63	226	42	Bacteroidales	42	4.93

Sample_T4A_bin.35	98.41	2.00	132	44.8	Clostridiales	44.8	2.90
Sample_T4A_bin.32	99.44	0.72	41	49.3	Lachnospiraceae	49.3	3.01
Sample_T4A_bin.33	98.12	0.00	38	57.2	Proteobacteria	57.2	2.44
Sample_T4A_bin.30	97.65	0.67	172	38.3	Clostridiales	38.3	3.06
Sample_T4A_bin.31	89.93	0.67	40	60.1	Clostridiales	60.1	1.94
Sample_T4A_bin.39	99.18	0.00	19	46.7	Lactobacillales	46.7	2.09
Sample_C13B_bin.16	86.79	3.51	289	45	Lactobacillales	45	1.75
Sample_C13B_bin.15	93.20	0.58	196	44.8	Clostridiales	44.8	1.73
Sample_C13B_bin.14	98.11	0.00	172	28.9	Bacteria	28.9	2.96
Sample_C10A_bin.18	85.86	1.01	281	44.2	Clostridiales	44.2	2.32
Sample_C13B_bin.11	98.87	1.12	52	28.3	Bacteria	28.3	2.08
Sample_C13B_bin.10	90.21	1.50	290	44.2	Clostridiales	44.2	2.87
Sample_C10A_bin.15	100.00	2.03	88	44.5	Clostridiales	44.5	3.23
Sample_C10A_bin.14	95.37	2.35	45	43.9	Clostridiales	43.9	2.69
Sample_C10A_bin.17	96.01	0.67	53	37.3	Clostridiales	37.3	2.80
Sample_C10A_bin.10	99.23	0.20	126	45.4	Bacteroidales	45.4	4.01
Sample_C13B_bin.19	99.51	2.04	52	54	Bacteroidetes	54	2.23
Sample_C13B_bin.18	97.09	0.84	148	48.8	Clostridiales	48.8	2.68
Sample_C1C_bin.4	86.00	2.58	331	62	Clostridiales	62	1.82
Sample_C1C_bin.5	96.51	0.11	142	50.5	Clostridiales	50.5	2.75
Sample_C1C_bin.6	99.98	2.10	41	43.6	Selenomonadales	43.6	2.40
Sample_C1C_bin.7	97.98	0.67	94	49.8	Clostridiales	49.8	2.16
Sample_T13B_bin.25	82.48	0.99	703	45.6	Bacteroidales	45.6	3.66
Sample_C1C_bin.1	99.30	1.17	65	58	Clostridiales	58	3.11
Sample_T13B_bin.27	96.81	2.23	40	42.1	Bacteroides	42.1	4.55
Sample_C5C_bin.20	95.21	0.51	65	58.1	Clostridiales	58.1	2.23
Sample_T14B_bin.4	99.51	0.48	216	41.6	Lachnospiraceae	41.6	2.68
Sample_T13B_bin.28	86.55	4.42	210	59.9	Clostridiales	59.9	2.02
Sample_C1C_bin.8	96.42	0.00	69	56.3	Clostridiales	56.3	2.70
Sample_C1C_bin.9	84.31	0.25	444	40.9	Clostridiales	40.9	3.17
Sample_T14C_bin.20	88.34	2.93	84	57.7	Clostridiales	57.7	2.49
Sample_T18C_bin.2	96.15	2.00	153	54.6	Bacteroidetes	54.6	2.24
Sample_T7C_bin.11	91.88	0.19	191	42.3	Bacteroidales	42.3	3.73
Sample_T14C_bin.23	81.66	0.06	69	48.1	Clostridiales	48.1	1.79
Sample_T18C_bin.4	99.25	0.74	70	49.6	Bacteroidales	49.6	2.71
Sample_T18C_bin.5	90.93	0.57	109	60.1	Clostridiales	60.1	1.95
Sample_T11C_bin.46	81.51	1.65	500	29.8	Bacteria	29.8	2.32
Sample_T11C_bin.44	90.14	1.67	113	42.1	Bacteroidales	42.1	3.33
Sample_T11C_bin.45	98.10	1.58	94	41.1	Clostridiales	41.1	3.52

Sample_T11C_bin.42	92.78	0.00	103	60.1	Bacteroidetes	60.1	2.63
Sample_T11C_bin.43	84.33	2.46	239	42.3	Lachnospiraceae	42.3	2.41
Sample_T11C_bin.40	99.51	3.85	27	54.6	Bacteroidetes	54.6	2.23
Sample_T8B_bin.9	95.30	0.34	22	36.3	Clostridiales	36.3	2.14
Sample_C10A_bin.4	96.77	0.90	99	26.8	Clostridiales	26.8	2.72
Sample_C10A_bin.7	85.40	2.75	91	61.4	Clostridiales	61.4	2.07
Sample_T16B_bin.27	98.65	0.00	21	36	Clostridiales	36	2.57
Sample_C10A_bin.1	93.35	2.01	49	49.3	Clostridiales	49.3	2.14
Sample_C10A_bin.3	97.62	0.00	60	59.1	Bacteroidetes	59.1	2.93
Sample_C10A_bin.2	94.61	0.24	24	49.6	Lachnospiraceae	49.6	2.96
Sample_T8B_bin.1	98.63	0.00	389	42.3	Clostridiales	42.3	3.09
Sample_C10A_bin.8	99.32	0.38	31	36.5	Clostridiales	36.5	2.28
Sample_T3A_bin.2	95.41	2.11	75	44	Clostridiales	44	3.10
Sample_T16B_bin.22	98.79	0.48	209	58.4	Bacteroidetes	58.4	3.10
Sample_T14C_bin.28	98.73	0.02	100	48.8	Firmicutes	48.8	2.11
Sample_T12A_bin.11	95.94	0.74	378	48.2	Bacteroidales	48.2	2.60
Sample_T12A_bin.12	85.29	3.90	54	60.9	Clostridia	60.9	2.00
Sample_T12A_bin.15	94.74	1.34	104	51.7	Clostridiales	51.7	2.05
Sample_T12A_bin.17	98.01	0.00	230	51.9	Firmicutes	51.9	2.49
Sample_T12A_bin.18	92.00	0.00	39	26.5	Bacteria	26.5	1.33
Sample_T12A_bin.19	97.09	0.73	84	56.6	Clostridiales	56.6	2.40
Sample_T11A_bin.13	94.15	0.03	86	41.8	Lachnospiraceae	41.8	2.33
Sample_T11A_bin.11	97.67	1.44	52	47.9	Clostridiales	47.9	3.13
Sample_T11A_bin.17	97.91	0.10	90	60.1	Bacteroidetes	60.1	2.69
Sample_T11A_bin.16	93.39	3.06	80	58.7	Clostridiales	58.7	2.27
Sample_T11A_bin.14	81.11	0.57	351	56.8	Clostridiales	56.8	1.98
Sample_T6A_bin.30	95.23	0.23	151	61	Clostridiales	61	2.16
Sample_T11A_bin.19	91.64	1.73	130	60.2	Bifidobacteriaceae	60.2	1.91
Sample_T11A_bin.18	82.40	0.10	656	60.3	Deltaproteobacteria	60.3	3.10
Sample_T6A_bin.31	98.99	0.00	75	45.1	Clostridiales	45.1	2.70
Sample_C10B_bin.4	96.96	1.93	69	43.5	Lachnospiraceae	43.5	2.94
Sample_C2A_bin.11	87.05	0.67	23	53.3	Clostridiales	53.3	2.03
Sample_T12A_bin.42	89.93	2.35	44	61.7	Clostridiales	61.7	1.93
Sample_T3A_bin.8	97.98	0.00	96	37.5	Clostridiales	37.5	2.66
Sample_C12A_bin.41	90.28	0.00	122	51.7	Clostridiales	51.7	1.92
Sample_C12A_bin.42	100.00	0.14	151	48.4	Proteobacteria	48.4	2.64
Sample_C12A_bin.43	86.39	2.01	264	57.3	Clostridiales	57.3	1.97
Sample_C1C_bin.51	99.32	0.00	97	44	Clostridiales	44	2.81
Sample_T7A_bin.39	89.04	2.25	151	26.5	Bacteria	26.5	1.06

Sample_T7A_bin.38	92.20	0.68	182	56.5	Clostridiales	56.5	2.30
Sample_T15C_bin.31	80.80	0.67	185	59.8	Clostridiales	59.8	1.72
Sample_T7A_bin.32	94.73	2.39	146	41.3	Bacteria	41.3	3.38
Sample_C10B_bin.3	97.35	2.14	174	37.4	Clostridiales	37.4	2.73
Sample_T7A_bin.34	99.25	0.00	116	50.4	Bacteroidales	50.4	2.86
Sample_C8B_bin.14	98.52	1.01	95	39.1	Clostridiales	39.1	1.97
Sample_C8B_bin.16	97.28	1.69	46	43.7	Lachnospiraceae	43.7	2.74
Sample_C8B_bin.10	94.25	1.37	384	48.5	Proteobacteria	48.5	2.53
Sample_T8A_bin.9	100.00	0.00	139	48.6	Proteobacteria	48.6	2.66
Sample_T18B_bin.3	98.73	3.38	84	43.4	Lachnospiraceae	43.4	3.04
Sample_T18B_bin.2	92.49	2.05	134	40.9	Lachnospiraceae	40.9	3.80
Sample_T13A_bin.19	98.63	0.00	37	42.9	Clostridiales	42.9	2.76
Sample_T18B_bin.7	97.98	2.01	74	40.9	Clostridiales	40.9	1.99
Sample_C14C_bin.15	99.35	1.50	65	41.6	Lachnospiraceae	41.6	2.90
Sample_T18B_bin.4	99.32	0.67	83	54.8	Clostridiales	54.8	2.77
Sample_T4C_bin.8	99.18	0.00	19	46.7	Lactobacillales	46.7	2.08
Sample_T18B_bin.9	87.72	0.93	210	47.2	Bacteroidales	47.2	2.81
Sample_T16B_bin.39	93.87	0.70	61	60.1	Clostridiales	60.1	2.51
Sample_T1C_bin.2	95.51	2.55	158	58.4	Clostridiales	58.4	2.27
Sample_T16B_bin.33	93.77	1.48	37	49	Clostridiales	49	2.55
Sample_T16B_bin.31	97.98	1.01	163	44.2	Clostridiales	44.2	2.19
Sample_T16B_bin.37	93.94	0.74	127	42	Bacteroidales	42	4.02
Sample_T16B_bin.36	84.67	0.89	271	42.6	Clostridiales	42.6	2.32
Sample_C8C_bin.13	99.21	2.24	302	43.7	Lachnospiraceae	43.7	3.05
Sample_C14C_bin.18	98.67	1.21	98	41.4	Lachnospiraceae	41.4	3.48
Sample_C11B_bin.28	96.64	1.34	55	51.8	Clostridiales	51.8	2.58
Sample_C11B_bin.29	92.86	2.20	362	48.8	Bacteroidales	48.8	3.60
Sample_C8C_bin.12	80.92	2.40	47	59.5	Bacteroidetes	59.5	2.12
Sample_C11B_bin.24	95.93	0.00	45	51.5	Clostridiales	51.5	2.17
Sample_C11B_bin.25	87.35	1.12	50	26.6	Bacteria	26.6	1.22
Sample_C11B_bin.26	87.77	4.46	94	42.2	Bacteroidales	42.2	3.22
Sample_C11B_bin.20	97.12	0.84	127	45.9	Prevotella	45.9	2.95
Sample_T10C_bin.8	92.70	2.16	302	44.1	Clostridiales	44.1	3.13
Sample_C11B_bin.22	95.56	1.61	194	59.8	Actinobacteria	59.8	2.20
Sample_C8B_bin.19	91.94	0.02	41	62.4	Clostridiales	62.4	1.84
Sample_C10B_bin.34	86.46	1.51	108	38.5	Clostridiales	38.5	1.94
Sample_T10C_bin.9	97.05	0.77	152	41.7	Lachnospiraceae	41.7	3.13
Sample_C10B_bin.32	90.60	0.00	47	60.8	Clostridiales	60.8	2.33
Sample_C10B_bin.33	97.26	1.34	389	42.9	Clostridiales	42.9	2.77

Sample_C10B_bin.30	95.06	3.46	91	42.7	Lachnospiraceae	42.7	2.73
Sample_C10B_bin.31	92.02	0.00	57	42.4	Lachnospiraceae	42.4	2.02
Sample_T6B_bin.39	90.93	0.00	100	62.4	Clostridiales	62.4	1.87
Sample_T6B_bin.38	92.96	0.58	135	41.6	Bacteroides	41.6	5.96
Sample_T14A_bin.2	96.66	0.00	60	37.4	Clostridiales	37.4	2.64
Sample_C9B_bin.15	96.68	0.00	45	37.2	Clostridiales	37.2	2.79
Sample_C9B_bin.14	99.42	0.38	78	44.9	Bacteroidales	44.9	4.79
Sample_C9B_bin.17	90.09	0.00	57	41.6	Lachnospiraceae	41.6	2.71
Sample_C9B_bin.11	97.36	1.17	52	40.8	Lachnospiraceae	40.8	2.65
Sample_T6B_bin.34	98.65	1.01	323	38.5	Clostridiales	38.5	2.32
Sample_C9B_bin.19	92.27	0.94	51	42.6	Lachnospiraceae	42.6	3.37
Sample_C9B_bin.18	84.06	1.30	191	47.7	Bacteroidales	47.7	3.26
Sample_C6B_bin.51	90.73	0.18	279	50	Clostridiales	50	2.44
Sample_T6B_bin.31	89.85	0.63	28	42.2	Bacteroidales	42.2	3.24
Sample_T14A_bin.9	97.98	0.34	45	44.3	Clostridiales	44.3	2.06
Sample_C4B_bin.32	89.86	2.85	163	44.2	Clostridiales	44.2	2.84
Sample_C4B_bin.33	94.03	2.82	137	59.6	Bifidobacteriaceae	59.6	1.99
Sample_T6B_bin.32	98.65	0.34	236	41.8	Clostridiales	41.8	2.80
Sample_C4B_bin.31	89.19	3.08	255	37.3	Clostridiales	37.3	2.51
Sample_T2A_bin.48	96.62	1.35	101	46.1	Prevotella	46.1	3.47
Sample_C12B_bin.4	84.89	0.00	111	60	Clostridiales	60	2.07
Sample_C6A_bin.42	91.27	0.71	74	58.3	Clostridiales	58.3	2.59
Sample_C12B_bin.1	93.12	2.56	90	59.3	Clostridiales	59.3	2.11
Sample_C6B_bin.52	93.82	0.00	263	36.5	Clostridiales	36.5	3.06
Sample_C12B_bin.3	95.00	2.24	155	44.3	Bacteroidales	44.3	3.55
Sample_C6B_bin.50	96.30	0.34	308	46.4	Clostridiales	46.4	2.44
Sample_T11B_bin.12	87.84	2.35	197	59.3	Clostridiales	59.3	1.83
Sample_C14C_bin.9	98.73	0.02	110	48.3	Firmicutes	48.3	2.16
Sample_C14C_bin.8	99.19	1.44	41	54.3	Bacteroidetes	54.3	2.33
Sample_C10B_bin.8	98.43	0.67	73	43.1	Clostridiales	43.1	3.20
Sample_C10B_bin.9	90.51	4.75	287	59.3	Bacteroidetes	59.3	2.76
Sample_C14C_bin.3	94.96	1.68	27	38.2	Clostridiales	38.2	2.42
Sample_C10B_bin.7	95.95	1.34	266	45.2	Clostridiales	45.2	2.40
Sample_C14C_bin.1	97.94	0.00	45	43	Clostridiales	43	2.46
Sample_C10B_bin.5	84.37	0.00	107	35.9	Bacteria	35.9	2.55
Sample_C14C_bin.7	98.98	3.66	120	45.1	Prevotella	45.1	3.47
Sample_C14C_bin.6	93.21	2.42	146	47.4	Lachnospiraceae	47.4	2.75
Sample_C14C_bin.4	96.70	0.70	97	46.1	Bacteroidales	46.1	3.04
Sample_T11B_bin.11	95.77	3.32	140	47	Lachnospiraceae	47	3.09

Sample_C6B_bin.54	100.00	1.88	35	63.1	Actinobacteria	63.1	2.75
Sample_T13B_bin.6	93.71	1.34	121	47.5	Lachnospiraceae	47.5	2.83
Sample_C6B_bin.55	97.76	0.00	69	56.9	Clostridiales	56.9	2.28
Sample_T11B_bin.17	97.98	0.00	141	46	Clostridiales	46	2.86
Sample_T3A_bin.38	98.86	0.58	97	41.8	Lachnospiraceae	41.8	2.47
Sample_T14C_bin.31	91.68	0.26	267	43.5	Bacteroidales	43.5	2.80
Sample_T3A_bin.35	98.31	0.00	43	43.2	Bacteroidales	43.2	4.73
Sample_T3A_bin.34	96.20	0.95	81	49.2	Clostridiales	49.2	2.52
Sample_T3A_bin.37	83.05	0.65	587	41.8	Bacteroides	41.8	5.50
Sample_T13B_bin.5	96.62	0.00	88	45.1	Clostridiales	45.1	2.41
Sample_T3A_bin.31	99.32	0.67	230	40.9	Clostridiales	40.9	2.09
Sample_T3A_bin.33	89.16	0.89	133	61	Clostridiales	61	1.69
Sample_T3A_bin.32	94.57	2.94	172	39.5	Pasteurellaceae	39.5	1.92
Sample_T11B_bin.18	87.57	0.34	199	63	Clostridiales	63	1.65
Sample_T11B_bin.19	96.20	0.11	230	56.5	Clostridiales	56.5	2.35
Sample_C12C_bin.28	90.93	2.01	176	46.9	Clostridiales	46.9	2.03
Sample_C12C_bin.20	98.65	0.34	171	38.7	Clostridiales	38.7	2.27
Sample_C12C_bin.22	97.98	1.01	73	37.7	Clostridiales	37.7	3.76
Sample_C12C_bin.24	90.16	1.12	180	26.6	Bacteria	26.6	1.25
Sample_C12C_bin.26	99.62	0.38	34	43.7	Bacteroidales	43.7	3.21
Sample_C12C_bin.27	92.17	0.23	175	60.3	Clostridiales	60.3	2.53
Sample_T13A_bin.33	99.07	0.93	58	49.5	Bacteroidales	49.5	2.84
Sample_T13A_bin.32	99.36	0.63	132	49.3	Proteobacteria	49.3	2.29
Sample_T13A_bin.30	99.25	0.42	72	45.2	Bacteroidales	45.2	3.68
Sample_T13A_bin.36	97.98	0.34	31	44.6	Clostridiales	44.6	1.90
Sample_T13A_bin.35	84.59	1.81	284	37.5	Clostridiales	37.5	2.08
Sample_T13A_bin.34	90.18	0.29	382	43.5	Bacteria	43.5	3.87
Sample_C6B_bin.40	97.98	0.00	165	37.6	Clostridiales	37.6	2.59
Sample_C6B_bin.43	96.49	1.08	14	33.4	Bacteria	33.4	2.20
Sample_C6B_bin.42	95.97	0.00	122	47.7	Clostridiales	47.7	2.32
Sample_C6B_bin.45	90.74	2.95	330	42	Bacteroides	42	5.21
Sample_T7B_bin.12	91.54	2.04	117	60.6	Clostridiales	60.6	2.29
Sample_T7B_bin.13	88.36	1.61	304	60.2	Actinobacteria	60.2	1.77
Sample_T7B_bin.10	97.31	0.13	58	58.6	Clostridiales	58.6	2.18
Sample_C9A_bin.10	99.36	1.27	42	38.4	Clostridiales	38.4	2.74
Sample_T7B_bin.17	98.11	0.85	254	42.3	Bacteroidales	42.3	5.14
Sample_T7B_bin.14	97.76	0.00	112	40.6	Bacteroidales	40.6	3.23
Sample_T7B_bin.15	93.28	1.01	116	62.4	Clostridiales	62.4	1.87
Sample_T7B_bin.19	84.05	0.97	40	43.5	Lachnospiraceae	43.5	2.68

Sample_T13A_bin.12	100.00	1.61	107	59.7	Actinobacteria	59.7	2.24
Sample_T13B_bin.14	100.00	3.80	20	60	Bifidobacteriaceae	60	2.58
Sample_C10A_bin.21	93.95	0.84	121	44.6	Clostridiales	44.6	1.82
Sample_T13B_bin.16	99.62	0.28	60	50.8	Enterobacteriaceae	50.8	4.66
Sample_T13B_bin.17	92.14	1.44	31	60.3	Bacteroidetes	60.3	2.40
Sample_C10A_bin.24	96.97	0.89	107	44.7	Clostridiales	44.7	2.74
Sample_T13B_bin.12	97.31	1.01	118	52.2	Clostridiales	52.2	2.13
Sample_T13B_bin.13	94.63	1.34	74	58.1	Clostridiales	58.1	2.74
Sample_C10A_bin.29	97.98	1.01	44	38.2	Clostridiales	38.2	2.76
Sample_T2B_bin.3	98.28	0.19	305	48.4	Bacteroidales	48.4	3.99
Sample_C2C_bin.10	88.20	1.12	15	27.3	Bacteria	27.3	1.03
Sample_T2B_bin.1	96.64	0.00	39	49.9	Clostridiales	49.9	2.38
Sample_C2C_bin.12	87.68	1.50	369	53.6	Selenomonadales	53.6	2.12
Sample_C2C_bin.14	100.00	0.11	18	29.3	Bacteria	29.3	1.56
Sample_T2B_bin.5	96.42	1.03	241	57.1	Clostridiales	57.1	2.30
Sample_T2B_bin.4	84.22	0.00	38	62.3	Clostridiales	62.3	1.65
Sample_T14A_bin.16	93.11	2.33	93	41.9	Lachnospiraceae	41.9	2.96
Sample_C2C_bin.18	99.32	0.22	27	43.6	Clostridiales	43.6	2.94
Sample_T2B_bin.9	98.65	0.00	27	41	Clostridiales	41	2.57
Sample_T2B_bin.8	97.31	0.00	194	52.2	Clostridiales	52.2	3.00
Sample_T14A_bin.12	85.81	0.42	246	41.8	Clostridiales	41.8	2.74
Sample_T14A_bin.13	98.77	0.38	123	43.4	Bacteroidales	43.4	3.07
Sample_T14A_bin.10	99.30	1.17	91	57.9	Clostridiales	57.9	3.10
Sample_T10A_bin.1	97.20	1.17	31	57.9	Clostridiales	57.9	3.11
Sample_T10A_bin.2	90.42	0.56	105	38.3	Lactobacillales	38.3	2.13
Sample_T10A_bin.3	91.25	1.58	195	58.9	Deltaproteobacteria	58.9	2.36
Sample_T10A_bin.4	98.24	0.00	72	41.8	Lachnospiraceae	41.8	2.36
Sample_T10A_bin.5	97.90	0.93	30	53	Clostridiales	53	2.34
Sample_T10A_bin.7	93.97	1.41	183	28.6	Clostridiales	28.6	2.63
Sample_T10A_bin.8	82.49	0.67	153	62	Clostridiales	62	1.70
Sample_T10A_bin.9	84.70	1.52	321	58.6	Bifidobacteriaceae	58.6	2.19
Sample_C11C_bin.8	81.22	1.58	401	53.5	Clostridiales	53.5	2.28
Sample_C11C_bin.9	100.00	1.27	135	31.1	Firmicutes	31.1	2.63
Sample_T14B_bin.19	97.66	0.58	72	41.9	Lachnospiraceae	41.9	2.30
Sample_T14B_bin.18	90.09	1.37	44	43.5	Lachnospiraceae	43.5	2.74
Sample_C11C_bin.1	87.67	0.53	20	49.8	Clostridiales	49.8	2.11
Sample_C11C_bin.2	82.65	0.32	29	47.4	Clostridiales	47.4	2.47
Sample_T14B_bin.13	91.20	0.72	113	42	Lachnospiraceae	42	2.61
Sample_C11C_bin.6	80.52	2.97	355	30.8	Bacteria	30.8	1.48

Sample_C11C_bin.7	99.42	1.15	45	45.1	Bacteroidales	45.1	4.56
Sample_T12C_bin.30	93.23	1.45	81	41.8	Lachnospiraceae	41.8	2.87
Sample_T12C_bin.31	98.07	2.47	39	45.8	Bacteroidales	45.8	3.31
Sample_T2A_bin.2	84.02	2.87	30	41.8	Lachnospiraceae	41.8	2.79
Sample_T2A_bin.3	96.64	0.67	72	40.9	Clostridiales	40.9	2.55
Sample_T2A_bin.4	96.49	0.00	101	41.7	Clostridiales	41.7	2.77
Sample_T2A_bin.5	96.10	0.89	176	57.1	Clostridiales	57.1	2.26
Sample_T2A_bin.6	100.00	1.48	158	31.2	Firmicutes	31.2	2.58
Sample_T8C_bin.19	99.98	1.70	28	43.4	Selenomonadales	43.4	2.49
Sample_C4C_bin.32	97.10	1.93	211	46.8	Lachnospiraceae	46.8	3.02
Sample_C2C_bin.16	87.73	1.46	292	42.2	Lachnospiraceae	42.2	2.01
Sample_C3B_bin.30	96.85	1.21	105	41.5	Lachnospiraceae	41.5	2.62
Sample_C3B_bin.33	96.93	1.33	133	58.5	Clostridiales	58.5	3.12
Sample_T8A_bin.21	87.06	0.67	162	61.9	Clostridiales	61.9	1.94
Sample_T8A_bin.20	99.03	0.98	33	54.3	Bacteroidetes	54.3	2.23
Sample_T8A_bin.25	95.97	0.67	122	48.3	Clostridiales	48.3	2.00
Sample_T8A_bin.26	99.36	1.27	49	38.4	Clostridiales	38.4	2.76
Sample_T7A_bin.29	99.31	0.16	39	50.7	Enterobacteriaceae	50.7	4.72
Sample_T7A_bin.22	97.98	0.34	152	44.5	Clostridiales	44.5	1.99
Sample_T7A_bin.21	92.46	2.24	22	43.6	Lachnospiraceae	43.6	2.69
Sample_T7A_bin.24	93.95	2.01	173	57.4	Clostridiales	57.4	3.16
Sample_T7A_bin.25	97.53	1.70	229	55.7	Clostridiales	55.7	3.02
Sample_T6C_bin.29	98.73	0.04	113	48.5	Firmicutes	48.5	2.03
Sample_T6C_bin.28	96.81	0.64	33	38.4	Clostridiales	38.4	2.69
Sample_T6C_bin.27	99.32	1.19	65	40.8	Clostridiales	40.8	2.25
Sample_T6C_bin.26	99.30	0.67	53	36.9	Clostridiales	36.9	2.88
Sample_T6C_bin.21	94.63	2.85	18	38.5	Clostridiales	38.5	2.61
Sample_T6C_bin.20	96.30	0.00	279	45	Clostridiales	45	2.81
Sample_T6C_bin.23	98.65	0.67	248	44.2	Clostridiales	44.2	2.70
Sample_C11B_bin.8	99.40	2.15	87	47.8	Selenomonadales	47.8	2.01
Sample_C11B_bin.3	98.65	0.34	19	38.5	Clostridiales	38.5	2.10
Sample_C11B_bin.5	97.53	1.45	93	33.3	Clostridiales	33.3	2.18
Sample_C11B_bin.4	100.00	1.27	44	31.5	Firmicutes	31.5	2.29
Sample_C11B_bin.7	95.34	0.00	35	35.9	Clostridiales	35.9	2.70
Sample_C11B_bin.6	94.63	0.00	48	37.5	Clostridiales	37.5	2.60
Sample_T16B_bin.48	87.08	4.14	280	43.2	Clostridiales	43.2	3.45
Sample_T16B_bin.49	97.09	1.34	59	40.6	Clostridiales	40.6	2.78
Sample_T16B_bin.46	90.77	0.54	39	41.6	Lachnospiraceae	41.6	2.56
Sample_T16B_bin.47	95.30	0.00	64	32	Clostridiales	32	2.78

Sample_T16B_bin.44	99.28	0.95	59	44.8	Clostridiales	44.8	2.89
Sample_T16B_bin.42	96.97	0.67	173	38.9	Clostridiales	38.9	2.19
Sample_T16B_bin.43	96.87	0.62	137	60.3	Bifidobacteriaceae	60.3	2.11
Sample_T16B_bin.40	99.55	0.00	43	60.4	Bifidobacteriaceae	60.4	1.91
Sample_C10B_bin.25	80.86	4.71	215	60.5	Clostridiales	60.5	1.98
Sample_C10B_bin.24	99.03	0.85	48	42.9	Lachnospiraceae	42.9	3.32
Sample_C11B_bin.31	99.51	1.69	283	41.8	Lachnospiraceae	41.8	2.87
Sample_C11B_bin.30	90.26	0.27	54	62.3	Clostridiales	62.3	1.92
Sample_C10B_bin.21	97.31	0.00	9	53.9	Clostridiales	53.9	1.85
Sample_C11B_bin.36	89.32	2.50	150	45	Bacteroidales	45	3.07
Sample_C10B_bin.23	98.24	0.00	100	42.9	Lachnospiraceae	42.9	2.43
Sample_C10B_bin.22	95.70	2.88	142	46.8	Bacteroidales	46.8	4.04
Sample_C4A_bin.47	81.16	3.24	275	61.6	Clostridiales	61.6	1.95
Sample_C4A_bin.44	95.17	2.18	255	37.4	Clostridiales	37.4	2.25
Sample_C4A_bin.45	98.65	0.67	36	44	Clostridiales	44	3.03
Sample_C4A_bin.43	99.32	0.48	79	49.3	Lachnospiraceae	49.3	3.33
Sample_C4A_bin.40	89.66	0.48	54	59.2	Bacteroidetes	59.2	2.46
Sample_C4A_bin.41	98.63	3.07	223	56.3	Clostridiales	56.3	2.46
Sample_C5A_bin.22	92.69	4.49	99	26.1	Bacteria	26.1	1.31
Sample_T3C_bin.12	94.55	0.00	31	60.1	Clostridiales	60.1	2.51
Sample_T11C_bin.1	97.33	0.00	48	60.4	Bifidobacteriaceae	60.4	1.88
Sample_C4B_bin.12	99.32	0.34	61	38.7	Clostridiales	38.7	3.28
Sample_C4B_bin.13	94.44	0.00	307	41.7	Lachnospiraceae	41.7	2.62
Sample_C4B_bin.14	81.33	0.74	129	50.3	Clostridiales	50.3	1.91
Sample_T11C_bin.7	97.98	0.78	34	37.5	Clostridiales	37.5	2.64
Sample_T11C_bin.6	84.99	4.35	270	48.1	Lachnospiraceae	48.1	2.24
Sample_T11C_bin.9	97.98	1.01	50	38	Clostridiales	38	2.91
Sample_T11C_bin.8	91.55	1.58	99	44.9	Clostridiales	44.9	2.57
Sample_T2C_bin.39	80.02	3.24	25	27.1	Clostridiales	27.1	1.75
Sample_T17C_bin.7	99.36	0.00	34	42.4	Clostridiales	42.4	2.70
Sample_T3C_bin.19	96.88	0.67	141	42.1	Clostridiales	42.1	2.58
Sample_C2B_bin.15	93.95	0.45	77	58.4	Clostridiales	58.4	2.09
Sample_T7C_bin.1	91.50	3.06	149	46.8	Lachnospiraceae	46.8	2.89
Sample_C10C_bin.27	89.93	0.67	33	44	Clostridiales	44	2.71
Sample_C10C_bin.24	95.01	1.58	44	40.9	Clostridiales	40.9	3.43
Sample_C10C_bin.25	98.63	2.01	46	37.3	Clostridiales	37.3	2.77
Sample_C10C_bin.23	98.65	2.13	79	38.8	Clostridiales	38.8	2.25
Sample_T7C_bin.5	84.62	1.53	282	62.7	Clostridiales	62.7	1.68
Sample_C10C_bin.29	96.62	0.00	45	41.6	Clostridiales	41.6	2.95

Sample_C10A_bin.44	94.20	0.97	52	47.2	Lachnospiraceae	47.2	2.99
Sample_T7C_bin.6	90.63	0.73	247	59.4	Clostridiales	59.4	1.76
Sample_T7C_bin.9	98.10	0.65	117	48.5	Firmicutes	48.5	2.05
Sample_T7C_bin.8	90.35	1.70	66	59	Clostridiales	59	1.92
Sample_T15A_bin.15	96.61	1.69	41	45.2	Lachnospiraceae	45.2	2.38
Sample_T17B_bin.10	93.78	0.62	131	62.1	Proteobacteria	62.1	2.27
Sample_T17B_bin.12	97.58	0.97	144	50.8	Enterobacteriaceae	50.8	4.45
Sample_T17B_bin.13	99.68	1.03	36	49	Clostridiales	49	2.93
Sample_T17B_bin.15	98.83	1.17	57	43.1	Lachnospiraceae	43.1	2.84
Sample_T17B_bin.16	86.39	0.00	748	49.8	Clostridiales	49.8	5.22
Sample_T17B_bin.17	83.14	0.00	364	29.2	Bacteria	29.2	1.86
Sample_T17B_bin.18	96.04	0.63	249	40.9	Clostridiales	40.9	3.94
Sample_C2B_bin.18	96.09	1.90	193	48.5	Clostridiales	48.5	2.75
Sample_C2B_bin.19	84.04	2.66	286	47.5	Bacteroidales	47.5	3.44
Sample_C1B_bin.51	99.60	2.05	80	44.1	Selenomonadales	44.1	2.31
Sample_C1B_bin.50	91.07	3.32	98	41.6	Clostridiales	41.6	4.02
Sample_C1B_bin.53	98.63	0.00	52	42.7	Clostridiales	42.7	2.86
Sample_C1B_bin.52	91.98	1.20	63	55.1	Bacteroidetes	55.1	1.91
Sample_T14A_bin.7	94.30	1.90	91	44.8	Clostridiales	44.8	3.06
Sample_C1B_bin.57	93.26	2.35	134	57.8	Clostridiales	57.8	2.07
Sample_C1B_bin.56	97.31	0.00	34	37.2	Clostridiales	37.2	2.57
Sample_T14C_bin.25	91.49	0.67	265	37.6	Clostridiales	37.6	2.40
Sample_T2C_bin.41	99.98	1.50	26	43.5	Selenomonadales	43.5	2.57
Sample_T2C_bin.40	92.57	2.80	192	49.4	Clostridiales	49.4	1.83
Sample_T2C_bin.47	94.42	1.63	103	41.4	Lachnospiraceae	41.4	3.22
Sample_T2C_bin.46	98.38	0.00	191	43.4	Bacteria	43.4	3.59
Sample_T2C_bin.45	98.65	1.01	21	33.1	Clostridiales	33.1	2.47
Sample_T14C_bin.22	97.82	1.28	313	43.6	Lachnospiraceae	43.6	2.86
Sample_T3A_bin.26	83.62	2.92	46	43.4	Lachnospiraceae	43.4	2.40
Sample_T2C_bin.49	99.36	2.11	138	31.2	Firmicutes	31.2	2.59
Sample_T2C_bin.48	92.72	0.96	68	59.2	Bacteroidetes	59.2	2.77
Sample_T3A_bin.22	98.63	0.23	36	56.7	Bifidobacteriaceae	56.7	2.19
Sample_T3A_bin.23	98.94	0.63	192	48	Clostridiales	48	3.02
Sample_T3A_bin.20	80.79	0.00	151	41.6	Lachnospiraceae	41.6	1.76
Sample_T3A_bin.21	98.84	0.00	68	46	Bacteroidales	46	4.91
Sample_C12A_bin.44	94.51	2.12	198	45	Bacteroidales	45	3.78
Sample_T16C_bin.13	93.45	0.56	72	42.1	Bacteroidales	42.1	3.31
Sample_T16C_bin.10	99.32	0.00	29	35.7	Clostridiales	35.7	2.93
Sample_T16C_bin.16	92.62	0.02	240	37.7	Clostridiales	37.7	2.37

Sample_T16C_bin.17	92.41	2.76	303	44.2	Selenomonadales	44.2	2.02
Sample_T16C_bin.14	98.79	0.48	33	58.4	Bacteroidetes	58.4	3.16
Sample_T16C_bin.15	96.42	0.67	115	38.3	Clostridiales	38.3	3.06
Sample_C8A_bin.7	87.12	0.37	134	47.2	Bacteroidales	47.2	3.42
Sample_T4C_bin.4	97.31	0.00	165	52.8	Clostridiales	52.8	2.09
Sample_T4C_bin.5	98.79	0.41	32	41.7	Lachnospiraceae	41.7	2.66
Sample_T4C_bin.6	100.00	0.81	40	59.8	Actinobacteria	59.8	2.24
Sample_C8A_bin.6	94.35	2.85	270	42	Bacteroidales	42	3.82
Sample_T4C_bin.1	98.83	0.00	58	34.7	Lactobacillus	34.7	1.93
Sample_T4C_bin.2	83.66	0.58	223	42	Lachnospiraceae	42	2.00
Sample_T4C_bin.3	97.69	0.92	28	62.9	Bifidobacteriaceae	62.9	2.14
Sample_T12B_bin.26	90.37	0.47	75	44	Bacteroidales	44	3.65
Sample_C8A_bin.1	91.55	2.30	69	59.3	Clostridiales	59.3	1.84
Sample_T4B_bin.34	85.25	3.08	520	46.4	Bacteroidales	46.4	3.25
Sample_T2C_bin.44	99.32	0.00	33	44	Clostridiales	44	2.82
Sample_T4C_bin.9	93.64	1.29	136	44.9	Clostridiales	44.9	2.91
Sample_C8A_bin.3	97.35	0.00	111	37.3	Clostridiales	37.3	2.67
Sample_C12C_bin.39	95.30	0.00	40	37.5	Clostridiales	37.5	2.58
Sample_C12C_bin.37	99.40	1.10	57	46.7	Selenomonadales	46.7	2.36
Sample_C12C_bin.36	98.13	1.17	128	58.2	Clostridiales	58.2	3.02
Sample_C12C_bin.35	97.27	0.00	27	51.7	Clostridiales	51.7	2.27
Sample_C12C_bin.33	91.69	0.47	489	44.4	Bacteroidales	44.4	3.17
Sample_C12C_bin.32	96.63	1.75	70	54.2	Bacteroidetes	54.2	2.68
Sample_C12C_bin.30	92.61	0.94	95	54.9	Clostridiales	54.9	2.64
Sample_C6B_bin.58	97.98	0.00	26	52.1	Clostridiales	52.1	2.43
Sample_C6B_bin.59	85.29	1.12	168	42.4	Bacteroidales	42.4	3.04
Sample_T4A_bin.19	98.73	0.02	98	48.3	Firmicutes	48.3	2.13
Sample_T4A_bin.14	99.32	0.67	220	40.1	Clostridiales	40.1	2.33
Sample_C6B_bin.53	100.00	0.23	20	56.2	Bifidobacteriaceae	56.2	2.20
Sample_T4A_bin.16	100.00	0.81	43	59.8	Actinobacteria	59.8	2.22
Sample_T4A_bin.17	95.65	1.18	72	46.7	Bacteroidales	46.7	4.03
Sample_T4A_bin.10	95.97	0.08	75	58.5	Clostridiales	58.5	2.25
Sample_T4A_bin.11	96.09	0.34	258	58.6	Clostridiales	58.6	2.36
Sample_T4A_bin.12	90.61	4.70	45	44.9	Clostridiales	44.9	2.57
Sample_T4A_bin.13	99.27	0.48	28	41.5	Lachnospiraceae	41.5	2.76
Sample_T7B_bin.27	81.49	0.68	66	59.4	Clostridiales	59.4	1.94
Sample_T7B_bin.26	96.15	2.40	43	54.1	Bacteroidetes	54.1	2.22
Sample_T7B_bin.24	93.98	0.36	335	36.1	Clostridiales	36.1	2.38
Sample_T7B_bin.22	98.73	2.11	82	48.1	Clostridiales	48.1	3.01

Sample_T7B_bin.21	93.71	0.39	73	43.6	Bacteroidales	43.6	2.82
Sample_T7B_bin.20	93.75	2.40	360	59.5	Bacteroidetes	59.5	2.71
Sample_T7B_bin.29	98.32	0.00	15	41.3	Clostridiales	41.3	2.57
Sample_T7B_bin.28	99.55	0.00	48	60.4	Bifidobacteriaceae	60.4	1.91
Sample_T4A_bin.41	99.54	0.68	37	57	Bifidobacteriaceae	57	2.43
Sample_C10A_bin.36	99.54	1.25	36	56.4	Bifidobacteriaceae	56.4	2.19
Sample_C10A_bin.35	99.51	0.48	51	46.2	Bacteroidetes	46.2	2.61
Sample_C10A_bin.33	98.27	4.47	68	57.7	Bacteria	57.7	3.26
Sample_C10A_bin.31	98.75	0.63	99	55.6	Proteobacteria	55.6	2.19
Sample_C10A_bin.30	96.15	1.97	24	54.9	Bacteroidetes	54.9	2.00
Sample_C10A_bin.39	95.30	0.00	33	36.4	Clostridiales	36.4	1.80
Sample_C10A_bin.38	98.99	0.00	56	45.2	Clostridiales	45.2	2.75
Sample_C3A_bin.58	86.01	1.48	37	59.4	Clostridiales	59.4	1.50
Sample_T3C_bin.15	99.51	1.21	41	41.9	Lachnospiraceae	41.9	2.57
Sample_T3C_bin.17	96.66	1.15	44	46.2	Bacteroidales	46.2	4.20
Sample_T3C_bin.16	97.41	0.62	332	55.2	Proteobacteria	55.2	2.76
Sample_T3C_bin.11	81.46	1.48	68	48.2	Clostridiales	48.2	1.65
Sample_T3C_bin.13	99.03	0.96	134	54.6	Bacteroidetes	54.6	2.24
Sample_T4A_bin.9	99.03	0.00	127	57.6	Bacteroidetes	57.6	3.65
Sample_T4A_bin.6	93.07	0.34	100	46.4	Prevotella	46.4	3.33
Sample_T13A_bin.28	96.98	3.31	112	47.2	Lachnospiraceae	47.2	3.15
Sample_T4A_bin.2	86.70	0.00	103	43.4	Bacteria	43.4	3.34
Sample_T3C_bin.18	100.00	1.27	99	31.4	Firmicutes	31.4	2.32
Sample_T4A_bin.1	99.09	1.36	24	56.5	Clostridiales	56.5	2.27
Sample_T12C_bin.29	87.91	0.67	23	59.3	Clostridiales	59.3	2.08
Sample_T12C_bin.28	87.47	0.82	66	40.7	Bacteroidales	40.7	2.80
Sample_T12C_bin.26	87.76	3.37	194	59.3	Bacteroidetes	59.3	2.74
Sample_T12C_bin.25	97.10	2.29	56	41.6	Lachnospiraceae	41.6	2.65
Sample_T12C_bin.24	93.62	2.42	132	47.2	Lachnospiraceae	47.2	2.99
Sample_T12C_bin.23	100.00	0.63	79	31.4	Firmicutes	31.4	2.34
Sample_C3A_bin.50	98.31	2.40	88	58.5	Bacteroidetes	58.5	3.22
Sample_T12C_bin.20	97.18	0.34	159	44.1	Clostridiales	44.1	2.64
Sample_T13A_bin.27	88.37	0.45	518	45.9	Bacteroidales	45.9	3.62
Sample_T13A_bin.22	93.28	0.00	290	51	Clostridiales	51	2.44
Sample_C13C_bin.6	100.00	0.00	25	37.2	Lactobacillales	37.2	2.08
Sample_C13C_bin.7	98.65	1.51	30	36.2	Clostridiales	36.2	2.64
Sample_C13C_bin.4	89.63	0.63	281	41.8	Clostridiales	41.8	2.86
Sample_C13C_bin.5	99.03	0.16	33	41.6	Lachnospiraceae	41.6	2.62
Sample_C13C_bin.2	99.38	2.17	61	43.6	Selenomonadales	43.6	2.45

Sample_C13C_bin.1	81.29	0.38	94	45.2	Clostridiales	45.2	1.48
Sample_C13C_bin.8	97.98	0.73	84	35.9	Clostridiales	35.9	2.65
Sample_C13C_bin.9	99.41	0.19	46	43.3	Lachnospiraceae	43.3	2.75
Sample_T1A_bin.11	99.05	0.47	103	33.6	Bacteria	33.6	2.35
Sample_T6A_bin.16	92.70	1.79	266	41.2	Clostridiales	41.2	2.98
Sample_C14B_bin.8	96.28	1.18	95	45.4	Prevotella	45.4	3.19
Sample_C3B_bin.28	100.00	0.00	45	55.6	Proteobacteria	55.6	2.84
Sample_T6A_bin.17	88.99	1.01	174	39	Clostridiales	39	1.79
Sample_C14B_bin.2	99.25	2.78	40	44.4	Bacteroidales	44.4	3.65
Sample_C3B_bin.27	97.98	0.67	29	33.2	Clostridiales	33.2	2.19
Sample_C14B_bin.1	100.00	2.11	34	60.4	Bifidobacteriaceae	60.4	1.96
Sample_C3B_bin.23	98.65	0.00	21	44.4	Clostridiales	44.4	2.62
Sample_C3B_bin.20	98.10	0.65	111	48.5	Firmicutes	48.5	2.16
Sample_T8A_bin.32	86.79	0.34	239	49.9	Clostridiales	49.9	2.00
Sample_T8A_bin.33	97.30	0.84	205	33.7	Bacteria	33.7	3.16
Sample_T8A_bin.30	86.53	2.13	316	46.4	Bacteroidales	46.4	3.30
Sample_T8A_bin.36	82.82	2.75	49	47.1	Bacteroidales	47.1	3.53
Sample_T8A_bin.37	99.03	0.36	52	42.8	Lachnospiraceae	42.8	3.44
Sample_T8A_bin.34	95.80	1.01	214	36.8	Clostridiales	36.8	1.79
Sample_T8A_bin.35	84.62	0.10	63	41.7	Lachnospiraceae	41.7	2.51
Sample_T8A_bin.38	89.83	3.74	223	57.6	Clostridiales	57.6	2.62
Sample_T8A_bin.39	83.86	0.63	114	49.2	Clostridiales	49.2	2.15
Sample_T7A_bin.19	97.45	2.31	187	45.4	Bacteroidales	45.4	4.08
Sample_T7A_bin.17	94.34	0.50	279	43.4	Bacteroidales	43.4	3.02
Sample_T7A_bin.13	89.42	3.95	302	59.4	Bifidobacteriaceae	59.4	1.87
Sample_T7A_bin.10	98.63	0.00	220	42.8	Clostridiales	42.8	2.86
Sample_C3C_bin.6	94.47	0.00	68	58.6	Clostridiales	58.6	2.23
Sample_T17C_bin.8	96.89	0.62	84	62	Proteobacteria	62	2.41
Sample_T3A_bin.29	100.00	0.63	71	31.4	Firmicutes	31.4	2.34
Sample_T14A_bin.51	94.34	0.34	113	48.5	Lachnospiraceae	48.5	2.84
Sample_T18B_bin.15	99.32	3.18	109	44.8	Prevotella	44.8	3.92
Sample_T6C_bin.38	99.17	1.31	114	60	Bifidobacteriaceae	60	2.17
Sample_T6C_bin.39	99.55	0.00	36	60.4	Bifidobacteriaceae	60.4	1.92
Sample_T6C_bin.36	98.65	0.04	39	51.4	Clostridiales	51.4	2.64
Sample_T6C_bin.34	97.98	0.00	107	38.2	Clostridiales	38.2	1.85
Sample_T6C_bin.35	92.99	1.57	183	59.3	Clostridiales	59.3	1.89
Sample_T6C_bin.32	99.25	0.23	80	45.4	Bacteroidales	45.4	3.42
Sample_T6C_bin.33	95.91	2.72	16	60.7	Clostridiales	60.7	2.38
Sample_T6C_bin.30	88.35	3.71	74	58	Clostridiales	58	2.71

Sample_C14A_bin.21	95.97	0.00	55	52.3	Clostridiales	52.3	2.45
Sample_C9C_bin.17	95.09	0.78	314	44.9	Clostridiales	44.9	2.51
Sample_C9C_bin.14	98.79	1.44	45	59.2	Bacteroidetes	59.2	2.81
Sample_C9C_bin.15	84.02	0.50	605	60.1	Deltaproteobacteria	60.1	3.43
Sample_C9C_bin.12	98.23	0.51	149	45.5	Bacteroidales	45.5	3.76
Sample_C9C_bin.11	87.52	2.04	224	59.9	Clostridiales	59.9	1.93
Sample_C3C_bin.1	98.65	0.00	40	36.6	Clostridiales	36.6	1.90
Sample_C9C_bin.18	99.98	1.50	26	43.7	Selenomonadales	43.7	2.38
Sample_C9C_bin.19	87.56	2.95	334	57.9	Clostridiales	57.9	2.10
Sample_T7C_bin.21	99.51	0.96	18	54.2	Bacteroidetes	54.2	2.22
Sample_T7C_bin.22	98.65	0.00	54	37.7	Clostridiales	37.7	2.10
Sample_T7C_bin.23	99.32	1.34	180	41.4	Clostridiales	41.4	2.51
Sample_T16B_bin.50	95.56	2.37	305	41.2	Clostridiales	41.2	3.54
Sample_C6B_bin.20	95.97	3.58	116	37.4	Clostridiales	37.4	2.28
Sample_T12B_bin.10	97.09	0.00	291	44	Clostridiales	44	1.88
Sample_C6B_bin.27	93.87	2.04	216	56.3	Clostridiales	56.3	2.37
Sample_C10B_bin.18	99.32	0.67	32	40.7	Clostridiales	40.7	2.20
Sample_C11B_bin.47	88.97	1.46	713	52.1	Enterobacteriaceae	52.1	4.61
Sample_C11B_bin.44	89.95	2.54	62	47.3	Lachnospiraceae	47.3	2.56
Sample_C11B_bin.45	91.05	1.85	250	41.2	Clostridiales	41.2	1.78
Sample_C14C_bin.30	99.54	0.11	103	59.7	Bifidobacteriaceae	59.7	2.08
Sample_C10B_bin.10	99.32	0.00	94	43.9	Clostridiales	43.9	2.85
Sample_C10B_bin.12	99.98	1.50	96	44.2	Selenomonadales	44.2	2.14
Sample_C6B_bin.24	92.61	2.01	49	58	Clostridiales	58	2.78
Sample_C10B_bin.14	94.73	0.19	250	41.3	Bacteroidales	41.3	4.58
Sample_C10B_bin.15	82.04	0.50	60	46.8	Bacteroidales	46.8	2.87
Sample_C11B_bin.49	98.65	0.40	24	44	Clostridiales	44	2.80
Sample_C11C_bin.11	91.47	2.07	17	41.3	Bacteroidales	41.3	5.09
Sample_T3A_bin.24	98.65	3.02	175	37.3	Clostridiales	37.3	2.39
Sample_C11C_bin.16	88.13	1.34	268	38.3	Clostridiales	38.3	1.59
Sample_C8B_bin.33	100.00	1.27	208	31.4	Firmicutes	31.4	2.69
Sample_C9B_bin.31	97.65	0.89	73	57.3	Clostridiales	57.3	2.38
Sample_C11C_bin.15	98.06	0.48	93	41.8	Lachnospiraceae	41.8	2.65
Sample_C8C_bin.38	96.09	0.67	158	38.4	Clostridiales	38.4	2.63
Sample_T14A_bin.18	98.13	0.83	144	62.3	Proteobacteria	62.3	2.40
Sample_C2A_bin.64	97.76	1.36	120	51.8	Clostridiales	51.8	2.30
Sample_C2A_bin.65	84.85	0.62	256	48.3	Clostridiales	48.3	1.63
Sample_C2A_bin.62	86.77	1.45	213	45.2	Lachnospiraceae	45.2	2.38
Sample_C2A_bin.63	83.79	2.01	295	47.2	Clostridiales	47.2	1.78

Sample_C2A_bin.60	88.03	0.85	63	32.7	Bacteria	32.7	2.16
Sample_C8C_bin.35	95.23	0.00	51	59.9	Clostridiales	59.9	2.64
Sample_C13C_bin.14	97.50	0.12	269	49.5	Bacteroidales	49.5	2.70
Sample_T10B_bin.13	93.29	1.18	146	46	Bacteroidales	46	3.21
Sample_T1A_bin.13	98.73	0.02	94	48.5	Firmicutes	48.5	2.06
Sample_C8C_bin.30	94.64	0.17	98	60.5	Clostridiales	60.5	2.09
Sample_C10C_bin.34	92.90	3.38	253	46.3	Bacteroidales	46.3	3.89
Sample_C5A_bin.9	83.42	2.75	342	41.1	Clostridiales	41.1	2.06
Sample_C8A_bin.5	99.84	0.34	34	56.3	Bifidobacteriaceae	56.3	2.34
Sample_C10C_bin.30	98.43	1.34	102	40.8	Clostridiales	40.8	2.80
Sample_C10C_bin.33	96.99	4.44	114	41.7	Bacteroidales	41.7	5.09
Sample_T10B_bin.17	97.35	0.00	52	41.9	Clostridiales	41.9	2.75
Sample_C8C_bin.32	99.32	1.01	34	40.6	Clostridiales	40.6	2.21
Sample_C14A_bin.1	97.02	1.63	35	58.6	Clostridiales	58.6	2.69
Sample_C14A_bin.2	82.27	1.34	138	54.5	Clostridiales	54.5	1.58
Sample_C14A_bin.7	97.29	3.27	98	45.1	Prevotella	45.1	3.33
Sample_C14A_bin.6	95.06	1.25	256	57.8	Proteobacteria	57.8	2.14
Sample_C14A_bin.9	99.51	0.05	86	41	Lachnospiraceae	41	3.12
Sample_C14A_bin.8	97.94	0.00	46	43.1	Clostridiales	43.1	2.48
Sample_C3B_bin.29	97.31	1.34	14	53.6	Clostridiales	53.6	1.92
Sample_C8C_bin.7	89.84	1.34	150	40.9	Clostridiales	40.9	2.38
Sample_C8C_bin.6	99.98	2.10	25	43.6	Selenomonadales	43.6	2.41
Sample_C8C_bin.5	98.10	0.17	289	41.8	Clostridiales	41.8	2.93
Sample_C8C_bin.4	98.87	0.00	81	41.7	Lachnospiraceae	41.7	2.73
Sample_C8C_bin.2	97.98	0.34	22	46.1	Clostridiales	46.1	2.91
Sample_C8C_bin.1	85.27	2.75	94	57.2	Clostridiales	57.2	3.12
Sample_C4C_bin.51	82.66	1.33	58	27.2	Bacteria	27.2	1.10
Sample_C4C_bin.50	98.10	2.85	91	40.9	Clostridiales	40.9	3.71
Sample_C8C_bin.9	91.50	0.75	149	42.1	Bacteroidales	42.1	4.80
Sample_C2B_bin.27	99.24	0.00	43	43.4	Bacteroidales	43.4	3.16
Sample_C2B_bin.26	92.13	1.61	121	43	Clostridia	43	1.37
Sample_C2B_bin.25	91.57	2.57	22	25.9	Bacteria	25.9	1.37
Sample_T10B_bin.18	89.46	1.47	535	48.9	Bacteroidales	48.9	3.56
Sample_C2B_bin.22	98.83	0.00	60	41.8	Lachnospiraceae	41.8	2.42
Sample_C2B_bin.29	81.77	1.43	578	43.8	Bacteria	43.8	2.94
Sample_C2B_bin.28	96.58	1.63	244	63.1	Proteobacteria	63.1	2.08
Sample_C1B_bin.41	88.46	0.00	73	57.6	Clostridiales	57.6	1.92
Sample_C1B_bin.45	95.07	2.01	164	37.3	Clostridiales	37.3	2.26
Sample_C1B_bin.48	94.21	1.36	196	56.6	Clostridiales	56.6	2.22

Sample_T14C_bin.18	97.98	0.34	20	44.1	Clostridiales	44.1	2.01
Sample_T14C_bin.19	99.51	1.44	68	54.7	Bacteroidetes	54.7	2.32
Sample_T2C_bin.50	86.28	0.67	107	62.2	Clostridiales	62.2	1.71
Sample_T2C_bin.52	98.31	1.69	12	45.5	Prevotella	45.5	3.85
Sample_T14C_bin.10	99.51	0.00	121	41.3	Lachnospiraceae	41.3	3.04
Sample_T14C_bin.11	90.64	1.10	58	40.3	Bacteroidales	40.3	3.24
Sample_T14C_bin.12	98.66	0.09	63	60.4	Bifidobacteriaceae	60.4	1.92
Sample_T14C_bin.15	93.13	1.56	229	60.2	Bacteroidetes	60.2	2.61
Sample_T14C_bin.17	95.59	0.87	59	46.7	Bacteroidales	46.7	3.91
Sample_T3A_bin.13	95.58	0.24	98	42.7	Bacteroides	42.7	5.67
Sample_T3A_bin.12	98.74	2.07	75	39.8	Streptococcus	39.8	1.96
Sample_T3A_bin.11	94.86	0.00	79	57.7	Clostridiales	57.7	2.09
Sample_T3A_bin.10	95.91	0.00	85	58	Clostridiales	58	3.37
Sample_T3A_bin.16	94.12	0.58	462	45.2	Bacteroidales	45.2	4.48
Sample_T3A_bin.14	96.97	0.34	464	47.8	Clostridiales	47.8	2.09
Sample_T18A_bin.13	97.98	0.00	29	49.3	Clostridiales	49.3	2.31
Sample_T18A_bin.10	98.65	0.79	238	35.8	Clostridiales	35.8	2.67
Sample_T18A_bin.11	89.28	1.01	37	49.7	Clostridiales	49.7	2.25
Sample_T18A_bin.16	100.00	0.00	90	48.9	Proteobacteria	48.9	2.32
Sample_T18A_bin.17	92.29	1.17	105	46.7	Bacteroidales	46.7	3.20
Sample_T18A_bin.15	85.33	1.24	70	46.3	Bacteroidales	46.3	2.70
Sample_C1B_bin.5	92.31	0.97	280	44.8	Lachnospiraceae	44.8	2.72
Sample_C1B_bin.4	98.86	0.00	269	54.1	Bacteroidales	54.1	2.47
Sample_C1B_bin.7	94.21	0.41	115	58.8	Clostridiales	58.8	3.17
Sample_C1B_bin.6	94.49	0.47	321	29.2	Bacteria	29.2	2.27
Sample_C1B_bin.1	91.13	1.48	191	62.3	Clostridiales	62.3	1.91
Sample_C1B_bin.3	94.63	0.00	51	36.3	Clostridiales	36.3	2.39
Sample_C1B_bin.2	83.84	2.55	24	59.3	Clostridiales	59.3	1.81
Sample_C1B_bin.9	87.99	2.53	55	45.3	Clostridiales	45.3	2.51
Sample_C1B_bin.8	98.65	0.00	28	37.7	Clostridiales	37.7	2.20
Sample_C2A_bin.9	86.54	0.79	325	46.6	Clostridiales	46.6	2.55
Sample_C2A_bin.8	98.79	1.44	27	54.5	Bacteroidetes	54.5	2.09
Sample_C2A_bin.3	98.14	2.42	60	46.6	Bacteroidales	46.6	4.20
Sample_T7B_bin.31	94.89	0.68	315	56.4	Clostridiales	56.4	2.28
Sample_T7B_bin.32	98.63	0.00	71	42.9	Clostridiales	42.9	2.98
Sample_T7B_bin.33	99.51	1.93	72	46.5	Lachnospiraceae	46.5	3.12
Sample_C2A_bin.7	86.91	4.64	456	41.3	Clostridiales	41.3	2.66
Sample_T7B_bin.35	82.75	2.53	412	44.3	Clostridiales	44.3	2.79
Sample_T7B_bin.36	99.32	4.17	116	44.5	Prevotella	44.5	3.93

Sample_C2A_bin.4	87.69	0.00	42	57.3	Clostridiales	57.3	1.88
Sample_T8B_bin.13	85.93	0.00	106	51	Clostridiales	51	2.15
Sample_T8B_bin.15	85.13	3.78	262	60.5	Clostridiales	60.5	2.05
Sample_T8B_bin.17	97.76	0.67	67	43	Clostridiales	43	3.20
Sample_C2C_bin.37	87.24	2.68	235	41.4	Clostridiales	41.4	2.24
Sample_C2C_bin.33	89.91	1.01	154	42.7	Clostridia	42.7	1.37
Sample_C2C_bin.32	99.36	1.90	232	31.2	Firmicutes	31.2	2.61
Sample_C2C_bin.31	99.51	0.48	67	41.5	Lachnospiraceae	41.5	2.88
Sample_C2C_bin.30	98.93	0.97	167	43.4	Bacteroidales	43.4	3.09
Sample_T16A_bin.21	99.53	0.00	80	60	Bifidobacteriaceae	60	2.38
Sample_T16A_bin.20	94.11	3.60	22	57.9	Clostridiales	57.9	2.25
Sample_T16A_bin.22	100.00	0.41	184	57.3	Enterobacteriaceae	57.3	5.47
Sample_C2C_bin.39	91.82	1.42	316	59	Clostridiales	59	2.91
Sample_C2C_bin.38	97.31	1.01	60	40.6	Clostridiales	40.6	2.02
Sample_C7B_bin.3	91.28	1.88	187	41.8	Bacteroides	41.8	4.48
Sample_C7B_bin.1	91.60	0.84	189	43.2	Lachnospiraceae	43.2	2.04
Sample_T14A_bin.33	90.26	2.18	284	37.5	Clostridiales	37.5	2.05
Sample_C7B_bin.7	99.51	0.96	30	54.3	Bacteroidetes	54.3	2.33
Sample_C7B_bin.4	97.31	0.00	39	43.3	Clostridiales	43.3	2.10
Sample_T14A_bin.38	84.86	1.24	60	47.3	Bacteroidales	47.3	3.00
Sample_T14A_bin.39	97.98	0.34	74	40.5	Clostridiales	40.5	2.66
Sample_C14A_bin.15	97.65	0.06	154	45	Clostridiales	45	2.52
Sample_T10C_bin.15	96.97	1.07	178	48.7	Firmicutes	48.7	2.05
Sample_C6C_bin.48	98.63	0.00	63	43.1	Clostridiales	43.1	2.59
Sample_T11A_bin.40	98.73	0.63	183	48.8	Clostridiales	48.8	2.90
Sample_T11A_bin.41	94.06	0.02	133	59.2	Clostridiales	59.2	1.95
Sample_T11A_bin.42	96.77	1.04	197	63.1	Bifidobacteriaceae	63.1	2.38
Sample_T11A_bin.43	94.93	0.00	171	41.4	Clostridiales	41.4	3.43
Sample_T11A_bin.44	98.58	0.94	164	29.1	Bacteria	29.1	2.98
Sample_T11A_bin.47	98.65	0.00	45	53.1	Clostridiales	53.1	2.60
Sample_T11B_bin.2	98.65	1.34	88	36.9	Clostridiales	36.9	2.77
Sample_T11B_bin.4	98.94	0.63	157	47.5	Clostridiales	47.5	3.35
Sample_T11B_bin.5	90.22	3.38	98	60	Bacteroidetes	60	2.64
Sample_T11B_bin.6	97.31	1.34	108	38.1	Clostridiales	38.1	2.48
Sample_T11B_bin.8	99.32	0.00	39	34.5	Clostridiales	34.5	2.34
Sample_T11B_bin.9	96.23	0.00	162	43.2	Bacteria	43.2	4.07
Sample_T2C_bin.2	96.24	1.01	32	33.1	Clostridiales	33.1	2.54
Sample_T4B_bin.50	84.55	2.25	34	26.7	Bacteria	26.7	1.22
Sample_T4B_bin.52	88.25	2.35	74	59.4	Clostridiales	59.4	2.08

Sample_T2C_bin.6	99.51	1.22	71	41.1	Lachnospiraceae	41.1	3.00
Sample_T4B_bin.54	94.15	0.88	86	43	Clostridia	43	1.35
Sample_T2C_bin.5	97.31	1.01	73	52.9	Clostridiales	52.9	2.05
Sample_T2C_bin.8	96.59	0.68	246	56.3	Clostridiales	56.3	2.41
Sample_T2C_bin.9	97.98	0.00	59	32.2	Clostridiales	32.2	2.72
Sample_C9A_bin.6	84.56	4.70	23	52.1	Clostridiales	52.1	2.27
Sample_C9A_bin.7	98.94	0.63	58	48.1	Clostridiales	48.1	2.93
Sample_C9A_bin.4	85.85	0.45	76	43.5	Bacteria	43.5	3.05
Sample_C9A_bin.5	92.72	0.47	69	42.7	Bacteroidales	42.7	6.20
Sample_C9A_bin.3	98.26	0.38	124	44.9	Bacteroidales	44.9	4.70
Sample_C9A_bin.1	94.44	0.71	33	40.7	Lachnospiraceae	40.7	2.59
Sample_C9A_bin.8	97.14	2.63	91	41.5	Bacteroidales	41.5	5.04
Sample_C9A_bin.9	96.04	1.73	250	49	Clostridiales	49	2.71
Sample_C13A_bin.16	97.98	0.34	14	44.3	Clostridiales	44.3	1.91
Sample_C13A_bin.17	98.63	1.56	147	43.4	Lachnospiraceae	43.4	2.83
Sample_C13A_bin.15	98.72	0.64	37	38.3	Clostridiales	38.3	2.81
Sample_C13A_bin.12	98.87	1.69	300	28.4	Bacteria	28.4	2.10
Sample_C13A_bin.13	88.35	1.90	296	54.8	Bacteroidetes	54.8	1.91
Sample_C13A_bin.11	96.61	0.85	78	43	Lachnospiraceae	43	3.30
Sample_C13A_bin.18	93.93	0.16	219	26.9	Clostridiales	26.9	2.79
Sample_C13A_bin.19	98.10	0.63	45	31.3	Firmicutes	31.3	2.44
Sample_C3B_bin.13	97.76	0.67	87	44.1	Clostridiales	44.1	2.78
Sample_C3B_bin.11	98.62	0.96	87	54.8	Bacteroidetes	54.8	2.09
Sample_C3B_bin.17	94.20	3.86	90	41.6	Lachnospiraceae	41.6	2.97
Sample_C3B_bin.16	94.07	1.93	114	46.6	Lachnospiraceae	46.6	2.87
Sample_C3B_bin.14	88.38	2.31	440	46	Bacteroidales	46	3.37
Sample_C3B_bin.19	96.67	0.95	154	31.6	Firmicutes	31.6	2.20
Sample_C3B_bin.18	95.91	1.69	87	43.6	Lachnospiraceae	43.6	2.66
Sample_C10A_bin.13	83.05	2.68	155	62.1	Clostridiales	62.1	1.84
Sample_T13B_bin.21	98.64	0.00	140	52.1	Firmicutes	52.1	2.59
Sample_T13B_bin.20	99.47	1.57	112	32.6	Lactobacillales	32.6	1.99
Sample_T13B_bin.23	99.38	0.60	59	55.8	Selenomonadales	55.8	2.39
Sample_T13B_bin.22	96.21	4.26	45	40	Streptococcus	40	1.99
Sample_C3A_bin.8	85.99	0.39	657	60.3	Deltaproteobacteria	60.3	3.33
Sample_C3A_bin.9	80.22	1.53	254	59.6	Clostridiales	59.6	1.58
Sample_C3A_bin.4	85.50	0.67	73	61.7	Clostridiales	61.7	1.87
Sample_C3A_bin.5	80.97	1.68	28	42.9	Clostridiales	42.9	2.25
Sample_C3A_bin.6	96.50	0.00	51	52.9	Clostridia	52.9	2.02
Sample_C3A_bin.7	91.08	0.96	299	45.6	Bacteroidales	45.6	3.73

Sample_C3A_bin.1	84.94	1.90	47	59.2	Clostridiales	59.2	2.08
Sample_C3A_bin.2	99.72	0.29	112	55.6	Enterobacteriaceae	55.6	4.63
Sample_C3C_bin.45	85.00	0.89	157	45.2	Lachnospiraceae	45.2	2.32
Sample_C3C_bin.44	93.43	1.61	140	41.1	Lachnospiraceae	41.1	2.39
Sample_C1C_bin.2	97.58	0.74	63	40.9	Lachnospiraceae	40.9	2.66
Sample_C9C_bin.22	98.65	0.67	42	37.1	Clostridiales	37.1	2.94
Sample_T13B_bin.26	96.47	2.17	55	43.5	Lachnospiraceae	43.5	2.88
Sample_T3B_bin.16	89.56	1.67	535	45.6	Bacteroidales	45.6	4.00
Sample_T3B_bin.14	99.54	4.96	57	56.7	Bifidobacteriaceae	56.7	2.38
Sample_T3B_bin.15	99.98	1.50	22	43.6	Selenomonadales	43.6	2.39
Sample_T3B_bin.12	91.59	1.73	323	42.8	Lachnospiraceae	42.8	3.17
Sample_T3B_bin.13	97.31	0.00	169	49.2	Clostridiales	49.2	3.03
Sample_T3B_bin.10	97.27	0.00	87	58.1	Clostridiales	58.1	3.28
Sample_T3B_bin.11	88.90	0.34	56	58.4	Clostridiales	58.4	2.07
Sample_T3B_bin.19	81.48	2.45	327	59.5	Clostridiales	59.5	1.68
Sample_T10B_bin.9	90.72	3.37	98	42.9	Lachnospiraceae	42.9	3.16
Sample_T10B_bin.8	82.48	0.68	68	60.1	Clostridiales	60.1	1.78
Sample_C11B_bin.50	93.28	4.87	170	59.4	Clostridiales	59.4	2.24
Sample_T5B_bin.8	89.71	0.00	112	40.6	Clostridiales	40.6	2.24
Sample_T5B_bin.9	99.05	0.11	235	48.5	Proteobacteria	48.5	2.75
Sample_T5B_bin.6	96.64	0.22	184	51.3	Clostridiales	51.3	2.61
Sample_T5B_bin.7	98.65	0.00	63	42	Clostridiales	42	2.78
Sample_T5B_bin.4	97.89	0.42	252	44.7	Clostridiales	44.7	2.94
Sample_T10B_bin.7	83.90	3.16	145	41.9	Clostridiales	41.9	2.70
Sample_T5B_bin.3	97.96	0.00	48	41.7	Clostridiales	41.7	2.76
Sample_T10B_bin.5	95.04	4.11	157	48.8	Clostridiales	48.8	2.54
Sample_T15B_bin.4	97.42	0.10	58	41.5	Lachnospiraceae	41.5	2.67
Sample_T15B_bin.6	97.58	1.70	171	59.8	Actinobacteria	59.8	2.12
Sample_C9B_bin.28	98.63	3.88	127	58	Clostridiales	58	2.74
Sample_T15B_bin.1	81.89	1.72	75	47.1	Bacteria	47.1	2.52
Sample_T15B_bin.2	97.51	0.62	205	62.9	Proteobacteria	62.9	2.30
Sample_C9B_bin.24	89.22	2.67	65	59.6	Bacteroidetes	59.6	2.57
Sample_T15B_bin.9	97.31	0.34	64	38.4	Clostridiales	38.4	2.12
Sample_C9B_bin.22	96.20	0.63	95	44	Clostridiales	44	3.14
Sample_C9B_bin.23	98.29	1.55	173	43.8	Selenomonadales	43.8	2.26
Sample_C2A_bin.59	97.98	4.36	72	58.7	Clostridiales	58.7	2.15
Sample_C1A_bin.49	100.00	0.23	26	56.2	Bifidobacteriaceae	56.2	2.23
Sample_C1A_bin.47	87.78	0.78	40	41.7	Lachnospiraceae	41.7	2.48
Sample_C1A_bin.46	95.18	1.67	405	45.2	Bacteroidales	45.2	4.43

Sample_C1A_bin.44	93.28	1.34	61	36.3	Clostridiales	36.3	2.41
Sample_C2A_bin.57	97.20	0.93	111	58	Clostridiales	58	2.94
Sample_C1A_bin.42	98.63	0.82	22	58.7	Clostridiales	58.7	3.24
Sample_C2A_bin.55	91.22	2.56	59	42.9	Lachnospiraceae	42.9	2.32
Sample_C1A_bin.40	92.90	0.71	125	28.9	Bacteria	28.9	2.50
Sample_T18C_bin.8	99.32	2.51	128	44.7	Prevotella	44.7	4.14
Sample_T18C_bin.9	95.89	2.42	82	41.9	Lachnospiraceae	41.9	2.55
Sample_C13C_bin.24	89.98	0.62	72	38.9	Clostridiales	38.9	2.02
Sample_C13C_bin.26	93.93	0.89	173	61.2	Clostridiales	61.2	1.97
Sample_T8B_bin.8	92.29	3.24	97	45	Lachnospiraceae	45	2.55
Sample_T2A_bin.18	96.46	2.09	279	48.4	Bacteroidales	48.4	4.26
Sample_C10A_bin.6	99.32	1.68	249	40.4	Clostridiales	40.4	2.35
Sample_C4C_bin.48	97.36	0.00	63	41	Lachnospiraceae	41	2.71
Sample_C4C_bin.49	97.98	0.67	24	43.9	Clostridiales	43.9	2.17
Sample_T2A_bin.12	98.07	1.54	26	54.8	Bacteroidetes	54.8	2.16
Sample_T2A_bin.13	96.92	1.19	73	41.7	Lachnospiraceae	41.7	2.56
Sample_T2A_bin.10	96.83	1.50	87	41.4	Bacteroidales	41.4	5.27
Sample_T2A_bin.11	97.14	0.00	84	36.9	Clostridiales	36.9	2.81
Sample_T2A_bin.16	95.66	1.32	371	43.5	Bacteroidales	43.5	2.87
Sample_T2A_bin.17	95.67	0.00	65	36.3	Clostridiales	36.3	2.34
Sample_T2A_bin.14	97.58	1.81	59	42.8	Lachnospiraceae	42.8	3.32
Sample_C4C_bin.43	98.83	0.02	229	44.3	Clostridiales	44.3	2.71
Sample_C2B_bin.38	100.00	2.02	155	59.8	Actinobacteria	59.8	2.10
Sample_C2B_bin.39	96.93	0.34	68	51.5	Clostridiales	51.5	2.12
Sample_C2B_bin.31	97.04	0.00	23	52.4	Clostridiales	52.4	2.36
Sample_C2B_bin.34	93.15	0.00	66	44.9	Lachnospiraceae	44.9	2.53
Sample_C2B_bin.35	97.83	0.96	23	54.3	Bacteroidetes	54.3	2.12
Sample_C2B_bin.36	92.13	1.12	26	25.7	Bacteria	25.7	1.11
Sample_C2B_bin.37	90.59	3.91	367	44.7	Clostridiales	44.7	2.41
Sample_T8B_bin.4	80.03	2.10	5	38.2	Clostridiales	38.2	2.40
Sample_T16C_bin.34	95.63	1.81	76	38.8	Clostridiales	38.8	2.58
Sample_T16C_bin.37	89.43	1.24	108	47.2	Bacteroidales	47.2	3.74
Sample_T16C_bin.30	98.65	0.00	49	36.4	Clostridiales	36.4	2.41
Sample_T16C_bin.31	98.43	1.34	122	40.7	Clostridiales	40.7	2.74
Sample_C12A_bin.28	100.00	0.00	19	31	Euryarchaeota	31	1.75
Sample_T16C_bin.33	92.33	1.21	180	42.2	Lachnospiraceae	42.2	2.53
Sample_C12A_bin.26	94.77	0.00	40	36.1	Clostridiales	36.1	2.57
Sample_C12A_bin.27	95.07	1.34	117	46.2	Clostridiales	46.2	2.35
Sample_C12A_bin.24	83.49	1.60	192	55.1	Bacteroidetes	55.1	1.76

Sample_T8B_bin.6	82.91	0.95	329	41	Clostridiales	41	3.05
Sample_T16C_bin.38	93.82	0.00	40	26.6	Bacteria	26.6	1.19
Sample_C12A_bin.23	100.00	0.00	113	60	Bifidobacteriaceae	60	2.40
Sample_C12A_bin.21	82.49	0.67	200	57.2	Clostridiales	57.2	1.96
Sample_T15A_bin.35	98.63	1.44	25	59.6	Bacteroidetes	59.6	2.98
Sample_T15A_bin.34	95.97	0.13	157	40.5	Clostridiales	40.5	2.71
Sample_T15A_bin.37	96.05	0.90	244	45.1	Bacteroidales	45.1	4.30
Sample_C6A_bin.10	98.99	1.07	82	41.2	Clostridiales	41.2	1.89
Sample_T4B_bin.18	82.71	0.79	43	53.5	Rhodospirillales	53.5	2.43
Sample_C3A_bin.16	98.65	0.34	69	38.5	Clostridiales	38.5	2.26
Sample_T8C_bin.31	98.65	0.00	187	44.3	Clostridiales	44.3	2.62
Sample_C3A_bin.14	93.66	0.27	64	58.9	Clostridia	58.9	2.79
Sample_C3A_bin.15	92.41	1.12	49	26.7	Bacteria	26.7	1.29
Sample_C3A_bin.12	85.50	0.40	51	58.6	Clostridia	58.6	2.44
Sample_C3A_bin.13	97.27	0.00	235	45	Bacteroidales	45	4.64
Sample_C3A_bin.10	92.97	2.93	102	51.2	Clostridiales	51.2	2.21
Sample_C3A_bin.11	98.38	1.08	342	53.6	Clostridia	53.6	1.85
Sample_C3B_bin.25	98.95	0.00	50	43.7	Lactobacillales	43.7	2.15
Sample_C3A_bin.18	94.24	2.55	56	60.5	Clostridiales	60.5	2.15
Sample_T7C_bin.25	98.47	3.66	115	44.6	Prevotella	44.6	3.79
Sample_C6A_bin.19	97.46	0.63	114	48.2	Clostridiales	48.2	3.17
Sample_C1A_bin.5	94.27	0.84	180	49.1	Clostridiales	49.1	3.13
Sample_T15A_bin.38	98.83	0.16	60	55.1	Firmicutes	55.1	2.03
Sample_C12A_bin.35	92.39	0.13	90	47	Clostridiales	47	2.08
Sample_T4B_bin.13	93.28	1.34	167	59.9	Clostridiales	59.9	2.38
Sample_C12A_bin.34	91.27	0.84	77	59.6	Clostridiales	59.6	2.21
Sample_C1A_bin.1	93.24	0.96	293	38.4	Clostridiales	38.4	2.52
Sample_C12A_bin.37	98.65	0.00	16	37.8	Clostridiales	37.8	2.03
Sample_C12A_bin.36	95.97	0.94	50	58.8	Clostridiales	58.8	2.02
Sample_T16A_bin.18	89.83	0.97	304	56.3	Clostridiales	56.3	2.30
Sample_T16A_bin.19	95.14	0.63	143	48.4	Clostridiales	48.4	2.73
Sample_C2C_bin.20	93.62	0.76	17	59.1	Clostridiales	59.1	1.89
Sample_T16A_bin.15	98.65	2.75	78	36.9	Clostridiales	36.9	2.73
Sample_T16A_bin.16	80.48	1.27	269	41.7	Clostridiales	41.7	2.43
Sample_T16A_bin.10	92.23	1.17	20	45.5	Bacteroidales	45.5	3.90
Sample_T16A_bin.11	91.77	0.00	48	36	Clostridiales	36	2.51
Sample_T16A_bin.12	99.03	0.85	88	42.7	Lachnospiraceae	42.7	3.44
Sample_C2C_bin.27	97.82	1.69	40	43.6	Lachnospiraceae	43.6	2.76
Sample_T14A_bin.26	88.39	2.22	281	48.7	Clostridiales	48.7	2.48

Sample_T14A_bin.25	93.47	1.11	190	38.3	Clostridiales	38.3	2.63
Sample_T14A_bin.22	99.32	0.31	51	42.5	Clostridiales	42.5	3.07
Sample_T14A_bin.20	100.00	1.27	20	31.3	Firmicutes	31.3	2.55
Sample_T3C_bin.33	80.57	0.00	359	35.8	Clostridiales	35.8	2.28
Sample_T3C_bin.30	98.73	0.89	21	49.2	Clostridiales	49.2	2.90
Sample_T3C_bin.37	98.65	0.00	23	43.7	Clostridiales	43.7	3.04
Sample_T14A_bin.29	84.26	1.12	139	26.2	Bacteria	26.2	1.04
Sample_T3C_bin.34	87.42	1.90	50	49.5	Clostridiales	49.5	2.19
Sample_C7C_bin.1	90.98	2.72	180	59.2	Clostridiales	59.2	1.97
Sample_C7C_bin.6	98.73	0.02	113	48.5	Firmicutes	48.5	2.17
Sample_C7C_bin.7	98.75	0.63	34	54.3	Proteobacteria	54.3	2.33
Sample_C7C_bin.5	87.06	0.51	80	59.8	Clostridiales	59.8	1.92
Sample_C7C_bin.8	81.82	0.95	404	44.1	Clostridiales	44.1	2.83
Sample_C10B_bin.19	98.32	0.00	137	37.2	Clostridiales	37.2	2.42
Sample_T10B_bin.34	83.41	0.67	353	44.9	Clostridiales	44.9	2.12
Sample_T6B_bin.30	99.94	0.11	92	49.2	Proteobacteria	49.2	2.36
Sample_T4B_bin.42	93.54	0.81	474	52.6	Clostridia	52.6	2.24
Sample_T4B_bin.43	94.72	1.89	73	51.2	Enterobacteriaceae	51.2	4.08
Sample_T4B_bin.40	100.00	0.00	47	31.3	Euryarchaeota	31.3	1.70
Sample_T4B_bin.41	97.04	0.00	52	52.6	Clostridiales	52.6	2.34
Sample_T4B_bin.46	98.10	0.34	109	48.4	Firmicutes	48.4	2.11
Sample_T4B_bin.47	88.25	0.67	195	53.8	Clostridiales	53.8	1.87
Sample_T4B_bin.44	99.32	1.68	49	39.9	Clostridiales	39.9	2.56
Sample_T4B_bin.45	84.65	2.64	31	26.3	Bacteria	26.3	1.08
Sample_T4B_bin.48	98.65	0.74	59	58.6	Clostridiales	58.6	2.13
Sample_C11B_bin.41	97.73	0.37	211	49.7	Bacteroidales	49.7	2.67
Sample_T6B_bin.10	99.42	0.38	44	45.1	Bacteroidales	45.1	4.55
Sample_T3C_bin.35	96.66	0.00	24	37.3	Clostridiales	37.3	2.70
Sample_T13B_bin.3	86.87	2.35	229	59.7	Bacteroidetes	59.7	2.46
Sample_T8A_bin.18	89.26	3.13	133	60.5	Clostridiales	60.5	1.88
Sample_T8A_bin.19	87.40	0.77	317	48.7	Lachnospiraceae	48.7	2.78
Sample_C12B_bin.8	98.83	1.17	152	57.8	Clostridiales	57.8	3.11
Sample_C12B_bin.9	97.30	0.90	55	46.8	Selenomonadales	46.8	2.29
Sample_T8A_bin.10	98.38	0.81	139	60	Actinobacteria	60	2.19
Sample_C12B_bin.5	98.63	0.00	63	42.7	Clostridiales	42.7	3.01
Sample_C12B_bin.6	95.05	2.42	199	47.3	Lachnospiraceae	47.3	2.98
Sample_C6A_bin.43	97.31	0.00	76	47	Clostridiales	47	2.66
Sample_T8A_bin.14	95.89	0.00	49	41.6	Lachnospiraceae	41.6	2.66
Sample_T8A_bin.15	96.64	0.02	142	37.6	Clostridiales	37.6	2.56

Sample_T8A_bin.16	93.12	2.20	158	41.8	Bacteroidales	41.8	3.68
Sample_T8A_bin.17	98.30	0.00	58	50	Bacteroidales	50	2.46
Sample_T13B_bin.2	99.32	2.01	38	37	Clostridiales	37	2.64
Sample_T6C_bin.15	99.51	3.64	24	41	Lachnospiraceae	41	2.66
Sample_T13B_bin.1	99.32	2.20	9	40.5	Clostridiales	40.5	2.30
Sample_T6C_bin.10	93.93	2.01	41	49.5	Clostridiales	49.5	2.10
Sample_T13B_bin.7	95.78	1.55	320	56.5	Clostridiales	56.5	2.45
Sample_T6C_bin.12	99.51	0.48	243	41.6	Lachnospiraceae	41.6	2.74
Sample_T6C_bin.13	93.65	2.06	40	56.3	Clostridiales	56.3	2.39
Sample_T13B_bin.8	98.95	0.96	103	58.7	Bacteroidetes	58.7	2.53
Sample_T13B_bin.9	98.63	0.68	35	43	Clostridiales	43	2.76
Sample_T6C_bin.18	90.35	3.46	64	46.6	Bacteroidales	46.6	4.05
Sample_C3B_bin.5	90.96	0.62	377	45	Clostridiales	45	2.26
Sample_C3B_bin.3	98.73	1.48	47	48.2	Clostridiales	48.2	2.95
Sample_C3B_bin.9	92.41	2.52	139	37.5	Clostridiales	37.5	2.39
Sample_C9B_bin.9	99.23	0.00	64	45	Bacteroidales	45	4.02
Sample_C9B_bin.8	99.96	0.43	130	50.5	Enterobacteriaceae	50.5	4.90
Sample_C9B_bin.5	95.89	2.96	134	44.5	Bacteroidales	44.5	4.18
Sample_C9B_bin.4	97.98	0.13	24	35.9	Clostridiales	35.9	2.58
Sample_C9B_bin.7	98.55	3.06	53	46.7	Lachnospiraceae	46.7	3.45
Sample_C9B_bin.1	97.76	3.37	53	42.7	Bacteroidales	42.7	6.68
Sample_C9B_bin.3	98.94	0.63	174	48.1	Clostridiales	48.1	3.19
Sample_T6B_bin.15	93.56	0.58	54	41.9	Lachnospiraceae	41.9	2.29
Sample_C5A_bin.28	92.61	1.54	54	62.5	Clostridiales	62.5	1.90
Sample_C5A_bin.23	94.14	1.13	287	41	Clostridiales	41	3.45
Sample_T1C_bin.9	99.05	0.47	121	33.6	Bacteria	33.6	2.28
Sample_C5A_bin.25	97.27	0.68	224	56.3	Clostridiales	56.3	2.40
Sample_C5A_bin.24	80.59	2.35	79	59.2	Clostridiales	59.2	2.08
Sample_C5A_bin.27	98.65	0.00	26	37.6	Clostridiales	37.6	2.19
Sample_C3A_bin.28	81.46	3.52	283	62	Clostridiales	62	1.76
Sample_T3B_bin.29	84.33	0.67	465	60.6	Clostridiales	60.6	2.65
Sample_T3B_bin.28	98.65	0.00	23	38	Clostridiales	38	1.96
Sample_C8A_bin.9	90.32	2.61	101	59.8	Clostridiales	59.8	1.86
Sample_C8A_bin.8	98.30	0.48	50	41.7	Lachnospiraceae	41.7	2.56
Sample_T3B_bin.23	91.45	1.51	66	61.4	Clostridiales	61.4	2.02
Sample_T3B_bin.22	98.94	1.79	73	47.9	Clostridiales	47.9	3.06
Sample_T3B_bin.21	99.32	1.34	109	40.7	Clostridiales	40.7	2.12
Sample_T3B_bin.20	92.48	2.71	41	37.3	Clostridiales	37.3	2.28
Sample_T3B_bin.27	99.27	0.00	63	55	Bacteroidetes	55	2.68

Sample_T3B_bin.26	89.57	1.44	222	55.8	Bacteroidetes	55.8	1.74
Sample_T3B_bin.25	100.00	2.85	221	31.2	Firmicutes	31.2	2.61
Sample_C8A_bin.2	88.60	4.12	60	57.8	Clostridiales	57.8	2.28
Sample_T12A_bin.6	97.31	1.34	104	37.5	Clostridiales	37.5	2.06
Sample_T12A_bin.7	94.92	2.28	227	44.1	Lachnospiraceae	44.1	2.90
Sample_T12A_bin.5	97.31	0.67	38	54.1	Clostridiales	54.1	2.02
Sample_T12A_bin.2	96.29	0.63	279	55.6	Proteobacteria	55.6	2.20
Sample_T12A_bin.3	80.61	1.87	219	44.1	Clostridiales	44.1	2.39
Sample_T12A_bin.1	97.98	0.34	53	38.7	Clostridiales	38.7	2.01
Sample_T13B_bin.4	99.15	0.19	35	37.5	Lactobacillales	37.5	2.77
Sample_T12A_bin.8	94.14	0.91	307	59.3	Clostridiales	59.3	2.87
Sample_T12A_bin.9	93.95	0.84	215	39.9	Clostridiales	39.9	2.13
Sample_C4B_bin.49	97.90	1.17	83	58.1	Clostridiales	58.1	3.04
Sample_C4B_bin.43	89.93	0.00	301	36.3	Clostridiales	36.3	2.40
Sample_C4B_bin.42	98.73	0.02	31	48.3	Firmicutes	48.3	2.17
Sample_C4B_bin.47	82.60	0.06	368	52.4	Clostridiales	52.4	2.08
Sample_C4B_bin.46	98.94	1.48	106	47.9	Clostridiales	47.9	3.17
Sample_C4B_bin.44	91.95	0.71	456	43.4	Clostridiales	43.4	2.27
Sample_C11C_bin.18	91.04	2.18	110	42.2	Bacteroides	42.2	4.73
Sample_C11C_bin.19	97.66	0.05	85	41.7	Lachnospiraceae	41.7	2.51
Sample_C2A_bin.48	88.96	0.40	145	60.2	Clostridia	60.2	2.26
Sample_C2A_bin.44	98.43	0.47	57	56.9	Clostridiales	56.9	2.45
Sample_C11C_bin.13	99.32	1.01	301	40.7	Clostridiales	40.7	2.14
Sample_C2A_bin.46	80.80	0.89	148	62	Clostridiales	62	1.81
Sample_C2A_bin.47	89.92	3.04	480	43.4	Bacteroidales	43.4	2.70
Sample_C2A_bin.40	93.25	3.93	50	25.5	Bacteria	25.5	1.35
Sample_C11C_bin.17	89.74	0.85	11	32.1	Bacteria	32.1	1.85
Sample_C2A_bin.42	96.64	2.01	237	34	Clostridiales	34	2.51
Sample_C2A_bin.43	90.76	2.72	77	60.1	Clostridiales	60.1	1.88
Sample_C1A_bin.51	85.81	0.00	368	42	Clostridiales	42	2.67
Sample_C1A_bin.52	84.26	3.90	25	49.6	Clostridiales	49.6	2.18
Sample_C1A_bin.53	98.65	0.00	36	37.7	Clostridiales	37.7	2.15
Sample_C1A_bin.54	99.32	0.34	234	36.5	Clostridiales	36.5	2.12
Sample_T11B_bin.27	96.97	1.34	18	33.8	Clostridiales	33.8	2.27
Sample_T14B_bin.10	91.23	2.43	110	46.5	Bacteroidales	46.5	3.10
Sample_C8B_bin.36	95.19	1.34	129	33.2	Clostridiales	33.2	2.16
Sample_C10C_bin.14	98.12	0.10	260	57.4	Proteobacteria	57.4	2.40
Sample_C10C_bin.13	97.31	0.06	112	53.9	Clostridiales	53.9	1.84
Sample_C10C_bin.11	97.98	1.48	9	33.1	Clostridiales	33.1	2.18

Sample_T2A_bin.29	94.30	2.27	191	44.2	Clostridiales	44.2	3.02
Sample_T2A_bin.28	97.31	0.67	10	53.2	Clostridiales	53.2	1.94
Sample_T2A_bin.27	99.25	3.07	88	44.8	Bacteroidales	44.8	3.81
Sample_T2A_bin.26	98.99	0.00	54	45.2	Clostridiales	45.2	2.75
Sample_T2A_bin.24	96.71	0.00	361	33.1	Clostridiales	33.1	2.60
Sample_T2A_bin.23	94.53	1.22	140	43.1	Lachnospiraceae	43.1	2.19
Sample_T2A_bin.22	89.39	1.27	147	49	Clostridiales	49	2.45
Sample_T2A_bin.21	99.77	0.00	245	43.6	Clostridiales	43.6	3.04
Sample_T2A_bin.20	99.51	1.22	56	40.9	Lachnospiraceae	40.9	2.67
Sample_T12B_bin.15	96.26	0.53	42	46	Bacteroidales	46	3.18
Sample_C2B_bin.48	96.83	0.65	99	48.7	Firmicutes	48.7	2.14
Sample_T12B_bin.14	98.41	3.29	122	44.2	Clostridiales	44.2	3.13
Sample_C2B_bin.45	92.17	0.67	19	45.2	Clostridiales	45.2	1.71
Sample_C2B_bin.47	95.97	1.01	46	40.7	Clostridiales	40.7	2.09
Sample_C2B_bin.41	96.32	0.00	74	37.5	Clostridiales	37.5	2.57
Sample_T16A_bin.8	99.25	0.19	113	37.6	Lactobacillales	37.6	2.75
Sample_C2B_bin.43	98.18	0.00	52	41.8	Clostridiales	41.8	3.00
Sample_T12B_bin.5	90.25	1.40	380	42.3	Clostridiales	42.3	2.37
Sample_T12B_bin.16	83.53	1.40	183	45.6	Prevotella	45.6	2.05
Sample_C13C_bin.25	94.92	1.45	81	41.8	Lachnospiraceae	41.8	3.05
Sample_C6C_bin.4	91.04	0.93	35	44.5	Bacteroidales	44.5	3.05
Sample_C6C_bin.7	97.90	0.04	91	56.5	Clostridiales	56.5	2.34
Sample_C6C_bin.6	96.93	1.44	90	59.8	Bacteroidetes	59.8	2.59
Sample_C13C_bin.21	97.98	1.76	62	59.4	Clostridiales	59.4	2.28
Sample_C13C_bin.20	94.32	1.72	144	41.6	Lachnospiraceae	41.6	3.36
Sample_C13C_bin.23	97.04	0.00	276	49	Clostridiales	49	2.72
Sample_C6C_bin.2	97.31	0.00	27	54	Clostridiales	54	1.83
Sample_T16C_bin.29	93.03	0.63	58	44.7	Clostridiales	44.7	2.55
Sample_T16C_bin.28	97.31	1.74	117	38.2	Clostridiales	38.2	2.56
Sample_C12A_bin.39	98.38	0.81	19	59.8	Actinobacteria	59.8	2.21
Sample_T12B_bin.3	98.65	0.08	46	43.8	Clostridiales	43.8	2.81
Sample_T16C_bin.23	100.00	0.00	142	60.4	Bifidobacteriaceae	60.4	1.91
Sample_T16C_bin.22	99.51	0.06	20	41.5	Lachnospiraceae	41.5	2.83
Sample_T16C_bin.21	98.65	0.67	40	33.1	Clostridiales	33.1	2.53
Sample_T1C_bin.1	92.67	4.59	110	44.1	Clostridiales	44.1	3.27
Sample_T16C_bin.27	91.07	0.59	59	46.4	Bacteroidales	46.4	3.48
Sample_T16C_bin.26	84.05	1.13	103	59.8	Clostridiales	59.8	2.01
Sample_T16C_bin.25	89.45	0.94	174	49.3	Clostridiales	49.3	2.28
Sample_T16C_bin.24	99.32	0.34	160	38.9	Clostridiales	38.9	2.19

Sample_T16A_bin.1	96.15	2.87	590	46.3	Bacteroidales	46.3	3.94
Sample_T16A_bin.6	99.36	0.63	47	44	Clostridiales	44	3.48
Sample_T6C_bin.6	97.27	0.06	44	51.3	Clostridiales	51.3	2.17
Sample_T6C_bin.7	80.68	1.92	621	57.6	Bacteroidetes	57.6	2.99
Sample_T6C_bin.4	96.38	1.15	169	45.3	Bacteroidales	45.3	4.27
Sample_T6C_bin.5	98.65	0.00	35	41.7	Clostridiales	41.7	2.77
Sample_T6C_bin.2	93.03	1.64	83	41.3	Clostridiales	41.3	2.81
Sample_T6C_bin.3	86.78	0.97	510	47.5	Lachnospiraceae	47.5	2.44
Sample_T6C_bin.1	90.60	0.00	33	60.3	Clostridiales	60.3	1.90
Sample_C3A_bin.23	92.48	1.83	40	41.8	Bacteroides	41.8	4.48
Sample_C3A_bin.22	83.26	1.34	76	58.6	Clostridiales	58.6	1.53
Sample_C3A_bin.20	96.23	0.00	64	43.4	Bacteria	43.4	3.79
Sample_C3A_bin.26	94.51	1.26	252	48.3	Proteobacteria	48.3	2.65
Sample_C3A_bin.25	89.20	2.01	211	59.4	Clostridiales	59.4	2.08
Sample_C3A_bin.24	81.87	1.52	71	41	Lachnospiraceae	41	1.95
Sample_T17A_bin.15	99.32	4.03	68	44.7	Clostridiales	44.7	3.04
Sample_T17A_bin.14	99.98	1.50	131	43.7	Selenomonadales	43.7	2.42
Sample_T17A_bin.11	97.12	0.63	44	44.1	Clostridiales	44.1	3.51
Sample_T17A_bin.13	97.71	0.16	25	38.6	Clostridiales	38.6	2.46
Sample_T1C_bin.7	100.00	0.63	95	31.3	Firmicutes	31.3	2.37
Sample_C12C_bin.52	92.61	0.00	23	42.2	Clostridiales	42.2	1.75
Sample_T17A_bin.19	95.80	0.84	37	46.9	Clostridiales	46.9	3.06
Sample_T17A_bin.18	97.02	0.70	49	40.7	Bacteroidales	40.7	3.61
Sample_C8C_bin.51	95.00	0.00	54	43.6	Proteobacteria	43.6	2.28
Sample_C8C_bin.50	87.85	0.27	266	60.4	Bacteroidetes	60.4	2.36
Sample_T8B_bin.38	80.78	1.02	427	55.8	Bacteria	55.8	2.21
Sample_T8B_bin.36	99.40	0.80	39	46.7	Selenomonadales	46.7	2.37
Sample_T8B_bin.35	95.86	0.67	157	38.1	Clostridiales	38.1	1.80
Sample_T8B_bin.34	95.43	2.79	64	45.2	Bacteroidales	45.2	3.76
Sample_T8B_bin.32	81.99	2.48	383	40.1	Lachnospiraceae	40.1	2.21
Sample_T8B_bin.31	96.36	0.77	58	48.5	Lachnospiraceae	48.5	2.89
Sample_T8B_bin.30	91.82	0.96	27	54.9	Bacteroidetes	54.9	1.96
Sample_C4B_bin.8	97.72	0.89	84	32.7	Clostridiales	32.7	2.06
Sample_C4B_bin.9	90.82	1.38	256	49.5	Clostridiales	49.5	2.40
Sample_C4B_bin.2	98.65	0.00	68	44.2	Clostridiales	44.2	2.84
Sample_C4B_bin.1	96.15	3.37	435	55	Bacteroidetes	55	2.09
Sample_C4B_bin.6	99.36	0.00	97	44.4	Clostridiales	44.4	2.84
Sample_C4B_bin.7	98.65	0.00	21	36.4	Clostridiales	36.4	1.87
Sample_C4B_bin.4	85.78	2.01	102	52.5	Clostridiales	52.5	2.70

Sample_T3C_bin.28	84.48	1.72	80	40.7	Bacteria	40.7	3.25
Sample_T3C_bin.29	98.06	2.42	115	46.5	Lachnospiraceae	46.5	3.63
Sample_T14C_bin.8	94.05	0.00	177	42	Clostridiales	42	2.61
Sample_T14C_bin.9	98.75	0.63	31	55.8	Proteobacteria	55.8	2.11
Sample_T14C_bin.6	95.48	1.18	162	56.7	Bifidobacteriaceae	56.7	1.87
Sample_T14C_bin.7	96.78	0.68	213	45.7	Bacteroidales	45.7	3.75
Sample_T14C_bin.4	99.30	1.17	98	57.6	Clostridiales	57.6	3.17
Sample_T14C_bin.5	99.32	0.57	32	42.6	Clostridiales	42.6	3.09
Sample_T14C_bin.2	99.03	2.42	193	46.3	Lachnospiraceae	46.3	3.12
Sample_T14C_bin.1	97.98	0.00	96	36.5	Clostridiales	36.5	1.93
Sample_T10A_bin.12	95.99	3.48	83	45.5	Firmicutes	45.5	1.67
Sample_T10A_bin.13	97.58	0.81	36	63	Actinobacteria	63	2.68
Sample_T10A_bin.10	99.51	1.22	156	40.7	Lachnospiraceae	40.7	2.82
Sample_T10A_bin.16	96.63	1.28	108	55.2	Bacteroidetes	55.2	2.00
Sample_T10A_bin.17	96.31	0.75	94	42	Bacteroidales	42	4.72
Sample_T10A_bin.14	86.58	0.00	94	59.4	Bacteroidetes	59.4	2.43
Sample_T10A_bin.15	93.93	1.34	45	49.5	Clostridiales	49.5	1.99
Sample_T10A_bin.19	94.29	0.67	17	58.2	Clostridiales	58.2	1.84
Sample_C8B_bin.6	98.10	0.02	95	48.5	Firmicutes	48.5	2.07
Sample_C8B_bin.7	97.65	0.00	57	37.4	Clostridiales	37.4	2.63
Sample_C8B_bin.4	98.38	0.89	219	41.9	Lachnospiraceae	41.9	2.53
Sample_C13B_bin.30	84.05	2.82	96	37.4	Clostridiales	37.4	3.91
Sample_C8B_bin.3	96.20	2.29	32	44.2	Clostridiales	44.2	3.24
Sample_C8B_bin.9	97.39	0.37	89	48.2	Bacteroidales	48.2	3.70
Sample_T13B_bin.50	93.66	0.84	521	33.3	Bacteria	33.3	2.21
Sample_T13B_bin.51	87.88	4.15	126	40.5	Bacteroidales	40.5	3.35
Sample_T13B_bin.52	84.27	0.35	612	28.7	Clostridiales	28.7	2.28
Sample_T13B_bin.53	80.12	0.03	38	60.5	Deltaproteobacteria	60.5	2.83
Sample_T13B_bin.54	97.98	0.34	140	44.2	Clostridiales	44.2	2.07
Sample_T13B_bin.55	83.27	0.48	289	48.4	Lachnospiraceae	48.4	2.38
Sample_T13B_bin.56	98.51	0.42	69	45.3	Bacteroidales	45.3	3.70
Sample_T13B_bin.58	100.00	0.56	18	60.4	Bifidobacteriaceae	60.4	1.91
Sample_T13C_bin.20	97.05	0.68	330	56.4	Clostridiales	56.4	2.43
Sample_T13C_bin.21	89.37	2.35	40	37.6	Clostridiales	37.6	2.55
Sample_T13C_bin.26	97.44	0.09	92	60.4	Bifidobacteriaceae	60.4	1.90
Sample_T13C_bin.27	99.51	0.48	39	41.5	Lachnospiraceae	41.5	2.82
Sample_T13C_bin.24	98.63	0.00	68	42.7	Clostridiales	42.7	2.83
Sample_T13C_bin.25	99.27	0.00	111	52.3	Firmicutes	52.3	2.57
Sample_T13C_bin.28	87.01	0.96	41	60.6	Bacteroidetes	60.6	2.16

Sample_T13C_bin.29	97.32	4.17	79	56.4	Bifidobacteriaceae	56.4	2.29
Sample_C13A_bin.30	97.98	2.01	293	61.1	Clostridiales	61.1	2.21
Sample_C13A_bin.31	91.69	2.53	102	41.8	Clostridiales	41.8	3.00
Sample_C13A_bin.32	97.84	0.10	36	51.3	Bacteria	51.3	1.80
Sample_C13A_bin.33	100.00	1.09	173	48.3	Clostridiales	48.3	2.82
Sample_C13A_bin.34	97.75	2.81	163	30.5	Bacteria	30.5	3.32
Sample_C13A_bin.35	94.86	0.90	519	45.1	Bacteroidales	45.1	4.39
Sample_C13A_bin.36	93.62	0.63	213	43.2	Bacteroidales	43.2	4.67
Sample_C13A_bin.37	98.65	0.34	31	38.8	Clostridiales	38.8	2.13
Sample_C12A_bin.32	90.18	3.34	56	44.8	Prevotella	44.8	3.34
Sample_C6A_bin.35	81.18	4.85	135	58.9	Clostridiales	58.9	2.16
Sample_C6A_bin.34	84.66	0.73	207	43.5	Clostridiales	43.5	2.58
Sample_C6A_bin.37	97.31	0.00	33	44.4	Clostridiales	44.4	2.63
Sample_C6A_bin.36	92.19	3.85	71	60.4	Bacteroidetes	60.4	2.49
Sample_C6A_bin.31	96.27	1.72	269	43.5	Bacteroidales	43.5	2.89
Sample_T7A_bin.33	97.98	0.00	76	52.3	Clostridiales	52.3	3.07
Sample_C6A_bin.32	99.32	1.13	62	50.8	Prevotella	50.8	3.44
Sample_T3C_bin.25	96.77	1.97	186	41.8	Bacteria	41.8	3.84
Sample_T3C_bin.27	99.98	2.10	21	43.5	Selenomonadales	43.5	2.40
Sample_T6A_bin.8	98.63	0.00	30	43.2	Clostridiales	43.2	2.54
Sample_T6A_bin.9	97.31	0.00	64	44.3	Clostridiales	44.3	2.65
Sample_T3C_bin.20	95.78	1.27	120	48.3	Clostridiales	48.3	2.81
Sample_T6A_bin.7	89.41	2.02	120	40.9	Lachnospiraceae	40.9	2.36
Sample_T6A_bin.1	95.97	1.28	42	46.8	Clostridiales	46.8	2.73
Sample_T6A_bin.2	94.33	1.92	76	58.3	Bacteroidetes	58.3	3.14
Sample_T15C_bin.40	87.28	2.85	383	46.3	Bacteroidales	46.3	3.60
Sample_T3C_bin.23	96.13	1.21	37	43.6	Lachnospiraceae	43.6	2.75
Sample_C5A_bin.38	80.55	0.23	234	60.3	Bifidobacteriaceae	60.3	1.73
Sample_C5A_bin.39	98.63	0.00	59	42.8	Clostridiales	42.8	2.85
Sample_C3C_bin.29	89.76	0.00	192	46	Bacteroidales	46	3.51
Sample_C3C_bin.28	100.00	0.00	116	55.5	Proteobacteria	55.5	2.75
Sample_C3C_bin.25	86.44	1.68	182	58.5	Clostridiales	58.5	1.74
Sample_C3C_bin.24	85.25	4.48	207	58.8	Clostridiales	58.8	2.07
Sample_C3C_bin.26	98.65	0.34	52	38.6	Clostridiales	38.6	2.31
Sample_C5A_bin.36	86.79	2.58	479	38.5	Bacteroidales	38.5	2.94
Sample_C3C_bin.20	95.67	1.92	67	54.5	Bacteroidetes	54.5	2.12
Sample_C3C_bin.23	84.75	1.01	220	59.7	Clostridiales	59.7	1.99
Sample_C3C_bin.22	98.65	2.68	31	42	Clostridiales	42	2.68
Sample_T3B_bin.35	82.75	1.72	150	42	Bacteria	42	4.74

Sample_T3B_bin.31	99.41	0.00	44	41.7	Lachnospiraceae	41.7	2.52
Sample_T3B_bin.32	99.61	0.00	65	45.4	Bacteroidales	45.4	4.19
Sample_T3B_bin.33	87.70	0.19	191	40.8	Bacteroidales	40.8	2.81
Sample_C10A_bin.47	83.77	1.68	210	56.2	Clostridiales	56.2	2.52
Sample_T4A_bin.7	98.63	0.00	52	43.2	Clostridiales	43.2	2.62
Sample_C12B_bin.17	99.53	0.00	29	60.1	Bifidobacteriaceae	60.1	2.39
Sample_C12B_bin.14	97.65	1.34	57	46.1	Clostridiales	46.1	2.38
Sample_C1C_bin.42	87.57	1.27	18	49.3	Clostridiales	49.3	2.31
Sample_C12B_bin.12	95.74	0.69	85	59.3	Clostridiales	59.3	1.86
Sample_C12B_bin.13	98.98	4.77	98	44.9	Prevotella	44.9	3.67
Sample_C1C_bin.47	89.93	0.00	64	47	Clostridiales	47	2.50
Sample_C1C_bin.46	90.71	0.95	210	48.5	Clostridiales	48.5	2.59
Sample_C1C_bin.49	83.28	0.65	272	46.1	Clostridiales	46.1	2.48
Sample_C1C_bin.48	97.98	1.51	133	58.6	Clostridiales	58.6	2.18
Sample_C10A_bin.45	97.61	0.00	276	56.4	Clostridiales	56.4	2.48
Sample_C1A_bin.24	98.11	0.00	74	54.4	Bacteroidales	54.4	2.41
Sample_T12B_bin.8	89.39	3.02	86	62.5	Clostridiales	62.5	1.76
Sample_C1A_bin.26	88.79	1.17	93	41	Lachnospiraceae	41	2.23
Sample_C1A_bin.21	97.98	0.31	60	58.9	Clostridiales	58.9	2.02
Sample_T8A_bin.4	89.47	2.73	84	59.5	Clostridiales	59.5	1.84
Sample_C1A_bin.23	98.63	0.00	61	42.7	Clostridiales	42.7	2.88
Sample_C1A_bin.22	97.46	1.58	119	41	Clostridiales	41	3.51
Sample_C1A_bin.29	88.15	3.62	277	59	Clostridiales	59	2.07
Sample_T2A_bin.31	97.98	0.00	232	32.1	Clostridiales	32.1	2.70
Sample_T2A_bin.32	98.63	1.36	240	56.3	Clostridiales	56.3	2.43
Sample_T2A_bin.34	84.09	1.90	29	62.8	Clostridiales	62.8	1.52
Sample_T2A_bin.35	89.74	0.85	12	32.1	Bacteria	32.1	1.86
Sample_T2A_bin.36	94.77	0.37	58	44.3	Bacteroidales	44.3	3.46
Sample_T2A_bin.38	98.02	1.93	103	44.6	Lachnospiraceae	44.6	2.72
Sample_T8A_bin.12	98.85	0.00	73	48.7	Bacteroidales	48.7	3.36
Sample_C5B_bin.1	93.36	0.38	241	58.9	Clostridiales	58.9	2.30
Sample_C5B_bin.3	93.98	0.46	272	59.8	Bifidobacteriaceae	59.8	2.40
Sample_C5B_bin.2	99.32	0.67	59	54.7	Clostridiales	54.7	2.63
Sample_C5B_bin.5	98.65	0.67	29	33.3	Clostridiales	33.3	2.10
Sample_C5B_bin.7	98.77	1.12	58	46.5	Bacteroidales	46.5	4.25
Sample_C5B_bin.6	98.34	0.34	26	35.8	Clostridiales	35.8	2.70
Sample_C5B_bin.9	92.45	1.79	43	43.4	Lachnospiraceae	43.4	2.74
Sample_C5B_bin.8	99.63	0.17	78	57.7	Clostridiales	57.7	2.54
Sample_T14A_bin.41	97.58	0.16	74	26.9	Clostridiales	26.9	2.77

Sample_T18A_bin.21	96.14	1.36	297	56.2	Clostridiales	56.2	2.43
Sample_T18A_bin.20	97.31	0.00	29	53.7	Clostridiales	53.7	1.86
Sample_T18A_bin.26	85.74	2.11	326	44.4	Clostridiales	44.4	2.67
Sample_C2B_bin.58	92.06	1.54	50	58.9	Bacteroidetes	58.9	2.88
Sample_T13C_bin.2	98.51	0.60	225	45.3	Bacteroidales	45.3	3.66
Sample_C2B_bin.57	92.82	2.18	112	49.6	Clostridiales	49.6	1.93
Sample_T18A_bin.29	99.32	1.01	17	40.4	Clostridiales	40.4	2.16
Sample_T18A_bin.28	99.25	0.37	71	49.5	Bacteroidales	49.5	2.76
Sample_C2B_bin.52	96.97	0.00	28	46.3	Clostridiales	46.3	2.19
Sample_T11C_bin.26	97.27	0.00	38	51.2	Clostridiales	51.2	2.30
Sample_C2B_bin.50	85.98	0.32	67	44.6	Clostridiales	44.6	2.60
Sample_C2B_bin.51	92.51	1.02	186	60.6	Clostridiales	60.6	2.20
Sample_C1B_bin.19	97.98	0.00	193	50.1	Clostridiales	50.1	2.80
Sample_C1B_bin.18	90.58	2.79	113	42.3	Bacteroidales	42.3	3.50
Sample_T13C_bin.7	87.58	2.36	104	57.2	Clostridiales	57.2	1.91
Sample_C1B_bin.17	96.64	4.70	63	59	Clostridiales	59	2.01
Sample_C1B_bin.10	95.63	2.43	204	45	Clostridiales	45	1.74
Sample_C1B_bin.13	94.94	2.44	55	38.3	Clostridiales	38.3	2.68
Sample_C1B_bin.12	93.57	1.67	81	41.7	Bacteroidales	41.7	4.09
Sample_T13C_bin.5	92.86	0.96	260	46.5	Bacteroidetes	46.5	2.00
Sample_T5C_bin.40	98.65	0.00	66	44.2	Clostridiales	44.2	2.80
Sample_T5C_bin.41	95.78	1.38	180	45.6	Bacteroidales	45.6	3.44
Sample_T5C_bin.42	91.12	0.67	94	57.2	Clostridiales	57.2	2.42
Sample_T5C_bin.44	84.11	1.06	27	62	Clostridiales	62	1.80
Sample_T5C_bin.45	86.57	2.03	53	59.2	Clostridiales	59.2	2.01
Sample_T5C_bin.46	87.91	1.01	47	56.4	Clostridiales	56.4	2.35
Sample_T5C_bin.48	97.95	0.68	25	55.4	Bacteria	55.4	2.72
Sample_T5C_bin.49	97.55	0.17	88	57.6	Clostridiales	57.6	2.33
Sample_T7C_bin.13	99.21	2.90	85	43.5	Lachnospiraceae	43.5	2.87
Sample_T18B_bin.1	98.14	0.37	193	49.8	Bacteroidales	49.8	2.71
Sample_T4C_bin.41	91.84	0.75	53	42.1	Bacteroidales	42.1	4.22
Sample_T4C_bin.40	99.77	0.94	150	38.2	Clostridiales	38.2	3.05
Sample_T4C_bin.43	95.10	1.01	67	46	Prevotella	46	3.64
Sample_T4C_bin.42	91.94	0.67	115	60.6	Clostridiales	60.6	2.35
Sample_T4C_bin.45	98.73	0.02	99	48.3	Firmicutes	48.3	2.16
Sample_T4C_bin.44	94.63	0.00	26	59.3	Bacteroidetes	59.3	2.63
Sample_T18B_bin.6	90.89	2.17	330	48.9	Clostridiales	48.9	2.38
Sample_C13C_bin.11	96.84	0.97	115	50	Lachnospiraceae	50	2.81
Sample_C13C_bin.12	99.96	0.20	52	50.8	Enterobacteriaceae	50.8	4.56

Sample_C13C_bin.13	98.94	2.11	84	47.7	Clostridiales	47.7	3.11
Sample_C3A_bin.38	95.45	4.20	222	41.7	Bacteroidales	41.7	5.22
Sample_C13C_bin.15	94.99	0.00	33	59	Clostridiales	59	2.56
Sample_C13C_bin.16	98.27	4.39	70	42.4	Bacteria	42.4	4.85
Sample_C13C_bin.17	93.69	0.32	145	40.6	Clostridiales	40.6	2.29
Sample_C13C_bin.18	98.60	1.17	138	57.7	Clostridiales	57.7	3.18
Sample_C13C_bin.19	99.42	0.13	54	44.9	Bacteroidales	44.9	4.85
Sample_C3A_bin.37	96.59	1.81	277	56.3	Clostridiales	56.3	2.68
Sample_C3A_bin.30	92.41	3.37	33	26	Bacteria	26	1.27
Sample_T4B_bin.7	93.93	2.01	84	49.7	Clostridiales	49.7	2.04
Sample_C3A_bin.32	87.79	0.67	35	45.2	Clostridiales	45.2	1.69
Sample_C3A_bin.33	95.00	0.93	162	49.9	Bacteroidales	49.9	2.55
Sample_C14A_bin.11	98.48	0.17	289	37.4	Clostridiales	37.4	2.74
Sample_C6B_bin.17	95.97	0.00	29	41.3	Clostridiales	41.3	2.56
Sample_C6B_bin.14	98.10	3.16	40	43.9	Clostridiales	43.9	2.82
Sample_T4B_bin.6	87.38	0.00	182	36.2	Clostridiales	36.2	2.20
Sample_C6B_bin.12	90.96	3.15	496	45.2	Clostridiales	45.2	2.56
Sample_C6B_bin.13	89.26	1.41	81	28.9	Clostridiales	28.9	2.75
Sample_C6B_bin.10	97.27	0.00	16	51.4	Clostridiales	51.4	2.09
Sample_C6B_bin.11	92.41	2.25	140	26	Bacteria	26	1.28
Sample_T17A_bin.20	98.65	1.13	70	43.4	Bacteroidales	43.4	4.93
Sample_T17A_bin.21	98.73	3.16	31	42.4	Clostridiales	42.4	2.73
Sample_T17A_bin.22	98.73	0.00	23	43.8	Clostridiales	43.8	2.89
Sample_T17A_bin.23	99.68	0.16	75	48.9	Clostridiales	48.9	2.98
Sample_T17A_bin.24	84.40	0.84	352	49.9	Proteobacteria	49.9	1.68
Sample_C6B_bin.18	90.18	4.44	172	58	Clostridiales	58	2.48
Sample_C6B_bin.19	89.70	0.00	295	43.7	Clostridiales	43.7	1.75
Sample_C2A_bin.50	93.93	1.34	152	49.6	Clostridiales	49.6	2.00
Sample_C8C_bin.40	97.31	0.00	240	46.2	Clostridiales	46.2	2.93
Sample_C8C_bin.41	96.42	0.68	126	56.2	Clostridiales	56.2	2.50
Sample_C8C_bin.42	83.98	0.05	46	26.7	Bacteria	26.7	1.09
Sample_C8C_bin.43	93.93	2.01	170	49.5	Clostridiales	49.5	1.99
Sample_C8C_bin.44	93.18	1.44	55	59.6	Bacteroidetes	59.6	3.13
Sample_C8C_bin.47	99.03	0.96	27	54.3	Bacteroidetes	54.3	2.14
Sample_T11A_bin.2	100.00	0.11	19	48.4	Proteobacteria	48.4	2.71
Sample_T11A_bin.7	97.23	0.67	40	35.9	Clostridiales	35.9	2.72
Sample_C2C_bin.45	98.65	0.46	52	44.1	Clostridiales	44.1	2.93
Sample_C2C_bin.42	98.65	0.67	23	33	Clostridiales	33	2.46
Sample_T15B_bin.13	95.41	0.71	90	34.1	Bacteria	34.1	2.06

Sample_T15B_bin.10	100.00	1.20	72	47.1	Selenomonadales	47.1	2.28
Sample_T15B_bin.11	100.00	1.27	78	31.3	Firmicutes	31.3	2.42
Sample_T11A_bin.4	89.90	2.18	114	59.7	Clostridiales	59.7	2.00
Sample_T15C_bin.37	88.37	0.81	258	60.3	Actinobacteria	60.3	1.79
Sample_T7C_bin.17	80.99	0.02	347	46.4	Clostridiales	46.4	1.74
Sample_T15C_bin.36	98.87	1.92	33	58.8	Bacteroidetes	58.8	3.04
Sample_T15C_bin.35	97.98	0.34	25	44.5	Clostridiales	44.5	2.03
Sample_T15C_bin.34	92.61	3.36	36	59.9	Clostridiales	59.9	2.09
Sample_T15C_bin.33	87.46	1.49	115	40.4	Bacteroidales	40.4	3.24
Sample_T8B_bin.20	96.58	0.67	66	37.4	Clostridiales	37.4	2.87
Sample_T8B_bin.21	98.30	0.00	54	50	Bacteroidales	50	2.49
Sample_T8B_bin.22	94.91	1.90	114	49.3	Clostridiales	49.3	2.80
Sample_T13B_bin.44	99.18	0.00	29	46.5	Lactobacillales	46.5	2.17
Sample_T8B_bin.25	99.68	0.04	165	48.7	Proteobacteria	48.7	2.56
Sample_T8B_bin.26	97.55	3.41	69	46.4	Bacteroidales	46.4	4.65
Sample_T8B_bin.27	87.79	1.59	153	60	Clostridiales	60	1.90
Sample_T8B_bin.28	91.41	3.34	502	49.2	Bacteroidales	49.2	3.05
Sample_T8B_bin.29	99.32	0.57	29	43.8	Clostridiales	43.8	2.89
Sample_T15C_bin.30	97.31	0.22	37	46.4	Bacteroidales	46.4	4.30
Sample_T13B_bin.49	91.94	0.67	39	59.7	Clostridiales	59.7	2.18
Sample_T13C_bin.31	82.55	0.00	54	62.5	Clostridiales	62.5	1.66
Sample_T13C_bin.30	98.94	1.27	29	48	Clostridiales	48	3.45
Sample_T13C_bin.32	100.00	1.61	43	59.6	Actinobacteria	59.6	2.28
Sample_T13C_bin.34	97.17	3.07	27	60.4	Bifidobacteriaceae	60.4	2.37
Sample_T13C_bin.36	88.89	0.84	359	41.8	Clostridiales	41.8	2.75
Sample_T13C_bin.38	99.38	0.60	41	56	Selenomonadales	56	2.35
Sample_C6C_bin.3	96.64	0.34	119	36.7	Clostridiales	36.7	1.97
Sample_T15A_bin.26	97.98	0.34	21	44.5	Clostridiales	44.5	1.93
Sample_T15A_bin.27	98.55	2.01	28	43.8	Clostridiales	43.8	3.19
Sample_T15A_bin.24	90.93	0.00	108	62	Clostridiales	62	2.02
Sample_T15A_bin.25	95.97	0.00	46	40.9	Clostridiales	40.9	2.72
Sample_C12A_bin.22	96.59	0.84	30	58.6	Clostridiales	58.6	2.98
Sample_T15A_bin.20	82.75	3.45	78	43.3	Bacteria	43.3	3.26
Sample_T15A_bin.21	95.41	0.00	197	42	Lachnospiraceae	42	2.29
Sample_T5A_bin.12	99.38	0.60	135	45.6	Selenomonadales	45.6	1.89
Sample_T11C_bin.2	86.03	0.84	23	38.4	Clostridiales	38.4	2.50
Sample_T15A_bin.29	85.46	0.56	42	43.7	Bacteroidales	43.7	2.80
Sample_T10B_bin.28	97.31	0.00	63	53.4	Clostridiales	53.4	2.21
Sample_T10B_bin.29	98.73	0.65	97	48.6	Firmicutes	48.6	2.12

Sample_T14B_bin.28	98.10	1.27	60	31.5	Firmicutes	31.5	2.27
Sample_T10B_bin.22	99.36	0.64	141	38.4	Clostridiales	38.4	2.78
Sample_T10B_bin.23	85.22	3.23	228	60.2	Clostridiales	60.2	1.77
Sample_T14B_bin.29	92.95	2.01	102	58.3	Clostridiales	58.3	2.78
Sample_T7C_bin.18	98.55	0.39	152	40.9	Lachnospiraceae	40.9	3.79
Sample_T3A_bin.19	98.53	0.67	36	38.1	Clostridiales	38.1	1.96
Sample_T10B_bin.24	92.84	1.27	41	44.2	Clostridiales	44.2	2.89
Sample_T10B_bin.25	99.32	0.67	65	40.9	Clostridiales	40.9	2.02
Sample_T12C_bin.8	98.65	0.00	27	44.2	Clostridiales	44.2	2.71
Sample_C13A_bin.29	96.01	0.00	133	49.4	Bacteroidales	49.4	2.69
Sample_C13A_bin.28	94.22	1.75	110	42.3	Clostridiales	42.3	2.35
Sample_T12C_bin.4	95.62	0.28	78	44.4	Bacteroidales	44.4	3.52
Sample_C13A_bin.26	99.98	1.50	87	44.1	Selenomonadales	44.1	2.28
Sample_T12C_bin.6	92.91	0.85	117	42.8	Lachnospiraceae	42.8	2.99
Sample_C13A_bin.24	99.32	0.00	23	36	Clostridiales	36	2.61
Sample_T12C_bin.1	100.00	0.95	111	44.4	Clostridiales	44.4	2.72
Sample_C13A_bin.21	85.89	0.00	354	42	Lachnospiraceae	42	2.19
Sample_C13A_bin.20	91.23	4.48	763	37	Clostridiales	37	4.11
Sample_C6A_bin.28	96.64	0.00	29	41.3	Clostridiales	41.3	2.53
Sample_C6A_bin.26	87.74	1.73	196	47.1	Lachnospiraceae	47.1	2.44
Sample_C6A_bin.27	93.47	1.01	97	36	Clostridiales	36	2.79
Sample_C6A_bin.25	83.36	0.48	265	43	Lachnospiraceae	43	1.90
Sample_C6A_bin.21	94.07	1.17	432	41.1	Clostridiales	41.1	2.93
Sample_T6B_bin.7	97.98	0.00	17	38.2	Clostridiales	38.2	1.86
Sample_T6B_bin.6	90.02	2.38	238	56.5	Clostridiales	56.5	2.27
Sample_T11B_bin.36	92.51	0.48	116	41.1	Lachnospiraceae	41.1	3.46
Sample_T6B_bin.4	82.41	0.69	69	61.6	Clostridiales	61.6	1.87
Sample_T6B_bin.3	93.94	1.48	92	44.1	Clostridiales	44.1	3.20
Sample_T11B_bin.31	99.51	1.44	36	54.5	Bacteroidetes	54.5	2.36
Sample_T11B_bin.32	97.27	1.01	29	51.3	Clostridiales	51.3	2.38
Sample_T11B_bin.38	84.22	1.01	376	55.4	Clostridiales	55.4	2.01
Sample_T6B_bin.9	91.98	3.48	180	40.7	Clostridiales	40.7	3.50
Sample_C11B_bin.23	96.91	0.00	173	43.1	Clostridiales	43.1	2.48
Sample_C11A_bin.45	86.89	1.67	77	46.4	Bacteroidales	46.4	3.31
Sample_C11A_bin.43	100.00	1.27	63	31.4	Firmicutes	31.4	2.37
Sample_C11A_bin.42	98.65	0.00	19	44	Clostridiales	44	2.76
Sample_C11A_bin.41	97.30	0.84	95	33.9	Bacteria	33.9	2.24
Sample_C3C_bin.38	96.56	0.56	104	57	Clostridiales	57	2.38
Sample_C3C_bin.39	99.30	3.26	128	58.3	Clostridiales	58.3	2.97

Sample_C3C_bin.34	98.70	1.11	52	49.7	Bacteroidales	49.7	2.66
Sample_C3C_bin.30	97.98	1.01	16	44.6	Clostridiales	44.6	1.98
Sample_C3C_bin.31	96.97	1.12	259	60.1	Bacteroidetes	60.1	2.78
Sample_T15A_bin.19	80.46	3.44	386	44.2	Clostridiales	44.2	2.81
Sample_C5C_bin.1	88.14	0.31	223	46.6	Bacteroidales	46.6	3.81
Sample_T7C_bin.12	94.83	1.16	35	48.7	Clostridiales	48.7	2.73
Sample_T8C_bin.8	98.81	0.00	112	60.1	Bacteroidetes	60.1	2.62
Sample_T8C_bin.1	83.48	1.36	30	56.7	Clostridiales	56.7	2.15
Sample_T8C_bin.2	98.65	0.34	66	39.1	Clostridiales	39.1	2.38
Sample_T8C_bin.3	91.72	0.72	414	42.4	Bacteria	42.4	3.90
Sample_T8C_bin.4	91.62	0.58	199	45.6	Bacteroidales	45.6	3.99
Sample_T8C_bin.5	98.38	0.81	57	52.9	Clostridia	52.9	2.17
Sample_T8C_bin.6	97.95	1.36	30	57.7	Bacteria	57.7	3.09
Sample_T8C_bin.7	88.32	1.01	295	58.9	Clostridiales	58.9	2.00
Sample_C5C_bin.2	89.10	0.70	42	46.3	Bacteroidales	46.3	3.32
Sample_T6B_bin.24	100.00	0.18	26	60	Bifidobacteriaceae	60	2.27
Sample_C1C_bin.52	98.10	0.02	185	48.6	Firmicutes	48.6	2.03
Sample_C1C_bin.50	81.40	2.35	23	61.4	Clostridiales	61.4	1.59
Sample_T6B_bin.25	97.31	0.00	15	45.8	Clostridiales	45.8	2.14
Sample_C11C_bin.38	98.13	0.62	85	62.3	Proteobacteria	62.3	2.50
Sample_C1C_bin.54	97.48	0.13	101	42.3	Clostridiales	42.3	2.53
Sample_C1C_bin.55	96.30	0.00	242	49.7	Clostridiales	49.7	2.91
Sample_C11C_bin.34	89.54	0.56	128	44.4	Bacteroidales	44.4	3.14
Sample_C11C_bin.35	94.09	3.19	207	46.8	Lachnospiraceae	46.8	2.84
Sample_C11C_bin.36	93.25	0.09	81	40.7	Bacteroidales	40.7	2.84
Sample_C11C_bin.37	99.62	0.38	44	44	Bacteroidales	44	3.04
Sample_C11C_bin.30	87.30	3.35	209	43.6	Lachnospiraceae	43.6	2.49
Sample_C11C_bin.31	85.19	0.93	80	42.2	Bacteroidales	42.2	3.21
Sample_C11C_bin.32	97.98	0.00	40	51.7	Clostridiales	51.7	2.56
Sample_C6C_bin.59	85.70	0.25	75	46.7	Bacteroidales	46.7	2.91
Sample_C6C_bin.58	97.04	0.00	44	51.5	Clostridiales	51.5	2.09
Sample_C1A_bin.34	93.28	1.13	47	62.2	Clostridiales	62.2	1.94
Sample_C9B_bin.21	83.93	0.59	552	60.7	Deltaproteobacteria	60.7	2.93
Sample_C1A_bin.33	89.65	0.63	87	44.6	Clostridiales	44.6	2.44
Sample_C1A_bin.30	99.32	1.68	28	40.6	Clostridiales	40.6	2.18
Sample_C6C_bin.51	93.77	0.63	219	48.7	Clostridiales	48.7	2.68
Sample_C6C_bin.50	89.41	2.16	230	59.8	Bacteroidetes	59.8	2.48
Sample_C6C_bin.53	90.60	0.00	54	61.3	Clostridiales	61.3	2.18
Sample_C6C_bin.52	96.04	0.00	215	56.4	Clostridiales	56.4	2.40

Sample_C6C_bin.55	92.41	2.25	18	26	Bacteria	26	1.30
Sample_C6C_bin.54	98.10	0.02	106	48.6	Firmicutes	48.6	2.13
Sample_C6C_bin.57	93.93	1.34	46	49.6	Clostridiales	49.6	2.19
Sample_C6C_bin.56	80.77	1.93	160	46.8	Lachnospiraceae	46.8	2.35
Sample_T2A_bin.45	97.98	0.00	239	50	Clostridiales	50	2.71
Sample_T2A_bin.44	87.89	0.46	46	46	Clostridiales	46	1.95
Sample_T2A_bin.41	93.82	0.56	338	24.6	Bacteria	24.6	1.31
Sample_T2A_bin.40	86.57	0.00	72	44.3	Clostridiales	44.3	2.98
Sample_T6A_bin.43	96.09	0.95	199	48.2	Clostridiales	48.2	3.08
Sample_T6A_bin.45	99.19	0.40	43	41.8	Lachnospiraceae	41.8	2.49
Sample_T6A_bin.44	81.20	1.34	296	59.6	Clostridiales	59.6	1.91
Sample_T11C_bin.21	90.93	0.67	239	62.1	Clostridiales	62.1	1.94
Sample_C6A_bin.30	99.30	1.86	84	58.2	Clostridiales	58.2	2.89
Sample_C2B_bin.1	100.00	0.11	23	29.4	Bacteria	29.4	1.56
Sample_C8B_bin.32	98.10	1.01	86	44.3	Clostridiales	44.3	2.88
Sample_C2B_bin.4	85.04	1.51	73	60.8	Clostridiales	60.8	1.74
Sample_C2B_bin.5	94.96	1.34	277	33.4	Clostridiales	33.4	2.22
Sample_C2B_bin.6	97.98	0.34	94	44.7	Clostridiales	44.7	1.85
Sample_C2B_bin.7	94.64	1.21	39	43.7	Lachnospiraceae	43.7	2.63
Sample_C2B_bin.9	89.93	0.00	70	60.8	Clostridiales	60.8	2.26
Sample_C12A_bin.17	87.24	1.34	38	59.4	Clostridiales	59.4	2.44
Sample_C12A_bin.16	98.93	1.55	95	46.1	Lachnospiraceae	46.1	3.24
Sample_C12A_bin.15	98.83	0.82	51	41.8	Lachnospiraceae	41.8	2.45
Sample_C12A_bin.14	97.30	0.84	31	33.5	Bacteria	33.5	2.23
Sample_C12A_bin.12	99.32	0.02	69	50	Clostridiales	50	3.08
Sample_C12A_bin.10	81.28	0.00	50	46	Clostridiales	46	1.98
Sample_C12A_bin.19	97.55	1.88	129	41.9	Bacteroidales	41.9	4.47
Sample_C12A_bin.18	85.18	1.32	319	48.3	Clostridiales	48.3	2.46
Sample_C2B_bin.63	97.98	0.00	33	44.3	Clostridiales	44.3	2.64
Sample_C2B_bin.61	99.03	1.49	29	41.5	Lachnospiraceae	41.5	3.16
Sample_C2B_bin.60	89.36	2.85	76	61.6	Clostridiales	61.6	2.02
Sample_C5B_bin.11	88.66	0.63	60	41.3	Clostridiales	41.3	2.91
Sample_C5B_bin.10	94.88	2.27	244	44	Clostridiales	44	3.13
Sample_C5B_bin.17	98.94	0.63	74	47.8	Clostridiales	47.8	3.03
Sample_C5B_bin.16	100.00	1.34	75	37	Clostridiales	37	2.72
Sample_C5B_bin.18	99.32	1.01	19	40.3	Clostridiales	40.3	2.34
Sample_T16C_bin.40	96.83	1.83	304	44.6	Clostridiales	44.6	3.28
Sample_T5C_bin.34	91.00	0.77	66	42.2	Bacteroidales	42.2	3.29
Sample_T5C_bin.37	97.17	0.58	96	40.7	Bacteroidales	40.7	3.57

Sample_T5C_bin.31	90.35	1.57	200	49.6	Clostridiales	49.6	1.80
Sample_T5C_bin.30	88.04	1.34	174	60	Clostridiales	60	1.94
Sample_T5C_bin.33	99.98	1.50	30	43.5	Selenomonadales	43.5	2.48
Sample_T5C_bin.32	97.57	1.36	50	46.6	Bacteroidales	46.6	4.12
Sample_T4B_bin.9	97.27	0.50	46	51.4	Clostridiales	51.4	2.15
Sample_C14A_bin.24	96.29	3.86	244	37.3	Clostridiales	37.3	2.53
Sample_C14A_bin.25	97.58	4.95	26	46.1	Lachnospiraceae	46.1	2.89
Sample_C3C_bin.4	88.03	2.24	15	59.1	Clostridiales	59.1	1.94
Sample_C14A_bin.27	98.65	0.67	101	37.1	Clostridiales	37.1	2.85
Sample_C2A_bin.5	87.37	0.67	48	60	Clostridiales	60	2.12
Sample_C14A_bin.23	96.77	1.61	249	59.9	Actinobacteria	59.9	2.23
Sample_C6B_bin.23	87.64	1.01	79	57.9	Clostridiales	57.9	2.04
Sample_C6B_bin.22	97.67	1.27	342	48.3	Clostridiales	48.3	2.88
Sample_C6B_bin.21	82.75	0.00	118	27	Bacteria	27	2.85
Sample_T4B_bin.8	90.70	1.33	197	60.9	Clostridiales	60.9	2.09
Sample_C14A_bin.28	89.39	3.48	463	41.3	Clostridiales	41.3	3.05
Sample_C6B_bin.26	98.32	0.00	85	44.5	Clostridiales	44.5	2.88
Sample_C3C_bin.8	97.98	0.67	54	36	Clostridiales	36	2.86
Sample_C3C_bin.9	99.36	1.74	107	31.6	Firmicutes	31.6	2.25
Sample_C8C_bin.39	98.55	2.74	104	46.7	Lachnospiraceae	46.7	3.40
Sample_C6B_bin.9	98.65	0.00	51	32.2	Clostridiales	32.2	2.73
Sample_C6B_bin.4	95.30	1.01	30	51.6	Clostridiales	51.6	2.14
Sample_C8C_bin.34	97.31	0.67	14	53.1	Clostridiales	53.1	2.06
Sample_C6B_bin.6	98.73	0.02	101	48.5	Firmicutes	48.5	2.14
Sample_C11B_bin.53	95.91	4.76	77	58.6	Clostridiales	58.6	3.11
Sample_C8C_bin.31	91.94	0.00	31	60.3	Clostridiales	60.3	1.95
Sample_C6B_bin.1	98.73	1.36	22	43.7	Lachnospiraceae	43.7	2.86
Sample_C6B_bin.2	90.62	0.00	197	59.2	Bacteroidetes	59.2	2.80
Sample_C6B_bin.3	99.05	0.38	86	43.4	Bacteroidales	43.4	3.13
Sample_T2B_bin.31	89.38	0.67	39	42.6	Clostridiales	42.6	2.26
Sample_T10A_bin.45	98.65	0.34	64	39	Clostridiales	39	2.65
Sample_T2B_bin.34	98.55	0.96	150	54.5	Bacteroidetes	54.5	2.38
Sample_T2B_bin.36	97.18	0.67	37	36.2	Clostridiales	36.2	2.46
Sample_T2B_bin.39	81.05	1.33	147	26.9	Bacteria	26.9	1.08
Sample_T2B_bin.38	95.31	0.95	230	49.2	Clostridiales	49.2	2.35
Sample_T10C_bin.16	95.90	1.37	77	44.3	Bacteroidales	44.3	3.78
Sample_T10C_bin.17	99.32	1.80	82	44.8	Prevotella	44.8	3.83
Sample_T10C_bin.10	94.51	0.84	81	48.8	Clostridiales	48.8	2.53
Sample_T10C_bin.11	92.35	1.32	255	50	Bacteroidales	50	2.36

Sample_T10C_bin.12	98.14	0.38	192	42.2	Bacteroidales	42.2	4.72
Sample_T10A_bin.35	98.07	0.58	65	45.1	Bacteroidales	45.1	4.24
Sample_T10A_bin.36	88.59	3.69	103	61.8	Clostridiales	61.8	1.92
Sample_T10A_bin.37	98.38	0.00	175	43.4	Bacteria	43.4	4.04
Sample_T10A_bin.30	97.85	0.68	49	57.3	Clostridiales	57.3	2.76
Sample_T10A_bin.31	88.59	0.00	178	60.9	Clostridiales	60.9	2.28
Sample_T10A_bin.33	96.25	0.34	231	61.2	Clostridiales	61.2	2.69
Sample_C12C_bin.55	84.65	1.72	154	60.5	Bacteria	60.5	2.58
Sample_T10A_bin.38	98.16	0.67	157	44.5	Clostridiales	44.5	2.55
Sample_T10A_bin.39	87.15	1.58	220	42.2	Clostridiales	42.2	2.23
Sample_C12C_bin.54	94.57	1.14	182	57.1	Clostridiales	57.1	2.24
Sample_T8B_bin.50	97.34	0.97	45	41.7	Lachnospiraceae	41.7	2.64
Sample_T5A_bin.17	97.18	0.00	55	43.3	Bacteroidales	43.3	4.65
Sample_T8A_bin.43	91.59	1.53	247	60.7	Clostridiales	60.7	2.28
Sample_T8A_bin.42	98.65	0.00	168	37.8	Clostridiales	37.8	1.98
Sample_T8A_bin.41	96.05	0.71	23	45.1	Bacteroidales	45.1	4.95
Sample_T8A_bin.40	98.10	0.63	359	44.6	Clostridiales	44.6	2.69
Sample_C6A_bin.17	97.31	1.34	16	51.8	Clostridiales	51.8	2.18
Sample_T14B_bin.9	95.63	2.34	78	40.8	Lachnospiraceae	40.8	2.57
Sample_T15A_bin.33	87.24	0.00	43	36.6	Clostridiales	36.6	1.72
Sample_T15A_bin.32	90.92	1.93	14	59.6	Clostridiales	59.6	2.07
Sample_C1A_bin.6	92.69	4.49	27	26.1	Bacteria	26.1	1.27
Sample_T4B_bin.14	88.64	0.81	21	45.9	Clostridia	45.9	1.67
Sample_T4B_bin.17	98.55	0.00	48	41.5	Lachnospiraceae	41.5	2.76
Sample_T8C_bin.28	97.95	0.00	128	58.3	Clostridiales	58.3	3.22
Sample_T4B_bin.11	98.32	0.00	100	36.3	Clostridiales	36.3	2.59
Sample_T4B_bin.10	99.30	1.17	66	57.9	Clostridiales	57.9	3.07
Sample_C8B_bin.5	98.79	1.92	36	58	Bacteroidetes	58	3.27
Sample_T4B_bin.12	95.97	0.00	73	38.1	Clostridiales	38.1	1.84
Sample_T10B_bin.39	92.94	0.00	138	43.1	Lachnospiraceae	43.1	2.21
Sample_T10B_bin.38	80.25	0.63	542	41.4	Clostridiales	41.4	2.70
Sample_T10B_bin.30	99.32	0.00	37	44	Clostridiales	44	2.80
Sample_T10B_bin.33	98.06	0.63	59	41.6	Lachnospiraceae	41.6	2.73
Sample_T8C_bin.25	99.32	1.01	35	40.8	Clostridiales	40.8	2.17
Sample_T10B_bin.37	87.08	1.04	137	44.3	Lachnospiraceae	44.3	2.00
Sample_T8C_bin.24	90.91	1.01	72	49.6	Clostridiales	49.6	2.39
Sample_T8C_bin.22	97.27	0.00	55	43.6	Lachnospiraceae	43.6	2.84
Sample_T14A_bin.40	98.65	1.34	105	36.4	Clostridiales	36.4	1.96
Sample_T8C_bin.21	89.27	1.97	137	51.2	Enterobacteriaceae	51.2	3.79

Sample_T11B_bin.29	97.26	0.00	66	42.3	Clostridiales	42.3	2.89
Sample_T11B_bin.22	99.24	1.13	209	43.4	Bacteroidales	43.4	3.06
Sample_T11B_bin.21	93.95	0.00	98	41.4	Clostridiales	41.4	3.04
Sample_T11B_bin.20	93.87	1.21	90	60.7	Clostridiales	60.7	2.29
Sample_C6B_bin.56	87.91	0.67	97	61.6	Clostridiales	61.6	1.94
Sample_T11B_bin.24	93.74	1.68	85	59.5	Bacteroidetes	59.5	2.97
Sample_T14A_bin.43	90.44	2.53	56	24.9	Bacteria	24.9	1.29
Sample_T12B_bin.9	96.64	2.10	230	56	Clostridiales	56	2.80
Sample_T7A_bin.49	99.51	2.19	30	40.9	Lachnospiraceae	40.9	2.61
Sample_T7A_bin.44	95.30	0.00	81	41.2	Clostridiales	41.2	2.60
Sample_T12B_bin.4	93.83	0.00	283	42.7	Clostridiales	42.7	2.70
Sample_T7A_bin.46	93.75	0.96	24	54.8	Bacteroidetes	54.8	1.95
Sample_T12B_bin.1	93.65	1.87	55	45.3	Lachnospiraceae	45.3	2.26
Sample_T11B_bin.16	90.18	3.32	437	41.4	Clostridiales	41.4	2.78
Sample_T7A_bin.42	96.64	0.00	26	42.2	Clostridiales	42.2	2.66
Sample_T7A_bin.43	99.32	0.34	30	36.4	Clostridiales	36.4	2.20
Sample_T13A_bin.15	91.53	0.00	100	42.2	Bacteroidales	42.2	3.99
Sample_T14A_bin.45	94.72	2.03	339	43.3	Clostridiales	43.3	3.04
Sample_C5A_bin.11	98.65	0.00	224	36.3	Clostridiales	36.3	2.51
Sample_C5A_bin.12	93.80	3.36	69	37.4	Clostridiales	37.4	2.23
Sample_C5A_bin.13	96.85	0.00	162	29	Bacteria	29	2.82
Sample_C5A_bin.15	86.23	3.29	201	42.2	Bacteroidales	42.2	3.77
Sample_C5A_bin.16	93.53	1.21	35	43.6	Lachnospiraceae	43.6	2.55
Sample_C5A_bin.17	95.91	0.34	55	60.6	Clostridiales	60.6	2.34
Sample_T14A_bin.30	92.57	2.01	118	59.8	Clostridiales	59.8	1.80
Sample_T14B_bin.21	98.73	0.02	100	48.8	Firmicutes	48.8	2.12
Sample_T14A_bin.44	99.98	1.70	105	43.4	Selenomonadales	43.4	2.59
Sample_T14A_bin.32	99.51	2.90	85	46.1	Lachnospiraceae	46.1	3.17
Sample_T14A_bin.34	99.32	0.67	37	40.1	Clostridiales	40.1	2.39
Sample_C6C_bin.31	92.46	1.93	101	44.2	Lachnospiraceae	44.2	2.43
Sample_T14A_bin.47	91.80	1.27	187	42.2	Lachnospiraceae	42.2	2.22
Sample_C5A_bin.35	97.59	0.96	75	54.6	Bacteroidetes	54.6	2.04
Sample_T15C_bin.5	90.93	0.00	16	62.2	Clostridiales	62.2	1.94
Sample_T15C_bin.7	97.98	0.04	95	34.1	Clostridiales	34.1	2.33
Sample_T15C_bin.6	90.21	1.48	134	41.8	Lachnospiraceae	41.8	3.02
Sample_T15C_bin.2	93.75	2.47	211	50.1	Lachnospiraceae	50.1	2.76
Sample_C12B_bin.30	90.12	1.34	22	42.1	Clostridiales	42.1	1.66
Sample_C12B_bin.31	100.00	0.92	24	56.1	Bifidobacteriaceae	56.1	2.16
Sample_C12B_bin.32	97.27	0.56	89	51.5	Clostridiales	51.5	2.20

Sample_C12B_bin.33	94.67	0.58	79	46.1	Bacteroidales	46.1	2.98
Sample_T15C_bin.9	98.63	0.00	89	42.5	Clostridiales	42.5	3.01
Sample_C11C_bin.23	87.04	0.14	326	60.6	Deltaproteobacteria	60.6	3.20
Sample_C11C_bin.22	85.70	0.08	599	44.5	Clostridiales	44.5	2.50
Sample_C11C_bin.21	96.05	0.19	279	48.4	Bacteroidales	48.4	3.85
Sample_C6C_bin.49	97.98	0.00	84	47.3	Clostridiales	47.3	2.57
Sample_C11C_bin.27	99.51	3.37	74	54.3	Bacteroidetes	54.3	2.27
Sample_C11C_bin.26	91.70	1.36	346	55.9	Bacteria	55.9	2.15
Sample_C11C_bin.25	97.27	0.68	71	58.9	Clostridiales	58.9	3.04
Sample_C6C_bin.40	99.62	0.00	80	43.2	Bacteroidales	43.2	3.23
Sample_C11C_bin.28	92.03	2.89	90	58.5	Clostridiales	58.5	2.16
Sample_C6C_bin.47	94.52	0.00	115	44.4	Clostridiales	44.4	2.58
Sample_C6C_bin.44	94.63	1.34	87	58.4	Clostridiales	58.4	2.50
Sample_T2A_bin.56	99.98	1.50	24	43.4	Selenomonadales	43.4	2.56
Sample_T2A_bin.57	97.30	0.84	55	33.4	Bacteria	33.4	2.18
Sample_T2A_bin.54	98.65	0.67	181	33.3	Clostridiales	33.3	2.24
Sample_T2A_bin.52	99.32	1.01	34	40.8	Clostridiales	40.8	2.01
Sample_T2A_bin.53	96.64	0.00	16	50.1	Clostridiales	50.1	2.44
Sample_T2A_bin.50	95.60	2.00	158	46	Clostridiales	46	2.53
Sample_T2A_bin.51	87.65	3.14	18	47.6	Lachnospiraceae	47.6	2.75
Sample_T2A_bin.58	83.61	0.63	90	45.7	Clostridiales	45.7	2.68
Sample_C11A_bin.32	95.90	1.50	471	59	Clostridiales	59	2.01
Sample_C11A_bin.30	99.21	1.45	85	44.5	Lachnospiraceae	44.5	2.87
Sample_C11A_bin.31	99.19	2.42	87	59.7	Actinobacteria	59.7	2.30
Sample_C11A_bin.36	98.87	2.25	97	29.3	Bacteria	29.3	2.60
Sample_C11A_bin.34	83.39	4.17	154	42	Bacteroidales	42	3.71
Sample_C11A_bin.35	98.43	0.00	91	38.2	Clostridiales	38.2	1.82
Sample_C11A_bin.38	97.63	1.47	172	45.6	Prevotella	45.6	3.38
Sample_C11A_bin.39	99.40	1.20	47	47.4	Selenomonadales	47.4	2.09
Sample_C1A_bin.9	98.60	1.17	113	58	Clostridiales	58	3.18
Sample_C6B_bin.16	95.00	0.68	114	44.7	Bacteroidales	44.7	3.60

Table S5. Genomic information of 389 species-level genome bins (SGBs)

MAG ID	SGBs	Genome information							CheckM estimation			Taxonomic assigment			
	Rename_ID	Number of contigs	Genome size (Mbp)	N50 length (bp)	N90 length (bp)	Max length (bp)	GC content (%)	Sequencing depth (X)	Completeness (%)	Contamination (%)	Checkm lineage	Cultured species (Y/N)	New_species (Y/N)	Phylum	Species
Sample_T17C.9	T17C.M009	45	3.77	116779	62315	371500	44.16	411.46	99.16	0.28	Bacteroidales	N	N	Bacteroidetes	Bacteroides plebeius
Sample_C13C.15	C13C.M015	33	2.56	112579	55823	269829	59.10	381.30	94.99	0.00	Clostridiales	N	N	Firmicutes	Faecalibacterium prausnitzii
Sample_C13B.16	C13B.M016	289	1.75	8140	2948	39305	45.03	8.12	86.79	3.51	Lactobacillales	N	N	Firmicutes	Weissella confusa
Sample_T17B.1	T17B.M001	36	2.10	101634	26149	261351	38.66	12.27	99.60	0.00	Selenomonadales	N	N	Firmicutes	Veillonella parvula
Sample_T7A.43	T7A.M043	26	2.20	167522	38870	323919	36.43	108.06	99.32	0.34	Clostridiales	Y	N	Firmicutes	uncultured Ruminococcus sp.
Sample_T13A.19	T13A.M019	37	2.76	220081	46507	452427	42.95	32.08	98.63	0.00	Clostridiales	Y	N	Firmicutes	uncultured Ruminococcus sp.
Sample_T16B.17	T16B.M017	81	2.87	55659	16738	234096	44.58	31.78	100.00	0.00	Clostridiales	Y	N	Firmicutes	uncultured Ruminococcus sp.
Sample_C14C.35	C14C.M035	124	3.01	59417	11600	189666	47.02	29.60	97.98	0.00	Clostridiales	Y	N	Firmicutes	uncultured Ruminococcus sp.
Sample_C12A.12	C12A.M012	50	3.08	167311	28601	516307	50.07	18.28	99.32	0.02	Clostridiales	Y	N	Firmicutes	uncultured Ruminococcus sp.
Sample_C14A.27	C14A.M027	101	2.85	68237	13140	249870	37.19	18.27	98.65	0.67	Clostridiales	Y	N	Firmicutes	uncultured Ruminococcus sp.
Sample_T3B.31	T3B.M031	59	2.52	70899	21337	227234	41.75	17.24	99.41	0.00	Lachnospiraceae	Y	N	Firmicutes	uncultured Ruminococcus sp.
Sample_C10A.24	C10A.M024	187	2.74	23643	6628	91830	44.77	10.31	96.97	0.89	Clostridiales	Y	N	Firmicutes	uncultured Ruminococcus sp.
Sample_T12A.51	T12A.M051	180	2.35	18435	6500	83702	44.27	9.92	92.95	0.76	Clostridiales	Y	N	Firmicutes	uncultured Ruminococcus sp.
Sample_C10A.69	C10A.M069	29	2.65	132030	59434	239729	40.90	25.11	99.51	0.98	Lachnospiraceae	Y	N	Firmicutes	uncultured Roseburia sp.
Sample_T10A.33	T10A.M033	178	2.69	22678	6585	177700	61.28	10.12	96.25	0.34	Clostridiales	Y	N	Firmicutes	uncultured Oscillibacter sp.
Sample_T7A.4	T7A.M004	81	3.59	95243	21431	227040	41.04	20.41	98.10	1.58	Clostridiales	Y	N	Firmicutes	uncultured Lachnospira sp.
Sample_C13A.30	C13A.M030	63	2.21	53956	19445	179456	61.12	13.40	97.98	2.01	Clostridiales	Y	N	Firmicutes	uncultured Flavonifractor sp.
Sample_T2B.11	T2B.M011	39	2.51	85986	41896	341464	33.18	14.60	97.98	0.00	Clostridiales	Y	N	Firmicutes	uncultured Eubacterium sp.
Sample_T5B.31	T5B.M031	165	3.28	27612	8869	125184	37.69	11.06	98.65	2.80	Clostridiales	Y	N	Firmicutes	uncultured Eubacterium sp.
Sample_T4C.40	T4C.M040	165	3.05	27382	8606	96118	38.29	10.72	99.77	0.94	Clostridiales	Y	N	Firmicutes	uncultured Eubacterium sp.
Sample_C12B.24	C12B.M024	17	2.66	242945	74320	462388	41.37	53.32	99.32	0.00	Clostridiales	Y	N	Firmicutes	uncultured Coprococcus sp.
Sample_T6B.21	T6B.M021	75	2.62	72973	18294	153722	29.86	232.59	99.05	0.00	Bacteria	Y	N	Firmicutes	uncultured Clostridium sp.
Sample_C12A.16	C12A.M016	95	3.24	50214	17562	209903	46.12	19.49	98.93	1.55	Lachnospiraceae	Y	N	Firmicutes	uncultured Clostridium sp.
Sample_C10A.44	C10A.M044	64	2.99	65422	25064	149310	47.21	16.76	94.20	0.97	Lachnospiraceae	Y	N	Firmicutes	uncultured Clostridium sp.
Sample_C1C.21	C1C.M021	47	2.73	77873	32084	266997	48.86	16.68	96.93	0.19	Lachnospiraceae	Y	N	Firmicutes	uncultured Clostridium sp.
Sample_T11C.9	T11C.M009	50	2.91	113272	36733	359814	38.02	16.06	97.98	1.01	Clostridiales	Y	N	Firmicutes	uncultured Clostridium sp.
Sample_C4B.12	C4B.M012	92	3.28	52893	18975	194584	38.79	13.63	99.32	0.34	Clostridiales	Y	Y	Firmicutes	uncultured Clostridium sp.
Sample_T14A.41	T14A.M041	105	2.77	40673	12430	128516	26.94	13.10	97.58	0.16	Clostridiales	Y	N	Firmicutes	uncultured Clostridium sp.
Sample_T3C.30	T3C.M030	112	2.90	36822	15801	134651	49.21	11.12	98.73	0.89	Clostridiales	Y	N	Firmicutes	uncultured Clostridium sp.
Sample_C13B.14	C13B.M014	172	2.96	30131	7858	83957	28.90	10.90	98.11	0.00	Bacteria	Y	N	Firmicutes	uncultured Clostridium sp.
Sample_C1C.5	C1C.M005	101	2.75	38656	14135	181165	50.51	10.78	96.51	0.11	Clostridiales	Y	N	Firmicutes	uncultured Clostridium sp.
Sample_T4C.28	T4C.M028	589	5.38	11977	4508	60010	50.16	8.09	89.86	1.90	Clostridiales	Y	N	Firmicutes	uncultured Clostridium sp.
Sample_C3A.5	C3A.M005	368	2.25	7041	3231	27638	42.97	7.31	80.97	1.68	Clostridiales	Y	Y	Firmicutes	uncultured Clostridium sp.

Sample_C6B.13	C6B.M013	496	2.75	6362	2789	55045	28.94	7.30	89.26	1.41	Clostridiales	Y	N	Firmicutes	uncultured Clostridium sp.
Sample_C11B.47	C11B.M047	713	4.61	8154	3039	46847	52.17	8.39	88.97	1.46	Enterobacteriaceae	Y	N	Proteobacteria	uncultured Citrobacter sp.
Sample_C5B.2	C5B.M002	63	2.63	69241	19543	262638	54.74	25.24	99.32	0.67	Clostridiales	Y	N	Firmicutes	uncultured Butyricicoccus sp.
Sample_T17C.7	T17C.M007	34	2.70	121062	42815	293346	42.44	33.86	99.36	0.00	Clostridiales	Y	Y	Firmicutes	uncultured Blautia sp.
Sample_T6B.40	T6B.M040	69	2.62	52201	21199	150384	44.84	15.67	92.40	4.11	Clostridiales	Y	N	Firmicutes	uncultured Blautia sp.
Sample_T11A.23	T11A.M023	50	2.89	99162	37087	176448	44.73	15.17	99.36	0.32	Clostridiales	Y	N	Firmicutes	uncultured Blautia sp.
Sample_T2A.58	T2A.M058	90	2.68	42563	15196	116379	45.76	12.22	83.61	0.63	Clostridiales	Y	N	Firmicutes	uncultured Blautia sp.
Sample_C9A.35	C9A.M035	259	3.04	14792	6175	41166	43.43	8.73	84.28	1.27	Clostridiales	Y	N	Firmicutes	uncultured Blautia sp.
Sample_C11C.18	C11C.M018	110	4.73	65220	21580	296138	42.20	14.16	91.04	2.18	Bacteroides	Y	N	Bacteroidetes	uncultured Bacteroides sp.
Sample_C13A.9	C13A.M009	67	2.59	101006	17380	195167	40.33	27.69	96.83	0.95	Clostridiales	N	N	Firmicutes	Tyzzerella nexilis
Sample_C5A.36	C5A.M036	479	2.94	7419	3161	24506	38.58	7.25	86.79	2.58	Bacteroidales	N	N	Bacteroidetes	Tannerella sp. AF04-6
Sample_C11A.16	C11A.M016	67	2.37	52598	19494	297847	62.54	28.32	98.13	0.62	Proteobacteria	N	N	Proteobacteria	Sutterella wadsworthensis
Sample_C3B.28	C3B.M028	45	2.84	100335	28051	408555	55.60	17.40	100.00	0.00	Proteobacteria	N	N	Proteobacteria	Sutterella wadsworthensis
Sample_T13C.16	T13C.M016	51	1.80	62301	15322	254451	47.07	12.39	98.02	1.88	Proteobacteria	N	N	Proteobacteria	Sutterella sp. CAG:521
Sample_T4C.37	T4C.M037	37	2.48	101349	32337	224581	57.17	26.88	98.12	0.00	Proteobacteria	N	N	Proteobacteria	Sutterella sp. AM18-8-1
Sample_C10A.31	C10A.M031	24	2.19	178214	45599	424273	55.63	29.31	98.75	0.63	Proteobacteria	N	N	Proteobacteria	Sutterella sp. AM11-39
Sample_C7C.7	C7C.M007	34	2.33	132096	29758	531694	54.31	14.82	98.75	0.63	Proteobacteria	N	N	Proteobacteria	Sutterella sp. AM11-39
Sample_T17C.8	T17C.M008	84	2.41	57601	13135	195610	62.01	14.57	96.89	0.62	Proteobacteria	N	N	Proteobacteria	Sutterella megalosphaeroides
Sample_T4A.11	T4A.M011	75	2.36	47834	13763	132133	58.67	41.55	96.09	0.34	Clostridiales	N	N	Firmicutes	Subdoligranulum variabile
Sample_C7A.17	C7A.M017	94	2.51	37201	14835	107565	59.09	21.15	95.59	0.79	Clostridiales	N	N	Firmicutes	Subdoligranulum sp. 60_17
Sample_C3C.6	C3C.M006	68	2.23	53423	16938	213423	58.62	18.51	94.47	0.00	Clostridiales	N	Y	Firmicutes	Subdoligranulum sp. 60_17
Sample_T3A.12	T3A.M012	79	1.96	39875	12081	139498	39.89	11.33	98.74	2.07	Streptococcus	N	N	Firmicutes	Streptococcus salivarius
Sample_C13C.6	C13C.M006	25	2.08	156572	28656	400021	37.29	29.62	100.00	0.00	Lactobacillales	N	N	Firmicutes	Streptococcus pasteurianus
Sample_T15A.38	T15A.M038	60	2.03	52838	20118	190562	55.14	12.00	98.83	0.16	Firmicutes	N	N	Firmicutes	Schwartzia succinivorans
Sample_T8C.28	T8C.M028	128	3.22	43205	13738	144565	58.32	15.39	97.95	0.00	Clostridiales	N	N	Firmicutes	Ruthenibacterium lactatiformans
Sample_T18A.13	T18A.M013	37	2.31	161290	39265	245527	49.40	73.85	97.98	0.00	Clostridiales	N	N	Firmicutes	Ruminococcus sp. OM06-36AC
Sample_T6C.36	T6C.M036	39	2.64	116251	35943	331684	51.46	22.95	98.65	0.04	Clostridiales	N	N	Firmicutes	Ruminococcus sp. OM06-36AC
Sample_C8C.18	C8C.M018	138	2.79	35735	7913	141347	45.92	9.37	94.94	1.75	Lachnospiraceae	N	N	Firmicutes	Ruminococcus sp. DSM 100440
Sample_T2C.29	T2C.M029	33	2.36	152272	33025	277649	49.96	12.23	96.64	0.00	Clostridiales	N	N	Firmicutes	Ruminococcus sp. CAG:579
Sample_C2C.8	C2C.M008	319	2.01	7674	3265	31292	44.37	7.29	85.06	0.84	Clostridiales	N	N	Firmicutes	Ruminococcus sp. CAG:563
Sample_C4C.48	C4C.M048	63	2.71	67630	21158	184824	41.07	17.25	97.36	0.00	Lachnospiraceae	N	N	Firmicutes	Ruminococcus sp. CAG:55
Sample_T12A.31	T12A.M031	25	2.25	141199	42529	276095	44.61	45.04	97.09	0.00	Clostridiales	N	N	Firmicutes	Ruminococcus sp. CAG:488
Sample_C4A.17	C4A.M017	12	2.12	257602	84265	368588	45.64	20.13	97.98	0.00	Clostridiales	N	N	Firmicutes	Ruminococcus sp. CAG:403
Sample_T17B.18	T17B.M018	249	3.94	25248	7372	138219	40.91	124.03	96.04	0.63	Clostridiales	N	N	Firmicutes	Ruminococcus sp. AF37-6AT
Sample_T4A.26	T4A.M026	95	2.58	89624	9848	223006	45.93	21.70	98.65	0.00	Clostridiales	Y	N	Firmicutes	Ruminococcus sp.
Sample_T12A.9	T12A.M009	215	2.13	13851	4530	62659	39.91	18.62	93.95	0.84	Clostridiales	Y	Y	Firmicutes	Ruminococcus sp.
Sample_C10B.23	C10B.M023	100	2.43	39510	11797	108380	42.99	17.11	98.24	0.00	Lachnospiraceae	N	N	Firmicutes	Ruminococcus lactaris
Sample_C1A.16	C1A.M016	199	2.82	24762	7110	105248	50.07	20.47	97.98	0.00	Clostridiales	N	N	Firmicutes	Ruminococcus callidus
Sample_C10B.18	C10B.M018	32	2.20	176404	40321	316548	40.78	62.61	99.32	0.67	Clostridiales	N	N	Firmicutes	Ruminococcus bromii

Sample_T15C.29	T15C.M029	42	2.33	141324	27922	417654	40.36	34.58	99.32	0.00	Clostridiales	N	N	Firmicutes	Ruminococcus bromii
Sample_C12A.29	C12A.M029	23	1.93	136649	65005	355211	42.40	127.72	95.30	0.00	Clostridiales	N	Y	Firmicutes	Ruminococcaceae bacterium
Sample_C12C.35	C12C.M035	27	2.27	182355	49949	340424	51.71	37.40	97.27	0.00	Clostridiales	N	N	Firmicutes	Ruminococcaceae bacterium
Sample_C13A.16	C13A.M016	14	1.91	207808	77316	489148	44.34	25.24	97.98	0.34	Clostridiales	N	N	Firmicutes	Ruminococcaceae bacterium
Sample_T5C.2	T5C.M002	15	2.19	277424	67732	534224	51.27	23.79	97.27	0.00	Clostridiales	N	N	Firmicutes	Ruminococcaceae bacterium
Sample_T11A.47	T11A.M047	45	2.60	172794	49441	468770	53.12	21.19	98.65	0.00	Clostridiales	N	N	Firmicutes	Ruminococcaceae bacterium
Sample_C10A.45	C10A.M045	276	2.48	13041	4130	35358	56.47	20.03	97.61	0.00	Clostridiales	N	N	Firmicutes	Ruminococcaceae bacterium
Sample_T12A.57	T12A.M057	68	2.24	62187	15704	301645	45.93	16.52	94.63	2.01	Clostridiales	N	N	Firmicutes	Ruminococcaceae bacterium
Sample_C12C.59	C12C.M059	55	2.02	60856	16094	187032	56.82	16.35	88.36	0.00	Clostridiales	N	Y	Firmicutes	Ruminococcaceae bacterium
Sample_C12A.27	C12A.M027	117	2.35	34404	8844	111372	46.29	13.72	95.07	1.34	Clostridiales	N	N	Firmicutes	Ruminococcaceae bacterium
Sample_C2A.64	C2A.M064	120	2.30	26695	9338	100460	51.81	11.68	97.76	1.36	Clostridiales	N	N	Firmicutes	Ruminococcaceae bacterium
Sample_T16C.24	T16C.M024	142	2.19	22094	7207	102418	38.97	10.27	99.32	0.34	Clostridiales	N	N	Firmicutes	Ruminococcaceae bacterium
Sample_C2A.11	C2A.M011	216	2.03	12413	4816	44837	53.31	8.98	87.05	0.67	Clostridiales	N	N	Firmicutes	Ruminococcaceae bacterium
Sample_T3B.29	T3B.M029	465	2.65	6748	2936	32701	60.62	7.08	84.33	0.67	Clostridiales	N	N	Firmicutes	Ruminococcaceae bacterium
Sample_T6B.35	T6B.M035	384	2.68	8453	3547	27033	42.85	7.89	84.49	3.16	Lachnospiraceae	N	N	Firmicutes	Roseburia sp. OM02-15
Sample_T15A.12	T15A.M012	79	3.50	76830	22249	215668	41.75	46.25	99.03	2.13	Lachnospiraceae	N	N	Firmicutes	Roseburia sp. OF03-24
Sample_T16C.31	T16C.M031	49	2.74	109168	28805	287542	40.77	24.40	98.43	1.34	Clostridiales	N	N	Firmicutes	Roseburia sp. CAG:303
Sample_C5A.5	C5A.M005	41	2.56	96032	29497	220505	44.95	24.01	94.92	0.00	Lachnospiraceae	N	N	Firmicutes	Roseburia sp. CAG:197
Sample_T15A.15	T15A.M015	41	2.38	92026	31369	165523	45.25	13.65	96.61	1.69	Lachnospiraceae	N	N	Firmicutes	Roseburia sp. AM16-25
Sample_T8B.32	T8B.M032	383	2.21	6809	2938	29806	40.11	7.04	81.99	2.48	Lachnospiraceae	N	N	Firmicutes	Roseburia sp. 1XD42-69
Sample_T13B.55	T13B.M055	289	2.38	10041	4408	41270	48.44	8.24	83.27	0.48	Lachnospiraceae	Y	Y	Firmicutes	Roseburia sp.
Sample_T7C.18	T7C.M018	152	3.79	40424	10391	265450	40.96	13.60	98.55	0.39	Lachnospiraceae	N	N	Firmicutes	Roseburia inulinivorans
Sample_T8A.37	T8A.M037	52	3.44	101680	36706	235638	42.81	34.79	99.03	0.36	Lachnospiraceae	N	N	Firmicutes	Roseburia intestinalis
Sample_C14B.22	C14B.M022	40	2.99	118368	37887	438640	49.59	34.72	99.44	0.24	Lachnospiraceae	N	N	Firmicutes	Roseburia hominis
Sample_C12B.22	C12B.M022	50	3.10	127928	28378	357364	43.35	33.03	99.21	0.97	Lachnospiraceae	N	N	Firmicutes	Roseburia faecis
Sample_C6B.21	C6B.M021	116	2.85	36022	12578	183190	27.05	25.57	82.75	0.00	Bacteria	N	N	Firmicutes	Romboutsia ilealis
Sample_T18A.16	T18A.M016	90	2.32	43182	11436	276326	48.94	21.01	100.00	0.00	Proteobacteria	N	N	Proteobacteria	Proteobacteria bacterium CAG:139
Sample_T12A.11	T12A.M011	53	2.60	86404	20528	201953	48.24	13.68	95.94	0.74	Bacteroidales	N	Y	Bacteroidetes	Prevotellaceae bacterium
Sample_C13B.31	C13B.M031	96	2.76	45235	13450	93157	49.45	20.96	99.25	0.12	Bacteroidales	N	N	Bacteroidetes	Prevotella stercorea
Sample_T4A.6	T4A.M006	100	3.33	55284	19133	243442	46.40	72.55	93.07	0.34	Prevotella	N	N	Bacteroidetes	Prevotella sp. TF12-30
Sample_C11B.52	C11B.M052	170	2.67	23211	7424	67133	47.60	15.67	96.34	0.74	Bacteroidales	N	N	Bacteroidetes	Prevotella sp. CAG:891
Sample_T7A.34	T7A.M034	76	2.86	60381	20206	170871	50.46	91.07	99.25	0.00	Bacteroidales	N	N	Bacteroidetes	Prevotella sp. CAG:520
Sample_T2B.23	T2B.M023	81	3.51	66098	22916	266137	45.60	34.13	99.25	0.23	Bacteroidales	N	N	Bacteroidetes	Prevotella sp. CAG:255
Sample_C1B.4	C1B.M004	73	2.47	54163	18714	194302	54.13	69.79	98.86	0.00	Bacteroidales	N	N	Bacteroidetes	Prevotella sp. CAG:1031
Sample_T15B.1	T15B.M001	90	2.52	40109	13674	145438	47.18	116.20	81.89	1.72	Bacteria	Y	Y	Bacteroidetes	Prevotella sp.
Sample_C6A.32	C6A.M032	62	3.44	98662	26260	232030	50.90	66.26	99.32	1.13	Prevotella	Y	Y	Bacteroidetes	Prevotella sp.
Sample_C11B.20	C11B.M020	127	2.95	36760	10953	165045	45.93	134.97	97.12	0.84	Prevotella	N	N	Bacteroidetes	Prevotella copri
Sample_C7B.12	C7B.M012	46	2.52	94240	25143	278358	49.90	31.72	98.30	0.00	Bacteroidales	N	N	Bacteroidetes	Porphyromonadaceae bacterium
Sample_T17C.17	T17C.M017	21	1.89	148145	66279	263476	42.26	18.28	100.00	1.10	Selenomonadales	N	N	Firmicutes	Phascolarctobacterium succinatutens

Sample_T8B.36	T8B.M036	39	2.37	116219	36745	249725	46.70	15.13	99.40	0.80	Selenomonadales	N	N	Firmicutes	Phascolarctobacterium succinatutens
Sample_T5A.12	T5A.M012	24	1.89	134885	53443	265958	45.61	26.02	99.38	0.60	Selenomonadales	N	N	Firmicutes	Phascolarctobacterium sp. CAG:266
Sample_C13B.9	C13B.M009	101	2.43	47797	15191	255470	43.90	13.78	99.80	1.35	Selenomonadales	N	N	Firmicutes	Phascolarctobacterium faecium
Sample_T5A.21	T5A.M021	41	3.54	116502	52599	244031	48.66	56.02	99.13	0.00	Bacteroidales	N	N	Bacteroidetes	Paraprevotella xylaniphila
Sample_T2C.19	T2C.M019	71	4.40	131692	39063	403550	48.23	23.34	99.25	0.19	Bacteroidales	N	N	Bacteroidetes	Paraprevotella clara
Sample_T3B.32	T3B.M032	44	4.19	147520	50621	280618	45.45	34.90	99.61	0.00	Bacteroidales	N	N	Bacteroidetes	Parabacteroides merdae
Sample_T5A.7	T5A.M007	46	4.15	118853	54386	333251	45.31	31.77	99.23	0.00	Bacteroidales	N	N	Bacteroidetes	Parabacteroides johnsonii CAG:246
Sample_C5A.66	C5A.M066	60	4.72	163417	41458	367268	45.09	14.41	99.42	0.00	Bacteroidales	N	N	Bacteroidetes	Parabacteroides distasonis
Sample_T5C.49	T5C.M049	88	2.33	51061	14983	116295	57.63	18.32	97.55	0.17	Clostridiales	N	N	Firmicutes	Oscillibacter sp. 57_20
Sample_C12C.53	C12C.M053	73	2.30	51713	17859	169143	57.50	15.01	91.96	3.69	Clostridiales	N	Y	Firmicutes	Oscillibacter sp. 57_20
Sample_T4C.10	T4C.M010	58	2.96	100009	21474	323815	57.23	83.65	98.65	0.00	Clostridiales	Y	N	Firmicutes	Oscillibacter sp.
Sample_T8C.37	T8C.M037	46	1.92	87990	19924	158486	61.86	76.23	92.44	1.01	Clostridiales	Y	N	Firmicutes	Oscillibacter sp.
Sample_C3A.1	C3A.M001	64	2.08	41323	15890	141869	59.29	72.32	84.94	1.90	Clostridiales	Y	Y	Firmicutes	Oscillibacter sp.
Sample_T12A.61	T12A.M061	84	2.15	39498	12237	130644	59.31	39.17	89.93	1.68	Clostridiales	Y	N	Firmicutes	Oscillibacter sp.
Sample_C3A.22	C3A.M022	64	1.53	33425	13553	131844	58.68	25.64	83.26	1.34	Clostridiales	Y	N	Firmicutes	Oscillibacter sp.
Sample_C7A.14	C7A.M014	26	2.03	109975	50403	283358	56.99	24.98	89.93	2.07	Clostridiales	Y	Y	Firmicutes	Oscillibacter sp.
Sample_T5C.44	T5C.M044	94	1.80	33177	9492	73457	62.03	24.71	84.11	1.06	Clostridiales	Y	Y	Firmicutes	Oscillibacter sp.
Sample_T10A.19	T10A.M019	17	1.84	151401	50874	238042	58.28	18.47	94.29	0.67	Clostridiales	Y	N	Firmicutes	Oscillibacter sp.
Sample_T14A.30	T14A.M030	118	1.80	22729	7645	70039	59.87	12.64	92.57	2.01	Clostridiales	Y	N	Firmicutes	Oscillibacter sp.
Sample_T15C.31	T15C.M031	185	1.72	12089	5055	40664	59.86	10.73	80.80	0.67	Clostridiales	Y	Y	Firmicutes	Oscillibacter sp.
Sample_C1C.50	C1C.M050	142	1.59	15037	5662	59402	61.49	8.94	81.40	2.35	Clostridiales	Y	N	Firmicutes	Oscillibacter sp.
Sample_T4C.11	T4C.M011	238	1.99	12130	3765	42045	62.70	8.93	92.59	2.54	Clostridiales	Y	N	Firmicutes	Oscillibacter sp.
Sample_T11A.22	T11A.M022	106	4.08	65415	19806	126893	43.27	11.65	99.01	0.00	Bacteria	N	N	Bacteroidetes	Odoribacter splanchnicus
Sample_T7A.7	T7A.M007	24	1.49	153635	48335	272397	27.18	49.46	92.13	1.85	Bacteria	N	N	Tenericutes	Mycoplasma sp. CAG:956
Sample_C4C.51	C4C.M051	58	1.10	27087	8395	78168	27.28	9.43	82.66	1.33	Bacteria	N	N	Tenericutes	Mycoplasma sp. CAG:877
Sample_C10A.59	C10A.M059	35	1.47	95265	20787	205564	25.33	26.06	92.41	3.37	Bacteria	N	N	Tenericutes	Mycoplasma sp. CAG:472
Sample_C12A.28	C12A.M028	19	1.75	129382	49753	426231	31.09	19.67	100.00	0.00	Euryarchaeota	N	N	Euryarchaeota	Methanobrevibacter smithii CAG:186
Sample_C2C.12	C2C.M012	369	2.12	6952	2975	24054	53.65	7.41	87.68	1.50	Selenomonadales	N	N	Firmicutes	Megasphaera sp. NM10
Sample_C13B.20	C13B.M020	45	2.47	91267	40989	166113	31.38	79.08	100.00	0.63	Firmicutes	N	N	Firmicutes	Megamonas funiformis
Sample_T13B.20	T13B.M020	38	1.99	117472	28778	333451	32.65	110.71	99.47	1.57	Lactobacillales	N	N	Firmicutes	Lactobacillus salivarius
Sample_C3B.25	C3B.M025	50	2.15	62987	26701	211369	43.72	49.40	98.95	0.00	Lactobacillales	N	N	Firmicutes	Lactobacillus ruminis
Sample_T4C.8	T4C.M008	19	2.08	192022	69510	467734	46.74	39.55	99.18	0.00	Lactobacillales	N	N	Firmicutes	Lactobacillus mucosae
Sample_T13A.25	T13A.M025	112	2.09	36169	7776	124938	36.69	10.59	99.02	0.13	Lactobacillus	N	N	Firmicutes	Lactobacillus crispatus
Sample_T17C.10	T17C.M010	88	1.99	47844	9888	120234	38.08	34.86	98.94	0.00	Lactobacillus	N	N	Firmicutes	Lactobacillus amylovorus
Sample_T4C.1	T4C.M001	71	1.93	44927	12896	205435	34.79	10.41	98.83	0.00	Lactobacillus	N	N	Firmicutes	Lactobacillus acidophilus
Sample_C14A.16	C14A.M016	220	2.76	20690	5747	61780	46.69	10.76	91.79	0.00	Clostridiales	N	N	Firmicutes	Lachnospiraceae bacterium AM48-27BH
Sample_T17B.5	T17B.M005	65	2.54	49699	20407	153936	38.70	12.65	97.53	0.00	Clostridiales	N	N	Firmicutes	Lachnospiraceae bacterium AM25-39
Sample_T5B.7	T5B.M007	63	2.78	79073	24320	207089	42.10	49.31	98.65	0.00	Clostridiales	N	N	Firmicutes	Lachnospiraceae bacterium
Sample_C10A.50	C10A.M050	43	2.76	89697	35964	157507	43.99	24.84	98.55	2.17	Lachnospiraceae	N	N	Firmicutes	Lachnospiraceae bacterium

Sample_C4A.36	C4A.M036	52	2.75	117998	24986	363828	40.56	20.40	98.65	0.00	Clostridiales	N	N	Firmicutes	Lachnospiraceae bacterium
Sample_C3B.23	C3B.M023	21	2.62	204619	86003	366618	44.46	15.20	98.65	0.00	Clostridiales	N	N	Firmicutes	Lachnospiraceae bacterium
Sample_T2C.47	T2C.M047	103	3.22	58463	12592	171887	41.47	12.02	94.42	1.63	Lachnospiraceae	N	N	Firmicutes	Lachnospiraceae bacterium
Sample_C8C.2	C8C.M002	80	2.91	53069	17467	157446	46.19	11.29	97.98	0.34	Clostridiales	N	N	Firmicutes	Lachnospiraceae bacterium
Sample_C10A.40	C10A.M040	99	1.97	27884	9390	111638	41.96	9.97	89.43	1.20	Lachnospiraceae	N	N	Firmicutes	Lachnospiraceae bacterium
Sample_T12A.7	T12A.M007	227	2.90	18549	6000	56413	44.14	9.51	94.92	2.28	Lachnospiraceae	N	N	Firmicutes	Lachnospiraceae bacterium
Sample_T16A.22	T16A.M022	80	5.47	158708	29467	378321	57.34	44.09	100.00	0.41	Enterobacteriaceae	N	N	Proteobacteria	Klebsiella pneumoniae
Sample_T8C.29	T8C.M029	104	6.46	89667	32618	265203	49.21	18.66	99.34	2.06	Clostridiales	N	N	Firmicutes	Hungatella hathewayi
Sample_T2A.57	T2A.M057	55	2.18	63660	25021	179384	33.50	16.00	97.30	0.84	Bacteria	N	N	Firmicutes	Holdemanella biformis
Sample_T3A.32	T3A.M032	230	1.92	11154	3935	43762	39.53	14.30	94.57	2.94	Pasteurellaceae	N	N	Proteobacteria	Haemophilus parainfluenzae
Sample_T3B.10	T3B.M010	87	3.28	95887	16796	292219	58.16	37.85	97.27	0.00	Clostridiales	N	N	Firmicutes	Gemmiger formicilis
Sample_C11A.36	C11A.M036	97	2.60	51266	12688	166748	29.35	160.74	98.87	2.25	Bacteria	N	N	Fusobacteria	Fusobacterium varium
Sample_C11A.15	C11A.M015	228	2.02	12559	3770	87691	30.39	9.43	97.19	1.12	Bacteria	N	N	Fusobacteria	Fusobacterium varium
Sample_C13A.34	C13A.M034	173	3.32	34869	8772	110675	30.53	13.95	97.75	2.81	Bacteria	N	N	Fusobacteria	Fusobacterium ulcerans
Sample_C13B.11	C13B.M011	52	2.08	86678	18834	167319	28.34	64.35	98.87	1.12	Bacteria	N	N	Fusobacteria	Fusobacterium mortiferum
Sample_T17B.17	T17B.M017	364	1.86	5952	2597	34608	29.29	7.08	83.14	0.00	Bacteria	N	N	Fusobacteria	Fusobacterium mortiferum
Sample_T3A.23	T3A.M023	36	3.02	147742	43626	371484	48.07	22.79	98.94	0.63	Clostridiales	N	N	Firmicutes	Fusicatenibacter saccharivorans
Sample_T8C.18	T8C.M018	71	2.33	48590	15997	113657	63.70	12.66	96.59	0.86	Clostridiales	N	N	Firmicutes	Fournierella massiliensis
Sample_C8B.35	C8B.M035	127	2.33	28047	8559	172333	61.50	10.48	98.32	3.60	Clostridiales	N	Y	Firmicutes	Flavonifractor sp. An100
Sample_C13C.26	C13C.M026	173	1.97	15115	5280	54105	61.24	8.79	93.93	0.89	Clostridiales	N	N	Firmicutes	Flavonifractor sp. An100
Sample_T11C.29	T11C.M029	95	2.77	45956	13522	138539	62.73	12.14	89.03	0.13	Clostridiales	N	N	Firmicutes	Flavonifractor plautii
Sample_T2A.21	T2A.M021	56	3.04	84292	26671	207728	43.65	22.10	99.77	0.00	Clostridiales	N	N	Firmicutes	Firmicutes bacterium CAG:882
Sample_T4C.19	T4C.M019	31	1.99	95126	38884	243023	60.02	24.30	92.61	0.67	Clostridiales	N	N	Firmicutes	Firmicutes bacterium CAG:83
Sample_T16C.38	T16C.M038	40	1.19	59058	14113	235357	26.63	40.94	93.82	0.00	Bacteria	N	N	Firmicutes	Firmicutes bacterium CAG:822
Sample_T4B.54	T4B.M054	86	1.35	25538	7287	77170	43.02	9.11	94.15	0.88	Clostridia	N	N	Firmicutes	Firmicutes bacterium CAG:552_39_19
Sample_C2B.25	C2B.M025	22	1.37	145242	48937	591300	25.92	21.52	91.57	2.57	Bacteria	N	Y	Firmicutes	Firmicutes bacterium CAG:460
Sample_T4B.45	T4B.M045	31	1.08	70093	16552	183188	26.35	15.19	84.65	2.64	Bacteria	N	N	Firmicutes	Firmicutes bacterium CAG:460
Sample_T12A.38	T12A.M038	65	1.04	23405	7063	82128	26.79	9.58	84.83	0.56	Bacteria	N	N	Firmicutes	Firmicutes bacterium CAG:460
Sample_C8B.25	C8B.M025	30	2.11	197919	55808	518489	36.49	52.11	98.65	0.00	Clostridiales	N	N	Firmicutes	Firmicutes bacterium CAG:341
Sample_C12C.60	C12C.M060	38	1.35	55965	21308	187304	26.00	21.59	92.00	1.33	Bacteria	N	N	Firmicutes	Firmicutes bacterium CAG:321
Sample_T2B.26	T2B.M026	14	1.15	301840	138245	367674	26.64	18.40	93.53	0.00	Bacteria	N	N	Firmicutes	Firmicutes bacterium CAG:321
Sample_C2C.14	C2C.M014	18	1.56	107210	42589	258160	29.34	31.85	100.00	0.11	Bacteria	N	N	Firmicutes	Firmicutes bacterium CAG:313
Sample_T4B.47	T4B.M047	195	1.87	13444	4661	71437	53.80	8.56	88.25	0.67	Clostridiales	N	N	Firmicutes	Firmicutes bacterium CAG:24053_14
Sample_T8C.26	T8C.M026	42	2.20	85900	28274	205527	61.50	24.88	91.94	1.01	Clostridiales	N	N	Firmicutes	Firmicutes bacterium CAG:176_63_11
Sample_C11B.11	C11B.M011	121	1.81	21589	7845	64477	62.34	10.15	90.51	0.67	Clostridiales	N	N	Firmicutes	Firmicutes bacterium CAG:176_63_11
Sample_C8C.31	C8C.M031	31	1.95	106454	31837	296203	60.33	17.66	91.94	0.00	Clostridiales	N	N	Firmicutes	Firmicutes bacterium CAG:176
Sample_C12B.1	C12B.M001	57	2.11	64492	18057	120936	59.39	16.02	93.12	2.56	Clostridiales	N	N	Firmicutes	Firmicutes bacterium CAG:170
Sample_C3A.58	C3A.M058	37	1.50	59677	25331	145901	59.50	13.43	86.01	1.48	Clostridiales	N	Y	Firmicutes	Firmicutes bacterium CAG:137
Sample_C6C.25	C6C.M025	164	1.52	11693	4550	63434	62.54	8.31	86.90	1.57	Clostridiales	N	N	Firmicutes	Firmicutes bacterium CAG:129

Sample_C6C.44	C6C.M044	80	2.50	50646	15158	95455	58.46	17.46	94.63	1.34	Clostridiales	N	N	Firmicutes	Firmicutes bacterium CAG:124
Sample_C14A.48	C14A.M048	91	2.39	42204	14515	245268	61.15	15.71	94.73	0.67	Clostridiales	N	N	Firmicutes	Firmicutes bacterium CAG:124
Sample_T12A.58	T12A.M058	292	2.92	13689	4705	61575	56.12	11.10	84.34	1.34	Clostridiales	N	N	Firmicutes	Firmicutes bacterium CAG:124
Sample_C13A.6	C13A.M006	116	3.24	45985	13774	128048	55.34	28.28	99.10	0.00	Clostridiales	N	N	Firmicutes	Firmicutes bacterium CAG:110
Sample_C6B.55	C6B.M055	69	2.28	50826	17851	165117	57.00	24.12	97.76	0.00	Clostridiales	N	N	Firmicutes	Firmicutes bacterium CAG:110
Sample_C7A.29	C7A.M029	25	2.16	124023	57815	230366	47.83	18.79	97.98	0.00	Clostridiales	N	N	Firmicutes	Firmicutes bacterium CAG:102
Sample_T10B.22	T10B.M022	35	2.78	91493	38361	265474	38.47	22.10	99.36	0.64	Clostridiales	N	N	Firmicutes	Firmicutes bacterium AM10-47
Sample_T12A.18	T12A.M018	39	1.33	46974	19041	138045	26.51	16.24	92.00	0.00	Bacteria	N	Y	Firmicutes	Firmicutes bacterium
Sample_C3A.15	C3A.M015	49	1.29	60061	14979	119543	26.71	14.25	92.41	1.12	Bacteria	N	N	Firmicutes	Firmicutes bacterium
Sample_T2A.41	T2A.M041	72	1.31	29471	9373	116361	24.65	10.71	93.82	0.56	Bacteria	N	N	Firmicutes	Firmicutes bacterium
Sample_T10A.25	T10A.M025	115	2.05	34733	7092	106361	51.59	10.08	97.20	0.00	Clostridiales	N	N	Firmicutes	Firmicutes bacterium
Sample_C13A.28	C13A.M028	110	2.35	46908	9630	144832	42.35	14.10	94.22	1.75	Clostridiales	N	N	Firmicutes	Faecalimonas umbilicata
Sample_C9A.36	C9A.M036	97	2.00	33514	8549	141866	59.81	121.21	93.13	0.17	Clostridiales	N	N	Firmicutes	Faecalibacterium prausnitzii
Sample_C5B.8	C5B.M008	78	2.54	114022	12867	212438	57.73	66.55	99.63	0.17	Clostridiales	N	N	Firmicutes	Faecalibacterium prausnitzii
Sample_C9A.39	C9A.M039	94	2.23	37555	11924	89420	58.45	57.59	94.40	3.57	Clostridiales	N	Y	Firmicutes	Faecalibacterium prausnitzii
Sample_T3A.11	T3A.M011	85	2.09	33121	12710	149739	57.80	44.79	94.86	0.00	Clostridiales	N	N	Firmicutes	Faecalibacterium prausnitzii
Sample_T4C.12	T4C.M012	51	2.16	71192	24802	159641	59.19	24.65	94.62	1.70	Clostridiales	N	N	Firmicutes	Faecalibacterium prausnitzii
Sample_T7C.8	T7C.M008	66	1.92	37756	15699	75650	59.01	21.31	90.35	1.70	Clostridiales	N	N	Firmicutes	Faecalibacterium prausnitzii
Sample_T2B.52	T2B.M052	71	2.01	41199	14881	174680	60.57	18.20	91.83	1.53	Clostridiales	N	N	Firmicutes	Faecalibacterium prausnitzii
Sample_T8C.7	T8C.M007	295	2.00	8304	3601	35127	58.97	7.37	88.32	1.01	Clostridiales	N	N	Firmicutes	Evtepia gabavorous
Sample_T11B.8	T11B.M008	39	2.34	71975	32037	235965	34.56	13.26	99.32	0.00	Clostridiales	N	N	Firmicutes	Eubacterium ventriosum
Sample_C11A.41	C11A.M041	95	2.24	36643	12090	100758	33.93	17.27	97.30	0.84	Bacteria	N	N	Firmicutes	Eubacterium sp. TM06-47
Sample_T10A.45	T10A.M045	64	2.65	119381	25065	370194	39.03	159.61	98.65	0.34	Clostridiales	N	N	Firmicutes	Eubacterium sp. OM08-24
Sample_C5A.11	C5A.M011	25	2.51	160753	52907	337239	36.35	38.27	98.65	0.00	Clostridiales	N	N	Firmicutes	Eubacterium sp. CAG:86
Sample_C2C.22	C2C.M022	17	1.77	165701	82371	226016	45.30	30.18	92.17	0.67	Clostridiales	N	N	Firmicutes	Eubacterium sp. CAG:841
Sample_T1B.7	T1B.M007	17	2.66	243557	71441	453789	36.00	16.31	99.32	0.00	Clostridiales	N	N	Firmicutes	Eubacterium sp. CAG:76
Sample_T2C.23	T2C.M023	57	2.02	61202	14613	154706	34.01	17.30	98.65	0.00	Clostridiales	N	N	Firmicutes	Eubacterium sp. CAG:581
Sample_C6B.9	C6B.M009	51	2.73	85770	28491	243606	32.22	28.12	98.65	0.00	Clostridiales	N	N	Firmicutes	Eubacterium sp. CAG:38
Sample_T6C.5	T6C.M005	35	2.77	132351	47246	297957	41.78	25.22	98.65	0.00	Clostridiales	N	N	Firmicutes	Eubacterium sp. CAG:38
Sample_T16C.21	T16C.M021	15	2.53	269593	119034	420107	33.14	52.65	98.65	0.67	Clostridiales	N	N	Firmicutes	Eubacterium sp. CAG:274
Sample_T11A.20	T11A.M020	19	2.27	183800	94893	305198	33.90	36.97	98.65	1.34	Clostridiales	N	N	Firmicutes	Eubacterium sp. CAG:274
Sample_C4A.13	C4A.M013	214	1.89	10843	4126	48731	33.01	8.36	96.41	0.34	Clostridiales	N	N	Firmicutes	Eubacterium sp. CAG:274
Sample_T2C.14	T2C.M014	47	3.05	103797	42138	220490	36.74	22.30	98.90	0.00	Clostridiales	N	N	Firmicutes	Eubacterium sp. CAG:252
Sample_C12B.26	C12B.M026	21	1.91	279536	38104	321054	38.08	17.49	98.65	0.00	Clostridiales	N	N	Firmicutes	Eubacterium sp. CAG:251
Sample_T7A.33	T7A.M033	146	3.07	37437	9251	83300	52.35	11.75	97.98	0.00	Clostridiales	N	N	Firmicutes	Eubacterium sp. CAG:115
Sample_T10A.21	T10A.M021	390	2.31	7298	2950	24746	37.85	7.14	83.49	1.17	Bacteria	N	N	Firmicutes	Eubacterium sp. AF18-3
Sample_C10B.8	C10B.M008	73	3.20	65985	24365	211479	43.18	43.84	98.43	0.67	Clostridiales	Y	N	Firmicutes	Eubacterium sp.
Sample_C3C.8	C3C.M008	54	2.86	87712	23092	410106	36.07	23.93	97.98	0.67	Clostridiales	Y	N	Firmicutes	Eubacterium sp.
Sample_C6A.21	C6A.M021	110	2.93	40177	13912	141802	41.16	20.28	94.07	1.17	Clostridiales	Y	N	Firmicutes	Eubacterium sp.

Sample_C3C.15	C3C.M015	28	2.44	132159	37577	361100	37.76	18.87	97.86	0.73	Clostridiales	Y	N	Firmicutes	Eubacterium sp.
Sample_T10A.40	T10A.M040	72	2.70	68723	20199	179651	43.31	30.87	95.11	1.29	Lachnospiraceae	N	N	Firmicutes	Eubacterium ramulus
Sample_T16B.8	T16B.M008	71	4.51	141646	29360	397190	50.94	17.36	99.66	0.07	Enterobacteriaceae	N	N	Proteobacteria	Escherichia coli
Sample_T10A.2	T10A.M002	321	2.13	8308	3374	30800	38.37	7.46	90.42	0.56	Lactobacillales	N	N	Firmicutes	Enterococcus hirae
Sample_T13B.4	T13B.M004	29	2.77	154650	50175	306601	37.54	34.39	99.15	0.19	Lactobacillales	N	N	Firmicutes	Enterococcus faecalis
Sample_C3A.2	C3A.M002	71	4.63	118099	31534	491269	55.63	208.96	99.72	0.29	Enterobacteriaceae	N	N	Proteobacteria	Enterobacter cloacae
Sample_T8C.32	T8C.M032	187	7.27	63620	21614	227413	46.74	30.29	98.27	3.74	Lachnospiraceae	N	N	Firmicutes	Eisenbergiella tayi
Sample_C14A.30	C14A.M030	146	1.67	15052	5686	70562	54.23	9.30	99.25	0.12	Actinobacteria	N	N	Actinobacteria	Eggerthella sp. CAG:298
Sample_T11A.13	T11A.M013	86	2.33	48186	12993	181056	41.87	12.10	94.15	0.03	Lachnospiraceae	N	N	Firmicutes	Dorea longicatena
Sample_C10A.66	C10A.M066	165	2.44	26143	6766	96721	51.71	71.42	98.73	0.00	Firmicutes	N	N	Firmicutes	Dialister succinatiphilus
Sample_T7A.6	T7A.M006	102	2.55	49199	12021	200373	52.47	46.60	99.90	0.00	Firmicutes	N	N	Firmicutes	Dialister succinatiphilus
Sample_T14C.28	T14C.M028	100	2.11	34307	9384	189452	48.86	32.70	98.73	0.02	Firmicutes	N	N	Firmicutes	Dialister sp. CAG:357
Sample_T15C.26	T15C.M026	28	1.76	120191	35181	279588	45.39	44.95	98.10	0.63	Firmicutes	N	N	Firmicutes	Dialister invisus
Sample_T10A.3	T10A.M003	231	2.36	14025	4988	63994	58.93	8.45	91.25	1.58	Deltaproteobacteria	Y	N	Proteobacteria	Desulfovibrio sp.
Sample_T12A.48	T12A.M048	329	2.00	7609	3164	30446	64.54	7.87	85.31	1.78	Deltaproteobacteria	N	N	Proteobacteria	Desulfovibrio piger
Sample_T8C.17	T8C.M017	76	3.59	73973	26758	340216	61.13	16.67	99.40	0.00	Deltaproteobacteria	N	N	Proteobacteria	Desulfovibrio fairfieldensis
Sample_T2A.35	T2A.M035	12	1.86	312029	73381	618619	32.20	40.29	89.74	0.85	Bacteria	N	N	Cyanobacteria	Cyanobacteria bacterium UBA10660
Sample_T13B.41	T13B.M041	35	2.11	96997	30118	233689	45.84	13.09	98.38	0.19	Actinobacteria	N	N	Actinobacteria	Cryptobacterium sp. CAG:338
Sample_C1C.51	C1C.M051	23	2.81	233021	60476	552475	44.07	103.92	99.32	0.00	Clostridiales	N	N	Firmicutes	Coprococcus eutactus
Sample_T14B.7	T14B.M007	27	3.09	252707	62717	531669	42.60	53.10	99.32	0.31	Clostridiales	N	N	Firmicutes	Coprococcus eutactus
Sample_C2A.55	C2A.M055	59	2.32	56973	21574	211975	42.97	18.29	91.22	2.56	Lachnospiraceae	N	N	Firmicutes	Coprococcus comes
Sample_C10A.18	C10A.M018	281	2.32	11370	3866	45714	44.24	8.02	85.86	1.01	Clostridiales	N	N	Firmicutes	Coprococcus catus
Sample_C3A.19	C3A.M019	375	2.28	7263	3090	36063	43.66	7.49	81.54	2.47	Clostridiales	N	N	Firmicutes	Coprococcus catus
Sample_T11A.35	T11A.M035	29	2.14	120742	50720	290430	60.04	31.60	100.00	0.81	Actinobacteria	N	N	Actinobacteria	Collinsella aerofaciens
Sample_T3B.4	T3B.M004	63	2.42	78849	19776	198584	59.84	27.73	99.19	0.81	Actinobacteria	N	N	Actinobacteria	Collinsella aerofaciens
Sample_C12B.25	C12B.M025	32	2.22	92143	40888	294291	59.81	20.06	100.00	0.81	Actinobacteria	N	N	Actinobacteria	Collinsella aerofaciens
Sample_C14A.23	C14A.M023	55	2.23	70485	24635	160322	59.92	18.70	96.77	1.61	Actinobacteria	N	N	Actinobacteria	Collinsella aerofaciens
Sample_T13C.32	T13C.M032	54	2.28	93628	24408	233881	59.69	17.49	100.00	1.61	Actinobacteria	N	N	Actinobacteria	Collinsella aerofaciens
Sample_T8A.10	T8A.M010	139	2.19	25344	7827	72622	60.09	12.80	98.38	0.81	Actinobacteria	N	Y	Actinobacteria	Collinsella aerofaciens
Sample_C11B.22	C11B.M022	194	2.20	16750	5225	74191	59.87	10.29	95.56	1.61	Actinobacteria	N	N	Actinobacteria	Collinsella aerofaciens
Sample_T5A.16	T5A.M016	57	3.02	145607	25382	263495	47.10	67.11	98.97	0.00	Clostridiales	N	N	Firmicutes	Clostridium sp. TM06-18
Sample_T16A.6	T16A.M006	47	3.48	124311	43724	231110	44.01	22.52	99.36	0.63	Clostridiales	N	N	Firmicutes	Clostridium sp. TM06-18
Sample_T5A.3	T5A.M003	111	3.05	39059	13014	130560	43.20	22.47	95.80	0.63	Clostridiales	N	N	Firmicutes	Clostridium sp. TM06-18
Sample_T3C.37	T3C.M037	23	3.04	142869	87208	266303	43.77	30.29	98.65	0.00	Clostridiales	N	N	Firmicutes	Clostridium sp. OM07-9AC
Sample_C2B.36	C2B.M036	26	1.11	56793	37668	91153	25.74	41.48	92.13	1.12	Bacteria	N	N	Firmicutes	Clostridium sp. CAG:914
Sample_C2C.10	C2C.M010	15	1.03	93432	39361	348974	27.40	18.24	88.20	1.12	Bacteria	N	N	Firmicutes	Clostridium sp. CAG:710
Sample_T2C.25	T2C.M025	67	1.17	25651	8614	74890	26.88	9.70	93.82	0.00	Bacteria	N	N	Firmicutes	Clostridium sp. CAG:710
Sample_C10A.63	C10A.M063	32	1.14	57347	16765	116948	26.39	51.57	91.57	2.25	Bacteria	N	N	Firmicutes	Clostridium sp. CAG:628
Sample_C11C.8	C11C.M008	401	2.28	6765	2945	27673	53.55	6.90	81.22	1.58	Clostridiales	N	N	Firmicutes	Clostridium sp. CAG:58

Sample_T12A.26	T12A.M026	44	2.96	145719	47110	343956	46.03	27.15	98.55	1.62	Lachnospiraceae	N	N	Firmicutes	Clostridium sp. CAG:510
Sample_T2C.39	T2C.M039	25	1.75	139423	33031	327383	27.13	41.07	80.02	3.24	Clostridiales	N	Y	Firmicutes	Clostridium sp. CAG:492
Sample_C10A.62	C10A.M062	41	1.23	37538	18334	142053	27.94	21.26	93.82	3.93	Bacteria	N	N	Firmicutes	Clostridium sp. CAG:451
Sample_C5A.58	C5A.M058	103	1.44	22926	6531	51666	24.95	9.57	92.69	0.56	Bacteria	N	N	Firmicutes	Clostridium sp. CAG:433
Sample_C12C.4	C12C.M004	4	1.25	486054	275427	489255	27.73	84.76	93.25	1.28	Bacteria	N	N	Firmicutes	Clostridium sp. CAG:417
Sample_C2B.31	C2B.M031	23	2.36	188514	59105	516707	52.47	44.87	97.04	0.00	Clostridiales	N	N	Firmicutes	Clostridium sp. CAG:413
Sample_T12A.35	T12A.M035	298	2.26	9136	3853	38348	28.88	7.95	90.90	2.55	Clostridiales	N	N	Firmicutes	Clostridium sp. CAG:265
Sample_T16B.3	T16B.M003	37	2.69	99335	49197	230667	38.79	14.26	95.30	0.00	Clostridiales	N	N	Firmicutes	Clostridium sp. CAG:230
Sample_C3A.6	C3A.M006	51	2.02	55273	23126	141107	52.91	40.77	96.50	0.00	Clostridia	N	N	Firmicutes	Clostridium sp. CAG:226
Sample_C8B.11	C8B.M011	279	2.31	10411	4041	51518	49.91	9.67	89.88	2.74	Clostridiales	N	N	Firmicutes	Clostridium sp. AM33-3
Sample_T5B.42	T5B.M042	61	2.55	75494	18876	245229	48.97	11.94	99.05	0.00	Clostridiales	N	N	Firmicutes	Clostridium sp. AM28-20LB
Sample_C4C.35	C4C.M035	190	4.17	64454	8622	158477	36.39	18.71	98.65	0.00	Clostridiales	N	N	Firmicutes	Clostridium sp. AF34-13
Sample_C6B.51	C6B.M051	279	2.44	11630	4268	46341	50.10	8.54	90.73	0.18	Clostridiales	N	N	Firmicutes	Clostridium sp. AF27-2AA
Sample_T2C.22	T2C.M022	27	2.69	163964	57421	246850	40.92	47.37	99.32	0.00	Clostridiales	N	N	Firmicutes	Clostridium sp. AF15-41
Sample_T10A.7	T10A.M007	183	2.63	20578	6472	80085	28.62	14.39	93.97	1.41	Clostridiales	N	N	Firmicutes	Clostridium bartlettii CAG:1329
Sample_T4A.23	T4A.M023	33	2.04	226498	33195	432377	61.93	139.04	93.28	0.00	Clostridiales	N	N	Firmicutes	Clostridiales bacterium
Sample_C14C.29	C14C.M029	11	1.97	492194	110107	507677	53.27	49.15	97.31	0.00	Clostridiales	N	N	Firmicutes	Clostridiales bacterium
Sample_T12A.33	T12A.M033	44	1.98	69654	19697	156262	60.36	47.38	93.95	2.01	Clostridiales	N	N	Firmicutes	Clostridiales bacterium
Sample_C12C.8	C12C.M008	48	2.18	67053	26173	158713	56.83	44.84	86.24	1.95	Clostridiales	N	Y	Firmicutes	Clostridiales bacterium
Sample_C12C.16	C12C.M016	20	1.93	142514	66237	277625	49.60	40.05	95.96	0.81	Clostridia	N	N	Firmicutes	Clostridiales bacterium
Sample_T4B.21	T4B.M021	99	2.33	40991	12504	112629	56.86	37.95	98.76	0.04	Clostridiales	N	N	Firmicutes	Clostridiales bacterium
Sample_T8C.10	T8C.M010	30	2.55	173464	48433	316317	60.39	35.28	94.96	1.34	Clostridiales	N	N	Firmicutes	Clostridiales bacterium
Sample_T4A.29	T4A.M029	47	2.30	84468	22403	196328	60.79	31.10	91.94	0.00	Clostridiales	N	N	Firmicutes	Clostridiales bacterium
Sample_T8B.40	T8B.M040	5	1.49	411227	143752	451461	47.56	28.55	92.40	0.70	Clostridiales	N	N	Firmicutes	Clostridiales bacterium
Sample_C3A.45	C3A.M045	92	2.57	63271	9803	215316	56.26	26.23	94.63	3.36	Clostridiales	N	N	Firmicutes	Clostridiales bacterium
Sample_C3A.12	C3A.M012	342	2.44	8693	3400	37241	58.64	22.37	85.50	0.40	Clostridia	N	N	Firmicutes	Clostridiales bacterium
Sample_C12C.54	C12C.M054	182	2.24	16202	5718	49293	57.15	20.33	94.57	1.14	Clostridiales	N	N	Firmicutes	Clostridiales bacterium
Sample_T15C.32	T15C.M032	55	2.47	98451	26056	188503	56.80	17.30	90.60	2.68	Clostridiales	N	Y	Firmicutes	Clostridiales bacterium
Sample_T10A.5	T10A.M005	30	2.34	134615	56849	424356	53.08	16.34	97.90	0.93	Clostridiales	N	N	Firmicutes	Clostridiales bacterium
Sample_C7B.32	C7B.M032	45	1.93	56794	22867	162782	61.56	15.21	94.29	1.01	Clostridiales	N	N	Firmicutes	Clostridiales bacterium
Sample_C4A.23	C4A.M023	61	2.37	63576	19133	260940	56.42	13.93	89.26	1.01	Clostridiales	N	Y	Firmicutes	Clostridiales bacterium
Sample_T4B.13	T4B.M013	73	2.38	51847	17794	185854	59.95	13.33	93.28	1.34	Clostridiales	N	Y	Firmicutes	Clostridiales bacterium
Sample_C1C.22	C1C.M022	144	2.58	31209	7733	131032	54.82	12.35	96.42	0.67	Clostridiales	N	N	Firmicutes	Clostridiales bacterium
Sample_C8B.19	C8B.M019	41	1.84	75471	20448	160587	62.43	12.29	91.94	0.02	Clostridiales	N	N	Firmicutes	Clostridiales bacterium
Sample_C12A.35	C12A.M035	90	2.08	36467	11255	116893	47.02	11.89	92.39	0.13	Clostridiales	N	N	Firmicutes	Clostridiales bacterium
Sample_C14A.21	C14A.M021	138	2.45	36772	7632	200675	52.32	11.68	95.97	0.00	Clostridiales	N	N	Firmicutes	Clostridiales bacterium
Sample_C6C.7	C6C.M007	91	2.34	36790	12846	130513	56.53	11.50	97.90	0.04	Clostridiales	N	N	Firmicutes	Clostridiales bacterium
Sample_C8C.22	C8C.M022	182	3.00	24838	8229	68669	58.23	11.32	99.30	0.93	Clostridiales	N	N	Firmicutes	Clostridiales bacterium
Sample_C3A.14	C3A.M014	235	2.79	16342	5846	69169	58.98	10.41	93.66	0.27	Clostridia	N	N	Firmicutes	Clostridiales bacterium

Sample_C4A.22	C4A.M022	188	2.06	14676	5256	83100	58.35	8.91	89.59	0.00	Clostridiales	N	N	Firmicutes	Clostridiales bacterium
Sample_C12C.30	C12C.M030	305	2.64	12080	4026	63882	54.99	8.89	92.61	0.94	Clostridiales	N	N	Firmicutes	Clostridiales bacterium
Sample_T12A.30	T12A.M030	219	1.98	13191	4077	43380	43.57	8.49	93.58	0.00	Clostridiales	N	N	Firmicutes	Clostridiales bacterium
Sample_T10A.43	T10A.M043	319	2.71	10536	4285	65158	60.90	8.16	91.87	0.96	Firmicutes	N	N	Firmicutes	Clostridiales bacterium
Sample_C3A.29	C3A.M029	324	2.38	9632	3604	36942	58.63	8.03	86.78	3.79	Clostridia	N	N	Firmicutes	Clostridiales bacterium
Sample_C6B.50	C6B.M050	308	2.44	10542	4049	55491	46.41	7.92	96.30	0.34	Clostridiales	N	N	Firmicutes	Clostridiales bacterium
Sample_T17A.23	T17A.M023	75	2.98	60023	22808	154465	48.90	17.78	99.68	0.16	Clostridiales	N	N	Firmicutes	Clostridiaceae bacterium TF01-6
Sample_T8C.15	T8C.M015	89	2.06	35238	11212	156510	53.17	13.97	98.38	1.08	Clostridia	N	N	Firmicutes	Clostridia bacterium
Sample_C2C.9	C2C.M009	123	2.72	35757	12131	95631	54.80	10.21	99.19	0.00	Clostridia	N	N	Firmicutes	Clostridia bacterium
Sample_T1A.11	T1A.M011	120	2.35	33110	9341	99729	33.61	18.24	99.05	0.47	Bacteria	N	N	Firmicutes	Catenibacterium sp. AM22-15
Sample_T2B.15	T2B.M015	27	1.97	138251	29491	445085	32.76	46.39	88.03	0.85	Bacteria	N	N	Melainabacteri	Candidatus Gastranaerophilales bacterium HUM_2
Sample_T12A.6	T12A.M006	94	2.06	33416	10345	99480	37.51	10.51	97.31	1.34	Clostridiales	Y	N	Firmicutes	Butyrivibrio sp.
Sample_T15A.23	T15A.M023	197	4.34	35481	11060	163264	42.22	11.52	98.01	0.00	Bacteria	N	N	Butyricimonas	Butyricimonas virosa
Sample_C8A.13	C8A.M013	820	4.55	6962	2664	36887	42.69	9.18	81.30	3.48	Bacteria	N	N	Butyricimonas	Butyricimonas synergistica
Sample_C9B.31	C9B.M031	174	2.38	20220	6095	104812	57.37	9.73	97.65	0.89	Clostridiales	N	N	Firmicutes	Butyricicoccus sp. OM04-18BH
Sample_C6B.23	C6B.M023	342	2.04	7146	2908	72907	57.99	8.24	87.64	1.01	Clostridiales	N	N	Firmicutes	Butyricicoccus sp. OM04-18BH
Sample_C8C.51	C8C.M051	266	2.28	12067	4285	48981	43.66	7.82	95.00	0.00	Proteobacteria	N	Y	Proteobacteria	Burkholderiales bacterium YL45
Sample_T11A.2	T11A.M002	93	2.71	48359	13640	161614	48.42	34.84	100.00	0.11	Proteobacteria	N	N	Proteobacteria	Burkholderiales bacterium
Sample_T8B.46	T8B.M046	78	3.08	69131	21756	247583	44.82	20.42	98.73	0.63	Clostridiales	N	N	Firmicutes	Blautia sp. OM07-19
Sample_T17A.4	T17A.M004	39	2.64	91661	35344	216567	44.35	114.37	97.37	0.00	Clostridiales	N	N	Firmicutes	Blautia sp. N6H1-15
Sample_T2A.50	T2A.M050	176	2.53	19562	6893	125829	46.10	9.38	95.60	2.00	Clostridiales	N	N	Firmicutes	Blautia sp. KGMB01111
Sample_T17A.22	T17A.M022	23	2.89	163242	70917	472243	43.90	26.95	98.73	0.00	Clostridiales	N	N	Firmicutes	Blautia sp. CAG:257
Sample_C13B.8	C13B.M008	146	3.59	43186	12149	139945	41.52	49.24	97.46	0.00	Clostridiales	N	N	Firmicutes	Blautia obeum
Sample_C2B.43	C2B.M043	74	3.00	66305	20558	205908	41.87	27.89	98.18	0.00	Clostridiales	N	N	Firmicutes	Blautia obeum
Sample_C9A.26	C9A.M026	279	4.22	26219	7072	141645	59.87	12.02	99.70	0.00	Deltaproteobacteria	N	N	Proteobacteria	Bilophila wadsworthia
Sample_C6B.53	C6B.M053	20	2.20	187935	81366	357371	56.29	17.95	100.00	0.23	Bifidobacteriaceae	N	N	Actinobacteria	Bifidobacterium pseudocatenulatum
Sample_C12A.23	C12A.M023	30	2.40	133636	53574	416725	60.09	60.12	100.00	0.00	Bifidobacteriaceae	N	N	Actinobacteria	Bifidobacterium longum
Sample_T10A.9	T10A.M009	321	2.19	8830	3200	51029	58.68	7.71	84.70	1.52	Bifidobacteriaceae	N	N	Actinobacteria	Bifidobacterium dentium
Sample_T4C.3	T4C.M003	30	2.14	100869	40851	277194	62.97	57.38	97.69	0.92	Bifidobacteriaceae	N	N	Actinobacteria	Bifidobacterium bifidum
Sample_T16C.23	T16C.M023	20	1.91	165679	53915	321439	60.47	63.01	100.00	0.00	Bifidobacteriaceae	N	N	Actinobacteria	Bifidobacterium animalis
Sample_C14C.30	C14C.M030	27	2.08	120896	39767	279004	59.71	26.16	99.54	0.11	Bifidobacteriaceae	N	N	Actinobacteria	Bifidobacterium adolescentis
Sample_C6B.45	C6B.M045	330	5.21	23054	7701	71637	42.01	19.46	90.74	2.95	Bacteroides	N	N	Bacteroidetes	Bacteroides xylanisolvens
Sample_C1C.31	C1C.M031	144	4.55	52346	15424	147454	42.19	121.66	98.77	0.38	Bacteroidales	N	N	Bacteroidetes	Bacteroides vulgatus
Sample_T4B.33	T4B.M033	76	4.12	86152	28597	293083	46.66	26.25	98.07	0.00	Bacteroidales	N	N	Bacteroidetes	Bacteroides uniformis
Sample_T3A.13	T3A.M013	75	5.67	133992	37572	319955	42.70	16.47	95.58	0.24	Bacteroides	N	N	Bacteroidetes	Bacteroides thetaiotaomicron
Sample_T18C.24	T18C.M024	39	3.41	127211	48286	431556	46.25	15.07	98.14	1.12	Bacteroidales	N	N	Bacteroidetes	Bacteroides stercoris
Sample_T4B.2	T4B.M002	77	3.25	68160	22735	216099	42.61	25.65	88.59	0.00	Bacteroidales	N	N	Bacteroidetes	Bacteroides sp. CAG:1076
Sample_C9A.20	C9A.M020	76	3.36	62399	22287	199851	42.27	77.65	94.06	0.19	Bacteroidales	N	N	Bacteroidetes	Bacteroides sp. AM16-15
Sample_C6C.40	C6C.M040	35	3.23	224828	57674	359092	43.28	18.99	99.62	0.00	Bacteroidales	N	N	Bacteroidetes	Bacteroides sp. AF14-46

Sample_T17A.20	T17A.M020	70	4.93	134055	41811	226875	43.45	48.96	98.65	1.13	Bacteroidales	N	N	Bacteroidetes	Bacteroides sartorii
Sample_T2C.32	T2C.M032	79	3.60	62599	25797	177373	44.33	81.62	98.75	0.47	Bacteroidales	N	N	Bacteroidetes	Bacteroides plebeius
Sample_T4A.21	T4A.M021	103	6.10	93146	31288	280203	41.88	156.83	97.33	1.43	Bacteroides	N	N	Bacteroidetes	Bacteroides ovatus
Sample_C3A.41	C3A.M041	73	4.84	109816	35744	347393	42.79	26.50	88.71	0.00	Bacteroidales	N	N	Bacteroidetes	Bacteroides intestinalis
Sample_T3A.35	T3A.M035	43	4.73	212391	53850	550226	43.26	26.55	98.31	0.00	Bacteroidales	N	N	Bacteroidetes	Bacteroides fragilis
Sample_T5A.17	T5A.M017	55	4.65	132233	46433	489963	43.38	20.46	97.18	0.00	Bacteroidales	N	N	Bacteroidetes	Bacteroides fragilis
Sample_T15A.17	T15A.M017	90	3.63	57561	18472	309392	44.77	16.23	94.85	3.10	Bacteroidales	N	N	Bacteroidetes	Bacteroides eggerthii
Sample_T10C.7	T10C.M007	66	3.26	98192	24510	180161	46.03	38.70	96.71	0.14	Bacteroidales	N	N	Bacteroidetes	Bacteroides coprophilus
Sample_T7B.14	T7B.M014	112	3.23	47193	14372	108691	40.67	40.82	97.76	0.00	Bacteroidales	N	N	Bacteroidetes	Bacteroides coprocola CAG:162
Sample_T5C.41	T5C.M041	66	3.44	88321	27823	218094	45.62	28.79	95.78	1.38	Bacteroidales	N	N	Bacteroidetes	Bacteroides clarus
Sample_C9A.5	C9A.M005	69	6.20	131979	48611	309863	42.76	25.56	92.72	0.47	Bacteroidales	N	N	Bacteroidetes	Bacteroides cellulosilyticus
Sample_T8C.16	T8C.M016	91	4.48	70720	24233	397618	42.02	54.77	92.13	0.90	Bacteroides	N	N	Bacteroidetes	Bacteroides caccae
Sample_C8C.16	C8C.M016	43	1.93	105578	20913	209440	51.01	33.81	97.84	0.00	Bacteria	N	N	Proteobacteria	Azospirillum sp. 51_20
Sample_T5C.17	T5C.M017	73	1.72	43028	10339	161182	47.29	11.44	95.69	0.00	Bacteria	Y	N	Proteobacteria	Azospirillum sp.
Sample_T18A.9	T18A.M009	40	2.80	120645	36319	239364	63.51	16.89	100.00	0.00	Actinobacteria	N	N	Actinobacteria	Asaccharobacter celatus
Sample_C7C.21	C7C.M021	30	2.02	104972	38656	402937	43.57	45.20	97.31	0.00	Clostridiales	N	N	Firmicutes	Anaerotruncus sp. CAG:528
Sample_T8C.30	T8C.M030	414	2.79	8613	3342	35617	63.87	7.79	91.54	0.36	Clostridiales	N	N	Firmicutes	Anaerotruncus sp. 22A2-44
Sample_C9C.17	C9C.M017	314	2.51	10298	3885	51517	44.95	8.05	95.09	0.78	Clostridiales	N	N	Firmicutes	Anaerotignum lactatifermentans
Sample_T17A.15	T17A.M015	68	3.04	68214	21270	236988	44.76	14.30	99.32	4.03	Clostridiales	N	N	Firmicutes	Anaerostipes sp. CAG:276
Sample_C10B.19	C10B.M019	137	2.42	28368	7210	120530	37.29	24.15	98.32	0.00	Clostridiales	N	N	Firmicutes	Anaerostipes hadrus
Sample_T3B.27	T3B.M027	63	2.68	75040	21443	136260	55.08	11.00	99.27	0.00	Bacteroidetes	N	N	Bacteroidetes	Alistipes sp. AF17-16
Sample_T13B.8	T13B.M008	103	2.53	32697	12948	139307	58.78	10.60	98.95	0.96	Bacteroidetes	N	N	Bacteroidetes	Alistipes sp. AF17-16
Sample_T5C.28	T5C.M028	102	2.57	39578	12657	115342	60.38	10.42	95.67	0.48	Bacteroidetes	N	N	Bacteroidetes	Alistipes sp. 6CPBBH3
Sample_T4A.9	T4A.M009	127	3.65	58039	12871	166492	57.62	44.52	99.03	0.00	Bacteroidetes	N	N	Bacteroidetes	Alistipes shahii
Sample_T6A.2	T6A.M002	307	3.14	13781	5058	73670	58.37	8.51	94.33	1.92	Bacteroidetes	N	N	Bacteroidetes	Alistipes shahii
Sample_T11A.24	T11A.M024	24	2.36	190710	41356	754951	54.50	44.90	99.51	0.96	Bacteroidetes	N	N	Bacteroidetes	Alistipes putredinis
Sample_T16B.22	T16B.M022	20	3.10	387013	149688	603995	58.44	31.63	98.79	0.48	Bacteroidetes	N	N	Bacteroidetes	Alistipes onderdonkii
Sample_C10A.35	C10A.M035	51	2.61	86862	32076	194409	46.21	12.87	99.51	0.48	Bacteroidetes	N	N	Bacteroidetes	Alistipes indistinctus
Sample_C11B.34	C11B.M034	70	2.77	76851	20479	196644	54.86	12.25	100.00	0.48	Bacteroidetes	N	N	Bacteroidetes	Alistipes indistinctus
Sample_C4A.40	C4A.M040	54	2.46	64944	23554	128496	59.22	13.15	89.66	0.48	Bacteroidetes	N	N	Bacteroidetes	Alistipes finegoldii CAG:68
Sample_C14A.10	C14A.M010	35	2.82	188077	52028	492714	55.25	89.23	97.95	0.68	Bacteria	N	N	/errucomicrobi	Akkermansia muciniphila
Sample_C4A.34	C4A.M034	43	3.00	122869	57477	232942	58.26	13.72	97.95	0.00	Bacteria	N	N	/errucomicrobi	Akkermansia muciniphila
Sample_T4B.14	T4B.M014	167	1.67	13785	4784	35100	45.97	8.49	88.64	0.81	Clostridia	N	N	Proteobacteria	Acidiphilium sp. CAG:727
Sample_T13C.38	T13C.M038	41	2.35	86820	30702	285636	56.03	38.96	99.38	0.60	Selenomonadales	N	N	Firmicutes	Acidaminococcus sp. CAG:542
Sample_T14A.43	T14A.M043	74	1.29	35088	6622	109203	24.99	10.79	90.44	2.53	Bacteria	N	N	Tenericutes	Acholeplasma sp. CAG:878
Sample_T4B.18	T4B.M018	43	2.43	96568	28460	252988	53.55	39.95	82.71	0.79	Rhodospirillales	N	N	Proteobacteria	Acetobacter sp. CAG:977
Sample_C13C.9	C13C.M009	46	2.75	103560	30422	245191	43.32	52.36	99.41	0.19	Lachnospiraceae	N	N	Firmicutes	[Ruminococcus] gnavus
Sample_C10A.38	C10A.M038	56	2.75	97128	27490	277919	45.23	48.87	98.99	0.00	Clostridiales	N	N	Firmicutes	[Eubacterium] siraeum
Sample_T15C.19	T15C.M019	79	3.05	105778	25903	194877	41.51	26.03	99.51	0.00	Lachnospiraceae	N	N	Firmicutes	[Eubacterium] rectale

Sample_C5A.41	C5A.M041	48	2.85	90800	37805	300168	37.35	73.24	98.65	0.02	Clostridiales	N	N	Firmicutes	[Eubacterium] eligens
Sample_C4A.32	C4A.M032	26	2.68	140251	49101	670344	37.38	21.97	98.63	0.00	Clostridiales	N	N	Firmicutes	[Eubacterium] eligens
Sample_T17A.9	T17A.M009	388	3.59	14149	4041	57058	45.05	10.08	91.50	0.00	Bacteria	N	N	Firmicutes	[Clostridium] innocuum
Sample_T5A.1	T5A.M001	76	5.95	135433	36632	590821	49.22	29.63	99.25	0.00	Clostridiales	N	N	Firmicutes	[Clostridium] bolteae
Sample_C3C.22	C3C.M022	31	2.68	160829	47299	245494	42.08	52.22	98.65	2.68	Clostridiales	N	N	Bacteroidetes	[Bacteroides] pectinophilus

Table S6. Significant differential species-level genome bins (SGBs) found between subgroups

Differential SGBs		Result statistics (Centered Log-ration transformed abundance)												Comparison between probiotic group at different time points<br>Corrected P-value, T-test		
SGB ID	Probiotic group day 0xonomy	Mean Probiotic group day 0	Mean Placebo group day 0	Mean Probiotic group day 30	Mean Placebo group day 30	Mean Probiotic group day 90	Mean Placebo group day 90	SD Probiotic group day 0	SD Placebo group day 0	SD Probiotic group day 30	SD Placebo group day 30	SD Probiotic group day 90	SD Placebo group day 90	Probiotic group day 0 vs Probiotic group day 30	Probiotic group day 30 vs Probiotic group day 90	Probiotic group day 0 vs Probiotic group day 90
T16C.M023	Bifidobacterium animalis	-0.49	-0.61	1.02	-0.18	1.05	-0.50	0.18	0.13	1.54	1.32	1.57	0.14	0.01	0.98	0.01
C1A.M016	Ruminococcus callidus	-0.21	0.08	0.47	0.06	0.02	0.17	0.77	1.18	1.16	1.04	0.75	1.06	0.05	0.99	0.96
C5A.M066	Parabacteroides distasonis	1.35	1.10	0.63	0.76	0.80	0.92	1.12	1.03	0.86	0.93	0.75	1.15	0.04	0.86	0.97
C14B.M022	Roseburia hominis	0.02	0.22	0.24	0.49	0.24	0.49	0.93	0.96	0.85	1.25	0.85	1.25	0.96	0.99	0.05

Differential SGBs		Result statistics (Centered Log-ration transformed abundance)												Comparison between placebo group at different time points<br>Corrected P-value, T-test		
SGB ID	Probiotic group day 0xonomy	Mean Probiotic group day 0	Mean Placebo group day 0	Mean Probiotic group day 30	Mean Placebo group day 30	Mean Probiotic group day 90	Mean Placebo group day 90	SD Probiotic group day 0	SD Placebo group day 0	SD Probiotic group day 30	SD Placebo group day 30	SD Probiotic group day 90	SD Placebo group day 90	Placebo group day 0 vs Placebo group day 30	Placebo group day 30 vs Placebo group day 90	Placebo group day 0 vs Placebo group day 90
C14A.M016	Lachnospiraceae bacterium AM48-27BH	-0.11	-0.04	0.13	0.56	-0.07	0.44	0.47	0.78	0.58	0.77	0.42	0.63	0.05	0.78	0.01
C8B.M011	Clostridium sp. AM33-3	-0.12	-0.12	-0.07	0.47	0.01	-0.12	0.48	0.62	0.46	0.77	0.57	0.50	0.02	0.04	0.68
T16C.M021	Eubacterium sp. CAG:274	-0.12	-0.11	0.11	0.64	0.58	0.52	0.86	1.01	0.94	1.26	1.66	1.41	0.03	0.96	0.05
T17A.M023	Clostridiaceae bacterium TF01-6	0.58	0.19	0.75	0.90	0.51	0.86	0.91	0.80	0.93	0.79	0.66	0.80	0.03	0.93	0.04
T16A.M006	Clostridium sp. TM06-18	1.34	0.87	1.18	1.62	1.32	0.89	1.49	1.05	1.27	0.71	1.27	0.68	0.03	0.01	0.95
C6C.M025	Firmicutes bacterium CAG:129	0.10	0.66	-0.09	-0.03	0.03	0.66	0.73	1.17	0.53	0.88	0.82	0.96	0.09	0.05	0.99
C12C.M016	Clostridiales bacterium	-0.12	0.31	-0.19	-0.07	0.35	0.80	0.75	1.21	0.74	0.75	1.50	1.34	0.88	0.04	0.69
T8B.M046	Blautia sp. OM07-19	0.39	0.99	0.55	1.11	-0.06	0.17	1.06	1.39	1.24	1.22	0.62	0.96	0.99	0.03	0.08
T17B.M018	Ruminococcus sp. AF37-6AT	0.74	1.11	0.48	0.99	0.44	0.15	1.26	1.13	1.73	1.10	1.53	0.82	0.99	0.03	0.02
T5B.M042	Clostridium sp. AM28-20LB	1.00	1.01	1.04	1.54	0.85	1.03	0.92	0.96	0.82	0.76	0.78	0.63	0.88	0.05	0.99
C13B.M008	Blautia obeum	1.57	2.10	1.94	2.16	1.37	1.33	1.09	1.39	1.29	0.83	1.37	1.02	0.99	0.03	0.62
C14C.M035	uncultured Ruminococcus sp.	-0.32	-0.18	-0.48	-0.09	-0.44	0.85	0.66	1.04	0.17	1.05	0.13	1.60	0.99	0.07	0.05
C1C.M051	Coprococcus eutactus	-0.23	-0.16	0.45	0.34	-0.04	1.07	0.57	1.13	1.31	1.48	0.96	1.79	0.88	0.89	0.04
T4C.M019	Firmicutes bacterium CAG:83	0.81	0.69	0.51	0.33	0.75	-0.30	1.17	1.33	0.98	1.11	1.47	0.42	0.88	0.06	0.02
T10B.M022	Firmicutes bacterium AM10-47	0.22	0.80	0.37	0.28	0.00	-0.11	1.01	1.12	1.05	0.79	0.73	0.75	0.88	0.89	0.02
T4C.M040	uncultured Eubacterium sp.	0.29	0.79	0.38	0.14	0.47	0.11	0.85	0.96	1.08	0.73	1.01	0.87	0.07	0.91	0.05

Differential SGBs		Result statistics (Centered Log-ration transformed abundance)												Comparison between probiotic and placebo groups at different time points<br>Corrected P-value, Wilcoxon-test		
SGB ID	Probiotic group day 0xonomy	Mean Probiotic group day 0	Mean Placebo group day 0	Mean Probiotic group day 30	Mean Placebo group day 30	Mean Probiotic group day 90	Mean Placebo group day 90	SD Probiotic group day 0	SD Placebo group day 0	SD Probiotic group day 30	SD Placebo group day 30	SD Probiotic group day 90	SD Placebo group day 90	Probiotic group day 0 vs Placebo group day 0	Probiotic group day 30 vs Placebo group day 30	Probiotic group day 90 vs Placebo group day 90
C12A.M023	Bifidobacterium longum	0.65	0.67	0.82	0.30	0.83	-0.24	1.78	1.58	1.37	1.15	1.57	0.62	0.91	0.92	0.01
T2B.M023	Prevotella sp. CAG:255	0.28	-0.16	0.26	0.04	0.42	-0.44	1.71	1.40	1.60	1.50	1.87	0.32	0.99	0.93	0.04
C8C.M022	Clostridiales bacterium	0.65	1.60	0.34	0.73	0.08	1.23	1.52	1.53	1.34	1.27	1.20	1.58	0.95	0.93	0.05

Table S7. Distribution of species-level genome bins (SGBs) and predicted metabolic modules across different phyla

Module ID	Annotation	Total number of SGBs	Percentage (%)	Actinobacteria	Bacteroidetes	Cyanobacteria	Euryarchaeota	Firmicutes	Fusobacteria	Melainabacteria	Proteobacteria	Tenericutes	Verrucomicrobia
GMM010	Butyrate synthesis I	108	27.76	0	39	0	0	66	2	0	1	0	0
GMM033	Melatonin synthesis	23	5.91	8	0	0	0	10	0	0	5	0	0
GMM011	Butyrate synthesis II	3	0.77	0	0	0	0	3	0	0	0	0	0
GMM012	Isovaleric acid synthesis I (KADH pathway)	2	0.51	0	1	0	0	1	0	0	0	0	0
GMM013	Isovaleric acid synthesis II (KADC pathway)	110	28.28	12	10	0	0	82	0	0	6	0	0
GMM014	Secondary bile acid biosynthesis	15	3.86	0	6	0	0	5	4	0	0	0	0
GMM036	GABA synthesis I	3	0.77	0	0	0	0	0	0	0	3	0	0
GMM015	Secondary bile acid biosynthesis	18	4.63	0	1	0	0	17	0	0	0	0	0
GMM037	GABA synthesis II	4	1.03	0	0	0	0	0	0	0	4	0	0
GMM038	GABA synthesis III	41	10.54	3	34	0	0	0	1	0	1	0	2
GMM039	GABA degradation	45	11.57	2	1	0	0	37	0	0	5	0	0
GMM030	Quinolinic acid degradation	361	92.80	15	53	1	1	263	5	1	20	0	2
GMM031	Serotonin synthesis I	5	1.29	0	0	0	0	5	0	0	0	0	0
GMM018	PUFAs synthesis (AA, EPA, DHA)	167	42.93	0	28	0	0	118	3	0	18	0	0
GMM019	Histamine synthesis	4	1.03	0	2	0	0	0	2	0	0	0	0
GMM021	Tryptophan synthesis	35	9.00	0	0	0	0	24	0	0	9	0	2
GMM022	Tryptophan degradation	46	11.83	0	21	0	0	22	0	0	1	0	2
GMM001	Acetate synthesis I	368	94.60	15	53	0	0	276	5	0	14	3	2
GMM002	Acetate synthesis II	1	0.26	0	0	0	0	1	0	0	0	0	0
GMM003	Acetate synthesis III	25	6.43	0	6	1	0	16	0	1	0	0	1
GMM005	Acetate degradation	97	24.94	1	37	0	1	52	0	0	6	0	0
GMM006	Propionate synthesis I	2	0.51	0	0	0	0	0	0	0	2	0	0
GMM040	Menaquinone synthesis (vitamin K2) I	50	12.85	3	36	0	0	3	0	0	6	0	2
GMM041	Menaquinone synthesis (vitamin K2) II	10	2.57	0	8	0	0	0	0	0	2	0	0
GMM020	Histamine degradation	3	0.77	0	0	0	0	0	0	0	3	0	0
GMM007	Propionate synthesis II	43	11.05	0	0	0	0	37	4	0	2	0	0
GMM008	Propionate synthesis III	56	14.40	0	44	0	0	10	0	0	0	0	2
GMM009	Propionate degradation I	2	0.51	0	0	0	0	0	0	0	2	0	0

Table S8. Significant differential serum metabolites identified between the probiotic and placebo groups

Differential metabolites	Between the probiotic and placebo groups												rected P-value, Wilcoxon		
	Mean Probiotic group day 0	Mean Placebo group day 0	Mean Probiotic group day 30	Mean Placebo group day 30	Mean Probiotic group day 90	Mean Placebo group day 90	SD Probiotic group day 0	SD Placebo group day 0	SD Probiotic group day 30	SD Placebo group day 30	SD Probiotic group day 90	SD Placebo group day 90	Probiotic group day 0 vs Placebo group day 0	Probiotic group day 30 vs Placebo group day 30	Probiotic group day 90 vs Placebo group day 90
sebacate	0.00011	0.00014	0.00011	0.00013	0.00010	0.00012	0.000032	0.000037	0.000029	0.000031	0.000029	0.000021	0.91	0.04	0.10
adrenic acid	0.00005	0.00005	0.00005	0.00004	0.00005	0.00005	0.000019	0.000011	0.000013	0.000009	0.000016	0.000014	0.99	0.04	0.78
urobilin	0.00063	0.00098	0.00056	0.00089	0.00055	0.00057	0.000481	0.000500	0.000328	0.000333	0.000360	0.000260	0.86	0.03	0.86
linoleoyl ethanolamide	0.00043	0.00038	0.00040	0.00034	0.00039	0.00033	0.000118	0.000084	0.000084	0.000120	0.000075	0.000052	0.73	0.03	0.03
C18.0e.MAG	0.00006	0.00006	0.00006	0.00005	0.00006	0.00006	0.000010	0.000009	0.000007	0.000006	0.000009	0.000006	0.99	0.02	0.39
C18.1 cholesterol ester	0.00011	0.00010	0.00011	0.00010	0.00011	0.00011	0.000015	0.000013	0.000010	0.000007	0.000014	0.000010	0.91	0.05	0.83
C18.0.SM	0.00003	0.00002	0.00003	0.00003	0.00003	0.00003	0.000003	0.000003	0.000002	0.000002	0.000003	0.000003	0.95	0.03	0.92
erythronic acid	0.00012	0.00011	0.00012	0.00010	0.00012	0.00011	0.000028	0.000026	0.000022	0.000020	0.000028	0.000018	0.99	0.05	0.27
methyladipate.pimelate	0.00006	0.00010	0.00006	0.00009	0.00005	0.00006	0.000059	0.000078	0.000058	0.000056	0.000040	0.000048	0.81	0.03	0.39
azelaic acid	0.00005	0.00006	0.00005	0.00006	0.00005	0.00005	0.000012	0.000010	0.000008	0.000008	0.000011	0.000008	0.89	0.03	0.77
N acetylglutamate	0.00026	0.00030	0.00024	0.00028	0.00024	0.00025	0.000082	0.000064	0.000062	0.000049	0.000066	0.000045	0.65	0.03	0.86
pseudouridine	0.00005	0.00005	0.00005	0.00005	0.00005	0.00005	0.000004	0.000005	0.000004	0.000005	0.000005	0.000005	0.91	0.02	0.32
N acetylglutamic.acid	0.00006	0.00007	0.00006	0.00007	0.00006	0.00006	0.000020	0.000013	0.000016	0.000011	0.000015	0.000011	0.91	0.02	0.89

Table S9. Sixteen potential differential serum metabolites identified by LC-MS

**Serum Metabolites**			**VIP Score**		**Signal intensity**												**Corrected P-value, Wilcoxon-test**		
MeProbiotic group day 0bolites	AnnoProbiotic group day 0tion	ChemiPlacebo group day 0l formula	VIP (Probiotic group day 30 vs Placebo group day 30)	VIP (Probiotic group day 90 vs Placebo group day 90)	Mean Probiotic group day 0	Mean Placebo group day 0	Mean Probiotic group day 30	Mean Placebo group day 30	Mean Probiotic group day 90	Mean Placebo group day 90	SD Probiotic group day 0	SD Placebo group day 0	SD Probiotic group day 30	SD Placebo group day 30	SD Probiotic group day 90	SD Placebo group day 90	Probiotic group day 0 vs Placebo group day 0	Probiotic group day 30 vs Placebo group day 30	Probiotic group day 90vs Placebo group day 90
10.50_448.3062m/z	cis-5-Tetradecenoylcarnitine	C21H39N1O4	4.20	2.32	330.26	150.61	64.02	252.45	121.03	192.72	81.39	72.31	80.94	60.56	66.72	41.36	0.240	0.14	0.92
10.25_397.0485m/z	myricetin	C15H10O8	2.13	2.52	211.59	179.03	220.22	166.89	193.81	151.34	89.16	57.91	80.31	62.81	38.57	68.24	0.560	0.23	0.23
10.52_464.3008m/z	O-linoleoylcarnitine	C25H45N1O4	2.48	2.49	123.89	76.17	49.75	81.99	63.02	258.29	72.81	41.46	69.66	26.41	48.02	57.34	0.510	0.08	0.18
14.64_239.0681m/z	succinic acid	C4H6O4	2.92	6.64	136.03	172.96	62.40	160.69	53.50	8.14	46.21	96.00	51.31	97.71	72.93	108.39	0.100	0.02	<0.001
13.60_477.253m/z	Retinoic acid	C20H28O2	2.12	2.91	84.93	86.27	65.26	153.51	148.99	93.74	260.39	139.49	297.06	158.46	176.28	270.20	0.400	0.08	0.08
2.34_279.1724m/z	5-Dodecenoic acid	C12H22O2	2.67	2.39	248.33	235.94	252.61	132.58	225.09	134.04	425.75	170.67	60.40	263.88	107.85	228.67	0.920	0.002	0.02
12.58_583.2535m/z	Schisanhenol	C23H30O6	3.42	3.91	74.31	73.61	62.33	182.99	45.03	108.33	153.98	88.04	56.05	70.85	56.02	341.62	0.860	0.003	0.005
11.59_373.2735m/z	Sphingomyelin (d18:0/16:1(9Z))	C39H79N2O6P1	2.38	3.54	97.50	129.26	48.73	185.60	320.73	111.04	42.64	198.71	0.00	65.89	32.08	157.87	0.540	0.01	0.02

1.14_386.2054m/z	Enterodiol	C18H22O4	2.16	2.43	137.06	93.92	134.03	73.97	105.85	57.29	124.77	159.63	25.33	148.42	392.48	107.92	0.080	0.04	0.05
2.44_277.0709m/z	Syringic acid	C9H10O5	2.38	4.77	87.95	69.98	154.20	243.06	263.01	96.24	96.58	121.15	28.35	199.96	286.72	76.92	0.260	0.02	0.001
10.14_448.1456m/z	L-tryptophan	C11H12N2O2	2.66	3.48	162.59	104.57	189.59	97.05	190.49	116.33	117.64	85.52	126.75	130.52	122.31	27.84	0.061	0.002	0.001
11.73_432.1418n	Prostaglandin E2	C20H32O5	2.09	4.68	137.54	115.57	195.39	135.21	117.76	29.27	73.13	61.26	37.49	127.79	57.97	114.25	0.440	0.17	0.01
10.14_512.1644n	1178-24-1	C22H24O9	3.30	2.86	129.02	78.07	141.51	55.30	129.57	82.86	67.97	56.34	63.87	135.38	88.84	105.33	0.020	<0.001	0.01
11.15_447.297n	Tetracosanoic acid	C24H48O2	9.65	2.27	83.78	179.80	0.00	91.38	55.32	191.53	88.64	98.26	40.17	115.13	31.58	6.10	0.160	<0.001	0.03
10.31_322.1922m/z	3-Methylglutarylcarnitine	C13H23N1O6	2.30	2.27	476.53	401.36	584.36	352.73	395.05	597.97	96.05	117.34	118.61	40.45	119.36	62.60	0.800	0.03	0.03
11.58_408.2866n	1-Palmitoyl-rac-glycerol	C19H38O4	2.47	3.84	133.82	156.35	38.85	129.08	398.58	118.06	46.46	40.75	95.84	100.24	137.18	66.15	0.950	0.06	0.03

Table S10. Viral operational taxonomic units (vOTUs) sequences and assembly statistics

Sample ID	Phage statistics					
	Contig length (kbp)	Gene count	Viral genes	Host genes	Checkv quality	Completeness
Sample_C10A_k141_138972	90197	85	1	44	High-quality	100
Sample_C10A_k141_108304	143968	137	1	67	High-quality	100
Sample_C10A_k141_48221	125630	125	1	74	High-quality	100
Sample_C10A_k141_256565	179066	167	1	129	High-quality	100
Sample_C10A_k141_194585	5735	8	1	0	High-quality	100
Sample_C10A_k141_203515	55218	64	0	14	Complete	100
Sample_C10B_k141_55409	609179	509	3	361	High-quality	100
Sample_C10B_k141_141183	189004	182	5	114	High-quality	100
Sample_C10B_k141_125779	274513	229	4	137	High-quality	100
Sample_C10B_k141_25163	5623	11	0	0	High-quality	100
Sample_C10C_k141_142102	116130	103	1	65	High-quality	100
Sample_C10C_k141_214621	31359	31	0	4	High-quality	100
Sample_C10C_k141_215026	145948	131	1	109	High-quality	100
Sample_C10C_k141_193447	179862	154	1	106	High-quality	100
Sample_C10C_k141_80166	110316	102	1	52	High-quality	100
Sample_C10C_k141_22295	110515	79	1	45	High-quality	100
Sample_C11A_k141_2792	58829	66	1	31	High-quality	100
Sample_C11A_k141_78808	9714	11	1	3	High-quality	100
Sample_C11A_k141_154236	237286	186	1	119	High-quality	100
Sample_C11A_k141_117896	336576	252	1	179	High-quality	100
Sample_C11A_k141_81884	218928	200	1	130	High-quality	100
Sample_C11B_k141_31112	169509	136	16	94	High-quality	100
Sample_C11B_k141_6618	165045	168	1	78	High-quality	100
Sample_C11B_k141_57344	7019	8	1	2	High-quality	100
Sample_C11B_k141_233397	271756	227	2	167	High-quality	100
Sample_C11B_k141_239259	343476	346	3	249	High-quality	100
Sample_C11B_k141_169624	63105	63	1	16	High-quality	100
Sample_C11C_k141_133932	340905	262	1	182	High-quality	100
Sample_C11C_k141_59629	549161	388	1	250	High-quality	100
Sample_C11C_k141_49175	706417	527	1	366	High-quality	100
Sample_C11C_k141_68261	564923	470	1	346	High-quality	100
Sample_C11C_k141_145003	701636	615	1	476	High-quality	100
Sample_C12A_k141_22399	270004	253	3	138	High-quality	100
Sample_C12A_k141_161947	261033	256	1	177	High-quality	100

Sample_C12A_k141_97033	404922	392	2	251	High-quality	100
Sample_C12B_k141_167146	242945	203	1	150	High-quality	100
Sample_C12B_k141_114343	293533	260	4	181	High-quality	100
Sample_C12B_k141_202227	6251	9	2	0	Complete	100
Sample_C12C_k141_144909	69542	67	1	44	High-quality	100
Sample_C12C_k141_129714	210022	170	1	130	High-quality	100
Sample_C12C_k141_227295	159149	132	3	63	Complete	100
Sample_C12C_k141_35184	207633	184	1	137	High-quality	100
Sample_C12C_k141_188111	327561	262	1	163	High-quality	100
Sample_C12C_k141_213286	275427	270	1	144	High-quality	100
Sample_C13A_k141_13032	99355	106	1	62	High-quality	100
Sample_C13A_k141_26915	289712	248	1	176	High-quality	100
Sample_C13A_k141_100484	70516	73	10	23	High-quality	100
Sample_C13A_k141_6605	70487	67	1	50	High-quality	100
Sample_C13A_k141_46041	489148	433	3	315	High-quality	100
Sample_C13C_k141_15292	74445	60	1	35	High-quality	100
Sample_C13C_k141_71895	325734	279	1	202	High-quality	100
Sample_C14C_k141_79190	229951	211	2	143	High-quality	100
Sample_C1A_k141_6186	477185	448	5	276	High-quality	100
Sample_C1A_k141_152452	295228	253	3	186	High-quality	100
Sample_C1A_k141_77737	262925	263	2	205	High-quality	100
Sample_C1B_k141_40809	344353	304	1	248	High-quality	100
Sample_C1B_k141_15965	648307	516	1	411	High-quality	100
Sample_C1C_k141_175795	456219	412	1	332	High-quality	100
Sample_C1C_k141_156768	515294	340	1	252	High-quality	100
Sample_C1C_k141_120693	118763	120	1	66	High-quality	100
Sample_C1C_k141_178916	71932	53	1	38	High-quality	100
Sample_C1C_k141_170835	237712	159	1	121	High-quality	100
Sample_C1C_k141_154161	150919	129	1	82	High-quality	100
Sample_C1C_k141_55759	179973	187	1	117	High-quality	100
Sample_C2A_k141_2148	58614	64	1	33	High-quality	100
Sample_C2A_k141_201050	172760	189	1	106	High-quality	100
Sample_C2A_k141_143708	243300	231	2	155	High-quality	100
Sample_C2A_k141_322474	265453	215	2	172	High-quality	100
Sample_C2A_k141_197769	325961	302	1	246	High-quality	100
Sample_C2B_k141_76325	150177	149	1	67	High-quality	100
Sample_C2B_k141_284761	262896	234	2	140	High-quality	100
Sample_C2B_k141_116891	591300	594	4	316	High-quality	100
Sample_C2B_k141_19228	48937	55	0	24	High-quality	100

Sample_C2B_k141_158930	114485	103	1	64	High-quality	100
Sample_C2B_k141_309501	192495	131	1	95	High-quality	100
Sample_C2C_k141_65504	395426	396	1	319	High-quality	100
Sample_C2C_k141_226562	348974	363	3	234	High-quality	100
Sample_C2C_k141_267130	443833	416	2	320	High-quality	100
Sample_C2C_k141_210733	14018	22	2	4	Complete	100
Sample_C2C_k141_50758	26539	30	2	2	High-quality	100
Sample_C2C_k141_181624	106773	113	1	56	High-quality	100
Sample_C2C_k141_314636	93501	103	1	59	High-quality	100
Sample_C2C_k141_157626	112637	132	2	60	High-quality	100
Sample_C3A_k141_131742	208510	174	1	123	High-quality	100
Sample_C3A_k141_60104	179600	155	1	106	High-quality	100
Sample_C3A_k141_86444	369841	281	1	203	High-quality	100
Sample_C3A_k141_87497	109816	75	1	51	High-quality	100
Sample_C3A_k141_38683	324859	242	1	168	High-quality	100
Sample_C3A_k141_94696	47310	74	12	1	High-quality	100
Sample_C3B_k141_213481	172368	137	1	85	High-quality	100
Sample_C3C_k141_28211	142683	150	2	72	High-quality	100
Sample_C3C_k141_136716	477936	546	4	327	High-quality	100
Sample_C3C_k141_171266	562487	617	4	378	High-quality	100
Sample_C3C_k141_227275	601314	576	1	480	High-quality	100
Sample_C3C_k141_199893	402943	374	1	294	High-quality	100
Sample_C3C_k141_182680	361100	320	1	225	High-quality	100
Sample_C4A_k141_26674	409593	358	2	265	High-quality	100
Sample_C4A_k141_82383	56274	48	2	16	High-quality	100
Sample_C4A_k141_61468	140443	154	7	77	Complete	100
Sample_C4A_k141_220594	296892	268	2	185	High-quality	100
Sample_C4A_k141_18396	236366	194	1	130	High-quality	100
Sample_C4A_k141_154973	187672	140	1	95	High-quality	100
Sample_C4B_k141_108219	74061	69	1	56	High-quality	100
Sample_C4B_k141_192562	192270	162	1	123	High-quality	100
Sample_C4B_k141_90906	79879	77	1	44	High-quality	100
Sample_C4B_k141_253004	158628	155	1	101	High-quality	100
Sample_C4B_k141_172558	70618	72	1	47	High-quality	100
Sample_C4B_k141_75716	83715	68	1	36	High-quality	100
Sample_C4B_k141_101799	260949	242	1	195	High-quality	100
Sample_C4C_k141_26966	321314	318	3	173	High-quality	100
Sample_C4C_k141_238383	362370	321	1	254	High-quality	100
Sample_C5A_k141_58954	5070	7	1	0	High-quality	100

Sample_C5A_k141_177353	39814	38	0	21	High-quality	100
Sample_C5A_k141_130903	88065	97	1	65	High-quality	100
Sample_C5A_k141_13296	189944	173	2	134	High-quality	100
Sample_C5B_k141_35482	810636	766	5	564	High-quality	100
Sample_C5B_k141_58135	161166	147	1	112	High-quality	100
Sample_C5C_k141_49338	27999	41	1	12	High-quality	100
Sample_C5C_k141_14578	907762	810	6	622	High-quality	100
Sample_C5C_k141_28799	452505	429	4	290	High-quality	100
Sample_C5C_k141_103495	152589	132	1	103	High-quality	100
Sample_C6A_k141_60782	87918	69	1	47	High-quality	100
Sample_C6B_k141_112047	262409	216	2	152	High-quality	100
Sample_C6B_k141_119019	184175	161	1	117	High-quality	100
Sample_C6C_k141_53742	346974	355	4	204	Complete	100
Sample_C6C_k141_5379	291786	285	1	198	High-quality	100
Sample_C6C_k141_81651	199475	196	1	109	High-quality	100
Sample_C6C_k141_42621	338414	230	1	154	High-quality	100
Sample_C6C_k141_192161	305221	251	1	173	High-quality	100
Sample_C8A_k141_73723	577400	551	4	388	High-quality	100
Sample_C8A_k141_94955	180157	137	1	94	High-quality	100
Sample_C8A_k141_58309	19807	20	3	10	High-quality	100
Sample_C8A_k141_149906	136063	149	1	21	Complete	100
Sample_C8A_k141_3432	589334	578	4	385	High-quality	100
Sample_C8A_k141_100037	319725	308	1	235	High-quality	100
Sample_C8B_k141_160293	85246	67	1	33	High-quality	100
Sample_C8B_k141_120934	135058	125	1	91	High-quality	100
Sample_C8B_k141_207778	11477	17	0	1	Complete	100
Sample_C8C_k141_143075	29665	36	3	3	High-quality	100
Sample_C8C_k141_125805	264937	226	1	170	High-quality	100
Sample_C8C_k141_3097	192288	162	1	93	High-quality	100
Sample_C8C_k141_21407	544217	442	1	295	High-quality	100
Sample_C8C_k141_40639	476756	408	1	317	High-quality	100
Sample_C8C_k141_131694	301878	278	1	171	High-quality	100
Sample_C8C_k141_134343	172933	175	1	116	High-quality	100
Sample_C8C_k141_136297	179016	141	1	105	High-quality	100
Sample_C8C_k141_64766	383477	302	3	210	High-quality	100
Sample_C8C_k141_16985	306301	234	1	154	High-quality	100
Sample_C8C_k141_106285	201859	202	2	158	High-quality	100
Sample_C9A_k141_68640	500387	398	3	320	High-quality	100
Sample_C9A_k141_98058	232904	180	1	128	High-quality	100

Sample_C9A_k141_5966	395272	310	2	223	High-quality	100
Sample_C9A_k141_90014	676169	580	1	429	High-quality	100
Sample_C9A_k141_92638	43780	41	1	16	High-quality	100
Sample_C9A_k141_105626	366947	246	1	191	High-quality	100
Sample_C9B_k141_18064	287933	180	1	137	High-quality	100
Sample_C9B_k141_55488	314428	261	1	177	High-quality	100
Sample_C9C_k141_83008	414054	324	1	236	High-quality	100
Sample_C9C_k141_10722	347165	263	2	199	High-quality	100
Sample_C9C_k141_35940	262516	238	1	175	High-quality	100
Sample_C9C_k141_15519	299062	260	1	190	High-quality	100
Sample_T10A_k141_1263	40797	36	0	24	High-quality	100
Sample_T10A_k141_124884	354262	285	11	193	High-quality	100
Sample_T10A_k141_34010	370194	362	3	265	High-quality	100
Sample_T10A_k141_184369	296025	203	1	140	High-quality	100
Sample_T10A_k141_184457	10651	15	2	4	Complete	100
Sample_T10B_k141_136006	260032	236	1	182	High-quality	100
Sample_T10B_k141_86246	55112	55	1	13	High-quality	100
Sample_T10B_k141_88934	194609	180	1	117	High-quality	100
Sample_T10B_k141_55634	496554	453	3	343	High-quality	100
Sample_T11A_k141_107952	314887	282	5	192	High-quality	100
Sample_T11A_k141_124706	317880	264	3	190	High-quality	100
Sample_T11A_k141_17704	269244	194	1	136	High-quality	100
Sample_T11A_k141_2552	305198	292	3	202	High-quality	100
Sample_T11A_k141_50042	249890	213	1	156	High-quality	100
Sample_T11A_k141_145237	379809	338	1	183	High-quality	100
Sample_T11A_k141_7302	187698	204	1	70	High-quality	100
Sample_T11A_k141_115926	181056	171	2	133	High-quality	100
Sample_T11B_k141_114581	20919	28	1	5	High-quality	100
Sample_T11B_k141_150680	160650	137	1	100	High-quality	100
Sample_T11B_k141_137924	235965	189	1	143	High-quality	100
Sample_T11B_k141_14501	7281	11	0	2	High-quality	100
Sample_T11C_k141_73711	45421	39	1	11	High-quality	100
Sample_T11C_k141_13423	359814	303	1	222	High-quality	100
Sample_T12A_k141_40024	344909	282	1	195	High-quality	100
Sample_T12A_k141_46083	20240	25	1	3	High-quality	100
Sample_T12A_k141_340991	138045	145	1	77	High-quality	100
Sample_T12B_k141_137132	41904	40	1	11	High-quality	100
Sample_T12B_k141_178670	564984	505	2	377	High-quality	100
Sample_T12B_k141_72850	190627	171	1	120	High-quality	100

Sample_T12C_k141_177251	11570	11	3	2	High-quality	100
Sample_T12C_k141_169558	333788	269	1	188	High-quality	100
Sample_T12C_k141_13986	175579	188	1	123	High-quality	100
Sample_T12C_k141_191073	304703	249	1	183	High-quality	100
Sample_T12C_k141_155620	179379	135	1	99	High-quality	100
Sample_T13A_k141_79882	565961	455	2	343	High-quality	100
Sample_T13A_k141_28090	63003	69	2	20	High-quality	100
Sample_T13A_k141_108489	427150	406	1	304	High-quality	100
Sample_T13A_k141_110821	223347	169	1	120	High-quality	100
Sample_T13A_k141_74588	146473	135	1	82	High-quality	100
Sample_T13B_k141_111338	535180	391	3	287	High-quality	100
Sample_T13B_k141_37988	321473	260	1	202	High-quality	100
Sample_T13B_k141_130065	381714	275	1	157	High-quality	100
Sample_T13B_k141_38196	252338	191	1	126	High-quality	100
Sample_T13B_k141_149892	306601	285	2	216	High-quality	100
Sample_T13B_k141_76154	128847	129	6	27	High-quality	100
Sample_T13B_k141_97853	249588	226	2	129	High-quality	100
Sample_T13B_k141_159054	403857	335	1	254	High-quality	100
Sample_T13B_k141_179570	333451	322	3	245	High-quality	100
Sample_T13B_k141_86651	115163	115	1	57	High-quality	100
Sample_T13B_k141_166007	328139	325	1	250	High-quality	100
Sample_T13C_k141_134514	285409	247	2	147	High-quality	100
Sample_T13C_k141_5043	209463	200	1	125	High-quality	100
Sample_T14A_k141_240234	225140	201	3	110	High-quality	100
Sample_T14B_k141_106966	37891	37	1	17	High-quality	100
Sample_T14B_k141_218812	6861	8	0	0	Complete	100
Sample_T14C_k141_66626	820226	681	3	469	High-quality	100
Sample_T14C_k141_121639	252707	245	3	176	High-quality	100
Sample_T14C_k141_12876	78394	91	1	26	High-quality	100
Sample_T15B_k141_98005	175695	163	1	121	High-quality	100
Sample_T15C_k141_76753	265578	196	2	150	High-quality	100
Sample_T15C_k141_78243	417654	361	6	252	High-quality	100
Sample_T15C_k141_52156	83743	86	2	43	High-quality	100
Sample_T15C_k141_82737	268050	240	3	141	High-quality	100
Sample_T16B_k141_59827	194457	189	1	93	High-quality	100
Sample_T16B_k141_173991	603995	500	1	358	High-quality	100
Sample_T16C_k141_123619	127459	117	1	90	High-quality	100
Sample_T16C_k141_65004	94418	112	20	28	High-quality	100
Sample_T16C_k141_96566	287542	265	1	205	High-quality	100

Sample_T16C_k141_40217	598372	511	3	318	High-quality	100
Sample_T16C_k141_116315	235357	262	3	172	High-quality	100
Sample_T17A_k141_27293	209384	186	1	102	High-quality	100
Sample_T17A_k141_32922	286134	239	1	145	High-quality	100
Sample_T17A_k141_47722	373909	278	1	214	High-quality	100
Sample_T17B_k141_11617	272547	244	2	141	High-quality	100
Sample_T17B_k141_4093	169299	158	3	90	High-quality	100
Sample_T17B_k141_12484	190772	152	1	99	High-quality	100
Sample_T17B_k141_2133	261351	225	1	160	High-quality	100
Sample_T17B_k141_2816	82529	74	1	51	High-quality	100
Sample_T17C_k141_36059	369566	342	1	283	High-quality	100
Sample_T17C_k141_16855	431793	401	3	304	High-quality	100
Sample_T18A_k141_118019	678232	641	2	478	High-quality	100
Sample_T18B_k141_63392	402554	268	2	191	High-quality	100
Sample_T18B_k141_64336	152027	127	3	88	High-quality	100
Sample_T18C_k141_16652	431556	345	1	251	High-quality	100
Sample_T18C_k141_2197	283257	235	1	164	High-quality	100
Sample_T18C_k141_51396	159152	140	1	103	High-quality	100
Sample_T18C_k141_116773	12668	18	1	1	High-quality	100
Sample_T18C_k141_137172	273221	231	6	136	High-quality	100
Sample_T1A_k141_37716	245323	208	1	170	High-quality	100
Sample_T1C_k141_44118	132156	140	2	89	High-quality	100
Sample_T2A_k141_195756	364799	360	1	292	High-quality	100
Sample_T2A_k141_253531	433472	392	2	305	High-quality	100
Sample_T2A_k141_114109	325004	315	1	267	High-quality	100
Sample_T2A_k141_86349	618619	597	2	324	High-quality	100
Sample_T2A_k141_89410	178283	191	2	117	High-quality	100
Sample_T2A_k141_233154	574080	492	2	346	High-quality	100
Sample_T2A_k141_233913	197311	199	1	102	High-quality	100
Sample_T2A_k141_121999	283073	259	1	188	High-quality	100
Sample_T2A_k141_97844	646320	494	3	373	High-quality	100
Sample_T2A_k141_240908	583019	560	2	439	High-quality	100
Sample_T2B_k141_101370	445085	453	1	244	High-quality	100
Sample_T2B_k141_21406	7611	8	0	0	Complete	100
Sample_T2B_k141_25453	367674	404	2	247	High-quality	100
Sample_T2C_k141_138961	89115	103	1	58	High-quality	100
Sample_T2C_k141_58126	403550	300	1	214	High-quality	100
Sample_T2C_k141_34343	299574	221	1	115	High-quality	100
Sample_T2C_k141_92046	210162	186	3	151	High-quality	100

Sample_T2C_k141_37492	104365	82	1	51	High-quality	100
Sample_T2C_k141_232490	307023	244	1	166	High-quality	100
Sample_T2C_k141_121167	208833	148	1	30	High-quality	100
Sample_T2C_k141_263151	327383	314	2	203	High-quality	100
Sample_T2C_k141_99810	119257	103	1	56	High-quality	100
Sample_T2C_k141_186247	325643	323	4	191	High-quality	100
Sample_T2C_k141_24894	118561	96	1	55	High-quality	100
Sample_T2C_k141_277022	69971	85	2	12	Complete	100
Sample_T3A_k141_41905	182596	126	1	86	High-quality	100
Sample_T3A_k141_42748	319955	265	1	190	High-quality	100
Sample_T3A_k141_99571	289258	183	2	124	High-quality	100
Sample_T3A_k141_89450	255361	222	5	127	High-quality	100
Sample_T3A_k141_50663	313132	203	1	144	High-quality	100
Sample_T3A_k141_120020	127202	125	1	75	High-quality	100
Sample_T3A_k141_78011	380922	295	2	235	High-quality	100
Sample_T3B_k141_154194	348944	278	1	177	High-quality	100
Sample_T3B_k141_80617	325002	298	1	200	High-quality	100
Sample_T3C_k141_59000	517161	371	1	277	High-quality	100
Sample_T3C_k141_104046	260737	183	1	132	High-quality	100
Sample_T3C_k141_60148	494179	460	1	369	High-quality	100
Sample_T3C_k141_106726	149923	115	1	46	High-quality	100
Sample_T3C_k141_54526	498947	464	2	293	High-quality	100
Sample_T3C_k141_84106	301505	290	1	227	High-quality	100
Sample_T4A_k141_39425	21966	25	1	13	High-quality	100
Sample_T4A_k141_79090	144657	125	1	85	High-quality	100
Sample_T4A_k141_43016	139867	121	1	22	High-quality	100
Sample_T4A_k141_31143	162666	113	1	74	High-quality	100
Sample_T4A_k141_94653	252008	267	2	162	High-quality	100
Sample_T4B_k141_227527	112526	113	1	71	High-quality	100
Sample_T4B_k141_195484	90021	105	1	75	High-quality	100
Sample_T4B_k141_48248	22985	25	1	2	High-quality	100
Sample_T4B_k141_163547	87666	86	1	50	High-quality	100
Sample_T4B_k141_275846	212688	250	1	146	High-quality	100
Sample_T4B_k141_243011	95686	93	1	63	High-quality	100
Sample_T4B_k141_359473	132547	134	1	67	High-quality	100
Sample_T4C_k141_39067	504869	355	1	235	High-quality	100
Sample_T4C_k141_15258	101590	109	22	14	High-quality	100
Sample_T4C_k141_53309	175928	109	1	73	High-quality	100
Sample_T4C_k141_17794	277194	214	2	149	High-quality	100

Sample_T4C_k141_72477	108673	98	1	65	High-quality	100
Sample_T4C_k141_112769	215068	178	1	110	High-quality	100
Sample_T5A_k141_42418	413129	394	2	224	High-quality	100
Sample_T5A_k141_39775	375963	298	1	198	High-quality	100
Sample_T5A_k141_14576	296015	223	1	129	High-quality	100
Sample_T5A_k141_40545	173280	125	1	83	High-quality	100
Sample_T5A_k141_45939	180640	168	1	96	High-quality	100
Sample_T5A_k141_15638	6025	10	2	0	High-quality	100
Sample_T5B_k141_41141	313284	223	1	158	High-quality	100
Sample_T5B_k141_11416	155292	120	16	75	High-quality	100
Sample_T5B_k141_79996	173776	167	1	93	High-quality	100
Sample_T5B_k141_39913	139562	111	2	64	High-quality	100
Sample_T5B_k141_32189	321829	231	1	180	High-quality	100
Sample_T5B_k141_49131	540098	428	2	327	High-quality	100
Sample_T5C_k141_21128	108818	104	2	49	High-quality	100
Sample_T5C_k141_101497	318017	253	1	159	High-quality	100
Sample_T5C_k141_61589	70268	78	1	39	High-quality	100
Sample_T5C_k141_104078	534224	482	1	384	High-quality	100
Sample_T5C_k141_109714	83289	74	1	38	High-quality	100
Sample_T5C_k141_76010	203460	137	1	98	High-quality	100
Sample_T6A_k141_223364	186648	175	1	128	High-quality	100
Sample_T6A_k141_162924	97306	98	1	33	High-quality	100
Sample_T6A_k141_131858	456279	345	1	262	High-quality	100
Sample_T6B_k141_24490	282218	217	3	149	High-quality	100
Sample_T6B_k141_187919	80336	91	25	10	High-quality	100
Sample_T6B_k141_127866	397828	333	1	198	High-quality	100
Sample_T6C_k141_224146	162277	137	1	94	High-quality	100
Sample_T7A_k141_69836	303449	258	1	181	High-quality	100
Sample_T7A_k141_116373	72749	87	17	8	High-quality	100
Sample_T7A_k141_26373	692608	607	3	489	High-quality	100
Sample_T7A_k141_141690	323919	269	1	202	High-quality	100
Sample_T7A_k141_33838	272397	265	3	131	High-quality	100
Sample_T7A_k141_38683	130406	109	1	72	High-quality	100
Sample_T7A_k141_154900	38705	31	1	4	Complete	100
Sample_T7A_k141_40065	225445	248	3	126	High-quality	100
Sample_T7B_k141_76897	631945	519	2	360	High-quality	100
Sample_T7C_k141_155544	157642	138	2	82	High-quality	100
Sample_T8A_k141_104811	42854	39	2	4	High-quality	100
Sample_T8A_k141_63251	33887	42	4	8	High-quality	100

Sample_T8A_k141_65961	34811	20	1	9	High-quality	100
Sample_T8A_k141_41463	162594	148	2	84	High-quality	100
Sample_T8A_k141_19567	42624	38	1	21	High-quality	100
Sample_T8A_k141_102859	462866	432	1	331	High-quality	100
Sample_T8A_k141_50886	50232	34	0	23	High-quality	100
Sample_T8B_k141_171140	228345	208	1	169	High-quality	100
Sample_T8B_k141_226962	391194	358	1	276	High-quality	100
Sample_T8B_k141_34368	81409	69	1	45	High-quality	100
Sample_T8B_k141_122070	46910	57	1	17	High-quality	100
Sample_T8C_k141_157493	148026	134	2	85	High-quality	100
Sample_T8C_k141_91163	201722	143	1	102	High-quality	100
Sample_T8C_k141_137660	70786	72	1	33	High-quality	100
Sample_T8C_k141_15608	193561	197	1	117	High-quality	100
Sample_C10A_k141_179928	105515	130	8	2	Complete	100
Sample_C10A_k141_342425	46023	76	45	0	Complete	100
Sample_C10A_k141_177158	74529	103	20	7	High-quality	100
Sample_C10A_k141_18034	11072	11	3	0	High-quality	100
Sample_C10A_k141_208919	182940	215	22	8	Complete	100
Sample_C10A_k141_318893	39078	62	16	1	Complete	100
Sample_C10A_k141_105950_fragment_1	49214	67	19	7	High-quality	100
Sample_C10A_k141_227889	6477	9	3	0	High-quality	100
Sample_C10A_k141_261965	83107	102	35	0	High-quality	100
Sample_C10A_k141_84788	55546	69	24	5	High-quality	100
Sample_C10A_k141_11358	42024	45	12	0	Complete	100
Sample_C10A_k141_94481_fragment_1	49004	59	3	3	High-quality	100
Sample_C10A_k141_155992_fragment_2	57371	57	21	3	High-quality	100
Sample_C10A_k141_332781_fragment_1	89273	89	27	11	High-quality	100
Sample_C10A_k141_121260_fragment_6	36584	62	19	5	High-quality	100
Sample_C10A_k141_202327	40076	54	7	1	High-quality	100
Sample_C10A_k141_205041_fragment_1	57203	91	22	3	High-quality	100
Sample_C10A_k141_176489_fragment_2	69084	76	25	10	Complete	100
Sample_C10A_k141_315916_fragment_2	6804	8	1	0	High-quality	100
Sample_C10A_k141_24305	34618	60	15	0	Complete	100
Sample_C10A_k141_275743_fragment_1	80647	103	41	3	High-quality	100
Sample_C10A_k141_342412	29587	47	11	1	Complete	100
Sample_C10B_k141_169764	78278	123	16	1	Complete	100
Sample_C10B_k141_105462_fragment_2	57980	100	20	1	High-quality	100
Sample_C10B_k141_140612	66691	105	21	3	High-quality	100
Sample_C10B_k141_13371_fragment_1	42750	67	25	2	High-quality	100

Sample_C10B_k141_35347	74803	127	17	3	Complete	100
Sample_C10B_k141_141957_fragment_1	69149	108	32	7	High-quality	100
Sample_C10B_k141_149061_fragment_2	38008	60	19	0	High-quality	100
Sample_C10B_k141_43765_fragment_2	48120	63	17	2	High-quality	100
Sample_C10C_k141_155910	38533	58	25	3	Complete	100
Sample_C10C_k141_93734	64135	93	21	3	High-quality	100
Sample_C10C_k141_161317_fragment_1	70020	98	34	5	High-quality	100
Sample_C10C_k141_145299_fragment_4	64338	71	24	4	High-quality	100
Sample_C10C_k141_147492	5122	7	1	1	High-quality	100
Sample_C10C_k141_6215_fragment_1	10412	11	1	0	High-quality	100
Sample_C10C_k141_214497_fragment_1	41902	49	5	3	High-quality	100
Sample_C10C_k141_230021	38933	55	20	1	Complete	100
Sample_C10C_k141_193041	55078	86	22	1	High-quality	100
Sample_C11A_k141_187220	197925	234	44	9	Complete	100
Sample_C11A_k141_44238_fragment_1	36344	45	5	4	High-quality	100
Sample_C11A_k141_14876_fragment_2	96154	136	20	10	Complete	100
Sample_C11A_k141_24590	49907	72	17	0	Complete	100
Sample_C11A_k141_122841_fragment_1	44509	66	21	1	High-quality	100
Sample_C11A_k141_187219	93141	95	10	0	Complete	100
Sample_C11A_k141_112355	43828	65	18	3	High-quality	100
Sample_C11A_k141_187226	185605	230	31	4	Complete	100
Sample_C11B_k141_249306	45759	57	40	0	Complete	100
Sample_C11B_k141_249302	39839	46	44	0	Complete	100
Sample_C11B_k141_89013_fragment_1	32943	39	29	0	High-quality	100
Sample_C11B_k141_210614	45779	69	42	2	High-quality	100
Sample_C11B_k141_77470	16069	24	8	0	Complete	100
Sample_C11B_k141_6650_fragment_1	51036	68	22	4	High-quality	100
Sample_C11B_k141_156496	62941	84	20	5	High-quality	100
Sample_C11B_k141_226505_fragment_1	85461	108	23	1	High-quality	100
Sample_C11C_k141_112644_fragment_2	53353	68	19	7	Complete	100
Sample_C11C_k141_150983_fragment_2	65451	105	36	1	High-quality	100
Sample_C11C_k141_22325_fragment_1	47073	54	3	5	High-quality	100
Sample_C11C_k141_187515	44816	78	50	0	Complete	100
Sample_C11C_k141_46924_fragment_1	40781	63	21	1	High-quality	100
Sample_C11C_k141_150287	45760	74	21	0	Complete	100
Sample_C11C_k141_30923	61806	98	33	0	High-quality	100
Sample_C11C_k141_430	64636	108	30	1	High-quality	100
Sample_C11C_k141_187506	43872	51	23	0	Complete	100
Sample_C11C_k141_187521	6417	7	4	0	Complete	100

Sample_C12A_k141_214076	59934	81	14	0	Complete	100
Sample_C12A_k141_35056	40907	63	19	5	Complete	100
Sample_C12A_k141_107526	41223	66	21	0	High-quality	100
Sample_C12A_k141_112072	43733	55	20	1	Complete	100
Sample_C12A_k141_52170_fragment_2	84600	142	16	5	High-quality	100
Sample_C12A_k141_214067	100355	95	16	1	Complete	100
Sample_C12B_k141_81560_fragment_1	39425	66	22	1	High-quality	100
Sample_C12B_k141_202229	7278	10	3	0	Complete	100
Sample_C12B_k141_71926_fragment_1	42944	69	21	2	High-quality	100
Sample_C12B_k141_71950	42946	55	25	1	High-quality	100
Sample_C12B_k141_76190_fragment_1	11364	17	0	2	High-quality	100
Sample_C12B_k141_202242	39463	68	18	0	Complete	100
Sample_C12C_k141_7702_fragment_4	44749	57	20	5	Complete	100
Sample_C12C_k141_225171	58267	91	21	1	High-quality	100
Sample_C12C_k141_108798	41426	52	12	3	High-quality	100
Sample_C12C_k141_216980_fragment_1	42330	48	23	5	Complete	100
Sample_C12C_k141_51799	102024	170	16	2	Complete	100
Sample_C12C_k141_3082	39746	76	17	0	Complete	100
Sample_C12C_k141_28687	35946	57	12	0	Complete	100
Sample_C12C_k141_76158	32030	48	19	1	Complete	100
Sample_C12C_k141_147995	129596	162	39	4	Complete	100
Sample_C12C_k141_240828	6133	9	4	0	Complete	100
Sample_C12C_k141_65053	96292	136	35	4	High-quality	100
Sample_C12C_k141_240834	41726	69	17	1	Complete	100
Sample_C13A_k141_95297_fragment_2	41372	59	12	2	High-quality	100
Sample_C13A_k141_120301	109866	140	24	6	Complete	100
Sample_C13A_k141_120298	42677	58	15	1	Complete	100
Sample_C13A_k141_97578	34612	61	16	1	Complete	100
Sample_C13A_k141_66497	12124	13	1	1	High-quality	100
Sample_C13A_k141_66772_fragment_1	102130	129	36	10	High-quality	100
Sample_C13A_k141_25906_fragment_1	42701	54	17	4	High-quality	100
Sample_C13A_k141_49554_fragment_3	54867	73	18	7	Complete	100
Sample_C13A_k141_49554_fragment_4	48153	50	4	4	High-quality	100
Sample_C13B_k141_80093_fragment_1	59577	74	15	13	Complete	100
Sample_C13B_k141_119965	24145	32	3	1	Complete	100
Sample_C13B_k141_103599	34230	58	17	0	Complete	100
Sample_C13B_k141_119964	159204	254	37	4	Complete	100
Sample_C13B_k141_68602_fragment_1	64561	87	13	8	Complete	100
Sample_C13B_k141_119983	6137	9	3	0	Complete	100

Sample_C13B_k141_7191	56424	86	26	1	Complete	100
Sample_C13C_k141_89411_fragment_1	41397	46	5	0	High-quality	100
Sample_C13C_k141_304_fragment_1	43114	73	15	1	High-quality	100
Sample_C13C_k141_111388	38589	56	22	0	Complete	100
Sample_C13C_k141_34796	39612	59	20	2	High-quality	100
Sample_C13C_k141_33538	54703	59	22	7	High-quality	100
Sample_C13C_k141_11694	16581	21	7	0	Complete	100
Sample_C13C_k141_111407	5204	8	7	0	Complete	100
Sample_C13C_k141_91394_fragment_1	72142	104	32	8	Complete	100
Sample_C13C_k141_70683_fragment_2	19465	29	2	0	High-quality	100
Sample_C13C_k141_91543	36652	61	21	0	Complete	100
Sample_C14A_k141_22873_fragment_1	39508	51	16	2	High-quality	100
Sample_C14A_k141_231873	6160	6	3	0	High-quality	100
Sample_C14A_k141_238943	43473	74	15	1	Complete	100
Sample_C14A_k141_238938	83441	113	15	2	Complete	100
Sample_C14A_k141_238936	78012	105	10	1	Complete	100
Sample_C14A_k141_32371_fragment_1	60806	83	23	6	High-quality	100
Sample_C14A_k141_13565	41967	73	19	0	Complete	100
Sample_C14A_k141_192469_fragment_3	47070	47	9	4	High-quality	100
Sample_C14A_k141_238946	95900	97	10	6	Complete	100
Sample_C14B_k141_69085_fragment_1	42635	56	17	6	High-quality	100
Sample_C14B_k141_79278_fragment_3	68896	100	30	6	Complete	100
Sample_C14B_k141_35432_fragment_2	41781	59	23	1	High-quality	100
Sample_C14B_k141_107258_fragment_1	39643	63	18	3	High-quality	100
Sample_C14B_k141_94559_fragment_2	36288	55	19	1	High-quality	100
Sample_C14B_k141_13475_fragment_1	59382	89	21	6	High-quality	100
Sample_C14C_k141_155654	43803	51	8	1	Complete	100
Sample_C14C_k141_154923_fragment_1	38363	51	24	3	High-quality	100
Sample_C14C_k141_141926	133546	249	19	13	High-quality	100
Sample_C14C_k141_40329	45814	70	18	7	High-quality	100
Sample_C14C_k141_189198	51945	76	21	1	Complete	100
Sample_C14C_k141_18812	44329	76	24	0	Complete	100
Sample_C14C_k141_136341	85763	145	17	1	Complete	100
Sample_C1A_k141_263133	181852	308	26	3	Complete	100
Sample_C1B_k141_124140	42132	69	17	0	Complete	100
Sample_C1B_k141_219692	76982	125	20	3	Complete	100
Sample_C1B_k141_2037	38479	55	13	1	High-quality	100
Sample_C1B_k141_144290	378307	552	49	20	Complete	100
Sample_C1B_k141_35778	80747	130	20	2	Complete	100

Sample_C1B_k141_204372	124793	152	32	8	Complete	100
Sample_C1B_k141_280959	100901	172	19	2	Complete	100
Sample_C1B_k141_280967	49864	78	24	1	Complete	100
Sample_C1C_k141_102302	51597	56	12	6	High-quality	100
Sample_C1C_k141_137851_fragment_1	39197	51	20	0	High-quality	100
Sample_C1C_k141_69794_fragment_1	53181	84	19	1	High-quality	100
Sample_C1C_k141_19685	38911	55	18	3	High-quality	100
Sample_C1C_k141_117218	73613	101	27	2	Complete	100
Sample_C1C_k141_131190_fragment_1	34707	59	14	0	High-quality	100
Sample_C2A_k141_22896_fragment_4	53055	58	25	3	High-quality	100
Sample_C2A_k141_212200	44752	47	15	5	High-quality	100
Sample_C2A_k141_330287	42662	63	15	1	Complete	100
Sample_C2A_k141_282625	46157	76	19	0	Complete	100
Sample_C2A_k141_41629_fragment_2	110362	175	27	1	High-quality	100
Sample_C2A_k141_199800_fragment_3	56200	82	22	5	Complete	100
Sample_C2A_k141_168470	45886	80	22	3	High-quality	100
Sample_C2A_k141_103709	41090	56	6	0	Complete	100
Sample_C2A_k141_105047	41880	60	16	6	Complete	100
Sample_C2A_k141_26924	5558	9	6	0	High-quality	100
Sample_C2A_k141_201647	54482	78	33	3	High-quality	100
Sample_C2A_k141_32930	42445	82	15	1	Complete	100
Sample_C2A_k141_236347	39712	63	12	2	Complete	100
Sample_C2A_k141_330290	61658	100	20	0	Complete	100
Sample_C2A_k141_268493_fragment_1	74029	103	22	2	High-quality	100
Sample_C2B_k141_346641	41369	47	9	0	Complete	100
Sample_C2B_k141_213011	75580	122	19	5	Complete	100
Sample_C2B_k141_52461	44639	72	16	1	Complete	100
Sample_C2B_k141_161172	14335	20	4	0	High-quality	100
Sample_C2B_k141_184423_fragment_1	57841	85	17	6	High-quality	100
Sample_C2B_k141_108374	39613	67	13	0	High-quality	100
Sample_C2B_k141_346631	55326	68	9	1	Complete	100
Sample_C2B_k141_346642	38155	65	19	0	Complete	100
Sample_C2B_k141_346647	29904	45	11	1	Complete	100
Sample_C2B_k141_70119	42626	77	17	0	Complete	100
Sample_C2B_k141_346650	46558	79	19	2	Complete	100
Sample_C2B_k141_102462	46356	72	12	1	High-quality	100
Sample_C2B_k141_279991_fragment_1	40601	58	16	2	High-quality	100
Sample_C2B_k141_107592	35992	52	12	3	High-quality	100
Sample_C2B_k141_183031	79559	127	45	0	High-quality	100

Sample_C2B_k141_216423	11008	18	1	1	High-quality	100
Sample_C2B_k141_82474	40416	52	9	0	Complete	100
Sample_C2B_k141_346630	136105	171	29	3	Complete	100
Sample_C2B_k141_346632	34851	61	19	0	Complete	100
Sample_C2B_k141_346639	59912	91	15	0	Complete	100
Sample_C2C_k141_233477	148264	189	25	4	Complete	100
Sample_C2C_k141_221907	46956	66	21	2	High-quality	100
Sample_C2C_k141_186502	57210	101	22	1	Complete	100
Sample_C2C_k141_276456	76601	94	19	0	High-quality	100
Sample_C2C_k141_222147_fragment_2	56268	65	24	1	High-quality	100
Sample_C2C_k141_281589	90618	108	9	8	Complete	100
Sample_C2C_k141_37389	81732	121	28	4	High-quality	100
Sample_C2C_k141_254616	38239	63	12	1	High-quality	100
Sample_C3A_k141_77400	47790	65	27	1	High-quality	100
Sample_C3A_k141_211621_fragment_2	14025	18	1	2	High-quality	100
Sample_C3A_k141_212548_fragment_1	46986	65	15	0	High-quality	100
Sample_C3A_k141_259454	87878	114	14	2	Complete	100
Sample_C3A_k141_238617	134086	182	33	3	High-quality	100
Sample_C3A_k141_259460	40011	47	44	0	Complete	100
Sample_C3A_k141_239223	13719	24	2	0	High-quality	100
Sample_C3A_k141_42808	10974	17	1	1	High-quality	100
Sample_C3A_k141_259461	42138	54	10	2	Complete	100
Sample_C3A_k141_221007	43734	81	17	1	High-quality	100
Sample_C3A_k141_170867_fragment_1	42447	58	38	2	High-quality	100
Sample_C3B_k141_204396	44386	56	31	3	High-quality	100
Sample_C3B_k141_60779_fragment_1	36429	49	18	0	High-quality	100
Sample_C3B_k141_44649_fragment_1	37688	53	32	0	High-quality	100
Sample_C3B_k141_219195	70228	117	22	6	High-quality	100
Sample_C3B_k141_92033	17164	21	6	0	Complete	100
Sample_C3C_k141_167078	43134	68	6	3	High-quality	100
Sample_C3C_k141_15477_fragment_1	58801	85	22	5	High-quality	100
Sample_C3C_k141_191120_fragment_1	90364	140	28	8	High-quality	100
Sample_C3C_k141_88524_fragment_1	133838	199	25	0	High-quality	100
Sample_C3C_k141_41389_fragment_1	40353	56	22	0	High-quality	100
Sample_C3C_k141_253099	59463	89	32	1	Complete	100
Sample_C3C_k141_266494_fragment_1	47803	69	15	2	High-quality	100
Sample_C3C_k141_269821	63794	67	6	0	Complete	100
Sample_C3C_k141_268380	62333	82	32	1	Complete	100
Sample_C3C_k141_143848	104110	158	28	1	High-quality	100

Sample_C3C_k141_127174_fragment_10	42377	81	21	0	High-quality	100
Sample_C4A_k141_174053	45447	60	13	1	Complete	100
Sample_C4A_k141_54750	57067	95	15	2	Complete	100
Sample_C4A_k141_262747	5177	8	5	0	Complete	100
Sample_C4A_k141_57994	45265	71	15	1	Complete	100
Sample_C4A_k141_76338	45255	82	16	0	High-quality	100
Sample_C4A_k141_262732	40447	71	15	0	Complete	100
Sample_C4A_k141_30076_fragment_1	71989	87	23	10	High-quality	100
Sample_C4B_k141_230636_fragment_2	59487	100	22	2	High-quality	100
Sample_C4B_k141_140102_fragment_1	48083	79	19	1	High-quality	100
Sample_C4B_k141_110919	62399	68	26	3	High-quality	100
Sample_C4B_k141_8391	85842	114	17	2	Complete	100
Sample_C4B_k141_200226	61611	107	23	0	Complete	100
Sample_C4B_k141_68027_fragment_1	61618	84	20	6	Complete	100
Sample_C4B_k141_68679	5225	8	1	0	High-quality	100
Sample_C4B_k141_261371_fragment_2	49186	60	3	1	High-quality	100
Sample_C4B_k141_23345	17152	27	7	0	Complete	100
Sample_C4B_k141_167444	59719	100	26	1	High-quality	100
Sample_C4C_k141_485_fragment_1	12973	15	1	6	High-quality	100
Sample_C4C_k141_266359	44291	72	17	0	Complete	100
Sample_C4C_k141_10535	42984	65	14	2	Complete	100
Sample_C4C_k141_57686_fragment_1	64428	62	23	8	Complete	100
Sample_C4C_k141_185454	153172	211	37	4	High-quality	100
Sample_C4C_k141_169692	46180	76	17	0	Complete	100
Sample_C4C_k141_266367	37940	53	24	0	Complete	100
Sample_C4C_k141_225732	79911	138	18	5	Complete	100
Sample_C4C_k141_223197	39203	60	16	1	Complete	100
Sample_C4C_k141_266364	118006	151	38	3	Complete	100
Sample_C4C_k141_93468_fragment_2	71308	112	23	8	High-quality	100
Sample_C4C_k141_231036	66567	89	24	3	High-quality	100
Sample_C4C_k141_66649	51193	59	3	6	Complete	100
Sample_C5A_k141_85212	44190	66	19	4	Complete	100
Sample_C5A_k141_224321	42777	67	21	3	High-quality	100
Sample_C5A_k141_239468	113654	135	13	4	High-quality	100
Sample_C5A_k141_291115	6329	8	2	0	Complete	100
Sample_C5A_k141_291098	86484	116	14	2	Complete	100
Sample_C5A_k141_291099	159981	168	14	4	Complete	100
Sample_C5A_k141_271588	20243	34	4	1	Complete	100
Sample_C5A_k141_151990_fragment_1	46098	51	19	3	High-quality	100

Sample_C5C_k141_109556	34866	43	23	0	High-quality	100
Sample_C5C_k141_30151_fragment_1	14908	15	5	0	High-quality	100
Sample_C5C_k141_120119	37723	57	13	3	Complete	100
Sample_C5C_k141_52268_fragment_2	35369	52	17	1	High-quality	100
Sample_C6A_k141_19224	6980	10	3	0	High-quality	100
Sample_C6A_k141_36275_fragment_2	39904	59	22	0	High-quality	100
Sample_C6A_k141_77660_fragment_1	58477	64	23	5	High-quality	100
Sample_C6A_k141_149074	85852	108	20	7	High-quality	100
Sample_C6A_k141_114932	35246	65	19	0	Complete	100
Sample_C6A_k141_212784_fragment_1	83771	113	16	8	High-quality	100
Sample_C6B_k141_50026_fragment_1	37137	45	16	0	High-quality	100
Sample_C6B_k141_1610	38497	48	6	0	Complete	100
Sample_C6B_k141_60682	36195	60	24	0	High-quality	100
Sample_C6B_k141_218088	29579	45	15	0	Complete	100
Sample_C6B_k141_125048_fragment_1	45163	60	16	6	High-quality	100
Sample_C6B_k141_146065	54425	77	22	10	Complete	100
Sample_C6B_k141_75790_fragment_2	42682	61	16	2	High-quality	100
Sample_C6B_k141_103062	84065	136	31	2	Complete	100
Sample_C6C_k141_95107	66176	62	13	10	High-quality	100
Sample_C6C_k141_165146_fragment_1	89957	126	29	5	High-quality	100
Sample_C6C_k141_170257	159273	210	20	4	Complete	100
Sample_C6C_k141_122860_fragment_1	79129	108	23	5	Complete	100
Sample_C6C_k141_244630	33798	54	16	0	Complete	100
Sample_C6C_k141_76681	6338	5	1	0	High-quality	100
Sample_C6C_k141_166530_fragment_3	37889	51	20	1	High-quality	100
Sample_C6C_k141_86800_fragment_1	38804	52	24	1	High-quality	100
Sample_C7A_k141_111088	101152	176	14	1	Complete	100
Sample_C7A_k141_61483	61518	90	36	1	High-quality	100
Sample_C7A_k141_5331_fragment_2	56659	67	21	4	High-quality	100
Sample_C7A_k141_120863_fragment_1	85668	116	39	4	High-quality	100
Sample_C7A_k141_1680	122169	152	38	3	High-quality	100
Sample_C7B_k141_184777	41360	78	18	1	Complete	100
Sample_C7B_k141_55721_fragment_4	63188	107	21	1	High-quality	100
Sample_C7B_k141_106634	5904	8	3	0	Complete	100
Sample_C7B_k141_184790	6184	6	4	0	Complete	100
Sample_C7C_k141_45241	17269	22	0	3	High-quality	100
Sample_C8A_k141_182399	176691	212	31	4	Complete	100
Sample_C8A_k141_161734	78638	79	30	9	High-quality	100
Sample_C8A_k141_60392	77484	122	19	3	Complete	100

Sample_C8A_k141_161111	100060	172	20	1	High-quality	100
Sample_C8A_k141_84594	18172	29	7	0	Complete	100
Sample_C8B_k141_22061_fragment_1	71257	96	17	5	High-quality	100
Sample_C8B_k141_104270_fragment_5	42842	68	24	2	High-quality	100
Sample_C8B_k141_127738	10345	14	1	0	High-quality	100
Sample_C8B_k141_207777	38304	51	6	1	Complete	100
Sample_C8B_k141_207170_fragment_1	137031	194	64	8	Complete	100
Sample_C8B_k141_166111	20620	35	3	1	High-quality	100
Sample_C8B_k141_146461	33517	48	13	0	Complete	100
Sample_C8B_k141_187910	42675	69	17	0	Complete	100
Sample_C8B_k141_175861	59840	91	22	2	High-quality	100
Sample_C8B_k141_109776	41005	46	4	1	High-quality	100
Sample_C8C_k141_119772_fragment_3	65507	106	28	0	High-quality	100
Sample_C8C_k141_178248	6187	7	4	0	Complete	100
Sample_C8C_k141_137029	43777	68	15	1	High-quality	100
Sample_C8C_k141_178229	40181	53	10	0	Complete	100
Sample_C8C_k141_57654_fragment_1	51822	78	18	9	High-quality	100
Sample_C8C_k141_7093_fragment_2	74846	111	42	5	High-quality	100
Sample_C8C_k141_54801_fragment_2	85197	135	30	6	High-quality	100
Sample_C8C_k141_50188	177939	264	26	4	High-quality	100
Sample_C8C_k141_178231	6387	6	3	0	Complete	100
Sample_C8C_k141_45546	35716	57	17	0	Complete	100
Sample_C8C_k141_145064	44618	66	19	3	High-quality	100
Sample_C8C_k141_172345	99200	116	19	3	Complete	100
Sample_C8C_k141_76497	55840	60	21	1	High-quality	100
Sample_C8C_k141_33206_fragment_4	58170	71	23	6	High-quality	100
Sample_C8C_k141_54847_fragment_1	61912	87	13	4	High-quality	100
Sample_C8C_k141_21129_fragment_2	117228	149	27	13	High-quality	100
Sample_C9A_k141_47763_fragment_1	45159	51	24	0	High-quality	100
Sample_C9A_k141_15424_fragment_1	63916	97	32	1	High-quality	100
Sample_C9A_k141_117382	44787	63	17	4	High-quality	100
Sample_C9A_k141_9399	41784	64	24	3	High-quality	100
Sample_C9A_k141_135364	119967	166	29	2	Complete	100
Sample_C9A_k141_128816	41058	63	19	2	Complete	100
Sample_C9A_k141_135370	5795	9	5	0	Complete	100
Sample_C9A_k141_29425	79252	127	17	1	High-quality	100
Sample_C9A_k141_135366	47336	61	12	0	Complete	100
Sample_C9B_k141_64969	46951	65	27	1	High-quality	100
Sample_C9B_k141_41435	8159	12	1	0	Complete	100

Sample_C9B_k141_107988_fragment_1	65728	88	16	0	High-quality	100
Sample_C9B_k141_55734_fragment_1	68371	95	26	2	High-quality	100
Sample_C9C_k141_88091	38368	57	17	0	High-quality	100
Sample_C9C_k141_56643	48292	61	20	0	Complete	100
Sample_C9C_k141_103559	36543	52	24	0	Complete	100
Sample_C9C_k141_49776	48027	67	30	1	High-quality	100
Sample_C9C_k141_73648	24854	31	3	1	Complete	100
Sample_C9C_k141_65996_fragment_1	38235	45	4	2	High-quality	100
Sample_C9C_k141_74396_fragment_2	56197	80	21	4	Complete	100
Sample_C9C_k141_77261	5418	10	6	0	Complete	100
Sample_C9C_k141_72908	75127	70	7	5	High-quality	100
Sample_T10A_k141_121564_fragment_1	53154	72	24	3	High-quality	100
Sample_T10A_k141_44794	16042	20	7	0	Complete	100
Sample_T10A_k141_184450	154143	199	15	4	Complete	100
Sample_T10A_k141_140046	111113	122	40	4	Complete	100
Sample_T10A_k141_45245	7038	12	0	0	High-quality	100
Sample_T10A_k141_19038_fragment_1	44832	63	18	0	High-quality	100
Sample_T10A_k141_94094	32394	48	17	1	Complete	100
Sample_T10A_k141_9994	44675	59	11	1	Complete	100
Sample_T10A_k141_41253_fragment_3	38728	55	17	1	High-quality	100
Sample_T10A_k141_170565	102907	186	33	1	Complete	100
Sample_T10A_k141_51046	43994	66	14	1	High-quality	100
Sample_T10B_k141_186414	80141	125	30	3	Complete	100
Sample_T10B_k141_84959	7149	11	1	1	High-quality	100
Sample_T10B_k141_19562_fragment_2	57566	84	20	3	High-quality	100
Sample_T10B_k141_21815_fragment_2	67703	94	35	4	High-quality	100
Sample_T10B_k141_180520	43565	71	21	0	High-quality	100
Sample_T10B_k141_49255	29741	39	6	2	High-quality	100
Sample_T10B_k141_183845_fragment_1	71051	98	21	3	High-quality	100
Sample_T10B_k141_52931	74536	88	5	9	Complete	100
Sample_T10B_k141_22023	78559	118	22	2	Complete	100
Sample_T10C_k141_89312	45680	77	18	0	Complete	100
Sample_T10C_k141_83426	96707	96	9	0	High-quality	100
Sample_T10C_k141_89310	45781	74	21	0	Complete	100
Sample_T11A_k141_40763	55643	94	17	1	Complete	100
Sample_T11A_k141_120974_fragment_2	42140	51	28	1	High-quality	100
Sample_T11A_k141_92220	61066	97	26	1	High-quality	100
Sample_T11A_k141_82524	9771	11	1	0	High-quality	100
Sample_T11A_k141_153661	42577	55	10	0	Complete	100

Sample_T11A_k141_149653_fragment_1	54906	65	13	2	High-quality	100
Sample_T11A_k141_66298	60342	102	18	4	Complete	100
Sample_T11A_k141_31785_fragment_1	43807	67	28	1	High-quality	100
Sample_T11A_k141_124272	66576	101	35	3	High-quality	100
Sample_T11A_k141_153664	6109	10	3	0	Complete	100
Sample_T11A_k141_1173_fragment_1	45698	66	25	3	High-quality	100
Sample_T11B_k141_130950_fragment_1	68724	95	29	4	High-quality	100
Sample_T11B_k141_39705_fragment_1	88963	152	38	0	High-quality	100
Sample_T11B_k141_8353_fragment_3	126544	151	11	18	High-quality	100
Sample_T11B_k141_118076_fragment_2	63904	76	7	0	High-quality	100
Sample_T11C_k141_49996_fragment_1	75264	124	18	7	High-quality	100
Sample_T11C_k141_95529_fragment_1	37747	54	9	0	High-quality	100
Sample_T11C_k141_58894	35568	52	15	1	High-quality	100
Sample_T11C_k141_139428_fragment_1	83811	126	45	2	High-quality	100
Sample_T11C_k141_10686	18548	24	3	1	High-quality	100
Sample_T11C_k141_167141	99661	169	13	2	Complete	100
Sample_T11C_k141_56104_fragment_2	41627	60	13	3	High-quality	100
Sample_T11C_k141_11538	84382	112	9	3	High-quality	100
Sample_T12A_k141_2183	53781	75	18	1	High-quality	100
Sample_T12A_k141_62034	17536	21	6	0	Complete	100
Sample_T12A_k141_344756	57816	94	20	0	Complete	100
Sample_T12A_k141_175439	42480	79	14	0	Complete	100
Sample_T12A_k141_138919_fragment_1	36162	49	16	1	High-quality	100
Sample_T12A_k141_188418	99752	98	14	1	Complete	100
Sample_T12A_k141_344769	55877	99	14	2	Complete	100
Sample_T12A_k141_43435_fragment_1	50960	57	14	4	High-quality	100
Sample_T12A_k141_329582	6626	6	3	0	High-quality	100
Sample_T12A_k141_276127	46974	83	27	1	Complete	100
Sample_T12A_k141_344761	35702	65	13	0	Complete	100
Sample_T12A_k141_183833_fragment_1	73577	76	23	11	High-quality	100
Sample_T12A_k141_2118_fragment_2	54853	65	23	2	High-quality	100
Sample_T12A_k141_250292	44404	61	21	2	High-quality	100
Sample_T12A_k141_79577	40888	70	19	2	Complete	100
Sample_T12A_k141_204622	71679	93	25	2	High-quality	100
Sample_T12A_k141_88914	45815	61	7	3	High-quality	100
Sample_T12A_k141_207812	46109	85	22	0	Complete	100
Sample_T12A_k141_344760	42299	44	9	1	Complete	100
Sample_T12A_k141_344772	5171	9	6	0	Complete	100
Sample_T12B_k141_174439	104554	162	27	1	Complete	100

Sample_T12B_k141_134060_fragment_1	39624	56	14	2	High-quality	100
Sample_T12B_k141_60174	41905	73	17	1	Complete	100
Sample_T12B_k141_189374	108693	132	27	2	Complete	100
Sample_T12B_k141_189375	6392	6	3	0	Complete	100
Sample_T12C_k141_186893	7265	9	3	0	Complete	100
Sample_T12C_k141_61294_fragment_1	42398	62	18	2	High-quality	100
Sample_T12C_k141_27078	45435	52	3	2	High-quality	100
Sample_T12C_k141_1331	57490	97	24	2	Complete	100
Sample_T12C_k141_196407	88403	147	18	3	Complete	100
Sample_T12C_k141_196416	58890	82	27	1	Complete	100
Sample_T12C_k141_14380_fragment_2	40059	59	18	0	High-quality	100
Sample_T13A_k141_132877	79470	115	18	0	Complete	100
Sample_T13A_k141_124272	18527	23	8	0	High-quality	100
Sample_T13A_k141_132876	43822	56	49	0	Complete	100
Sample_T13A_k141_120565	68540	94	35	1	High-quality	100
Sample_T13A_k141_132883	46335	55	9	0	Complete	100
Sample_T13B_k141_184398	45760	71	11	1	Complete	100
Sample_T13B_k141_35777_fragment_1	35924	53	17	3	High-quality	100
Sample_T13B_k141_72989	11152	12	1	1	High-quality	100
Sample_T13B_k141_74963_fragment_2	149251	210	29	5	High-quality	100
Sample_T13B_k141_131879_fragment_2	38587	53	23	0	High-quality	100
Sample_T13B_k141_130801_fragment_1	41562	66	29	0	High-quality	100
Sample_T13B_k141_24824	17815	27	8	0	High-quality	100
Sample_T13B_k141_54216	31042	48	13	1	High-quality	100
Sample_T13B_k141_46918	17449	26	9	0	Complete	100
Sample_T13C_k141_124423_fragment_2	55985	84	29	1	High-quality	100
Sample_T13C_k141_145369	5957	9	4	0	Complete	100
Sample_T13C_k141_23189_fragment_6	37801	61	20	0	High-quality	100
Sample_T13C_k141_145370	40521	70	21	0	Complete	100
Sample_T13C_k141_15970_fragment_3	50065	58	19	7	High-quality	100
Sample_T13C_k141_123276	43036	60	13	1	High-quality	100
Sample_T13C_k141_145371	43727	71	18	0	Complete	100
Sample_T14A_k141_35971	45273	70	21	0	Complete	100
Sample_T14A_k141_242962	36007	75	11	1	Complete	100
Sample_T14A_k141_189019_fragment_1	49324	71	14	10	Complete	100
Sample_T14A_k141_242969	61636	113	24	0	Complete	100
Sample_T14A_k141_160595	10745	13	1	0	High-quality	100
Sample_T14A_k141_15934_fragment_3	58509	90	19	4	High-quality	100
Sample_T14A_k141_94084_fragment_6	40520	60	14	4	High-quality	100

Sample_T14B_k141_102073	77764	98	22	17	Complete	100
Sample_T14B_k141_177008	73333	101	24	5	High-quality	100
Sample_T14B_k141_35849_fragment_3	46229	68	19	1	High-quality	100
Sample_T14B_k141_78918	37926	57	12	3	High-quality	100
Sample_T14B_k141_169946_fragment_1	70750	89	21	19	Complete	100
Sample_T14C_k141_37388	19047	29	8	0	Complete	100
Sample_T14C_k141_18709_fragment_1	47300	63	27	0	High-quality	100
Sample_T14C_k141_24865_fragment_1	34547	42	15	0	High-quality	100
Sample_T14C_k141_165958	38008	57	13	3	Complete	100
Sample_T14C_k141_70264_fragment_1	42539	45	4	0	High-quality	100
Sample_T14C_k141_95605_fragment_5	7883	13	0	0	High-quality	100
Sample_T15A_k141_165274	38706	62	16	4	Complete	100
Sample_T15A_k141_167021_fragment_1	66821	94	29	4	Complete	100
Sample_T15A_k141_99106_fragment_1	45408	61	27	3	High-quality	100
Sample_T15A_k141_109627	242157	272	2	20	High-quality	100
Sample_T15A_k141_168442_fragment_3	132619	179	8	12	High-quality	100
Sample_T15B_k141_121042_fragment_1	43589	67	20	2	High-quality	100
Sample_T15B_k141_35398_fragment_1	45458	43	7	2	High-quality	100
Sample_T15B_k141_114413_fragment_1	62736	98	32	2	High-quality	100
Sample_T15B_k141_151148	99163	126	14	0	Complete	100
Sample_T15B_k141_16979	104568	159	30	1	Complete	100
Sample_T15C_k141_124202_fragment_2	41578	57	14	1	High-quality	100
Sample_T15C_k141_71007	53245	75	19	1	High-quality	100
Sample_T15C_k141_70091	75817	128	19	2	Complete	100
Sample_T15C_k141_52346_fragment_1	47386	86	22	4	High-quality	100
Sample_T15C_k141_49043_fragment_5	65265	80	3	7	High-quality	100
Sample_T15C_k141_78884_fragment_2	57886	79	18	2	High-quality	100
Sample_T15C_k141_32637_fragment_1	167095	213	4	12	High-quality	100
Sample_T15C_k141_150669	99639	94	11	0	Complete	100
Sample_T16A_k141_105581	13355	18	4	0	Complete	100
Sample_T16A_k141_28010_fragment_2	38418	43	32	3	High-quality	100
Sample_T16B_k141_6566_fragment_1	46553	65	35	1	High-quality	100
Sample_T16C_k141_39448_fragment_1	89152	123	22	5	High-quality	100
Sample_T16C_k141_17052	5785	8	5	2	High-quality	100
Sample_T16C_k141_54509	13940	23	1	0	High-quality	100
Sample_T16C_k141_91113	12383	19	0	1	High-quality	100
Sample_T16C_k141_136011_fragment_2	41139	64	16	1	High-quality	100
Sample_T16C_k141_46649_fragment_1	44672	49	6	2	High-quality	100
Sample_T16C_k141_145047	36946	50	23	1	High-quality	100

Sample_T16C_k141_146718	188959	326	26	3	Complete	100
Sample_T16C_k141_8526_fragment_1	81706	102	32	19	Complete	100
Sample_T16C_k141_110317_fragment_5	47992	54	17	6	High-quality	100
Sample_T16C_k141_47250_fragment_1	43583	57	14	6	Complete	100
Sample_T17A_k141_20666	37877	56	13	1	Complete	100
Sample_T17A_k141_28935_fragment_1	45746	54	26	3	High-quality	100
Sample_T17A_k141_23777_fragment_2	49485	53	4	4	High-quality	100
Sample_T17B_k141_37912	39162	51	48	0	Complete	100
Sample_T17C_k141_23948_fragment_1	41286	57	29	0	High-quality	100
Sample_T17C_k141_39423	45847	77	20	1	Complete	100
Sample_T17C_k141_7401_fragment_1	59772	83	20	3	High-quality	100
Sample_T17C_k141_39428	43693	58	45	0	Complete	100
Sample_T18A_k141_200728_fragment_2	49787	65	18	7	Complete	100
Sample_T18A_k141_204594	5510	10	6	0	Complete	100
Sample_T18A_k141_143758_fragment_1	43750	60	24	1	High-quality	100
Sample_T18A_k141_87101	77470	130	15	2	Complete	100
Sample_T18B_k141_91132	46081	67	25	4	High-quality	100
Sample_T18B_k141_63934	56562	101	17	2	Complete	100
Sample_T18C_k141_63458	43951	67	16	1	Complete	100
Sample_T18C_k141_145428	62410	94	19	0	High-quality	100
Sample_T18C_k141_70431	49831	77	24	0	Complete	100
Sample_T18C_k141_122829	40271	48	6	0	High-quality	100
Sample_T18C_k141_154253	101097	154	9	2	Complete	100
Sample_T18C_k141_101925	43234	51	7	0	High-quality	100
Sample_T1A_k141_3534_fragment_1	42029	48	30	1	High-quality	100
Sample_T1A_k141_23452	41372	66	45	0	Complete	100
Sample_T1A_k141_25707_fragment_1	39409	55	31	1	High-quality	100
Sample_T1A_k141_14441	99864	119	21	2	High-quality	100
Sample_T1B_k141_44053	55902	100	22	4	Complete	100
Sample_T1B_k141_44049	46662	69	21	1	Complete	100
Sample_T1C_k141_71842	7079	11	1	0	Complete	100
Sample_T1C_k141_32501	108547	134	9	2	Complete	100
Sample_T1C_k141_34693_fragment_1	99906	138	13	8	Complete	100
Sample_T2A_k141_115058	42501	69	19	0	Complete	100
Sample_T2A_k141_279074	48294	83	48	0	Complete	100
Sample_T2A_k141_226904	52642	63	13	2	High-quality	100
Sample_T2A_k141_114442_fragment_1	167295	248	31	5	High-quality	100
Sample_T2B_k141_99948	62684	84	8	8	High-quality	100
Sample_T2B_k141_177025	50595	74	21	3	High-quality	100

Sample_T2B_k141_70448	13767	16	4	0	High-quality	100
Sample_T2B_k141_320851	5965	6	1	0	Complete	100
Sample_T2B_k141_71618	43302	56	11	1	Complete	100
Sample_T2B_k141_176056	39259	51	14	1	High-quality	100
Sample_T2B_k141_76478	43864	69	15	0	High-quality	100
Sample_T2B_k141_320850	6394	7	3	0	Complete	100
Sample_T2B_k141_262111	146778	188	6	15	High-quality	100
Sample_T2B_k141_235571	53688	85	29	3	High-quality	100
Sample_T2B_k141_320848	5667	8	2	0	Complete	100
Sample_T2C_k141_277029	6326	7	3	0	Complete	100
Sample_T2C_k141_179798_fragment_1	40818	64	26	1	High-quality	100
Sample_T2C_k141_172890_fragment_1	60596	67	21	13	Complete	100
Sample_T2C_k141_157000	42295	66	24	0	Complete	100
Sample_T2C_k141_93097	36221	60	8	1	Complete	100
Sample_T2C_k141_9745_fragment_2	53336	71	27	4	Complete	100
Sample_T2C_k141_65371	152025	210	30	7	High-quality	100
Sample_T2C_k141_52521	40716	63	11	2	High-quality	100
Sample_T2C_k141_80902_fragment_1	39208	58	14	0	High-quality	100
Sample_T2C_k141_57555	14684	16	0	3	High-quality	100
Sample_T2C_k141_141009_fragment_2	59659	70	15	11	Complete	100
Sample_T2C_k141_196660	38321	69	15	1	Complete	100
Sample_T2C_k141_277020	41329	53	6	0	Complete	100
Sample_T2C_k141_86598	5807	8	5	0	Complete	100
Sample_T2C_k141_277036	5174	9	6	0	Complete	100
Sample_T3A_k141_107537	38712	47	6	0	Complete	100
Sample_T3A_k141_83896_fragment_1	58092	68	35	8	High-quality	100
Sample_T3A_k141_113137	6202	7	3	0	Complete	100
Sample_T3A_k141_51574_fragment_2	75479	96	12	8	High-quality	100
Sample_T3A_k141_37854	40630	58	15	1	High-quality	100
Sample_T3A_k141_84551_fragment_1	14356	18	0	1	High-quality	100
Sample_T3A_k141_35394	34236	46	12	0	Complete	100
Sample_T3A_k141_47264	43762	56	29	3	High-quality	100
Sample_T3B_k141_102008	85012	95	33	4	High-quality	100
Sample_T3B_k141_186515	38827	55	16	0	Complete	100
Sample_T3B_k141_11572_fragment_1	38973	59	15	1	High-quality	100
Sample_T3B_k141_42389	19277	30	8	0	Complete	100
Sample_T3B_k141_122130	41945	61	10	0	Complete	100
Sample_T3B_k141_27568	17555	24	6	0	High-quality	100
Sample_T3B_k141_3850_fragment_2	59140	86	18	6	High-quality	100

Sample_T3B_k141_157090_fragment_1	51458	73	19	2	High-quality	100
Sample_T3C_k141_121932_fragment_2	61892	70	24	2	High-quality	100
Sample_T3C_k141_43810_fragment_1	94606	140	31	5	High-quality	100
Sample_T3C_k141_141857	5399	9	5	0	Complete	100
Sample_T3C_k141_113662_fragment_2	52791	73	29	5	Complete	100
Sample_T3C_k141_102340	89753	149	31	0	High-quality	100
Sample_T3C_k141_32711	6198	7	4	0	Complete	100
Sample_T3C_k141_141861	5567	8	6	0	Complete	100
Sample_T3C_k141_90385_fragment_5	65885	60	14	4	High-quality	100
Sample_T3C_k141_17486	60266	91	29	0	Complete	100
Sample_T3C_k141_122631	6143	9	3	0	Complete	100
Sample_T4A_k141_76842_fragment_2	44097	54	19	6	Complete	100
Sample_T4A_k141_91435_fragment_1	42098	51	6	5	High-quality	100
Sample_T4A_k141_94762	61308	69	23	4	High-quality	100
Sample_T4A_k141_99972	70156	114	30	6	High-quality	100
Sample_T4B_k141_285155	100352	170	17	1	High-quality	100
Sample_T4B_k141_337711_fragment_1	8648	14	0	0	High-quality	100
Sample_T4B_k141_373739	55360	99	18	0	Complete	100
Sample_T4B_k141_341131	30768	53	16	0	Complete	100
Sample_T4B_k141_322050_fragment_1	46700	75	18	3	High-quality	100
Sample_T4B_k141_56684	51288	77	19	4	High-quality	100
Sample_T4B_k141_276935_fragment_1	59120	97	21	3	High-quality	100
Sample_T4B_k141_42743	45739	69	20	0	Complete	100
Sample_T4B_k141_261292	45871	75	14	0	High-quality	100
Sample_T4B_k141_307184	107119	145	34	0	Complete	100
Sample_T4B_k141_317222	30499	55	10	1	Complete	100
Sample_T4B_k141_373742	42034	68	16	0	Complete	100
Sample_T4B_k141_64428	76810	136	15	4	Complete	100
Sample_T4B_k141_96162_fragment_1	50025	86	16	1	High-quality	100
Sample_T4B_k141_282736_fragment_1	72673	96	12	8	Complete	100
Sample_T4B_k141_224722_fragment_1	41616	60	11	1	High-quality	100
Sample_T4B_k141_188638	33721	56	15	1	Complete	100
Sample_T4B_k141_166765	42266	78	22	1	High-quality	100
Sample_T4B_k141_233505_fragment_2	39022	58	12	2	High-quality	100
Sample_T4C_k141_84305_fragment_3	43363	74	18	3	High-quality	100
Sample_T4C_k141_125422	68537	98	18	2	Complete	100
Sample_T5A_k141_48737	6537	7	4	0	High-quality	100
Sample_T5A_k141_46617	7177	8	1	1	High-quality	100
Sample_T5A_k141_17989_fragment_2	61410	85	27	7	High-quality	100

Sample_T5A_k141_57347_fragment_1	55308	75	30	3	High-quality	100
Sample_T5A_k141_23816	39428	52	20	0	Complete	100
Sample_T5A_k141_44639	49715	74	19	2	High-quality	100
Sample_T5A_k141_59308	36108	63	25	1	Complete	100
Sample_T5A_k141_25053_fragment_1	51279	66	19	6	High-quality	100
Sample_T5A_k141_9360_fragment_1	42092	52	17	1	High-quality	100
Sample_T5A_k141_18142_fragment_1	45966	69	30	2	High-quality	100
Sample_T5A_k141_7018	10331	13	1	0	High-quality	100
Sample_T5B_k141_59832_fragment_1	74603	88	12	8	High-quality	100
Sample_T5B_k141_82064	98978	158	17	1	Complete	100
Sample_T5B_k141_26295_fragment_1	62887	90	33	3	High-quality	100
Sample_T5B_k141_76397_fragment_1	74258	117	16	1	High-quality	100
Sample_T5B_k141_40521	39344	52	20	0	High-quality	100
Sample_T5B_k141_336	58973	90	19	4	High-quality	100
Sample_T5B_k141_46929_fragment_2	68154	65	23	4	High-quality	100
Sample_T5C_k141_198725	78730	115	15	0	Complete	100
Sample_T5C_k141_112401	43854	67	15	2	High-quality	100
Sample_T5C_k141_112999	41020	71	13	0	High-quality	100
Sample_T5C_k141_141839	37748	59	13	0	High-quality	100
Sample_T5C_k141_64876_fragment_2	65184	93	19	6	High-quality	100
Sample_T5C_k141_198735	28778	49	17	0	Complete	100
Sample_T5C_k141_198747	5027	9	6	0	Complete	100
Sample_T5C_k141_198722	44201	66	32	0	Complete	100
Sample_T5C_k141_164630_fragment_1	72165	96	22	9	High-quality	100
Sample_T5C_k141_198731	98537	159	19	1	Complete	100
Sample_T5C_k141_67198	42558	78	19	1	Complete	100
Sample_T5C_k141_176003	47725	75	19	5	Complete	100
Sample_T5C_k141_61733	46459	77	23	2	High-quality	100
Sample_T5C_k141_198727	35036	62	9	1	Complete	100
Sample_T5C_k141_198721	100678	102	11	1	Complete	100
Sample_T6A_k141_194395	35018	54	20	4	High-quality	100
Sample_T6A_k141_39810	67901	109	20	9	Complete	100
Sample_T6A_k141_114453_fragment_2	73960	70	2	8	High-quality	100
Sample_T6A_k141_56295	41559	64	25	0	High-quality	100
Sample_T6A_k141_5244	49987	53	22	4	High-quality	100
Sample_T6A_k141_137200	52250	70	15	2	High-quality	100
Sample_T6B_k141_202148	7622	15	1	0	Complete	100
Sample_T6B_k141_46601_fragment_1	37815	63	15	0	High-quality	100
Sample_T6B_k141_202142	210300	323	50	9	Complete	100

Sample_T6B_k141_37858_fragment_1	83638	97	11	12	High-quality	100
Sample_T6B_k141_7831_fragment_1	64590	78	3	1	High-quality	100
Sample_T6B_k141_144347_fragment_2	56479	89	23	0	High-quality	100
Sample_T6B_k141_48911_fragment_1	47656	54	25	1	High-quality	100
Sample_T6B_k141_21972	38276	64	9	1	Complete	100
Sample_T6C_k141_118693_fragment_1	55267	69	33	8	High-quality	100
Sample_T6C_k141_158503	46521	63	32	1	High-quality	100
Sample_T6C_k141_131908	113728	151	18	4	High-quality	100
Sample_T6C_k141_154820_fragment_1	46922	63	15	1	High-quality	100
Sample_T7A_k141_85587	58526	74	5	5	High-quality	100
Sample_T7A_k141_128969_fragment_2	71629	112	34	7	High-quality	100
Sample_T7A_k141_29771_fragment_1	71744	99	21	4	High-quality	100
Sample_T7A_k141_146475	38356	59	21	1	High-quality	100
Sample_T7A_k141_47247_fragment_1	33266	43	31	0	High-quality	100
Sample_T7A_k141_229003	5720	9	6	0	Complete	100
Sample_T7A_k141_7215_fragment_1	47755	59	38	4	High-quality	100
Sample_T7A_k141_229002	45966	79	21	0	Complete	100
Sample_T7A_k141_229007	35914	58	23	1	Complete	100
Sample_T7A_k141_229010	46002	70	16	0	Complete	100
Sample_T7A_k141_652	55004	97	47	0	Complete	100
Sample_T7A_k141_70750	170187	170	14	14	High-quality	100
Sample_T7A_k141_137586_fragment_1	36372	47	30	1	High-quality	100
Sample_T7A_k141_16138	7103	9	1	1	High-quality	100
Sample_T7A_k141_207986	64037	93	31	3	High-quality	100
Sample_T7B_k141_111110_fragment_1	35658	47	22	1	High-quality	100
Sample_T7B_k141_120797	53136	83	23	6	Complete	100
Sample_T7B_k141_124389_fragment_1	59888	101	20	2	High-quality	100
Sample_T7B_k141_45182_fragment_1	39920	48	7	0	High-quality	100
Sample_T7C_k141_162616	6577	8	3	0	Complete	100
Sample_T8A_k141_102946	94324	93	9	1	High-quality	100
Sample_T8A_k141_140150	72032	112	18	1	High-quality	100
Sample_T8A_k141_175688	44293	50	10	8	Complete	100
Sample_T8A_k141_223472	100398	165	15	1	Complete	100
Sample_T8A_k141_20937_fragment_2	38509	53	16	1	High-quality	100
Sample_T8A_k141_252853	71627	134	4	2	High-quality	100
Sample_T8A_k141_63499	47946	60	12	4	High-quality	100
Sample_T8B_k141_125937_fragment_1	120544	155	28	13	High-quality	100
Sample_T8B_k141_67249_fragment_1	84094	108	33	4	High-quality	100
Sample_T8B_k141_188950	100574	168	16	1	High-quality	100

Sample_T8B_k141_97067	46465	67	16	4	High-quality	100
Sample_T8B_k141_272687	162197	208	40	2	High-quality	100
Sample_T8B_k141_280025	46369	55	8	1	Complete	100
Sample_T8B_k141_69296	32525	47	11	0	Complete	100
Sample_T8B_k141_207622_fragment_1	51457	64	23	1	High-quality	100
Sample_T8B_k141_280022	40649	75	17	0	Complete	100
Sample_T8B_k141_280028	44032	55	7	1	Complete	100
Sample_T8B_k141_38801	36616	56	23	2	High-quality	100
Sample_T8C_k141_136265_fragment_1	43711	50	22	2	High-quality	100
Sample_T8C_k141_70732	100760	171	20	2	Complete	100
Sample_T8C_k141_55157	46962	83	19	1	Complete	100
Sample_T8C_k141_17544	102034	108	24	0	High-quality	100
Sample_T8C_k141_220779	51043	79	18	1	Complete	100
Sample_T8C_k141_75853_fragment_1	37024	43	1	7	High-quality	100
Sample_T8C_k141_220773	76413	90	16	1	Complete	100
Sample_T8C_k141_220762	38342	54	22	1	Complete	100
Sample_T8C_k141_220765	101860	130	21	4	Complete	100
Sample_T8C_k141_102001	43397	71	14	3	High-quality	100
Sample_T8C_k141_158990	42569	81	16	0	Complete	100
Sample_T8C_k141_220772	5173	9	5	0	Complete	100
Sample_T8C_k141_128938_fragment_2	39915	45	16	2	High-quality	100
Sample_T8C_k141_52200_fragment_2	43274	49	23	0	High-quality	100
Sample_T13B_k141_135470_fragment_2	55116	74	12	3	High-quality	99.99
Sample_T12A_k141_336036	46841	74	27	0	High-quality	99.98
Sample_T2C_k141_259480	37562	59	26	3	High-quality	99.98
Sample_T16C_k141_128143	46768	36	0	18	High-quality	99.96
Sample_T7A_k141_185647_fragment_1	166488	243	27	12	High-quality	99.96
Sample_T3A_k141_18283	43430	56	29	0	High-quality	99.94
Sample_T6A_k141_248321	35169	60	10	0	High-quality	99.94
Sample_T14A_k141_178501_fragment_1	33230	47	13	0	High-quality	99.91
Sample_T5A_k141_13334	590821	523	6	388	High-quality	99.9
Sample_T8C_k141_74250_fragment_2	33259	48	8	2	High-quality	99.88
Sample_C14A_k141_21472	57578	81	30	2	High-quality	99.79
Sample_C9C_k141_22552_fragment_2	39915	39	5	0	High-quality	99.79
Sample_C9B_k141_82784_fragment_1	39435	55	38	1	High-quality	99.78
Sample_C6B_k141_15690_fragment_1	61964	96	33	2	High-quality	99.76
Sample_T16A_k141_42942_fragment_1	48107	64	19	9	High-quality	99.75
Sample_C4A_k141_162034	38563	63	19	2	High-quality	99.72
Sample_T3B_k141_124107	41316	70	15	1	High-quality	99.71

Sample_C12C_k141_90382_fragment_2	42336	60	16	4	High-quality	99.7
Sample_C7B_k141_121863	15212	19	1	0	High-quality	99.69
Sample_T10A_k141_118057_fragment_1	82076	93	24	0	High-quality	99.69
Sample_T14B_k141_43796_fragment_1	13155	16	2	0	High-quality	99.69
Sample_C4B_k141_132982	42126	71	19	0	High-quality	99.68
Sample_T4B_k141_20101_fragment_2	33914	65	15	1	High-quality	99.68
Sample_T17B_k141_25674_fragment_1	38018	55	20	1	High-quality	99.67
Sample_T3A_k141_90266_fragment_1	60912	94	20	2	High-quality	99.67
Sample_C3C_k141_37123_fragment_3	71289	107	29	5	High-quality	99.66
Sample_C6A_k141_87585	32902	51	18	0	High-quality	99.66
Sample_T6B_k141_81691_fragment_1	39358	56	23	2	High-quality	99.65
Sample_C3C_k141_102604_fragment_1	64828	84	30	12	High-quality	99.63
Sample_T8C_k141_194333	6478	9	3	0	High-quality	99.61
Sample_C8A_k141_116974_fragment_1	43383	55	42	2	High-quality	99.59
Sample_C12C_k141_148549	120744	92	2	57	High-quality	99.57
Sample_C8C_k141_98367_fragment_2	46792	58	7	7	High-quality	99.56
Sample_C5A_k141_229889_fragment_2	60972	96	15	5	High-quality	99.53
Sample_T17A_k141_28037_fragment_1	47928	56	39	5	High-quality	99.53
Sample_T5A_k141_17305_fragment_1	44673	68	16	3	High-quality	99.53
Sample_T4B_k141_272896	76288	105	36	12	High-quality	99.52
Sample_T6C_k141_10410_fragment_1	38855	53	21	0	High-quality	99.51
Sample_C3C_k141_211464_fragment_1	66513	101	36	3	High-quality	99.48
Sample_C1B_k141_45294	86197	96	1	48	High-quality	99.46
Sample_C10A_k141_43742_fragment_3	108578	159	35	0	High-quality	99.46
Sample_T7A_k141_152753	76626	88	12	12	High-quality	99.42
Sample_T12A_k141_341193	34630	48	13	1	High-quality	99.41
Sample_T5C_k141_144118	12990	18	0	0	High-quality	99.4
Sample_C9B_k141_62223	176469	228	40	14	High-quality	99.39
Sample_C7B_k141_53133	129884	136	3	70	High-quality	99.37
Sample_C5A_k141_266654_fragment_1	42164	60	22	2	High-quality	99.36
Sample_T17A_k141_5871_fragment_1	41986	54	24	1	High-quality	99.36
Sample_C11B_k141_82569	92540	91	10	0	High-quality	99.33
Sample_C11A_k141_175841_fragment_1	116993	161	12	7	High-quality	99.32
Sample_C3B_k141_39335	84260	119	33	15	High-quality	99.3
Sample_C14C_k141_65618	54809	89	16	0	High-quality	99.29
Sample_T11A_k141_29613_fragment_1	41523	62	19	0	High-quality	99.29
Sample_C6C_k141_15010	151188	149	4	29	High-quality	99.26
Sample_C9C_k141_65039	60714	113	15	0	High-quality	99.26
Sample_T16B_k141_18665	46259	76	22	2	High-quality	99.26

Sample_C13B_k141_60370	32737	56	26	0	High-quality	99.25
Sample_T11A_k141_131184	56639	94	19	2	High-quality	99.23
Sample_T1C_k141_27645	41074	62	6	0	High-quality	99.23
Sample_C11B_k141_203627_fragment_2	39479	55	17	2	High-quality	99.22
Sample_C4C_k141_69840_fragment_3	38751	64	21	2	High-quality	99.19
Sample_C8B_k141_50394	168304	144	1	73	High-quality	99.18
Sample_C3C_k141_91876_fragment_1	47493	62	27	2	High-quality	99.18
Sample_T11A_k141_55716_fragment_1	18897	24	3	1	High-quality	99.16
Sample_T16B_k141_123510	34626	56	24	1	High-quality	99.15
Sample_C14C_k141_66746_fragment_1	37813	56	10	1	High-quality	99.12
Sample_C3A_k141_135664	5039	8	6	0	High-quality	99.11
Sample_T10A_k141_166304_fragment_1	65760	84	24	8	High-quality	99.11
Sample_T6A_k141_270385_fragment_1	38313	60	18	0	High-quality	99.11
Sample_C5B_k141_30497	48013	65	32	2	High-quality	99.09
Sample_T7C_k141_109488	31974	48	18	1	High-quality	99.06
Sample_T17A_k141_6811_fragment_1	40800	59	36	2	High-quality	99.05
Sample_T4C_k141_115428	172672	172	2	140	High-quality	99.04
Sample_T10B_k141_126040	11654	14	5	0	High-quality	99.02
Sample_C8C_k141_70284_fragment_1	38659	52	25	2	High-quality	99.01
Sample_T10B_k141_77567	87532	143	30	4	High-quality	99.01
Sample_C5B_k141_47177_fragment_1	46378	71	53	0	High-quality	98.99
Sample_T10A_k141_51721_fragment_2	55459	86	15	3	High-quality	98.99
Sample_C13C_k141_7882_fragment_1	165952	258	43	5	High-quality	98.93
Sample_C3A_k141_98778	149248	144	3	86	High-quality	98.91
Sample_T16B_k141_145085_fragment_1	41679	73	17	0	High-quality	98.91
Sample_C1C_k141_82352	61622	79	32	1	High-quality	98.85
Sample_C3B_k141_34919	41703	48	3	2	High-quality	98.85
Sample_T18C_k141_105796_fragment_2	40935	62	6	2	High-quality	98.83
Sample_T7B_k141_32364_fragment_1	38557	52	15	4	High-quality	98.83
Sample_T4B_k141_152293_fragment_1	37627	50	18	0	High-quality	98.82
Sample_C5B_k141_53606_fragment_2	49714	69	26	7	High-quality	98.81
Sample_C2B_k141_7383	79985	94	2	44	High-quality	98.79
Sample_C4C_k141_60890	41310	76	19	0	High-quality	98.79
Sample_C11B_k141_76327_fragment_2	55030	71	33	3	High-quality	98.74
Sample_C7B_k141_60805_fragment_3	62091	92	31	0	High-quality	98.73
Sample_T15C_k141_98279_fragment_1	39225	57	13	0	High-quality	98.71
Sample_C12C_k141_204221	486054	509	4	300	High-quality	98.7
Sample_C3A_k141_213857	283688	233	2	187	High-quality	98.7
Sample_C10A_k141_46207_fragment_1	38526	54	19	0	High-quality	98.7

Sample_C12C_k141_74287_fragment_1	36835	52	15	1	High-quality	98.7
Sample_C11C_k141_43380_fragment_3	49700	78	29	2	High-quality	98.69
Sample_C11B_k141_228958	39164	78	16	2	High-quality	98.68
Sample_T7B_k141_6164	353131	289	2	200	High-quality	98.67
Sample_T6C_k141_65311_fragment_3	33860	52	16	0	High-quality	98.67
Sample_C13B_k141_14966	167319	153	1	128	High-quality	98.65
Sample_T11B_k141_123292	180539	168	3	98	High-quality	98.62
Sample_C12B_k141_105716	41640	69	20	0	High-quality	98.62
Sample_C10A_k141_68661	46408	70	23	1	High-quality	98.6
Sample_C3A_k141_10017	53020	84	20	5	High-quality	98.59
Sample_C14A_k141_196082	28281	26	0	4	High-quality	98.58
Sample_T13C_k141_15427	29186	43	19	0	High-quality	98.55
Sample_T15B_k141_46089	56814	91	17	2	High-quality	98.55
Sample_T11A_k141_151272_fragment_1	37043	55	20	1	High-quality	98.54
Sample_C10B_k141_108070	5652	11	1	1	High-quality	98.46
Sample_C9A_k141_13768_fragment_1	47937	72	16	5	High-quality	98.46
Sample_C1C_k141_169631_fragment_1	38261	36	4	0	High-quality	98.44
Sample_C2A_k141_88272	233308	298	14	21	High-quality	98.41
Sample_T2B_k141_245773_fragment_1	59100	51	5	6	High-quality	98.4
Sample_T17A_k141_4577	18751	27	2	0	High-quality	98.39
Sample_C13C_k141_79964	156572	167	2	133	High-quality	98.37
Sample_C7B_k141_64610_fragment_1	39019	52	14	3	High-quality	98.36
Sample_T4C_k141_92468_fragment_2	134753	178	36	6	High-quality	98.35
Sample_C9A_k141_22623_fragment_1	88359	95	24	5	High-quality	98.33
Sample_C4B_k141_46019	41313	32	0	22	High-quality	98.3
Sample_C2C_k141_188116	38816	57	9	1	High-quality	98.27
Sample_C4C_k141_227607	47589	65	21	4	High-quality	98.26
Sample_C4B_k141_254537	79205	123	21	4	High-quality	98.24
Sample_T11C_k141_85717	188462	174	4	60	High-quality	98.23
Sample_C13B_k141_57996	13123	16	4	0	High-quality	98.23
Sample_T17C_k141_8141_fragment_2	173269	273	42	4	High-quality	98.21
Sample_T6B_k141_152191	108348	99	1	56	High-quality	98.2
Sample_T17B_k141_20688_fragment_1	40757	67	17	5	High-quality	98.18
Sample_T3C_k141_84873_fragment_1	38698	49	7	0	High-quality	98.18
Sample_T8A_k141_122726	65125	89	22	2	High-quality	98.18
Sample_C11A_k141_21789	64545	60	2	39	High-quality	98.16
Sample_T10A_k141_39858_fragment_1	38373	55	20	0	High-quality	98.16
Sample_C3A_k141_242979	40133	72	17	1	High-quality	98.15
Sample_T3B_k141_91254_fragment_1	42241	58	13	7	High-quality	98.13

Sample_C4B_k141_4453	18695	31	6	2	High-quality	98.1
Sample_T4B_k141_50359	183188	180	1	102	High-quality	98.06
Sample_C1B_k141_244261_fragment_2	51929	58	13	1	High-quality	98.05
Sample_C8C_k141_139847_fragment_1	53280	71	15	4	High-quality	98.05
Sample_T13A_k141_48041	31593	61	15	0	High-quality	98.02
Sample_C8A_k141_95430_fragment_1	42411	57	18	0	High-quality	97.98
Sample_T4B_k141_196760	287817	345	12	34	High-quality	97.98
Sample_T2B_k141_196613	57113	81	13	2	High-quality	97.96
Sample_T13C_k141_17830	13084	18	4	0	High-quality	97.94
Sample_T15B_k141_113721_fragment_1	44283	51	2	4	High-quality	97.93
Sample_T3A_k141_110712_fragment_1	40652	63	19	1	High-quality	97.93
Sample_C8C_k141_95882	33137	52	18	0	High-quality	97.91
Sample_T15C_k141_136462	65789	93	23	5	High-quality	97.88
Sample_T15A_k141_114057_fragment_1	65293	93	13	7	High-quality	97.87
Sample_C10B_k141_1724	36204	59	20	1	High-quality	97.84
Sample_C7A_k141_77406	61426	83	22	4	High-quality	97.81
Sample_C9A_k141_16007_fragment_1	30681	38	11	4	High-quality	97.8
Sample_T14A_k141_74924_fragment_1	65107	100	29	2	High-quality	97.8
Sample_T12A_k141_190546_fragment_5	36028	49	16	2	High-quality	97.79
Sample_C2B_k141_343189	45294	77	18	0	High-quality	97.78
Sample_C3A_k141_31824_fragment_1	34252	49	16	1	High-quality	97.78
Sample_C11A_k141_84190	100490	178	28	1	High-quality	97.77
Sample_T17A_k141_22903_fragment_1	39323	60	14	0	High-quality	97.77
Sample_T11A_k141_104938_fragment_1	63179	97	29	0	High-quality	97.75
Sample_C2B_k141_139571	50252	70	19	2	High-quality	97.72
Sample_T11B_k141_55754_fragment_1	84929	101	13	12	High-quality	97.69
Sample_C10A_k141_194162	56011	69	30	2	High-quality	97.68
Sample_T11B_k141_130303	13048	16	4	0	High-quality	97.67
Sample_C1C_k141_49705_fragment_2	37756	53	17	4	High-quality	97.65
Sample_C9A_k141_105520_fragment_1	36518	55	20	1	High-quality	97.63
Sample_T11B_k141_17411	56107	55	5	4	High-quality	97.61
Sample_T3A_k141_80611	151625	147	1	102	High-quality	97.58
Sample_T4C_k141_69330	197678	214	5	90	High-quality	97.58
Sample_T6C_k141_223830_fragment_3	37806	41	8	0	High-quality	97.58
Sample_T4B_k141_75764_fragment_1	44137	51	3	2	High-quality	97.55
Sample_T8A_k141_111862	58502	97	20	2	High-quality	97.54
Sample_C14A_k141_79774_fragment_1	46572	68	23	2	High-quality	97.52
Sample_C1C_k141_59071	216233	228	3	148	High-quality	97.5
Sample_C12A_k141_174271_fragment_1	32859	60	23	0	High-quality	97.5

Sample_T4C_k141_63506	40862	71	31	2	High-quality	97.5
Sample_T7B_k141_34219	64520	97	32	5	High-quality	97.5
Sample_C12C_k141_180066_fragment_2	30372	57	14	0	High-quality	97.48
Sample_C14C_k141_59006	43920	73	17	0	High-quality	97.45
Sample_T10B_k141_165906_fragment_2	34553	54	22	0	High-quality	97.44
Sample_C10A_k141_168339	42932	58	33	0	High-quality	97.41
Sample_T8C_k141_15282	96684	174	21	1	High-quality	97.41
Sample_T15A_k141_114774_fragment_1	43623	61	18	6	High-quality	97.39
Sample_C2A_k141_129915	42135	64	15	0	High-quality	97.38
Sample_T11C_k141_97857_fragment_1	55307	79	22	4	High-quality	97.38
Sample_T6C_k141_111290_fragment_2	28836	42	15	0	High-quality	97.38
Sample_T5B_k141_44934_fragment_1	37389	51	14	3	High-quality	97.37
Sample_C3C_k141_142500_fragment_1	52936	73	16	0	High-quality	97.36
Sample_T12C_k141_75553	84456	112	13	1	High-quality	97.34
Sample_C2A_k141_326487	33188	49	13	2	High-quality	97.33
Sample_T8C_k141_113721_fragment_1	42059	51	15	4	High-quality	97.33
Sample_T13B_k141_34116_fragment_1	39496	56	32	0	High-quality	97.31
Sample_T18C_k141_94868	210877	211	3	118	High-quality	97.27
Sample_C12B_k141_142768_fragment_1	67403	97	20	5	High-quality	97.25
Sample_T13B_k141_64194_fragment_1	48254	74	28	0	High-quality	97.24
Sample_T13C_k141_107238_fragment_1	39888	59	25	6	High-quality	97.23
Sample_C8B_k141_27979_fragment_1	36051	53	30	1	High-quality	97.18
Sample_C11A_k141_39455_fragment_1	37911	54	14	6	High-quality	97.17
Sample_C14A_k141_24421	45916	59	18	7	High-quality	97.17
Sample_C8A_k141_165788_fragment_1	112807	145	18	8	High-quality	97.17
Sample_T14C_k141_111835_fragment_2	44295	45	3	3	High-quality	97.15
Sample_C12C_k141_39734_fragment_4	100670	117	36	7	High-quality	97.1
Sample_T4B_k141_303023	78447	90	1	32	High-quality	97.09
Sample_C14A_k141_160402_fragment_2	43364	58	19	5	High-quality	97.09
Sample_T5C_k141_111254	33177	51	15	1	High-quality	97.08
Sample_T5A_k141_33751	39953	69	17	1	High-quality	97.06
Sample_C13C_k141_95846	40523	64	14	3	High-quality	97.05
Sample_T13C_k141_88200_fragment_1	47959	67	30	0	High-quality	97.05
Sample_C14C_k141_81950_fragment_1	39119	56	21	0	High-quality	96.93
Sample_T4B_k141_118753	202394	240	11	14	High-quality	96.93
Sample_C2A_k141_129866	141535	124	1	86	High-quality	96.91
Sample_C3B_k141_22762	317395	403	10	29	High-quality	96.89
Sample_T14A_k141_192459_fragment_1	33738	53	20	0	High-quality	96.89
Sample_C12A_k141_184915	64432	70	3	43	High-quality	96.88

Sample_C1B_k141_138451	79396	95	13	0	High-quality	96.83
Sample_C7B_k141_154868	76404	134	16	3	High-quality	96.82
Sample_T6B_k141_44265_fragment_1	63264	90	13	5	High-quality	96.8
Sample_T4B_k141_41114	34318	50	15	1	High-quality	96.76
Sample_C4B_k141_82947_fragment_1	50321	71	18	1	High-quality	96.75
Sample_C13C_k141_67174	178372	142	1	106	High-quality	96.74
Sample_T2C_k141_67460_fragment_2	32515	48	13	2	High-quality	96.74
Sample_C6A_k141_148773_fragment_1	47668	63	20	1	High-quality	96.68
Sample_T13A_k141_75046_fragment_1	142250	197	41	3	High-quality	96.6
Sample_T12A_k141_77574_fragment_2	30124	56	17	0	High-quality	96.57
Sample_C10A_k141_243259	41646	72	16	5	High-quality	96.55
Sample_C11A_k141_83652	81378	139	17	1	High-quality	96.55
Sample_C6A_k141_129111_fragment_1	52830	81	21	3	High-quality	96.51
Sample_T4A_k141_64317	64709	89	13	5	High-quality	96.51
Sample_T13B_k141_85007_fragment_1	46417	63	27	6	High-quality	96.45
Sample_T14A_k141_159142	13190	17	4	0	High-quality	96.45
Sample_C11A_k141_183850	99045	95	2	31	High-quality	96.43
Sample_C2C_k141_224837	40030	75	20	4	High-quality	96.42
Sample_T6B_k141_122698	5891	7	4	0	High-quality	96.42
Sample_C10A_k141_283124_fragment_1	41073	57	28	2	High-quality	96.37
Sample_T13B_k141_99329	19774	26	5	5	High-quality	96.32
Sample_T10B_k141_94041_fragment_1	47092	73	20	2	High-quality	96.32
Sample_C10A_k141_107111_fragment_1	40761	60	24	0	High-quality	96.29
Sample_T15C_k141_77534_fragment_3	66294	88	13	3	High-quality	96.29
Sample_T12C_k141_39915_fragment_1	55881	86	16	2	High-quality	96.26
Sample_T8B_k141_167481	14887	17	1	0	High-quality	96.21
Sample_T4B_k141_1449	293083	235	3	151	High-quality	96.18
Sample_T4B_k141_168818	40240	71	21	0	High-quality	96.12
Sample_T5C_k141_60686	230412	206	3	116	High-quality	96.11
Sample_C14B_k141_19598_fragment_1	58661	86	25	3	High-quality	96.1
Sample_T12C_k141_6537	13096	17	4	0	High-quality	96.1
Sample_T17B_k141_11328_fragment_1	37210	55	13	0	High-quality	96.1
Sample_T8B_k141_22749	56275	101	20	0	High-quality	96.07
Sample_C11C_k141_120295	6556	8	3	0	High-quality	96.04
Sample_T18B_k141_78754	39919	49	22	3	High-quality	96.01
Sample_T3A_k141_78848	39777	69	16	0	High-quality	95.99
Sample_C8C_k141_69656_fragment_2	46454	49	6	3	High-quality	95.97
Sample_C14A_k141_157372_fragment_2	79001	89	24	2	High-quality	95.96
Sample_T8B_k141_196088	52798	70	19	3	High-quality	95.96

Sample_T3C_k141_28820	244241	213	4	141	High-quality	95.94
Sample_C11C_k141_173719_fragment_1	34761	51	15	1	High-quality	95.9
Sample_T16B_k141_18941	36397	38	6	1	High-quality	95.87
Sample_C10B_k141_29481	79838	74	5	22	High-quality	95.85
Sample_T12A_k141_75688_fragment_1	49543	80	21	2	High-quality	95.85
Sample_T2C_k141_19497_fragment_2	56859	66	20	3	High-quality	95.8
Sample_C1C_k141_2084_fragment_1	128059	184	31	0	High-quality	95.79
Sample_T5A_k141_7150_fragment_2	14843	23	2	1	High-quality	95.78
Sample_T17B_k141_10966_fragment_1	44169	62	38	0	High-quality	95.69
Sample_T5C_k141_41360_fragment_1	45693	62	7	4	High-quality	95.68
Sample_C4A_k141_93819_fragment_1	41956	54	20	3	High-quality	95.65
Sample_C4C_k141_57566_fragment_1	35240	49	16	2	High-quality	95.65
Sample_T6B_k141_83017_fragment_1	105976	133	14	3	High-quality	95.62
Sample_C10C_k141_1286	40370	41	0	16	High-quality	95.59
Sample_C13A_k141_14544	181434	169	1	120	High-quality	95.57
Sample_C11C_k141_113834	40031	54	23	1	High-quality	95.52
Sample_T18A_k141_92894	73237	68	5	7	High-quality	95.51
Sample_T18B_k141_141006	50974	78	27	0	High-quality	95.51
Sample_T5B_k141_59199_fragment_1	50589	59	27	8	High-quality	95.51
Sample_C8C_k141_3791_fragment_1	41819	47	7	0	High-quality	95.49
Sample_C4B_k141_23689_fragment_1	16041	26	7	0	High-quality	95.47
Sample_T18C_k141_82937_fragment_2	68575	90	23	7	High-quality	95.46
Sample_C11C_k141_187492	6917	8	3	1	High-quality	95.44
Sample_T13B_k141_111723	39741	57	21	0	High-quality	95.42
Sample_C14C_k141_154327	87693	104	12	18	High-quality	95.37
Sample_T2B_k141_27301_fragment_2	197521	209	3	27	High-quality	95.31
Sample_C13A_k141_48859	214472	188	3	125	High-quality	95.29
Sample_C1C_k141_50529_fragment_1	46259	70	23	1	High-quality	95.29
Sample_T5C_k141_28092_fragment_1	45928	66	21	1	High-quality	95.27
Sample_T5A_k141_44607	41503	80	20	0	High-quality	95.21
Sample_C12B_k141_56025_fragment_1	37635	57	15	6	High-quality	95.15
Sample_T12C_k141_79893	99168	119	11	2	High-quality	95.14
Sample_C7A_k141_25121_fragment_1	57534	65	29	1	High-quality	95.13
Sample_T12A_k141_108674	35172	52	11	2	High-quality	95.1
Sample_C11A_k141_45944	56875	81	31	0	High-quality	95.05
Sample_T14A_k141_147625_fragment_1	61298	94	27	5	High-quality	95.05
Sample_C5A_k141_208982	46909	66	17	1	High-quality	95.03
Sample_C11B_k141_69133	188052	136	1	99	High-quality	95.01
Sample_T14C_k141_11679_fragment_1	39472	51	4	0	High-quality	95.01

Sample_T18A_k141_43633_fragment_1	38631	59	21	0	High-quality	95.01
Sample_C8B_k141_11746_fragment_1	38150	52	21	0	High-quality	94.96
Sample_T8A_k141_169132	46952	69	27	1	High-quality	94.95
Sample_T5B_k141_11379_fragment_1	30804	46	13	1	High-quality	94.94
Sample_C12A_k141_120292_fragment_2	60415	82	15	5	High-quality	94.89
Sample_C13A_k141_113447_fragment_2	18082	26	2	0	High-quality	94.88
Sample_T12C_k141_35132	33488	54	18	1	High-quality	94.88
Sample_T13B_k141_77557_fragment_1	48613	60	24	7	High-quality	94.86
Sample_T3C_k141_14860_fragment_3	36428	46	18	0	High-quality	94.83
Sample_T17C_k141_789_fragment_3	37986	55	13	3	High-quality	94.81
Sample_C5A_k141_122136	206558	199	2	121	High-quality	94.77
Sample_T10C_k141_55895	103215	154	18	1	High-quality	94.76
Sample_T10B_k141_63341_fragment_1	39653	54	21	1	High-quality	94.74
Sample_T7A_k141_77584_fragment_2	51447	82	18	3	High-quality	94.74
Sample_C11B_k141_169387	34732	49	9	1	High-quality	94.67
Sample_C2B_k141_14652	43321	82	24	0	High-quality	94.66
Sample_C2B_k141_178144	56105	76	31	0	High-quality	94.6
Sample_T5C_k141_66503	99733	113	2	11	High-quality	94.59
Sample_C10A_k141_66913	149892	128	1	74	High-quality	94.58
Sample_C1A_k141_28638	315808	292	4	201	High-quality	94.58
Sample_T13A_k141_50134_fragment_1	61413	101	24	1	High-quality	94.55
Sample_T3B_k141_4559	37052	51	10	0	High-quality	94.55
Sample_C14A_k141_168150	44272	65	21	1	High-quality	94.5
Sample_C12A_k141_185732	177837	183	1	119	High-quality	94.49
Sample_T5C_k141_63527	42345	43	5	4	High-quality	94.47
Sample_C3A_k141_110020_fragment_1	149514	164	7	19	High-quality	94.46
Sample_C11B_k141_56766_fragment_1	49201	52	26	1	High-quality	94.45
Sample_C3C_k141_62753	46476	54	9	6	High-quality	94.44
Sample_C6C_k141_115044	53960	95	13	1	High-quality	94.43
Sample_C8C_k141_126373	60457	43	1	33	High-quality	94.41
Sample_T15A_k141_35712	38488	54	16	0	High-quality	94.39
Sample_T2A_k141_225750	174003	155	1	57	High-quality	94.38
Sample_C14A_k141_53676	15890	19	2	1	High-quality	94.36
Sample_T13B_k141_68497_fragment_1	37896	48	32	3	High-quality	94.36
Sample_C4C_k141_63860	84867	62	0	43	High-quality	94.3
Sample_C7B_k141_141831	21361	35	3	4	High-quality	94.3
Sample_T8C_k141_152747	14592	16	0	1	High-quality	94.29
Sample_T2A_k141_2964	43115	60	14	0	High-quality	94.25
Sample_T5B_k141_34147	75997	104	23	5	High-quality	94.25

Sample_T2C_k141_93502_fragment_1	38676	55	18	2	High-quality	94.21
Sample_T10B_k141_110985	57198	78	21	12	High-quality	94.12
Sample_T5B_k141_75400_fragment_1	49137	51	5	1	High-quality	94.11
Sample_C3A_k141_132196_fragment_1	69286	76	4	10	High-quality	94.07
Sample_C3B_k141_165550_fragment_2	32469	49	15	0	High-quality	94.07
Sample_T2C_k141_55412	42507	66	19	0	High-quality	94.03
Sample_T8C_k141_44672_fragment_1	32553	51	20	1	High-quality	94.03
Sample_C13A_k141_81830	104651	105	4	47	High-quality	94.01
Sample_C6B_k141_203635	74767	60	0	12	High-quality	93.99
Sample_C7C_k141_61017_fragment_1	36671	48	20	1	High-quality	93.99
Sample_C1B_k141_106541	32141	48	13	4	High-quality	93.97
Sample_C10A_k141_306856_fragment_1	70654	76	9	8	High-quality	93.94
Sample_T1A_k141_10294	107661	121	34	2	High-quality	93.94
Sample_T16C_k141_89252_fragment_3	62015	85	16	5	High-quality	93.92
Sample_T3A_k141_135581	13044	19	5	0	High-quality	93.92
Sample_T10B_k141_67047_fragment_4	265997	372	12	24	High-quality	93.9
Sample_C5A_k141_1888	42256	66	17	0	High-quality	93.89
Sample_C14C_k141_77058	33927	46	20	0	High-quality	93.87
Sample_C3A_k141_259430_fragment_1	36502	45	32	2	High-quality	93.8
Sample_C1C_k141_39922	191708	160	3	103	High-quality	93.78
Sample_T2A_k141_54976	149577	157	3	95	High-quality	93.78
Sample_C9A_k141_24615_fragment_1	35879	46	14	0	High-quality	93.77
Sample_T13B_k141_136019_fragment_1	38715	53	22	2	High-quality	93.72
Sample_C5A_k141_74556	132364	134	2	73	High-quality	93.68
Sample_T12B_k141_115067	33690	54	22	0	High-quality	93.66
Sample_T13C_k141_53604_fragment_1	47220	59	19	2	High-quality	93.58
Sample_T7A_k141_126017	54389	75	18	4	High-quality	93.58
Sample_C9A_k141_54863	42847	41	2	1	High-quality	93.55
Sample_C11A_k141_102157_fragment_3	45100	69	20	2	High-quality	93.53
Sample_C6C_k141_214905	46657	53	18	3	High-quality	93.48
Sample_C10C_k141_211157_fragment_2	56377	79	15	1	High-quality	93.41
Sample_C14B_k141_25233	35285	48	17	0	High-quality	93.41
Sample_C11B_k141_21663_fragment_1	54394	99	23	0	High-quality	93.39
Sample_T2B_k141_208679	200711	149	2	119	High-quality	93.31
Sample_C10B_k141_111501_fragment_1	51310	58	7	7	High-quality	93.3
Sample_C4B_k141_165352	5235	5	0	1	High-quality	93.22
Sample_T2C_k141_148008_fragment_1	43966	57	24	3	High-quality	93.2
Sample_T11C_k141_124121_fragment_2	40302	62	28	0	High-quality	93.12
Sample_C7B_k141_109589_fragment_1	61295	87	11	8	High-quality	93.11

Sample_T11B_k141_17133_fragment_1	32902	50	17	3	High-quality	93.04
Sample_T1A_k141_6836	41178	54	23	4	High-quality	93.02
Sample_C11A_k141_51947	43534	42	7	2	High-quality	93.01
Sample_C12B_k141_115450	13313	20	3	1	High-quality	93.01
Sample_T11C_k141_50232_fragment_1	58127	87	18	0	High-quality	93.01
Sample_T16A_k141_62093_fragment_1	54746	86	25	4	High-quality	93.01
Sample_T3C_k141_133467_fragment_1	60454	69	21	10	High-quality	92.99
Sample_C6C_k141_52784_fragment_1	53613	65	24	8	High-quality	92.98
Sample_C10A_k141_280900_fragment_1	39055	60	19	0	High-quality	92.96
Sample_C13C_k141_94466_fragment_2	12136	20	2	0	High-quality	92.95
Sample_C10B_k141_71011	35354	48	23	0	High-quality	92.94
Sample_C14C_k141_160588_fragment_5	34020	45	5	2	High-quality	92.8
Sample_C5A_k141_198515_fragment_2	82157	113	20	13	High-quality	92.72
Sample_T4B_k141_327358_fragment_3	44805	74	15	6	High-quality	92.66
Sample_T8A_k141_235691_fragment_1	37332	59	18	2	High-quality	92.64
Sample_T11A_k141_40225	292940	235	3	173	High-quality	92.61
Sample_T10B_k141_84318	42468	60	26	1	High-quality	92.55
Sample_T2A_k141_241183_fragment_2	37072	55	12	0	High-quality	92.53
Sample_T7A_k141_125965_fragment_1	44870	50	24	2	High-quality	92.53
Sample_T12A_k141_195205_fragment_2	44845	60	9	0	High-quality	92.49
Sample_C1C_k141_16632_fragment_1	96618	169	24	11	High-quality	92.46
Sample_C13C_k141_70322	32041	15	1	4	High-quality	92.4
Sample_C2A_k141_235023_fragment_2	104048	166	35	5	High-quality	92.37
Sample_C2B_k141_256757_fragment_2	40517	59	21	3	High-quality	92.36
Sample_T11C_k141_90820_fragment_1	29940	47	14	1	High-quality	92.25
Sample_C3C_k141_244244_fragment_2	40387	42	24	1	High-quality	92.24
Sample_C7B_k141_7029	38886	59	17	3	High-quality	92.24
Sample_C13C_k141_35877	32134	49	13	1	High-quality	92.22
Sample_C7A_k141_155146_fragment_2	44475	61	22	1	High-quality	92.22
Sample_T5C_k141_11084	17560	26	6	0	High-quality	92.21
Sample_T6A_k141_15920	49412	65	13	9	High-quality	92.21
Sample_T10A_k141_27819_fragment_3	45739	52	9	5	High-quality	92.19
Sample_T12B_k141_94405_fragment_1	39205	62	29	1	High-quality	92.19
Sample_C13C_k141_13326_fragment_1	43508	61	27	2	High-quality	92.16
Sample_C8B_k141_154874	15673	20	7	1	High-quality	92.14
Sample_T14A_k141_74898	37472	33	5	1	High-quality	92.11
Sample_C8C_k141_167785_fragment_1	36530	53	24	0	High-quality	92.08
Sample_C11B_k141_95000	85902	88	2	29	High-quality	92.06
Sample_T16B_k141_89977	35926	47	19	0	High-quality	92.06

Sample_C3B_k141_100921	88347	85	9	0	High-quality	92.01
Sample_C6C_k141_49430_fragment_1	42513	53	22	2	High-quality	92
Sample_T8A_k141_219986_fragment_2	42586	56	27	1	High-quality	92
Sample_T2B_k141_76576_fragment_1	34772	43	16	0	High-quality	91.98
Sample_C13B_k141_72647_fragment_1	36173	52	19	0	High-quality	91.94
Sample_C2A_k141_301323	26507	34	10	0	High-quality	91.89
Sample_C12A_k141_194182_fragment_2	17505	26	3	0	High-quality	91.86
Sample_T4B_k141_185956	42038	56	31	0	High-quality	91.76
Sample_C11B_k141_238532	42460	60	36	0	High-quality	91.74
Sample_C3A_k141_54716_fragment_1	46205	64	22	5	High-quality	91.73
Sample_T4B_k141_112724_fragment_1	30831	38	10	0	High-quality	91.71
Sample_T17A_k141_6914	10785	17	5	0	High-quality	91.63
Sample_C8C_k141_5976_fragment_1	31908	34	1	5	High-quality	91.59
Sample_T4C_k141_16588_fragment_1	52734	69	19	7	High-quality	91.55
Sample_T15C_k141_32935	32156	45	18	1	High-quality	91.52
Sample_C12A_k141_20803	32590	46	14	2	High-quality	91.51
Sample_C14A_k141_54912	35709	50	13	1	High-quality	91.51
Sample_T11B_k141_49665_fragment_3	45125	62	20	5	High-quality	91.47
Sample_C12A_k141_162714_fragment_1	30040	47	19	0	High-quality	91.43
Sample_T11C_k141_52353_fragment_2	32038	44	18	0	High-quality	91.41
Sample_T7B_k141_152843_fragment_1	32272	40	19	0	High-quality	91.41
Sample_T2A_k141_38359_fragment_1	49705	68	22	4	High-quality	91.4
Sample_T16B_k141_125657_fragment_1	38996	51	12	3	High-quality	91.36
Sample_T14A_k141_58246_fragment_3	35362	38	6	0	High-quality	91.28
Sample_C9C_k141_71985_fragment_2	42460	59	29	5	High-quality	91.23
Sample_T14C_k141_96655_fragment_2	45214	57	13	1	High-quality	91.23
Sample_C2C_k141_263854	38339	38	0	19	High-quality	91.22
Sample_C1B_k141_34173_fragment_1	34984	50	18	1	High-quality	91.15
Sample_T4C_k141_92186_fragment_1	35828	48	20	3	High-quality	91.12
Sample_T4A_k141_110256	40962	54	28	0	High-quality	91.08
Sample_T13C_k141_73612_fragment_1	51613	62	19	4	High-quality	91.01
Sample_T5C_k141_85959	175864	143	3	96	High-quality	90.99
Sample_T3C_k141_58140_fragment_2	53397	77	30	5	High-quality	90.94
Sample_T5A_k141_31574	36397	68	24	0	High-quality	90.94
Sample_C7B_k141_75906_fragment_3	36201	58	19	1	High-quality	90.9
Sample_T14C_k141_13322	148043	141	1	87	High-quality	90.86
Sample_C13C_k141_45372	39749	51	25	0	High-quality	90.86
Sample_T6A_k141_124644	281975	223	2	157	High-quality	90.81
Sample_T2C_k141_102710_fragment_1	42914	63	17	2	High-quality	90.81

Sample_C6B_k141_35192_fragment_1	46855	63	18	5	High-quality	90.78
Sample_T16B_k141_13168_fragment_1	76878	102	16	17	High-quality	90.69
Sample_T16C_k141_12360_fragment_1	34411	52	22	0	High-quality	90.69
Sample_T5B_k141_14771	161881	109	1	71	High-quality	90.67
Sample_T12A_k141_311794_fragment_1	43246	40	6	2	High-quality	90.66
Sample_T8B_k141_101301_fragment_1	33853	67	16	0	High-quality	90.65
Sample_T13A_k141_122128	184798	178	1	73	High-quality	90.64
Sample_T15A_k141_6238	104241	95	2	29	High-quality	90.64
Sample_T14B_k141_121326	61270	81	13	5	High-quality	90.61
Sample_T18C_k141_118782_fragment_1	45528	64	18	1	High-quality	90.61
Sample_T8C_k141_160850_fragment_1	56110	93	19	5	High-quality	90.6
Sample_C11B_k141_123152	16768	21	9	0	High-quality	90.55
Sample_T4A_k141_7468	280203	196	2	138	High-quality	90.53
Sample_C8B_k141_143762_fragment_1	41779	62	30	0	High-quality	90.53
Sample_T2C_k141_86497	16714	22	9	0	High-quality	90.53
Sample_T2C_k141_134103_fragment_2	31242	43	14	0	High-quality	90.52
Sample_C3C_k141_7977_fragment_1	39353	61	26	1	High-quality	90.48
Sample_T13B_k141_132107	386648	372	2	209	High-quality	90.45
Sample_C10C_k141_139764_fragment_1	36703	58	28	0	High-quality	90.44
Sample_T16B_k141_148332	10694	18	5	1	High-quality	90.41
Sample_T11C_k141_146798_fragment_2	49389	64	27	7	High-quality	90.39
Sample_C12A_k141_26735	44940	69	20	3	High-quality	90.33
Sample_C12B_k141_69871	168641	165	1	87	High-quality	90.28
Sample_C9B_k141_107392	7278	10	1	0	High-quality	90.25
Sample_C14A_k141_5807_fragment_1	54545	88	29	0	High-quality	90.24
Sample_T4A_k141_40582	34987	53	19	0	High-quality	90.18
Sample_T14C_k141_127299_fragment_1	54833	59	4	6	High-quality	90.13
Sample_T4B_k141_60928_fragment_2	44797	52	3	4	High-quality	90.11
Sample_C8C_k141_80137_fragment_1	32502	43	21	1	High-quality	90.01
Sample_C7C_k141_105742_fragment_1	44432	66	26	5	High-quality	90
Sample_C2C_k141_195050	5850	8	2	0	Medium-quality	89.94
Sample_T16B_k141_150745	33558	53	20	1	Medium-quality	89.88
Sample_T2C_k141_183655_fragment_1	31904	40	21	1	Medium-quality	89.83
Sample_T6A_k141_289730_fragment_1	43959	60	21	3	Medium-quality	89.8
Sample_T6A_k141_229124_fragment_1	56915	83	35	3	Medium-quality	89.74
Sample_T12A_k141_109632	13179	22	3	0	Medium-quality	89.73
Sample_T12C_k141_17897_fragment_1	37452	51	26	0	Medium-quality	89.72
Sample_C10C_k141_47522	37536	58	16	1	Medium-quality	89.71
Sample_C11C_k141_156664_fragment_1	30612	48	19	1	Medium-quality	89.63

Sample_T12A_k141_4371	52485	83	24	6	Medium-quality	89.62
Sample_T2B_k141_219399_fragment_1	49537	72	20	1	Medium-quality	89.59
Sample_T7A_k141_102421_fragment_1	41043	53	39	0	Medium-quality	89.58
Sample_C13C_k141_101670	11056	15	1	0	Medium-quality	89.55
Sample_C2B_k141_234098	13555	18	1	2	Medium-quality	89.54
Sample_C8C_k141_92789_fragment_1	42727	59	10	4	Medium-quality	89.54
Sample_T13C_k141_91629_fragment_1	34997	57	18	0	Medium-quality	89.54
Sample_T5C_k141_131122	5461	7	4	0	Medium-quality	89.51
Sample_T6C_k141_28187	48505	75	20	0	Medium-quality	89.5
Sample_C5A_k141_52102	33068	48	19	0	Medium-quality	89.48
Sample_T15A_k141_112179	13640	16	2	1	Medium-quality	89.47
Sample_T4A_k141_68324	63099	61	2	15	Medium-quality	89.41
Sample_T13C_k141_7517_fragment_2	30859	44	13	1	Medium-quality	89.41
Sample_T12A_k141_297679	35085	62	13	0	Medium-quality	89.37
Sample_T4B_k141_291602	33000	54	16	0	Medium-quality	89.37
Sample_T11A_k141_87512_fragment_1	44612	81	23	1	Medium-quality	89.29
Sample_T12C_k141_130806	151182	188	8	11	Medium-quality	89.28
Sample_C6C_k141_4094	14959	24	6	0	Medium-quality	89.23
Sample_T8C_k141_6231_fragment_1	32441	49	16	2	Medium-quality	89.22
Sample_T3B_k141_56917	77849	87	23	3	Medium-quality	89.21
Sample_T5C_k141_182659_fragment_2	35243	44	19	1	Medium-quality	89.2
Sample_C4B_k141_138888_fragment_1	35947	41	23	0	Medium-quality	89.16
Sample_C4A_k141_143780	670344	608	2	481	Medium-quality	89.15
Sample_C13A_k141_78897	45970	61	11	5	Medium-quality	89.15
Sample_T2B_k141_40071	37371	46	9	1	Medium-quality	89.13
Sample_C13C_k141_111306_fragment_1	39015	41	23	0	Medium-quality	89.11
Sample_C14C_k141_44601	38803	52	28	1	Medium-quality	89.05
Sample_T4B_k141_150947	192186	157	3	75	Medium-quality	89.02
Sample_T15A_k141_173738_fragment_1	44146	63	17	1	Medium-quality	89.02
Sample_T2A_k141_84521	150868	223	35	3	Medium-quality	88.94
Sample_C5A_k141_1290	144297	138	1	89	Medium-quality	88.93
Sample_T15B_k141_97166	40004	46	1	27	Medium-quality	88.91
Sample_T3B_k141_44490_fragment_1	31908	45	16	1	Medium-quality	88.91
Sample_C3C_k141_222114	186098	187	3	120	Medium-quality	88.89
Sample_T6C_k141_120982_fragment_1	46933	66	17	6	Medium-quality	88.89
Sample_C4C_k141_77872_fragment_1	38755	53	16	2	Medium-quality	88.85
Sample_T6B_k141_20430_fragment_1	38868	53	15	1	Medium-quality	88.84
Sample_C13A_k141_27657	211250	185	1	116	Medium-quality	88.82
Sample_C10A_k141_279851	73111	78	24	2	Medium-quality	88.8

Sample_C5A_k141_175383	152212	133	1	81	Medium-quality	88.75
Sample_T2C_k141_48723	149755	181	2	110	Medium-quality	88.71
Sample_T4B_k141_154950	216506	195	2	117	Medium-quality	88.71
Sample_C11A_k141_65217	87691	85	1	57	Medium-quality	88.67
Sample_C3A_k141_69993	51092	69	19	2	Medium-quality	88.67
Sample_T10A_k141_9385	30609	40	24	1	Medium-quality	88.63
Sample_T6C_k141_113038	219711	175	1	120	Medium-quality	88.54
Sample_C11B_k141_104540	55681	59	6	0	Medium-quality	88.54
Sample_C9C_k141_91879	42593	60	17	4	Medium-quality	88.51
Sample_T17B_k141_32463	51840	60	1	43	Medium-quality	88.5
Sample_T11C_k141_158952	26863	35	15	0	Medium-quality	88.47
Sample_T10A_k141_21939	39897	63	13	1	Medium-quality	88.45
Sample_C3A_k141_140344	34512	42	21	0	Medium-quality	88.43
Sample_C4B_k141_51677_fragment_1	40456	58	28	1	Medium-quality	88.41
Sample_T7C_k141_52536_fragment_1	30193	48	17	1	Medium-quality	88.39
Sample_T10A_k141_170822	57078	49	2	22	Medium-quality	88.37
Sample_T2C_k141_142701	165024	147	1	77	Medium-quality	88.34
Sample_C4C_k141_72210	31835	49	10	3	Medium-quality	88.34
Sample_C2B_k141_177672	117770	127	6	54	Medium-quality	88.3
Sample_C14B_k141_88890	31398	54	18	2	Medium-quality	88.3
Sample_T13B_k141_58453_fragment_1	184331	196	10	26	Medium-quality	88.28
Sample_C13C_k141_109684	29476	48	21	2	Medium-quality	88.22
Sample_T7A_k141_40958	16800	23	2	0	Medium-quality	88.16
Sample_C7B_k141_131548_fragment_4	125660	139	2	16	Medium-quality	88.15
Sample_T2A_k141_244513	36140	51	28	0	Medium-quality	88.15
Sample_T18B_k141_6720	31700	47	12	0	Medium-quality	88.14
Sample_T16A_k141_54996_fragment_1	35464	46	18	0	Medium-quality	88.11
Sample_C10A_k141_260119	38246	39	8	0	Medium-quality	88.06
Sample_C2C_k141_6755_fragment_7	33084	57	16	1	Medium-quality	88.03
Sample_T10A_k141_133475_fragment_1	51916	70	11	5	Medium-quality	88
Sample_C4C_k141_215628	199278	163	2	96	Medium-quality	87.99
Sample_C12C_k141_54102_fragment_4	43865	73	16	5	Medium-quality	87.89
Sample_T7C_k141_88925	39135	71	17	1	Medium-quality	87.78
Sample_C11C_k141_23818	144331	122	1	82	Medium-quality	87.77
Sample_T13B_k141_64817	53234	86	33	0	Medium-quality	87.77
Sample_T11C_k141_142696	38341	57	23	2	Medium-quality	87.74
Sample_C11C_k141_76656_fragment_1	25291	29	9	0	Medium-quality	87.67
Sample_T17B_k141_32649	262226	213	2	139	Medium-quality	87.59
Sample_C1B_k141_113478_fragment_1	40635	54	15	3	Medium-quality	87.56

Sample_C6A_k141_172073_fragment_1	35448	43	10	5	Medium-quality	87.55
Sample_T7A_k141_106905_fragment_1	75106	108	22	17	Medium-quality	87.55
Sample_T7B_k141_115494	40099	55	32	0	Medium-quality	87.52
Sample_C11A_k141_134572	52359	54	0	24	Medium-quality	87.45
Sample_C10A_k141_281652	35612	51	22	2	Medium-quality	87.43
Sample_C13C_k141_62912_fragment_1	35562	40	28	0	Medium-quality	87.38
Sample_C1A_k141_231449	240158	195	2	120	Medium-quality	87.37
Sample_T5A_k141_35926_fragment_2	43900	48	8	4	Medium-quality	87.36
Sample_C9A_k141_115974_fragment_2	37242	53	20	1	Medium-quality	87.33
Sample_C13A_k141_107768	217408	193	1	138	Medium-quality	87.32
Sample_T2A_k141_263098	167512	157	3	83	Medium-quality	87.31
Sample_C3B_k141_170987	5957	8	3	0	Medium-quality	87.26
Sample_T3B_k141_40116	33858	45	15	0	Medium-quality	87.22
Sample_T15C_k141_119522_fragment_1	38636	53	11	6	Medium-quality	87.17
Sample_C4C_k141_183560_fragment_2	30247	49	14	1	Medium-quality	87.14
Sample_C6C_k141_158526_fragment_1	34312	44	15	3	Medium-quality	87.12
Sample_T4B_k141_331659_fragment_2	37053	46	24	0	Medium-quality	87.12
Sample_C6C_k141_118940	32904	45	15	1	Medium-quality	87.11
Sample_T10A_k141_29408_fragment_1	10404	11	2	0	Medium-quality	87.11
Sample_T11A_k141_4046_fragment_1	42309	69	30	4	Medium-quality	86.98
Sample_T16C_k141_94822	142935	123	1	79	Medium-quality	86.92
Sample_T18C_k141_133753	43785	53	9	1	Medium-quality	86.89
Sample_T5C_k141_156283	132286	151	2	80	Medium-quality	86.86
Sample_C3A_k141_35322	36761	45	6	1	Medium-quality	86.84
Sample_T15C_k141_127502_fragment_1	30192	47	21	1	Medium-quality	86.82
Sample_C12B_k141_84086	47000	73	21	0	Medium-quality	86.75
Sample_T4B_k141_71632	179336	168	3	98	Medium-quality	86.73
Sample_C4C_k141_262980_fragment_1	33461	60	15	1	Medium-quality	86.72
Sample_T4B_k141_323424	92462	111	1	58	Medium-quality	86.7
Sample_C12C_k141_121426_fragment_2	41762	53	22	5	Medium-quality	86.7
Sample_T15B_k141_109043	43685	52	0	12	Medium-quality	86.68
Sample_C10A_k141_27147	67360	70	2	27	Medium-quality	86.66
Sample_C3B_k141_89226	32903	50	25	0	Medium-quality	86.63
Sample_T13C_k141_4530	41009	54	20	4	Medium-quality	86.61
Sample_C6B_k141_214583_fragment_1	40150	56	12	7	Medium-quality	86.6
Sample_T4B_k141_325160	35839	57	17	0	Medium-quality	86.57
Sample_C14A_k141_71534	32764	50	16	0	Medium-quality	86.53
Sample_C5B_k141_14543_fragment_1	40097	63	42	0	Medium-quality	86.5
Sample_C9A_k141_63234	40896	62	12	1	Medium-quality	86.47

Sample_C5A_k141_34281_fragment_1	25274	39	14	1	Medium-quality	86.45
Sample_T10A_k141_166560	48168	56	11	7	Medium-quality	86.39
Sample_C14C_k141_125295	30181	52	19	2	Medium-quality	86.38
Sample_C12A_k141_131713_fragment_1	40051	51	16	2	Medium-quality	86.34
Sample_T5C_k141_10805_fragment_1	29611	40	15	0	Medium-quality	86.33
Sample_C3A_k141_11919	38870	49	19	0	Medium-quality	86.28
Sample_C4C_k141_81131	34642	47	23	0	Medium-quality	86.27
Sample_C10A_k141_73594_fragment_1	36586	50	18	2	Medium-quality	86.25
Sample_C4B_k141_250754	37328	60	16	1	Medium-quality	86.23
Sample_C7C_k141_25769	35176	52	14	0	Medium-quality	86.21
Sample_C8A_k141_37849	31768	43	21	0	Medium-quality	86.2
Sample_C11B_k141_231523	69384	116	19	2	Medium-quality	86.19
Sample_T15B_k141_14855	55162	58	1	24	Medium-quality	86.14
Sample_C2B_k141_218921	140285	93	2	51	Medium-quality	86.13
Sample_C4C_k141_114970	45120	56	20	2	Medium-quality	86.1
Sample_T11A_k141_49161_fragment_1	39407	54	16	7	Medium-quality	86.09
Sample_T4A_k141_106576	5565	10	0	0	Medium-quality	86.08
Sample_C11C_k141_87481	38164	43	22	3	Medium-quality	86.05
Sample_T10B_k141_49077	110701	125	5	16	Medium-quality	86.05
Sample_C13A_k141_96335_fragment_1	28309	31	9	2	Medium-quality	85.99
Sample_T6A_k141_243508	57438	51	1	23	Medium-quality	85.93
Sample_C5A_k141_160793	348538	366	3	226	Medium-quality	85.91
Sample_T17A_k141_19751	44978	52	12	8	Medium-quality	85.89
Sample_C12A_k141_44696	204161	194	1	155	Medium-quality	85.84
Sample_T3A_k141_60888	200268	168	1	112	Medium-quality	85.79
Sample_C9A_k141_1646_fragment_1	39654	50	26	3	Medium-quality	85.76
Sample_T6C_k141_160069_fragment_2	51996	69	10	2	Medium-quality	85.75
Sample_C11C_k141_117699	129447	164	22	2	Medium-quality	85.66
Sample_C3A_k141_140205	196758	144	1	111	Medium-quality	85.63
Sample_C12A_k141_208917_fragment_2	49575	65	29	5	Medium-quality	85.62
Sample_T3B_k141_34756	30116	39	13	0	Medium-quality	85.6
Sample_T18B_k141_28937_fragment_1	47646	56	11	1	Medium-quality	85.58
Sample_T3B_k141_41799	39755	42	1	9	Medium-quality	85.51
Sample_C4B_k141_187335	55230	88	28	0	Medium-quality	85.5
Sample_C3C_k141_115073_fragment_1	28774	45	17	0	Medium-quality	85.48
Sample_C10A_k141_59908	205564	207	3	123	Medium-quality	85.46
Sample_C2B_k141_163508_fragment_2	38233	62	24	2	Medium-quality	85.46
Sample_T8B_k141_11266	48490	69	26	8	Medium-quality	85.35
Sample_T10A_k141_169746	30265	33	12	0	Medium-quality	85.23

Sample_T14B_k141_186529	134610	147	3	49	Medium-quality	85.14
Sample_T16A_k141_7518_fragment_1	49740	65	30	3	Medium-quality	85.06
Sample_C10B_k141_59051	67988	83	2	8	Medium-quality	85.01
Sample_C10B_k141_46522	16023	22	7	0	Medium-quality	84.97
Sample_T16C_k141_124125	130728	145	14	1	Medium-quality	84.96
Sample_C14A_k141_90804_fragment_1	42803	54	7	3	Medium-quality	84.95
Sample_C10A_k141_60977_fragment_1	33304	61	21	1	Medium-quality	84.94
Sample_T8C_k141_22209	34451	45	14	0	Medium-quality	84.94
Sample_T2A_k141_18354	29450	19	0	8	Medium-quality	84.93
Sample_C6C_k141_65181	32029	50	13	1	Medium-quality	84.77
Sample_C10C_k141_107064	31186	49	28	2	Medium-quality	84.76
Sample_T12C_k141_25550	61386	77	11	4	Medium-quality	84.73
Sample_C3C_k141_224145	50194	75	14	3	Medium-quality	84.73
Sample_T12A_k141_73467	47499	58	9	3	Medium-quality	84.69
Sample_T3B_k141_99384	70050	86	17	8	Medium-quality	84.53
Sample_C1B_k141_6969	5251	10	3	0	Medium-quality	84.52
Sample_C7C_k141_43449_fragment_2	47359	67	29	2	Medium-quality	84.52
Sample_C11A_k141_55792_fragment_1	48663	84	17	0	Medium-quality	84.48
Sample_C11B_k141_128404_fragment_1	49426	67	18	9	Medium-quality	84.48
Sample_T8C_k141_53457_fragment_2	47508	68	11	3	Medium-quality	84.46
Sample_T3C_k141_53772_fragment_2	33069	48	15	0	Medium-quality	84.33
Sample_T11A_k141_59250_fragment_1	32495	59	15	0	Medium-quality	84.31
Sample_C11C_k141_101773	37592	63	23	0	Medium-quality	84.23
Sample_T15B_k141_111690_fragment_1	31848	51	18	0	Medium-quality	84.22
Sample_T15B_k141_106747	16114	25	8	0	Medium-quality	84.16
Sample_T7B_k141_63587_fragment_1	75024	131	11	4	Medium-quality	84.14
Sample_T8B_k141_148465_fragment_3	33962	46	14	0	Medium-quality	84.14
Sample_T12A_k141_156836_fragment_1	25469	34	12	1	Medium-quality	84.07
Sample_C6C_k141_52659	15586	16	3	9	Medium-quality	84.03
Sample_T8A_k141_166198	15141	12	3	2	Medium-quality	83.99
Sample_C13B_k141_38365_fragment_1	51567	59	4	4	Medium-quality	83.99
Sample_T11C_k141_147097_fragment_1	50981	72	25	0	Medium-quality	83.98
Sample_T18A_k141_52670	42314	58	26	4	Medium-quality	83.93
Sample_T11A_k141_7810_fragment_1	37603	57	14	1	Medium-quality	83.92
Sample_C4A_k141_171215_fragment_2	41905	60	27	2	Medium-quality	83.91
Sample_C6C_k141_224960	55855	61	26	3	Medium-quality	83.83
Sample_T5C_k141_27285	31625	43	15	0	Medium-quality	83.77
Sample_C10A_k141_337509	73540	79	2	42	Medium-quality	83.7
Sample_C8B_k141_169530	46649	58	9	1	Medium-quality	83.69

Sample_T4C_k141_27107	31024	46	21	0	Medium-quality	83.67
Sample_C9C_k141_98793	27148	46	12	0	Medium-quality	83.64
Sample_T4B_k141_240169_fragment_1	34134	51	24	0	Medium-quality	83.63
Sample_T11C_k141_87733	78811	74	1	62	Medium-quality	83.56
Sample_T13B_k141_85630	33043	50	16	0	Medium-quality	83.56
Sample_T10B_k141_21815_fragment_3	38068	49	20	2	Medium-quality	83.54
Sample_T4B_k141_128624	92490	73	1	41	Medium-quality	83.52
Sample_T2C_k141_158847	34933	39	6	0	Medium-quality	83.48
Sample_T14B_k141_12180_fragment_2	37367	60	13	2	Medium-quality	83.41
Sample_T2B_k141_107999	147610	141	1	90	Medium-quality	83.4
Sample_T13B_k141_177852	66275	76	3	9	Medium-quality	83.38
Sample_C2B_k141_178224	73869	82	2	24	Medium-quality	83.37
Sample_C8A_k141_87022	39472	39	6	2	Medium-quality	83.33
Sample_T13C_k141_12206	97002	100	3	16	Medium-quality	83.3
Sample_T2C_k141_60541	37292	69	12	1	Medium-quality	83.3
Sample_T6B_k141_32849	145074	146	38	5	Medium-quality	83.26
Sample_C14C_k141_169292	34612	44	20	4	Medium-quality	83.21
Sample_T14B_k141_9221	48442	43	1	22	Medium-quality	83.14
Sample_C11C_k141_11664	40147	46	12	1	Medium-quality	83.12
Sample_C10C_k141_73727	26920	44	14	0	Medium-quality	83.1
Sample_C10C_k141_96331_fragment_1	37235	60	14	2	Medium-quality	83.06
Sample_C5A_k141_249565	37765	51	22	5	Medium-quality	83.06
Sample_C13A_k141_50305	41451	39	1	34	Medium-quality	83.03
Sample_T6C_k141_63706_fragment_1	29452	44	19	0	Medium-quality	82.96
Sample_T4B_k141_155361	134100	150	1	68	Medium-quality	82.87
Sample_C2B_k141_154859	118185	114	2	70	Medium-quality	82.73
Sample_T14A_k141_98089	128516	117	1	94	Medium-quality	82.71
Sample_C10A_k141_121265	33614	41	19	2	Medium-quality	82.62
Sample_C10A_k141_57239	70115	89	19	14	Medium-quality	82.61
Sample_T6C_k141_214111_fragment_1	62901	78	9	9	Medium-quality	82.54
Sample_T5C_k141_61485_fragment_1	37056	53	14	2	Medium-quality	82.53
Sample_T7A_k141_132870	42232	62	6	1	Medium-quality	82.53
Sample_C10A_k141_63337	30618	41	16	0	Medium-quality	82.47
Sample_C12C_k141_140447_fragment_1	48543	62	33	1	Medium-quality	82.38
Sample_T7A_k141_195012_fragment_1	28866	50	16	0	Medium-quality	82.36
Sample_T10A_k141_28106_fragment_1	53702	79	33	0	Medium-quality	82.35
Sample_T12C_k141_126141_fragment_1	40236	33	2	8	Medium-quality	82.28
Sample_T12A_k141_69089	180482	178	21	1	Medium-quality	82.27
Sample_C12A_k141_205787	194768	168	2	13	Medium-quality	82.25

Sample_T17A_k141_18309_fragment_1	86688	97	24	13	Medium-quality	82.21
Sample_C9A_k141_112775	38351	53	22	2	Medium-quality	82.15
Sample_T17B_k141_5940	42657	59	28	1	Medium-quality	82.1
Sample_C12C_k141_134397	39730	45	8	2	Medium-quality	82.08
Sample_C1B_k141_14145	158911	136	1	91	Medium-quality	82.04
Sample_C6A_k141_126198	45903	56	10	9	Medium-quality	81.95
Sample_C5C_k141_48449	38344	69	42	0	Medium-quality	81.94
Sample_C13A_k141_11688	15826	19	9	0	Medium-quality	81.9
Sample_T17B_k141_8647	33619	38	11	2	Medium-quality	81.86
Sample_C14C_k141_114371	27687	39	16	0	Medium-quality	81.79
Sample_C1B_k141_5886	43703	47	19	4	Medium-quality	81.73
Sample_C3A_k141_119143_fragment_1	24832	31	15	4	Medium-quality	81.68
Sample_T12C_k141_68933_fragment_1	30163	42	22	1	Medium-quality	81.65
Sample_T6B_k141_184640	31976	52	12	1	Medium-quality	81.56
Sample_C4C_k141_194433_fragment_1	41718	60	14	4	Medium-quality	81.53
Sample_T2C_k141_151682	35101	57	18	0	Medium-quality	81.5
Sample_T11B_k141_104741	36803	34	2	14	Medium-quality	81.48
Sample_C10A_k141_86233_fragment_1	36751	47	26	0	Medium-quality	81.47
Sample_C13B_k141_56957	34071	53	22	0	Medium-quality	81.46
Sample_C5A_k141_161307_fragment_1	35217	48	17	2	Medium-quality	81.46
Sample_T5C_k141_181665	36971	56	14	0	Medium-quality	81.45
Sample_T12A_k141_75569_fragment_1	39833	56	19	1	Medium-quality	81.44
Sample_T10B_k141_159511	29136	41	13	0	Medium-quality	81.4
Sample_T1A_k141_28748	50622	71	19	5	Medium-quality	81.4
Sample_C2A_k141_119617	142410	190	23	3	Medium-quality	81.33
Sample_C4C_k141_229447	29649	45	19	0	Medium-quality	81.31
Sample_T6B_k141_115767_fragment_2	32840	27	8	0	Medium-quality	81.26
Sample_C2B_k141_228085	136858	145	1	91	Medium-quality	81.25
Sample_C10A_k141_324409	40848	41	12	6	Medium-quality	81.19
Sample_T11A_k141_109837	29783	35	2	4	Medium-quality	81.18
Sample_C12A_k141_65091_fragment_1	42960	63	11	8	Medium-quality	81.15
Sample_T13C_k141_89034_fragment_2	58709	72	16	5	Medium-quality	81.13
Sample_T12B_k141_153615_fragment_1	40258	54	15	0	Medium-quality	81.06
Sample_C6B_k141_190744_fragment_1	47929	59	14	7	Medium-quality	81.03
Sample_T13B_k141_69417	37627	31	0	23	Medium-quality	81.01
Sample_T12B_k141_83438	34716	61	16	0	Medium-quality	80.98
Sample_C12A_k141_32565	26163	31	14	1	Medium-quality	80.97
Sample_C2B_k141_292505_fragment_1	39769	58	14	4	Medium-quality	80.97
Sample_C10A_k141_69985_fragment_1	34698	33	3	0	Medium-quality	80.89

Sample_T5C_k141_101017_fragment_2	39533	51	17	0	Medium-quality	80.83
Sample_T17C_k141_1816	50332	74	19	6	Medium-quality	80.82
Sample_T4B_k141_174096	46599	83	10	1	Medium-quality	80.79
Sample_T8A_k141_215722	36472	41	6	4	Medium-quality	80.77
Sample_T4C_k141_39098_fragment_1	13731	17	7	0	Medium-quality	80.65
Sample_C4B_k141_14334	125259	126	1	108	Medium-quality	80.61
Sample_T5B_k141_1377	44866	66	11	1	Medium-quality	80.55
Sample_T2A_k141_35171	13122	16	0	0	Medium-quality	80.53
Sample_T15C_k141_16856_fragment_1	31735	53	18	2	Medium-quality	80.53
Sample_T14C_k141_25853	40078	36	8	1	Medium-quality	80.47
Sample_C8C_k141_55607	54936	50	0	11	Medium-quality	80.46
Sample_C11A_k141_18491_fragment_1	30693	39	17	0	Medium-quality	80.46
Sample_C2A_k141_210146	63707	89	9	1	Medium-quality	80.41
Sample_T4C_k141_20057_fragment_1	49359	66	11	0	Medium-quality	80.37
Sample_T8A_k141_138708	119538	123	1	30	Medium-quality	80.36
Sample_T13B_k141_174719	31971	46	3	1	Medium-quality	80.35
Sample_C1A_k141_366	153050	144	2	83	Medium-quality	80.3
Sample_T3A_k141_52358	29940	23	1	7	Medium-quality	80.25
Sample_C10B_k141_128914	10834	14	0	1	Medium-quality	80.18
Sample_T10C_k141_34738	54764	44	0	9	Medium-quality	80.01
Sample_C9C_k141_3370	204415	197	2	107	Medium-quality	80
Sample_T3B_k141_47318	12352	10	1	0	Medium-quality	79.94
Sample_T6C_k141_6304	59050	94	18	5	Medium-quality	79.87
Sample_C4B_k141_107345	121050	131	3	66	Medium-quality	79.79
Sample_C10A_k141_140301	36124	46	7	0	Medium-quality	79.78
Sample_T15C_k141_120081_fragment_1	25455	35	15	1	Medium-quality	79.77
Sample_T18A_k141_73832	197886	172	1	118	Medium-quality	79.75
Sample_T16C_k141_40226_fragment_1	33482	42	18	0	Medium-quality	79.75
Sample_C11C_k141_573_fragment_1	180410	220	42	6	Medium-quality	79.74
Sample_T17B_k141_4187_fragment_1	57273	88	31	1	Medium-quality	79.71
Sample_C7A_k141_170291	31831	31	1	8	Medium-quality	79.68
Sample_T4B_k141_373737	33250	46	18	0	Medium-quality	79.66
Sample_T13C_k141_24893_fragment_1	32426	44	21	1	Medium-quality	79.57
Sample_C11A_k141_120496_fragment_2	37578	51	20	2	Medium-quality	79.56
Sample_T4B_k141_194013	435157	407	1	248	Medium-quality	79.47
Sample_T4B_k141_183365	33172	40	1	20	Medium-quality	79.44
Sample_T4B_k141_112863	125036	137	3	76	Medium-quality	79.42
Sample_T4B_k141_267836	17784	24	7	0	Medium-quality	79.42
Sample_T2A_k141_18033	87949	49	1	23	Medium-quality	79.41

Sample_C6B_k141_140822	32949	46	10	3	Medium-quality	79.39
Sample_T8C_k141_71984_fragment_1	44118	68	17	4	Medium-quality	79.38
Sample_T11B_k141_160350_fragment_3	43160	48	23	2	Medium-quality	79.33
Sample_C3A_k141_42337_fragment_1	73263	96	14	6	Medium-quality	79.31
Sample_T12B_k141_59025	62326	84	26	9	Medium-quality	79.22
Sample_T4B_k141_16587	37857	37	4	3	Medium-quality	79.22
Sample_T4C_k141_113518_fragment_1	31617	49	18	0	Medium-quality	79.22
Sample_C12C_k141_120893	56179	56	2	13	Medium-quality	79.19
Sample_C4C_k141_243864	35860	50	15	2	Medium-quality	79.18
Sample_C7A_k141_152339	107475	116	31	1	Medium-quality	79.15
Sample_C10A_k141_111966_fragment_1	31067	42	22	0	Medium-quality	79.11
Sample_T13C_k141_5953_fragment_1	39751	50	12	3	Medium-quality	79.1
Sample_C5B_k141_47456	13935	19	7	0	Medium-quality	78.98
Sample_T11A_k141_118130_fragment_1	40123	41	4	2	Medium-quality	78.97
Sample_T8B_k141_140787	36745	49	16	0	Medium-quality	78.88
Sample_C4B_k141_74879	27188	39	16	2	Medium-quality	78.87
Sample_T12B_k141_120554	32964	24	1	6	Medium-quality	78.82
Sample_T2C_k141_275171	29845	43	5	0	Medium-quality	78.73
Sample_T15A_k141_49222	61939	57	0	18	Medium-quality	78.69
Sample_T11C_k141_83600	117362	134	1	61	Medium-quality	78.65
Sample_C6B_k141_94597	35944	54	13	1	Medium-quality	78.61
Sample_T14A_k141_185058_fragment_1	30869	38	25	1	Medium-quality	78.59
Sample_T12B_k141_27561	35318	40	3	1	Medium-quality	78.56
Sample_C5A_k141_71891_fragment_1	35459	49	20	3	Medium-quality	78.54
Sample_C5C_k141_1863	22601	33	10	1	Medium-quality	78.35
Sample_T10C_k141_46709_fragment_1	45147	75	17	0	Medium-quality	78.3
Sample_C1C_k141_102365	28374	31	14	1	Medium-quality	78.26
Sample_C2A_k141_252630	112495	133	41	6	Medium-quality	78.24
Sample_T18C_k141_89176	158511	142	3	65	Medium-quality	78.23
Sample_T6C_k141_89971_fragment_2	36307	47	14	8	Medium-quality	78.18
Sample_C4C_k141_195787	427943	388	1	240	Medium-quality	78.16
Sample_C2A_k141_118054	47234	66	15	3	Medium-quality	78.13
Sample_T12A_k141_194457_fragment_3	12651	15	3	4	Medium-quality	78.12
Sample_T17A_k141_20651	38906	53	24	0	Medium-quality	78.12
Sample_T16A_k141_101578_fragment_1	36861	52	19	3	Medium-quality	78.11
Sample_C4A_k141_262734	53935	88	28	0	Medium-quality	78.06
Sample_T6A_k141_84179	35838	48	4	0	Medium-quality	78.04
Sample_T17A_k141_28883_fragment_1	59509	84	14	5	Medium-quality	78
Sample_T3C_k141_19558	135383	120	1	33	Medium-quality	77.97

Sample_T2B_k141_122137	115919	95	1	38	Medium-quality	77.92
Sample_T10C_k141_81274	50364	67	12	2	Medium-quality	77.9
Sample_T6C_k141_2994_fragment_1	49166	58	25	2	Medium-quality	77.89
Sample_C3C_k141_145838	42760	43	0	18	Medium-quality	77.88
Sample_T4B_k141_57557_fragment_2	37631	54	16	1	Medium-quality	77.86
Sample_C9A_k141_46447_fragment_1	52466	57	27	5	Medium-quality	77.83
Sample_T5C_k141_71139_fragment_1	45340	61	16	7	Medium-quality	77.75
Sample_C10A_k141_93891_fragment_1	48544	41	5	1	Medium-quality	77.72
Sample_T7B_k141_86518_fragment_1	30900	45	8	0	Medium-quality	77.66
Sample_T5B_k141_57714	41081	48	11	4	Medium-quality	77.52
Sample_T13B_k141_1190	69790	74	3	7	Medium-quality	77.41
Sample_T11A_k141_131845	127273	88	1	66	Medium-quality	77.4
Sample_T12B_k141_151843	127264	97	1	79	Medium-quality	77.39
Sample_T1C_k141_59558	71177	117	15	9	Medium-quality	77.38
Sample_T17C_k141_13381_fragment_1	35438	43	13	0	Medium-quality	77.35
Sample_T2A_k141_100529	111174	135	2	63	Medium-quality	77.34
Sample_T5C_k141_94002	51695	54	1	26	Medium-quality	77.33
Sample_T4B_k141_301066_fragment_1	35232	46	18	1	Medium-quality	77.32
Sample_T18C_k141_128427	114340	163	8	10	Medium-quality	77.31
Sample_C13C_k141_69201	75811	133	10	1	Medium-quality	77.19
Sample_T11A_k141_80553	42648	61	15	5	Medium-quality	77.11
Sample_T17B_k141_6674	38473	24	1	13	Medium-quality	77.06
Sample_C12B_k141_18312	57022	58	17	12	Medium-quality	77.06
Sample_C9B_k141_15744	197409	132	2	87	Medium-quality	77.02
Sample_C12A_k141_65755	46092	50	0	22	Medium-quality	76.99
Sample_T3C_k141_99723	11278	20	3	0	Medium-quality	76.98
Sample_C3A_k141_31991_fragment_1	35185	49	33	3	Medium-quality	76.95
Sample_T13B_k141_4613	44256	57	37	4	Medium-quality	76.93
Sample_T13A_k141_57369	29670	41	15	1	Medium-quality	76.92
Sample_T13A_k141_98666_fragment_1	27999	40	24	1	Medium-quality	76.92
Sample_C1C_k141_55278_fragment_1	35716	47	23	1	Medium-quality	76.91
Sample_T5B_k141_27809_fragment_1	46911	56	13	1	Medium-quality	76.9
Sample_T14B_k141_92194_fragment_2	119036	135	6	10	Medium-quality	76.83
Sample_T7A_k141_81809	11179	17	1	0	Medium-quality	76.81
Sample_T1C_k141_34747	58789	96	11	2	Medium-quality	76.8
Sample_C3C_k141_39928	39862	55	17	1	Medium-quality	76.78
Sample_T4C_k141_27983	31714	44	13	2	Medium-quality	76.75
Sample_C12A_k141_34700_fragment_1	29385	45	18	3	Medium-quality	76.71
Sample_T2A_k141_266073	80246	86	3	48	Medium-quality	76.68

Sample_T7C_k141_69560	113990	97	1	67	Medium-quality	76.63
Sample_T7B_k141_99177	27661	45	7	0	Medium-quality	76.62
Sample_T8B_k141_4821	53273	39	0	12	Medium-quality	76.58
Sample_T2C_k141_259081_fragment_1	29058	32	23	0	Medium-quality	76.55
Sample_T15B_k141_110519	52465	57	1	11	Medium-quality	76.45
Sample_T12A_k141_177437	67308	93	8	3	Medium-quality	76.43
Sample_C10B_k141_24982	16832	21	7	0	Medium-quality	76.35
Sample_T10A_k141_103577	22303	30	13	1	Medium-quality	76.28
Sample_T14A_k141_237174	27944	46	10	1	Medium-quality	76.25
Sample_T4B_k141_217730_fragment_1	34675	51	15	1	Medium-quality	76.24
Sample_T16C_k141_132757_fragment_1	52460	69	28	3	Medium-quality	76.16
Sample_T6C_k141_157077	48678	46	8	4	Medium-quality	76.16
Sample_C7A_k141_7390	47103	72	25	3	Medium-quality	76.07
Sample_C13A_k141_85582	120692	133	2	78	Medium-quality	76.06
Sample_C13B_k141_29310	17665	27	8	0	Medium-quality	76.05
Sample_C2C_k141_244821_fragment_1	58322	72	21	6	Medium-quality	76.05
Sample_C10A_k141_68513_fragment_1	35604	45	8	2	Medium-quality	76.04
Sample_T13C_k141_98645_fragment_4	47322	82	20	1	Medium-quality	76.03
Sample_C13B_k141_20500	24673	35	11	4	Medium-quality	76.02
Sample_C11B_k141_217575	32755	48	14	0	Medium-quality	75.94
Sample_C3A_k141_20631_fragment_1	37427	44	6	4	Medium-quality	75.87
Sample_T14B_k141_53791	200610	174	3	115	Medium-quality	75.83
Sample_T13C_k141_143041_fragment_2	30197	48	19	1	Medium-quality	75.82
Sample_C6B_k141_211260_fragment_1	40110	52	9	2	Medium-quality	75.78
Sample_C13A_k141_6576_fragment_1	33238	43	7	1	Medium-quality	75.75
Sample_T4B_k141_78224	21679	17	1	10	Medium-quality	75.57
Sample_T11A_k141_148520	25419	47	22	0	Medium-quality	75.57
Sample_T6C_k141_167684	33901	48	2	2	Medium-quality	75.52
Sample_C4A_k141_234710_fragment_1	87991	94	27	10	Medium-quality	75.46
Sample_C13C_k141_30080_fragment_1	31219	48	14	0	Medium-quality	75.42
Sample_T15A_k141_31552	186976	139	3	107	Medium-quality	75.32
Sample_T7C_k141_140099	43969	57	9	1	Medium-quality	75.26
Sample_T2A_k141_27416_fragment_1	112662	120	40	6	Medium-quality	75.25
Sample_T6B_k141_57709	57828	62	1	41	Medium-quality	75.23
Sample_C6A_k141_59450_fragment_2	54885	83	14	6	Medium-quality	75.18
Sample_T12A_k141_178227	37475	37	8	1	Medium-quality	75.12
Sample_C4B_k141_113003	33498	53	22	0	Medium-quality	75.07
Sample_T5A_k141_37312_fragment_1	30913	51	18	0	Medium-quality	75.04
Sample_C3A_k141_66314	10162	14	1	0	Medium-quality	75.01

Sample_C6B_k141_200782_fragment_1	28162	43	16	0	Medium-quality	74.89
Sample_T12C_k141_68639	32948	40	1	2	Medium-quality	74.85
Sample_T8C_k141_71518	263034	244	1	188	Medium-quality	74.84
Sample_C8B_k141_100786	27067	36	16	0	Medium-quality	74.84
Sample_T8B_k141_266223	21445	22	3	1	Medium-quality	74.75
Sample_C2A_k141_238760	87946	104	29	2	Medium-quality	74.73
Sample_T10C_k141_70278_fragment_1	33615	44	15	0	Medium-quality	74.68
Sample_C1C_k141_128162_fragment_1	39856	54	8	0	Medium-quality	74.65
Sample_C11A_k141_74514_fragment_1	42502	58	19	4	Medium-quality	74.61
Sample_T6B_k141_53521_fragment_3	46609	80	30	1	Medium-quality	74.61
Sample_T11A_k141_58212	26918	40	18	1	Medium-quality	74.6
Sample_C5A_k141_29955	57486	54	0	12	Medium-quality	74.57
Sample_C6C_k141_49147	26457	38	7	1	Medium-quality	74.56
Sample_T4C_k141_14582_fragment_1	29278	45	10	0	Medium-quality	74.54
Sample_C10A_k141_56652	120497	111	1	81	Medium-quality	74.46
Sample_T8A_k141_225413	28404	43	2	0	Medium-quality	74.46
Sample_C3B_k141_219836_fragment_1	39336	56	29	4	Medium-quality	74.45
Sample_C2C_k141_237008	116158	150	29	4	Medium-quality	74.41
Sample_C4C_k141_142055	27975	41	15	1	Medium-quality	74.41
Sample_T4B_k141_180609	49723	58	1	25	Medium-quality	74.38
Sample_C13C_k141_107247	42526	57	5	4	Medium-quality	74.24
Sample_C4A_k141_187943_fragment_1	33676	56	27	0	Medium-quality	74.22
Sample_T15C_k141_87649	30028	37	11	0	Medium-quality	74.2
Sample_C4A_k141_142275	33529	39	10	2	Medium-quality	74.13
Sample_C2C_k141_223668	27228	49	11	4	Medium-quality	74.12
Sample_C5A_k141_21860_fragment_1	41775	51	7	1	Medium-quality	74.12
Sample_T5A_k141_49139_fragment_3	52422	65	13	5	Medium-quality	74.11
Sample_T17A_k141_27997_fragment_2	25463	40	16	0	Medium-quality	74.09
Sample_C11B_k141_157394	68616	88	21	4	Medium-quality	74.06
Sample_T2B_k141_318772	138245	139	1	65	Medium-quality	74.01
Sample_C10C_k141_165136	46009	55	21	3	Medium-quality	73.98
Sample_T7C_k141_62215_fragment_1	54990	92	16	4	Medium-quality	73.94
Sample_C3A_k141_244074	16766	21	3	0	Medium-quality	73.91
Sample_T13B_k141_31917	159299	144	2	94	Medium-quality	73.9
Sample_T4C_k141_45414	109900	96	1	57	Medium-quality	73.88
Sample_T2A_k141_83039	10136	11	0	2	Medium-quality	73.84
Sample_T15A_k141_154913	35485	46	12	0	Medium-quality	73.82
Sample_C11C_k141_137766_fragment_1	34619	51	8	0	Medium-quality	73.74
Sample_T15C_k141_115204	77325	102	26	1	Medium-quality	73.7

Sample_T15C_k141_136512	61878	58	1	15	Medium-quality	73.68
Sample_C5A_k141_32283_fragment_1	36789	63	18	1	Medium-quality	73.65
Sample_T4B_k141_131030_fragment_1	35519	61	14	0	Medium-quality	73.58
Sample_T1B_k141_31370	25494	16	0	6	Medium-quality	73.52
Sample_C13B_k141_60867	37328	49	2	3	Medium-quality	73.52
Sample_C6A_k141_62987_fragment_1	33893	49	15	4	Medium-quality	73.51
Sample_T13B_k141_66590	117472	130	2	107	Medium-quality	73.5
Sample_C11C_k141_172842_fragment_1	34478	46	21	0	Medium-quality	73.5
Sample_T15A_k141_155425_fragment_1	47742	41	4	3	Medium-quality	73.47
Sample_T3C_k141_124248	31675	44	24	2	Medium-quality	73.44
Sample_T8B_k141_277897	109132	88	1	33	Medium-quality	73.36
Sample_T8C_k141_6692_fragment_1	34099	43	19	0	Medium-quality	73.25
Sample_T5A_k141_17900	25388	33	16	0	Medium-quality	73.24
Sample_T5C_k141_98453	26000	28	20	0	Medium-quality	73.15
Sample_C1A_k141_132159	134831	121	1	90	Medium-quality	73.13
Sample_C13A_k141_3640	28903	40	16	2	Medium-quality	73.11
Sample_T7A_k141_91157	29486	41	5	0	Medium-quality	73.1
Sample_T8C_k141_1695	27587	41	9	2	Medium-quality	73.09
Sample_C9B_k141_62585_fragment_1	52736	68	23	10	Medium-quality	73.08
Sample_C13C_k141_37609_fragment_3	42701	63	24	2	Medium-quality	72.93
Sample_C12B_k141_93914	108478	101	1	46	Medium-quality	72.92
Sample_C11A_k141_123683	59299	59	10	5	Medium-quality	72.92
Sample_C6C_k141_67134	36162	41	10	0	Medium-quality	72.87
Sample_T8C_k141_75613	63604	70	2	9	Medium-quality	72.84
Sample_T5B_k141_20444	30047	27	2	2	Medium-quality	72.84
Sample_C10B_k141_73255	27343	44	15	0	Medium-quality	72.83
Sample_C10A_k141_150640	108354	158	12	9	Medium-quality	72.81
Sample_C10A_k141_216407	13369	12	1	3	Medium-quality	72.8
Sample_T16B_k141_168011	41925	52	2	5	Medium-quality	72.76
Sample_T8B_k141_121576	56115	61	1	11	Medium-quality	72.7
Sample_C8A_k141_79685	23987	31	17	1	Medium-quality	72.7
Sample_T10A_k141_124490	28277	34	11	0	Medium-quality	72.67
Sample_T2B_k141_199148_fragment_1	32116	35	6	3	Medium-quality	72.64
Sample_C2C_k141_84123	107982	106	1	82	Medium-quality	72.59
Sample_T15C_k141_27034	291100	271	9	165	Medium-quality	72.57
Sample_T14A_k141_73625_fragment_1	58433	77	5	23	Medium-quality	72.57
Sample_T8A_k141_41143_fragment_2	37942	56	19	2	Medium-quality	72.56
Sample_C2B_k141_240195	105617	126	2	94	Medium-quality	72.55
Sample_C13B_k141_20660_fragment_2	52412	63	12	2	Medium-quality	72.53

Sample_T15B_k141_124710_fragment_1	70584	66	24	6	Medium-quality	72.49
Sample_T12A_k141_4045	42118	53	20	1	Medium-quality	72.44
Sample_T15A_k141_97405_fragment_1	49773	63	3	3	Medium-quality	72.4
Sample_C2C_k141_133539	39132	63	25	2	Medium-quality	72.29
Sample_T12A_k141_113291_fragment_1	27745	38	7	0	Medium-quality	72.28
Sample_C11A_k141_106062_fragment_1	33476	50	2	2	Medium-quality	72.24
Sample_C11A_k141_159685	71411	68	1	40	Medium-quality	72.21
Sample_C5A_k141_144148_fragment_2	23040	33	14	1	Medium-quality	72.21
Sample_T10A_k141_83827_fragment_1	39891	55	41	1	Medium-quality	72.18
Sample_C4C_k141_266338	29369	39	11	0	Medium-quality	72.06
Sample_T18B_k141_141223	29380	40	17	0	Medium-quality	72.06
Sample_T5C_k141_54538_fragment_2	39702	57	8	3	Medium-quality	72.03
Sample_C11B_k141_36149	34173	40	20	1	Medium-quality	72.02
Sample_C2C_k141_196281	27286	42	12	0	Medium-quality	72.02
Sample_C13A_k141_66427	9831	13	4	0	Medium-quality	71.97
Sample_T14C_k141_92994	24832	26	19	0	Medium-quality	71.91
Sample_C10A_k141_225427	53625	49	2	14	Medium-quality	71.9
Sample_C10A_k141_107905_fragment_2	51833	66	13	2	Medium-quality	71.9
Sample_T16A_k141_27181_fragment_1	46264	56	13	2	Medium-quality	71.89
Sample_C9B_k141_26824	102132	118	2	39	Medium-quality	71.87
Sample_C3C_k141_191221	132486	121	1	91	Medium-quality	71.86
Sample_C10A_k141_32819	111638	96	1	69	Medium-quality	71.85
Sample_C1B_k141_128660	39272	58	14	1	Medium-quality	71.81
Sample_C6C_k141_142243	90954	95	29	13	Medium-quality	71.79
Sample_T14A_k141_141635	63288	75	10	8	Medium-quality	71.77
Sample_C3A_k141_29431	111505	92	1	62	Medium-quality	71.76
Sample_C4A_k141_96999	48362	44	1	12	Medium-quality	71.6
Sample_T10C_k141_26859	20522	21	0	2	Medium-quality	71.53
Sample_T10A_k141_5342_fragment_1	53920	75	12	11	Medium-quality	71.52
Sample_T18C_k141_24890	39588	54	6	3	Medium-quality	71.44
Sample_T14C_k141_98879	38107	60	8	4	Medium-quality	71.34
Sample_T7A_k141_171912_fragment_4	33708	44	16	3	Medium-quality	71.27
Sample_C12B_k141_66230	70206	84	2	19	Medium-quality	71.23
Sample_T3C_k141_108881	25918	38	13	1	Medium-quality	71.18
Sample_T2C_k141_14090_fragment_2	33690	46	19	1	Medium-quality	71.16
Sample_T13A_k141_94757	27990	32	8	0	Medium-quality	71.13
Sample_T4C_k141_100391	169814	206	11	7	Medium-quality	71.13
Sample_T3A_k141_5604	28733	44	13	0	Medium-quality	71.11
Sample_C11B_k141_153135	33054	32	1	5	Medium-quality	71.1

Sample_C10A_k141_53561_fragment_1	33994	43	14	0	Medium-quality	71.1
Sample_T11A_k141_31932_fragment_1	45531	52	24	8	Medium-quality	71.08
Sample_T13B_k141_85617_fragment_1	27102	39	11	0	Medium-quality	71.07
Sample_C2C_k141_288348	32062	54	15	0	Medium-quality	71.04
Sample_T8C_k141_153951	26236	37	9	1	Medium-quality	71.01
Sample_C2C_k141_178798	56255	83	8	1	Medium-quality	71
Sample_C1C_k141_58204	165593	139	1	85	Medium-quality	70.94
Sample_T7B_k141_81550	39547	36	1	8	Medium-quality	70.92
Sample_C13B_k141_60371	46755	72	25	0	Medium-quality	70.92
Sample_C1C_k141_48362	53656	43	0	12	Medium-quality	70.85
Sample_T17A_k141_12249_fragment_1	37824	46	3	3	Medium-quality	70.82
Sample_C7A_k141_9862	8987	15	6	0	Medium-quality	70.77
Sample_T6A_k141_35720	46255	61	11	2	Medium-quality	70.74
Sample_C11B_k141_202094	106876	133	17	4	Medium-quality	70.71
Sample_C12C_k141_207242	33988	42	15	0	Medium-quality	70.68
Sample_C4C_k141_213382	25428	41	13	0	Medium-quality	70.68
Sample_T11B_k141_88766	17877	27	14	0	Medium-quality	70.66
Sample_T2A_k141_91576	182592	150	1	101	Medium-quality	70.62
Sample_T16C_k141_32061	30520	43	16	0	Medium-quality	70.6
Sample_C6A_k141_47244	38261	46	10	2	Medium-quality	70.58
Sample_T12A_k141_34901	82519	79	2	23	Medium-quality	70.56
Sample_T1C_k141_53403_fragment_1	26383	31	20	0	Medium-quality	70.53
Sample_T11B_k141_19768	33507	44	9	2	Medium-quality	70.48
Sample_T4A_k141_68123	93747	69	2	45	Medium-quality	70.45
Sample_T10A_k141_77740_fragment_2	22150	31	23	1	Medium-quality	70.42
Sample_T6B_k141_72159	16258	22	7	0	Medium-quality	70.41
Sample_C6C_k141_145636	81728	110	8	11	Medium-quality	70.39
Sample_C12B_k141_164197	23990	34	9	1	Medium-quality	70.33
Sample_T14B_k141_179823	32379	40	1	5	Medium-quality	70.25
Sample_C6B_k141_47274	56069	59	4	27	Medium-quality	70.23
Sample_T2B_k141_145221_fragment_1	39606	53	8	2	Medium-quality	70.23
Sample_T6C_k141_149574	10271	17	0	0	Medium-quality	70.18
Sample_C8B_k141_200111_fragment_1	32099	47	31	0	Medium-quality	70.17
Sample_C5B_k141_58709	58861	59	1	24	Medium-quality	70.09
Sample_T8A_k141_238841	59766	49	7	7	Medium-quality	70.06
Sample_T7C_k141_81877	41969	47	7	1	Medium-quality	70
Sample_T11A_k141_20225_fragment_1	35547	44	8	0	Medium-quality	69.98
Sample_C7A_k141_137528_fragment_1	32544	40	21	0	Medium-quality	69.97
Sample_T5A_k141_32917_fragment_2	20181	26	10	0	Medium-quality	69.96

Sample_T7A_k141_24808	52810	70	13	3	Medium-quality	69.96
Sample_T16B_k141_41122	30178	40	2	0	Medium-quality	69.94
Sample_T5C_k141_56394	36479	50	16	2	Medium-quality	69.92
Sample_C13A_k141_112738_fragment_1	33837	44	8	0	Medium-quality	69.91
Sample_T7A_k141_133633	24791	26	19	0	Medium-quality	69.91
Sample_T12A_k141_45471	10815	14	0	0	Medium-quality	69.85
Sample_T4C_k141_59763_fragment_1	58734	65	17	12	Medium-quality	69.82
Sample_T13C_k141_94783	81437	100	3	9	Medium-quality	69.79
Sample_C10A_k141_44268	40712	45	11	4	Medium-quality	69.78
Sample_T2B_k141_79480	46321	87	23	1	Medium-quality	69.78
Sample_T8B_k141_6701	27895	40	13	0	Medium-quality	69.77
Sample_C8B_k141_49247	71447	85	2	12	Medium-quality	69.74
Sample_T11C_k141_154022	80894	69	2	7	Medium-quality	69.74
Sample_T13C_k141_116468	57482	77	14	2	Medium-quality	69.72
Sample_C9A_k141_112208_fragment_2	56682	57	25	11	Medium-quality	69.7
Sample_C12C_k141_38518	30869	43	19	3	Medium-quality	69.69
Sample_T5B_k141_6263_fragment_1	59137	62	14	6	Medium-quality	69.59
Sample_T4A_k141_65300	128227	121	1	33	Medium-quality	69.55
Sample_C4C_k141_161569	129949	107	2	70	Medium-quality	69.53
Sample_T17B_k141_12158	109736	116	1	46	Medium-quality	69.52
Sample_T10A_k141_88923	44158	62	17	2	Medium-quality	69.47
Sample_C9C_k141_18685	49707	39	0	16	Medium-quality	69.46
Sample_T6A_k141_282109_fragment_1	104112	125	17	4	Medium-quality	69.45
Sample_C2C_k141_178646	70038	65	2	37	Medium-quality	69.43
Sample_T12A_k141_36040	35271	47	26	6	Medium-quality	69.34
Sample_T4B_k141_312806	29122	25	0	17	Medium-quality	69.29
Sample_C10C_k141_200306	43209	62	1	4	Medium-quality	69.26
Sample_C10A_k141_312842	41545	45	8	2	Medium-quality	69.18
Sample_T16C_k141_2871	19841	15	0	8	Medium-quality	69.16
Sample_T2C_k141_198556_fragment_1	31973	53	21	1	Medium-quality	69.16
Sample_C11B_k141_31792_fragment_1	22357	31	24	0	Medium-quality	69.08
Sample_T5B_k141_45131	31068	44	14	2	Medium-quality	69.07
Sample_C2C_k141_178430_fragment_1	23679	37	12	1	Medium-quality	68.98
Sample_T18A_k141_123447	95695	97	1	37	Medium-quality	68.94
Sample_T12B_k141_97391_fragment_1	50837	73	15	5	Medium-quality	68.9
Sample_C4C_k141_201635	29028	51	17	0	Medium-quality	68.83
Sample_C12C_k141_206829	15650	19	8	0	Medium-quality	68.8
Sample_C8B_k141_181930	35375	47	6	3	Medium-quality	68.75
Sample_T7A_k141_208194_fragment_1	34367	37	4	3	Medium-quality	68.74

Sample_C4B_k141_70909	33496	34	1	20	Medium-quality	68.67
Sample_C11A_k141_67576	57515	81	10	2	Medium-quality	68.66
Sample_C2C_k141_221798	128224	114	1	66	Medium-quality	68.64
Sample_T11B_k141_83310	31912	26	0	8	Medium-quality	68.64
Sample_T10C_k141_39212	41479	62	17	0	Medium-quality	68.6
Sample_T3B_k141_56224	28252	32	22	2	Medium-quality	68.59
Sample_C8B_k141_191456	25189	33	14	1	Medium-quality	68.58
Sample_T3C_k141_131781_fragment_1	37802	43	11	0	Medium-quality	68.58
Sample_C9A_k141_126286_fragment_1	26398	44	6	3	Medium-quality	68.52
Sample_C3A_k141_85758	27638	48	19	0	Medium-quality	68.51
Sample_T18B_k141_17597	65720	53	3	18	Medium-quality	68.46
Sample_T17A_k141_34714	191624	247	1	14	Medium-quality	68.46
Sample_C11B_k141_113682_fragment_1	41740	84	10	1	Medium-quality	68.45
Sample_C14A_k141_98805_fragment_1	30628	39	4	1	Medium-quality	68.41
Sample_C10C_k141_49307	236439	222	16	129	Medium-quality	68.36
Sample_T2C_k141_33307_fragment_2	31795	36	15	0	Medium-quality	68.36
Sample_C4B_k141_6688	130480	135	1	72	Medium-quality	68.33
Sample_T15A_k141_111022_fragment_1	26478	43	15	1	Medium-quality	68.3
Sample_T17C_k141_14754_fragment_1	46285	64	11	4	Medium-quality	68.28
Sample_C2C_k141_69545	25063	25	7	0	Medium-quality	68.22
Sample_C13A_k141_92645	15596	20	10	0	Medium-quality	68.17
Sample_C4A_k141_101462_fragment_1	25470	45	10	0	Medium-quality	68.16
Sample_T2C_k141_276846	15697	20	10	0	Medium-quality	68.12
Sample_C6A_k141_112330	15133	19	6	0	Medium-quality	68.11
Sample_C11A_k141_186272	37965	34	10	0	Medium-quality	68.08
Sample_C2A_k141_147698	8092	11	0	5	Medium-quality	68.07
Sample_T16A_k141_118145	97026	81	0	74	Medium-quality	68.07
Sample_C9A_k141_99058	25132	44	17	1	Medium-quality	68.07
Sample_T14A_k141_85439_fragment_1	32183	36	12	0	Medium-quality	68.07
Sample_T8C_k141_10341	35617	46	16	6	Medium-quality	68.05
Sample_C4B_k141_130849	85514	113	24	3	Medium-quality	68.04
Sample_C8C_k141_119772_fragment_1	12967	18	3	0	Medium-quality	68.04
Sample_T2A_k141_219421_fragment_2	40990	51	14	6	Medium-quality	68.02
Sample_T2A_k141_253968	138455	121	1	65	Medium-quality	67.91
Sample_T5B_k141_35277	31639	36	15	0	Medium-quality	67.87
Sample_T15C_k141_53883	27922	40	12	1	Medium-quality	67.8
Sample_T15A_k141_117618	28455	43	13	0	Medium-quality	67.79
Sample_T2C_k141_45468	111629	149	23	4	Medium-quality	67.77
Sample_T6C_k141_32217	25951	37	20	0	Medium-quality	67.77

Sample_T15A_k141_149040	80402	94	3	3	Medium-quality	67.75
Sample_T6B_k141_145255	27607	44	17	0	Medium-quality	67.73
Sample_T8B_k141_184526	15831	21	7	0	Medium-quality	67.72
Sample_T13A_k141_36206	28284	28	5	0	Medium-quality	67.71
Sample_T5C_k141_171024	15568	22	7	0	Medium-quality	67.71
Sample_T4B_k141_261231	28453	31	0	21	Medium-quality	67.7
Sample_T16C_k141_68905	32103	42	21	0	Medium-quality	67.64
Sample_C14B_k141_9282	28883	45	17	0	Medium-quality	67.63
Sample_C7A_k141_79811	49969	57	8	4	Medium-quality	67.62
Sample_T12C_k141_45806	15567	21	9	0	Medium-quality	67.62
Sample_C10C_k141_105925	22243	33	13	1	Medium-quality	67.58
Sample_C9B_k141_11312	32678	31	7	2	Medium-quality	67.51
Sample_T4A_k141_19376	81858	81	2	53	Medium-quality	67.43
Sample_T2B_k141_148595_fragment_1	46519	64	22	5	Medium-quality	67.39
Sample_C3A_k141_379	39784	41	1	9	Medium-quality	67.35
Sample_T10C_k141_5323	27198	23	6	0	Medium-quality	67.35
Sample_C2C_k141_281474	9214	12	4	0	Medium-quality	67.34
Sample_T2C_k141_238894	9382	16	5	1	Medium-quality	67.29
Sample_C1A_k141_28229_fragment_1	27267	38	17	0	Medium-quality	67.23
Sample_T10A_k141_181671_fragment_1	45812	54	10	7	Medium-quality	67.15
Sample_C3C_k141_109001	27888	36	2	1	Medium-quality	67.13
Sample_C2A_k141_144100	69002	89	2	22	Medium-quality	67.12
Sample_T3A_k141_7551	31777	42	19	0	Medium-quality	67.12
Sample_T2B_k141_258884_fragment_1	29599	45	21	0	Medium-quality	67.11
Sample_T11C_k141_35983_fragment_1	30639	50	12	0	Medium-quality	67.06
Sample_C1A_k141_237796	159336	156	1	105	Medium-quality	66.99
Sample_T2B_k141_263738	99903	80	1	61	Medium-quality	66.94
Sample_C7A_k141_88379	34033	51	18	0	Medium-quality	66.92
Sample_T15A_k141_14802	26116	21	0	10	Medium-quality	66.91
Sample_T10C_k141_48321_fragment_1	22675	35	12	0	Medium-quality	66.91
Sample_C4C_k141_212533_fragment_1	25237	39	13	0	Medium-quality	66.88
Sample_C10A_k141_66888	99945	85	2	47	Medium-quality	66.82
Sample_C8B_k141_70923	24670	27	7	1	Medium-quality	66.82
Sample_T14B_k141_215017	57794	44	1	28	Medium-quality	66.79
Sample_T13B_k141_120787_fragment_1	43236	55	11	3	Medium-quality	66.79
Sample_T3C_k141_121174	8718	13	3	0	Medium-quality	66.7
Sample_T6B_k141_129970	32955	44	2	0	Medium-quality	66.69
Sample_C7B_k141_44995	36101	50	5	1	Medium-quality	66.68
Sample_T8C_k141_7779_fragment_2	31925	46	27	2	Medium-quality	66.59

Sample_C14B_k141_56736	23406	29	16	0	Medium-quality	66.57
Sample_C6C_k141_146986	29326	39	2	3	Medium-quality	66.57
Sample_C9A_k141_5824_fragment_1	24156	31	15	0	Medium-quality	66.55
Sample_C10A_k141_208618_fragment_1	30673	41	25	0	Medium-quality	66.52
Sample_T12C_k141_192242	29836	43	12	0	Medium-quality	66.37
Sample_C3B_k141_121193	31054	36	1	7	Medium-quality	66.33
Sample_T11C_k141_122742_fragment_1	39109	49	19	4	Medium-quality	66.33
Sample_C10A_k141_286043	22901	34	14	1	Medium-quality	66.32
Sample_C11B_k141_25089_fragment_1	45294	54	27	6	Medium-quality	66.32
Sample_C10A_k141_190834_fragment_1	52544	78	26	10	Medium-quality	66.29
Sample_C8C_k141_83428	49510	35	0	17	Medium-quality	66.22
Sample_C2A_k141_228560	25904	42	5	0	Medium-quality	66.16
Sample_C9C_k141_49901_fragment_2	73280	74	11	2	Medium-quality	66.14
Sample_C2C_k141_211777	83506	87	2	25	Medium-quality	66.11
Sample_T12A_k141_69488_fragment_2	30598	40	7	2	Medium-quality	66.09
Sample_T7A_k141_66312	11809	20	3	0	Medium-quality	66.03
Sample_C9A_k141_121675	37939	53	19	3	Medium-quality	66.02
Sample_C8A_k141_100624_fragment_1	41478	48	24	0	Medium-quality	65.96
Sample_T15C_k141_66608	36085	56	25	2	Medium-quality	65.94
Sample_T1B_k141_27422	25540	34	12	2	Medium-quality	65.89
Sample_T7A_k141_101544	26899	33	20	0	Medium-quality	65.89
Sample_T10B_k141_100071_fragment_1	144400	130	4	15	Medium-quality	65.83
Sample_T8C_k141_212113	23407	34	18	0	Medium-quality	65.79
Sample_C5B_k141_29296_fragment_1	21337	30	23	0	Medium-quality	65.78
Sample_T16B_k141_54099	106031	112	1	41	Medium-quality	65.74
Sample_C12A_k141_66845	30551	36	0	5	Medium-quality	65.72
Sample_T7B_k141_110896_fragment_3	28281	42	8	1	Medium-quality	65.71
Sample_T11C_k141_154884_fragment_1	29850	41	15	3	Medium-quality	65.62
Sample_C9A_k141_60789_fragment_1	27606	41	14	0	Medium-quality	65.57
Sample_C2B_k141_226693	56735	54	1	41	Medium-quality	65.56
Sample_T5C_k141_4922	72573	89	1	40	Medium-quality	65.53
Sample_T3C_k141_51664	39858	60	25	0	Medium-quality	65.46
Sample_T4C_k141_17071_fragment_1	29782	46	13	0	Medium-quality	65.46
Sample_C8B_k141_189510	26282	39	15	2	Medium-quality	65.45
Sample_C12C_k141_169010	54055	67	25	9	Medium-quality	65.41
Sample_T3A_k141_1195	30240	38	1	8	Medium-quality	65.4
Sample_T1B_k141_6393	39618	74	18	2	Medium-quality	65.37
Sample_C11C_k141_77552_fragment_1	35492	50	16	0	Medium-quality	65.35
Sample_C10B_k141_34029	35359	59	15	0	Medium-quality	65.26

Sample_C14C_k141_155034	22099	32	8	0	Medium-quality	65.21
Sample_C1A_k141_257562	28348	37	3	3	Medium-quality	65.17
Sample_T15B_k141_140832	32763	47	18	1	Medium-quality	65.17
Sample_C10A_k141_261508	22486	32	12	0	Medium-quality	65.16
Sample_C1C_k141_158474_fragment_2	40424	53	21	1	Medium-quality	65.12
Sample_C12B_k141_118772	51163	47	0	22	Medium-quality	65.1
Sample_T4B_k141_149752	82863	90	1	31	Medium-quality	65.09
Sample_C13C_k141_79647_fragment_1	32454	36	16	0	Medium-quality	65.07
Sample_T11B_k141_154560	41881	52	7	2	Medium-quality	65.05
Sample_C12C_k141_21538	8245	14	2	0	Medium-quality	64.98
Sample_C5A_k141_120591_fragment_1	71497	96	5	8	Medium-quality	64.98
Sample_T3A_k141_27585_fragment_1	26340	44	8	1	Medium-quality	64.97
Sample_T17A_k141_30864	38893	41	0	12	Medium-quality	64.96
Sample_C8A_k141_113838	15059	19	7	0	Medium-quality	64.94
Sample_C4C_k141_242757	35389	57	8	1	Medium-quality	64.92
Sample_C14A_k141_190663	25384	39	11	1	Medium-quality	64.91
Sample_T3B_k141_52419	40435	59	14	0	Medium-quality	64.9
Sample_T2C_k141_108733_fragment_1	39315	55	9	2	Medium-quality	64.83
Sample_C4B_k141_87779	29239	29	1	15	Medium-quality	64.8
Sample_C12B_k141_171581_fragment_1	32934	48	14	3	Medium-quality	64.79
Sample_T3C_k141_23780_fragment_1	25574	34	9	0	Medium-quality	64.78
Sample_T17C_k141_5723	11880	19	1	0	Medium-quality	64.7
Sample_C13A_k141_48846	38222	47	17	1	Medium-quality	64.66
Sample_C13C_k141_72236	37813	54	19	1	Medium-quality	64.64
Sample_T4B_k141_312409	32680	37	1	16	Medium-quality	64.6
Sample_C10A_k141_335615	24843	28	6	1	Medium-quality	64.58
Sample_T18B_k141_94457	31049	48	17	0	Medium-quality	64.58
Sample_C3C_k141_245408_fragment_2	43402	56	13	9	Medium-quality	64.5
Sample_T13C_k141_45867	111293	115	3	13	Medium-quality	64.44
Sample_C11C_k141_168300	87600	109	25	1	Medium-quality	64.41
Sample_C8A_k141_96157	28026	39	1	1	Medium-quality	64.4
Sample_C14A_k141_51343	160274	149	1	84	Medium-quality	64.37
Sample_C3C_k141_110465_fragment_1	36300	44	8	5	Medium-quality	64.34
Sample_C1A_k141_213369	122533	109	3	71	Medium-quality	64.32
Sample_C5B_k141_22462_fragment_1	32357	42	28	2	Medium-quality	64.31
Sample_C4B_k141_227392	55637	53	1	11	Medium-quality	64.29
Sample_C3C_k141_81281_fragment_3	24900	31	17	1	Medium-quality	64.28
Sample_T2B_k141_256156	8796	11	3	0	Medium-quality	64.26
Sample_T4B_k141_163379	71693	67	1	45	Medium-quality	64.16

Sample_C5A_k141_31733_fragment_1	24235	37	19	0	Medium-quality	64.15
Sample_T8B_k141_163875	28236	43	23	1	Medium-quality	64.11
Sample_C1C_k141_2362	144787	177	10	29	Medium-quality	64.09
Sample_C2C_k141_41267	118141	84	1	59	Medium-quality	64.08
Sample_C3A_k141_165857	119543	117	1	67	Medium-quality	63.99
Sample_T5C_k141_180566_fragment_1	22717	27	2	0	Medium-quality	63.96
Sample_T11C_k141_72411	41479	50	10	10	Medium-quality	63.93
Sample_C4B_k141_158862_fragment_1	34415	44	19	0	Medium-quality	63.92
Sample_C5A_k141_100811	32693	44	8	2	Medium-quality	63.88
Sample_C10A_k141_8109	24604	33	14	0	Medium-quality	63.88
Sample_C2C_k141_208839	55240	43	1	27	Medium-quality	63.84
Sample_T5C_k141_98847	103284	103	1	51	Medium-quality	63.82
Sample_T8C_k141_22422	29850	35	8	2	Medium-quality	63.81
Sample_T17C_k141_22156	162262	127	2	84	Medium-quality	63.77
Sample_T6B_k141_104178	64674	103	28	5	Medium-quality	63.76
Sample_C10A_k141_201062	27092	17	0	2	Medium-quality	63.75
Sample_C5A_k141_191831	81156	67	1	49	Medium-quality	63.75
Sample_T10C_k141_83130	56181	65	8	3	Medium-quality	63.74
Sample_C5A_k141_182490	34124	38	16	6	Medium-quality	63.73
Sample_T4C_k141_17037	30396	47	22	3	Medium-quality	63.73
Sample_T13B_k141_131786_fragment_1	29972	39	23	0	Medium-quality	63.72
Sample_T2B_k141_65122	42084	39	1	11	Medium-quality	63.68
Sample_C5A_k141_57669	94688	67	1	30	Medium-quality	63.65
Sample_C13A_k141_69240	55027	56	1	47	Medium-quality	63.59
Sample_T12C_k141_108633	8683	14	2	0	Medium-quality	63.58
Sample_C12A_k141_64493	57400	46	1	12	Medium-quality	63.57
Sample_T6C_k141_7875	51443	74	35	8	Medium-quality	63.52
Sample_C4A_k141_157033_fragment_1	33939	32	14	2	Medium-quality	63.5
Sample_C4C_k141_33821	25016	37	17	0	Medium-quality	63.46
Sample_C5A_k141_174626	41458	55	10	4	Medium-quality	63.44
Sample_C4B_k141_159698	102640	114	1	50	Medium-quality	63.43
Sample_T15A_k141_127655	60801	58	1	26	Medium-quality	63.43
Sample_C5A_k141_27971	22489	28	13	0	Medium-quality	63.42
Sample_T11C_k141_153295	68986	81	17	1	Medium-quality	63.41
Sample_T4A_k141_21061	33532	34	10	3	Medium-quality	63.4
Sample_T8C_k141_73427_fragment_1	68488	63	9	4	Medium-quality	63.4
Sample_C12C_k141_220605_fragment_1	23859	36	3	1	Medium-quality	63.35
Sample_C10A_k141_172610	24646	42	10	0	Medium-quality	63.31
Sample_T11C_k141_156497	25309	36	19	0	Medium-quality	63.3

Sample_T4B_k141_132775	25931	35	11	1	Medium-quality	63.28
Sample_C10C_k141_65428	29217	38	21	0	Medium-quality	63.26
Sample_C14A_k141_109520	8053	10	1	0	Medium-quality	63.26
Sample_T12C_k141_19949	44210	33	2	7	Medium-quality	63.2
Sample_T11B_k141_19142	22036	30	15	1	Medium-quality	63.17
Sample_C6B_k141_44192	33003	51	14	3	Medium-quality	63.16
Sample_T12C_k141_73657	49035	37	1	7	Medium-quality	63.08
Sample_T15A_k141_24109	43069	31	1	8	Medium-quality	63.02
Sample_T14B_k141_65973	17620	24	2	1	Medium-quality	63.01
Sample_T13A_k141_14829	24093	27	12	0	Medium-quality	63
Sample_C12B_k141_147771	26864	50	13	0	Medium-quality	62.93
Sample_T4B_k141_63033_fragment_1	35570	49	19	4	Medium-quality	62.91
Sample_T12C_k141_14724	97405	176	23	1	Medium-quality	62.89
Sample_T6C_k141_225382	25082	29	20	0	Medium-quality	62.86
Sample_C3A_k141_145173	24327	34	10	1	Medium-quality	62.84
Sample_T10A_k141_8502_fragment_1	37752	42	9	0	Medium-quality	62.84
Sample_C6B_k141_218086	102891	101	4	12	Medium-quality	62.83
Sample_C2A_k141_98256	111152	111	1	66	Medium-quality	62.8
Sample_T2A_k141_128830_fragment_1	64458	64	24	7	Medium-quality	62.69
Sample_C7A_k141_94781	127800	128	1	51	Medium-quality	62.68
Sample_T14C_k141_83992_fragment_4	34657	45	16	3	Medium-quality	62.68
Sample_C5A_k141_226701	27612	34	1	1	Medium-quality	62.66
Sample_T4B_k141_299985	30378	48	2	2	Medium-quality	62.66
Sample_T14A_k141_132046	49158	71	13	4	Medium-quality	62.64
Sample_C5A_k141_112900	31520	37	2	4	Medium-quality	62.56
Sample_C2C_k141_79401	44624	55	1	24	Medium-quality	62.55
Sample_C6B_k141_165965	82754	89	1	65	Medium-quality	62.55
Sample_T16A_k141_29017_fragment_1	23468	23	14	1	Medium-quality	62.53
Sample_T2A_k141_98930_fragment_1	79950	111	14	13	Medium-quality	62.53
Sample_T17B_k141_31867	25129	27	9	0	Medium-quality	62.42
Sample_T14A_k141_214427	94372	131	18	2	Medium-quality	62.41
Sample_T13C_k141_21039_fragment_1	60133	72	14	10	Medium-quality	62.4
Sample_C3C_k141_33671_fragment_2	38922	58	20	0	Medium-quality	62.36
Sample_C2A_k141_272691	23391	36	11	2	Medium-quality	62.23
Sample_C6B_k141_13063	116046	146	20	5	Medium-quality	62.23
Sample_T12B_k141_133128	33858	47	14	0	Medium-quality	62.22
Sample_T16B_k141_150234	24826	28	9	1	Medium-quality	62.21
Sample_T10A_k141_157265	26159	39	14	0	Medium-quality	62.21
Sample_T2C_k141_120802	106474	93	2	51	Medium-quality	62.2

Sample_C12C_k141_150409	23397	33	16	0	Medium-quality	62.19
Sample_T7A_k141_35174	28908	25	0	6	Medium-quality	62.18
Sample_T17A_k141_19155	28452	30	16	2	Medium-quality	62.15
Sample_C8C_k141_53832	160849	150	2	22	Medium-quality	62.13
Sample_C6B_k141_133785	49987	58	4	21	Medium-quality	62.11
Sample_C13B_k141_58618	47213	50	2	14	Medium-quality	62.1
Sample_T13C_k141_110355	30527	45	15	2	Medium-quality	62.04
Sample_T5C_k141_110669	31224	38	1	23	Medium-quality	62.03
Sample_C12A_k141_2995_fragment_1	20169	34	11	1	Medium-quality	62.01
Sample_T5A_k141_54332	56852	60	2	11	Medium-quality	61.88
Sample_C12C_k141_121202	35882	50	7	2	Medium-quality	61.86
Sample_C2A_k141_74650	46055	40	2	23	Medium-quality	61.85
Sample_C12B_k141_58369	14223	24	1	1	Medium-quality	61.76
Sample_T2C_k141_142981	53426	41	1	22	Medium-quality	61.74
Sample_C8C_k141_20925	222326	196	12	58	Medium-quality	61.73
Sample_T7A_k141_658	52847	54	2	33	Medium-quality	61.71
Sample_T18B_k141_4006_fragment_2	23315	37	5	0	Medium-quality	61.71
Sample_C2C_k141_201825_fragment_2	28162	40	21	1	Medium-quality	61.68
Sample_T14A_k141_197014_fragment_1	23719	31	11	0	Medium-quality	61.65
Sample_C13B_k141_64840	113600	107	1	28	Medium-quality	61.61
Sample_T7B_k141_69439	28003	40	12	0	Medium-quality	61.53
Sample_C4A_k141_31203	53889	62	20	6	Medium-quality	61.51
Sample_C6B_k141_197660	29280	40	16	0	Medium-quality	61.5
Sample_C10B_k141_42177	113358	89	1	40	Medium-quality	61.47
Sample_T11A_k141_44439	39331	39	1	24	Medium-quality	61.42
Sample_C8C_k141_145683_fragment_1	37199	54	13	8	Medium-quality	61.37
Sample_T10A_k141_140208_fragment_1	43435	57	7	5	Medium-quality	61.37
Sample_C11B_k141_165848	7221	14	5	0	Medium-quality	61.35
Sample_C2C_k141_110192_fragment_1	113852	183	22	5	Medium-quality	61.31
Sample_C2C_k141_181360	114483	104	1	60	Medium-quality	61.29
Sample_C3B_k141_116525	53644	69	7	3	Medium-quality	61.28
Sample_C13A_k141_109419	68311	96	15	1	Medium-quality	61.26
Sample_C13B_k141_15207	58011	88	12	0	Medium-quality	61.26
Sample_C4A_k141_190056_fragment_1	20597	31	4	2	Medium-quality	61.25
Sample_T16C_k141_25843	30459	42	18	4	Medium-quality	61.25
Sample_C4B_k141_18805	24457	41	11	1	Medium-quality	61.22
Sample_T13B_k141_137304_fragment_1	30692	31	11	3	Medium-quality	61.21
Sample_C8C_k141_132732	37181	43	23	1	Medium-quality	61.19
Sample_C14B_k141_104700	23718	30	15	0	Medium-quality	61.17

Sample_C11A_k141_68298_fragment_1	37659	54	16	2	Medium-quality	61.15
Sample_T16B_k141_96942_fragment_1	98415	147	25	2	Medium-quality	61.12
Sample_T1C_k141_18679	12878	13	0	8	Medium-quality	61.09
Sample_T12A_k141_59716	7837	12	2	0	Medium-quality	61.08
Sample_C11C_k141_97978_fragment_1	12732	14	1	1	Medium-quality	61.03
Sample_C2B_k141_284463	33775	42	16	2	Medium-quality	61.03
Sample_C11B_k141_9955_fragment_1	38363	50	5	5	Medium-quality	61.01
Sample_C13C_k141_35471	106301	111	24	2	Medium-quality	61.01
Sample_T5B_k141_5135	40334	39	0	16	Medium-quality	60.99
Sample_T11A_k141_64652	27182	44	19	0	Medium-quality	60.97
Sample_T13A_k141_97947	46505	44	2	24	Medium-quality	60.95
Sample_C4A_k141_191465	349120	348	23	224	Medium-quality	60.94
Sample_C2A_k141_73277	11619	13	6	0	Medium-quality	60.94
Sample_T6A_k141_172440	8417	9	5	0	Medium-quality	60.87
Sample_T8B_k141_117236	35206	43	3	2	Medium-quality	60.86
Sample_T1A_k141_42366	43415	28	0	11	Medium-quality	60.83
Sample_C12C_k141_240827	21497	28	10	0	Medium-quality	60.74
Sample_C6A_k141_201742	151810	164	2	69	Medium-quality	60.67
Sample_T12C_k141_14381	46407	73	8	2	Medium-quality	60.66
Sample_C11C_k141_178920_fragment_3	21344	24	6	4	Medium-quality	60.63
Sample_T12A_k141_100710	35722	42	25	1	Medium-quality	60.62
Sample_T17A_k141_38116	29242	51	12	2	Medium-quality	60.62
Sample_C10A_k141_216084	67064	80	1	45	Medium-quality	60.56
Sample_T3B_k141_83235_fragment_1	44556	71	28	0	Medium-quality	60.54
Sample_C6C_k141_205312	93259	83	1	68	Medium-quality	60.35
Sample_T4B_k141_231502_fragment_1	16964	20	13	1	Medium-quality	60.28
Sample_T17B_k141_26391	49257	46	0	31	Medium-quality	60.27
Sample_C2B_k141_103692	26797	26	0	18	Medium-quality	60.23
Sample_C8C_k141_83692	38563	41	1	34	Medium-quality	60.22
Sample_C13C_k141_13419_fragment_1	27857	35	21	2	Medium-quality	60.22
Sample_T5C_k141_165743	20956	28	9	1	Medium-quality	60.15
Sample_C2A_k141_4958	94919	108	1	20	Medium-quality	60.13
Sample_T8A_k141_69628_fragment_1	42177	54	18	3	Medium-quality	60.13
Sample_T17A_k141_40895	21097	36	14	0	Medium-quality	60.12
Sample_T15A_k141_73687	41783	38	1	11	Medium-quality	60
Sample_T6C_k141_197361	30142	23	1	8	Medium-quality	59.96
Sample_C4B_k141_92779_fragment_1	29324	43	24	4	Medium-quality	59.96
Sample_T5C_k141_91016	41136	37	1	12	Medium-quality	59.94
Sample_C14A_k141_172954	21504	25	14	0	Medium-quality	59.87

Sample_T12A_k141_327611	75078	154	13	3	Medium-quality	59.87
Sample_C10A_k141_208590_fragment_1	25068	32	6	0	Medium-quality	59.84
Sample_C9A_k141_15473_fragment_1	37605	56	14	5	Medium-quality	59.83
Sample_T11A_k141_4241_fragment_1	26094	37	7	1	Medium-quality	59.83
Sample_T13B_k141_175420	105335	90	1	62	Medium-quality	59.8
Sample_T5A_k141_36234	36251	50	12	5	Medium-quality	59.79
Sample_T17C_k141_9107	87217	74	1	63	Medium-quality	59.75
Sample_C8B_k141_202900_fragment_1	30813	40	8	3	Medium-quality	59.74
Sample_T6C_k141_121960	22892	32	4	1	Medium-quality	59.72
Sample_T18C_k141_93980_fragment_1	25167	33	18	2	Medium-quality	59.67
Sample_C5C_k141_95381	19435	29	22	2	Medium-quality	59.62
Sample_C5A_k141_279842	22530	28	12	0	Medium-quality	59.61
Sample_T4B_k141_239997_fragment_2	21751	24	10	5	Medium-quality	59.6
Sample_T3A_k141_66246	37986	46	10	9	Medium-quality	59.56
Sample_T12A_k141_85626	47673	73	7	1	Medium-quality	59.48
Sample_C4A_k141_30381	57470	48	2	14	Medium-quality	59.47
Sample_T15B_k141_83010	19212	29	9	0	Medium-quality	59.45
Sample_C9A_k141_81979	58048	39	0	17	Medium-quality	59.44
Sample_C9B_k141_98451	195914	175	2	118	Medium-quality	59.42
Sample_C4C_k141_160581_fragment_1	33328	46	20	0	Medium-quality	59.42
Sample_C4B_k141_205339_fragment_1	22216	33	16	2	Medium-quality	59.41
Sample_C7B_k141_180709	22865	31	17	0	Medium-quality	59.35
Sample_C13C_k141_83957_fragment_1	35008	56	10	1	Medium-quality	59.32
Sample_T2B_k141_31100	35548	48	11	0	Medium-quality	59.32
Sample_C3C_k141_206706	35919	63	10	0	Medium-quality	59.29
Sample_T11B_k141_118919	30829	39	14	0	Medium-quality	59.27
Sample_T17A_k141_45206	47898	55	7	5	Medium-quality	59.27
Sample_C12C_k141_14905	38300	40	2	9	Medium-quality	59.23
Sample_T2B_k141_292348	29567	29	1	10	Medium-quality	59.23
Sample_C12A_k141_137967	44342	40	1	11	Medium-quality	59.21
Sample_T11C_k141_135670	46161	55	8	6	Medium-quality	59.21
Sample_C2B_k141_111577_fragment_1	23839	28	7	0	Medium-quality	59.15
Sample_T16C_k141_135127	39115	41	0	14	Medium-quality	59.14
Sample_T12A_k141_117026	24472	38	18	0	Medium-quality	59.13
Sample_T7C_k141_79973	32712	63	9	4	Medium-quality	59.13
Sample_C10A_k141_42513	49136	74	9	2	Medium-quality	59.12
Sample_T4B_k141_174331	34070	61	7	2	Medium-quality	59.09
Sample_C13C_k141_65976	23651	34	26	0	Medium-quality	59.04
Sample_C10A_k141_106802	32524	49	7	4	Medium-quality	59

Sample_C11A_k141_173511_fragment_1	26837	42	24	0	Medium-quality	58.98
Sample_T3B_k141_13597	14929	28	11	0	Medium-quality	58.97
Sample_T17B_k141_27582	25376	34	14	0	Medium-quality	58.96
Sample_C11B_k141_36519	44231	52	23	5	Medium-quality	58.93
Sample_T8A_k141_176147	31977	46	15	0	Medium-quality	58.91
Sample_C5A_k141_130003	20415	14	0	4	Medium-quality	58.87
Sample_T14C_k141_149756	20312	37	9	1	Medium-quality	58.84
Sample_T4B_k141_173027_fragment_1	25355	38	6	0	Medium-quality	58.82
Sample_T11C_k141_108371	47821	62	10	13	Medium-quality	58.81
Sample_C2B_k141_113246_fragment_2	39892	57	24	0	Medium-quality	58.8
Sample_C6A_k141_197687_fragment_2	40504	53	9	8	Medium-quality	58.74
Sample_C8B_k141_164665	34902	60	13	0	Medium-quality	58.7
Sample_T12C_k141_148758	47259	62	15	0	Medium-quality	58.69
Sample_T8A_k141_200664	22363	34	3	0	Medium-quality	58.62
Sample_C6A_k141_234415	6662	8	0	1	Medium-quality	58.6
Sample_C3C_k141_183679	22865	36	5	0	Medium-quality	58.57
Sample_T14B_k141_180460_fragment_1	45227	64	28	3	Medium-quality	58.57
Sample_T17B_k141_12511	32363	39	7	6	Medium-quality	58.53
Sample_C8B_k141_22819	33241	37	5	0	Medium-quality	58.49
Sample_T5B_k141_3404	37074	50	18	8	Medium-quality	58.48
Sample_C5A_k141_132241	35084	43	1	6	Medium-quality	58.46
Sample_C11B_k141_132726	94561	91	1	40	Medium-quality	58.43
Sample_T3C_k141_141865	8626	12	1	0	Medium-quality	58.43
Sample_C13A_k141_410	28795	40	8	0	Medium-quality	58.4
Sample_C11B_k141_229232	33828	40	11	2	Medium-quality	58.36
Sample_T12A_k141_328740	22202	31	13	0	Medium-quality	58.33
Sample_T12A_k141_115717	62659	58	1	28	Medium-quality	58.32
Sample_C13C_k141_46875	144486	134	1	86	Medium-quality	58.23
Sample_C5A_k141_258133_fragment_1	31589	48	15	6	Medium-quality	58.22
Sample_T8B_k141_91416	24119	46	10	1	Medium-quality	58.22
Sample_T15C_k141_141042	22421	36	12	0	Medium-quality	58.16
Sample_C11A_k141_28400	23473	34	13	0	Medium-quality	58.14
Sample_T7B_k141_137203_fragment_1	22447	29	15	0	Medium-quality	58.08
Sample_T3C_k141_140226	73066	64	3	24	Medium-quality	58.07
Sample_T8A_k141_78373	26255	30	7	3	Medium-quality	58.06
Sample_T10C_k141_50951	39385	33	6	0	Medium-quality	58.05
Sample_C8C_k141_61726	42983	58	18	6	Medium-quality	58.04
Sample_T14C_k141_29643	94305	82	3	17	Medium-quality	58.03
Sample_T14B_k141_38671_fragment_2	94836	126	20	14	Medium-quality	58.03

Sample_T14A_k141_2991	40337	46	8	5	Medium-quality	57.95
Sample_C7B_k141_126861	27431	45	9	1	Medium-quality	57.94
Sample_T12A_k141_104510_fragment_1	99466	179	21	8	Medium-quality	57.94
Sample_C8A_k141_77647	51744	52	0	26	Medium-quality	57.91
Sample_C2A_k141_33756	25668	41	9	0	Medium-quality	57.86
Sample_T12C_k141_91426	100702	110	24	1	Medium-quality	57.84
Sample_T5A_k141_52939	25843	27	6	1	Medium-quality	57.84
Sample_T6B_k141_89778	95077	123	7	9	Medium-quality	57.84
Sample_C3A_k141_240061	22503	35	7	1	Medium-quality	57.8
Sample_T7B_k141_50007_fragment_1	24469	31	16	1	Medium-quality	57.76
Sample_C6A_k141_4795	57587	54	5	12	Medium-quality	57.75
Sample_C4B_k141_214445	25998	26	4	3	Medium-quality	57.74
Sample_C13B_k141_45242	22116	28	16	0	Medium-quality	57.74
Sample_C9B_k141_41962	31827	33	5	0	Medium-quality	57.74
Sample_T3A_k141_61275	27180	38	9	0	Medium-quality	57.73
Sample_C12C_k141_105528	40956	34	1	9	Medium-quality	57.72
Sample_C8C_k141_178226	97566	82	1	14	Medium-quality	57.68
Sample_T3A_k141_51694	24407	37	18	0	Medium-quality	57.67
Sample_T5B_k141_42381	54697	69	12	1	Medium-quality	57.67
Sample_T2C_k141_89508	24360	46	9	0	Medium-quality	57.66
Sample_T4B_k141_227991	23474	34	8	0	Medium-quality	57.63
Sample_T14A_k141_79830	97428	94	1	36	Medium-quality	57.6
Sample_T7A_k141_78895	26396	37	2	2	Medium-quality	57.59
Sample_C6A_k141_13047_fragment_1	23681	41	21	0	Medium-quality	57.58
Sample_T10A_k141_63300	23597	42	13	1	Medium-quality	57.56
Sample_T10A_k141_96570_fragment_1	21656	28	18	0	Medium-quality	57.55
Sample_C4B_k141_35464	24293	31	3	0	Medium-quality	57.54
Sample_C11A_k141_65520	52630	43	1	12	Medium-quality	57.52
Sample_T4A_k141_37325	39720	35	1	18	Medium-quality	57.48
Sample_C8A_k141_174728_fragment_1	36897	39	33	0	Medium-quality	57.44
Sample_T17C_k141_24451	27753	34	12	0	Medium-quality	57.42
Sample_C1B_k141_19540	44418	46	2	8	Medium-quality	57.36
Sample_T11B_k141_28724	23108	21	6	0	Medium-quality	57.29
Sample_T8B_k141_259496	24948	36	13	1	Medium-quality	57.28
Sample_C11B_k141_52285	24171	32	12	1	Medium-quality	57.27
Sample_C2C_k141_141792	10574	16	1	2	Medium-quality	57.22
Sample_T4B_k141_372710_fragment_1	54272	79	18	13	Medium-quality	57.22
Sample_T17A_k141_11775	34231	36	0	8	Medium-quality	57.18
Sample_T2C_k141_163656	23341	42	15	0	Medium-quality	57.13

Sample_T6C_k141_110689_fragment_1	21444	33	12	1	Medium-quality	57.12
Sample_T2B_k141_78831	20099	28	13	0	Medium-quality	57.08
Sample_T10A_k141_68511	8832	9	0	1	Medium-quality	57.05
Sample_T4A_k141_37334	38579	36	11	4	Medium-quality	57
Sample_T4B_k141_120433_fragment_1	25771	33	15	2	Medium-quality	56.99
Sample_T2C_k141_213221_fragment_1	13883	17	7	2	Medium-quality	56.83
Sample_C8B_k141_34571	27497	38	8	0	Medium-quality	56.81
Sample_C12A_k141_148549	51916	75	3	9	Medium-quality	56.8
Sample_T1B_k141_19497_fragment_2	25403	38	6	3	Medium-quality	56.78
Sample_T12C_k141_9695	29065	36	0	6	Medium-quality	56.76
Sample_C6B_k141_152435	140797	124	1	87	Medium-quality	56.74
Sample_T16B_k141_164064	36166	29	0	22	Medium-quality	56.73
Sample_T13B_k141_26525	35365	33	1	3	Medium-quality	56.72
Sample_C8C_k141_91228	27443	34	8	1	Medium-quality	56.7
Sample_T14A_k141_46019	20316	22	6	0	Medium-quality	56.69
Sample_C13A_k141_68927	25672	25	4	0	Medium-quality	56.67
Sample_T13C_k141_104609	25326	43	12	0	Medium-quality	56.63
Sample_C8B_k141_105661	16323	21	0	0	Medium-quality	56.58
Sample_C3C_k141_259132_fragment_1	22654	32	19	0	Medium-quality	56.58
Sample_C4B_k141_107792	23731	21	0	16	Medium-quality	56.47
Sample_T8A_k141_133571	37306	35	1	11	Medium-quality	56.45
Sample_T12C_k141_81984	219667	183	5	102	Medium-quality	56.41
Sample_T18B_k141_3075	33753	59	27	0	Medium-quality	56.33
Sample_C10A_k141_316982_fragment_1	25967	30	17	0	Medium-quality	56.31
Sample_C2A_k141_98060	25350	40	7	0	Medium-quality	56.29
Sample_T3A_k141_117066	20961	26	11	0	Medium-quality	56.29
Sample_T6A_k141_95493	24283	25	1	2	Medium-quality	56.28
Sample_T5A_k141_12059	55865	86	10	3	Medium-quality	56.26
Sample_C1C_k141_47039	100423	86	1	39	Medium-quality	56.25
Sample_T2B_k141_26110_fragment_1	62354	87	6	8	Medium-quality	56.19
Sample_T4B_k141_9950	26597	39	8	1	Medium-quality	56.15
Sample_T8C_k141_121472_fragment_3	18518	20	0	2	Medium-quality	56.08
Sample_C7A_k141_110889	22611	37	20	0	Medium-quality	56.07
Sample_C4B_k141_225070	21166	33	4	0	Medium-quality	56.02
Sample_C3A_k141_185155	21285	31	13	1	Medium-quality	55.98
Sample_C12A_k141_145080	19133	27	5	0	Medium-quality	55.97
Sample_C9B_k141_31504	20267	29	12	0	Medium-quality	55.83
Sample_C1C_k141_91356_fragment_1	21025	28	3	0	Medium-quality	55.81
Sample_T4B_k141_278325	21330	19	3	0	Medium-quality	55.8

Sample_T15C_k141_20277	81604	62	3	10	Medium-quality	55.79
Sample_T4C_k141_113092_fragment_1	48514	63	9	9	Medium-quality	55.77
Sample_C3A_k141_145880	94559	63	1	46	Medium-quality	55.72
Sample_T5A_k141_23495	18798	26	13	0	Medium-quality	55.7
Sample_T6C_k141_254694	44749	74	19	6	Medium-quality	55.7
Sample_C8B_k141_123385	27353	43	14	0	Medium-quality	55.68
Sample_C4A_k141_182654	138108	134	1	83	Medium-quality	55.66
Sample_C2B_k141_183591	48218	58	1	25	Medium-quality	55.64
Sample_T4B_k141_90793	69989	71	1	7	Medium-quality	55.63
Sample_T10A_k141_22909	42276	38	0	11	Medium-quality	55.58
Sample_C10A_k141_135814	48157	48	1	21	Medium-quality	55.57
Sample_T2A_k141_90145_fragment_1	61084	70	18	11	Medium-quality	55.56
Sample_C13B_k141_87589	27147	32	6	0	Medium-quality	55.54
Sample_T5B_k141_64801	19260	30	14	1	Medium-quality	55.53
Sample_T5B_k141_54837	26023	39	9	0	Medium-quality	55.47
Sample_T12C_k141_111627	35494	34	1	22	Medium-quality	55.43
Sample_C2B_k141_216720	22614	48	4	0	Medium-quality	55.43
Sample_T7A_k141_127299	19754	23	12	0	Medium-quality	55.43
Sample_T7A_k141_183019	75623	82	2	16	Medium-quality	55.42
Sample_T13C_k141_25399	19300	34	7	0	Medium-quality	55.41
Sample_C5B_k141_32555	16458	21	6	0	Medium-quality	55.39
Sample_C9A_k141_5227	24279	31	20	0	Medium-quality	55.39
Sample_C2C_k141_240215	35466	38	1	17	Medium-quality	55.38
Sample_T11B_k141_58168	23565	15	0	4	Medium-quality	55.38
Sample_T2C_k141_10602	32552	43	8	1	Medium-quality	55.38
Sample_T10A_k141_59716	22548	33	14	0	Medium-quality	55.33
Sample_C11B_k141_19129	21212	28	14	0	Medium-quality	55.32
Sample_C13B_k141_76267	23811	34	10	0	Medium-quality	55.3
Sample_T4A_k141_52411_fragment_2	22207	23	16	0	Medium-quality	55.26
Sample_C10A_k141_217231	66564	90	2	24	Medium-quality	55.25
Sample_C14A_k141_117099	20282	31	10	0	Medium-quality	55.19
Sample_T8C_k141_76980	8534	12	0	1	Medium-quality	55.16
Sample_C3B_k141_258194	128149	116	1	90	Medium-quality	55.1
Sample_T15C_k141_82646	47731	41	1	26	Medium-quality	55.07
Sample_T11A_k141_48637	36821	52	18	8	Medium-quality	55.07
Sample_T11A_k141_29962_fragment_1	39150	39	9	0	Medium-quality	54.98
Sample_T13B_k141_129764_fragment_1	35119	49	36	0	Medium-quality	54.89
Sample_C10A_k141_19668	142053	144	2	79	Medium-quality	54.82
Sample_T7B_k141_48742	84664	74	1	30	Medium-quality	54.79

Sample_T13B_k141_73333	19378	33	7	0	Medium-quality	54.79
Sample_C3A_k141_147232_fragment_1	36187	33	1	5	Medium-quality	54.76
Sample_C2C_k141_132450_fragment_2	89969	155	23	6	Medium-quality	54.68
Sample_C3A_k141_49112	61034	70	2	20	Medium-quality	54.65
Sample_C2C_k141_86576_fragment_4	81233	82	5	17	Medium-quality	54.6
Sample_C10A_k141_272202	43158	47	19	4	Medium-quality	54.59
Sample_T8A_k141_152766	24561	40	2	0	Medium-quality	54.59
Sample_T6A_k141_271052	36506	39	13	4	Medium-quality	54.53
Sample_C6B_k141_27963	64287	70	2	48	Medium-quality	54.5
Sample_T13B_k141_25172	19311	27	15	0	Medium-quality	54.5
Sample_T16B_k141_56501	23543	36	4	1	Medium-quality	54.48
Sample_T16A_k141_18367	42943	29	0	9	Medium-quality	54.46
Sample_T6B_k141_36899	49397	49	1	39	Medium-quality	54.46
Sample_T2A_k141_89228	20597	30	13	0	Medium-quality	54.45
Sample_T7A_k141_95643	26021	33	12	0	Medium-quality	54.45
Sample_C6C_k141_147124	22689	32	10	1	Medium-quality	54.44
Sample_T15B_k141_51966	18864	9	0	4	Medium-quality	54.4
Sample_T6B_k141_130619_fragment_2	22563	27	15	0	Medium-quality	54.36
Sample_C11B_k141_21882_fragment_1	36324	40	13	10	Medium-quality	54.33
Sample_T8B_k141_260852	27025	47	9	0	Medium-quality	54.32
Sample_T2C_k141_19236	81039	56	1	38	Medium-quality	54.3
Sample_T7C_k141_90633	21640	27	1	4	Medium-quality	54.3
Sample_T16B_k141_87408	87285	124	26	0	Medium-quality	54.29
Sample_T4C_k141_113584_fragment_2	23343	34	6	1	Medium-quality	54.26
Sample_C6B_k141_137022	32423	29	9	4	Medium-quality	54.25
Sample_C2A_k141_159016	54307	45	0	41	Medium-quality	54.22
Sample_T13C_k141_80553	18914	26	11	2	Medium-quality	54.22
Sample_C6C_k141_227080	83762	82	1	35	Medium-quality	54.21
Sample_T15B_k141_39085	68166	130	13	2	Medium-quality	54.19
Sample_T7A_k141_35824	37421	59	5	4	Medium-quality	54.18
Sample_C9A_k141_99125_fragment_1	23676	29	19	0	Medium-quality	54.17
Sample_T17C_k141_30894	21343	23	11	0	Medium-quality	54.12
Sample_T7B_k141_145328	27719	36	11	0	Medium-quality	54.11
Sample_C2A_k141_167560	6143	6	1	0	Medium-quality	54.1
Sample_C4C_k141_120306	20900	15	3	0	Medium-quality	54.04
Sample_C6C_k141_221381	75474	74	1	58	Medium-quality	54.02
Sample_C8B_k141_69881_fragment_2	22659	29	17	0	Medium-quality	54.02
Sample_T5A_k141_15112	38629	22	0	6	Medium-quality	53.98
Sample_T15A_k141_85598	34549	38	1	21	Medium-quality	53.95

Sample_C1C_k141_78829	85473	95	6	9	Medium-quality	53.95
Sample_C11B_k141_51008	63896	64	2	29	Medium-quality	53.93
Sample_T2C_k141_215857_fragment_1	21200	20	4	1	Medium-quality	53.92
Sample_T17B_k141_3507	25377	29	21	0	Medium-quality	53.91
Sample_T14C_k141_52708_fragment_2	164154	168	1	9	Medium-quality	53.82
Sample_T18C_k141_93172	22029	18	7	0	Medium-quality	53.82
Sample_T12B_k141_182079	22010	28	17	0	Medium-quality	53.76
Sample_C8A_k141_136687	34420	34	1	20	Medium-quality	53.75
Sample_T6B_k141_17603_fragment_2	34599	39	8	2	Medium-quality	53.75
Sample_T2C_k141_206550	20986	18	4	0	Medium-quality	53.72
Sample_C3B_k141_98993_fragment_1	20261	25	14	0	Medium-quality	53.7
Sample_T17B_k141_34535	93927	137	22	3	Medium-quality	53.68
Sample_T3B_k141_148371	50569	55	14	10	Medium-quality	53.66
Sample_T1A_k141_544	15549	19	7	0	Medium-quality	53.65
Sample_T6B_k141_40065	28297	35	14	1	Medium-quality	53.6
Sample_C2B_k141_270783	15920	17	6	0	Medium-quality	53.58
Sample_C4A_k141_204664_fragment_1	23328	24	0	3	Medium-quality	53.57
Sample_C10C_k141_118441	37267	25	1	14	Medium-quality	53.51
Sample_C4C_k141_35203	80241	77	1	37	Medium-quality	53.4
Sample_C3C_k141_117868	20947	12	4	0	Medium-quality	53.38
Sample_C6B_k141_175277	18637	30	6	2	Medium-quality	53.36
Sample_C14A_k141_100734	25956	33	10	1	Medium-quality	53.35
Sample_C11A_k141_162967	21148	32	13	0	Medium-quality	53.34
Sample_C4C_k141_218068	31899	31	0	9	Medium-quality	53.28
Sample_C9B_k141_36912_fragment_1	27744	40	33	0	Medium-quality	53.28
Sample_T8A_k141_92665	23341	27	1	0	Medium-quality	53.22
Sample_C6C_k141_149724	26366	31	4	4	Medium-quality	53.2
Sample_C2C_k141_154494	21700	28	17	0	Medium-quality	53.17
Sample_C6A_k141_95801_fragment_1	30252	37	6	0	Medium-quality	53.17
Sample_T10A_k141_162330	32942	46	7	3	Medium-quality	53.08
Sample_T2C_k141_250314	24162	31	23	1	Medium-quality	53.06
Sample_T6B_k141_124249	28797	48	11	0	Medium-quality	53.01
Sample_T3B_k141_119750	6883	8	0	0	Medium-quality	53
Sample_T13B_k141_91562	58061	42	1	32	Medium-quality	52.98
Sample_T12C_k141_96722	20531	27	12	0	Medium-quality	52.98
Sample_T8C_k141_139655	8191	13	1	0	Medium-quality	52.96
Sample_C5C_k141_57060	21287	24	10	0	Medium-quality	52.95
Sample_C12B_k141_142269	22238	24	0	8	Medium-quality	52.91
Sample_C13B_k141_16326	26398	29	1	12	Medium-quality	52.88

Sample_C8B_k141_140600_fragment_1	23560	32	24	0	Medium-quality	52.85
Sample_T14A_k141_208845	32701	45	10	2	Medium-quality	52.84
Sample_T7A_k141_223563	21095	28	16	0	Medium-quality	52.83
Sample_C10C_k141_37715_fragment_1	24888	33	14	1	Medium-quality	52.82
Sample_C3A_k141_173087	22678	36	5	1	Medium-quality	52.79
Sample_T12A_k141_318124	39683	39	1	12	Medium-quality	52.77
Sample_T16C_k141_30958	26904	36	22	3	Medium-quality	52.77
Sample_T16B_k141_122407	68391	88	1	15	Medium-quality	52.74
Sample_T8B_k141_157621_fragment_1	57421	91	18	2	Medium-quality	52.69
Sample_T8B_k141_98967_fragment_1	24728	26	13	0	Medium-quality	52.61
Sample_T2B_k141_226299_fragment_1	6733	9	2	1	Medium-quality	52.58
Sample_T12B_k141_157152	70165	92	19	0	Medium-quality	52.57
Sample_C8A_k141_166317	30670	44	5	0	Medium-quality	52.56
Sample_T7A_k141_144307_fragment_2	23157	14	3	1	Medium-quality	52.54
Sample_C7A_k141_67810	21834	16	0	4	Medium-quality	52.5
Sample_T8C_k141_99259	16614	21	17	1	Medium-quality	52.49
Sample_C11C_k141_91386	37124	44	11	9	Medium-quality	52.48
Sample_C5C_k141_40402	88722	74	0	67	Medium-quality	52.46
Sample_T17C_k141_3415	307681	294	23	180	Medium-quality	52.44
Sample_T5B_k141_77826	39120	45	9	3	Medium-quality	52.43
Sample_C6B_k141_50502	39069	45	19	4	Medium-quality	52.42
Sample_T11C_k141_28399	7837	15	0	1	Medium-quality	52.4
Sample_T8A_k141_77934	107645	197	23	3	Medium-quality	52.4
Sample_C8B_k141_85209	23009	35	2	1	Medium-quality	52.39
Sample_T13C_k141_68229	22655	27	17	0	Medium-quality	52.39
Sample_T11B_k141_16944	20289	31	15	0	Medium-quality	52.36
Sample_C10B_k141_157019	135749	118	3	77	Medium-quality	52.35
Sample_C13A_k141_13559	97370	100	1	73	Medium-quality	52.34
Sample_T8A_k141_186121	10251	10	2	2	Medium-quality	52.31
Sample_C4C_k141_166819	24843	28	17	1	Medium-quality	52.29
Sample_C5A_k141_107357_fragment_1	22083	24	9	0	Medium-quality	52.29
Sample_T4B_k141_220445	45292	47	1	29	Medium-quality	52.26
Sample_C7B_k141_107894	24141	35	20	0	Medium-quality	52.26
Sample_T16C_k141_44993	31264	31	0	3	Medium-quality	52.22
Sample_T7A_k141_33424	24223	32	17	0	Medium-quality	52.19
Sample_T11B_k141_40537	25777	28	11	1	Medium-quality	52.18
Sample_T4C_k141_78938	30863	48	13	0	Medium-quality	52.14
Sample_T4C_k141_30162	21934	27	9	0	Medium-quality	52.12
Sample_C9C_k141_86314	24922	28	9	0	Medium-quality	52.07

Sample_T8B_k141_224230_fragment_1	32004	59	13	1	Medium-quality	52.06
Sample_C3B_k141_21027	30468	38	15	8	Medium-quality	52.04
Sample_T13B_k141_136803	19031	23	14	0	Medium-quality	52.04
Sample_T8B_k141_236104	7438	13	1	0	Medium-quality	52.01
Sample_C3C_k141_211170	29964	45	8	1	Medium-quality	51.99
Sample_C8B_k141_198503	27688	33	26	2	Medium-quality	51.99
Sample_C8C_k141_6024	17796	24	13	0	Medium-quality	51.98
Sample_T16C_k141_82389	9424	10	4	0	Medium-quality	51.96
Sample_C4B_k141_32952	20327	33	11	2	Medium-quality	51.95
Sample_C4C_k141_242362	29811	22	3	3	Medium-quality	51.92
Sample_T10B_k141_18626	35587	42	1	11	Medium-quality	51.85
Sample_C6B_k141_147467	25521	27	3	2	Medium-quality	51.85
Sample_C13C_k141_93875	34260	26	1	10	Medium-quality	51.84
Sample_T10C_k141_29057	43112	51	9	5	Medium-quality	51.84
Sample_C10B_k141_53584	19291	28	8	0	Medium-quality	51.83
Sample_T7B_k141_58920	62635	56	11	26	Medium-quality	51.82
Sample_T12B_k141_120816_fragment_1	21167	31	12	0	Medium-quality	51.79
Sample_T6B_k141_41325	169028	143	2	101	Medium-quality	51.78
Sample_T11A_k141_68611	37769	28	1	14	Medium-quality	51.76
Sample_C13A_k141_3768_fragment_1	18005	22	10	0	Medium-quality	51.74
Sample_T12A_k141_141700	23509	39	10	0	Medium-quality	51.73
Sample_C2B_k141_124462	24478	25	9	1	Medium-quality	51.68
Sample_C12A_k141_40579	20504	27	14	0	Medium-quality	51.63
Sample_T12B_k141_96687_fragment_1	120566	114	16	3	Medium-quality	51.57
Sample_T12A_k141_185044	21537	21	11	0	Medium-quality	51.56
Sample_C10C_k141_174420	42661	57	2	14	Medium-quality	51.54
Sample_C8A_k141_29379	21774	40	13	0	Medium-quality	51.51
Sample_C2A_k141_44674	23177	42	15	0	Medium-quality	51.5
Sample_T3C_k141_23862_fragment_1	23559	31	23	0	Medium-quality	51.5
Sample_C9C_k141_23853_fragment_1	23166	16	3	0	Medium-quality	51.49
Sample_C11A_k141_1637	137055	130	4	62	Medium-quality	51.48
Sample_T6A_k141_202900	94146	94	4	8	Medium-quality	51.46
Sample_C12A_k141_3735	16755	24	10	0	Medium-quality	51.44
Sample_T5A_k141_58579_fragment_1	37319	50	21	0	Medium-quality	51.34
Sample_T2B_k141_143454	17670	30	16	1	Medium-quality	51.29
Sample_T6B_k141_171378	27818	40	12	0	Medium-quality	51.28
Sample_T10A_k141_75015	16793	21	13	1	Medium-quality	51.25
Sample_C3C_k141_57131	19587	28	11	0	Medium-quality	51.23
Sample_C11A_k141_40478	98886	177	20	9	Medium-quality	51.19

Sample_C5C_k141_110013	21365	31	16	0	Medium-quality	51.17
Sample_C10A_k141_39315	44339	46	1	10	Medium-quality	51.16
Sample_C12C_k141_128626_fragment_1	19992	33	16	1	Medium-quality	51.15
Sample_C4A_k141_134253	23911	42	4	1	Medium-quality	51.1
Sample_T8B_k141_263659	15847	18	6	0	Medium-quality	51.09
Sample_T12C_k141_59375	29428	49	9	1	Medium-quality	51.07
Sample_T6A_k141_164248	19562	24	3	0	Medium-quality	51.03
Sample_T10B_k141_134585	131905	130	2	19	Medium-quality	50.95
Sample_C4B_k141_114680	7169	13	0	0	Medium-quality	50.94
Sample_C14C_k141_160769	23243	25	4	0	Medium-quality	50.92
Sample_C7C_k141_38420_fragment_1	33885	50	16	1	Medium-quality	50.9
Sample_C11C_k141_128759	22450	20	0	3	Medium-quality	50.89
Sample_C10A_k141_235752	103268	121	2	97	Medium-quality	50.88
Sample_T11A_k141_153660	24766	20	0	2	Medium-quality	50.8
Sample_T10A_k141_9386	17760	22	13	0	Medium-quality	50.79
Sample_T10A_k141_114684	13602	12	0	5	Medium-quality	50.78
Sample_T5B_k141_59532	33535	30	1	6	Medium-quality	50.74
Sample_C14C_k141_85472	24339	35	15	0	Medium-quality	50.73
Sample_T11B_k141_145563	163695	131	17	83	Medium-quality	50.7
Sample_T17A_k141_23531	38653	39	0	11	Medium-quality	50.7
Sample_C3A_k141_2041	20687	31	17	0	Medium-quality	50.69
Sample_T12A_k141_85234	9680	11	7	0	Medium-quality	50.62
Sample_T11C_k141_39380	42505	35	1	3	Medium-quality	50.61
Sample_T5C_k141_176725	21265	23	0	15	Medium-quality	50.6
Sample_T17B_k141_9308	20625	28	14	0	Medium-quality	50.6
Sample_T14A_k141_96193	36091	48	1	20	Medium-quality	50.59
Sample_T2A_k141_135818	18525	27	3	2	Medium-quality	50.57
Sample_T5A_k141_53982	19561	25	9	1	Medium-quality	50.57
Sample_T12A_k141_252977	20482	18	12	0	Medium-quality	50.56
Sample_T11C_k141_164548	24238	32	1	10	Medium-quality	50.54
Sample_C7C_k141_54611	50909	67	16	5	Medium-quality	50.51
Sample_C11B_k141_185575	19920	31	5	0	Medium-quality	50.46
Sample_T15A_k141_110833	20057	26	9	1	Medium-quality	50.45
Sample_C13A_k141_68869	30197	34	1	11	Medium-quality	50.44
Sample_C4B_k141_178567_fragment_1	30882	30	21	0	Medium-quality	50.43
Sample_T6B_k141_171406	22358	36	7	0	Medium-quality	50.42
Sample_C10A_k141_94820	18329	21	14	0	Medium-quality	50.39
Sample_T2C_k141_66727_fragment_1	22713	32	24	0	Medium-quality	50.39
Sample_C6C_k141_7578	17293	25	11	0	Medium-quality	50.38

Sample_C9C_k141_26178	18971	31	8	1	Medium-quality	50.37
Sample_T5A_k141_44063_fragment_1	20169	29	5	1	Medium-quality	50.37
Sample_T13B_k141_2912	6797	9	1	2	Medium-quality	50.36
Sample_T7B_k141_83223	19589	28	16	0	Medium-quality	50.35
Sample_T10A_k141_59353	6762	9	1	0	Medium-quality	50.33
Sample_T16C_k141_18585	269593	224	17	154	Medium-quality	50.33
Sample_T12A_k141_344764	10655	16	1	0	Medium-quality	50.32
Sample_C13A_k141_41495	41009	43	1	6	Medium-quality	50.31
Sample_T2A_k141_231166	92692	88	1	27	Medium-quality	50.27
Sample_C13C_k141_2926	20296	23	17	0	Medium-quality	50.26
Sample_T5C_k141_103063_fragment_1	30977	51	9	0	Medium-quality	50.26
Sample_C9A_k141_50067	21660	27	12	1	Medium-quality	50.19
Sample_T12B_k141_90895	64371	117	4	5	Medium-quality	50.18
Sample_C2C_k141_240077	42867	39	0	25	Medium-quality	50.17
Sample_T13B_k141_101758_fragment_1	9065	10	2	0	Medium-quality	50.14
Sample_C10C_k141_175679	19031	24	11	0	Medium-quality	50.13
Sample_C2A_k141_169782	82770	90	23	1	Medium-quality	50.06
Sample_C10C_k141_100557_fragment_1	25277	30	17	2	Medium-quality	50.03
Sample_T14A_k141_138340	77299	83	1	53	Medium-quality	50.02
Sample_C5A_k141_164826	6429	10	2	0	Medium-quality	50.02
Sample_T4B_k141_119286	47570	63	7	12	Low-quality	49.95
Sample_T10A_k141_56109	18607	21	2	0	Low-quality	49.94
Sample_C11A_k141_39108	36069	54	17	1	Low-quality	49.94
Sample_C3B_k141_34198	30204	59	10	0	Low-quality	49.94
Sample_C3C_k141_224776_fragment_1	25453	35	20	0	Low-quality	49.9
Sample_T8C_k141_101186	29085	47	20	5	Low-quality	49.89
Sample_T5B_k141_35294	18228	15	0	8	Low-quality	49.87
Sample_C10A_k141_296053	24079	31	11	0	Low-quality	49.86
Sample_C3C_k141_39278	19973	33	16	0	Low-quality	49.86
Sample_C13C_k141_45196	29896	42	13	2	Low-quality	49.85
Sample_T12B_k141_63878	22473	30	12	0	Low-quality	49.85
Sample_T18C_k141_90472	19018	17	0	5	Low-quality	49.79
Sample_T7A_k141_76771	49371	89	11	0	Low-quality	49.79
Sample_C8C_k141_72314_fragment_2	18845	31	4	0	Low-quality	49.76
Sample_C8B_k141_38612	25310	14	0	11	Low-quality	49.73
Sample_C1A_k141_210819_fragment_2	18236	29	9	1	Low-quality	49.73
Sample_T8C_k141_88391	14640	17	9	0	Low-quality	49.73
Sample_C1A_k141_1122	30447	33	3	2	Low-quality	49.7
Sample_T17A_k141_2713	44020	32	0	9	Low-quality	49.7

Sample_C11A_k141_89314	14257	8	0	2	Low-quality	49.7
Sample_C11A_k141_105778	30093	42	9	0	Low-quality	49.7
Sample_C8B_k141_96704	22761	34	22	2	Low-quality	49.69
Sample_T16B_k141_114686	22037	29	10	0	Low-quality	49.66
Sample_T16B_k141_6288_fragment_1	21232	36	10	0	Low-quality	49.65
Sample_T11C_k141_81245	64549	59	2	10	Low-quality	49.59
Sample_T16B_k141_35521	18956	29	9	0	Low-quality	49.58
Sample_C5C_k141_64645	42561	37	1	13	Low-quality	49.57
Sample_T7A_k141_57170	82793	104	2	6	Low-quality	49.57
Sample_C13C_k141_3038	20157	26	22	0	Low-quality	49.56
Sample_C12C_k141_137156_fragment_1	22500	32	8	2	Low-quality	49.52
Sample_T15B_k141_63437_fragment_1	42256	44	20	3	Low-quality	49.51
Sample_C10C_k141_144279	6655	11	2	0	Low-quality	49.5
Sample_C14C_k141_152686	64560	46	1	23	Low-quality	49.48
Sample_C8B_k141_165164	20054	22	6	1	Low-quality	49.47
Sample_C12A_k141_67165_fragment_1	20729	27	7	0	Low-quality	49.46
Sample_T3A_k141_85155	23177	28	13	1	Low-quality	49.45
Sample_C8C_k141_42328	31362	27	1	2	Low-quality	49.37
Sample_T4C_k141_53040_fragment_1	20583	33	14	0	Low-quality	49.31
Sample_T14B_k141_93219_fragment_1	9050	12	1	2	Low-quality	49.28
Sample_C6A_k141_95850	71955	59	1	38	Low-quality	49.27
Sample_C7C_k141_26807	16835	20	6	0	Low-quality	49.26
Sample_C2B_k141_145826_fragment_2	67563	90	11	13	Low-quality	49.24
Sample_C8A_k141_62293	24984	28	8	0	Low-quality	49.2
Sample_T12A_k141_97928	10129	11	0	1	Low-quality	49.2
Sample_T4B_k141_48805	16185	41	2	0	Low-quality	49.2
Sample_T7B_k141_141747_fragment_1	46269	52	12	8	Low-quality	49.2
Sample_C5A_k141_62363	71399	59	2	8	Low-quality	49.14
Sample_T8C_k141_147686_fragment_4	16358	19	0	2	Low-quality	49.13
Sample_T8B_k141_36300	72986	73	1	40	Low-quality	49.06
Sample_C13C_k141_110405	15627	17	7	0	Low-quality	49.05
Sample_C6C_k141_151551	26233	35	7	1	Low-quality	49.04
Sample_T5C_k141_24191	17309	21	14	0	Low-quality	49.04
Sample_T6A_k141_270967	34860	30	7	1	Low-quality	49.04
Sample_T16A_k141_25269	20592	19	0	7	Low-quality	49
Sample_T6B_k141_107824	14136	16	5	0	Low-quality	49
Sample_C4A_k141_242001	36711	32	0	9	Low-quality	48.97
Sample_T8A_k141_54326	28793	31	7	1	Low-quality	48.92
Sample_T13A_k141_90771	5842	8	1	0	Low-quality	48.91

Sample_C10C_k141_215216	28238	32	23	0	Low-quality	48.9
Sample_T8C_k141_217448	70131	63	1	51	Low-quality	48.89
Sample_C3A_k141_128703	24583	28	6	0	Low-quality	48.89
Sample_T5B_k141_47440	32301	32	1	17	Low-quality	48.88
Sample_C4C_k141_118200	23148	30	9	1	Low-quality	48.82
Sample_T10B_k141_158399	16097	20	6	0	Low-quality	48.79
Sample_C2B_k141_200821	19998	26	11	0	Low-quality	48.77
Sample_T5A_k141_54773	27171	31	1	6	Low-quality	48.72
Sample_T3B_k141_131112	16062	20	6	0	Low-quality	48.68
Sample_C12A_k141_134075	6972	13	2	0	Low-quality	48.59
Sample_T8B_k141_91729_fragment_2	52952	51	16	1	Low-quality	48.59
Sample_C10A_k141_272784	38083	62	4	1	Low-quality	48.55
Sample_T4B_k141_4606	21000	32	2	2	Low-quality	48.53
Sample_T2A_k141_278004	38324	26	0	9	Low-quality	48.49
Sample_T10A_k141_163153	18806	33	12	0	Low-quality	48.48
Sample_C10A_k141_109985	22170	25	22	0	Low-quality	48.43
Sample_T8B_k141_172925	21704	40	5	1	Low-quality	48.41
Sample_T2B_k141_204627	14106	19	8	0	Low-quality	48.4
Sample_C1A_k141_56233_fragment_1	18777	34	10	1	Low-quality	48.38
Sample_T12C_k141_171378	35890	61	6	2	Low-quality	48.38
Sample_C10C_k141_165559	29549	48	18	4	Low-quality	48.37
Sample_C13C_k141_9081	23160	44	5	1	Low-quality	48.36
Sample_T10A_k141_122527	22476	23	1	7	Low-quality	48.35
Sample_T16C_k141_59620	30824	49	9	2	Low-quality	48.35
Sample_T8B_k141_95802_fragment_1	21724	22	3	0	Low-quality	48.35
Sample_T16B_k141_141014	45528	42	0	26	Low-quality	48.32
Sample_C11A_k141_6903	20787	28	2	0	Low-quality	48.32
Sample_T6A_k141_31047	36756	36	0	11	Low-quality	48.23
Sample_C4A_k141_129796	16653	34	10	0	Low-quality	48.22
Sample_T13B_k141_73642	23525	23	0	21	Low-quality	48.21
Sample_C10A_k141_174165	18316	43	4	1	Low-quality	48.21
Sample_T8C_k141_183150	27580	31	8	1	Low-quality	48.21
Sample_C12B_k141_115942	15844	24	8	1	Low-quality	48.2
Sample_C2A_k141_298705	98259	105	1	44	Low-quality	48.19
Sample_T10A_k141_156587	20055	31	2	2	Low-quality	48.19
Sample_T17C_k141_26367	34935	58	11	4	Low-quality	48.19
Sample_T18B_k141_76007	50931	66	18	8	Low-quality	48.15
Sample_C6C_k141_4022	22457	28	9	1	Low-quality	48.09
Sample_T12A_k141_331341	20197	20	0	15	Low-quality	48.06

Sample_T4B_k141_133806	118325	104	1	52	Low-quality	48.06
Sample_T15B_k141_1924	16935	20	15	0	Low-quality	48.06
Sample_T2B_k141_157437_fragment_1	6336	9	0	1	Low-quality	48.01
Sample_T13C_k141_15397_fragment_1	43203	38	1	9	Low-quality	48
Sample_T8A_k141_186548	20244	13	1	4	Low-quality	47.98
Sample_C9B_k141_78673	22076	33	17	0	Low-quality	47.98
Sample_C6C_k141_7511	19961	31	10	0	Low-quality	47.93
Sample_T12C_k141_14922_fragment_1	30579	46	11	2	Low-quality	47.93
Sample_C12B_k141_155377_fragment_2	69561	86	7	15	Low-quality	47.91
Sample_T4B_k141_319340_fragment_1	17731	35	11	0	Low-quality	47.9
Sample_T10C_k141_88456	16784	22	20	0	Low-quality	47.88
Sample_T13B_k141_102866	83413	125	20	6	Low-quality	47.86
Sample_C12C_k141_153536_fragment_2	18233	28	4	0	Low-quality	47.83
Sample_T7A_k141_174766	28144	56	9	1	Low-quality	47.82
Sample_T11C_k141_119364	7035	12	1	0	Low-quality	47.79
Sample_T8A_k141_209147_fragment_1	28558	47	8	0	Low-quality	47.79
Sample_C13A_k141_76836	28004	30	6	3	Low-quality	47.76
Sample_C1B_k141_111283	37998	66	22	1	Low-quality	47.72
Sample_C7C_k141_43843	16327	31	9	0	Low-quality	47.72
Sample_T5B_k141_51397	81845	68	2	16	Low-quality	47.69
Sample_T7C_k141_37948_fragment_1	18636	28	13	1	Low-quality	47.69
Sample_C4B_k141_179835_fragment_1	19110	39	11	0	Low-quality	47.68
Sample_T12C_k141_78449	44847	44	17	3	Low-quality	47.66
Sample_T11C_k141_68142	16591	21	5	1	Low-quality	47.65
Sample_C2B_k141_217231	15912	23	4	1	Low-quality	47.64
Sample_C1C_k141_59097_fragment_1	8377	13	1	1	Low-quality	47.62
Sample_C5B_k141_12056	17671	23	13	0	Low-quality	47.62
Sample_T17A_k141_28511	14884	19	8	0	Low-quality	47.61
Sample_T8C_k141_4521	36044	32	1	11	Low-quality	47.48
Sample_C5B_k141_80111	17460	41	9	1	Low-quality	47.47
Sample_T4B_k141_182382	15851	22	4	0	Low-quality	47.45
Sample_T10A_k141_101326	16307	28	12	0	Low-quality	47.37
Sample_C11C_k141_184028_fragment_2	43384	43	7	3	Low-quality	47.34
Sample_T13A_k141_121109	81195	76	10	3	Low-quality	47.33
Sample_T17C_k141_18934	24892	31	17	0	Low-quality	47.32
Sample_C2B_k141_28309	145242	176	1	82	Low-quality	47.31
Sample_T12C_k141_66820	23873	25	0	4	Low-quality	47.3
Sample_T2C_k141_185506	13450	22	3	1	Low-quality	47.29
Sample_T17B_k141_5073	24801	35	30	0	Low-quality	47.25

Sample_C9A_k141_118084	22565	28	11	0	Low-quality	47.23
Sample_C2C_k141_193919	73639	69	1	22	Low-quality	47.18
Sample_T5B_k141_66179	8654	13	2	0	Low-quality	47.13
Sample_T17C_k141_28140	293346	279	1	209	Low-quality	47.12
Sample_T2C_k141_204294	15577	21	7	1	Low-quality	47.12
Sample_C12C_k141_53332	39557	40	1	12	Low-quality	47.1
Sample_C5C_k141_113550	33650	23	1	7	Low-quality	47.09
Sample_T6A_k141_291360	19074	33	6	0	Low-quality	47.05
Sample_C2A_k141_46414	84021	88	1	41	Low-quality	47.04
Sample_T15B_k141_117079	20540	18	2	2	Low-quality	47.03
Sample_T4B_k141_313698	131690	159	1	76	Low-quality	47.02
Sample_T15C_k141_58470	73618	81	2	8	Low-quality	47.02
Sample_C13B_k141_83931	145796	123	3	71	Low-quality	47
Sample_C13C_k141_48401	21519	31	15	0	Low-quality	46.99
Sample_C3A_k141_145813	35422	30	1	10	Low-quality	46.96
Sample_T4B_k141_224651	24049	24	0	8	Low-quality	46.96
Sample_T12A_k141_96873	87982	96	3	10	Low-quality	46.95
Sample_T2A_k141_156053	116361	131	1	74	Low-quality	46.89
Sample_T2B_k141_166314	21552	29	8	3	Low-quality	46.83
Sample_T10B_k141_13315	19434	22	9	0	Low-quality	46.83
Sample_C10B_k141_22829	18215	23	11	0	Low-quality	46.8
Sample_T7A_k141_101206	34797	32	1	20	Low-quality	46.76
Sample_C7B_k141_95249	87615	109	1	39	Low-quality	46.74
Sample_C7B_k141_70692	41594	26	1	12	Low-quality	46.69
Sample_T6C_k141_184943	17895	12	0	1	Low-quality	46.68
Sample_C3C_k141_34484_fragment_1	18534	31	9	0	Low-quality	46.68
Sample_C2B_k141_41157	60909	82	1	53	Low-quality	46.67
Sample_C6A_k141_70705	42790	28	0	22	Low-quality	46.65
Sample_T5B_k141_18743	76937	80	2	31	Low-quality	46.65
Sample_C7A_k141_143286	19049	21	14	0	Low-quality	46.64
Sample_T7A_k141_93041	17048	18	5	1	Low-quality	46.62
Sample_T4C_k141_1751	18417	36	6	1	Low-quality	46.62
Sample_T2A_k141_226062	37931	31	0	10	Low-quality	46.61
Sample_T6B_k141_55086	34725	30	0	6	Low-quality	46.57
Sample_T8B_k141_176780_fragment_1	24875	33	6	2	Low-quality	46.57
Sample_T16B_k141_98100	16451	20	7	0	Low-quality	46.53
Sample_T8B_k141_66698	35072	36	0	18	Low-quality	46.52
Sample_C11B_k141_158739	19615	21	2	0	Low-quality	46.49
Sample_C3A_k141_207314	26786	37	9	0	Low-quality	46.49

Sample_T12B_k141_116145	24927	26	6	0	Low-quality	46.46
Sample_T8C_k141_92770	16830	22	10	0	Low-quality	46.45
Sample_C8C_k141_49517	16715	23	12	2	Low-quality	46.44
Sample_T7B_k141_94325_fragment_1	17751	31	7	0	Low-quality	46.44
Sample_T3A_k141_114668	19094	26	2	9	Low-quality	46.41
Sample_C13A_k141_110368	16851	27	9	0	Low-quality	46.41
Sample_T7C_k141_147364	38968	33	1	12	Low-quality	46.4
Sample_T12B_k141_94171	28174	30	13	0	Low-quality	46.39
Sample_C9A_k141_129948	15965	16	0	7	Low-quality	46.37
Sample_T12B_k141_25296_fragment_1	16539	30	8	1	Low-quality	46.35
Sample_T8B_k141_59749_fragment_1	20600	20	2	0	Low-quality	46.27
Sample_T4B_k141_138653	19477	12	0	6	Low-quality	46.25
Sample_T8A_k141_115066	48333	48	0	14	Low-quality	46.25
Sample_C5A_k141_62265_fragment_1	24660	26	5	2	Low-quality	46.21
Sample_C6B_k141_93582	32883	38	2	22	Low-quality	46.2
Sample_T12A_k141_72850	8484	7	1	3	Low-quality	46.2
Sample_T5C_k141_105820	23966	32	16	0	Low-quality	46.18
Sample_T15B_k141_94611	17418	30	7	1	Low-quality	46.16
Sample_T15B_k141_8116	35077	53	10	4	Low-quality	46.14
Sample_C4A_k141_139383	25108	31	12	0	Low-quality	46.11
Sample_T12B_k141_176900_fragment_1	62881	74	2	4	Low-quality	46.08
Sample_T6B_k141_111078	35470	31	0	10	Low-quality	46.07
Sample_T16B_k141_172628_fragment_1	28706	32	4	1	Low-quality	46.07
Sample_T11C_k141_63062	21379	28	2	4	Low-quality	46.02
Sample_C8C_k141_16396	17507	23	3	0	Low-quality	46.01
Sample_T8B_k141_72041	13198	8	0	2	Low-quality	46
Sample_C6B_k141_25814	21660	28	10	0	Low-quality	46
Sample_C8C_k141_39763	18275	30	17	0	Low-quality	45.96
Sample_C5A_k141_236732	113958	104	1	68	Low-quality	45.92
Sample_T18C_k141_37826	17907	22	12	0	Low-quality	45.9
Sample_C2B_k141_341308	113770	102	1	49	Low-quality	45.85
Sample_C8A_k141_65444	36464	65	9	0	Low-quality	45.85
Sample_C2B_k141_221176	73076	85	1	57	Low-quality	45.84
Sample_C11B_k141_81943	16339	19	13	0	Low-quality	45.84
Sample_T16C_k141_57146_fragment_1	17911	22	11	1	Low-quality	45.84
Sample_T6B_k141_109451_fragment_2	24480	28	18	2	Low-quality	45.8
Sample_C8A_k141_48160	25533	21	0	9	Low-quality	45.79
Sample_C6B_k141_4114	16795	21	11	0	Low-quality	45.77
Sample_T2A_k141_256856	26920	45	8	0	Low-quality	45.76

Sample_C6B_k141_44859	21267	12	0	4	Low-quality	45.75
Sample_C4A_k141_32357	15272	21	4	0	Low-quality	45.72
Sample_C14A_k141_216323	18439	35	4	3	Low-quality	45.71
Sample_T8B_k141_208833	21247	24	0	9	Low-quality	45.7
Sample_T16B_k141_50481_fragment_1	15321	27	2	0	Low-quality	45.7
Sample_T3B_k141_181868	15263	22	4	0	Low-quality	45.69
Sample_C3A_k141_59156	20535	23	3	0	Low-quality	45.67
Sample_C10B_k141_150894	30404	20	1	4	Low-quality	45.64
Sample_C6C_k141_22781	15825	8	0	4	Low-quality	45.64
Sample_T10A_k141_119291	17430	20	0	3	Low-quality	45.61
Sample_C10A_k141_127748	13626	17	3	0	Low-quality	45.58
Sample_T18B_k141_32704	55505	54	2	26	Low-quality	45.57
Sample_C11C_k141_156554	26778	29	9	2	Low-quality	45.55
Sample_T3A_k141_130803	18264	18	12	0	Low-quality	45.55
Sample_T4B_k141_285578	16179	20	11	0	Low-quality	45.54
Sample_T7A_k141_124777	25378	29	0	11	Low-quality	45.51
Sample_T15B_k141_144849	13596	18	3	0	Low-quality	45.48
Sample_T7B_k141_84770	35982	51	11	2	Low-quality	45.47
Sample_T15C_k141_35225	113541	113	2	59	Low-quality	45.46
Sample_T15A_k141_119623_fragment_3	81433	90	17	4	Low-quality	45.46
Sample_T12B_k141_158983	24284	27	20	0	Low-quality	45.43
Sample_T5C_k141_51095	18453	22	15	0	Low-quality	45.42
Sample_C4B_k141_209159	8651	12	1	1	Low-quality	45.4
Sample_C2B_k141_164917	18855	29	7	0	Low-quality	45.39
Sample_T5B_k141_74936	22758	26	2	1	Low-quality	45.37
Sample_C1C_k141_79005	83632	76	1	44	Low-quality	45.36
Sample_C8A_k141_107199	35836	59	6	3	Low-quality	45.35
Sample_C10A_k141_173287	12607	19	2	0	Low-quality	45.35
Sample_T2A_k141_227610	22674	38	9	0	Low-quality	45.35
Sample_T13B_k141_157070	17692	28	0	0	Low-quality	45.34
Sample_C12C_k141_48058_fragment_1	7891	11	0	0	Low-quality	45.3
Sample_T13B_k141_33721	69954	55	1	50	Low-quality	45.27
Sample_T5A_k141_49012	19039	14	0	3	Low-quality	45.27
Sample_C2A_k141_84756	20207	22	19	0	Low-quality	45.26
Sample_T16C_k141_127643_fragment_2	80725	90	9	5	Low-quality	45.26
Sample_T6C_k141_159023_fragment_1	36215	57	14	0	Low-quality	45.23
Sample_T12C_k141_98745_fragment_1	32795	42	13	3	Low-quality	45.22
Sample_T15C_k141_36628_fragment_1	36952	48	7	3	Low-quality	45.19
Sample_T6B_k141_147105	27434	49	15	0	Low-quality	45.18

Sample_T2C_k141_195309	20361	26	7	1	Low-quality	45.13
Sample_T14B_k141_146336	21706	26	13	1	Low-quality	45.11
Sample_T7A_k141_116000	29068	37	9	2	Low-quality	45.1
Sample_T16B_k141_161433	19551	34	9	1	Low-quality	45.09
Sample_T6A_k141_249226	8332	6	2	0	Low-quality	45.09
Sample_T5B_k141_6916	17027	19	4	0	Low-quality	45.07
Sample_T5B_k141_36162_fragment_1	18049	20	14	0	Low-quality	45.06
Sample_C4C_k141_244411	18906	18	0	10	Low-quality	44.99
Sample_T15A_k141_57243	51404	54	21	12	Low-quality	44.98
Sample_T5B_k141_18247	72521	64	2	32	Low-quality	44.97
Sample_C8B_k141_43805	19918	36	8	1	Low-quality	44.97
Sample_T10A_k141_48589_fragment_2	22583	26	13	1	Low-quality	44.96
Sample_C11C_k141_141569	27762	44	9	1	Low-quality	44.95
Sample_T7C_k141_73264	12890	17	2	1	Low-quality	44.93
Sample_T18B_k141_75908	18828	26	12	0	Low-quality	44.9
Sample_C7B_k141_67565	25159	46	5	0	Low-quality	44.87
Sample_T8A_k141_63313	30514	37	0	6	Low-quality	44.86
Sample_C9B_k141_72948	29150	55	5	0	Low-quality	44.86
Sample_T17A_k141_22426_fragment_1	24641	40	23	1	Low-quality	44.86
Sample_T8B_k141_259934	19285	12	3	0	Low-quality	44.85
Sample_C11A_k141_1635	17419	18	0	5	Low-quality	44.82
Sample_C10A_k141_284231	38030	33	0	23	Low-quality	44.77
Sample_T10A_k141_84988	21669	36	4	3	Low-quality	44.77
Sample_C11B_k141_56745	20955	18	4	1	Low-quality	44.75
Sample_C1B_k141_187257	21660	21	4	1	Low-quality	44.75
Sample_T17C_k141_29334	19275	25	3	0	Low-quality	44.74
Sample_T16C_k141_122191	19381	30	11	1	Low-quality	44.73
Sample_T5C_k141_64978	13335	18	4	0	Low-quality	44.72
Sample_C2C_k141_114439	49500	46	1	28	Low-quality	44.7
Sample_C9B_k141_48273	14988	30	0	0	Low-quality	44.69
Sample_C10A_k141_292912	20671	22	5	0	Low-quality	44.67
Sample_T8C_k141_130900_fragment_1	30468	33	12	4	Low-quality	44.67
Sample_C10A_k141_265	15966	23	6	0	Low-quality	44.65
Sample_T7B_k141_140566	17567	20	7	0	Low-quality	44.65
Sample_T4B_k141_62907	18155	32	4	2	Low-quality	44.58
Sample_C13A_k141_105681	138268	124	3	68	Low-quality	44.57
Sample_T8C_k141_105931	17945	22	12	0	Low-quality	44.56
Sample_T5C_k141_30278	19623	31	3	1	Low-quality	44.54
Sample_C3B_k141_79547	16392	32	7	1	Low-quality	44.54

Sample_C12A_k141_50703	172317	158	1	107	Low-quality	44.53
Sample_C8B_k141_163293_fragment_1	27376	54	12	3	Low-quality	44.48
Sample_C13A_k141_40969	27466	49	10	0	Low-quality	44.4
Sample_T4B_k141_278302	15719	19	13	0	Low-quality	44.37
Sample_C11C_k141_7640_fragment_1	65370	98	16	4	Low-quality	44.36
Sample_C3C_k141_20700	19646	13	0	2	Low-quality	44.35
Sample_T2B_k141_11145	32997	27	1	15	Low-quality	44.29
Sample_C4B_k141_262412	17778	20	7	0	Low-quality	44.29
Sample_T14B_k141_89547	90264	80	1	31	Low-quality	44.27
Sample_C1C_k141_127072	24673	21	0	4	Low-quality	44.24
Sample_C2B_k141_170454	13216	16	3	1	Low-quality	44.21
Sample_C5A_k141_274883	35676	31	0	23	Low-quality	44.2
Sample_T11B_k141_153152_fragment_1	28018	41	13	4	Low-quality	44.19
Sample_T12A_k141_324203	6881	7	0	0	Low-quality	44.18
Sample_C2B_k141_216583	19444	10	1	4	Low-quality	44.16
Sample_C9B_k141_36822_fragment_1	20666	23	4	3	Low-quality	44.16
Sample_C4B_k141_139958	5793	8	0	2	Low-quality	44.13
Sample_T10A_k141_168346_fragment_1	15317	23	15	0	Low-quality	44.12
Sample_C11A_k141_101558	26497	43	11	0	Low-quality	44.11
Sample_C6C_k141_185290	32945	19	1	6	Low-quality	44.08
Sample_C6A_k141_51785	37964	54	6	0	Low-quality	44.08
Sample_C7A_k141_102867	22202	30	6	2	Low-quality	44.08
Sample_T10B_k141_63573	21238	28	15	0	Low-quality	44.08
Sample_C11B_k141_117392	22051	25	19	1	Low-quality	44.05
Sample_C3A_k141_1366	20717	25	16	0	Low-quality	44.05
Sample_T17A_k141_9422	20633	29	12	1	Low-quality	44.05
Sample_C7C_k141_74349_fragment_1	15847	25	13	0	Low-quality	44.04
Sample_T4B_k141_222861	17047	14	4	0	Low-quality	44.04
Sample_C8C_k141_167719	21346	40	6	2	Low-quality	44
Sample_T11A_k141_41424	81082	77	1	9	Low-quality	43.98
Sample_T11B_k141_54244	238242	226	1	172	Low-quality	43.98
Sample_T5A_k141_6021	102084	126	2	7	Low-quality	43.96
Sample_T11C_k141_9837	68722	78	2	7	Low-quality	43.92
Sample_T6B_k141_27068_fragment_1	21729	32	5	0	Low-quality	43.89
Sample_C1C_k141_24547	18639	34	9	1	Low-quality	43.88
Sample_C4B_k141_87861	16105	27	3	3	Low-quality	43.88
Sample_C8A_k141_150140	32799	26	1	12	Low-quality	43.85
Sample_T12A_k141_309340	86379	80	1	55	Low-quality	43.85
Sample_T15C_k141_1225	18002	33	6	1	Low-quality	43.85

Sample_T3A_k141_90623	31131	17	0	7	Low-quality	43.84
Sample_C13A_k141_89256	18409	16	0	16	Low-quality	43.8
Sample_T4B_k141_281745	19807	25	9	0	Low-quality	43.73
Sample_T16B_k141_70342	19265	27	0	3	Low-quality	43.71
Sample_C12A_k141_28397	16734	17	1	5	Low-quality	43.69
Sample_C4C_k141_58973	35965	30	0	22	Low-quality	43.69
Sample_T6B_k141_101500	88702	164	22	6	Low-quality	43.67
Sample_T14C_k141_93824	469522	421	2	282	Low-quality	43.62
Sample_T4B_k141_117096	26154	47	10	0	Low-quality	43.59
Sample_C14A_k141_132972	17509	22	13	1	Low-quality	43.57
Sample_T7A_k141_39079	32780	36	6	0	Low-quality	43.56
Sample_C13C_k141_12478	26073	32	1	2	Low-quality	43.55
Sample_C11C_k141_155095_fragment_1	64325	76	19	0	Low-quality	43.55
Sample_T12A_k141_13497	28760	26	1	2	Low-quality	43.52
Sample_T11C_k141_5366	235630	218	1	156	Low-quality	43.5
Sample_T12A_k141_339899	26040	29	0	5	Low-quality	43.49
Sample_T12A_k141_279871_fragment_2	16573	33	9	0	Low-quality	43.49
Sample_T11A_k141_136002	15423	23	12	1	Low-quality	43.48
Sample_T10A_k141_75290	20189	18	0	15	Low-quality	43.47
Sample_C10C_k141_196360	17483	21	9	1	Low-quality	43.46
Sample_C5C_k141_3022	26699	40	11	2	Low-quality	43.46
Sample_T6A_k141_151134	20084	25	1	2	Low-quality	43.43
Sample_C4C_k141_8186	20449	39	7	0	Low-quality	43.42
Sample_T17C_k141_35585	43555	40	0	28	Low-quality	43.4
Sample_T8A_k141_178365_fragment_1	20334	26	11	1	Low-quality	43.4
Sample_C5B_k141_57613	144567	134	1	96	Low-quality	43.37
Sample_C6C_k141_175038	64710	74	1	36	Low-quality	43.37
Sample_T12C_k141_76012	16769	27	4	1	Low-quality	43.37
Sample_T11A_k141_118599	20649	28	1	6	Low-quality	43.34
Sample_C10A_k141_84286	6699	11	1	0	Low-quality	43.32
Sample_T13B_k141_128565	27103	38	8	3	Low-quality	43.31
Sample_C5A_k141_285182	57247	64	1	4	Low-quality	43.26
Sample_C12A_k141_25636	42880	44	9	8	Low-quality	43.24
Sample_T17A_k141_38345	42709	37	1	22	Low-quality	43.22
Sample_T8B_k141_57790_fragment_1	21433	22	0	2	Low-quality	43.2
Sample_T6B_k141_159898	76390	80	1	36	Low-quality	43.16
Sample_T3B_k141_69648	19090	31	8	3	Low-quality	43.15
Sample_T6C_k141_169285	18959	27	8	4	Low-quality	43.15
Sample_C10A_k141_299103	17191	22	4	0	Low-quality	43.13

Sample_C12A_k141_123806	37257	47	2	8	Low-quality	43.11
Sample_T10B_k141_181069	17742	39	8	0	Low-quality	43.11
Sample_T11A_k141_64901	18312	17	0	3	Low-quality	43.1
Sample_T12A_k141_93760_fragment_2	19439	20	4	0	Low-quality	43.08
Sample_C6B_k141_40434	17035	24	1	10	Low-quality	43.05
Sample_C8B_k141_140600_fragment_2	20283	36	15	2	Low-quality	43.04
Sample_C13C_k141_2378	13995	19	5	0	Low-quality	43.03
Sample_C14C_k141_57880	15128	28	7	0	Low-quality	43.01
Sample_C11B_k141_167994	22991	34	29	0	Low-quality	42.99
Sample_C6B_k141_155198	9748	7	0	0	Low-quality	42.97
Sample_T5B_k141_12010	17851	14	1	0	Low-quality	42.97
Sample_C6A_k141_32766	44032	39	0	24	Low-quality	42.96
Sample_T12A_k141_261811	80231	63	1	37	Low-quality	42.95
Sample_T14C_k141_84507	36072	50	1	13	Low-quality	42.95
Sample_T15C_k141_49271	17836	14	1	1	Low-quality	42.93
Sample_C10C_k141_66331	20472	25	13	0	Low-quality	42.92
Sample_C12A_k141_127175	7975	12	4	0	Low-quality	42.92
Sample_T18C_k141_35663	25938	43	6	1	Low-quality	42.89
Sample_T17C_k141_19655	511205	431	1	288	Low-quality	42.85
Sample_C1C_k141_68676	27756	25	0	13	Low-quality	42.84
Sample_T10A_k141_145907	16734	30	11	0	Low-quality	42.84
Sample_T8C_k141_34938	6293	5	0	0	Low-quality	42.79
Sample_C5A_k141_2405	23856	28	12	0	Low-quality	42.76
Sample_T16B_k141_60503	82218	94	5	10	Low-quality	42.76
Sample_T4B_k141_260357	13641	16	11	0	Low-quality	42.73
Sample_C5A_k141_69929	42170	35	8	0	Low-quality	42.72
Sample_T8C_k141_161354	5464	9	0	0	Low-quality	42.72
Sample_T10A_k141_132346	18161	26	19	0	Low-quality	42.7
Sample_C11C_k141_72457	14299	20	10	0	Low-quality	42.69
Sample_C7A_k141_16991	17825	16	0	11	Low-quality	42.67
Sample_T13C_k141_46547	17537	23	5	1	Low-quality	42.64
Sample_T8B_k141_200612_fragment_1	18200	37	6	1	Low-quality	42.64
Sample_T8C_k141_217572	16482	18	12	0	Low-quality	42.6
Sample_C6A_k141_151424	78501	67	1	59	Low-quality	42.58
Sample_T7C_k141_86800	20742	20	3	2	Low-quality	42.54
Sample_C9B_k141_109885	67072	121	14	1	Low-quality	42.52
Sample_T4B_k141_166366	78220	76	3	8	Low-quality	42.52
Sample_C4B_k141_124826	72345	81	0	38	Low-quality	42.5
Sample_T8B_k141_231132_fragment_1	22824	29	2	4	Low-quality	42.5

Sample_T8C_k141_209516	25152	34	10	1	Low-quality	42.48
Sample_C10A_k141_52568	27197	26	1	10	Low-quality	42.47
Sample_T17C_k141_37651	19345	22	5	3	Low-quality	42.46
Sample_T12C_k141_177286	29568	48	23	1	Low-quality	42.45
Sample_C13B_k141_56229	33602	41	2	10	Low-quality	42.4
Sample_T14B_k141_161899	16443	31	6	0	Low-quality	42.39
Sample_T14B_k141_172598	15760	19	12	1	Low-quality	42.37
Sample_C4B_k141_238453	23605	20	1	4	Low-quality	42.33
Sample_T6C_k141_229919	17728	14	0	9	Low-quality	42.31
Sample_C3A_k141_92113_fragment_1	32623	30	14	3	Low-quality	42.31
Sample_T10A_k141_90864	16619	22	9	0	Low-quality	42.31
Sample_T3A_k141_74155_fragment_1	17014	23	8	1	Low-quality	42.31
Sample_C11C_k141_161297	16350	19	10	0	Low-quality	42.27
Sample_C6B_k141_161734	20999	29	13	0	Low-quality	42.27
Sample_C8C_k141_128118_fragment_2	29587	44	10	5	Low-quality	42.22
Sample_C9A_k141_120073	17817	17	0	2	Low-quality	42.21
Sample_C2C_k141_226962_fragment_1	29400	44	2	2	Low-quality	42.21
Sample_T12C_k141_108451	18205	22	2	0	Low-quality	42.17
Sample_T12C_k141_6645	63242	51	0	8	Low-quality	42.15
Sample_T13B_k141_178641	14686	20	15	0	Low-quality	42.07
Sample_C12A_k141_28786	13241	22	1	3	Low-quality	42.03
Sample_C2B_k141_203415	18915	28	11	0	Low-quality	42.03
Sample_T12B_k141_57806	48039	61	12	16	Low-quality	42.01
Sample_T2A_k141_90098_fragment_1	26445	39	3	0	Low-quality	42.01
Sample_T2A_k141_45038	16643	16	3	0	Low-quality	42.01
Sample_T16B_k141_54825_fragment_1	25107	30	8	3	Low-quality	41.95
Sample_T6C_k141_162184	17498	34	7	0	Low-quality	41.94
Sample_C3A_k141_159319	17571	37	3	0	Low-quality	41.89
Sample_C1B_k141_207201	5672	7	3	0	Low-quality	41.86
Sample_C8B_k141_146024	29179	38	6	0	Low-quality	41.83
Sample_C1B_k141_9025	32560	32	0	6	Low-quality	41.82
Sample_C1B_k141_55826	23323	28	0	5	Low-quality	41.82
Sample_T2C_k141_131364	35659	37	0	23	Low-quality	41.81
Sample_C1C_k141_18500	14959	27	3	0	Low-quality	41.79
Sample_T10A_k141_121448	37889	45	5	7	Low-quality	41.78
Sample_T11A_k141_110159	25250	26	4	7	Low-quality	41.74
Sample_T1A_k141_44351	40533	55	0	3	Low-quality	41.68
Sample_C7B_k141_51192	30051	47	7	3	Low-quality	41.65
Sample_T7A_k141_16839	14674	24	4	1	Low-quality	41.59

Sample_T5C_k141_20545	32030	32	0	9	Low-quality	41.58
Sample_C8B_k141_177275	24750	40	13	1	Low-quality	41.58
Sample_T7B_k141_63656	18761	25	4	2	Low-quality	41.58
Sample_C10C_k141_16600	21154	20	5	2	Low-quality	41.55
Sample_C3C_k141_182787	122123	103	1	61	Low-quality	41.54
Sample_T5B_k141_69051	61733	70	1	62	Low-quality	41.5
Sample_C6B_k141_77233	13161	17	5	0	Low-quality	41.5
Sample_T16C_k141_58777	24840	29	0	9	Low-quality	41.49
Sample_C9B_k141_104160_fragment_1	26310	32	7	1	Low-quality	41.41
Sample_C7A_k141_58258	27356	26	1	14	Low-quality	41.39
Sample_C8A_k141_70335	35817	19	1	14	Low-quality	41.39
Sample_C12A_k141_207263_fragment_1	19628	36	5	2	Low-quality	41.36
Sample_C11A_k141_101479	24902	41	12	2	Low-quality	41.33
Sample_T1B_k141_4612	15900	23	9	0	Low-quality	41.31
Sample_T8A_k141_245560	17600	30	4	0	Low-quality	41.31
Sample_C10A_k141_11764	41077	31	0	24	Low-quality	41.3
Sample_C4C_k141_58556_fragment_1	25486	33	8	1	Low-quality	41.3
Sample_T5C_k141_174034_fragment_1	45612	57	4	6	Low-quality	41.3
Sample_T6B_k141_189886	7582	8	1	1	Low-quality	41.29
Sample_C4A_k141_14958	19641	28	10	1	Low-quality	41.28
Sample_C2A_k141_46515	77092	78	1	49	Low-quality	41.27
Sample_C4C_k141_84112_fragment_1	19609	21	8	1	Low-quality	41.27
Sample_T4C_k141_46440_fragment_1	19036	18	3	5	Low-quality	41.23
Sample_T16A_k141_39375_fragment_1	18936	26	19	0	Low-quality	41.22
Sample_T5C_k141_35148	15404	20	12	0	Low-quality	41.22
Sample_C5C_k141_62940_fragment_1	17119	23	2	1	Low-quality	41.21
Sample_T12A_k141_90032_fragment_1	24280	39	10	0	Low-quality	41.21
Sample_C11B_k141_185401_fragment_1	62282	66	16	1	Low-quality	41.15
Sample_C14C_k141_117766	17468	17	0	3	Low-quality	41.09
Sample_C14A_k141_81163	21963	26	14	2	Low-quality	41.09
Sample_C5A_k141_49403	16604	31	7	0	Low-quality	41.07
Sample_T5B_k141_11880	15339	20	9	0	Low-quality	41.06
Sample_C5C_k141_1606	11777	18	4	0	Low-quality	41.05
Sample_C8C_k141_73937	22891	12	0	3	Low-quality	41.05
Sample_C9C_k141_13236	22600	41	6	1	Low-quality	41
Sample_T3A_k141_28910_fragment_1	16012	30	3	0	Low-quality	41
Sample_T8C_k141_8893	50176	44	2	12	Low-quality	40.99
Sample_T17B_k141_27085	24532	28	0	5	Low-quality	40.98
Sample_C3C_k141_113664	51286	108	11	4	Low-quality	40.98

Sample_T4B_k141_270839	7295	11	1	0	Low-quality	40.97
Sample_T13B_k141_23240	15254	21	13	0	Low-quality	40.92
Sample_T16A_k141_67157	73298	65	1	34	Low-quality	40.9
Sample_T5C_k141_184276	80219	76	2	40	Low-quality	40.89
Sample_C5A_k141_19970	18246	18	4	1	Low-quality	40.88
Sample_C1C_k141_119215	25494	34	1	7	Low-quality	40.86
Sample_C2B_k141_254784	16921	27	6	1	Low-quality	40.82
Sample_C12A_k141_71387	73766	80	1	37	Low-quality	40.8
Sample_T14B_k141_76656	114641	97	1	69	Low-quality	40.79
Sample_C11A_k141_42917	14443	29	7	0	Low-quality	40.79
Sample_C1C_k141_171377	31243	27	1	8	Low-quality	40.77
Sample_T8C_k141_109137	15471	18	4	0	Low-quality	40.77
Sample_C4C_k141_169393	19440	23	12	0	Low-quality	40.72
Sample_T14A_k141_74680	22986	39	4	1	Low-quality	40.69
Sample_T7A_k141_142381	15427	26	6	0	Low-quality	40.69
Sample_T16C_k141_49676	37384	40	0	25	Low-quality	40.66
Sample_T11B_k141_7697	30352	23	0	12	Low-quality	40.61
Sample_C4B_k141_132069	100584	119	14	3	Low-quality	40.6
Sample_C9B_k141_93982_fragment_1	18733	19	3	4	Low-quality	40.58
Sample_T18C_k141_113790	22855	27	3	2	Low-quality	40.53
Sample_T10A_k141_125493	13086	15	8	0	Low-quality	40.49
Sample_T17B_k141_15196	5395	9	0	3	Low-quality	40.48
Sample_T5C_k141_103238	33818	62	10	5	Low-quality	40.48
Sample_C5A_k141_155885_fragment_2	24192	41	7	0	Low-quality	40.47
Sample_T11B_k141_50849	17373	22	2	1	Low-quality	40.46
Sample_C2C_k141_292382	55259	56	1	30	Low-quality	40.45
Sample_C1A_k141_224061	56498	62	1	21	Low-quality	40.44
Sample_T5A_k141_36494	15257	24	5	3	Low-quality	40.44
Sample_T4C_k141_13891	18122	24	19	0	Low-quality	40.41
Sample_T13B_k141_25208_fragment_2	24389	34	2	2	Low-quality	40.4
Sample_C9A_k141_44286	33918	32	1	8	Low-quality	40.39
Sample_T1B_k141_38960	221177	191	1	108	Low-quality	40.39
Sample_C2A_k141_302428	16414	26	10	0	Low-quality	40.39
Sample_C7B_k141_83985	65292	71	1	41	Low-quality	40.35
Sample_T5B_k141_51358	24159	29	0	7	Low-quality	40.35
Sample_C12C_k141_100858	18997	30	10	0	Low-quality	40.35
Sample_T17B_k141_28945	29901	45	5	4	Low-quality	40.34
Sample_T13C_k141_116685	142335	119	1	80	Low-quality	40.32
Sample_C2A_k141_297886	7448	5	2	0	Low-quality	40.31

Sample_C1C_k141_21600	64184	68	1	38	Low-quality	40.26
Sample_C3A_k141_107847_fragment_2	25065	41	11	2	Low-quality	40.26
Sample_C7B_k141_163882	101068	87	1	53	Low-quality	40.25
Sample_T17B_k141_36244	25761	25	1	12	Low-quality	40.23
Sample_C2B_k141_86031	12025	15	3	0	Low-quality	40.22
Sample_C7A_k141_1379	31457	27	0	8	Low-quality	40.19
Sample_T7A_k141_63739	14569	19	9	0	Low-quality	40.19
Sample_C10B_k141_123161	22462	24	1	5	Low-quality	40.18
Sample_T12C_k141_92800	34758	22	1	8	Low-quality	40.17
Sample_T13A_k141_91767	18128	29	14	2	Low-quality	40.17
Sample_T18B_k141_76529	21453	24	15	1	Low-quality	40.14
Sample_C6A_k141_96622	15107	23	1	2	Low-quality	40.11
Sample_T2C_k141_76715_fragment_1	28148	10	2	1	Low-quality	40.1
Sample_T13B_k141_79049	19595	27	11	1	Low-quality	40.07
Sample_C14B_k141_49498	75137	132	18	2	Low-quality	40.06
Sample_T12C_k141_80999	23080	34	10	1	Low-quality	40.06
Sample_C5A_k141_281655	67756	70	1	10	Low-quality	40.05
Sample_C3A_k141_27120_fragment_1	16673	16	3	2	Low-quality	40.04
Sample_C10A_k141_311857	99335	87	1	60	Low-quality	40.03
Sample_T4B_k141_140795	70093	66	1	35	Low-quality	40.03
Sample_T7C_k141_37388	35921	34	0	30	Low-quality	40.01
Sample_T10A_k141_119156	14668	18	12	0	Low-quality	40.01
Sample_C2B_k141_285309	15160	32	2	1	Low-quality	40
Sample_T17B_k141_2651	16277	17	15	0	Low-quality	39.99
Sample_T12A_k141_314063	11953	16	3	0	Low-quality	39.98
Sample_C11B_k141_100987	15477	22	6	0	Low-quality	39.91
Sample_C1C_k141_157185_fragment_1	18250	20	5	2	Low-quality	39.9
Sample_C2C_k141_298055	25538	23	1	10	Low-quality	39.88
Sample_C11B_k141_207305_fragment_1	26035	58	10	0	Low-quality	39.88
Sample_T6C_k141_223552	21304	34	14	0	Low-quality	39.88
Sample_C11B_k141_163125	6170	5	1	0	Low-quality	39.87
Sample_C2A_k141_27840	22312	27	0	8	Low-quality	39.85
Sample_C10A_k141_127626	14324	19	13	0	Low-quality	39.84
Sample_T16C_k141_1247	23841	25	0	2	Low-quality	39.82
Sample_C5A_k141_30164_fragment_1	15286	19	7	1	Low-quality	39.82
Sample_C9B_k141_74651_fragment_1	60990	99	15	3	Low-quality	39.82
Sample_T6A_k141_25427	13321	24	3	0	Low-quality	39.75
Sample_C5A_k141_79821	14832	26	7	1	Low-quality	39.73
Sample_C12B_k141_168337	14157	24	14	0	Low-quality	39.72

Sample_C2B_k141_63743	9212	11	5	0	Low-quality	39.71
Sample_T13B_k141_89658	30406	20	0	9	Low-quality	39.69
Sample_T17B_k141_29005	23274	38	10	1	Low-quality	39.69
Sample_C3A_k141_199751	18670	21	1	0	Low-quality	39.66
Sample_T7A_k141_155640	17677	13	0	2	Low-quality	39.66
Sample_T13C_k141_101243	21849	39	6	0	Low-quality	39.64
Sample_C2A_k141_67414	37011	58	2	7	Low-quality	39.62
Sample_T17B_k141_6064	18568	32	20	2	Low-quality	39.61
Sample_T4B_k141_95140	43122	55	5	1	Low-quality	39.61
Sample_C1C_k141_104536	98260	97	1	64	Low-quality	39.6
Sample_C1A_k141_3399	22654	30	3	2	Low-quality	39.59
Sample_T6B_k141_49493	68405	102	14	0	Low-quality	39.59
Sample_T17A_k141_38992	18960	28	19	1	Low-quality	39.56
Sample_T16B_k141_96863	72886	68	1	47	Low-quality	39.53
Sample_C1B_k141_22617	16801	20	1	5	Low-quality	39.52
Sample_C12A_k141_212964	15634	20	1	6	Low-quality	39.51
Sample_C4A_k141_135705	14458	21	7	1	Low-quality	39.51
Sample_T16B_k141_74463	20601	23	8	3	Low-quality	39.51
Sample_T7B_k141_125838_fragment_1	20071	21	0	3	Low-quality	39.48
Sample_C9A_k141_18192	30933	29	0	8	Low-quality	39.47
Sample_C7B_k141_59074	66962	58	1	34	Low-quality	39.46
Sample_C4C_k141_241985	5787	13	1	0	Low-quality	39.46
Sample_C12A_k141_121688	27065	31	1	6	Low-quality	39.44
Sample_C8C_k141_173625_fragment_1	16102	21	10	0	Low-quality	39.39
Sample_T1A_k141_11838	16522	21	11	0	Low-quality	39.37
Sample_C3A_k141_238000	18190	23	8	0	Low-quality	39.35
Sample_T10A_k141_7247	6124	6	1	4	Low-quality	39.34
Sample_C10C_k141_210729	77625	69	1	53	Low-quality	39.3
Sample_T11C_k141_96540	15401	11	3	0	Low-quality	39.3
Sample_C8B_k141_133461	25161	27	1	16	Low-quality	39.29
Sample_C12C_k141_102267	99672	99	1	80	Low-quality	39.27
Sample_C13A_k141_48933	18854	31	7	0	Low-quality	39.27
Sample_C9A_k141_133896_fragment_1	19007	37	9	3	Low-quality	39.27
Sample_C14C_k141_58621	95283	91	2	14	Low-quality	39.26
Sample_T17B_k141_7569	15618	19	11	0	Low-quality	39.21
Sample_C4C_k141_19285	21096	35	5	1	Low-quality	39.19
Sample_C9C_k141_48061	11994	12	2	0	Low-quality	39.18
Sample_C6A_k141_214012	15160	26	13	0	Low-quality	39.17
Sample_C9C_k141_78780	11991	14	2	0	Low-quality	39.17

Sample_T11C_k141_54873	19725	31	9	2	Low-quality	39.17
Sample_T4C_k141_76886	23584	38	19	2	Low-quality	39.16
Sample_T13B_k141_119060	13017	11	0	9	Low-quality	39.15
Sample_T16B_k141_31947	56644	51	1	34	Low-quality	39.15
Sample_T5A_k141_8686	43622	40	3	6	Low-quality	39.15
Sample_T11B_k141_89771	15787	31	4	0	Low-quality	39.14
Sample_T3A_k141_125816_fragment_1	23856	50	8	1	Low-quality	39.13
Sample_C2B_k141_38748	35282	63	7	1	Low-quality	39.12
Sample_C13C_k141_27009_fragment_1	15160	26	3	0	Low-quality	39.11
Sample_T11B_k141_91478	21105	23	15	1	Low-quality	39.11
Sample_T12B_k141_174249	10399	20	1	1	Low-quality	39.1
Sample_T12C_k141_142527_fragment_1	21551	34	6	0	Low-quality	39.1
Sample_C10C_k141_65761	102394	93	1	54	Low-quality	39.07
Sample_C8B_k141_166967	30872	40	12	0	Low-quality	39.07
Sample_T7A_k141_15157	11680	12	3	0	Low-quality	39.07
Sample_C10C_k141_225722	15788	21	14	0	Low-quality	39.06
Sample_T17A_k141_54478	17688	23	9	0	Low-quality	39.06
Sample_T4B_k141_347516	16268	17	3	0	Low-quality	39.06
Sample_T18C_k141_53344_fragment_1	28561	34	6	3	Low-quality	39.05
Sample_T3C_k141_10268	5201	8	0	1	Low-quality	39.03
Sample_T8B_k141_152901_fragment_3	17547	26	0	1	Low-quality	39.02
Sample_T10B_k141_133956	23233	26	0	5	Low-quality	39.01
Sample_T11A_k141_20877	14139	20	6	0	Low-quality	38.95
Sample_C13A_k141_78598	16303	19	9	0	Low-quality	38.94
Sample_C1C_k141_32951	18404	22	12	1	Low-quality	38.94
Sample_T2A_k141_6783	55537	56	2	6	Low-quality	38.9
Sample_C2A_k141_130825	21268	28	0	7	Low-quality	38.89
Sample_C7B_k141_183222	25774	29	8	6	Low-quality	38.87
Sample_C3A_k141_245477	16333	15	1	6	Low-quality	38.86
Sample_C4C_k141_41871	5723	9	2	0	Low-quality	38.86
Sample_T6B_k141_147015	39417	76	6	0	Low-quality	38.86
Sample_T10C_k141_18783	90210	74	1	41	Low-quality	38.84
Sample_C10A_k141_224163	69560	83	1	28	Low-quality	38.82
Sample_T7C_k141_12882	23179	27	9	3	Low-quality	38.8
Sample_C5B_k141_19427	25622	23	1	7	Low-quality	38.77
Sample_T14A_k141_80926	131638	127	1	92	Low-quality	38.77
Sample_C12C_k141_65494	19023	19	8	2	Low-quality	38.76
Sample_C6C_k141_172633	14240	17	11	0	Low-quality	38.75
Sample_C14C_k141_100359	24315	33	4	1	Low-quality	38.73

Sample_C7A_k141_135020	15195	26	5	0	Low-quality	38.73
Sample_C7B_k141_158280	16104	19	2	3	Low-quality	38.72
Sample_T3A_k141_14206	24971	29	12	2	Low-quality	38.72
Sample_C3C_k141_223344	54905	52	8	24	Low-quality	38.69
Sample_C11B_k141_58422	15829	19	13	0	Low-quality	38.69
Sample_T7B_k141_60904	21387	40	9	0	Low-quality	38.69
Sample_T8B_k141_15070_fragment_1	35486	39	2	4	Low-quality	38.66
Sample_T17C_k141_11205	5149	6	0	2	Low-quality	38.64
Sample_C13B_k141_86774	17716	23	1	0	Low-quality	38.64
Sample_C5A_k141_52756	24324	24	5	1	Low-quality	38.6
Sample_T2B_k141_259335	25097	34	6	1	Low-quality	38.6
Sample_T5A_k141_35551	31187	20	0	9	Low-quality	38.59
Sample_C11C_k141_57605	23907	25	10	2	Low-quality	38.58
Sample_C4B_k141_250763	15976	12	7	1	Low-quality	38.57
Sample_T14A_k141_58586	16059	17	1	3	Low-quality	38.57
Sample_T15A_k141_232	16477	14	4	1	Low-quality	38.54
Sample_C2A_k141_70795	17893	17	0	15	Low-quality	38.52
Sample_T15A_k141_160025_fragment_1	69037	80	17	1	Low-quality	38.52
Sample_C9B_k141_57351	17406	22	20	0	Low-quality	38.5
Sample_T8B_k141_166032	16577	36	3	0	Low-quality	38.5
Sample_T4B_k141_329100	75557	68	2	28	Low-quality	38.49
Sample_T5A_k141_13501	28347	28	7	0	Low-quality	38.49
Sample_C9B_k141_95611	29496	19	0	8	Low-quality	38.44
Sample_T14A_k141_114509	17265	33	12	0	Low-quality	38.43
Sample_C6A_k141_89245_fragment_1	15698	22	10	0	Low-quality	38.42
Sample_T10A_k141_171667	29167	23	0	6	Low-quality	38.39
Sample_C3C_k141_156543	100214	78	1	53	Low-quality	38.38
Sample_T14B_k141_23012_fragment_1	15174	26	7	0	Low-quality	38.37
Sample_T8A_k141_188691	17817	15	0	12	Low-quality	38.36
Sample_T14A_k141_213792	68755	44	3	17	Low-quality	38.33
Sample_T16B_k141_112451	8682	9	0	2	Low-quality	38.28
Sample_C14A_k141_39791_fragment_1	14913	25	8	1	Low-quality	38.27
Sample_C3A_k141_129322	22292	17	1	6	Low-quality	38.26
Sample_C5A_k141_277503	19337	22	1	6	Low-quality	38.22
Sample_T14B_k141_668	21292	21	0	6	Low-quality	38.18
Sample_T3A_k141_100259	5477	10	1	0	Low-quality	38.18
Sample_T2A_k141_49665	18033	19	5	0	Low-quality	38.18
Sample_T4C_k141_76093	22020	38	6	0	Low-quality	38.17
Sample_C9C_k141_23896	17272	37	5	1	Low-quality	38.16

Sample_C10B_k141_104876	55679	50	1	26	Low-quality	38.13
Sample_C3B_k141_5702	36524	36	0	19	Low-quality	38.12
Sample_C8A_k141_79727	23335	32	3	0	Low-quality	38.11
Sample_C3A_k141_231792	22446	22	14	0	Low-quality	38.1
Sample_C3A_k141_198825	17112	25	1	1	Low-quality	38.09
Sample_T14A_k141_239085	30022	29	1	13	Low-quality	38.04
Sample_C13C_k141_31418	13840	16	11	0	Low-quality	38.04
Sample_T8B_k141_114763	41706	40	0	25	Low-quality	38.03
Sample_T13C_k141_79182	17670	16	0	3	Low-quality	38.01
Sample_T4B_k141_43377	58687	59	1	33	Low-quality	37.98
Sample_C2A_k141_100539	52700	51	1	32	Low-quality	37.97
Sample_T11A_k141_52710	66847	50	1	36	Low-quality	37.95
Sample_T8B_k141_223878	12944	20	14	0	Low-quality	37.95
Sample_T8A_k141_6547	13867	24	2	0	Low-quality	37.94
Sample_T13B_k141_44333	16981	19	0	15	Low-quality	37.93
Sample_T5C_k141_119649_fragment_1	20364	29	3	2	Low-quality	37.91
Sample_C12A_k141_22370	25047	47	4	0	Low-quality	37.9
Sample_C12B_k141_71046	15470	21	1	1	Low-quality	37.89
Sample_T18A_k141_121200	29195	30	14	4	Low-quality	37.88
Sample_C6B_k141_169961	15414	23	6	0	Low-quality	37.85
Sample_C14C_k141_28691	26897	30	1	6	Low-quality	37.84
Sample_C12A_k141_70750	7796	10	0	0	Low-quality	37.84
Sample_T3B_k141_154496	16010	17	1	0	Low-quality	37.82
Sample_C3A_k141_154747	21081	21	0	7	Low-quality	37.8
Sample_C12C_k141_79923	25506	18	1	2	Low-quality	37.77
Sample_T4B_k141_41258	91732	102	1	42	Low-quality	37.76
Sample_C6C_k141_77426	56335	56	1	35	Low-quality	37.75
Sample_T11B_k141_158050	16894	26	8	0	Low-quality	37.75
Sample_T14A_k141_220140_fragment_1	10809	8	0	1	Low-quality	37.74
Sample_C7C_k141_51978_fragment_2	13822	27	5	0	Low-quality	37.73
Sample_C13A_k141_24723	21273	23	0	4	Low-quality	37.71
Sample_C7C_k141_87988	21879	34	7	2	Low-quality	37.69
Sample_T10A_k141_169638	15275	20	3	0	Low-quality	37.66
Sample_T8A_k141_189627	26254	18	1	4	Low-quality	37.64
Sample_T11B_k141_91096	14506	11	4	0	Low-quality	37.62
Sample_C14A_k141_145613	22480	26	1	5	Low-quality	37.55
Sample_T7C_k141_120879	20576	27	11	0	Low-quality	37.55
Sample_C10A_k141_23230	8857	10	5	0	Low-quality	37.54
Sample_T12A_k141_104575	26138	22	1	14	Low-quality	37.53

Sample_C8B_k141_102779_fragment_1	16718	23	5	0	Low-quality	37.53
Sample_C4A_k141_106712	17441	16	0	2	Low-quality	37.52
Sample_T10A_k141_43049	14648	24	12	0	Low-quality	37.52
Sample_C10A_k141_312059	15763	19	0	11	Low-quality	37.51
Sample_C12C_k141_206540	18033	20	9	0	Low-quality	37.51
Sample_T12B_k141_23739	16898	31	1	1	Low-quality	37.51
Sample_C12B_k141_29784	16033	13	2	0	Low-quality	37.49
Sample_T18B_k141_70727	14386	18	2	1	Low-quality	37.48
Sample_T15A_k141_81920	18016	28	2	1	Low-quality	37.48
Sample_T11A_k141_80869	17863	30	9	0	Low-quality	37.47
Sample_T4B_k141_170430	14059	23	8	0	Low-quality	37.46
Sample_T13B_k141_24811	14981	23	12	0	Low-quality	37.44
Sample_T11A_k141_88598	167752	153	4	67	Low-quality	37.42
Sample_C3C_k141_174511	14887	23	7	0	Low-quality	37.41
Sample_C6A_k141_118356	30808	20	0	19	Low-quality	37.4
Sample_T10C_k141_52211	33000	68	6	1	Low-quality	37.4
Sample_T13A_k141_75458	17514	21	8	0	Low-quality	37.4
Sample_C5B_k141_62212_fragment_1	30747	31	0	4	Low-quality	37.37
Sample_T6B_k141_78001	17318	24	4	0	Low-quality	37.37
Sample_C4C_k141_7083	24319	17	3	0	Low-quality	37.32
Sample_T17A_k141_17686	31336	33	1	10	Low-quality	37.31
Sample_T1B_k141_2548	33573	29	0	15	Low-quality	37.3
Sample_C11B_k141_55172	18649	20	1	2	Low-quality	37.3
Sample_T4A_k141_31341	52614	46	2	26	Low-quality	37.27
Sample_T11C_k141_78665	92445	87	1	56	Low-quality	37.26
Sample_C3B_k141_21803	29799	37	7	1	Low-quality	37.25
Sample_T13C_k141_110612	24609	31	1	8	Low-quality	37.24
Sample_T17B_k141_20784	17443	23	17	0	Low-quality	37.2
Sample_C8A_k141_178716	20722	19	1	6	Low-quality	37.16
Sample_T17B_k141_25130	14735	14	10	0	Low-quality	37.15
Sample_T4C_k141_11543	65538	82	2	5	Low-quality	37.15
Sample_C1C_k141_137734	16412	22	14	0	Low-quality	37.14
Sample_C3C_k141_90221	30981	33	2	2	Low-quality	37.14
Sample_C2A_k141_8693	23270	20	1	3	Low-quality	37.11
Sample_C9A_k141_71559_fragment_1	7609	8	1	1	Low-quality	37.1
Sample_T16C_k141_113802	19059	25	3	1	Low-quality	37.1
Sample_C2B_k141_5927	21300	25	1	6	Low-quality	37.08
Sample_C10A_k141_68286	13331	19	4	3	Low-quality	37.07
Sample_C8B_k141_53286	14244	22	4	0	Low-quality	37.06

Sample_C3B_k141_139744	68232	62	1	36	Low-quality	37.03
Sample_T18A_k141_107052	41643	42	1	9	Low-quality	36.97
Sample_T6C_k141_57839	15540	14	0	3	Low-quality	36.97
Sample_C4B_k141_85784	15275	15	4	1	Low-quality	36.97
Sample_C7B_k141_124045	13578	21	9	0	Low-quality	36.95
Sample_T1C_k141_49518	11768	14	7	0	Low-quality	36.94
Sample_T6A_k141_174002	18267	18	5	0	Low-quality	36.91
Sample_C1C_k141_25422_fragment_1	13814	27	4	1	Low-quality	36.9
Sample_T8B_k141_52237	20538	27	0	16	Low-quality	36.89
Sample_C6C_k141_74644	24083	37	4	1	Low-quality	36.88
Sample_C8B_k141_149309	12570	17	6	0	Low-quality	36.88
Sample_T4B_k141_72828	67972	60	1	49	Low-quality	36.87
Sample_C7A_k141_130376	18449	44	4	0	Low-quality	36.87
Sample_C13A_k141_32881	15304	17	9	0	Low-quality	36.85
Sample_T2C_k141_234848	17126	14	0	2	Low-quality	36.84
Sample_C13C_k141_13636	15794	27	7	1	Low-quality	36.84
Sample_C9A_k141_132518_fragment_1	15403	12	6	0	Low-quality	36.83
Sample_T3B_k141_125661	54482	54	13	13	Low-quality	36.81
Sample_T10A_k141_102993	18383	41	4	0	Low-quality	36.8
Sample_T4B_k141_250731_fragment_5	20753	21	4	1	Low-quality	36.8
Sample_T10A_k141_98510	13254	24	9	0	Low-quality	36.79
Sample_C2C_k141_272288	15090	18	10	0	Low-quality	36.78
Sample_T8C_k141_92796_fragment_1	15558	24	9	0	Low-quality	36.78
Sample_C3C_k141_22955	15453	29	0	29	Low-quality	36.77
Sample_C4B_k141_68591	19024	24	10	0	Low-quality	36.76
Sample_C2B_k141_227033	15075	29	10	0	Low-quality	36.75
Sample_C5A_k141_153697	38318	40	7	1	Low-quality	36.75
Sample_C11A_k141_62779	20479	15	0	3	Low-quality	36.72
Sample_C6C_k141_192887	17399	17	0	8	Low-quality	36.72
Sample_C13A_k141_15427	14470	28	6	0	Low-quality	36.71
Sample_C1C_k141_82158	14538	21	7	0	Low-quality	36.71
Sample_T2A_k141_157385	28117	31	0	4	Low-quality	36.69
Sample_C13A_k141_79059	30984	50	19	0	Low-quality	36.69
Sample_T2C_k141_113894	15527	25	5	0	Low-quality	36.68
Sample_C5A_k141_273448_fragment_1	18666	30	9	2	Low-quality	36.67
Sample_C7B_k141_62452	38322	45	2	23	Low-quality	36.66
Sample_T4B_k141_194967	77445	57	2	29	Low-quality	36.66
Sample_C12C_k141_145634	19382	20	3	3	Low-quality	36.66
Sample_C4C_k141_89686	27293	20	1	8	Low-quality	36.64

Sample_T18A_k141_127534	15583	11	0	3	Low-quality	36.64
Sample_C2A_k141_154277	7056	7	5	0	Low-quality	36.64
Sample_C5B_k141_41699	17381	23	2	1	Low-quality	36.63
Sample_T15A_k141_158632	28221	20	2	2	Low-quality	36.61
Sample_T6B_k141_55033	13375	27	5	1	Low-quality	36.57
Sample_T16B_k141_54858	16680	19	10	0	Low-quality	36.55
Sample_T6A_k141_195738	18175	33	8	0	Low-quality	36.54
Sample_T15C_k141_55722_fragment_1	28192	51	5	1	Low-quality	36.53
Sample_T8A_k141_18190	28153	15	0	6	Low-quality	36.5
Sample_T18B_k141_40010	38792	44	19	4	Low-quality	36.5
Sample_C13A_k141_65900	21136	35	7	3	Low-quality	36.48
Sample_T7C_k141_128143	16443	21	8	0	Low-quality	36.47
Sample_C8A_k141_148293_fragment_1	22858	39	25	2	Low-quality	36.46
Sample_C7B_k141_126436	17559	23	9	0	Low-quality	36.45
Sample_C7B_k141_91465	16466	21	4	0	Low-quality	36.44
Sample_T5B_k141_2626	24395	31	0	7	Low-quality	36.42
Sample_T8A_k141_190027	14660	17	9	0	Low-quality	36.38
Sample_C11B_k141_48374	31519	40	1	16	Low-quality	36.37
Sample_C10A_k141_131970	58643	54	2	27	Low-quality	36.36
Sample_C2B_k141_257186	56710	60	1	25	Low-quality	36.34
Sample_C2A_k141_74850	14260	14	0	3	Low-quality	36.33
Sample_T4B_k141_191036	38464	38	0	23	Low-quality	36.32
Sample_C10C_k141_205206	16462	20	15	0	Low-quality	36.32
Sample_T2C_k141_258653	20244	13	0	4	Low-quality	36.3
Sample_T18B_k141_72293	19565	25	5	1	Low-quality	36.29
Sample_T4A_k141_104381_fragment_2	78772	84	5	8	Low-quality	36.26
Sample_C9B_k141_11908	23948	25	1	6	Low-quality	36.24
Sample_T7B_k141_131668	20404	26	0	4	Low-quality	36.24
Sample_C3A_k141_72460	19417	32	7	1	Low-quality	36.23
Sample_T6A_k141_105891	73852	59	1	14	Low-quality	36.22
Sample_T6A_k141_279285	18254	23	11	0	Low-quality	36.22
Sample_T8A_k141_2123_fragment_1	19718	34	7	0	Low-quality	36.21
Sample_C1B_k141_42358	33485	16	2	0	Low-quality	36.19
Sample_C12A_k141_144542	16797	18	0	16	Low-quality	36.16
Sample_C2B_k141_190772	21130	35	8	0	Low-quality	36.14
Sample_T17B_k141_26743	13398	16	11	0	Low-quality	36.11
Sample_T2B_k141_174280	16656	22	1	8	Low-quality	36.1
Sample_T3A_k141_62631	13459	15	0	6	Low-quality	36.1
Sample_T10A_k141_104938	31768	21	0	7	Low-quality	36.09

Sample_T11A_k141_107419	59981	51	1	36	Low-quality	36.07
Sample_C4C_k141_140960	19695	41	10	1	Low-quality	36.07
Sample_T11A_k141_130329	15339	11	0	3	Low-quality	36.04
Sample_T14B_k141_151856	40329	43	1	7	Low-quality	36.04
Sample_T12C_k141_138250	27396	52	11	1	Low-quality	36.03
Sample_T6A_k141_64411	19197	24	6	2	Low-quality	36.03
Sample_T10B_k141_32334	14149	19	12	0	Low-quality	36
Sample_C11A_k141_93911	7579	9	1	0	Low-quality	35.99
Sample_C10A_k141_210965	13047	9	2	0	Low-quality	35.98
Sample_C4C_k141_224	22201	32	21	0	Low-quality	35.97
Sample_C3A_k141_89321	12010	17	1	2	Low-quality	35.96
Sample_C6C_k141_179130_fragment_1	13216	35	5	0	Low-quality	35.96
Sample_T13B_k141_132373_fragment_1	14628	18	12	0	Low-quality	35.96
Sample_T3A_k141_92891_fragment_4	17872	39	3	0	Low-quality	35.96
Sample_T15C_k141_107961	13140	17	3	0	Low-quality	35.95
Sample_C6C_k141_74965	21043	30	6	0	Low-quality	35.93
Sample_T6C_k141_224402	35440	39	0	12	Low-quality	35.92
Sample_C9C_k141_90437	39781	41	5	2	Low-quality	35.9
Sample_T6A_k141_19086	25326	20	1	6	Low-quality	35.89
Sample_T5B_k141_33841	14131	29	4	1	Low-quality	35.88
Sample_C12C_k141_19361	14854	20	5	0	Low-quality	35.85
Sample_T14B_k141_207693	12631	20	12	0	Low-quality	35.85
Sample_C10C_k141_208782	6228	8	3	0	Low-quality	35.84
Sample_C5C_k141_116744	15195	17	16	0	Low-quality	35.78
Sample_T12B_k141_149734	88004	107	1	11	Low-quality	35.77
Sample_T2A_k141_238410	19808	23	10	3	Low-quality	35.77
Sample_C2B_k141_332115	11449	16	2	0	Low-quality	35.76
Sample_T16C_k141_80032	23611	24	1	10	Low-quality	35.73
Sample_T6A_k141_130132_fragment_1	12691	15	8	0	Low-quality	35.73
Sample_C10A_k141_294757	84898	71	1	43	Low-quality	35.69
Sample_T15B_k141_40481	14368	21	6	0	Low-quality	35.67
Sample_C12A_k141_107221	28134	21	1	6	Low-quality	35.65
Sample_T8B_k141_118026	19842	19	0	5	Low-quality	35.64
Sample_C3A_k141_111466	16611	14	4	0	Low-quality	35.64
Sample_T6C_k141_94163	18084	29	5	0	Low-quality	35.64
Sample_T6B_k141_190149_fragment_1	33799	45	2	2	Low-quality	35.62
Sample_C8C_k141_907	12388	15	5	1	Low-quality	35.6
Sample_C10A_k141_223093	15555	26	6	0	Low-quality	35.59
Sample_C4B_k141_124073	14567	23	14	0	Low-quality	35.58

Sample_C4C_k141_120146	12257	16	9	0	Low-quality	35.58
Sample_T13B_k141_61250	22019	24	4	1	Low-quality	35.58
Sample_T12A_k141_189962	82636	87	17	2	Low-quality	35.56
Sample_C6C_k141_89080	15980	14	4	1	Low-quality	35.55
Sample_T2B_k141_201209	22595	27	0	6	Low-quality	35.55
Sample_C4C_k141_154184	22875	23	2	1	Low-quality	35.55
Sample_T2C_k141_215259	13401	20	6	0	Low-quality	35.55
Sample_C1B_k141_31102	48216	46	2	8	Low-quality	35.5
Sample_C12C_k141_81627	14523	18	2	2	Low-quality	35.49
Sample_C9A_k141_85306	20280	23	2	4	Low-quality	35.49
Sample_C13C_k141_59881_fragment_1	17085	32	11	2	Low-quality	35.47
Sample_C8A_k141_54247	17167	16	2	1	Low-quality	35.47
Sample_T16B_k141_93435	15662	35	6	2	Low-quality	35.47
Sample_T5A_k141_33372	57152	55	2	16	Low-quality	35.44
Sample_T8B_k141_502_fragment_1	22398	31	12	4	Low-quality	35.42
Sample_C14A_k141_47936	21197	26	1	7	Low-quality	35.41
Sample_C7C_k141_24372	18998	29	6	1	Low-quality	35.41
Sample_T10B_k141_35262	19337	21	1	10	Low-quality	35.4
Sample_C3A_k141_156103	14824	12	0	2	Low-quality	35.35
Sample_C7B_k141_180666	13367	18	7	0	Low-quality	35.34
Sample_T17C_k141_32375_fragment_1	15317	26	4	0	Low-quality	35.33
Sample_C2C_k141_288574	27180	27	0	7	Low-quality	35.32
Sample_T1C_k141_5745	26271	43	5	3	Low-quality	35.28
Sample_T13B_k141_77555_fragment_1	24388	33	4	3	Low-quality	35.27
Sample_T7C_k141_103173	28821	22	1	2	Low-quality	35.26
Sample_T7C_k141_96026	21111	24	0	8	Low-quality	35.26
Sample_T8B_k141_7690	83840	67	2	43	Low-quality	35.25
Sample_C1B_k141_82122	14230	12	1	0	Low-quality	35.25
Sample_C11C_k141_96386	21755	40	5	0	Low-quality	35.22
Sample_C3A_k141_84716_fragment_2	25716	22	8	2	Low-quality	35.21
Sample_T15B_k141_93124_fragment_2	28368	27	7	1	Low-quality	35.21
Sample_T15A_k141_149478	27612	30	1	19	Low-quality	35.18
Sample_T8C_k141_210412	27012	23	0	5	Low-quality	35.18
Sample_C6A_k141_94365	13144	19	12	0	Low-quality	35.17
Sample_C3A_k141_243019	14969	29	14	0	Low-quality	35.16
Sample_T7A_k141_227956	5146	11	2	0	Low-quality	35.16
Sample_T8B_k141_197434	5306	6	2	0	Low-quality	35.16
Sample_T16B_k141_143677	39014	28	5	2	Low-quality	35.15
Sample_T16C_k141_63268	16581	26	7	1	Low-quality	35.15

Sample_C4A_k141_80803	119314	92	1	57	Low-quality	35.14
Sample_C12B_k141_143327	15159	25	5	0	Low-quality	35.08
Sample_C10A_k141_87103	15534	16	0	5	Low-quality	35.06
Sample_C2A_k141_209176	16654	24	3	0	Low-quality	35.06
Sample_C2A_k141_73019	13678	6	2	0	Low-quality	35.06
Sample_C10A_k141_210305	21603	43	12	0	Low-quality	35.05
Sample_T6A_k141_252670	5459	9	1	0	Low-quality	35.03
Sample_T6C_k141_104387_fragment_3	32508	39	10	6	Low-quality	35.01
Sample_C6A_k141_41586	20951	29	9	4	Low-quality	35
Sample_T7C_k141_156312	15837	19	4	3	Low-quality	34.99
Sample_C12A_k141_213002	19845	27	1	7	Low-quality	34.98
Sample_C1C_k141_79400	16868	25	0	3	Low-quality	34.98
Sample_C11A_k141_127732	83174	69	1	56	Low-quality	34.97
Sample_C4B_k141_13806	28364	23	1	5	Low-quality	34.95
Sample_T16C_k141_38705	14565	10	0	2	Low-quality	34.94
Sample_T17A_k141_10236	13470	20	8	0	Low-quality	34.93
Sample_T5C_k141_9207	15803	22	17	0	Low-quality	34.92
Sample_T8A_k141_115932	12760	13	3	0	Low-quality	34.91
Sample_C1B_k141_71604	15729	14	1	0	Low-quality	34.91
Sample_T6C_k141_83822	20334	21	1	6	Low-quality	34.9
Sample_T14A_k141_102133	19086	34	6	1	Low-quality	34.9
Sample_C9C_k141_73649	24614	34	4	4	Low-quality	34.8
Sample_C5B_k141_73753	14616	15	7	0	Low-quality	34.8
Sample_C2B_k141_345278	12832	20	5	0	Low-quality	34.78
Sample_C9A_k141_102964	19613	19	2	1	Low-quality	34.77
Sample_T11A_k141_104955	14749	12	0	2	Low-quality	34.76
Sample_T17B_k141_303	14606	21	0	8	Low-quality	34.75
Sample_T18B_k141_137223	18568	21	11	3	Low-quality	34.74
Sample_T8C_k141_70943	8012	9	5	0	Low-quality	34.74
Sample_C10C_k141_85026	16144	14	0	3	Low-quality	34.73
Sample_C14B_k141_68677	12950	21	1	0	Low-quality	34.71
Sample_T5C_k141_163584	5398	9	0	0	Low-quality	34.71
Sample_T2C_k141_124495	15640	36	5	0	Low-quality	34.7
Sample_T3B_k141_152213	17680	21	11	2	Low-quality	34.7
Sample_T6A_k141_227335	25354	26	1	12	Low-quality	34.68
Sample_T12A_k141_88504	82608	70	10	3	Low-quality	34.68
Sample_C6C_k141_86320	60518	52	0	46	Low-quality	34.67
Sample_T14C_k141_148044	14813	12	0	1	Low-quality	34.66
Sample_T6B_k141_124436	7745	11	2	0	Low-quality	34.66

Sample_T16B_k141_14699	14651	9	4	0	Low-quality	34.65
Sample_C4C_k141_214462	21538	35	9	2	Low-quality	34.63
Sample_T3B_k141_136760	60378	58	2	13	Low-quality	34.63
Sample_T4B_k141_299361	55021	60	1	37	Low-quality	34.63
Sample_T5C_k141_144838_fragment_2	25863	21	7	2	Low-quality	34.62
Sample_T18C_k141_72599	14201	25	0	0	Low-quality	34.61
Sample_T2A_k141_148115	50543	42	0	33	Low-quality	34.6
Sample_C2B_k141_316803	15299	30	4	0	Low-quality	34.6
Sample_T10B_k141_41053	13358	15	11	0	Low-quality	34.6
Sample_T13A_k141_80522	28259	33	1	5	Low-quality	34.58
Sample_T13C_k141_139223	19279	15	1	6	Low-quality	34.57
Sample_C10A_k141_112843	55076	59	1	24	Low-quality	34.55
Sample_C4A_k141_134699	15087	30	2	1	Low-quality	34.55
Sample_T1A_k141_18831	24806	28	1	11	Low-quality	34.5
Sample_T11B_k141_66869	22254	23	2	7	Low-quality	34.49
Sample_T8C_k141_3031	19202	15	0	13	Low-quality	34.49
Sample_C3C_k141_119116	61519	54	1	36	Low-quality	34.46
Sample_C8B_k141_154193	15251	20	9	0	Low-quality	34.46
Sample_C12C_k141_128716	10384	12	6	0	Low-quality	34.45
Sample_C13A_k141_85720	17789	21	0	4	Low-quality	34.45
Sample_C5C_k141_24669	14267	6	3	0	Low-quality	34.45
Sample_T10B_k141_176110	60427	112	18	1	Low-quality	34.45
Sample_T3C_k141_121506	5334	4	0	0	Low-quality	34.44
Sample_T6B_k141_110769	25479	32	5	6	Low-quality	34.43
Sample_C13C_k141_52889_fragment_1	20223	40	8	0	Low-quality	34.41
Sample_C8B_k141_64584_fragment_1	11724	11	1	1	Low-quality	34.39
Sample_C11A_k141_43065	23595	30	1	8	Low-quality	34.38
Sample_T5A_k141_46515	14035	24	6	1	Low-quality	34.37
Sample_C3A_k141_12678	30610	37	1	6	Low-quality	34.36
Sample_C9A_k141_57916	17529	26	0	2	Low-quality	34.33
Sample_C4B_k141_117521	85129	77	11	0	Low-quality	34.31
Sample_C10A_k141_4028	14647	21	6	0	Low-quality	34.29
Sample_T15B_k141_56376	12972	17	6	0	Low-quality	34.29
Sample_T14A_k141_4095	13243	28	1	0	Low-quality	34.26
Sample_T12B_k141_20749_fragment_1	12670	15	4	0	Low-quality	34.24
Sample_T7C_k141_54492	13953	22	18	0	Low-quality	34.24
Sample_T15B_k141_4779	22622	26	1	4	Low-quality	34.23
Sample_C10A_k141_323481	23264	40	2	7	Low-quality	34.2
Sample_C4B_k141_164882	13266	26	2	0	Low-quality	34.2

Sample_C8C_k141_135905	10767	15	2	0	Low-quality	34.2
Sample_T6A_k141_31077	22594	25	1	8	Low-quality	34.19
Sample_T14B_k141_114137	14455	14	1	2	Low-quality	34.17
Sample_T8C_k141_111097	22207	36	3	3	Low-quality	34.15
Sample_T2B_k141_261052	15782	15	0	2	Low-quality	34.12
Sample_C12A_k141_6571	23276	19	0	14	Low-quality	34.11
Sample_C3A_k141_110906	13542	9	0	0	Low-quality	34.11
Sample_C1A_k141_2350	36099	34	1	11	Low-quality	34.09
Sample_T15C_k141_32286	83933	76	1	33	Low-quality	34.09
Sample_T15C_k141_122345	54947	51	2	15	Low-quality	34.07
Sample_C12A_k141_155923	36835	49	0	38	Low-quality	34.06
Sample_T8B_k141_189970	15817	15	0	5	Low-quality	34.02
Sample_T17C_k141_9274_fragment_1	22858	28	11	7	Low-quality	34.02
Sample_T8A_k141_177801	14616	24	10	0	Low-quality	34.02
Sample_C6B_k141_162939	24591	21	0	7	Low-quality	34.01
Sample_T8B_k141_5837	62656	46	1	35	Low-quality	33.98
Sample_C11C_k141_24473	16656	16	0	12	Low-quality	33.96
Sample_T4A_k141_76742	18995	19	3	3	Low-quality	33.96
Sample_C10A_k141_125978	63428	65	1	38	Low-quality	33.95
Sample_T10C_k141_81109	123871	105	1	69	Low-quality	33.94
Sample_T15B_k141_47022	63100	56	1	17	Low-quality	33.92
Sample_C4B_k141_15539	20300	25	4	4	Low-quality	33.92
Sample_T5A_k141_53348_fragment_1	16445	21	9	0	Low-quality	33.91
Sample_C14A_k141_137653	18099	31	14	0	Low-quality	33.9
Sample_T8C_k141_183565	16898	22	2	3	Low-quality	33.89
Sample_T6A_k141_166027	12357	16	5	0	Low-quality	33.88
Sample_T10B_k141_106379	51831	46	2	14	Low-quality	33.86
Sample_C10B_k141_59497	18369	28	6	0	Low-quality	33.84
Sample_T7C_k141_69974	26728	63	6	3	Low-quality	33.84
Sample_T8C_k141_67193	14063	21	5	1	Low-quality	33.83
Sample_T8B_k141_117265	13384	12	3	0	Low-quality	33.82
Sample_T3B_k141_122518	13482	22	3	0	Low-quality	33.79
Sample_C8B_k141_55898	17699	25	9	0	Low-quality	33.78
Sample_C7C_k141_1498	24377	19	2	3	Low-quality	33.77
Sample_C10A_k141_246157	11287	20	7	0	Low-quality	33.76
Sample_C2B_k141_11720	13310	17	8	0	Low-quality	33.76
Sample_T12A_k141_211399	37377	37	1	14	Low-quality	33.75
Sample_T6B_k141_90135	12636	20	10	0	Low-quality	33.74
Sample_C6B_k141_117494	18377	18	2	2	Low-quality	33.72

Sample_C13C_k141_65981	13637	16	12	0	Low-quality	33.71
Sample_T16B_k141_65623_fragment_1	58288	92	18	3	Low-quality	33.71
Sample_C14A_k141_110678	22231	33	14	1	Low-quality	33.68
Sample_C4C_k141_245827	27098	15	0	5	Low-quality	33.67
Sample_C11C_k141_133875	30092	30	4	5	Low-quality	33.66
Sample_C7B_k141_78343	11461	18	10	0	Low-quality	33.66
Sample_C11B_k141_184562	12297	28	4	2	Low-quality	33.65
Sample_C2C_k141_297634	57429	65	2	26	Low-quality	33.64
Sample_T18B_k141_13614	22229	28	1	7	Low-quality	33.64
Sample_C11A_k141_143838_fragment_3	63184	65	10	5	Low-quality	33.64
Sample_T12A_k141_26626	14228	28	3	0	Low-quality	33.63
Sample_C8C_k141_95732	19430	27	3	1	Low-quality	33.62
Sample_C2C_k141_48412	29916	42	1	3	Low-quality	33.58
Sample_T1A_k141_33333	18718	16	0	4	Low-quality	33.57
Sample_T7B_k141_3225	12918	13	0	1	Low-quality	33.57
Sample_T2A_k141_141246	15601	13	1	5	Low-quality	33.56
Sample_C5A_k141_126417	36060	28	0	23	Low-quality	33.55
Sample_T10A_k141_24907	21557	24	0	6	Low-quality	33.54
Sample_C3A_k141_171947	20097	37	9	0	Low-quality	33.54
Sample_T11C_k141_59959	13137	26	1	0	Low-quality	33.52
Sample_C10C_k141_170897	37692	34	1	6	Low-quality	33.51
Sample_C2B_k141_186143	20046	24	0	6	Low-quality	33.48
Sample_C9B_k141_22634	38711	53	7	3	Low-quality	33.48
Sample_T2B_k141_160581	36739	18	1	4	Low-quality	33.46
Sample_T5B_k141_6663	16131	21	11	1	Low-quality	33.45
Sample_C2B_k141_77170	13843	22	0	0	Low-quality	33.45
Sample_C3A_k141_240655	14622	24	2	0	Low-quality	33.44
Sample_T7B_k141_108776	14402	29	8	0	Low-quality	33.43
Sample_T6A_k141_198291	13956	14	0	2	Low-quality	33.39
Sample_C4B_k141_248324	17972	38	5	0	Low-quality	33.37
Sample_T16C_k141_16585	38830	46	1	21	Low-quality	33.37
Sample_T17A_k141_43120	37142	43	1	14	Low-quality	33.37
Sample_C14A_k141_238892	8688	12	3	0	Low-quality	33.36
Sample_T12C_k141_131086	26887	54	9	2	Low-quality	33.36
Sample_C13A_k141_113897	36925	43	2	2	Low-quality	33.32
Sample_T10B_k141_120802_fragment_2	12867	24	4	1	Low-quality	33.32
Sample_C6B_k141_177414	28126	29	5	10	Low-quality	33.31
Sample_T10C_k141_89313	42327	41	2	12	Low-quality	33.31
Sample_T6A_k141_11387	22747	44	11	6	Low-quality	33.29

Sample_T17C_k141_34031	17611	35	2	1	Low-quality	33.27
Sample_T15A_k141_125430	21827	23	4	6	Low-quality	33.26
Sample_T5C_k141_160710	15819	29	3	0	Low-quality	33.25
Sample_T10B_k141_80028	12727	19	4	0	Low-quality	33.24
Sample_C10B_k141_5682	19219	22	8	4	Low-quality	33.23
Sample_T14C_k141_5446	11995	16	11	0	Low-quality	33.23
Sample_T7A_k141_140904	61430	57	2	8	Low-quality	33.2
Sample_C13B_k141_109061_fragment_1	13323	16	9	0	Low-quality	33.2
Sample_T16C_k141_133808_fragment_4	12314	20	4	0	Low-quality	33.2
Sample_T6B_k141_73453	12928	22	12	0	Low-quality	33.19
Sample_T7A_k141_128531	16364	29	3	1	Low-quality	33.19
Sample_T11B_k141_83744	21249	18	1	5	Low-quality	33.18
Sample_C6C_k141_52736	71802	55	1	44	Low-quality	33.17
Sample_T4A_k141_53967_fragment_1	17642	22	2	0	Low-quality	33.15
Sample_T15B_k141_123070	28102	53	2	4	Low-quality	33.13
Sample_T5B_k141_82066	25869	31	1	7	Low-quality	33.13
Sample_T15A_k141_110676	18365	37	7	1	Low-quality	33.12
Sample_T6A_k141_166113	27920	25	1	14	Low-quality	33.11
Sample_C14A_k141_51622	13211	18	9	0	Low-quality	33.11
Sample_T13B_k141_166595	16232	11	0	3	Low-quality	33.09
Sample_T11B_k141_22490_fragment_3	18611	25	10	5	Low-quality	33.09
Sample_C2B_k141_40762	12357	21	8	0	Low-quality	33.08
Sample_T13C_k141_93106	15322	24	4	1	Low-quality	33.07
Sample_C2A_k141_117133_fragment_1	13463	19	6	1	Low-quality	33.06
Sample_T4B_k141_255557	12355	20	5	0	Low-quality	33.06
Sample_C12C_k141_222453	13341	16	12	0	Low-quality	33.05
Sample_C13C_k141_94466_fragment_1	11746	22	10	0	Low-quality	33.04
Sample_T13C_k141_72909	5112	7	1	0	Low-quality	33.04
Sample_T3B_k141_162877	9478	11	4	0	Low-quality	33.04
Sample_T6B_k141_85609	40709	33	1	15	Low-quality	33.02
Sample_C8A_k141_74615_fragment_1	11237	22	0	0	Low-quality	33.02
Sample_T8A_k141_202831	18285	9	0	7	Low-quality	33.01
Sample_T5A_k141_47915_fragment_1	26570	50	11	1	Low-quality	33.01
Sample_C1B_k141_155273	13919	19	8	0	Low-quality	33
Sample_T5C_k141_97195_fragment_2	14198	22	4	0	Low-quality	33
Sample_T12A_k141_75468	60823	63	1	51	Low-quality	32.99
Sample_T16C_k141_77362	14190	27	6	1	Low-quality	32.98
Sample_C5A_k141_196838_fragment_4	12502	14	4	0	Low-quality	32.97
Sample_C7A_k141_45799_fragment_1	12632	24	6	0	Low-quality	32.95

Sample_C10A_k141_108715	60696	59	1	47	Low-quality	32.91
Sample_C7A_k141_86631	43824	58	12	0	Low-quality	32.91
Sample_T7B_k141_151969	21647	35	3	1	Low-quality	32.91
Sample_T17C_k141_5900	18346	17	0	4	Low-quality	32.9
Sample_T8C_k141_29520	53336	48	1	19	Low-quality	32.9
Sample_T8C_k141_78531	9847	10	0	3	Low-quality	32.9
Sample_C12B_k141_116531	14452	14	4	0	Low-quality	32.88
Sample_T4B_k141_325809	28436	24	1	10	Low-quality	32.87
Sample_T10B_k141_45338_fragment_1	19899	23	0	6	Low-quality	32.87
Sample_T5C_k141_179544	20930	27	0	3	Low-quality	32.86
Sample_T17C_k141_29132	9417	9	0	4	Low-quality	32.82
Sample_T13A_k141_124846	14408	15	7	1	Low-quality	32.82
Sample_T5C_k141_115001	18613	29	4	4	Low-quality	32.8
Sample_C2C_k141_73512	17074	33	1	2	Low-quality	32.79
Sample_T10C_k141_44444	14159	28	7	0	Low-quality	32.79
Sample_T12A_k141_286705	14559	25	2	1	Low-quality	32.79
Sample_C14B_k141_35048	13388	21	7	0	Low-quality	32.77
Sample_T8B_k141_246391	14459	16	0	1	Low-quality	32.76
Sample_T7B_k141_112689	15494	15	1	2	Low-quality	32.75
Sample_C13B_k141_72567	16019	30	6	0	Low-quality	32.75
Sample_C11A_k141_75972	12809	18	1	3	Low-quality	32.73
Sample_T4B_k141_127067	64589	68	2	48	Low-quality	32.73
Sample_C14C_k141_59059	36852	41	1	8	Low-quality	32.72
Sample_T15C_k141_113199	49722	35	0	18	Low-quality	32.72
Sample_T16C_k141_76714	13043	17	0	0	Low-quality	32.71
Sample_C1B_k141_4851	17855	31	8	0	Low-quality	32.66
Sample_C6B_k141_114669_fragment_1	19347	17	6	2	Low-quality	32.66
Sample_T8B_k141_31324	15422	20	7	1	Low-quality	32.66
Sample_T7A_k141_119875	52655	56	2	9	Low-quality	32.65
Sample_T5C_k141_31872	24862	19	0	3	Low-quality	32.64
Sample_T6B_k141_100918	12219	24	2	3	Low-quality	32.64
Sample_C12B_k141_35719	80948	72	1	32	Low-quality	32.62
Sample_C10A_k141_286318	18847	25	14	4	Low-quality	32.61
Sample_C2B_k141_56731	13270	25	4	3	Low-quality	32.59
Sample_T16B_k141_131473	10776	14	3	0	Low-quality	32.59
Sample_T17C_k141_1444	11371	13	6	0	Low-quality	32.59
Sample_T16B_k141_126275	55300	71	2	4	Low-quality	32.58
Sample_C7A_k141_45435_fragment_1	14534	28	4	2	Low-quality	32.56
Sample_T18B_k141_74729	80019	80	1	35	Low-quality	32.52

Sample_C8C_k141_89290	14491	17	2	2	Low-quality	32.51
Sample_T13B_k141_19022_fragment_1	13686	20	5	0	Low-quality	32.51
Sample_T4C_k141_11847	9373	14	0	2	Low-quality	32.49
Sample_C8B_k141_85668	14987	18	13	0	Low-quality	32.49
Sample_T11C_k141_156225_fragment_1	13726	18	5	1	Low-quality	32.48
Sample_C12C_k141_171733	32612	32	0	8	Low-quality	32.46
Sample_C2A_k141_212032_fragment_1	13448	15	3	0	Low-quality	32.45
Sample_T5B_k141_18057	30731	64	6	1	Low-quality	32.44
Sample_T10B_k141_97784	16994	36	6	1	Low-quality	32.43
Sample_T10C_k141_18923	9300	13	3	0	Low-quality	32.42
Sample_C3C_k141_137362	22244	28	1	8	Low-quality	32.41
Sample_T13C_k141_18595	12232	18	11	0	Low-quality	32.41
Sample_T3A_k141_84166_fragment_1	15278	20	14	2	Low-quality	32.41
Sample_C4C_k141_213989	51607	75	4	25	Low-quality	32.38
Sample_T7A_k141_207354	12883	17	0	2	Low-quality	32.38
Sample_T11C_k141_62205	15037	17	0	0	Low-quality	32.36
Sample_T3C_k141_120469	18075	12	0	3	Low-quality	32.33
Sample_C12A_k141_75593	19348	22	0	4	Low-quality	32.32
Sample_T13C_k141_119010	12041	6	1	0	Low-quality	32.31
Sample_C9C_k141_11313	13576	14	0	10	Low-quality	32.3
Sample_T4B_k141_35390	12328	17	4	0	Low-quality	32.3
Sample_C10B_k141_159234	12315	29	0	0	Low-quality	32.29
Sample_C2C_k141_217565	15285	19	5	0	Low-quality	32.27
Sample_C12A_k141_193723_fragment_4	13153	17	6	0	Low-quality	32.26
Sample_T8C_k141_146554_fragment_5	34766	44	6	4	Low-quality	32.26
Sample_T17A_k141_31894	13220	19	12	0	Low-quality	32.25
Sample_C14A_k141_221639	17738	24	12	0	Low-quality	32.23
Sample_T10A_k141_28926_fragment_1	11031	19	3	0	Low-quality	32.23
Sample_C14A_k141_32900	14382	18	6	0	Low-quality	32.22
Sample_C12A_k141_86393	13036	17	9	0	Low-quality	32.21
Sample_T13A_k141_82552	12893	23	10	0	Low-quality	32.2
Sample_T10B_k141_145824	16237	20	2	2	Low-quality	32.18
Sample_C11C_k141_3364_fragment_1	11684	14	10	0	Low-quality	32.17
Sample_T4B_k141_321552	12074	19	9	0	Low-quality	32.17
Sample_C10B_k141_19651	43129	52	0	7	Low-quality	32.14
Sample_C8A_k141_142771_fragment_1	17681	30	7	0	Low-quality	32.14
Sample_T3A_k141_842	15539	22	4	0	Low-quality	32.14
Sample_C3A_k141_120760_fragment_1	12889	19	1	1	Low-quality	32.12
Sample_T10B_k141_172618	56616	54	13	2	Low-quality	32.12

Sample_T12C_k141_6869	24021	39	4	3	Low-quality	32.12
Sample_C11A_k141_79656	76926	82	6	17	Low-quality	32.1
Sample_T1A_k141_15559	13582	21	15	0	Low-quality	32.1
Sample_C14C_k141_124115	11679	14	2	2	Low-quality	32.06
Sample_C10A_k141_227910	20003	32	8	1	Low-quality	32.05
Sample_C11A_k141_133431_fragment_1	19742	29	9	0	Low-quality	32.05
Sample_T7A_k141_190911	17003	31	2	4	Low-quality	32.05
Sample_T8C_k141_2714	8430	9	2	0	Low-quality	32.05
Sample_T7B_k141_135124	19036	32	1	1	Low-quality	32.04
Sample_C14A_k141_159413	14400	27	5	0	Low-quality	32.03
Sample_T5B_k141_13117	23745	12	0	3	Low-quality	32.01
Sample_C13C_k141_24552_fragment_1	16905	26	4	0	Low-quality	32
Sample_C4A_k141_126934	12970	9	0	1	Low-quality	31.99
Sample_C5C_k141_25550	34208	31	0	5	Low-quality	31.98
Sample_T12A_k141_317922	14292	20	6	0	Low-quality	31.95
Sample_T5A_k141_45267	33658	37	3	2	Low-quality	31.94
Sample_T12C_k141_24285_fragment_1	13087	13	4	2	Low-quality	31.93
Sample_T14A_k141_205005	15466	30	7	0	Low-quality	31.93
Sample_T15C_k141_50862_fragment_1	11669	13	0	0	Low-quality	31.92
Sample_C11A_k141_142925	15709	30	6	0	Low-quality	31.9
Sample_C2B_k141_141063_fragment_1	12992	12	2	1	Low-quality	31.9
Sample_C1C_k141_40649	12987	20	18	0	Low-quality	31.89
Sample_T16B_k141_65838	14872	24	13	1	Low-quality	31.88
Sample_C4B_k141_91216	50486	57	1	27	Low-quality	31.85
Sample_T13B_k141_104087	172502	173	2	145	Low-quality	31.84
Sample_C10A_k141_240059_fragment_1	12323	23	11	0	Low-quality	31.84
Sample_T2A_k141_144664	27531	27	1	7	Low-quality	31.82
Sample_C3A_k141_115940_fragment_1	13586	19	9	1	Low-quality	31.81
Sample_T16C_k141_1940_fragment_1	15573	20	4	1	Low-quality	31.8
Sample_C4C_k141_174468	112412	84	1	60	Low-quality	31.78
Sample_T4B_k141_332858	52445	103	10	2	Low-quality	31.78
Sample_T16B_k141_634	58571	53	1	32	Low-quality	31.77
Sample_C10A_k141_195880	11093	13	6	0	Low-quality	31.77
Sample_T6A_k141_10503	12512	19	12	0	Low-quality	31.74
Sample_T15B_k141_110781	15762	35	4	0	Low-quality	31.73
Sample_T6C_k141_211191	139127	125	12	43	Low-quality	31.72
Sample_C1B_k141_49343	14307	13	1	0	Low-quality	31.72
Sample_C12A_k141_48679	20299	21	1	13	Low-quality	31.7
Sample_C12A_k141_48887	11635	15	8	0	Low-quality	31.7

Sample_C10A_k141_31586	378060	344	1	268	Low-quality	31.69
Sample_C7A_k141_149120	12944	11	3	1	Low-quality	31.69
Sample_T8B_k141_66071	22524	22	1	10	Low-quality	31.69
Sample_C9A_k141_100511_fragment_1	31983	33	2	4	Low-quality	31.68
Sample_T2B_k141_297810	14221	15	1	1	Low-quality	31.67
Sample_T2C_k141_4053	9077	10	4	0	Low-quality	31.64
Sample_C1C_k141_126060	26161	29	1	11	Low-quality	31.62
Sample_T5A_k141_20006	16718	15	3	1	Low-quality	31.62
Sample_C9A_k141_55985	13099	23	9	0	Low-quality	31.61
Sample_T11B_k141_24758	19296	27	2	1	Low-quality	31.61
Sample_C13C_k141_57572	15453	22	19	0	Low-quality	31.6
Sample_C3C_k141_42994	12351	15	0	1	Low-quality	31.58
Sample_C2A_k141_174303	37488	52	6	3	Low-quality	31.58
Sample_T13B_k141_24386	11835	19	10	0	Low-quality	31.58
Sample_T7B_k141_82945	16881	21	13	1	Low-quality	31.58
Sample_T7C_k141_54544	18222	22	1	7	Low-quality	31.56
Sample_T10A_k141_115856	13144	19	11	0	Low-quality	31.54
Sample_C4B_k141_226552	11881	19	7	1	Low-quality	31.53
Sample_C8B_k141_192328	22248	19	1	10	Low-quality	31.52
Sample_C9B_k141_12935	24097	26	3	6	Low-quality	31.52
Sample_T17A_k141_39485	11480	15	9	0	Low-quality	31.5
Sample_C11B_k141_228792	11773	21	7	0	Low-quality	31.49
Sample_C10A_k141_105455	14834	19	9	0	Low-quality	31.48
Sample_T10B_k141_92189	16828	22	7	2	Low-quality	31.45
Sample_T12A_k141_54102	14218	15	14	0	Low-quality	31.45
Sample_T13A_k141_50813	30744	39	19	2	Low-quality	31.45
Sample_T3A_k141_48978	10204	11	6	0	Low-quality	31.44
Sample_T5C_k141_32173	19244	12	0	2	Low-quality	31.44
Sample_T12B_k141_71836	11875	14	4	0	Low-quality	31.43
Sample_C14C_k141_120478	11957	13	5	0	Low-quality	31.4
Sample_T3B_k141_48518	12895	26	1	0	Low-quality	31.39
Sample_T13C_k141_126870_fragment_1	13984	19	7	0	Low-quality	31.37
Sample_T6B_k141_84324	14306	20	6	2	Low-quality	31.35
Sample_C11B_k141_148759	19552	34	2	0	Low-quality	31.34
Sample_C12C_k141_170999	109998	96	1	65	Low-quality	31.3
Sample_T7C_k141_150027	14211	28	1	2	Low-quality	31.3
Sample_C9C_k141_57148	9574	4	0	0	Low-quality	31.28
Sample_T15C_k141_116632	15944	19	1	3	Low-quality	31.27
Sample_C11C_k141_56460_fragment_1	14537	10	0	2	Low-quality	31.27

Sample_T13B_k141_131493_fragment_1	20096	15	12	0	Low-quality	31.27
Sample_T11C_k141_135865	43385	43	1	15	Low-quality	31.26
Sample_C2A_k141_269521	15780	21	6	2	Low-quality	31.26
Sample_T15A_k141_178928	17077	32	8	0	Low-quality	31.26
Sample_C10A_k141_134474	27082	31	1	7	Low-quality	31.25
Sample_T5B_k141_34827_fragment_1	13787	24	1	3	Low-quality	31.25
Sample_C3B_k141_171334	18685	22	0	5	Low-quality	31.21
Sample_C2A_k141_274500	13678	16	4	2	Low-quality	31.21
Sample_C4A_k141_5657	11062	16	8	0	Low-quality	31.2
Sample_C3C_k141_130759	25487	19	0	12	Low-quality	31.19
Sample_T8A_k141_91744	16560	23	3	0	Low-quality	31.19
Sample_T15C_k141_30175_fragment_2	31204	51	6	0	Low-quality	31.18
Sample_C13A_k141_7170	33710	30	3	3	Low-quality	31.17
Sample_C6B_k141_51815	12585	20	16	0	Low-quality	31.14
Sample_T5B_k141_61616	30704	27	0	13	Low-quality	31.13
Sample_C12C_k141_72253_fragment_2	12906	14	4	1	Low-quality	31.12
Sample_C9C_k141_3116	12583	17	0	0	Low-quality	31.1
Sample_T4B_k141_73313	52095	53	1	26	Low-quality	31.09
Sample_C2A_k141_323696	21210	10	0	2	Low-quality	31.08
Sample_C2C_k141_261051	27759	33	1	8	Low-quality	31.08
Sample_T5A_k141_17986	20512	17	1	2	Low-quality	31.04
Sample_C2B_k141_217559	28635	51	8	1	Low-quality	31.04
Sample_T8C_k141_70755	17307	29	0	1	Low-quality	31.04
Sample_C12B_k141_163095	14656	20	12	0	Low-quality	31.03
Sample_C14A_k141_231849_fragment_2	12144	18	6	0	Low-quality	31.03
Sample_C11A_k141_163858	13227	20	0	0	Low-quality	31.02
Sample_T7A_k141_188517	48335	41	1	14	Low-quality	30.97
Sample_T2C_k141_156702	13541	32	5	0	Low-quality	30.97
Sample_T8C_k141_114753_fragment_1	27875	30	2	1	Low-quality	30.97
Sample_C13B_k141_88000	75207	79	1	12	Low-quality	30.96
Sample_C9C_k141_101762	17778	21	0	3	Low-quality	30.96
Sample_T2B_k141_141069	17311	24	1	6	Low-quality	30.96
Sample_C2A_k141_270489	14063	38	9	0	Low-quality	30.96
Sample_T2A_k141_76147	16708	35	7	0	Low-quality	30.96
Sample_T8A_k141_177403	10765	15	7	0	Low-quality	30.96
Sample_T6A_k141_190285	15849	19	0	3	Low-quality	30.95
Sample_C14A_k141_12744	67228	83	4	4	Low-quality	30.95
Sample_T16B_k141_143591	12656	21	8	0	Low-quality	30.95
Sample_T5B_k141_61288	18217	16	1	1	Low-quality	30.95

Sample_T5C_k141_139527	13724	17	15	0	Low-quality	30.95
Sample_C5B_k141_2995	16237	11	1	1	Low-quality	30.91
Sample_T15C_k141_115306	32428	67	11	3	Low-quality	30.91
Sample_C8A_k141_104521	73460	82	1	30	Low-quality	30.89
Sample_C3A_k141_110936	11031	11	6	0	Low-quality	30.88
Sample_C3A_k141_53051	14534	22	5	0	Low-quality	30.87
Sample_T8C_k141_120079_fragment_2	12246	13	3	2	Low-quality	30.84
Sample_C13B_k141_21620	25520	34	2	8	Low-quality	30.83
Sample_C5A_k141_87501	18019	29	6	0	Low-quality	30.82
Sample_T17A_k141_27306	27161	14	1	4	Low-quality	30.8
Sample_C8A_k141_4629	49658	52	2	11	Low-quality	30.79
Sample_C11C_k141_39722	18084	25	5	1	Low-quality	30.76
Sample_T4B_k141_101303	55730	54	1	25	Low-quality	30.75
Sample_T11C_k141_47323_fragment_1	15032	12	0	2	Low-quality	30.75
Sample_C6A_k141_111311	8814	12	3	0	Low-quality	30.73
Sample_T13B_k141_159519_fragment_1	12765	12	3	2	Low-quality	30.72
Sample_C13A_k141_7377	26254	40	8	1	Low-quality	30.7
Sample_T11B_k141_140929	15232	17	1	1	Low-quality	30.69
Sample_T15B_k141_66870	16688	7	2	0	Low-quality	30.69
Sample_T16C_k141_98167	13967	21	6	0	Low-quality	30.66
Sample_C12A_k141_79918	17970	27	6	0	Low-quality	30.65
Sample_C2C_k141_41261	47810	52	2	7	Low-quality	30.63
Sample_C6A_k141_197687_fragment_1	14358	11	1	0	Low-quality	30.62
Sample_T2C_k141_119643	11516	16	2	1	Low-quality	30.62
Sample_C8A_k141_1393	17216	14	3	1	Low-quality	30.6
Sample_T15C_k141_91106	23428	25	0	12	Low-quality	30.59
Sample_C8B_k141_47618	50304	64	3	25	Low-quality	30.58
Sample_C2B_k141_87666	12541	18	11	0	Low-quality	30.58
Sample_T6B_k141_4695	11007	12	3	0	Low-quality	30.58
Sample_T7A_k141_15235_fragment_1	16428	20	6	2	Low-quality	30.58
Sample_C12C_k141_87616	65117	70	4	16	Low-quality	30.57
Sample_C6C_k141_70137	23915	30	2	17	Low-quality	30.57
Sample_C4A_k141_11562_fragment_1	12654	17	3	0	Low-quality	30.56
Sample_T3A_k141_4237	11368	21	7	0	Low-quality	30.54
Sample_T5B_k141_42227	19518	29	8	1	Low-quality	30.53
Sample_C8C_k141_115296	12321	19	4	1	Low-quality	30.51
Sample_C11A_k141_187118	13019	14	2	1	Low-quality	30.51
Sample_T6B_k141_144618	29218	32	0	6	Low-quality	30.49
Sample_C4C_k141_168223	18157	32	5	1	Low-quality	30.48

Sample_T4C_k141_53877	56187	57	1	17	Low-quality	30.47
Sample_C8B_k141_122484	9980	17	4	0	Low-quality	30.47
Sample_T6A_k141_213273	16904	21	9	0	Low-quality	30.47
Sample_T8C_k141_100441	10075	20	4	0	Low-quality	30.46
Sample_C10C_k141_22046	11784	22	10	1	Low-quality	30.44
Sample_T6B_k141_81276	14590	18	10	1	Low-quality	30.41
Sample_T6B_k141_17826	13841	21	5	0	Low-quality	30.39
Sample_C4A_k141_77271	14812	22	11	1	Low-quality	30.38
Sample_T15A_k141_149743	19071	9	2	2	Low-quality	30.37
Sample_C11C_k141_181970	11967	15	5	0	Low-quality	30.37
Sample_T7A_k141_206865	5306	7	3	0	Low-quality	30.37
Sample_T4B_k141_166488	48961	53	1	22	Low-quality	30.36
Sample_C12A_k141_161659	14070	19	7	1	Low-quality	30.36
Sample_C12C_k141_13840_fragment_2	27306	36	6	3	Low-quality	30.35
Sample_T13A_k141_53782	12581	27	2	0	Low-quality	30.35
Sample_T11A_k141_58604	16534	13	0	3	Low-quality	30.34
Sample_C12B_k141_23843	23171	20	5	2	Low-quality	30.33
Sample_T8C_k141_160627	12757	9	2	0	Low-quality	30.29
Sample_C3A_k141_23689	48970	57	1	14	Low-quality	30.28
Sample_C1A_k141_216118	20007	26	1	7	Low-quality	30.27
Sample_T12A_k141_198558	75103	66	1	42	Low-quality	30.27
Sample_C1C_k141_7540	12172	16	12	0	Low-quality	30.27
Sample_T8B_k141_15291_fragment_1	15778	20	12	1	Low-quality	30.26
Sample_T11C_k141_153271	8916	13	1	0	Low-quality	30.25
Sample_C4C_k141_105145	11892	26	3	0	Low-quality	30.24
Sample_C13B_k141_81125	41965	43	1	11	Low-quality	30.23
Sample_C2B_k141_321649_fragment_3	14056	14	0	3	Low-quality	30.23
Sample_T18C_k141_69732	8673	9	3	0	Low-quality	30.23
Sample_T2B_k141_126325	17596	18	3	0	Low-quality	30.22
Sample_T8A_k141_105033	8671	11	3	0	Low-quality	30.22
Sample_C7B_k141_4777	13075	22	7	1	Low-quality	30.18
Sample_C2A_k141_191160	22846	29	18	3	Low-quality	30.17
Sample_C12C_k141_121426_fragment_1	17429	28	7	4	Low-quality	30.11
Sample_T2C_k141_28181	12729	24	4	0	Low-quality	30.09
Sample_T14A_k141_152569	19110	18	0	7	Low-quality	30.07
Sample_T10A_k141_114826	11649	20	12	0	Low-quality	30.05
Sample_C8C_k141_83426	26422	23	0	7	Low-quality	30.04
Sample_T6A_k141_94047	8501	12	0	0	Low-quality	30.04
Sample_C5C_k141_15520	12610	13	9	0	Low-quality	30.04

Sample_T14B_k141_66024	19847	20	1	3	Low-quality	30.03
Sample_T16B_k141_81670	10977	9	4	0	Low-quality	30.02
Sample_C9A_k141_94696	14001	17	10	0	Low-quality	30.01
Sample_T12A_k141_178504	10021	11	1	4	Low-quality	30
Sample_C12A_k141_96389	10769	21	3	1	Low-quality	29.99
Sample_T12C_k141_49291	13357	17	0	1	Low-quality	29.99
Sample_T14A_k141_232959_fragment_2	14670	16	10	1	Low-quality	29.96
Sample_T14B_k141_34194	10676	17	1	1	Low-quality	29.92
Sample_C4C_k141_187377	15250	27	4	0	Low-quality	29.92
Sample_T13B_k141_50310	11481	18	11	0	Low-quality	29.92
Sample_T11C_k141_83978_fragment_1	15196	17	0	0	Low-quality	29.91
Sample_T2A_k141_109026	11049	22	6	0	Low-quality	29.91
Sample_C11C_k141_70397	26048	29	3	0	Low-quality	29.9
Sample_T10A_k141_137934	11863	22	3	0	Low-quality	29.9
Sample_T5A_k141_48338_fragment_1	12193	20	8	0	Low-quality	29.9
Sample_C4B_k141_17051	11904	24	2	1	Low-quality	29.89
Sample_T1B_k141_3271	19308	29	13	0	Low-quality	29.89
Sample_C11A_k141_160798	81443	78	1	55	Low-quality	29.88
Sample_T12C_k141_174032	12908	18	0	0	Low-quality	29.88
Sample_T13C_k141_45263	10618	21	3	1	Low-quality	29.88
Sample_C10A_k141_184681	26607	33	1	8	Low-quality	29.87
Sample_T3C_k141_45673_fragment_1	12582	26	5	0	Low-quality	29.87
Sample_C11B_k141_111750	83325	85	1	56	Low-quality	29.86
Sample_C7A_k141_133732	8567	6	0	2	Low-quality	29.86
Sample_C9A_k141_71878_fragment_1	17653	25	11	4	Low-quality	29.86
Sample_T8B_k141_125867	22747	26	1	16	Low-quality	29.85
Sample_T12A_k141_274537	13851	12	0	10	Low-quality	29.82
Sample_C3A_k141_154718	44163	48	15	0	Low-quality	29.82
Sample_C12C_k141_105184	12508	16	3	1	Low-quality	29.81
Sample_C4C_k141_61420	25849	22	6	2	Low-quality	29.81
Sample_C4A_k141_166777	17765	20	1	2	Low-quality	29.8
Sample_C3A_k141_164179	12080	19	0	0	Low-quality	29.8
Sample_T12B_k141_39135	11559	18	5	0	Low-quality	29.8
Sample_T4B_k141_321471	11179	18	9	0	Low-quality	29.79
Sample_C10B_k141_133614	11371	8	0	0	Low-quality	29.78
Sample_C2C_k141_134711	26896	28	1	17	Low-quality	29.78
Sample_T10C_k141_68435	22597	20	0	6	Low-quality	29.78
Sample_T10B_k141_152484	39509	61	12	0	Low-quality	29.78
Sample_T15A_k141_56003_fragment_2	60425	93	18	7	Low-quality	29.78

Sample_T5A_k141_25235_fragment_1	24014	16	7	1	Low-quality	29.78
Sample_T15B_k141_45835	22315	19	1	6	Low-quality	29.76
Sample_C11C_k141_149286	10706	18	3	0	Low-quality	29.76
Sample_T16B_k141_32254	36376	44	1	16	Low-quality	29.74
Sample_T18A_k141_176287	11769	11	1	7	Low-quality	29.74
Sample_T4B_k141_96417	12494	10	0	8	Low-quality	29.73
Sample_C6B_k141_1265	17794	23	0	4	Low-quality	29.72
Sample_C3C_k141_34042	23938	30	10	6	Low-quality	29.72
Sample_C6C_k141_144156	17426	24	12	0	Low-quality	29.72
Sample_C3C_k141_269835	8984	11	0	0	Low-quality	29.71
Sample_C2B_k141_144072	13434	14	4	0	Low-quality	29.71
Sample_T6C_k141_176492	11088	17	5	0	Low-quality	29.71
Sample_T17A_k141_6722_fragment_1	15476	21	1	2	Low-quality	29.69
Sample_T11B_k141_160938	10625	20	2	1	Low-quality	29.66
Sample_C7B_k141_25723	54635	44	1	28	Low-quality	29.63
Sample_T18A_k141_130510	12082	11	3	0	Low-quality	29.63
Sample_T7A_k141_173988	18148	32	6	1	Low-quality	29.62
Sample_C4A_k141_41239	13760	13	0	1	Low-quality	29.6
Sample_C11B_k141_182116	11046	18	8	0	Low-quality	29.56
Sample_C12C_k141_77235_fragment_2	11397	14	3	1	Low-quality	29.56
Sample_T2B_k141_99989	13414	26	5	0	Low-quality	29.55
Sample_C1C_k141_157143	23757	37	2	9	Low-quality	29.52
Sample_T7A_k141_99989	25580	35	1	9	Low-quality	29.52
Sample_C12B_k141_190568	19502	23	1	6	Low-quality	29.51
Sample_T11B_k141_5923	18152	14	1	3	Low-quality	29.51
Sample_C10A_k141_98475	19323	13	2	2	Low-quality	29.48
Sample_T18C_k141_870_fragment_1	26034	54	7	1	Low-quality	29.48
Sample_C6C_k141_45512	19377	30	4	3	Low-quality	29.47
Sample_C11A_k141_66389_fragment_1	12623	15	1	2	Low-quality	29.47
Sample_T5C_k141_35368	16294	25	1	1	Low-quality	29.47
Sample_C5C_k141_2801	12381	11	0	5	Low-quality	29.46
Sample_T5C_k141_69404	10405	8	3	0	Low-quality	29.46
Sample_C4B_k141_7085	11831	13	0	3	Low-quality	29.45
Sample_C11A_k141_142292	11001	20	5	0	Low-quality	29.44
Sample_T8B_k141_266377	19442	23	1	11	Low-quality	29.42
Sample_T5A_k141_49578	46867	49	4	11	Low-quality	29.41
Sample_C6C_k141_37072	10725	13	9	0	Low-quality	29.41
Sample_C11C_k141_115027	15767	16	11	2	Low-quality	29.4
Sample_T4B_k141_158219	12492	18	7	0	Low-quality	29.39

Sample_T4C_k141_32542	17351	21	7	0	Low-quality	29.39
Sample_T5A_k141_21932	10246	18	6	0	Low-quality	29.39
Sample_T16C_k141_54749	11396	26	9	1	Low-quality	29.38
Sample_T8A_k141_119754	26161	28	1	4	Low-quality	29.37
Sample_T10A_k141_122708	12261	29	5	1	Low-quality	29.37
Sample_T13A_k141_104169	11899	13	9	0	Low-quality	29.37
Sample_T18B_k141_93928	25403	22	1	5	Low-quality	29.36
Sample_T10A_k141_112696	11645	11	4	0	Low-quality	29.36
Sample_T6A_k141_200476	10428	19	6	0	Low-quality	29.35
Sample_T8C_k141_109517	13541	16	8	0	Low-quality	29.31
Sample_C3B_k141_218789	11219	16	14	0	Low-quality	29.3
Sample_T6A_k141_34633	20616	20	0	14	Low-quality	29.28
Sample_C3B_k141_277324	10310	17	13	0	Low-quality	29.26
Sample_C10A_k141_258322_fragment_1	12968	16	7	0	Low-quality	29.23
Sample_C10C_k141_5416	11184	4	0	0	Low-quality	29.2
Sample_T15C_k141_94815	32058	26	1	6	Low-quality	29.19
Sample_C12C_k141_100426	21494	23	8	2	Low-quality	29.17
Sample_C11A_k141_128331	12546	15	0	3	Low-quality	29.16
Sample_C6B_k141_63853	9972	14	7	1	Low-quality	29.16
Sample_T4B_k141_214281	25261	30	1	10	Low-quality	29.15
Sample_C2B_k141_317205	25860	38	2	2	Low-quality	29.14
Sample_T15A_k141_98862	12446	8	0	3	Low-quality	29.14
Sample_T11B_k141_112640	11122	25	3	0	Low-quality	29.14
Sample_T3A_k141_3187	12241	24	0	23	Low-quality	29.13
Sample_C13A_k141_109937	20276	17	1	4	Low-quality	29.12
Sample_C9C_k141_80830	13246	17	2	0	Low-quality	29.12
Sample_T11B_k141_118968	10279	17	4	1	Low-quality	29.12
Sample_C11A_k141_8266	10950	16	11	0	Low-quality	29.11
Sample_T3B_k141_170184	9806	15	6	0	Low-quality	29.1
Sample_T10B_k141_93363	31110	32	2	0	Low-quality	29.09
Sample_C4B_k141_211444_fragment_1	12874	26	4	1	Low-quality	29.06
Sample_C5A_k141_48415	23008	36	5	1	Low-quality	29.06
Sample_T5A_k141_36490	11545	15	8	0	Low-quality	29.06
Sample_C11B_k141_138689	15193	24	13	0	Low-quality	29.05
Sample_C2A_k141_173731	12737	20	3	0	Low-quality	29.05
Sample_T12A_k141_141819	11075	14	8	0	Low-quality	29.05
Sample_T13C_k141_73490	16632	16	0	5	Low-quality	29.04
Sample_T15A_k141_85080	11054	19	6	0	Low-quality	29.03
Sample_C3C_k141_265507	15443	8	0	2	Low-quality	29.02

Sample_C12A_k141_108513	10437	18	1	1	Low-quality	29.02
Sample_C10B_k141_42472	35697	36	1	7	Low-quality	29.01
Sample_T7A_k141_11493	17369	22	1	3	Low-quality	29.01
Sample_T13C_k141_27827	10109	16	5	0	Low-quality	29.01
Sample_T11C_k141_118540	15478	19	0	2	Low-quality	29
Sample_T6B_k141_20340	11720	16	0	4	Low-quality	28.99
Sample_C3C_k141_147782	8317	10	3	0	Low-quality	28.99
Sample_T3A_k141_68000	10776	12	1	4	Low-quality	28.98
Sample_C5A_k141_31333	29874	43	9	0	Low-quality	28.98
Sample_C3A_k141_184669	28726	21	0	7	Low-quality	28.95
Sample_T8A_k141_232027	30769	33	7	0	Low-quality	28.94
Sample_C9C_k141_43318	19097	18	2	3	Low-quality	28.93
Sample_T5B_k141_42945	12153	5	0	3	Low-quality	28.92
Sample_T11A_k141_103002	12145	12	0	12	Low-quality	28.9
Sample_T15A_k141_96488	15973	30	0	0	Low-quality	28.9
Sample_C2A_k141_155435_fragment_1	35547	27	6	0	Low-quality	28.89
Sample_T8A_k141_125578	12452	12	0	0	Low-quality	28.88
Sample_T11A_k141_8772	12688	20	0	7	Low-quality	28.87
Sample_T3C_k141_14332	18016	19	2	0	Low-quality	28.87
Sample_C3C_k141_143585	11188	16	8	0	Low-quality	28.86
Sample_T11C_k141_153595_fragment_1	10206	22	4	1	Low-quality	28.86
Sample_C6B_k141_46886	44583	43	1	32	Low-quality	28.85
Sample_T18C_k141_81701	10546	10	0	3	Low-quality	28.85
Sample_C5A_k141_204424	17795	22	18	0	Low-quality	28.83
Sample_C3C_k141_249249	23391	21	1	8	Low-quality	28.82
Sample_C4A_k141_15720_fragment_1	16116	26	1	2	Low-quality	28.82
Sample_T8C_k141_154464	9000	19	3	0	Low-quality	28.82
Sample_C3C_k141_141826	9284	10	7	0	Low-quality	28.79
Sample_T12A_k141_182904	9551	12	4	0	Low-quality	28.76
Sample_T7A_k141_30759_fragment_1	10468	21	9	0	Low-quality	28.76
Sample_T17A_k141_22491	19000	24	1	6	Low-quality	28.75
Sample_T12B_k141_126571	16352	25	4	3	Low-quality	28.75
Sample_T6C_k141_221062	21306	31	9	9	Low-quality	28.72
Sample_C9A_k141_118183	16003	15	0	5	Low-quality	28.7
Sample_T3A_k141_66785	128286	126	11	56	Low-quality	28.7
Sample_C12C_k141_169964_fragment_2	17063	21	9	0	Low-quality	28.7
Sample_C10C_k141_190820	27023	23	8	3	Low-quality	28.68
Sample_T2A_k141_62482	11157	27	8	0	Low-quality	28.68
Sample_C6C_k141_206027	15548	14	1	10	Low-quality	28.67

Sample_C12A_k141_1787	23603	35	4	4	Low-quality	28.67
Sample_T6C_k141_82876	10763	14	10	0	Low-quality	28.67
Sample_C3C_k141_216221	10939	13	0	7	Low-quality	28.66
Sample_C2B_k141_19531	10147	24	2	0	Low-quality	28.66
Sample_T8C_k141_124666_fragment_1	12368	14	2	0	Low-quality	28.66
Sample_C12B_k141_56340	155005	153	1	84	Low-quality	28.61
Sample_C10A_k141_193547	56993	52	1	21	Low-quality	28.6
Sample_T5B_k141_24400	11199	15	3	0	Low-quality	28.59
Sample_C5A_k141_221994	23259	21	2	3	Low-quality	28.58
Sample_C5C_k141_59984	8065	9	0	2	Low-quality	28.58
Sample_T6A_k141_216640	54286	49	0	38	Low-quality	28.57
Sample_T17A_k141_17991	14495	23	14	0	Low-quality	28.57
Sample_T8C_k141_93941	9782	23	6	0	Low-quality	28.57
Sample_C10C_k141_187139	37306	36	2	12	Low-quality	28.56
Sample_C8B_k141_44187_fragment_1	17861	17	10	4	Low-quality	28.56
Sample_T1A_k141_18058_fragment_1	11565	22	9	0	Low-quality	28.56
Sample_T1A_k141_18080	12082	18	12	0	Low-quality	28.55
Sample_C1B_k141_180830	13356	27	3	2	Low-quality	28.53
Sample_T16C_k141_92461	11988	16	0	12	Low-quality	28.52
Sample_T16C_k141_87505	18532	14	3	2	Low-quality	28.52
Sample_C12C_k141_35673	20974	24	0	9	Low-quality	28.51
Sample_C2C_k141_59827	22495	15	0	3	Low-quality	28.51
Sample_C5A_k141_89094	17070	22	1	1	Low-quality	28.51
Sample_C10B_k141_5638	17061	21	0	5	Low-quality	28.5
Sample_C8A_k141_55344_fragment_1	17019	26	4	3	Low-quality	28.48
Sample_T5A_k141_35000	11448	23	3	1	Low-quality	28.45
Sample_C10C_k141_117413	23271	23	10	2	Low-quality	28.44
Sample_T8A_k141_253946	12097	18	9	0	Low-quality	28.44
Sample_T10A_k141_121355	10817	12	3	0	Low-quality	28.43
Sample_C13C_k141_38693	14553	20	0	2	Low-quality	28.42
Sample_T11A_k141_96771	45361	46	1	7	Low-quality	28.42
Sample_C13A_k141_15538	11233	14	11	0	Low-quality	28.42
Sample_C11A_k141_105896	20816	20	0	4	Low-quality	28.4
Sample_C10C_k141_66229	12775	19	8	0	Low-quality	28.39
Sample_C3A_k141_112087	10240	15	12	0	Low-quality	28.39
Sample_T11C_k141_131974	28823	27	3	4	Low-quality	28.38
Sample_C14A_k141_195363	13111	21	1	1	Low-quality	28.35
Sample_C11A_k141_38792	16513	12	1	5	Low-quality	28.34
Sample_C13B_k141_62488	13822	20	1	4	Low-quality	28.33

Sample_T5B_k141_8431	17477	20	1	6	Low-quality	28.33
Sample_T15A_k141_179915	10836	21	7	0	Low-quality	28.33
Sample_C2B_k141_157053	9679	26	2	0	Low-quality	28.32
Sample_C6B_k141_16100	12539	8	3	0	Low-quality	28.3
Sample_C3C_k141_257346	10860	19	1	1	Low-quality	28.29
Sample_C11C_k141_117245	9872	14	7	0	Low-quality	28.28
Sample_C14B_k141_37670	16288	20	10	1	Low-quality	28.28
Sample_T10B_k141_93412	5105	5	0	0	Low-quality	28.27
Sample_C9A_k141_88246	11280	14	5	0	Low-quality	28.26
Sample_C8A_k141_84827	154685	138	1	78	Low-quality	28.25
Sample_C12A_k141_64577	11491	19	1	0	Low-quality	28.25
Sample_C4B_k141_74342	10826	15	7	0	Low-quality	28.24
Sample_T4B_k141_73986	27459	30	2	9	Low-quality	28.22
Sample_C5C_k141_89603	10847	4	0	0	Low-quality	28.21
Sample_T17B_k141_2956_fragment_1	78124	87	13	16	Low-quality	28.21
Sample_T18B_k141_59164_fragment_1	10657	8	4	0	Low-quality	28.21
Sample_T4B_k141_208329	11482	11	3	0	Low-quality	28.21
Sample_C12B_k141_109721	10800	21	5	0	Low-quality	28.2
Sample_T13C_k141_89677	20235	12	0	4	Low-quality	28.19
Sample_T10A_k141_121179	12864	19	8	0	Low-quality	28.19
Sample_C6B_k141_9856	69925	65	1	44	Low-quality	28.18
Sample_T10A_k141_57443	11843	22	3	3	Low-quality	28.17
Sample_T11B_k141_70301	22761	26	1	8	Low-quality	28.17
Sample_T8B_k141_163705	12268	26	6	3	Low-quality	28.17
Sample_T4A_k141_79761	13254	17	9	1	Low-quality	28.17
Sample_T15C_k141_107380	15766	21	5	0	Low-quality	28.16
Sample_T6B_k141_188872	11995	18	0	2	Low-quality	28.15
Sample_C6B_k141_109278	18590	25	1	8	Low-quality	28.13
Sample_T2B_k141_263327	240495	238	2	161	Low-quality	28.13
Sample_C2B_k141_106640	10475	13	11	0	Low-quality	28.13
Sample_T10A_k141_162214	23123	28	9	0	Low-quality	28.13
Sample_T10A_k141_10663	11246	15	9	0	Low-quality	28.12
Sample_T5B_k141_49202	8061	8	0	2	Low-quality	28.1
Sample_T8C_k141_27844	11128	13	11	0	Low-quality	28.09
Sample_C8C_k141_58665	10769	8	0	3	Low-quality	28.07
Sample_C2B_k141_325329	25902	32	8	1	Low-quality	28.07
Sample_T5C_k141_8829_fragment_1	15838	15	0	1	Low-quality	28.06
Sample_T8A_k141_30102	15717	23	2	1	Low-quality	28.05
Sample_C1B_k141_12132	21943	17	0	5	Low-quality	28.04

Sample_T5A_k141_55034_fragment_3	15940	17	2	2	Low-quality	28.04
Sample_C10B_k141_161765	13620	20	0	4	Low-quality	28.03
Sample_C2A_k141_231114	9464	12	8	0	Low-quality	28.03
Sample_C2C_k141_286773	36551	34	1	20	Low-quality	28.01
Sample_T7B_k141_105499	13113	15	8	0	Low-quality	28.01
Sample_T6A_k141_11545	23177	28	2	5	Low-quality	28
Sample_C6A_k141_54130	13187	20	2	1	Low-quality	28
Sample_C3B_k141_175637	51614	44	1	9	Low-quality	27.99
Sample_T2C_k141_10155	12564	21	2	1	Low-quality	27.99
Sample_T8C_k141_64540	12923	15	8	0	Low-quality	27.98
Sample_C10A_k141_269818	10307	14	6	0	Low-quality	27.97
Sample_C13C_k141_90156	35894	34	0	29	Low-quality	27.96
Sample_T7A_k141_133501	18711	19	5	2	Low-quality	27.96
Sample_C4C_k141_266366	85796	93	1	11	Low-quality	27.95
Sample_T12B_k141_24644_fragment_1	21669	31	7	3	Low-quality	27.95
Sample_T4B_k141_202926_fragment_1	24895	24	1	1	Low-quality	27.95
Sample_T3B_k141_10517	14308	18	0	1	Low-quality	27.94
Sample_T15A_k141_89573	5418	10	3	0	Low-quality	27.93
Sample_T10B_k141_132014	27500	23	0	15	Low-quality	27.91
Sample_C3C_k141_212726	11080	19	6	1	Low-quality	27.9
Sample_T2C_k141_86063	14019	10	0	4	Low-quality	27.9
Sample_T4B_k141_87151_fragment_1	50415	55	14	1	Low-quality	27.89
Sample_T1A_k141_40852	13966	22	5	3	Low-quality	27.88
Sample_C4B_k141_81229_fragment_1	11287	18	5	0	Low-quality	27.87
Sample_T12B_k141_55728	15534	11	0	5	Low-quality	27.86
Sample_C10B_k141_153616	30576	28	1	10	Low-quality	27.84
Sample_C12A_k141_210095	9706	15	7	0	Low-quality	27.84
Sample_C3C_k141_226940	11714	8	0	3	Low-quality	27.82
Sample_C12C_k141_1385	12903	11	1	2	Low-quality	27.81
Sample_C5A_k141_40767	150551	143	1	80	Low-quality	27.79
Sample_T4B_k141_155194	48063	40	1	24	Low-quality	27.79
Sample_C10B_k141_105714	15398	26	1	1	Low-quality	27.78
Sample_C3C_k141_198476	14167	21	6	1	Low-quality	27.78
Sample_T7A_k141_75924	16418	26	5	0	Low-quality	27.77
Sample_T6A_k141_35854	9626	4	0	1	Low-quality	27.76
Sample_T8B_k141_214476	21276	28	1	8	Low-quality	27.76
Sample_C11C_k141_174699	10395	12	8	0	Low-quality	27.76
Sample_C12C_k141_224133	12529	18	4	0	Low-quality	27.76
Sample_T4B_k141_86319	10114	24	5	0	Low-quality	27.75

Sample_C6B_k141_74095	10263	13	0	0	Low-quality	27.73
Sample_T6A_k141_207743	10544	13	0	3	Low-quality	27.72
Sample_T7A_k141_150726	10568	12	2	1	Low-quality	27.72
Sample_C7A_k141_38668	10476	22	2	0	Low-quality	27.72
Sample_C14B_k141_98371	20890	25	2	6	Low-quality	27.69
Sample_C1A_k141_152935_fragment_3	10907	19	2	0	Low-quality	27.69
Sample_T11C_k141_54784	11733	18	5	0	Low-quality	27.69
Sample_T5A_k141_29596	11554	22	4	2	Low-quality	27.68
Sample_C8B_k141_164391	13338	16	0	2	Low-quality	27.67
Sample_T17B_k141_9762	24921	21	5	0	Low-quality	27.67
Sample_T4B_k141_102245	11203	15	3	0	Low-quality	27.67
Sample_T18C_k141_18333	16189	28	9	3	Low-quality	27.66
Sample_T4B_k141_203269_fragment_1	51865	85	13	3	Low-quality	27.66
Sample_T5C_k141_149558	22342	27	1	6	Low-quality	27.65
Sample_C11B_k141_171985	21042	21	0	5	Low-quality	27.64
Sample_C3B_k141_47266	8595	3	3	0	Low-quality	27.61
Sample_T6B_k141_125738	17604	20	0	10	Low-quality	27.61
Sample_C12C_k141_232893	18919	26	2	1	Low-quality	27.6
Sample_T7C_k141_132078	14704	21	2	3	Low-quality	27.59
Sample_C10C_k141_11990	10060	23	6	0	Low-quality	27.59
Sample_T12A_k141_78502	9908	19	2	0	Low-quality	27.55
Sample_T5C_k141_62248	11301	9	5	0	Low-quality	27.54
Sample_C14A_k141_24156	12216	20	3	0	Low-quality	27.53
Sample_C2A_k141_146808	23858	21	1	10	Low-quality	27.53
Sample_T7A_k141_676	41090	39	1	20	Low-quality	27.53
Sample_T2B_k141_32051	13061	19	14	0	Low-quality	27.53
Sample_T7A_k141_14540	25151	20	1	11	Low-quality	27.52
Sample_C4B_k141_214007	10526	13	5	0	Low-quality	27.5
Sample_C2C_k141_17348	32207	34	0	16	Low-quality	27.49
Sample_C9A_k141_68765	16459	19	0	3	Low-quality	27.49
Sample_T13B_k141_99505	22213	39	1	2	Low-quality	27.49
Sample_T8B_k141_52469	21591	11	0	3	Low-quality	27.48
Sample_T11B_k141_14420	11553	8	0	6	Low-quality	27.46
Sample_T2A_k141_247987	11793	17	6	0	Low-quality	27.46
Sample_T10B_k141_172725	9519	5	0	1	Low-quality	27.45
Sample_T5B_k141_6767	10635	20	2	0	Low-quality	27.45
Sample_C14C_k141_84102	12757	14	0	3	Low-quality	27.44
Sample_C3C_k141_202818	8909	16	7	0	Low-quality	27.44
Sample_C6B_k141_105384	12449	12	4	0	Low-quality	27.44

Sample_T2C_k141_122773	21081	21	0	2	Low-quality	27.43
Sample_T3A_k141_105321	16421	22	1	4	Low-quality	27.43
Sample_T7A_k141_55858	50581	41	1	10	Low-quality	27.43
Sample_T2A_k141_45744_fragment_1	11776	12	1	0	Low-quality	27.42
Sample_T6C_k141_166631_fragment_1	10335	8	2	0	Low-quality	27.42
Sample_C14B_k141_45091	11201	19	10	0	Low-quality	27.39
Sample_T17C_k141_20955	11442	16	11	0	Low-quality	27.39
Sample_C8B_k141_28940	50476	50	1	17	Low-quality	27.38
Sample_T12A_k141_280062	14972	20	1	2	Low-quality	27.38
Sample_T12A_k141_241952	9781	17	6	1	Low-quality	27.38
Sample_T12A_k141_287926_fragment_2	43631	37	13	1	Low-quality	27.37
Sample_C10A_k141_278829	9674	20	1	0	Low-quality	27.36
Sample_T11B_k141_124756	22372	32	3	7	Low-quality	27.32
Sample_T12B_k141_135256	19339	17	0	3	Low-quality	27.31
Sample_C10A_k141_56083	12678	16	12	0	Low-quality	27.3
Sample_C4A_k141_10275	29009	32	4	0	Low-quality	27.3
Sample_T11C_k141_24267	19004	16	1	6	Low-quality	27.29
Sample_C2C_k141_171037_fragment_2	12982	16	7	0	Low-quality	27.29
Sample_C12A_k141_49930	10651	16	4	0	Low-quality	27.27
Sample_T8C_k141_17789	15156	25	4	0	Low-quality	27.27
Sample_C5A_k141_71118	25208	18	1	0	Low-quality	27.25
Sample_T6B_k141_63828	10194	12	1	0	Low-quality	27.24
Sample_C5A_k141_261759_fragment_2	10992	15	2	1	Low-quality	27.23
Sample_T1B_k141_19284	16293	17	0	1	Low-quality	27.21
Sample_C4C_k141_87890	46134	43	11	8	Low-quality	27.21
Sample_T14B_k141_3145	12671	18	9	0	Low-quality	27.21
Sample_T8B_k141_273011	12482	17	16	0	Low-quality	27.21
Sample_C3A_k141_257244	11773	6	0	0	Low-quality	27.19
Sample_T12B_k141_17215	25817	17	0	10	Low-quality	27.19
Sample_C3C_k141_65020_fragment_2	29413	26	9	3	Low-quality	27.18
Sample_T4B_k141_77653	25075	31	15	0	Low-quality	27.18
Sample_C3B_k141_47636	23513	16	1	10	Low-quality	27.17
Sample_T4A_k141_24980	12457	22	2	0	Low-quality	27.17
Sample_T16B_k141_17501_fragment_3	22320	26	1	4	Low-quality	27.15
Sample_T1A_k141_29580	16670	33	6	0	Low-quality	27.14
Sample_T8A_k141_138307	10386	14	7	0	Low-quality	27.14
Sample_T11B_k141_38964	14370	19	0	3	Low-quality	27.13
Sample_C5A_k141_213354	29926	37	1	18	Low-quality	27.12
Sample_C7C_k141_79741	10492	19	3	0	Low-quality	27.12

Sample_T7B_k141_38747	10334	14	9	0	Low-quality	27.11
Sample_T17B_k141_25153	15422	20	7	1	Low-quality	27.1
Sample_T14A_k141_128825_fragment_1	35567	37	1	4	Low-quality	27.08
Sample_T8A_k141_85283	16207	18	0	1	Low-quality	27.07
Sample_C6B_k141_16707	11994	18	8	0	Low-quality	27.07
Sample_T7B_k141_84802	14475	18	12	0	Low-quality	27.07
Sample_C6C_k141_230909	10289	14	9	0	Low-quality	27.06
Sample_T4B_k141_295280	41335	44	0	28	Low-quality	27.05
Sample_C1C_k141_72601	10007	14	3	0	Low-quality	27.05
Sample_T2B_k141_300985	11077	11	0	2	Low-quality	27.04
Sample_C4C_k141_34362	12902	18	4	0	Low-quality	27.03
Sample_C4A_k141_97556	23677	52	7	3	Low-quality	27.01
Sample_C6C_k141_178224_fragment_1	33466	29	9	4	Low-quality	27.01
Sample_T10B_k141_159432	17851	19	1	1	Low-quality	27.01
Sample_T7A_k141_88222	6125	5	0	0	Low-quality	27
Sample_C2B_k141_209646	10055	11	9	0	Low-quality	27
Sample_T6C_k141_144045	10840	18	9	3	Low-quality	26.99
Sample_T8C_k141_147089	9675	17	8	0	Low-quality	26.99
Sample_C10A_k141_124524	10174	16	4	0	Low-quality	26.98
Sample_T12B_k141_33855	11364	12	4	1	Low-quality	26.96
Sample_T4B_k141_199658	24877	42	6	1	Low-quality	26.96
Sample_T8B_k141_200842	12620	26	2	1	Low-quality	26.96
Sample_C4A_k141_90094	16006	28	4	1	Low-quality	26.95
Sample_T10B_k141_181384	14329	15	1	3	Low-quality	26.95
Sample_T2C_k141_149009	15732	17	0	3	Low-quality	26.95
Sample_T4B_k141_365019	29389	50	5	3	Low-quality	26.95
Sample_C8B_k141_114079	15089	17	1	4	Low-quality	26.92
Sample_C11A_k141_184931	10776	11	7	0	Low-quality	26.91
Sample_C8C_k141_123048	9876	14	3	0	Low-quality	26.91
Sample_T3B_k141_168477	13292	21	7	2	Low-quality	26.91
Sample_T3C_k141_138243	11282	11	4	1	Low-quality	26.91
Sample_T15A_k141_156308	15207	17	1	0	Low-quality	26.89
Sample_C4B_k141_186812	14578	20	1	1	Low-quality	26.89
Sample_C2C_k141_66874	16576	24	1	11	Low-quality	26.87
Sample_T12A_k141_225977	12583	14	4	0	Low-quality	26.86
Sample_C11B_k141_204329	21695	25	1	9	Low-quality	26.85
Sample_T8C_k141_150802_fragment_1	14039	20	9	1	Low-quality	26.85
Sample_C12C_k141_207127	11879	14	9	0	Low-quality	26.83
Sample_C3C_k141_113531	15511	32	2	0	Low-quality	26.83

Sample_T5C_k141_131369	21481	28	2	2	Low-quality	26.81
Sample_T10B_k141_37607	9594	16	1	1	Low-quality	26.8
Sample_T6C_k141_92747	28350	45	6	3	Low-quality	26.77
Sample_T13B_k141_57317_fragment_1	14324	26	16	2	Low-quality	26.75
Sample_T3A_k141_2793	9859	7	0	0	Low-quality	26.75
Sample_T10A_k141_64087	12432	10	0	3	Low-quality	26.74
Sample_C14A_k141_40347	16768	17	3	2	Low-quality	26.72
Sample_C5B_k141_55388_fragment_1	14289	14	8	2	Low-quality	26.72
Sample_T10B_k141_177810	35569	54	10	0	Low-quality	26.72
Sample_C6A_k141_95852	23222	21	1	9	Low-quality	26.71
Sample_T10A_k141_144074	91735	86	5	36	Low-quality	26.71
Sample_T4C_k141_100062	46623	37	0	28	Low-quality	26.71
Sample_C10A_k141_214316	9905	11	2	0	Low-quality	26.71
Sample_T17B_k141_33551	11866	23	2	1	Low-quality	26.71
Sample_T7C_k141_23480	10826	16	9	0	Low-quality	26.71
Sample_T7B_k141_28374	10251	12	5	1	Low-quality	26.7
Sample_C11C_k141_40171	14288	20	5	2	Low-quality	26.69
Sample_C2C_k141_244458	11297	14	1	1	Low-quality	26.68
Sample_C3A_k141_234415	7380	3	0	3	Low-quality	26.68
Sample_T5A_k141_7337	54386	52	1	33	Low-quality	26.68
Sample_C13B_k141_55231	10842	12	6	0	Low-quality	26.66
Sample_T2C_k141_5729	14504	19	1	0	Low-quality	26.66
Sample_C13C_k141_35439	27268	33	2	7	Low-quality	26.64
Sample_C2B_k141_214522	17608	24	1	5	Low-quality	26.64
Sample_C6B_k141_190792	11196	13	0	11	Low-quality	26.64
Sample_T15C_k141_30428	21364	30	12	0	Low-quality	26.64
Sample_C3C_k141_182647	14995	15	6	1	Low-quality	26.63
Sample_C8A_k141_59965	13011	14	0	2	Low-quality	26.62
Sample_T15A_k141_65717	26788	24	0	5	Low-quality	26.62
Sample_T7B_k141_80253	41039	50	3	7	Low-quality	26.61
Sample_C5A_k141_27125	14591	7	0	6	Low-quality	26.6
Sample_T2C_k141_197058	145582	120	1	80	Low-quality	26.59
Sample_T6B_k141_109202	24695	26	0	2	Low-quality	26.59
Sample_C10A_k141_269580_fragment_2	10992	23	6	0	Low-quality	26.59
Sample_C5B_k141_7132_fragment_1	12642	13	1	2	Low-quality	26.59
Sample_T12A_k141_297701_fragment_1	11417	20	5	0	Low-quality	26.59
Sample_C4A_k141_152345	9752	20	1	0	Low-quality	26.57
Sample_C12B_k141_167400	10742	12	1	2	Low-quality	26.56
Sample_T6B_k141_189662	9766	21	1	0	Low-quality	26.55

Sample_T14A_k141_84769	16480	20	16	1	Low-quality	26.54
Sample_C3A_k141_117008	11059	12	3	0	Low-quality	26.53
Sample_C4A_k141_94500	19808	23	16	4	Low-quality	26.53
Sample_C11A_k141_84385	7950	9	2	0	Low-quality	26.52
Sample_C10A_k141_210143	9831	16	5	0	Low-quality	26.51
Sample_T6C_k141_50566	11089	9	1	0	Low-quality	26.5
Sample_T11B_k141_49065	26712	21	1	13	Low-quality	26.49
Sample_C4C_k141_85035	46361	48	1	27	Low-quality	26.48
Sample_T10A_k141_39835	10384	15	8	0	Low-quality	26.48
Sample_T10B_k141_122901	11413	26	6	0	Low-quality	26.48
Sample_T14C_k141_123892	43831	42	4	2	Low-quality	26.48
Sample_T2B_k141_232907_fragment_2	21830	30	5	4	Low-quality	26.48
Sample_T3A_k141_63275	41940	32	1	24	Low-quality	26.46
Sample_C14B_k141_26453_fragment_4	18408	17	3	2	Low-quality	26.46
Sample_T5C_k141_98703_fragment_1	21617	23	2	4	Low-quality	26.45
Sample_C1C_k141_234	8785	12	6	0	Low-quality	26.44
Sample_C1C_k141_161391	12407	14	10	0	Low-quality	26.43
Sample_C4A_k141_241454	10851	17	0	1	Low-quality	26.43
Sample_T11A_k141_48888	11577	13	1	1	Low-quality	26.43
Sample_T10B_k141_100629	12061	14	2	0	Low-quality	26.42
Sample_C12C_k141_92138	12505	14	7	0	Low-quality	26.42
Sample_T8B_k141_137002	20570	25	8	1	Low-quality	26.42
Sample_C8C_k141_36563	10123	4	1	0	Low-quality	26.41
Sample_T12B_k141_119183_fragment_1	21176	27	3	2	Low-quality	26.41
Sample_T4B_k141_86374	9576	23	3	0	Low-quality	26.41
Sample_T4B_k141_342991	9945	6	1	0	Low-quality	26.41
Sample_T6C_k141_125274	9123	14	13	0	Low-quality	26.4
Sample_C2C_k141_50169	276498	258	4	184	Low-quality	26.39
Sample_C10C_k141_157624	10049	10	3	0	Low-quality	26.39
Sample_C5A_k141_168826_fragment_1	20344	34	5	5	Low-quality	26.38
Sample_T3A_k141_46747	10728	12	7	0	Low-quality	26.38
Sample_C9A_k141_16393	20606	17	0	4	Low-quality	26.37
Sample_T16C_k141_67155	66702	66	1	6	Low-quality	26.36
Sample_T14C_k141_12302	9341	13	10	0	Low-quality	26.36
Sample_T11A_k141_1418	57246	53	0	8	Low-quality	26.35
Sample_T11C_k141_120272	17215	18	2	1	Low-quality	26.35
Sample_T7B_k141_109345	11927	25	8	0	Low-quality	26.34
Sample_T8C_k141_23092	23662	22	11	0	Low-quality	26.29
Sample_C8C_k141_46147	14106	25	9	1	Low-quality	26.28

Sample_T8A_k141_31504	10462	13	0	2	Low-quality	26.26
Sample_T8B_k141_94391	17199	32	1	3	Low-quality	26.25
Sample_C4C_k141_258994	21201	30	1	5	Low-quality	26.24
Sample_T6A_k141_65463	11268	11	1	6	Low-quality	26.23
Sample_T8B_k141_20477	15405	14	0	2	Low-quality	26.23
Sample_C14B_k141_70289	49966	103	10	3	Low-quality	26.23
Sample_T4B_k141_249380	11646	29	5	0	Low-quality	26.22
Sample_C5B_k141_29431	18868	29	8	0	Low-quality	26.21
Sample_C14C_k141_27888	11852	18	6	0	Low-quality	26.2
Sample_T6A_k141_220350	11271	12	1	0	Low-quality	26.2
Sample_T6A_k141_49360	32622	50	3	0	Low-quality	26.19
Sample_T14C_k141_161844	29902	28	1	9	Low-quality	26.17
Sample_T4A_k141_90580	16722	20	0	3	Low-quality	26.16
Sample_T10C_k141_20171	16481	14	1	0	Low-quality	26.16
Sample_C12C_k141_76766	6237	8	2	3	Low-quality	26.15
Sample_T13A_k141_99107	10986	20	0	20	Low-quality	26.14
Sample_T15C_k141_989	30921	27	1	5	Low-quality	26.14
Sample_C1C_k141_59692	17277	20	1	3	Low-quality	26.14
Sample_T8B_k141_88378	14655	20	7	0	Low-quality	26.14
Sample_T14A_k141_239031	53985	52	3	6	Low-quality	26.12
Sample_C11B_k141_209669	15213	12	1	6	Low-quality	26.11
Sample_T6B_k141_180533	14395	25	4	0	Low-quality	26.11
Sample_C12A_k141_122909	10993	10	8	0	Low-quality	26.1
Sample_C1C_k141_192900	12259	17	10	0	Low-quality	26.1
Sample_C4A_k141_92192	27734	36	8	2	Low-quality	26.1
Sample_C2A_k141_280836	9195	14	8	0	Low-quality	26.08
Sample_T5B_k141_11336	35516	31	1	7	Low-quality	26.06
Sample_C3C_k141_44700	15004	29	3	2	Low-quality	26.05
Sample_T7C_k141_104954_fragment_1	63530	76	10	4	Low-quality	26.05
Sample_C6B_k141_116841	9913	6	0	6	Low-quality	26.03
Sample_T16B_k141_136039	11087	18	8	0	Low-quality	26.02
Sample_C11A_k141_134573	61176	63	2	7	Low-quality	26.01
Sample_C14C_k141_38734	9831	7	0	0	Low-quality	26.01
Sample_T3A_k141_35243	15197	23	5	1	Low-quality	26.01
Sample_C5C_k141_27539	12200	17	12	0	Low-quality	26
Sample_T8A_k141_26156	15551	19	0	9	Low-quality	25.97
Sample_C10C_k141_213679	31208	54	3	0	Low-quality	25.96
Sample_T7B_k141_62226	11090	18	3	1	Low-quality	25.96
Sample_C2C_k141_243359	14907	16	1	4	Low-quality	25.95

Sample_C6B_k141_52293	28394	29	1	23	Low-quality	25.94
Sample_C10C_k141_126406	13799	26	5	0	Low-quality	25.94
Sample_T12A_k141_337327	10133	15	5	0	Low-quality	25.94
Sample_C13B_k141_73976_fragment_1	16301	23	2	0	Low-quality	25.93
Sample_T8C_k141_188786	28719	40	4	2	Low-quality	25.93
Sample_C4B_k141_150063	15090	19	0	6	Low-quality	25.92
Sample_T10A_k141_47988	40062	36	1	31	Low-quality	25.92
Sample_C5A_k141_140618	24585	18	2	4	Low-quality	25.91
Sample_C8C_k141_113520	14154	18	0	2	Low-quality	25.91
Sample_T5C_k141_27210	12411	16	10	0	Low-quality	25.91
Sample_C12B_k141_80930	9092	14	7	0	Low-quality	25.9
Sample_T5C_k141_157482	11091	25	8	0	Low-quality	25.89
Sample_T7A_k141_144511	10590	21	1	1	Low-quality	25.88
Sample_C11C_k141_87920	37047	30	4	10	Low-quality	25.87
Sample_C2A_k141_279319	20852	48	7	1	Low-quality	25.86
Sample_T11B_k141_58988	57591	43	7	20	Low-quality	25.85
Sample_C11B_k141_6425	15081	21	9	3	Low-quality	25.85
Sample_T18A_k141_118052	13842	16	5	1	Low-quality	25.84
Sample_C3C_k141_203157	12254	23	6	0	Low-quality	25.83
Sample_C2A_k141_80932	15459	16	0	2	Low-quality	25.82
Sample_T18A_k141_85351	15698	14	2	1	Low-quality	25.82
Sample_T14A_k141_12817	41596	44	2	9	Low-quality	25.79
Sample_C4B_k141_195552_fragment_1	25991	32	8	4	Low-quality	25.79
Sample_C10C_k141_127284	17038	21	1	2	Low-quality	25.78
Sample_C12C_k141_145033	10337	14	2	1	Low-quality	25.78
Sample_T13A_k141_88960	12737	24	4	0	Low-quality	25.78
Sample_C8A_k141_20382	14088	22	11	1	Low-quality	25.76
Sample_C8C_k141_60990	5374	4	1	0	Low-quality	25.76
Sample_T2C_k141_161037	9726	14	8	0	Low-quality	25.76
Sample_T18B_k141_51568	9253	12	1	0	Low-quality	25.73
Sample_T14B_k141_156080_fragment_1	10774	16	3	1	Low-quality	25.71
Sample_T15C_k141_120604	9397	13	0	0	Low-quality	25.71
Sample_C9A_k141_55162	30038	26	4	5	Low-quality	25.7
Sample_C11A_k141_8432	108858	98	2	61	Low-quality	25.69
Sample_T12C_k141_58697	10470	23	3	0	Low-quality	25.69
Sample_C11B_k141_228348	33498	37	1	8	Low-quality	25.67
Sample_C12A_k141_18910	71604	59	1	37	Low-quality	25.66
Sample_C1A_k141_107818	9882	10	5	0	Low-quality	25.66
Sample_T15C_k141_138314	9268	16	1	2	Low-quality	25.66

Sample_T4C_k141_60323_fragment_1	23092	21	4	2	Low-quality	25.66
Sample_T13B_k141_95246	12996	28	3	0	Low-quality	25.65
Sample_C14C_k141_105154	9626	18	5	0	Low-quality	25.64
Sample_T8B_k141_196865_fragment_1	13858	23	2	2	Low-quality	25.64
Sample_C4B_k141_54187	63540	51	1	34	Low-quality	25.61
Sample_T16B_k141_41250	16570	17	7	0	Low-quality	25.6
Sample_T8B_k141_45197	10951	11	0	0	Low-quality	25.6
Sample_T18B_k141_18162	11827	16	2	0	Low-quality	25.58
Sample_C9B_k141_91740	13964	20	6	0	Low-quality	25.57
Sample_T18B_k141_28488	28452	33	11	6	Low-quality	25.57
Sample_C1A_k141_159877	12455	14	5	0	Low-quality	25.55
Sample_C1B_k141_60798	11698	20	5	0	Low-quality	25.54
Sample_C6C_k141_162057	12357	15	8	2	Low-quality	25.54
Sample_C14B_k141_23308	10100	15	5	0	Low-quality	25.52
Sample_C3A_k141_255492	11234	14	5	0	Low-quality	25.52
Sample_C3C_k141_154984_fragment_2	27672	44	2	2	Low-quality	25.51
Sample_C6C_k141_88664	19966	18	0	5	Low-quality	25.5
Sample_T2B_k141_178995	31998	28	0	20	Low-quality	25.5
Sample_C8B_k141_86505	8017	8	0	1	Low-quality	25.5
Sample_T6C_k141_191203	11955	17	7	0	Low-quality	25.5
Sample_T14A_k141_154793_fragment_4	10565	10	4	0	Low-quality	25.48
Sample_C6B_k141_110283	33674	30	1	22	Low-quality	25.45
Sample_C3C_k141_96137	19620	14	0	2	Low-quality	25.44
Sample_C2C_k141_128604	41611	68	9	4	Low-quality	25.44
Sample_T6A_k141_204546	28523	22	0	17	Low-quality	25.42
Sample_T16C_k141_93360	10241	19	7	0	Low-quality	25.4
Sample_T11B_k141_161759	63013	56	1	48	Low-quality	25.39
Sample_T14B_k141_82347	8987	13	7	0	Low-quality	25.39
Sample_T2B_k141_145922	19952	23	9	3	Low-quality	25.39
Sample_T8C_k141_146607	9621	18	5	0	Low-quality	25.39
Sample_T6B_k141_143474	25251	20	0	17	Low-quality	25.37
Sample_T7A_k141_82121	58765	79	4	22	Low-quality	25.37
Sample_C6B_k141_50595	21956	16	1	5	Low-quality	25.36
Sample_C9C_k141_42428	16754	19	1	5	Low-quality	25.35
Sample_T10A_k141_176377	20987	19	1	5	Low-quality	25.32
Sample_C10A_k141_287659	21472	18	1	7	Low-quality	25.31
Sample_C11B_k141_109463	20447	27	1	7	Low-quality	25.31
Sample_C4A_k141_88851	138562	113	1	74	Low-quality	25.31
Sample_C4C_k141_223737	14765	16	1	2	Low-quality	25.31

Sample_T8A_k141_26407	16363	22	2	2	Low-quality	25.31
Sample_C7B_k141_50216	9299	24	4	0	Low-quality	25.3
Sample_T10B_k141_23842	7259	9	3	0	Low-quality	25.3
Sample_C13B_k141_56327	19079	16	0	5	Low-quality	25.29
Sample_C2A_k141_101638	36084	34	0	22	Low-quality	25.29
Sample_C10C_k141_214592	12719	14	0	1	Low-quality	25.29
Sample_C5A_k141_79390	16710	24	1	3	Low-quality	25.28
Sample_T8C_k141_55123	20062	15	0	2	Low-quality	25.28
Sample_C3A_k141_146939	25372	45	6	0	Low-quality	25.26
Sample_C8A_k141_18719	62631	52	1	38	Low-quality	25.24
Sample_C8B_k141_186599_fragment_2	28224	38	6	7	Low-quality	25.2
Sample_T3A_k141_72019	8375	10	0	9	Low-quality	25.19
Sample_T3A_k141_37393	9337	17	1	1	Low-quality	25.19
Sample_C10A_k141_53978	15340	18	6	0	Low-quality	25.19
Sample_T14A_k141_215204	8978	20	6	0	Low-quality	25.19
Sample_T16C_k141_18593	22740	19	1	8	Low-quality	25.18
Sample_C13C_k141_25094	11840	14	9	0	Low-quality	25.18
Sample_T5B_k141_67344	9440	17	4	0	Low-quality	25.18
Sample_C8A_k141_1266	10396	18	3	0	Low-quality	25.16
Sample_T7B_k141_23270	70161	61	1	34	Low-quality	25.14
Sample_T8C_k141_154718_fragment_1	7941	9	8	0	Low-quality	25.13
Sample_T12A_k141_55547	9895	9	2	0	Low-quality	25.12
Sample_T3C_k141_45926_fragment_2	10379	20	4	1	Low-quality	25.1
Sample_T12A_k141_120080	11987	19	3	1	Low-quality	25.09
Sample_T17C_k141_38420	10715	13	5	0	Low-quality	25.08
Sample_C11A_k141_45742	49114	45	0	33	Low-quality	25.07
Sample_T14A_k141_220290	19028	13	0	5	Low-quality	25.06
Sample_T7C_k141_11795	16565	15	1	8	Low-quality	25.06
Sample_T2A_k141_271540	10103	13	7	0	Low-quality	25.05
Sample_C2B_k141_5699	11237	10	4	0	Low-quality	25.04
Sample_T6A_k141_183610	22528	19	0	8	Low-quality	25.03
Sample_T12B_k141_170448	21912	26	2	4	Low-quality	25.02
Sample_T15C_k141_90871	15702	19	1	4	Low-quality	25.02
Sample_C2B_k141_113706	39029	38	1	18	Low-quality	25.01
Sample_C3C_k141_114695	14387	20	3	0	Low-quality	25.01
Sample_C5C_k141_59079	11599	26	11	0	Low-quality	25.01
Sample_C11B_k141_54291	9289	24	5	0	Low-quality	25
Sample_C3A_k141_87546	9794	23	2	0	Low-quality	25
Sample_T17C_k141_15969	14941	15	0	1	Low-quality	24.96

Sample_C10B_k141_25061	10206	13	7	0	Low-quality	24.96
Sample_T15C_k141_135414	9123	7	0	0	Low-quality	24.96
Sample_T4C_k141_91539	10750	9	2	0	Low-quality	24.96
Sample_C2C_k141_96131	18664	7	0	2	Low-quality	24.95
Sample_T13B_k141_133788	10267	14	8	0	Low-quality	24.95
Sample_C12C_k141_237211	28272	20	1	10	Low-quality	24.94
Sample_C13B_k141_45089	10736	4	0	0	Low-quality	24.94
Sample_C1C_k141_40323	38532	38	1	17	Low-quality	24.94
Sample_T18B_k141_34395	24645	47	7	3	Low-quality	24.94
Sample_T5A_k141_46564	36075	27	0	24	Low-quality	24.93
Sample_C1A_k141_135759	10151	11	3	1	Low-quality	24.92
Sample_T6C_k141_209230	9048	15	4	0	Low-quality	24.92
Sample_T11B_k141_85385	28470	30	1	6	Low-quality	24.91
Sample_C9A_k141_117977	11671	17	11	0	Low-quality	24.91
Sample_T13B_k141_85198	9042	14	4	0	Low-quality	24.91
Sample_T8C_k141_202110_fragment_1	10826	17	3	0	Low-quality	24.91
Sample_T8C_k141_67711	37036	43	1	5	Low-quality	24.9
Sample_C5C_k141_75326	11195	14	0	0	Low-quality	24.9
Sample_C2A_k141_116800	136292	116	1	75	Low-quality	24.89
Sample_T15B_k141_30843_fragment_1	14515	14	4	3	Low-quality	24.88
Sample_T7C_k141_78624	11232	13	5	0	Low-quality	24.88
Sample_T2C_k141_142213	35959	32	0	20	Low-quality	24.87
Sample_T10A_k141_25941	33244	36	10	0	Low-quality	24.87
Sample_C4B_k141_241135	11165	25	1	1	Low-quality	24.86
Sample_T6A_k141_130759	20086	29	1	12	Low-quality	24.86
Sample_C10C_k141_84322	14928	30	2	1	Low-quality	24.85
Sample_C5A_k141_129327	22964	35	2	0	Low-quality	24.85
Sample_C11B_k141_201113	11917	19	12	0	Low-quality	24.84
Sample_C14C_k141_78558	20846	16	0	5	Low-quality	24.82
Sample_C12C_k141_217390_fragment_1	10038	16	2	1	Low-quality	24.82
Sample_C14A_k141_103275	23315	39	5	1	Low-quality	24.8
Sample_C13A_k141_87086	63366	71	2	20	Low-quality	24.79
Sample_T4B_k141_23292	26122	24	0	11	Low-quality	24.79
Sample_T2B_k141_237322	11763	20	10	0	Low-quality	24.79
Sample_C6C_k141_197657	40714	42	1	12	Low-quality	24.78
Sample_C12A_k141_200366	16021	31	7	0	Low-quality	24.78
Sample_T5A_k141_7842	9732	26	6	0	Low-quality	24.78
Sample_C12A_k141_174375	8250	12	7	0	Low-quality	24.77
Sample_T6A_k141_52699	10655	11	2	0	Low-quality	24.77

Sample_C7A_k141_97700	10545	21	3	0	Low-quality	24.76
Sample_T1A_k141_23052	18504	6	0	2	Low-quality	24.75
Sample_T6B_k141_39446	12667	14	1	2	Low-quality	24.75
Sample_C10A_k141_41135	18669	24	2	6	Low-quality	24.74
Sample_T13A_k141_98615	9190	15	1	0	Low-quality	24.74
Sample_C1B_k141_254705	19663	11	2	1	Low-quality	24.7
Sample_C3A_k141_217740	14237	37	3	1	Low-quality	24.7
Sample_T17C_k141_6096	10350	17	16	0	Low-quality	24.7
Sample_T6C_k141_68217	10144	16	8	0	Low-quality	24.69
Sample_T5C_k141_92585	16594	22	0	7	Low-quality	24.67
Sample_T7A_k141_29054	14762	14	0	10	Low-quality	24.66
Sample_C10C_k141_73421	25111	32	4	6	Low-quality	24.66
Sample_T15A_k141_106715	10134	7	0	2	Low-quality	24.64
Sample_C3B_k141_43596	18696	13	0	5	Low-quality	24.63
Sample_T15C_k141_32461	61088	62	1	39	Low-quality	24.62
Sample_T17B_k141_34417	13721	11	0	1	Low-quality	24.61
Sample_C10A_k141_288571	19253	16	1	2	Low-quality	24.6
Sample_T17C_k141_33028_fragment_1	12865	20	10	1	Low-quality	24.6
Sample_C4A_k141_94069_fragment_1	9493	8	4	0	Low-quality	24.57
Sample_C1B_k141_98968	21245	24	1	11	Low-quality	24.56
Sample_C6B_k141_131099	12908	18	12	0	Low-quality	24.55
Sample_C8C_k141_153098	8961	12	4	0	Low-quality	24.55
Sample_C10B_k141_106950	19584	20	10	2	Low-quality	24.52
Sample_C3C_k141_164261	15023	16	2	2	Low-quality	24.52
Sample_T13A_k141_4320	9402	17	1	1	Low-quality	24.52
Sample_C13A_k141_31472	23986	24	0	5	Low-quality	24.51
Sample_T4C_k141_111813	14737	13	0	2	Low-quality	24.51
Sample_T14A_k141_124001	40256	45	1	10	Low-quality	24.5
Sample_T6C_k141_63796	10507	17	5	1	Low-quality	24.5
Sample_C7C_k141_80336	46032	40	2	11	Low-quality	24.49
Sample_C2A_k141_282657	20794	33	5	1	Low-quality	24.49
Sample_T11A_k141_48527_fragment_4	16840	21	3	1	Low-quality	24.49
Sample_T8C_k141_42806	8725	15	7	0	Low-quality	24.49
Sample_T13B_k141_119878	36505	33	2	26	Low-quality	24.48
Sample_C3B_k141_213969	15268	17	4	1	Low-quality	24.48
Sample_C8C_k141_24556	9926	7	3	0	Low-quality	24.48
Sample_C13A_k141_111117	11682	15	0	5	Low-quality	24.46
Sample_C2C_k141_290245	28463	32	1	15	Low-quality	24.46
Sample_C2A_k141_4057	29933	35	4	1	Low-quality	24.46

Sample_T4B_k141_247596	37790	38	1	25	Low-quality	24.45
Sample_C5C_k141_93066_fragment_1	10157	16	3	0	Low-quality	24.45
Sample_T6B_k141_50043	10545	15	5	0	Low-quality	24.45
Sample_T5C_k141_146406	8537	13	8	0	Low-quality	24.44
Sample_C14A_k141_110528	10481	20	5	0	Low-quality	24.43
Sample_T2C_k141_226814	80592	82	7	39	Low-quality	24.42
Sample_C1C_k141_183219	10256	10	0	6	Low-quality	24.4
Sample_T6B_k141_110128	14599	20	0	5	Low-quality	24.38
Sample_C3A_k141_232275_fragment_1	37488	51	8	2	Low-quality	24.38
Sample_C5A_k141_126217	9312	11	8	0	Low-quality	24.38
Sample_T10C_k141_23120	24387	24	0	3	Low-quality	24.37
Sample_T6C_k141_109439	17960	21	0	6	Low-quality	24.37
Sample_T16C_k141_92492	9465	16	7	0	Low-quality	24.36
Sample_T7A_k141_213056	8554	20	0	0	Low-quality	24.36
Sample_T4B_k141_292440	46477	42	1	30	Low-quality	24.34
Sample_T3B_k141_58335	9521	13	1	2	Low-quality	24.33
Sample_C6B_k141_154638	16070	20	1	6	Low-quality	24.32
Sample_T2B_k141_87901	10170	8	4	0	Low-quality	24.32
Sample_C1C_k141_124580_fragment_1	9906	9	4	0	Low-quality	24.31
Sample_T7B_k141_144589	10856	23	2	0	Low-quality	24.31
Sample_C11A_k141_10526	42427	38	0	30	Low-quality	24.3
Sample_C11B_k141_81018	9548	21	3	0	Low-quality	24.29
Sample_C1C_k141_141901	9846	16	4	0	Low-quality	24.29
Sample_C5A_k141_130697	9415	15	4	0	Low-quality	24.29
Sample_T3C_k141_94625	16050	15	1	1	Low-quality	24.29
Sample_C13A_k141_14060	20013	21	1	14	Low-quality	24.28
Sample_C2C_k141_262039	18159	9	0	2	Low-quality	24.28
Sample_T12B_k141_142014	28781	28	1	5	Low-quality	24.28
Sample_T5B_k141_371	9485	13	0	3	Low-quality	24.28
Sample_C2A_k141_173647_fragment_1	9785	20	2	0	Low-quality	24.28
Sample_T13A_k141_2875	11360	14	8	0	Low-quality	24.28
Sample_T5B_k141_4906	17515	20	0	2	Low-quality	24.27
Sample_T8B_k141_206244	15723	11	0	9	Low-quality	24.27
Sample_T6C_k141_170178	15841	13	3	0	Low-quality	24.27
Sample_T1C_k141_43634	22443	23	0	16	Low-quality	24.26
Sample_C13C_k141_83342	7678	13	3	0	Low-quality	24.26
Sample_T15C_k141_76564	10047	24	7	0	Low-quality	24.26
Sample_T17B_k141_26937	11174	16	8	1	Low-quality	24.25
Sample_T2A_k141_9910	10313	11	0	1	Low-quality	24.24

Sample_T7A_k141_162448	13173	12	3	2	Low-quality	24.24
Sample_C4B_k141_60261	9085	12	6	0	Low-quality	24.22
Sample_C14C_k141_21941	11806	19	1	2	Low-quality	24.2
Sample_T7A_k141_208251	14929	26	5	0	Low-quality	24.2
Sample_C11B_k141_201056	11861	19	8	3	Low-quality	24.19
Sample_C5B_k141_64601_fragment_1	7753	8	7	0	Low-quality	24.19
Sample_T11A_k141_65941	9352	9	0	5	Low-quality	24.18
Sample_T2C_k141_111005	16836	28	1	5	Low-quality	24.18
Sample_T4B_k141_38732	9389	20	7	0	Low-quality	24.18
Sample_C3C_k141_119807	9484	19	2	0	Low-quality	24.17
Sample_T4B_k141_10474	13929	28	1	0	Low-quality	24.17
Sample_C8B_k141_166197	8920	23	2	0	Low-quality	24.16
Sample_T6C_k141_123235	8793	15	2	0	Low-quality	24.16
Sample_C12C_k141_223902	14946	21	5	2	Low-quality	24.15
Sample_C9A_k141_59353	9212	12	1	1	Low-quality	24.15
Sample_T10A_k141_4854	8942	10	3	0	Low-quality	24.15
Sample_T16B_k141_144914	38810	56	10	2	Low-quality	24.15
Sample_T12B_k141_4075	26471	24	0	3	Low-quality	24.14
Sample_T6C_k141_139394	15633	12	9	0	Low-quality	24.14
Sample_T4C_k141_117837	40121	39	2	8	Low-quality	24.13
Sample_T12B_k141_44717	9505	16	4	1	Low-quality	24.13
Sample_T17B_k141_16200	10171	14	9	0	Low-quality	24.11
Sample_C13B_k141_23636	9421	17	4	0	Low-quality	24.1
Sample_C14A_k141_225704_fragment_1	10365	13	1	2	Low-quality	24.1
Sample_C13B_k141_68124	7953	9	0	0	Low-quality	24.09
Sample_C4B_k141_112513_fragment_1	9190	14	3	0	Low-quality	24.09
Sample_T5C_k141_45512	46188	42	2	21	Low-quality	24.08
Sample_T3C_k141_88612	9924	23	6	0	Low-quality	24.08
Sample_C2C_k141_253791	25666	18	1	12	Low-quality	24.07
Sample_T10A_k141_3187	11210	21	3	0	Low-quality	24.07
Sample_C7A_k141_94468	6898	9	2	0	Low-quality	24.05
Sample_C7A_k141_33774	18392	14	0	3	Low-quality	24.05
Sample_T16B_k141_49840	25799	18	0	3	Low-quality	24.05
Sample_T2C_k141_81542	17328	18	0	6	Low-quality	24.05
Sample_C6C_k141_150147	12499	17	1	4	Low-quality	24.04
Sample_C4A_k141_231650	17730	20	4	1	Low-quality	24.04
Sample_T10C_k141_55254	16310	34	3	1	Low-quality	24.04
Sample_C12A_k141_149268	16146	18	0	3	Low-quality	24.03
Sample_T13C_k141_78497	32657	37	6	0	Low-quality	24.02

Sample_C10C_k141_95509	10617	13	1	0	Low-quality	24.02
Sample_T10A_k141_205	12741	15	1	1	Low-quality	24.01
Sample_T17A_k141_3912	15864	20	1	2	Low-quality	24
Sample_T8A_k141_155087_fragment_1	9828	10	3	0	Low-quality	24
Sample_T12B_k141_44763	13978	27	4	0	Low-quality	23.99
Sample_T16C_k141_7992	15851	21	1	5	Low-quality	23.98
Sample_T10A_k141_53273	11147	13	9	0	Low-quality	23.98
Sample_C11B_k141_16436	10173	10	0	1	Low-quality	23.97
Sample_T15A_k141_18001	8995	14	0	6	Low-quality	23.97
Sample_T11A_k141_139243	9335	15	2	0	Low-quality	23.96
Sample_T14A_k141_76832	45572	44	1	29	Low-quality	23.94
Sample_C10C_k141_123123	14665	18	1	3	Low-quality	23.93
Sample_T12C_k141_105561	7802	16	2	0	Low-quality	23.93
Sample_C14A_k141_220784	15809	13	1	5	Low-quality	23.92
Sample_C4A_k141_189478	11900	12	1	5	Low-quality	23.92
Sample_C9C_k141_2258	21310	23	1	6	Low-quality	23.92
Sample_C11C_k141_19210	11264	8	1	0	Low-quality	23.92
Sample_T7A_k141_4801	21395	29	2	5	Low-quality	23.9
Sample_T11C_k141_118428	10163	7	0	2	Low-quality	23.9
Sample_T4B_k141_190735	24845	25	0	12	Low-quality	23.89
Sample_T17B_k141_20977	22043	15	0	3	Low-quality	23.88
Sample_C2B_k141_122807	11462	21	9	0	Low-quality	23.86
Sample_C4B_k141_99394	8794	6	2	0	Low-quality	23.86
Sample_T7B_k141_72147	9103	19	4	0	Low-quality	23.86
Sample_T4C_k141_76392	20612	20	2	4	Low-quality	23.85
Sample_T8C_k141_203583	19327	15	0	4	Low-quality	23.85
Sample_C1A_k141_20533	9888	14	3	0	Low-quality	23.85
Sample_T2B_k141_206696	9328	11	8	0	Low-quality	23.85
Sample_T8A_k141_192110	16027	10	0	2	Low-quality	23.84
Sample_T4A_k141_48877	11055	16	0	2	Low-quality	23.84
Sample_C8C_k141_54616	8375	9	7	0	Low-quality	23.82
Sample_T14B_k141_168869	59025	26	1	16	Low-quality	23.79
Sample_C14C_k141_126679	9565	10	0	2	Low-quality	23.78
Sample_C11B_k141_77175	9380	16	1	2	Low-quality	23.77
Sample_T12B_k141_181825	45813	96	12	1	Low-quality	23.77
Sample_T14C_k141_132100	15014	24	2	0	Low-quality	23.77
Sample_T4B_k141_230094	8937	9	2	0	Low-quality	23.77
Sample_T7A_k141_128290	11894	20	3	0	Low-quality	23.77
Sample_T2C_k141_59061	20080	24	0	11	Low-quality	23.76

Sample_C5A_k141_224370	14086	29	8	1	Low-quality	23.76
Sample_T13B_k141_92491	14073	20	1	2	Low-quality	23.75
Sample_C12C_k141_238066	20019	33	9	2	Low-quality	23.75
Sample_T13C_k141_108337_fragment_1	11723	14	10	0	Low-quality	23.75
Sample_C11A_k141_29935	21364	22	0	7	Low-quality	23.74
Sample_T7B_k141_65193	10876	10	0	1	Low-quality	23.74
Sample_T11C_k141_8678	12152	16	2	1	Low-quality	23.73
Sample_C6A_k141_13032	8673	12	6	0	Low-quality	23.72
Sample_T10B_k141_172360_fragment_1	9243	12	4	0	Low-quality	23.72
Sample_T14A_k141_51022	22456	20	0	7	Low-quality	23.71
Sample_C8A_k141_92547	14547	17	8	0	Low-quality	23.71
Sample_T10B_k141_40003	31589	37	2	0	Low-quality	23.7
Sample_C10C_k141_75530	18218	17	1	3	Low-quality	23.69
Sample_T8A_k141_109352_fragment_1	9029	20	3	0	Low-quality	23.68
Sample_C8A_k141_157492	11183	21	3	0	Low-quality	23.66
Sample_C11A_k141_120182	17486	35	8	2	Low-quality	23.65
Sample_C9C_k141_32853	10474	21	7	0	Low-quality	23.64
Sample_C4C_k141_144954	14150	17	0	4	Low-quality	23.63
Sample_C11A_k141_53125	8924	12	5	0	Low-quality	23.62
Sample_T10B_k141_167177	13705	13	1	2	Low-quality	23.61
Sample_C13B_k141_102046	9617	14	7	1	Low-quality	23.6
Sample_T10B_k141_122308	11120	12	2	0	Low-quality	23.6
Sample_T12C_k141_93177	18059	18	2	3	Low-quality	23.6
Sample_C5A_k141_20447	32723	39	1	9	Low-quality	23.58
Sample_T6A_k141_166589	9617	16	6	0	Low-quality	23.58
Sample_C14C_k141_14505	9663	17	8	2	Low-quality	23.57
Sample_T13B_k141_45212	14198	22	1	0	Low-quality	23.57
Sample_T18A_k141_98902	8438	16	1	1	Low-quality	23.57
Sample_T5A_k141_43371_fragment_1	9182	18	5	0	Low-quality	23.57
Sample_C1C_k141_126649	12143	18	13	0	Low-quality	23.56
Sample_T10A_k141_46018	9337	20	2	0	Low-quality	23.56
Sample_C10B_k141_158271	9225	10	0	8	Low-quality	23.55
Sample_T6B_k141_51167	16786	21	1	4	Low-quality	23.53
Sample_C3A_k141_150699	6838	11	4	0	Low-quality	23.51
Sample_T13A_k141_60249	10643	16	8	0	Low-quality	23.51
Sample_T17A_k141_52428	8212	15	4	0	Low-quality	23.5
Sample_C14B_k141_72513	9890	10	6	0	Low-quality	23.49
Sample_C2B_k141_252134	19332	19	0	3	Low-quality	23.48
Sample_C11A_k141_173893	12653	14	7	0	Low-quality	23.45

Sample_T18A_k141_176449	10009	17	3	0	Low-quality	23.45
Sample_C2C_k141_88414	15491	16	1	2	Low-quality	23.44
Sample_C8B_k141_182000	9257	19	0	1	Low-quality	23.44
Sample_T8B_k141_179668_fragment_1	9608	10	3	0	Low-quality	23.43
Sample_C5A_k141_147065	16621	14	0	4	Low-quality	23.42
Sample_C2A_k141_319829	9378	18	5	0	Low-quality	23.42
Sample_T15A_k141_140109_fragment_1	41601	55	10	2	Low-quality	23.42
Sample_T5A_k141_54008	9482	7	5	0	Low-quality	23.42
Sample_C11C_k141_84012	46337	44	1	19	Low-quality	23.41
Sample_T6A_k141_16033	15926	15	0	5	Low-quality	23.41
Sample_C9A_k141_43514	39451	27	6	0	Low-quality	23.41
Sample_C2A_k141_137381	28854	20	1	6	Low-quality	23.4
Sample_T5B_k141_17471	10869	10	0	3	Low-quality	23.38
Sample_T10A_k141_124970	38936	47	2	2	Low-quality	23.37
Sample_C8C_k141_32162	11853	11	0	1	Low-quality	23.36
Sample_C9C_k141_53250	23483	17	0	12	Low-quality	23.36
Sample_C3C_k141_126824	9522	13	1	1	Low-quality	23.36
Sample_C6A_k141_190316	12371	18	12	0	Low-quality	23.36
Sample_T2B_k141_251694	15731	12	2	0	Low-quality	23.35
Sample_C8C_k141_151094	9970	10	0	0	Low-quality	23.34
Sample_T16A_k141_125627	28718	41	3	2	Low-quality	23.34
Sample_T6B_k141_57833	8667	7	4	0	Low-quality	23.34
Sample_T16B_k141_25441	13867	25	4	0	Low-quality	23.32
Sample_T6C_k141_78319_fragment_1	9706	13	1	1	Low-quality	23.32
Sample_C3B_k141_1182	10827	10	0	3	Low-quality	23.29
Sample_T10B_k141_4296	20801	16	1	4	Low-quality	23.29
Sample_T5C_k141_1271	16965	18	0	6	Low-quality	23.29
Sample_C1B_k141_59493	10535	18	4	0	Low-quality	23.29
Sample_T12C_k141_6569	8812	12	5	0	Low-quality	23.29
Sample_C2C_k141_98747	53646	48	1	24	Low-quality	23.27
Sample_C1B_k141_118886	13738	14	0	7	Low-quality	23.26
Sample_T17A_k141_45683_fragment_1	13844	20	10	2	Low-quality	23.26
Sample_C6B_k141_183114_fragment_1	9961	10	0	1	Low-quality	23.25
Sample_T8A_k141_224961	13527	22	4	0	Low-quality	23.22
Sample_C5A_k141_95792	9972	10	3	0	Low-quality	23.21
Sample_C8A_k141_117194	12246	23	1	2	Low-quality	23.21
Sample_C8B_k141_161647	14980	36	8	0	Low-quality	23.21
Sample_T16A_k141_49509	14315	39	4	0	Low-quality	23.19
Sample_T5C_k141_79870	10354	14	4	0	Low-quality	23.19

Sample_C6B_k141_156242	36022	32	1	27	Low-quality	23.18
Sample_T6C_k141_33949	13877	16	0	3	Low-quality	23.18
Sample_C12C_k141_48406	9790	13	7	0	Low-quality	23.18
Sample_T8A_k141_256708	47367	73	13	2	Low-quality	23.18
Sample_T4B_k141_17270	8187	14	9	0	Low-quality	23.16
Sample_C11C_k141_165700	7741	12	5	0	Low-quality	23.15
Sample_C5A_k141_141134	14634	12	2	0	Low-quality	23.15
Sample_T10B_k141_42653	9667	18	8	0	Low-quality	23.15
Sample_T8C_k141_160679	11776	13	4	0	Low-quality	23.15
Sample_C1C_k141_105606	47163	41	1	10	Low-quality	23.13
Sample_C4B_k141_156377	9504	6	4	0	Low-quality	23.12
Sample_T17B_k141_33254	24458	26	6	2	Low-quality	23.12
Sample_C3A_k141_226283	10660	15	10	0	Low-quality	23.11
Sample_C4A_k141_199687	9401	9	3	0	Low-quality	23.11
Sample_T11C_k141_7804	14597	15	0	6	Low-quality	23.1
Sample_T4C_k141_3687	9498	11	1	4	Low-quality	23.1
Sample_C11A_k141_96515	36484	36	3	0	Low-quality	23.1
Sample_C3A_k141_95543_fragment_1	10296	13	10	1	Low-quality	23.1
Sample_C4A_k141_69165	12350	19	11	1	Low-quality	23.1
Sample_T14A_k141_50335	13822	16	0	3	Low-quality	23.09
Sample_T11A_k141_98941	275330	282	1	212	Low-quality	23.08
Sample_C3A_k141_195486	8367	17	1	0	Low-quality	23.08
Sample_T10A_k141_2843	12998	10	3	0	Low-quality	23.08
Sample_T10A_k141_179633	8888	6	2	0	Low-quality	23.08
Sample_T16C_k141_48782	12373	11	1	0	Low-quality	23.07
Sample_C14A_k141_164867	14314	14	2	0	Low-quality	23.07
Sample_C9A_k141_126520	11266	11	5	1	Low-quality	23.07
Sample_C12C_k141_135509	7798	13	8	0	Low-quality	23.06
Sample_C2B_k141_200385	9388	18	3	0	Low-quality	23.06
Sample_T2A_k141_14242	5969	5	0	0	Low-quality	23.04
Sample_T8A_k141_204989	8924	11	0	1	Low-quality	23.04
Sample_C11C_k141_154890	8234	12	6	0	Low-quality	23.04
Sample_T7C_k141_97846	56193	64	10	1	Low-quality	23.04
Sample_T16C_k141_23808	35791	27	1	22	Low-quality	23.03
Sample_C14A_k141_92597_fragment_2	15276	18	2	0	Low-quality	23.03
Sample_T16B_k141_56482	8555	15	10	0	Low-quality	23.03
Sample_C9C_k141_80169	14816	11	0	2	Low-quality	23.02
Sample_T4B_k141_122926	40699	91	3	1	Low-quality	23.02
Sample_T16C_k141_28905	33925	35	12	6	Low-quality	23.02

Sample_T6B_k141_71889	10661	9	9	0	Low-quality	23.02
Sample_T3A_k141_57830	13779	17	0	1	Low-quality	23.01
Sample_T8C_k141_57582	10341	10	8	0	Low-quality	23.01
Sample_T11C_k141_105027	15197	20	1	6	Low-quality	23
Sample_T15B_k141_130646	18779	15	3	1	Low-quality	22.99
Sample_T16B_k141_30584	9845	15	7	0	Low-quality	22.99
Sample_T2C_k141_253752	9928	25	3	1	Low-quality	22.99
Sample_T7C_k141_32244	9761	18	3	0	Low-quality	22.98
Sample_T14B_k141_35831	10451	14	6	0	Low-quality	22.97
Sample_T13B_k141_53802_fragment_1	9135	14	1	1	Low-quality	22.96
Sample_T12A_k141_95750	9342	15	2	0	Low-quality	22.94
Sample_T12C_k141_161260	7873	11	0	4	Low-quality	22.93
Sample_C12B_k141_186845	8437	17	4	0	Low-quality	22.93
Sample_T12A_k141_281706	8876	14	2	0	Low-quality	22.93
Sample_T8C_k141_211496_fragment_1	7858	8	0	1	Low-quality	22.92
Sample_T18B_k141_80737	12778	12	0	3	Low-quality	22.91
Sample_T7A_k141_192994	8230	9	3	1	Low-quality	22.91
Sample_C12C_k141_49495	18714	16	0	1	Low-quality	22.9
Sample_C4B_k141_230291	44606	49	4	1	Low-quality	22.9
Sample_T7A_k141_28165	13300	22	5	0	Low-quality	22.9
Sample_T18C_k141_127291	13705	20	4	0	Low-quality	22.89
Sample_T16B_k141_15107	41761	50	11	5	Low-quality	22.88
Sample_T4A_k141_102235	13509	16	8	0	Low-quality	22.88
Sample_C5A_k141_234014	9845	7	0	2	Low-quality	22.87
Sample_C9B_k141_74535	13802	26	5	0	Low-quality	22.87
Sample_C14C_k141_78657_fragment_1	36243	37	1	6	Low-quality	22.87
Sample_C6C_k141_18272	11240	11	6	1	Low-quality	22.87
Sample_C10C_k141_188729	8035	12	8	0	Low-quality	22.86
Sample_T4B_k141_304404	17240	21	2	2	Low-quality	22.85
Sample_C10A_k141_288906	18995	31	4	2	Low-quality	22.85
Sample_C10C_k141_230027	8503	11	4	0	Low-quality	22.85
Sample_C3A_k141_201146	125045	103	1	73	Low-quality	22.84
Sample_C5C_k141_44814	88409	74	1	53	Low-quality	22.84
Sample_T8C_k141_69146	7232	10	0	0	Low-quality	22.83
Sample_C4C_k141_241655	88300	76	1	55	Low-quality	22.82
Sample_T1A_k141_5743	38935	36	0	24	Low-quality	22.82
Sample_C11B_k141_231186_fragment_1	7838	8	6	0	Low-quality	22.82
Sample_T11B_k141_8763	8425	21	0	0	Low-quality	22.82
Sample_T2B_k141_226643	13762	14	11	0	Low-quality	22.82

Sample_T3A_k141_97713	7406	16	1	0	Low-quality	22.82
Sample_T8B_k141_51466	11957	15	7	0	Low-quality	22.82
Sample_T10B_k141_178576	9905	7	0	2	Low-quality	22.81
Sample_C8C_k141_23976	10236	19	2	0	Low-quality	22.81
Sample_T3C_k141_10084	12233	15	3	0	Low-quality	22.8
Sample_T10A_k141_50545	85830	86	3	41	Low-quality	22.79
Sample_T15B_k141_19859_fragment_1	11600	18	0	1	Low-quality	22.79
Sample_C8B_k141_115069	18993	20	2	9	Low-quality	22.78
Sample_C3A_k141_5400	10645	15	6	0	Low-quality	22.78
Sample_T16A_k141_54526	7817	5	0	3	Low-quality	22.77
Sample_C11B_k141_194507	8105	9	6	0	Low-quality	22.77
Sample_C8A_k141_167522	9635	12	5	0	Low-quality	22.77
Sample_C12A_k141_50887	56468	49	1	25	Low-quality	22.76
Sample_C12B_k141_153211	9642	15	5	0	Low-quality	22.76
Sample_T10A_k141_175141	11448	17	0	0	Low-quality	22.76
Sample_T6B_k141_92811	8886	14	9	0	Low-quality	22.76
Sample_C6A_k141_159012	12161	16	8	3	Low-quality	22.75
Sample_T16B_k141_52118	9426	8	1	0	Low-quality	22.75
Sample_T5A_k141_51491	12685	7	0	2	Low-quality	22.75
Sample_T12A_k141_333313	36780	40	1	21	Low-quality	22.73
Sample_T11A_k141_122079	26060	29	1	7	Low-quality	22.72
Sample_T14A_k141_142149	10562	10	0	1	Low-quality	22.72
Sample_T18A_k141_36466	8718	16	1	1	Low-quality	22.72
Sample_T6C_k141_57061	19724	12	0	3	Low-quality	22.72
Sample_T15A_k141_60725_fragment_1	11392	16	3	0	Low-quality	22.72
Sample_T11A_k141_136396	10651	13	7	1	Low-quality	22.71
Sample_T4A_k141_12318	8778	12	1	1	Low-quality	22.7
Sample_C5A_k141_166414	17340	15	0	3	Low-quality	22.69
Sample_T13C_k141_37340	18705	33	2	2	Low-quality	22.68
Sample_C13A_k141_73907	15998	18	1	10	Low-quality	22.67
Sample_C7B_k141_85395	18521	21	0	5	Low-quality	22.66
Sample_T10A_k141_147813	22601	19	0	16	Low-quality	22.66
Sample_T16C_k141_143315	9591	8	0	5	Low-quality	22.66
Sample_C8C_k141_60821	9384	13	12	0	Low-quality	22.66
Sample_T12A_k141_9762	12630	11	1	3	Low-quality	22.65
Sample_C2B_k141_88083	13274	25	1	0	Low-quality	22.63
Sample_C12A_k141_150900	13535	16	0	2	Low-quality	22.61
Sample_T4C_k141_85998	13535	12	0	5	Low-quality	22.61
Sample_C11B_k141_232249	8449	7	5	0	Low-quality	22.61

Sample_T6C_k141_97232	6162	8	2	0	Low-quality	22.6
Sample_T13B_k141_3282_fragment_1	9171	13	2	0	Low-quality	22.6
Sample_C13A_k141_72156	8937	19	4	0	Low-quality	22.59
Sample_C4B_k141_83023	20717	19	0	14	Low-quality	22.59
Sample_T15B_k141_19670	32406	37	1	5	Low-quality	22.59
Sample_T18C_k141_44156	9671	21	1	2	Low-quality	22.58
Sample_C11B_k141_94070	7817	11	5	0	Low-quality	22.58
Sample_T2A_k141_250846	9587	15	0	0	Low-quality	22.58
Sample_T2C_k141_217659_fragment_1	9436	13	4	0	Low-quality	22.58
Sample_C1C_k141_129784	13513	15	0	2	Low-quality	22.57
Sample_C6A_k141_51510	18035	27	1	1	Low-quality	22.57
Sample_C7A_k141_14189	8608	13	8	0	Low-quality	22.57
Sample_T8C_k141_135067	9319	10	3	0	Low-quality	22.57
Sample_T11A_k141_66063	15474	17	1	4	Low-quality	22.56
Sample_T16B_k141_154777	13504	14	0	2	Low-quality	22.56
Sample_C10A_k141_19869	8969	11	5	0	Low-quality	22.56
Sample_T14A_k141_132048	9061	15	1	1	Low-quality	22.56
Sample_C12A_k141_78832	9013	18	1	0	Low-quality	22.55
Sample_C1A_k141_137590	10645	14	8	0	Low-quality	22.55
Sample_C3C_k141_72571	16143	28	2	2	Low-quality	22.54
Sample_C11A_k141_168819	13935	30	2	0	Low-quality	22.54
Sample_C6B_k141_45758_fragment_1	41984	64	16	2	Low-quality	22.54
Sample_T8B_k141_94691	9082	20	2	0	Low-quality	22.54
Sample_C13A_k141_29699	8995	10	0	1	Low-quality	22.53
Sample_C2C_k141_66984	24956	25	1	11	Low-quality	22.53
Sample_T12A_k141_329141	8472	14	0	0	Low-quality	22.53
Sample_T6C_k141_204473	8529	9	0	0	Low-quality	22.53
Sample_C13A_k141_85676	14881	19	1	4	Low-quality	22.52
Sample_T4C_k141_6992	6699	7	0	4	Low-quality	22.52
Sample_C6B_k141_66515	10456	9	0	5	Low-quality	22.51
Sample_T1A_k141_19962	35747	43	17	6	Low-quality	22.51
Sample_T6A_k141_84024	12078	17	11	2	Low-quality	22.51
Sample_C5C_k141_115311	248306	246	3	124	Low-quality	22.5
Sample_T4B_k141_228900	18935	19	0	5	Low-quality	22.48
Sample_T7A_k141_210356	14355	14	1	2	Low-quality	22.48
Sample_T10B_k141_75629	8062	8	4	0	Low-quality	22.47
Sample_C3A_k141_118062	10068	16	9	0	Low-quality	22.46
Sample_T2C_k141_275601	9849	16	4	0	Low-quality	22.46
Sample_T5C_k141_101057	8075	8	4	0	Low-quality	22.46

Sample_C12A_k141_202586	73286	59	3	11	Low-quality	22.45
Sample_T3A_k141_70760	21237	16	0	10	Low-quality	22.45
Sample_T8A_k141_233950	15842	16	1	8	Low-quality	22.45
Sample_T8B_k141_2592	14403	20	0	2	Low-quality	22.45
Sample_T15C_k141_105434	9059	13	0	2	Low-quality	22.43
Sample_C7A_k141_146756	11859	16	1	1	Low-quality	22.43
Sample_C6B_k141_216023	46103	103	10	2	Low-quality	22.42
Sample_C10A_k141_106633	6470	9	0	0	Low-quality	22.4
Sample_T1C_k141_20746	7860	7	5	0	Low-quality	22.4
Sample_T17C_k141_19340	10967	13	0	3	Low-quality	22.39
Sample_T6C_k141_53700	35488	31	1	17	Low-quality	22.39
Sample_C3A_k141_210872	11771	13	6	0	Low-quality	22.39
Sample_T4A_k141_12849	11018	11	6	1	Low-quality	22.39
Sample_T4B_k141_341039	8463	15	7	0	Low-quality	22.39
Sample_C2C_k141_311770	121937	119	1	75	Low-quality	22.38
Sample_T8B_k141_133603	22198	19	0	18	Low-quality	22.38
Sample_C7A_k141_118406	10172	14	5	0	Low-quality	22.38
Sample_C8B_k141_50663	11243	14	7	0	Low-quality	22.38
Sample_T11B_k141_65605	5658	11	1	0	Low-quality	22.38
Sample_T11C_k141_66609_fragment_2	25228	29	5	8	Low-quality	22.38
Sample_T2B_k141_87040	13406	21	3	0	Low-quality	22.38
Sample_C2B_k141_184343	7728	14	12	0	Low-quality	22.37
Sample_C3A_k141_2771_fragment_1	10662	12	2	2	Low-quality	22.37
Sample_T14C_k141_3794	10077	13	7	0	Low-quality	22.36
Sample_T12A_k141_212489	11168	9	0	5	Low-quality	22.35
Sample_C3A_k141_194155	8874	7	0	0	Low-quality	22.33
Sample_C4B_k141_198026	7743	3	0	2	Low-quality	22.33
Sample_T13A_k141_75087	13552	14	4	0	Low-quality	22.33
Sample_T18C_k141_136521	10377	10	0	3	Low-quality	22.32
Sample_T8C_k141_147336	9407	14	7	0	Low-quality	22.32
Sample_T12A_k141_4301	14634	20	4	3	Low-quality	22.31
Sample_T16B_k141_38496	55336	46	1	40	Low-quality	22.3
Sample_T3A_k141_27831	12182	13	0	1	Low-quality	22.3
Sample_T14A_k141_4806_fragment_1	11132	16	1	2	Low-quality	22.3
Sample_C10C_k141_113833	8918	14	3	1	Low-quality	22.29
Sample_C13A_k141_78542	21115	34	3	1	Low-quality	22.29
Sample_C2A_k141_22607	9253	10	3	0	Low-quality	22.28
Sample_T13B_k141_173696_fragment_1	18427	30	4	3	Low-quality	22.28
Sample_T3A_k141_412	8265	8	0	1	Low-quality	22.27

Sample_C3C_k141_231277	8811	13	5	0	Low-quality	22.27
Sample_T7A_k141_45264	14587	35	1	0	Low-quality	22.27
Sample_C11A_k141_99795	14711	21	1	4	Low-quality	22.26
Sample_C9A_k141_68769	8787	12	0	1	Low-quality	22.26
Sample_T3C_k141_58184	10564	18	2	2	Low-quality	22.26
Sample_C10C_k141_173936_fragment_1	11559	13	1	1	Low-quality	22.25
Sample_C6B_k141_173265_fragment_1	10138	15	7	2	Low-quality	22.25
Sample_T13B_k141_145153	16506	20	0	5	Low-quality	22.24
Sample_T7A_k141_46156_fragment_1	18038	16	1	7	Low-quality	22.22
Sample_C10A_k141_218421	9382	13	5	0	Low-quality	22.21
Sample_C8C_k141_140929	8258	14	2	0	Low-quality	22.21
Sample_T10A_k141_108177	24488	26	8	5	Low-quality	22.2
Sample_T13B_k141_105602	13294	17	0	3	Low-quality	22.2
Sample_C2A_k141_250921	9320	2	0	0	Low-quality	22.19
Sample_T11B_k141_115919	24225	23	0	10	Low-quality	22.19
Sample_T4A_k141_86226	13283	13	0	6	Low-quality	22.19
Sample_T6C_k141_266395	10308	9	0	5	Low-quality	22.19
Sample_T15C_k141_66291	14663	18	1	1	Low-quality	22.19
Sample_T8C_k141_108344	15253	17	0	4	Low-quality	22.18
Sample_C3C_k141_265359_fragment_1	10380	15	4	0	Low-quality	22.18
Sample_T16A_k141_17862	12038	21	4	1	Low-quality	22.17
Sample_T8A_k141_45092	12122	25	5	0	Low-quality	22.17
Sample_T11A_k141_73852	18107	13	0	6	Low-quality	22.16
Sample_T4B_k141_123848	8776	8	0	1	Low-quality	22.15
Sample_T4B_k141_152614	8170	13	4	0	Low-quality	22.15
Sample_C1C_k141_142144	11697	17	1	3	Low-quality	22.14
Sample_T6C_k141_27112	13660	17	1	12	Low-quality	22.14
Sample_C11C_k141_160669	10425	13	1	0	Low-quality	22.14
Sample_C4B_k141_124581	10175	19	8	0	Low-quality	22.14
Sample_C3B_k141_173466	12838	17	1	0	Low-quality	22.13
Sample_T2B_k141_232977	10576	13	5	0	Low-quality	22.13
Sample_T13B_k141_136190	13242	18	0	5	Low-quality	22.12
Sample_C7A_k141_100287	14526	23	2	1	Low-quality	22.12
Sample_T10A_k141_165280	8112	16	4	1	Low-quality	22.12
Sample_T2C_k141_13380	9013	14	6	0	Low-quality	22.12
Sample_T11B_k141_17077	9667	14	2	0	Low-quality	22.11
Sample_C14B_k141_10969	10055	17	1	3	Low-quality	22.1
Sample_T16B_k141_114761	14602	17	1	5	Low-quality	22.09
Sample_T7A_k141_143806	7563	10	3	0	Low-quality	22.09

Sample_C11C_k141_100606	34980	32	2	4	Low-quality	22.08
Sample_T5C_k141_123712	19862	18	0	3	Low-quality	22.07
Sample_T5C_k141_84640	11729	16	1	1	Low-quality	22.06
Sample_T2C_k141_39462	17050	22	1	7	Low-quality	22.05
Sample_C8B_k141_170213	8855	6	1	0	Low-quality	22.05
Sample_T4B_k141_2328_fragment_1	34989	42	2	1	Low-quality	22.05
Sample_T12A_k141_122183	9442	2	0	0	Low-quality	22.04
Sample_T6C_k141_56233	29089	27	11	5	Low-quality	22.04
Sample_C3B_k141_265128	16480	29	2	4	Low-quality	22.03
Sample_C4A_k141_256027	23769	20	0	8	Low-quality	22.03
Sample_C3B_k141_107484	8942	12	7	0	Low-quality	22.03
Sample_C10A_k141_114867	170073	175	4	96	Low-quality	22.02
Sample_T10C_k141_32003	35036	41	1	4	Low-quality	22.02
Sample_C3C_k141_51447	12684	22	3	1	Low-quality	22.02
Sample_T12A_k141_158778	8517	15	8	0	Low-quality	22.02
Sample_T5B_k141_36543	9082	14	1	0	Low-quality	22.02
Sample_C10A_k141_106008	9797	15	7	0	Low-quality	22
Sample_T6A_k141_122451	7234	15	4	0	Low-quality	22
Sample_T8B_k141_189351	8771	17	8	2	Low-quality	22
Sample_T8C_k141_101288	8403	12	5	0	Low-quality	22
Sample_C4A_k141_212597	9249	4	0	4	Low-quality	21.97
Sample_C6A_k141_211994	36123	31	1	16	Low-quality	21.97
Sample_T2B_k141_280960	27084	15	1	5	Low-quality	21.97
Sample_C3C_k141_50992	12652	20	3	1	Low-quality	21.97
Sample_T11A_k141_93360	9130	10	0	1	Low-quality	21.96
Sample_C6B_k141_170659	8901	7	0	1	Low-quality	21.95
Sample_T16B_k141_64624	8214	12	12	0	Low-quality	21.95
Sample_T7C_k141_118052	13064	13	5	0	Low-quality	21.94
Sample_T18A_k141_192611	9283	9	0	2	Low-quality	21.93
Sample_C2B_k141_131729	34164	55	1	41	Low-quality	21.89
Sample_C1A_k141_21252	9195	12	0	5	Low-quality	21.88
Sample_T8B_k141_253481	14777	7	2	1	Low-quality	21.87
Sample_C10C_k141_38460	9253	17	1	1	Low-quality	21.87
Sample_C9A_k141_123893	8421	15	3	0	Low-quality	21.87
Sample_T12B_k141_126946	17156	21	0	4	Low-quality	21.86
Sample_C13A_k141_60262_fragment_1	22000	32	4	2	Low-quality	21.86
Sample_T3B_k141_111349	10407	16	5	0	Low-quality	21.86
Sample_C3A_k141_234962	11602	15	5	0	Low-quality	21.85
Sample_T12A_k141_91764_fragment_1	11684	16	1	0	Low-quality	21.85

Sample_C10C_k141_203153	21374	21	0	5	Low-quality	21.83
Sample_C1C_k141_82721	43769	49	2	11	Low-quality	21.83
Sample_C6A_k141_31766	54931	50	1	19	Low-quality	21.83
Sample_C3A_k141_166746	15953	18	1	4	Low-quality	21.82
Sample_C13A_k141_32861	7288	8	1	0	Low-quality	21.82
Sample_C8C_k141_50166	9385	11	0	1	Low-quality	21.82
Sample_T8C_k141_98486	10061	13	10	1	Low-quality	21.82
Sample_T13B_k141_142894	9320	6	0	2	Low-quality	21.81
Sample_T17C_k141_13901	23575	30	0	21	Low-quality	21.8
Sample_C6A_k141_6027	13574	20	3	0	Low-quality	21.8
Sample_C6C_k141_121437	38048	30	0	23	Low-quality	21.79
Sample_T3A_k141_100887	11916	16	8	0	Low-quality	21.78
Sample_T7A_k141_84882	13527	22	3	0	Low-quality	21.78
Sample_T13C_k141_65146	11005	10	2	2	Low-quality	21.76
Sample_T18C_k141_2885	15928	19	0	4	Low-quality	21.76
Sample_T18C_k141_82759	10863	10	1	6	Low-quality	21.76
Sample_T8C_k141_96748	15076	33	9	0	Low-quality	21.75
Sample_C13C_k141_70773	18639	14	1	4	Low-quality	21.74
Sample_T10B_k141_96368	12559	25	4	1	Low-quality	21.74
Sample_T3B_k141_113111	10318	9	0	1	Low-quality	21.73
Sample_T7A_k141_143952	8688	11	0	0	Low-quality	21.73
Sample_C2A_k141_113193	9435	12	5	0	Low-quality	21.73
Sample_T11A_k141_54101	15444	15	1	5	Low-quality	21.72
Sample_T7A_k141_128625	8399	9	0	4	Low-quality	21.72
Sample_C4A_k141_175205	7945	11	2	0	Low-quality	21.72
Sample_T7C_k141_63787	9790	17	6	0	Low-quality	21.72
Sample_C14B_k141_65750	17537	22	1	7	Low-quality	21.71
Sample_T5B_k141_73834	8801	14	2	0	Low-quality	21.71
Sample_T15B_k141_103685	8071	13	4	0	Low-quality	21.71
Sample_C4C_k141_68651	14192	19	0	4	Low-quality	21.68
Sample_T11C_k141_135623	8747	18	4	0	Low-quality	21.68
Sample_C8A_k141_171387	14322	11	1	5	Low-quality	21.67
Sample_C12A_k141_165183	8906	8	8	0	Low-quality	21.67
Sample_C4C_k141_190473	7388	12	1	0	Low-quality	21.67
Sample_C11A_k141_118116	18765	11	0	4	Low-quality	21.65
Sample_C8B_k141_77918	8369	11	1	1	Low-quality	21.64
Sample_C11C_k141_56192	23381	15	0	9	Low-quality	21.63
Sample_C9A_k141_38607	20688	25	0	3	Low-quality	21.63
Sample_T14B_k141_95693	14114	13	0	11	Low-quality	21.63

Sample_C11C_k141_7354	9496	13	4	0	Low-quality	21.62
Sample_T18C_k141_151691	22682	32	4	0	Low-quality	21.6
Sample_T2C_k141_54216	6193	7	1	0	Low-quality	21.59
Sample_C8C_k141_24929	16780	17	0	2	Low-quality	21.58
Sample_C10B_k141_82873	13341	8	3	0	Low-quality	21.58
Sample_T17A_k141_6310	12433	15	3	1	Low-quality	21.58
Sample_T8A_k141_59980	12276	15	4	0	Low-quality	21.58
Sample_C2B_k141_8048	31381	37	2	23	Low-quality	21.57
Sample_T16B_k141_131320	9424	10	1	3	Low-quality	21.57
Sample_C6A_k141_208900	9087	25	4	1	Low-quality	21.57
Sample_C7C_k141_99528	10515	13	1	2	Low-quality	21.56
Sample_T6A_k141_125995	10775	9	0	6	Low-quality	21.56
Sample_C1B_k141_175299	15861	8	0	0	Low-quality	21.56
Sample_C9A_k141_9771	9959	17	11	0	Low-quality	21.56
Sample_C11C_k141_26870	12082	21	2	10	Low-quality	21.55
Sample_T3C_k141_106650	14245	24	1	2	Low-quality	21.55
Sample_C13C_k141_22589	14233	17	1	4	Low-quality	21.54
Sample_T17B_k141_2547	7650	6	3	1	Low-quality	21.54
Sample_C4C_k141_84506	8329	9	1	0	Low-quality	21.53
Sample_C5A_k141_262915	7740	14	6	2	Low-quality	21.53
Sample_T13B_k141_62812	11980	14	0	9	Low-quality	21.52
Sample_T8A_k141_215318	9045	17	0	16	Low-quality	21.52
Sample_T15B_k141_88344	8656	10	5	0	Low-quality	21.52
Sample_T4A_k141_108735_fragment_1	8682	13	2	0	Low-quality	21.52
Sample_T14A_k141_9051	16637	18	0	9	Low-quality	21.51
Sample_T2B_k141_18983	7925	9	4	0	Low-quality	21.51
Sample_T7A_k141_206921	21570	18	0	9	Low-quality	21.5
Sample_C8C_k141_118142	8159	15	1	1	Low-quality	21.5
Sample_C1A_k141_251871	25455	25	2	8	Low-quality	21.48
Sample_C12C_k141_187310	8987	10	2	0	Low-quality	21.48
Sample_T10A_k141_4708	8578	14	8	0	Low-quality	21.48
Sample_T6A_k141_112461	6192	7	0	2	Low-quality	21.46
Sample_C1C_k141_7424	8748	19	3	1	Low-quality	21.46
Sample_T14A_k141_48381	19305	17	0	4	Low-quality	21.45
Sample_C4B_k141_222991	17911	24	6	3	Low-quality	21.45
Sample_T4B_k141_186770	7715	8	0	0	Low-quality	21.45
Sample_C5A_k141_41011	21171	24	3	1	Low-quality	21.44
Sample_T11A_k141_72044	8062	10	4	0	Low-quality	21.44
Sample_T4B_k141_84320	65807	43	1	25	Low-quality	21.43

Sample_T8B_k141_273790	18018	28	2	2	Low-quality	21.43
Sample_C10A_k141_228133	7717	15	1	1	Low-quality	21.43
Sample_C12B_k141_58745	7632	16	0	0	Low-quality	21.43
Sample_T2B_k141_242611	9298	18	8	0	Low-quality	21.43
Sample_C6C_k141_134206	20189	13	0	3	Low-quality	21.42
Sample_T13B_k141_43681	8323	9	0	0	Low-quality	21.42
Sample_C4A_k141_83991	9228	15	1	0	Low-quality	21.42
Sample_T7A_k141_161016	17983	18	1	2	Low-quality	21.41
Sample_T8A_k141_84220	9739	9	2	0	Low-quality	21.41
Sample_C8B_k141_81913	12340	24	2	0	Low-quality	21.4
Sample_T15C_k141_43509	14706	21	0	0	Low-quality	21.4
Sample_C11A_k141_45691	17963	24	1	6	Low-quality	21.39
Sample_T4A_k141_86752	12792	17	1	6	Low-quality	21.37
Sample_C1A_k141_210645	17590	18	0	1	Low-quality	21.36
Sample_C6C_k141_35296	5619	3	1	0	Low-quality	21.36
Sample_C7B_k141_61064	14114	11	1	5	Low-quality	21.36
Sample_T10A_k141_85849	35092	36	1	19	Low-quality	21.36
Sample_T8B_k141_198011	8262	3	1	0	Low-quality	21.36
Sample_C6C_k141_34341	8329	12	10	0	Low-quality	21.36
Sample_C9B_k141_100782	12368	20	4	1	Low-quality	21.36
Sample_T5C_k141_99228	8129	16	5	0	Low-quality	21.36
Sample_T7B_k141_89503	54964	69	3	1	Low-quality	21.36
Sample_T12A_k141_267831	10532	12	6	0	Low-quality	21.34
Sample_T6A_k141_158299	7866	8	3	0	Low-quality	21.34
Sample_T8A_k141_87050	8717	17	1	0	Low-quality	21.34
Sample_C14A_k141_142194	7271	10	5	0	Low-quality	21.33
Sample_C4C_k141_155391	7800	11	7	0	Low-quality	21.33
Sample_C9A_k141_10209	8869	10	0	2	Low-quality	21.32
Sample_C9C_k141_35699	33647	35	1	10	Low-quality	21.32
Sample_T6B_k141_63878	21133	17	0	15	Low-quality	21.32
Sample_C4C_k141_251532	9008	9	1	0	Low-quality	21.32
Sample_C10A_k141_198079	16708	15	0	6	Low-quality	21.31
Sample_C7A_k141_54475	31708	35	1	11	Low-quality	21.31
Sample_T12A_k141_135189	52449	56	1	17	Low-quality	21.3
Sample_T13A_k141_36391	7974	16	0	1	Low-quality	21.3
Sample_T16B_k141_42843	6110	9	4	0	Low-quality	21.3
Sample_C10C_k141_99205	12738	15	0	6	Low-quality	21.28
Sample_T2B_k141_294003_fragment_2	9182	10	0	2	Low-quality	21.28
Sample_T6A_k141_135274	20101	16	0	2	Low-quality	21.27

Sample_C8C_k141_158517_fragment_1	7976	14	5	0	Low-quality	21.27
Sample_C4B_k141_184986_fragment_1	8742	16	3	0	Low-quality	21.26
Sample_C3C_k141_136358	11839	14	0	1	Low-quality	21.23
Sample_T7A_k141_191249	13721	20	0	4	Low-quality	21.22
Sample_C2B_k141_70704	10702	10	0	1	Low-quality	21.21
Sample_C3A_k141_256660	9839	16	9	0	Low-quality	21.21
Sample_T10B_k141_162947	13699	25	6	1	Low-quality	21.21
Sample_T2A_k141_236653	10398	10	0	4	Low-quality	21.2
Sample_C9A_k141_100000	22428	24	0	9	Low-quality	21.19
Sample_C14A_k141_158112_fragment_2	13823	19	3	2	Low-quality	21.19
Sample_C3B_k141_45641	12682	14	0	2	Low-quality	21.18
Sample_T15C_k141_105834	17284	22	2	5	Low-quality	21.18
Sample_C4A_k141_162997	15608	23	4	3	Low-quality	21.18
Sample_C7B_k141_77274	6075	4	0	2	Low-quality	21.17
Sample_T18C_k141_16091	16337	17	0	4	Low-quality	21.17
Sample_T7A_k141_79866	10906	10	1	3	Low-quality	21.17
Sample_C5A_k141_123587	9385	16	8	0	Low-quality	21.17
Sample_C7C_k141_91282_fragment_1	36937	31	7	5	Low-quality	21.17
Sample_C11B_k141_243587_fragment_1	14819	20	8	0	Low-quality	21.15
Sample_C1C_k141_744_fragment_1	8946	15	3	0	Low-quality	21.15
Sample_T16B_k141_155189_fragment_4	10275	19	3	0	Low-quality	21.15
Sample_C9A_k141_19901	8696	6	3	1	Low-quality	21.14
Sample_T11C_k141_53803	9790	8	6	0	Low-quality	21.14
Sample_C6C_k141_4714	7980	19	1	0	Low-quality	21.12
Sample_T13B_k141_166188	7831	14	6	0	Low-quality	21.11
Sample_T14C_k141_146979	16419	11	0	3	Low-quality	21.1
Sample_T17C_k141_26971	10045	16	10	0	Low-quality	21.1
Sample_C13A_k141_46005	8760	12	9	0	Low-quality	21.09
Sample_T14B_k141_69582	7877	14	1	0	Low-quality	21.08
Sample_T2C_k141_111022	115437	86	1	56	Low-quality	21.08
Sample_C12B_k141_82196	11271	14	12	0	Low-quality	21.08
Sample_T7A_k141_61397_fragment_1	8327	14	3	0	Low-quality	21.08
Sample_T18A_k141_116346	9000	10	0	5	Low-quality	21.07
Sample_T2C_k141_67019	52274	37	1	26	Low-quality	21.07
Sample_C6B_k141_82893	9002	12	1	1	Low-quality	21.07
Sample_T17B_k141_19388	18983	33	12	0	Low-quality	21.06
Sample_T3A_k141_47953	19707	13	1	10	Low-quality	21.05
Sample_T6C_k141_4873	13486	16	0	6	Low-quality	21.05
Sample_T16A_k141_92405	11299	26	6	2	Low-quality	21.05

Sample_T7A_k141_144063	12187	11	0	1	Low-quality	21.04
Sample_T6C_k141_256258	12817	16	7	0	Low-quality	21.04
Sample_T17A_k141_51366	13338	12	0	3	Low-quality	21.02
Sample_C3A_k141_55238	9627	15	1	0	Low-quality	21.02
Sample_T2A_k141_155543	16577	11	0	4	Low-quality	21
Sample_T6B_k141_188762	8969	14	1	2	Low-quality	21
Sample_T8A_k141_134147	11788	13	0	7	Low-quality	21
Sample_T7C_k141_31415	8538	10	5	0	Low-quality	21
Sample_C6B_k141_97600	10243	12	1	3	Low-quality	20.99
Sample_C8C_k141_28463	8149	6	0	1	Low-quality	20.99
Sample_T16B_k141_139081	11204	13	1	7	Low-quality	20.99
Sample_C13C_k141_102304	10469	23	15	0	Low-quality	20.99
Sample_C11C_k141_98095	23349	18	0	6	Low-quality	20.98
Sample_T11C_k141_23351	37698	34	0	22	Low-quality	20.98
Sample_C10C_k141_212120	7653	10	5	0	Low-quality	20.98
Sample_C12B_k141_140539	55178	28	6	0	Low-quality	20.98
Sample_T2A_k141_91592	51616	47	1	6	Low-quality	20.96
Sample_C7B_k141_82335	7941	14	5	0	Low-quality	20.95
Sample_T3A_k141_104098	11058	15	5	0	Low-quality	20.95
Sample_T6B_k141_171181	10454	20	1	3	Low-quality	20.94
Sample_C5A_k141_118303	11673	7	0	1	Low-quality	20.93
Sample_T4A_k141_42274	11652	11	0	9	Low-quality	20.93
Sample_C10A_k141_78241_fragment_1	8720	8	4	0	Low-quality	20.93
Sample_C10A_k141_334218	7006	10	7	0	Low-quality	20.93
Sample_C3A_k141_216193	8769	21	4	1	Low-quality	20.93
Sample_C6B_k141_126922	9842	16	11	0	Low-quality	20.93
Sample_T10A_k141_64503	8451	11	3	0	Low-quality	20.93
Sample_T5C_k141_27259	10017	14	10	0	Low-quality	20.93
Sample_C1B_k141_16601	20596	19	0	15	Low-quality	20.92
Sample_C9B_k141_84635_fragment_3	32875	31	8	0	Low-quality	20.92
Sample_T11C_k141_31601	13757	19	1	1	Low-quality	20.92
Sample_C12B_k141_151653	7474	5	4	1	Low-quality	20.91
Sample_T8B_k141_39977	21624	19	0	12	Low-quality	20.91
Sample_C12C_k141_160452	10667	6	1	0	Low-quality	20.9
Sample_C9B_k141_2023	8549	9	2	1	Low-quality	20.89
Sample_C9C_k141_45905_fragment_1	7622	16	6	0	Low-quality	20.89
Sample_T15A_k141_128576	33095	31	1	4	Low-quality	20.88
Sample_T8C_k141_115247	15532	17	1	1	Low-quality	20.87
Sample_C10A_k141_277038	14524	16	2	8	Low-quality	20.86

Sample_C2A_k141_40914	6016	7	0	1	Low-quality	20.85
Sample_T16B_k141_171313	9693	7	0	4	Low-quality	20.85
Sample_T2B_k141_74898	12515	13	1	3	Low-quality	20.85
Sample_T4B_k141_230036	22895	23	1	3	Low-quality	20.85
Sample_C12A_k141_119019	8862	20	2	0	Low-quality	20.84
Sample_C4C_k141_63161	7948	7	0	1	Low-quality	20.84
Sample_C9B_k141_24829	9233	9	6	0	Low-quality	20.84
Sample_T3C_k141_67717	12703	27	7	0	Low-quality	20.84
Sample_C9C_k141_88505	30514	32	2	5	Low-quality	20.83
Sample_T5C_k141_61185	8819	12	0	1	Low-quality	20.83
Sample_T7B_k141_88009	11982	13	0	1	Low-quality	20.83
Sample_C4A_k141_6509	9854	15	11	0	Low-quality	20.83
Sample_C4B_k141_182941	8251	5	0	4	Low-quality	20.82
Sample_C5C_k141_88129	51673	41	1	24	Low-quality	20.82
Sample_C10A_k141_142023	20515	26	1	2	Low-quality	20.82
Sample_C6A_k141_98677	7649	15	4	0	Low-quality	20.82
Sample_C3A_k141_55385	6854	6	3	0	Low-quality	20.81
Sample_C11A_k141_3986	12453	16	0	4	Low-quality	20.8
Sample_C4C_k141_201577	26177	31	1	6	Low-quality	20.8
Sample_T6B_k141_132069	8774	9	1	0	Low-quality	20.79
Sample_T12A_k141_255625	9986	19	5	0	Low-quality	20.79
Sample_T3B_k141_138732	9730	12	6	0	Low-quality	20.79
Sample_C12B_k141_28367	15641	13	0	4	Low-quality	20.78
Sample_C4A_k141_161345	9660	8	0	3	Low-quality	20.78
Sample_C7C_k141_58638	19745	13	1	4	Low-quality	20.78
Sample_C3B_k141_92949	9073	15	3	0	Low-quality	20.78
Sample_C4A_k141_27379	21493	20	0	15	Low-quality	20.77
Sample_C7A_k141_44684	10016	16	0	1	Low-quality	20.77
Sample_T10A_k141_117047	7682	20	5	0	Low-quality	20.77
Sample_T18C_k141_75015_fragment_1	13025	14	2	1	Low-quality	20.77
Sample_T3A_k141_920	12593	14	5	0	Low-quality	20.77
Sample_C2B_k141_228312	7507	8	6	0	Low-quality	20.76
Sample_T14C_k141_61636	8859	19	3	0	Low-quality	20.76
Sample_T17B_k141_21480	9175	12	1	0	Low-quality	20.76
Sample_T18B_k141_6813	7246	8	7	0	Low-quality	20.76
Sample_T17C_k141_29705	8768	12	9	0	Low-quality	20.75
Sample_T12C_k141_37682	9644	9	0	3	Low-quality	20.74
Sample_C7A_k141_114601	28790	35	1	7	Low-quality	20.74
Sample_C8A_k141_60489_fragment_1	10219	12	0	0	Low-quality	20.74

Sample_C6B_k141_112089	18653	26	0	4	Low-quality	20.73
Sample_T14C_k141_86892	51433	37	1	26	Low-quality	20.73
Sample_T12C_k141_41629	43596	77	6	7	Low-quality	20.73
Sample_T3B_k141_26804	7837	15	8	0	Low-quality	20.72
Sample_T12A_k141_37532	24854	35	10	10	Low-quality	20.7
Sample_C14B_k141_57035	5282	11	0	0	Low-quality	20.7
Sample_C13A_k141_25377	12134	18	0	3	Low-quality	20.69
Sample_C12C_k141_188620	8357	13	5	0	Low-quality	20.69
Sample_C3B_k141_153438	13099	14	3	2	Low-quality	20.69
Sample_T8C_k141_58133	7907	10	0	1	Low-quality	20.68
Sample_C4A_k141_165467	13134	26	4	0	Low-quality	20.68
Sample_T17C_k141_38059_fragment_1	8537	8	0	3	Low-quality	20.68
Sample_C2B_k141_295347	8772	12	5	0	Low-quality	20.66
Sample_C5A_k141_235018	8331	12	6	0	Low-quality	20.66
Sample_T8A_k141_249501	13647	17	1	7	Low-quality	20.65
Sample_T11C_k141_74626	22432	22	4	1	Low-quality	20.65
Sample_T12B_k141_182195	12543	28	2	0	Low-quality	20.65
Sample_C2A_k141_9479	7982	6	0	2	Low-quality	20.64
Sample_T2C_k141_31136	32299	38	1	23	Low-quality	20.64
Sample_C4C_k141_112474	13333	18	1	0	Low-quality	20.64
Sample_C4C_k141_45696	7741	20	4	0	Low-quality	20.64
Sample_T18C_k141_125424_fragment_2	9991	17	2	0	Low-quality	20.64
Sample_T2B_k141_200159	9793	24	4	1	Low-quality	20.64
Sample_C4B_k141_71444	31822	31	0	16	Low-quality	20.63
Sample_T10B_k141_86537	50121	41	1	11	Low-quality	20.63
Sample_T10A_k141_122844	9475	11	8	0	Low-quality	20.63
Sample_T2B_k141_247257_fragment_1	7938	13	6	0	Low-quality	20.63
Sample_T12A_k141_144530	42838	38	1	27	Low-quality	20.62
Sample_T13C_k141_75478	11032	17	6	0	Low-quality	20.62
Sample_T12A_k141_69728	14356	17	1	4	Low-quality	20.61
Sample_C3B_k141_129913	19971	34	4	8	Low-quality	20.59
Sample_C5A_k141_45920	19051	23	3	0	Low-quality	20.59
Sample_T16B_k141_111171	17696	17	0	3	Low-quality	20.57
Sample_C14A_k141_36003	7850	13	1	0	Low-quality	20.56
Sample_C7C_k141_64706	8933	15	3	1	Low-quality	20.56
Sample_T13B_k141_157572	8176	14	0	0	Low-quality	20.56
Sample_T13B_k141_48017	7632	14	4	0	Low-quality	20.56
Sample_T3B_k141_137229	8555	8	4	1	Low-quality	20.56
Sample_T4C_k141_100821	8853	8	0	0	Low-quality	20.56

Sample_T8A_k141_80539	10666	13	0	2	Low-quality	20.55
Sample_T4A_k141_105578	12380	17	11	0	Low-quality	20.55
Sample_T8C_k141_73737	8416	18	5	0	Low-quality	20.55
Sample_T15A_k141_109788	6246	9	0	0	Low-quality	20.54
Sample_C1C_k141_191043_fragment_1	11952	9	0	1	Low-quality	20.54
Sample_C8A_k141_160155	7424	12	1	0	Low-quality	20.54
Sample_T13C_k141_22171	9927	19	3	1	Low-quality	20.53
Sample_T2C_k141_261111	8793	18	1	1	Low-quality	20.53
Sample_C10A_k141_172664	14994	16	1	2	Low-quality	20.52
Sample_C10B_k141_97167	16545	27	7	2	Low-quality	20.51
Sample_T2A_k141_121729	9195	19	2	0	Low-quality	20.51
Sample_C14A_k141_105680	11200	14	6	2	Low-quality	20.5
Sample_T6C_k141_122990	31833	24	1	15	Low-quality	20.49
Sample_C10C_k141_26936_fragment_1	8558	15	3	0	Low-quality	20.49
Sample_C11A_k141_130022_fragment_1	10682	17	1	0	Low-quality	20.49
Sample_C9C_k141_11672	12108	13	3	1	Low-quality	20.49
Sample_T12A_k141_85252	8520	9	5	0	Low-quality	20.49
Sample_T15B_k141_14447	10463	13	12	0	Low-quality	20.49
Sample_C2A_k141_257361	30592	36	8	1	Low-quality	20.48
Sample_T2B_k141_35898	27695	36	14	0	Low-quality	20.46
Sample_C10A_k141_95103	44213	34	0	29	Low-quality	20.45
Sample_T5B_k141_3023	20552	15	0	7	Low-quality	20.45
Sample_C11A_k141_9567	36614	59	9	0	Low-quality	20.44
Sample_C12B_k141_151236	7211	17	5	0	Low-quality	20.44
Sample_T14B_k141_109768	7302	17	3	0	Low-quality	20.44
Sample_T16A_k141_102544	7225	10	8	0	Low-quality	20.44
Sample_T12A_k141_83267	13547	26	5	0	Low-quality	20.43
Sample_T8B_k141_125221_fragment_1	13456	30	8	2	Low-quality	20.43
Sample_C14A_k141_123166	8341	10	5	0	Low-quality	20.4
Sample_C10A_k141_140309	11882	11	1	5	Low-quality	20.39
Sample_C5C_k141_18359	12209	15	1	2	Low-quality	20.39
Sample_C10A_k141_97068	7617	16	0	0	Low-quality	20.39
Sample_C11B_k141_246168	7616	13	6	0	Low-quality	20.38
Sample_T8B_k141_46977	9325	11	2	0	Low-quality	20.38
Sample_T14A_k141_8446	7225	13	5	0	Low-quality	20.37
Sample_T14B_k141_207965	7601	9	6	0	Low-quality	20.37
Sample_T6B_k141_156974	10083	16	0	6	Low-quality	20.36
Sample_T8B_k141_3421	29441	41	9	0	Low-quality	20.36
Sample_C13C_k141_92297	14766	15	0	6	Low-quality	20.35

Sample_C7B_k141_91359	7151	10	8	0	Low-quality	20.34
Sample_T10B_k141_171850_fragment_1	27828	26	2	0	Low-quality	20.34
Sample_C13B_k141_37202	8542	9	0	6	Low-quality	20.33
Sample_C1C_k141_79544	21853	26	0	5	Low-quality	20.33
Sample_C2C_k141_253078	16136	12	0	2	Low-quality	20.33
Sample_C10A_k141_111139	8839	14	1	0	Low-quality	20.33
Sample_T11B_k141_118944	28189	38	1	8	Low-quality	20.31
Sample_C12A_k141_100977	6613	8	2	0	Low-quality	20.3
Sample_C9B_k141_32997	13131	21	5	0	Low-quality	20.3
Sample_T11C_k141_153715	29280	39	2	1	Low-quality	20.29
Sample_T6C_k141_221110	7923	5	1	0	Low-quality	20.29
Sample_T10B_k141_53339	16703	17	10	0	Low-quality	20.29
Sample_T2B_k141_316389	11153	20	9	0	Low-quality	20.28
Sample_T4B_k141_289172	7201	9	2	0	Low-quality	20.27
Sample_C5C_k141_66668_fragment_1	35961	43	1	9	Low-quality	20.27
Sample_C10C_k141_80913	22550	19	0	6	Low-quality	20.26
Sample_C4C_k141_72790	8183	6	0	1	Low-quality	20.26
Sample_C11C_k141_28107	6175	9	5	0	Low-quality	20.26
Sample_C7A_k141_168854	13383	16	1	2	Low-quality	20.25
Sample_C7A_k141_132286	16112	20	0	8	Low-quality	20.25
Sample_C10A_k141_257476	11418	14	0	0	Low-quality	20.25
Sample_C8B_k141_42653	12057	14	4	0	Low-quality	20.25
Sample_T12C_k141_143603	14016	21	2	0	Low-quality	20.25
Sample_T2C_k141_72077_fragment_2	13401	12	4	1	Low-quality	20.25
Sample_T15C_k141_141271	141324	149	4	81	Low-quality	20.23
Sample_T10C_k141_34118	9341	13	4	1	Low-quality	20.23
Sample_T8C_k141_53262	10201	11	3	0	Low-quality	20.23
Sample_C5A_k141_225767	11273	8	0	1	Low-quality	20.22
Sample_T10C_k141_49172	12223	26	7	0	Low-quality	20.21
Sample_T15A_k141_15325	12517	15	3	1	Low-quality	20.21
Sample_C2B_k141_330727	20024	20	3	3	Low-quality	20.2
Sample_C11A_k141_179853_fragment_1	8677	16	2	0	Low-quality	20.2
Sample_C11B_k141_203184	8150	7	0	0	Low-quality	20.2
Sample_C11C_k141_104476	9450	9	3	1	Low-quality	20.2
Sample_T10B_k141_160964	7561	13	2	1	Low-quality	20.2
Sample_C3A_k141_214548	31932	34	2	8	Low-quality	20.19
Sample_T17A_k141_24688	14849	17	0	4	Low-quality	20.18
Sample_T13B_k141_58812	9110	10	8	0	Low-quality	20.18
Sample_C6B_k141_48031	36533	41	1	21	Low-quality	20.16

Sample_T12B_k141_88806	9303	13	12	0	Low-quality	20.16
Sample_C14C_k141_88240	8035	11	8	0	Low-quality	20.14
Sample_C8C_k141_21926	9285	12	11	0	Low-quality	20.14
Sample_T11A_k141_24455	11195	12	0	4	Low-quality	20.13
Sample_T10A_k141_156827_fragment_1	10308	15	2	1	Low-quality	20.13
Sample_T5C_k141_54838	11473	14	0	3	Low-quality	20.13
Sample_C13A_k141_80338	8078	10	5	0	Low-quality	20.12
Sample_C3C_k141_223006	7644	6	0	1	Low-quality	20.11
Sample_C1B_k141_8307	9109	18	4	0	Low-quality	20.11
Sample_C9A_k141_134640	7452	16	0	6	Low-quality	20.1
Sample_T13C_k141_82185	11989	15	1	2	Low-quality	20.1
Sample_T7A_k141_117044	18088	22	1	6	Low-quality	20.1
Sample_T15A_k141_36145	33009	74	5	3	Low-quality	20.1
Sample_T2B_k141_171393	7272	12	6	0	Low-quality	20.1
Sample_C3A_k141_167122	13546	11	2	0	Low-quality	20.09
Sample_T17B_k141_20925_fragment_1	8507	15	1	0	Low-quality	20.08
Sample_T3B_k141_172242	9248	13	8	0	Low-quality	20.06
Sample_C9C_k141_93250	15247	8	2	5	Low-quality	20.05
Sample_C10A_k141_210180	7928	14	2	0	Low-quality	20.05
Sample_C14A_k141_237127	14448	12	2	2	Low-quality	20.05
Sample_T8C_k141_63329	8377	12	0	0	Low-quality	20.05
Sample_C3B_k141_157503	12645	15	0	2	Low-quality	20.04
Sample_C3C_k141_138869	6460	11	1	0	Low-quality	20.04
Sample_C12C_k141_128686	6772	12	2	0	Low-quality	20.03
Sample_C8B_k141_3001_fragment_1	15870	26	6	0	Low-quality	20.03
Sample_T16C_k141_26196	12523	17	13	0	Low-quality	20.03
Sample_T8C_k141_87087	8555	11	7	0	Low-quality	20.03
Sample_C9A_k141_125566	7532	8	0	3	Low-quality	20.02
Sample_C14B_k141_51326	8848	11	0	0	Low-quality	20.02
Sample_C6C_k141_13746_fragment_2	19756	22	4	1	Low-quality	20.02
Sample_T10A_k141_29508	9417	15	5	0	Low-quality	20.02
Sample_T8C_k141_80095	8019	12	5	0	Low-quality	20.02
Sample_T7A_k141_70871	21655	21	0	6	Low-quality	20.01
Sample_C3A_k141_104738	9507	12	5	0	Low-quality	20.01
Sample_T4C_k141_1753	12884	22	3	0	Low-quality	20.01
Sample_C10A_k141_266384	17993	20	0	7	Low-quality	19.99
Sample_T13B_k141_15285	9583	19	5	0	Low-quality	19.99
Sample_T16B_k141_152492	29068	23	6	3	Low-quality	19.99
Sample_C11B_k141_50636	8078	17	3	0	Low-quality	19.98

Sample_C5A_k141_115645	6846	9	1	0	Low-quality	19.98
Sample_T14B_k141_199430	22480	20	1	2	Low-quality	19.98
Sample_C3A_k141_172341	10946	10	5	3	Low-quality	19.97
Sample_T4C_k141_43964	10674	15	11	1	Low-quality	19.97
Sample_T17B_k141_32398	15381	27	3	1	Low-quality	19.97
Sample_T16B_k141_17774	24399	18	0	9	Low-quality	19.96
Sample_T7C_k141_64136	7616	7	0	2	Low-quality	19.96
Sample_C2A_k141_84808	19801	17	0	4	Low-quality	19.95
Sample_C2B_k141_308547	15641	19	1	4	Low-quality	19.95
Sample_T2C_k141_247564	29672	34	1	4	Low-quality	19.95
Sample_C8B_k141_36106	14837	22	1	8	Low-quality	19.94
Sample_T17A_k141_48663_fragment_1	8144	11	4	1	Low-quality	19.94
Sample_T5A_k141_23923	9653	12	8	0	Low-quality	19.93
Sample_T13C_k141_17484	13151	14	1	4	Low-quality	19.9
Sample_C4A_k141_238617	10661	14	4	0	Low-quality	19.9
Sample_T13B_k141_136084	10294	11	7	0	Low-quality	19.9
Sample_T15C_k141_73693	37371	50	4	0	Low-quality	19.9
Sample_T2A_k141_128482	8074	20	1	0	Low-quality	19.9
Sample_C2C_k141_25206	37158	40	1	18	Low-quality	19.89
Sample_C4B_k141_104078	7035	18	3	0	Low-quality	19.89
Sample_C3B_k141_257562	16472	20	2	2	Low-quality	19.88
Sample_C8C_k141_37541	8048	9	0	2	Low-quality	19.88
Sample_T17B_k141_37909	13056	13	2	2	Low-quality	19.88
Sample_C1C_k141_181578	7520	18	3	0	Low-quality	19.87
Sample_T18B_k141_36417	7775	13	4	0	Low-quality	19.86
Sample_C11A_k141_65555	6440	11	1	0	Low-quality	19.85
Sample_C11B_k141_162811	8341	8	0	4	Low-quality	19.85
Sample_C11C_k141_98447	8379	11	0	1	Low-quality	19.85
Sample_T7A_k141_6202	7174	12	5	0	Low-quality	19.85
Sample_T13C_k141_67309	5692	9	2	0	Low-quality	19.84
Sample_C3B_k141_64655_fragment_1	26040	49	5	3	Low-quality	19.84
Sample_T14B_k141_196658_fragment_1	17314	20	5	5	Low-quality	19.84
Sample_C1B_k141_211339	15411	16	0	5	Low-quality	19.83
Sample_T5A_k141_37214	29476	31	1	11	Low-quality	19.83
Sample_T16C_k141_20773	19655	12	0	5	Low-quality	19.82
Sample_T10A_k141_67233	7415	10	4	0	Low-quality	19.82
Sample_T2A_k141_234872	7064	11	7	0	Low-quality	19.82
Sample_C10C_k141_133422	6941	12	3	1	Low-quality	19.8
Sample_T8B_k141_252539	6544	14	1	0	Low-quality	19.8

Sample_T17A_k141_7400	11341	14	0	1	Low-quality	19.78
Sample_T15C_k141_67647	6420	16	2	0	Low-quality	19.78
Sample_T5A_k141_46760	9238	10	0	1	Low-quality	19.78
Sample_C11B_k141_137367	55513	57	1	34	Low-quality	19.77
Sample_C10C_k141_19195	17124	13	1	6	Low-quality	19.76
Sample_C9B_k141_99190	7893	7	0	1	Low-quality	19.76
Sample_T12A_k141_106505	7830	10	0	0	Low-quality	19.76
Sample_T17B_k141_3736	13055	14	1	1	Low-quality	19.75
Sample_T4B_k141_153800	67048	68	1	38	Low-quality	19.75
Sample_T5C_k141_170031	20636	46	2	0	Low-quality	19.75
Sample_T14C_k141_71132	8298	8	0	7	Low-quality	19.74
Sample_T6B_k141_192933	21380	19	0	4	Low-quality	19.74
Sample_T5A_k141_48163	7627	8	0	4	Low-quality	19.72
Sample_T14A_k141_204000_fragment_1	9169	9	0	1	Low-quality	19.72
Sample_C13B_k141_19883	10109	8	0	1	Low-quality	19.71
Sample_C1B_k141_228182	7226	10	5	0	Low-quality	19.71
Sample_T14B_k141_148535	12357	10	0	0	Low-quality	19.71
Sample_C12C_k141_12994	6348	13	1	2	Low-quality	19.7
Sample_C2B_k141_75774	13019	17	1	2	Low-quality	19.7
Sample_C5B_k141_9823	9563	14	6	0	Low-quality	19.7
Sample_T5C_k141_49114	6683	7	3	0	Low-quality	19.7
Sample_T15A_k141_37753	8357	6	0	2	Low-quality	19.69
Sample_C4B_k141_127806	45074	42	1	26	Low-quality	19.68
Sample_T12A_k141_81411	7684	8	0	3	Low-quality	19.68
Sample_T7A_k141_92061	25953	24	1	3	Low-quality	19.68
Sample_C7B_k141_16126	11876	18	7	0	Low-quality	19.68
Sample_T13B_k141_56067	8771	10	0	0	Low-quality	19.68
Sample_C4C_k141_1136	48439	37	1	6	Low-quality	19.67
Sample_T18C_k141_26668	13214	17	1	5	Low-quality	19.67
Sample_C6B_k141_168469	7273	15	2	0	Low-quality	19.67
Sample_T3A_k141_24633	11772	13	0	4	Low-quality	19.66
Sample_C2C_k141_264562_fragment_2	19891	21	1	4	Low-quality	19.66
Sample_T13B_k141_90231	7232	10	6	0	Low-quality	19.66
Sample_T6B_k141_47200	7697	12	0	9	Low-quality	19.65
Sample_C10B_k141_73562	7202	14	6	0	Low-quality	19.65
Sample_T12B_k141_172074	7684	12	3	0	Low-quality	19.65
Sample_T14A_k141_178726_fragment_2	9842	24	4	0	Low-quality	19.65
Sample_T8B_k141_39071	12575	15	1	11	Low-quality	19.64
Sample_C14C_k141_85125	8210	6	0	2	Low-quality	19.63

Sample_C3C_k141_250479	7443	12	1	2	Low-quality	19.63
Sample_T12A_k141_329799	13113	16	1	4	Low-quality	19.62
Sample_C12A_k141_122214_fragment_1	9942	16	4	0	Low-quality	19.62
Sample_C3A_k141_108706	9183	14	3	0	Low-quality	19.62
Sample_T7A_k141_128813	7770	12	7	0	Low-quality	19.62
Sample_T15B_k141_4363	36163	26	1	8	Low-quality	19.61
Sample_C12C_k141_17909	7445	17	6	0	Low-quality	19.61
Sample_C2B_k141_325109	8342	17	5	0	Low-quality	19.61
Sample_T12C_k141_102595	9490	11	3	1	Low-quality	19.61
Sample_T10B_k141_70424	14973	13	2	2	Low-quality	19.6
Sample_C12A_k141_133359	7128	8	4	1	Low-quality	19.59
Sample_C8B_k141_178195	7439	11	4	0	Low-quality	19.59
Sample_T8B_k141_26010	8544	14	4	0	Low-quality	19.59
Sample_C14A_k141_30974	7717	11	7	0	Low-quality	19.58
Sample_T11B_k141_76156	6731	10	8	0	Low-quality	19.58
Sample_C4B_k141_142113	54753	44	1	27	Low-quality	19.57
Sample_C6B_k141_202804	17504	27	0	4	Low-quality	19.57
Sample_C13C_k141_17629	9321	18	5	0	Low-quality	19.57
Sample_C2A_k141_134128	8731	11	7	0	Low-quality	19.56
Sample_T6C_k141_95419	8298	20	4	0	Low-quality	19.56
Sample_C11A_k141_54009	8476	7	0	1	Low-quality	19.55
Sample_C2C_k141_147326	7540	9	0	1	Low-quality	19.54
Sample_T8C_k141_59181	8772	12	0	2	Low-quality	19.54
Sample_C7B_k141_159556	11712	18	3	2	Low-quality	19.53
Sample_T17B_k141_7152	9499	19	2	0	Low-quality	19.53
Sample_C4A_k141_10124	29039	28	1	6	Low-quality	19.52
Sample_T14B_k141_77377	16073	13	0	1	Low-quality	19.52
Sample_C1B_k141_12159	7413	16	3	0	Low-quality	19.52
Sample_C3A_k141_137688	9240	11	3	0	Low-quality	19.52
Sample_T8A_k141_117312	8726	16	7	1	Low-quality	19.52
Sample_T11B_k141_37295	18417	13	1	4	Low-quality	19.51
Sample_T4C_k141_60597	9075	16	4	1	Low-quality	19.51
Sample_T17A_k141_37079	10858	8	0	7	Low-quality	19.5
Sample_C2A_k141_53959	9030	7	3	0	Low-quality	19.5
Sample_T4B_k141_53445	15471	12	0	3	Low-quality	19.49
Sample_C1C_k141_124082	11413	14	1	1	Low-quality	19.49
Sample_T8C_k141_105831	8434	16	1	0	Low-quality	19.49
Sample_C2B_k141_234254	8188	9	0	2	Low-quality	19.48
Sample_T11A_k141_7548	12873	16	1	4	Low-quality	19.48

Sample_T7C_k141_56615	8307	11	1	0	Low-quality	19.48
Sample_C13A_k141_79189	8272	11	3	0	Low-quality	19.48
Sample_T7A_k141_4932	7970	17	4	0	Low-quality	19.48
Sample_T5A_k141_20565	31376	26	2	2	Low-quality	19.47
Sample_T5B_k141_32593	17477	14	0	8	Low-quality	19.46
Sample_T1C_k141_27168	6754	9	1	0	Low-quality	19.46
Sample_C1A_k141_28251	7485	14	2	1	Low-quality	19.45
Sample_C3A_k141_37159	10147	7	3	1	Low-quality	19.45
Sample_T13B_k141_42103	11613	18	0	1	Low-quality	19.45
Sample_T4B_k141_92148	8005	13	7	0	Low-quality	19.44
Sample_T15A_k141_36291	31870	66	6	1	Low-quality	19.42
Sample_C10A_k141_89113	8583	16	6	0	Low-quality	19.41
Sample_T17B_k141_28004	8416	12	2	0	Low-quality	19.41
Sample_T7A_k141_99761_fragment_1	11871	13	0	2	Low-quality	19.41
Sample_C5C_k141_108328	10577	10	4	1	Low-quality	19.4
Sample_C8B_k141_27979_fragment_2	10765	18	13	1	Low-quality	19.4
Sample_T5A_k141_22857	16218	40	4	0	Low-quality	19.4
Sample_C12A_k141_18832	9128	11	5	0	Low-quality	19.39
Sample_C3A_k141_169833	10323	8	4	0	Low-quality	19.39
Sample_C10C_k141_60006	7762	14	3	0	Low-quality	19.38
Sample_C1C_k141_24756	8064	17	1	0	Low-quality	19.38
Sample_T6C_k141_35029	6943	10	4	0	Low-quality	19.38
Sample_T2C_k141_252735	9771	13	0	0	Low-quality	19.36
Sample_T12A_k141_33245	19187	10	0	10	Low-quality	19.35
Sample_T13B_k141_56318	7629	7	0	0	Low-quality	19.35
Sample_T13B_k141_107721	11330	18	3	3	Low-quality	19.35
Sample_T2B_k141_180985	7165	12	1	1	Low-quality	19.35
Sample_C3A_k141_171753	7996	22	2	0	Low-quality	19.35
Sample_T8B_k141_212608	9343	16	3	1	Low-quality	19.35
Sample_C12B_k141_145235	44437	54	1	27	Low-quality	19.34
Sample_C14C_k141_18922	20585	36	3	1	Low-quality	19.34
Sample_T6B_k141_147094	11601	12	0	5	Low-quality	19.34
Sample_T17A_k141_3546	8344	14	1	0	Low-quality	19.34
Sample_C14A_k141_118593	16698	14	0	3	Low-quality	19.33
Sample_T5B_k141_62039	7946	13	4	0	Low-quality	19.33
Sample_T10A_k141_61178	9159	13	1	0	Low-quality	19.32
Sample_T11A_k141_20994	52295	52	2	30	Low-quality	19.32
Sample_C5A_k141_177219	12736	16	0	4	Low-quality	19.31
Sample_T2C_k141_85150	14778	6	0	2	Low-quality	19.31

Sample_T8B_k141_131781	18750	17	0	3	Low-quality	19.31
Sample_T10A_k141_27180	16382	15	1	2	Low-quality	19.3
Sample_C14C_k141_35580_fragment_1	7832	11	5	0	Low-quality	19.3
Sample_C3A_k141_209607_fragment_2	9413	12	1	4	Low-quality	19.3
Sample_C7A_k141_178473	25227	32	2	0	Low-quality	19.29
Sample_C8A_k141_150320	9628	11	1	5	Low-quality	19.29
Sample_C8B_k141_129090	10783	15	3	0	Low-quality	19.28
Sample_T13B_k141_135486_fragment_1	8723	14	4	0	Low-quality	19.28
Sample_T6A_k141_10169	10046	13	1	0	Low-quality	19.27
Sample_T2B_k141_260390	11638	22	9	0	Low-quality	19.27
Sample_C13C_k141_45622	9834	16	6	2	Low-quality	19.26
Sample_T16C_k141_32106	7942	5	0	1	Low-quality	19.26
Sample_T6A_k141_93164	8094	9	0	6	Low-quality	19.26
Sample_T6C_k141_192040	7530	12	2	0	Low-quality	19.26
Sample_C8C_k141_78035	8402	9	2	1	Low-quality	19.25
Sample_C12A_k141_164338	8294	11	0	3	Low-quality	19.24
Sample_C8C_k141_97051	8629	8	0	3	Low-quality	19.24
Sample_T2A_k141_104975	20885	21	3	7	Low-quality	19.24
Sample_C10C_k141_9187	47382	44	1	2	Low-quality	19.24
Sample_C14A_k141_31657	10413	12	7	0	Low-quality	19.23
Sample_C8A_k141_81444	13000	19	2	1	Low-quality	19.23
Sample_T10A_k141_40396_fragment_1	34758	47	1	5	Low-quality	19.23
Sample_C11C_k141_180006	8839	11	9	1	Low-quality	19.22
Sample_C3A_k141_221864_fragment_1	8793	10	4	0	Low-quality	19.22
Sample_T8A_k141_63361_fragment_2	8043	11	1	0	Low-quality	19.22
Sample_C9A_k141_1778	238785	223	1	176	Low-quality	19.21
Sample_T3A_k141_118227	10098	11	3	0	Low-quality	19.21
Sample_T8C_k141_50215_fragment_1	8171	10	1	0	Low-quality	19.21
Sample_C5C_k141_107691	8953	3	1	0	Low-quality	19.2
Sample_C6C_k141_139219	8917	8	0	4	Low-quality	19.2
Sample_C14A_k141_1633_fragment_1	11369	9	3	0	Low-quality	19.2
Sample_C2C_k141_313384	15684	18	0	10	Low-quality	19.19
Sample_T16B_k141_58358	8113	15	1	1	Low-quality	19.19
Sample_T16B_k141_173424	6353	8	0	0	Low-quality	19.19
Sample_T18C_k141_148545	11227	19	4	1	Low-quality	19.19
Sample_T6C_k141_169910	7780	11	0	1	Low-quality	19.19
Sample_C13B_k141_40084	9098	12	9	1	Low-quality	19.19
Sample_T5A_k141_14192	7095	7	6	0	Low-quality	19.19
Sample_C12C_k141_201182	8630	12	1	0	Low-quality	19.18

Sample_C14C_k141_8839	10278	10	1	2	Low-quality	19.18
Sample_T11A_k141_128940	19893	20	0	16	Low-quality	19.18
Sample_T11C_k141_128808	14657	9	0	2	Low-quality	19.18
Sample_C1B_k141_182795	10555	6	0	0	Low-quality	19.18
Sample_C9A_k141_116986	20714	20	1	3	Low-quality	19.17
Sample_C3B_k141_249960	11051	25	1	0	Low-quality	19.17
Sample_C5A_k141_257842	8967	10	0	0	Low-quality	19.17
Sample_C11A_k141_11314	10061	14	0	3	Low-quality	19.16
Sample_T3A_k141_132495	18172	16	1	4	Low-quality	19.16
Sample_T13A_k141_14966	12284	16	5	1	Low-quality	19.16
Sample_C12C_k141_171035	14001	21	1	4	Low-quality	19.15
Sample_T14A_k141_134536	17231	13	0	6	Low-quality	19.15
Sample_T16B_k141_11210	9645	8	0	1	Low-quality	19.15
Sample_T2C_k141_158153	58153	55	0	37	Low-quality	19.15
Sample_C11B_k141_118565	7802	8	5	0	Low-quality	19.15
Sample_C3A_k141_145755	8584	10	1	0	Low-quality	19.15
Sample_C13B_k141_36639	7440	13	3	0	Low-quality	19.14
Sample_C1A_k141_55164	20097	29	7	0	Low-quality	19.14
Sample_C2B_k141_182710	8086	14	5	1	Low-quality	19.14
Sample_C4B_k141_258419	7812	7	6	0	Low-quality	19.14
Sample_C8C_k141_97639	10637	15	3	1	Low-quality	19.14
Sample_T12A_k141_315714	7528	11	4	0	Low-quality	19.14
Sample_T6B_k141_170694	5518	6	0	0	Low-quality	19.13
Sample_C4B_k141_144478	10492	13	5	0	Low-quality	19.13
Sample_C11B_k141_50332	7649	8	0	1	Low-quality	19.12
Sample_C2B_k141_95557	8007	19	5	0	Low-quality	19.12
Sample_T12A_k141_246508	11920	16	2	1	Low-quality	19.12
Sample_T6C_k141_141250	11232	11	3	0	Low-quality	19.12
Sample_T8C_k141_136629	8769	14	5	3	Low-quality	19.12
Sample_C9B_k141_97010	15618	19	0	10	Low-quality	19.11
Sample_T3B_k141_136626	121004	123	6	26	Low-quality	19.11
Sample_C12C_k141_224123	7435	12	6	0	Low-quality	19.11
Sample_C2B_k141_160966	7687	13	5	0	Low-quality	19.11
Sample_C3A_k141_97061_fragment_1	13546	20	11	0	Low-quality	19.11
Sample_C6C_k141_125937	9818	21	5	1	Low-quality	19.11
Sample_T5B_k141_66394	17189	14	0	1	Low-quality	19.1
Sample_T8C_k141_207075	11056	22	4	0	Low-quality	19.1
Sample_C2A_k141_76636	29492	28	1	12	Low-quality	19.09
Sample_C3A_k141_156306	31430	58	5	5	Low-quality	19.09

Sample_C7B_k141_138857	9455	19	1	0	Low-quality	19.08
Sample_T10C_k141_67805	7142	15	0	1	Low-quality	19.08
Sample_C4B_k141_71694	6976	7	6	0	Low-quality	19.08
Sample_C11B_k141_236001	10633	7	0	2	Low-quality	19.07
Sample_C3B_k141_259175	25985	51	3	11	Low-quality	19.06
Sample_T3A_k141_39100	6339	7	0	5	Low-quality	19.06
Sample_C14B_k141_87795_fragment_1	31850	46	7	1	Low-quality	19.06
Sample_C5B_k141_14121	8457	17	5	0	Low-quality	19.06
Sample_T12B_k141_71232	6673	12	7	0	Low-quality	19.06
Sample_T13B_k141_12821	8656	8	6	0	Low-quality	19.06
Sample_T18C_k141_97519	5370	5	0	0	Low-quality	19.05
Sample_C1B_k141_114689	5466	7	3	0	Low-quality	19.05
Sample_C3B_k141_18455	7932	9	0	1	Low-quality	19.04
Sample_C8C_k141_111925	13259	11	1	5	Low-quality	19.04
Sample_T3B_k141_10867	28317	32	1	7	Low-quality	19.04
Sample_C10A_k141_91882_fragment_2	8204	8	0	1	Low-quality	19.04
Sample_C6A_k141_59733	8408	16	2	0	Low-quality	19.04
Sample_T2B_k141_266708	6917	16	1	0	Low-quality	19.04
Sample_T2B_k141_101430	47214	30	1	22	Low-quality	19.03
Sample_C11C_k141_126153	33690	37	11	2	Low-quality	19.02
Sample_C13B_k141_108941	11462	14	2	0	Low-quality	19.02
Sample_T11B_k141_69143	21208	46	4	0	Low-quality	19.02
Sample_C5A_k141_220128	23444	16	1	5	Low-quality	19.01
Sample_C2B_k141_312996	8284	10	7	0	Low-quality	19.01
Sample_C2B_k141_315134	15319	38	6	0	Low-quality	19.01
Sample_T12A_k141_182006	25324	19	3	2	Low-quality	19.01
Sample_T15C_k141_39099	8496	16	5	0	Low-quality	19.01
Sample_T17B_k141_19879	7764	9	7	0	Low-quality	19.01
Sample_T2C_k141_61750	8874	11	7	0	Low-quality	19.01
Sample_C4C_k141_65571	9422	16	7	0	Low-quality	19
Sample_C2A_k141_94593	16128	26	4	0	Low-quality	18.99
Sample_C13B_k141_6301	18640	12	0	4	Low-quality	18.97
Sample_C8B_k141_125661	6807	10	4	0	Low-quality	18.97
Sample_T17A_k141_24198	7722	10	5	0	Low-quality	18.96
Sample_C9A_k141_28064	11242	16	0	1	Low-quality	18.95
Sample_T18B_k141_93460	34941	38	1	15	Low-quality	18.95
Sample_T4C_k141_88770	12524	12	1	2	Low-quality	18.95
Sample_T17B_k141_19719	5846	10	6	0	Low-quality	18.95
Sample_T6A_k141_5471	11339	13	1	0	Low-quality	18.94

Sample_C13C_k141_51280	6593	10	7	0	Low-quality	18.94
Sample_C9A_k141_31387	6832	13	5	0	Low-quality	18.94
Sample_T6C_k141_109653	8883	14	1	0	Low-quality	18.94
Sample_T13B_k141_36009	8122	17	4	0	Low-quality	18.93
Sample_T16B_k141_129946	8986	7	3	0	Low-quality	18.92
Sample_C13B_k141_5306	30497	32	2	7	Low-quality	18.91
Sample_C2A_k141_80554	7719	9	0	2	Low-quality	18.91
Sample_T7A_k141_51018	8246	12	0	0	Low-quality	18.91
Sample_C12C_k141_125514	8505	15	0	0	Low-quality	18.91
Sample_T13C_k141_90868	11184	12	3	0	Low-quality	18.91
Sample_T3B_k141_128713	9277	9	6	1	Low-quality	18.91
Sample_T8A_k141_73308_fragment_1	6825	11	2	1	Low-quality	18.91
Sample_C13B_k141_17991	7945	9	0	5	Low-quality	18.9
Sample_C3A_k141_157412_fragment_1	6847	15	5	0	Low-quality	18.9
Sample_T15B_k141_79817	5424	5	3	0	Low-quality	18.9
Sample_T3C_k141_91927	8083	10	1	0	Low-quality	18.9
Sample_C8C_k141_108395	12481	15	1	3	Low-quality	18.89
Sample_C4B_k141_164468	7411	10	6	0	Low-quality	18.89
Sample_T10A_k141_25587	6635	12	7	0	Low-quality	18.89
Sample_T13B_k141_114272_fragment_2	14704	15	1	0	Low-quality	18.89
Sample_C13A_k141_24038	9290	9	6	0	Low-quality	18.88
Sample_C10A_k141_33576	16587	15	0	9	Low-quality	18.86
Sample_T2A_k141_143549	14316	10	0	2	Low-quality	18.86
Sample_T5C_k141_169593	18219	19	2	0	Low-quality	18.86
Sample_C10A_k141_132779	6921	11	2	0	Low-quality	18.86
Sample_C12A_k141_73485_fragment_1	14956	17	2	1	Low-quality	18.86
Sample_C9C_k141_1201	7014	7	0	0	Low-quality	18.86
Sample_C9C_k141_76619_fragment_1	7334	14	5	0	Low-quality	18.85
Sample_C12C_k141_149298	7415	9	1	0	Low-quality	18.84
Sample_T10B_k141_178894	9548	10	1	4	Low-quality	18.84
Sample_T17A_k141_49936	6816	10	6	0	Low-quality	18.84
Sample_T17A_k141_20482	7515	5	0	0	Low-quality	18.83
Sample_C2B_k141_282388	13794	15	4	1	Low-quality	18.83
Sample_C2B_k141_53541	12909	14	0	1	Low-quality	18.82
Sample_C4C_k141_230973	16737	8	1	4	Low-quality	18.82
Sample_T8A_k141_30298	7004	12	1	0	Low-quality	18.82
Sample_T10B_k141_104009	30070	36	1	2	Low-quality	18.81
Sample_C11C_k141_62486	18141	21	5	2	Low-quality	18.8
Sample_C8C_k141_38951	7502	17	0	6	Low-quality	18.8

Sample_T16B_k141_17800	8821	20	7	1	Low-quality	18.8
Sample_C7C_k141_41710	12743	18	1	5	Low-quality	18.79
Sample_C8C_k141_106146	29304	37	2	2	Low-quality	18.79
Sample_T4B_k141_93565	119780	112	6	62	Low-quality	18.79
Sample_T8C_k141_3254	5689	7	4	0	Low-quality	18.79
Sample_T16B_k141_85256	32696	54	5	0	Low-quality	18.78
Sample_T13B_k141_115716	6995	9	1	0	Low-quality	18.77
Sample_T7C_k141_46193	45795	34	5	1	Low-quality	18.77
Sample_C10C_k141_69497	17208	16	0	13	Low-quality	18.76
Sample_C13B_k141_69561	9053	11	5	0	Low-quality	18.75
Sample_T8A_k141_30898	31330	27	1	7	Low-quality	18.74
Sample_C3A_k141_2271	11174	11	3	0	Low-quality	18.73
Sample_T12A_k141_95268	8435	17	4	1	Low-quality	18.73
Sample_C4C_k141_177441	7642	13	3	1	Low-quality	18.72
Sample_C8B_k141_197663	8193	9	0	0	Low-quality	18.72
Sample_T11A_k141_109933	7572	16	7	0	Low-quality	18.72
Sample_C4B_k141_213615	7559	10	0	0	Low-quality	18.71
Sample_C10C_k141_144457	7440	15	5	0	Low-quality	18.71
Sample_C2A_k141_177405	7889	12	3	0	Low-quality	18.7
Sample_T8C_k141_139704	12230	20	2	0	Low-quality	18.7
Sample_T15B_k141_111165	8861	9	0	2	Low-quality	18.69
Sample_T16A_k141_58125	14329	16	0	2	Low-quality	18.69
Sample_C14A_k141_216897	11689	12	3	3	Low-quality	18.69
Sample_C3A_k141_74740_fragment_1	7452	10	2	0	Low-quality	18.69
Sample_T14B_k141_111968	24377	24	1	8	Low-quality	18.68
Sample_C11B_k141_186527	8086	14	3	0	Low-quality	18.68
Sample_T11B_k141_60919	6673	11	3	0	Low-quality	18.68
Sample_T16A_k141_70977	7009	15	6	0	Low-quality	18.68
Sample_T16B_k141_40537	11275	26	6	0	Low-quality	18.68
Sample_T3A_k141_29159	9029	11	4	0	Low-quality	18.68
Sample_T10A_k141_70062	11180	15	1	7	Low-quality	18.67
Sample_T16C_k141_67381	11816	12	3	2	Low-quality	18.67
Sample_T10A_k141_160919	26068	21	1	16	Low-quality	18.66
Sample_T4C_k141_27167	9848	13	3	0	Low-quality	18.66
Sample_T6C_k141_237734_fragment_1	9896	14	4	1	Low-quality	18.66
Sample_C6A_k141_165089	45912	47	1	21	Low-quality	18.65
Sample_C8C_k141_9868	16656	13	1	5	Low-quality	18.65
Sample_T15B_k141_47464	29566	35	1	9	Low-quality	18.65
Sample_T15C_k141_129117	7662	7	0	2	Low-quality	18.65

Sample_T2C_k141_3385	6711	7	5	0	Low-quality	18.65
Sample_T13A_k141_28515	17573	18	0	5	Low-quality	18.64
Sample_T2C_k141_227033	51455	45	3	15	Low-quality	18.64
Sample_T13B_k141_15005	12322	9	1	0	Low-quality	18.64
Sample_T10B_k141_109888	7418	8	3	0	Low-quality	18.63
Sample_T18B_k141_141979	9805	14	5	0	Low-quality	18.63
Sample_T7C_k141_128952	8404	16	1	0	Low-quality	18.63
Sample_T5B_k141_2436	15040	8	0	1	Low-quality	18.62
Sample_T8C_k141_163302	11145	11	0	2	Low-quality	18.62
Sample_T3C_k141_71700_fragment_1	27982	27	4	3	Low-quality	18.62
Sample_T10C_k141_3519	6967	11	8	0	Low-quality	18.61
Sample_C13A_k141_112985	7594	10	1	0	Low-quality	18.59
Sample_T8C_k141_8856	7410	15	2	0	Low-quality	18.59
Sample_C13B_k141_37760	44203	34	1	25	Low-quality	18.58
Sample_T16B_k141_10725	12277	16	1	0	Low-quality	18.58
Sample_T7A_k141_93824	17446	17	0	3	Low-quality	18.58
Sample_C10C_k141_58021	8186	10	7	0	Low-quality	18.58
Sample_C2A_k141_33789_fragment_1	7559	12	5	0	Low-quality	18.58
Sample_C2C_k141_215680	7889	10	3	0	Low-quality	18.58
Sample_T17B_k141_26289	8287	5	4	0	Low-quality	18.57
Sample_C11A_k141_62577	20054	18	0	6	Low-quality	18.56
Sample_C14A_k141_102751	7907	14	6	0	Low-quality	18.56
Sample_T2C_k141_235971	8010	8	2	0	Low-quality	18.56
Sample_C6C_k141_89136	9252	12	5	0	Low-quality	18.55
Sample_T11B_k141_54552	37455	28	0	24	Low-quality	18.55
Sample_T11B_k141_13900	7746	12	1	0	Low-quality	18.55
Sample_T4C_k141_41100	16692	18	0	2	Low-quality	18.55
Sample_C12A_k141_168667	7066	16	6	0	Low-quality	18.55
Sample_C2B_k141_340289	10069	16	0	0	Low-quality	18.55
Sample_T17A_k141_11544	8395	13	5	0	Low-quality	18.55
Sample_T8C_k141_23708	6952	10	10	0	Low-quality	18.55
Sample_C11B_k141_166345	25715	31	1	4	Low-quality	18.53
Sample_C14C_k141_130951	40466	49	0	27	Low-quality	18.53
Sample_C2C_k141_49589	19016	10	0	8	Low-quality	18.53
Sample_T6B_k141_54134	12839	16	4	0	Low-quality	18.53
Sample_C4B_k141_236134	10099	7	3	0	Low-quality	18.52
Sample_C9A_k141_57077_fragment_1	16672	17	0	1	Low-quality	18.52
Sample_T8C_k141_50781	8234	11	0	3	Low-quality	18.51
Sample_C13C_k141_37916	220675	198	1	144	Low-quality	18.5

Sample_C3B_k141_167649	12225	13	1	1	Low-quality	18.5
Sample_C10A_k141_305126	6737	10	5	0	Low-quality	18.5
Sample_C4A_k141_112973	7850	12	4	0	Low-quality	18.5
Sample_T6B_k141_11857	6666	12	8	0	Low-quality	18.5
Sample_C2B_k141_346598	5919	16	2	0	Low-quality	18.49
Sample_T17B_k141_646	11063	14	0	6	Low-quality	18.48
Sample_T8B_k141_156328	7152	5	0	1	Low-quality	18.48
Sample_T2C_k141_203090	40035	41	1	28	Low-quality	18.47
Sample_C3A_k141_241605_fragment_1	16138	20	3	0	Low-quality	18.47
Sample_C3A_k141_95943	8600	11	5	0	Low-quality	18.47
Sample_T12A_k141_109922_fragment_1	8156	14	3	0	Low-quality	18.46
Sample_T17C_k141_18446	6445	8	4	0	Low-quality	18.46
Sample_T10B_k141_142038	8309	15	6	0	Low-quality	18.45
Sample_T15C_k141_19954	8579	8	0	2	Low-quality	18.45
Sample_T12A_k141_246707	7522	14	4	0	Low-quality	18.45
Sample_C1B_k141_166057	5924	13	1	0	Low-quality	18.44
Sample_C1B_k141_252152	62594	60	1	36	Low-quality	18.44
Sample_C6B_k141_73825	100977	83	1	58	Low-quality	18.44
Sample_T7A_k141_154703	27433	35	1	1	Low-quality	18.44
Sample_T5B_k141_11698	8562	11	0	10	Low-quality	18.43
Sample_C14C_k141_139705	14127	18	1	0	Low-quality	18.43
Sample_T6C_k141_50733	18533	19	3	0	Low-quality	18.43
Sample_T18B_k141_73503	9883	11	2	0	Low-quality	18.42
Sample_T11B_k141_42852	30422	28	1	21	Low-quality	18.41
Sample_T16C_k141_60556	18719	14	0	11	Low-quality	18.41
Sample_C11C_k141_181207	8669	16	3	0	Low-quality	18.41
Sample_T6B_k141_109451_fragment_1	20396	20	4	3	Low-quality	18.4
Sample_C8B_k141_75275	8273	17	5	0	Low-quality	18.39
Sample_C5B_k141_13419	8722	9	2	0	Low-quality	18.39
Sample_T12A_k141_160395	7291	14	1	0	Low-quality	18.38
Sample_C6B_k141_48527	28711	25	1	17	Low-quality	18.37
Sample_T12A_k141_325095	6617	10	5	0	Low-quality	18.37
Sample_T4C_k141_81207	12943	20	7	0	Low-quality	18.37
Sample_C7A_k141_50070	11616	14	8	0	Low-quality	18.36
Sample_T10A_k141_75311	6993	13	4	0	Low-quality	18.36
Sample_T15B_k141_124552	10985	13	1	2	Low-quality	18.35
Sample_C3A_k141_130930	29055	30	1	9	Low-quality	18.33
Sample_C8C_k141_57367	18384	16	0	4	Low-quality	18.33
Sample_T8B_k141_120692	12112	14	1	7	Low-quality	18.33

Sample_C8B_k141_150888	10564	24	1	2	Low-quality	18.33
Sample_T8C_k141_93558_fragment_1	8551	12	6	0	Low-quality	18.33
Sample_C3A_k141_67794	7429	8	0	0	Low-quality	18.32
Sample_T13B_k141_117285	9145	10	1	8	Low-quality	18.32
Sample_C13A_k141_53145	15463	21	5	0	Low-quality	18.32
Sample_C3C_k141_49601	7252	9	2	0	Low-quality	18.32
Sample_T2B_k141_183984	8165	8	2	0	Low-quality	18.32
Sample_T6C_k141_58182_fragment_1	11964	11	1	1	Low-quality	18.32
Sample_C6B_k141_8820	9743	4	0	0	Low-quality	18.31
Sample_T12A_k141_257086	7721	3	0	2	Low-quality	18.31
Sample_T18A_k141_77016	7241	16	6	0	Low-quality	18.31
Sample_T4B_k141_275852	7220	16	1	0	Low-quality	18.31
Sample_T8A_k141_75637	8989	16	1	0	Low-quality	18.31
Sample_T12A_k141_14734	14046	13	0	1	Low-quality	18.3
Sample_T13A_k141_99776	11763	16	0	8	Low-quality	18.3
Sample_T4C_k141_88814	9138	19	1	4	Low-quality	18.3
Sample_T16C_k141_291	7834	11	7	0	Low-quality	18.3
Sample_T2C_k141_22601	6190	7	4	0	Low-quality	18.3
Sample_C4C_k141_220295	7520	12	3	0	Low-quality	18.29
Sample_T8A_k141_5033	14835	23	1	5	Low-quality	18.28
Sample_T8B_k141_91689	6415	14	11	0	Low-quality	18.28
Sample_C10B_k141_76544	13599	16	1	6	Low-quality	18.27
Sample_C7B_k141_11099	8633	16	1	2	Low-quality	18.27
Sample_C14A_k141_112331	8472	9	3	0	Low-quality	18.27
Sample_C6B_k141_203093	6979	8	5	0	Low-quality	18.27
Sample_C6C_k141_98001	9641	13	3	0	Low-quality	18.27
Sample_C10A_k141_58424	16658	11	0	10	Low-quality	18.25
Sample_C11C_k141_151918	6373	13	1	1	Low-quality	18.25
Sample_C12B_k141_39507	6964	9	2	1	Low-quality	18.25
Sample_C12C_k141_219221	7066	13	2	1	Low-quality	18.25
Sample_C3A_k141_56574	14801	15	6	1	Low-quality	18.25
Sample_C9C_k141_65758	8772	8	0	5	Low-quality	18.24
Sample_C5A_k141_149031	8128	12	11	0	Low-quality	18.24
Sample_T3B_k141_90116	11631	20	4	0	Low-quality	18.24
Sample_C11A_k141_9999	9241	12	0	5	Low-quality	18.23
Sample_C2C_k141_69094	27393	23	1	14	Low-quality	18.23
Sample_T12A_k141_64947	6714	8	0	5	Low-quality	18.23
Sample_C1A_k141_16147	10089	13	6	0	Low-quality	18.23
Sample_T6B_k141_27529	7097	15	1	0	Low-quality	18.23

Sample_C10A_k141_207755	26290	22	0	7	Low-quality	18.22
Sample_C12C_k141_172629	47744	49	1	25	Low-quality	18.22
Sample_C1A_k141_58261	16417	15	0	12	Low-quality	18.22
Sample_T2A_k141_31717	16202	14	0	4	Low-quality	18.22
Sample_C1A_k141_5512	7985	16	4	0	Low-quality	18.22
Sample_C3A_k141_168519	8316	11	5	0	Low-quality	18.22
Sample_T14C_k141_143535	7095	13	1	0	Low-quality	18.22
Sample_T6C_k141_149014	7728	11	5	0	Low-quality	18.22
Sample_C1C_k141_184481	6952	12	1	1	Low-quality	18.21
Sample_C5B_k141_85440	6803	4	2	0	Low-quality	18.2
Sample_T7A_k141_122327	9055	16	0	1	Low-quality	18.2
Sample_C5C_k141_14962	7243	8	5	0	Low-quality	18.19
Sample_T6C_k141_228849	8892	14	2	0	Low-quality	18.19
Sample_T4B_k141_183730	6071	4	3	0	Low-quality	18.18
Sample_T12A_k141_133427	13460	19	3	3	Low-quality	18.17
Sample_C6B_k141_69117	8113	7	0	1	Low-quality	18.16
Sample_T11C_k141_111530	38567	31	2	4	Low-quality	18.16
Sample_C3C_k141_82010	10279	19	5	2	Low-quality	18.16
Sample_T13B_k141_184399	6730	8	0	0	Low-quality	18.15
Sample_T8B_k141_239031	7216	8	1	0	Low-quality	18.15
Sample_C10A_k141_19478	32375	33	1	10	Low-quality	18.14
Sample_C8B_k141_118927	7974	7	1	2	Low-quality	18.14
Sample_T12C_k141_133461	30191	32	0	12	Low-quality	18.14
Sample_T7A_k141_24220	6909	5	0	5	Low-quality	18.14
Sample_C2C_k141_303564	8615	14	10	0	Low-quality	18.14
Sample_C8C_k141_33278	14356	11	0	2	Low-quality	18.13
Sample_C11C_k141_655	8104	9	7	0	Low-quality	18.13
Sample_C13A_k141_31027	12260	16	1	1	Low-quality	18.13
Sample_C11B_k141_33710	30102	35	9	9	Low-quality	18.12
Sample_C12C_k141_193865	11096	17	3	1	Low-quality	18.12
Sample_T3A_k141_7482	20928	25	0	4	Low-quality	18.12
Sample_C12A_k141_179641	7181	9	0	0	Low-quality	18.12
Sample_C1A_k141_246673	10593	21	2	0	Low-quality	18.12
Sample_T12B_k141_166369	9716	22	5	0	Low-quality	18.12
Sample_T16B_k141_82067	18078	28	2	0	Low-quality	18.12
Sample_T16A_k141_1552	31744	42	4	2	Low-quality	18.11
Sample_C5A_k141_50413	9714	13	2	0	Low-quality	18.11
Sample_T13A_k141_97864	8408	14	10	0	Low-quality	18.11
Sample_C10C_k141_208958	6969	6	3	0	Low-quality	18.1

Sample_C12A_k141_124610	6595	6	4	0	Low-quality	18.1
Sample_T3A_k141_101752	6883	14	13	0	Low-quality	18.1
Sample_T14A_k141_204256	11955	15	1	3	Low-quality	18.09
Sample_C1B_k141_237133	6448	11	0	0	Low-quality	18.09
Sample_C2A_k141_246990	8501	12	7	1	Low-quality	18.09
Sample_T16B_k141_117560	6952	13	2	0	Low-quality	18.09
Sample_T2C_k141_37396	40846	48	1	29	Low-quality	18.08
Sample_T4B_k141_339787	6850	13	7	0	Low-quality	18.08
Sample_T4C_k141_35655	8917	14	9	0	Low-quality	18.08
Sample_C8A_k141_95728	13860	14	0	1	Low-quality	18.07
Sample_T16C_k141_11060	7996	11	4	0	Low-quality	18.07
Sample_T6A_k141_268722	9660	14	10	0	Low-quality	18.07
Sample_C2A_k141_263609	15882	17	2	1	Low-quality	18.06
Sample_C14A_k141_496	7327	10	5	0	Low-quality	18.06
Sample_C6C_k141_151186	17625	13	0	10	Low-quality	18.05
Sample_T2B_k141_95629	145402	128	2	107	Low-quality	18.05
Sample_T11C_k141_45258	19544	36	7	0	Low-quality	18.05
Sample_T3B_k141_121043	11248	30	3	1	Low-quality	18.05
Sample_T10B_k141_80919	17853	13	0	6	Low-quality	18.04
Sample_T5B_k141_77572	19483	25	0	4	Low-quality	18.04
Sample_T4B_k141_320946	6833	19	7	0	Low-quality	18.03
Sample_T10B_k141_147383	11440	13	0	4	Low-quality	18.02
Sample_T5B_k141_23090	27964	20	1	16	Low-quality	18
Sample_C10A_k141_115272	8179	12	4	0	Low-quality	17.99
Sample_C3A_k141_172804	8225	9	0	0	Low-quality	17.99
Sample_T15C_k141_103168	6698	11	2	0	Low-quality	17.99
Sample_C7C_k141_11597	13138	11	1	1	Low-quality	17.97
Sample_T3A_k141_17537	10666	10	0	3	Low-quality	17.97
Sample_T2B_k141_13958	6016	7	4	0	Low-quality	17.97
Sample_C11A_k141_63100	19715	23	1	5	Low-quality	17.95
Sample_T11B_k141_84804	26148	51	9	5	Low-quality	17.95
Sample_C3A_k141_216058_fragment_1	19122	26	6	2	Low-quality	17.94
Sample_T14B_k141_116394	12236	9	1	2	Low-quality	17.94
Sample_T16B_k141_90279	9308	6	1	0	Low-quality	17.94
Sample_T7C_k141_27582	16449	16	0	11	Low-quality	17.93
Sample_T8C_k141_16115	10732	13	0	0	Low-quality	17.93
Sample_C10B_k141_13094_fragment_1	25311	26	2	4	Low-quality	17.93
Sample_T4B_k141_262362	7578	13	4	0	Low-quality	17.93
Sample_C10A_k141_84805	9388	13	2	1	Low-quality	17.92

Sample_T4B_k141_151096	7039	11	3	0	Low-quality	17.92
Sample_T5C_k141_63900	10503	18	3	0	Low-quality	17.92
Sample_C12A_k141_73626	9573	9	3	1	Low-quality	17.91
Sample_T5B_k141_32950	7494	12	2	0	Low-quality	17.91
Sample_C10A_k141_77048	21230	31	2	2	Low-quality	17.9
Sample_T5C_k141_132587	7623	9	0	2	Low-quality	17.9
Sample_C5A_k141_192226	8358	11	6	0	Low-quality	17.9
Sample_T17B_k141_18471	8456	9	3	0	Low-quality	17.9
Sample_C4C_k141_96300	5948	7	0	5	Low-quality	17.89
Sample_C1C_k141_161500	8657	9	1	0	Low-quality	17.89
Sample_C11C_k141_121873	17804	16	0	6	Low-quality	17.88
Sample_T16A_k141_29033	23433	25	1	9	Low-quality	17.88
Sample_C1B_k141_260303	11762	22	2	0	Low-quality	17.88
Sample_C2B_k141_15079_fragment_1	9451	17	4	2	Low-quality	17.88
Sample_T15C_k141_128793_fragment_1	8527	15	3	0	Low-quality	17.88
Sample_T14A_k141_52922	6412	9	6	0	Low-quality	17.86
Sample_C4B_k141_144223	5122	3	0	1	Low-quality	17.85
Sample_T14B_k141_47446	10686	14	0	1	Low-quality	17.85
Sample_T3B_k141_153027_fragment_1	7653	9	0	0	Low-quality	17.85
Sample_C11A_k141_147038	32300	73	4	1	Low-quality	17.84
Sample_C11B_k141_149128	8154	12	8	0	Low-quality	17.84
Sample_T2C_k141_48393	6651	13	1	0	Low-quality	17.84
Sample_C10B_k141_138077	6503	5	1	0	Low-quality	17.83
Sample_C1C_k141_44760	7472	9	3	0	Low-quality	17.83
Sample_C3A_k141_173538_fragment_1	22284	18	1	3	Low-quality	17.83
Sample_T17A_k141_1949	7003	12	12	0	Low-quality	17.82
Sample_T2B_k141_148774	7648	6	2	0	Low-quality	17.82
Sample_T5C_k141_169664	8006	16	5	0	Low-quality	17.82
Sample_C10B_k141_100594	23870	25	1	20	Low-quality	17.81
Sample_T14A_k141_232112	7416	4	1	0	Low-quality	17.81
Sample_T15C_k141_126828	7101	10	1	0	Low-quality	17.8
Sample_T17B_k141_31554	23183	16	0	16	Low-quality	17.8
Sample_C2A_k141_249383	6453	9	6	0	Low-quality	17.8
Sample_C2A_k141_1284	14197	24	2	1	Low-quality	17.8
Sample_C3C_k141_113957	25423	48	3	1	Low-quality	17.8
Sample_T6B_k141_144238	10544	6	0	0	Low-quality	17.79
Sample_C8B_k141_196703	10919	20	3	0	Low-quality	17.79
Sample_T12C_k141_144280_fragment_1	6705	11	1	0	Low-quality	17.79
Sample_T10A_k141_178754	23767	26	6	2	Low-quality	17.78

Sample_T8B_k141_105371	7471	7	0	4	Low-quality	17.78
Sample_C10A_k141_209114	8760	16	2	0	Low-quality	17.78
Sample_C13A_k141_111433	7173	8	1	1	Low-quality	17.78
Sample_C1C_k141_60337	18309	14	0	4	Low-quality	17.77
Sample_T17C_k141_12670	49909	34	1	24	Low-quality	17.77
Sample_T4B_k141_72262	6786	7	0	3	Low-quality	17.77
Sample_C3A_k141_182167	28296	37	6	2	Low-quality	17.77
Sample_C9A_k141_29795	38875	45	3	3	Low-quality	17.77
Sample_T12B_k141_166463	35337	76	4	2	Low-quality	17.76
Sample_T2B_k141_262902	37674	27	1	20	Low-quality	17.76
Sample_T6B_k141_88831	32227	38	4	1	Low-quality	17.76
Sample_C1B_k141_136487	7474	7	3	0	Low-quality	17.76
Sample_T4B_k141_293222	9270	13	2	0	Low-quality	17.76
Sample_C10C_k141_159426	9785	15	0	3	Low-quality	17.75
Sample_T14B_k141_99522_fragment_1	14061	23	6	0	Low-quality	17.75
Sample_C3A_k141_4850	8394	9	0	1	Low-quality	17.74
Sample_C3B_k141_189379	5682	6	0	3	Low-quality	17.74
Sample_T13C_k141_123705	12936	13	2	2	Low-quality	17.74
Sample_T5A_k141_18362	7238	7	0	1	Low-quality	17.74
Sample_C10C_k141_8023_fragment_1	11134	10	3	2	Low-quality	17.73
Sample_C3C_k141_234389	8513	13	6	0	Low-quality	17.73
Sample_C8C_k141_41767	7180	11	6	0	Low-quality	17.73
Sample_T8C_k141_184566	6239	9	4	0	Low-quality	17.73
Sample_T12A_k141_233224	13822	4	2	0	Low-quality	17.72
Sample_T12A_k141_168575	38348	35	1	24	Low-quality	17.72
Sample_C12C_k141_3100	7536	10	6	0	Low-quality	17.72
Sample_C14A_k141_218326	8602	13	1	0	Low-quality	17.72
Sample_T2B_k141_147705	8300	13	9	0	Low-quality	17.72
Sample_C2B_k141_98041	31613	33	1	12	Low-quality	17.71
Sample_C9B_k141_6752	9054	10	8	1	Low-quality	17.71
Sample_T6C_k141_173514	7269	13	9	0	Low-quality	17.71
Sample_T15C_k141_141171	14462	16	0	2	Low-quality	17.7
Sample_T4B_k141_231096	6514	10	4	0	Low-quality	17.7
Sample_C6B_k141_170904	17371	16	3	5	Low-quality	17.69
Sample_T6B_k141_12814	34263	27	1	6	Low-quality	17.69
Sample_T17B_k141_28955	6940	12	9	0	Low-quality	17.69
Sample_T17B_k141_10112	14291	31	1	1	Low-quality	17.69
Sample_T6A_k141_172159	7239	10	2	0	Low-quality	17.69
Sample_T5C_k141_136782	30857	28	0	20	Low-quality	17.68

Sample_C2A_k141_114192	11384	14	3	1	Low-quality	17.67
Sample_T11C_k141_111801	11836	23	2	1	Low-quality	17.67
Sample_T14A_k141_35644_fragment_1	9656	12	0	2	Low-quality	17.66
Sample_T8C_k141_167792	7088	12	0	0	Low-quality	17.66
Sample_C6A_k141_106492	14528	14	0	3	Low-quality	17.65
Sample_C9A_k141_68035	7441	10	5	0	Low-quality	17.64
Sample_C10C_k141_121628	7255	13	0	0	Low-quality	17.62
Sample_T11C_k141_18222	9648	8	1	3	Low-quality	17.62
Sample_T5C_k141_154013	27224	29	1	14	Low-quality	17.62
Sample_T6A_k141_36125	6109	7	0	2	Low-quality	17.62
Sample_C2A_k141_130295	7025	12	8	0	Low-quality	17.62
Sample_C1B_k141_168123	13652	5	0	2	Low-quality	17.61
Sample_T6A_k141_42075	16893	20	1	8	Low-quality	17.61
Sample_C6C_k141_210997	14382	13	0	10	Low-quality	17.6
Sample_T4B_k141_111845	10344	10	1	0	Low-quality	17.6
Sample_C5A_k141_284994	12414	13	0	3	Low-quality	17.59
Sample_C13C_k141_68373	6805	16	1	0	Low-quality	17.59
Sample_T18A_k141_47670	7151	11	6	0	Low-quality	17.59
Sample_C5B_k141_21922	7655	10	0	1	Low-quality	17.58
Sample_C6B_k141_206955	10312	13	0	3	Low-quality	17.58
Sample_T10B_k141_101772	7387	8	0	4	Low-quality	17.58
Sample_T7C_k141_72787_fragment_1	8344	15	2	0	Low-quality	17.58
Sample_C5A_k141_258047	22926	31	1	25	Low-quality	17.57
Sample_C1C_k141_47847	18909	22	0	3	Low-quality	17.56
Sample_T6B_k141_114822	8163	7	0	1	Low-quality	17.56
Sample_C3B_k141_249270	10146	31	1	0	Low-quality	17.56
Sample_T12B_k141_46528	10415	18	6	0	Low-quality	17.56
Sample_T16B_k141_159383	17373	11	0	6	Low-quality	17.55
Sample_T3B_k141_119984	8759	17	1	3	Low-quality	17.55
Sample_C2A_k141_135771	6139	7	6	0	Low-quality	17.55
Sample_T11C_k141_12545	7293	10	4	0	Low-quality	17.55
Sample_T3B_k141_8532	7526	13	0	1	Low-quality	17.55
Sample_C3A_k141_241860_fragment_1	7313	8	2	0	Low-quality	17.54
Sample_C4B_k141_126326	7753	18	2	1	Low-quality	17.53
Sample_C11C_k141_104358	10453	8	0	2	Low-quality	17.52
Sample_C3A_k141_697	14155	17	1	6	Low-quality	17.52
Sample_C6C_k141_235463	28940	31	1	15	Low-quality	17.52
Sample_C8C_k141_162361	15590	20	2	8	Low-quality	17.52
Sample_T16C_k141_24030	5751	9	3	0	Low-quality	17.52

Sample_T6B_k141_100490	6278	5	4	0	Low-quality	17.52
Sample_T5C_k141_69959	26053	30	1	5	Low-quality	17.51
Sample_C4A_k141_64230_fragment_2	11704	19	1	0	Low-quality	17.51
Sample_C7C_k141_51301	11879	12	1	3	Low-quality	17.51
Sample_C7C_k141_21048	11404	14	4	0	Low-quality	17.5
Sample_C8C_k141_92655	38119	40	0	28	Low-quality	17.5
Sample_T3C_k141_35124	15180	16	3	1	Low-quality	17.5
Sample_C13B_k141_113319	7027	6	3	0	Low-quality	17.5
Sample_C1A_k141_94416	7063	5	3	0	Low-quality	17.5
Sample_T16B_k141_166509	6936	11	6	0	Low-quality	17.5
Sample_C9B_k141_18857	7398	9	1	0	Low-quality	17.49
Sample_C1B_k141_276023	6977	11	5	0	Low-quality	17.49
Sample_C1B_k141_216389	7397	14	5	0	Low-quality	17.49
Sample_C2B_k141_184443	40829	48	0	16	Low-quality	17.48
Sample_C5A_k141_265731	9383	4	0	1	Low-quality	17.48
Sample_T2A_k141_66276	6668	6	1	1	Low-quality	17.47
Sample_T13A_k141_27702	6646	10	3	0	Low-quality	17.47
Sample_T14B_k141_124196	6226	9	2	0	Low-quality	17.47
Sample_T8A_k141_228148	31804	24	2	6	Low-quality	17.47
Sample_C10A_k141_292340	12731	8	0	1	Low-quality	17.46
Sample_C13B_k141_55584	31297	35	1	7	Low-quality	17.46
Sample_T12B_k141_147159	11966	12	0	1	Low-quality	17.46
Sample_T14A_k141_171100	25492	18	1	9	Low-quality	17.46
Sample_C4C_k141_59524	10335	15	3	0	Low-quality	17.46
Sample_T15B_k141_91020	6748	10	0	3	Low-quality	17.45
Sample_C10A_k141_288892	13250	13	2	1	Low-quality	17.45
Sample_T4B_k141_203951_fragment_1	18388	15	0	5	Low-quality	17.45
Sample_C1C_k141_98692	30445	24	0	21	Low-quality	17.44
Sample_C3A_k141_226116	18265	33	6	0	Low-quality	17.44
Sample_T7C_k141_94407	6707	4	4	0	Low-quality	17.44
Sample_C4B_k141_171174	5841	9	1	4	Low-quality	17.43
Sample_C10C_k141_82332	6387	9	2	0	Low-quality	17.43
Sample_C8A_k141_27029	7616	19	2	1	Low-quality	17.43
Sample_T12A_k141_270866	5785	8	1	0	Low-quality	17.43
Sample_T2B_k141_204858	26162	45	7	2	Low-quality	17.42
Sample_T11B_k141_51164	26905	29	1	11	Low-quality	17.41
Sample_C14C_k141_46911	6889	8	1	0	Low-quality	17.41
Sample_C9B_k141_66220	9523	12	1	1	Low-quality	17.41
Sample_T17A_k141_39406	6038	5	4	0	Low-quality	17.41

Sample_T7C_k141_70745	7844	13	6	0	Low-quality	17.41
Sample_C5C_k141_88862	6737	9	5	0	Low-quality	17.4
Sample_T4B_k141_37283	6459	16	0	0	Low-quality	17.4
Sample_T8B_k141_262111	5751	9	0	0	Low-quality	17.4
Sample_C8B_k141_145894	13328	12	0	1	Low-quality	17.39
Sample_C3A_k141_185485	7531	13	0	0	Low-quality	17.39
Sample_T17A_k141_38990	6367	13	2	1	Low-quality	17.38
Sample_C3B_k141_220662	10011	12	4	0	Low-quality	17.38
Sample_C8C_k141_71817	15894	23	1	2	Low-quality	17.37
Sample_T16A_k141_63354	8905	8	0	3	Low-quality	17.37
Sample_T10A_k141_123720	5909	4	4	0	Low-quality	17.37
Sample_T4A_k141_73459	10151	21	4	0	Low-quality	17.37
Sample_T11C_k141_18613	7296	9	0	5	Low-quality	17.36
Sample_C7B_k141_84511	6564	10	4	0	Low-quality	17.36
Sample_T8C_k141_185659	11360	16	3	0	Low-quality	17.36
Sample_T5C_k141_133675	7868	10	1	0	Low-quality	17.35
Sample_T15B_k141_97490	9033	15	5	0	Low-quality	17.35
Sample_C3A_k141_188621	6360	10	2	0	Low-quality	17.34
Sample_T10A_k141_151204	22627	14	1	2	Low-quality	17.34
Sample_T8C_k141_13188	6069	5	4	0	Low-quality	17.34
Sample_C4A_k141_237449	15822	10	0	2	Low-quality	17.33
Sample_C5A_k141_165909	14150	18	2	3	Low-quality	17.33
Sample_T14B_k141_116267	18251	17	0	14	Low-quality	17.33
Sample_T17C_k141_31240	7953	7	0	3	Low-quality	17.33
Sample_C13A_k141_13276	5854	14	0	0	Low-quality	17.33
Sample_T15B_k141_75942	15040	15	0	1	Low-quality	17.32
Sample_C2A_k141_109122	10361	14	0	3	Low-quality	17.31
Sample_T12A_k141_284391_fragment_1	7806	16	4	1	Low-quality	17.31
Sample_C14B_k141_89000	21631	21	0	4	Low-quality	17.3
Sample_C2C_k141_224914	205776	180	2	105	Low-quality	17.3
Sample_C10C_k141_205096	7333	12	1	0	Low-quality	17.29
Sample_T18B_k141_123031	10354	14	0	4	Low-quality	17.29
Sample_C13B_k141_67427	16373	26	2	0	Low-quality	17.29
Sample_C4B_k141_252323	8811	11	3	0	Low-quality	17.29
Sample_C10C_k141_203359	10343	9	0	1	Low-quality	17.28
Sample_T14B_k141_12461	45112	44	1	29	Low-quality	17.28
Sample_C5A_k141_191010	18085	13	0	3	Low-quality	17.27
Sample_T14B_k141_140127	7260	7	0	5	Low-quality	17.27
Sample_T17B_k141_3740	14966	15	1	9	Low-quality	17.27

Sample_C3B_k141_114410	8556	20	3	0	Low-quality	17.27
Sample_T4B_k141_105271	7028	12	5	0	Low-quality	17.27
Sample_T6C_k141_32247	6572	8	3	0	Low-quality	17.27
Sample_T10C_k141_51978	31823	31	1	8	Low-quality	17.26
Sample_T6C_k141_80300	9769	10	2	1	Low-quality	17.26
Sample_C5A_k141_30504	26660	26	1	7	Low-quality	17.25
Sample_T6B_k141_176144	16929	14	0	3	Low-quality	17.25
Sample_T7C_k141_107278	8019	7	0	2	Low-quality	17.25
Sample_T8A_k141_256935	6836	5	5	0	Low-quality	17.25
Sample_C11B_k141_34657	14275	15	2	2	Low-quality	17.24
Sample_C7C_k141_46610	11392	9	1	0	Low-quality	17.24
Sample_T3A_k141_15832	10665	14	0	4	Low-quality	17.24
Sample_T8C_k141_28396	10631	16	4	2	Low-quality	17.24
Sample_T10A_k141_44760	10310	16	0	2	Low-quality	17.22
Sample_T2A_k141_103820	6946	7	6	0	Low-quality	17.22
Sample_C11C_k141_115248	18994	24	0	7	Low-quality	17.21
Sample_C11C_k141_44213	7906	8	0	3	Low-quality	17.21
Sample_C4B_k141_28965	7708	7	0	4	Low-quality	17.2
Sample_C9A_k141_37280	94196	80	1	51	Low-quality	17.2
Sample_T2A_k141_17902	7120	11	2	1	Low-quality	17.2
Sample_C2C_k141_237735	14437	18	1	6	Low-quality	17.19
Sample_T6A_k141_177560	7255	19	2	0	Low-quality	17.19
Sample_T14B_k141_129649	7339	11	1	0	Low-quality	17.18
Sample_T8C_k141_135062	6444	10	1	0	Low-quality	17.18
Sample_C1C_k141_98097	26673	19	1	16	Low-quality	17.17
Sample_T8B_k141_199371	8728	10	1	0	Low-quality	17.17
Sample_C10C_k141_9321	7080	8	3	0	Low-quality	17.17
Sample_C4B_k141_254199	6016	11	4	0	Low-quality	17.17
Sample_T10A_k141_138058	6768	20	2	0	Low-quality	17.17
Sample_T12A_k141_34759	6042	9	1	0	Low-quality	17.17
Sample_T12B_k141_9179	6635	5	0	1	Low-quality	17.16
Sample_T13A_k141_105153	9095	14	2	0	Low-quality	17.16
Sample_T4B_k141_151095	6855	10	3	0	Low-quality	17.16
Sample_T6A_k141_104253	8476	17	1	0	Low-quality	17.16
Sample_C11C_k141_105022	6336	6	0	0	Low-quality	17.15
Sample_C5A_k141_87830	41655	40	1	12	Low-quality	17.15
Sample_T10B_k141_158441	7753	9	8	0	Low-quality	17.15
Sample_T12A_k141_58449	9363	10	1	0	Low-quality	17.15
Sample_T2A_k141_42357	16624	12	0	2	Low-quality	17.14

Sample_C2B_k141_312599	18041	12	0	11	Low-quality	17.13
Sample_C8A_k141_111527	18806	17	1	4	Low-quality	17.13
Sample_T18A_k141_190423	9357	12	9	1	Low-quality	17.13
Sample_T15B_k141_115152	8529	13	7	0	Low-quality	17.13
Sample_C10C_k141_185792	7173	9	1	0	Low-quality	17.11
Sample_C5A_k141_18945	8038	9	5	0	Low-quality	17.11
Sample_T12A_k141_319127_fragment_2	6049	9	2	1	Low-quality	17.11
Sample_T17C_k141_10423	12868	10	0	2	Low-quality	17.11
Sample_C10B_k141_132001	11300	16	1	4	Low-quality	17.1
Sample_C11C_k141_39559	8052	10	4	0	Low-quality	17.1
Sample_C10C_k141_59813	6078	7	4	0	Low-quality	17.09
Sample_C14A_k141_155993	5795	8	5	0	Low-quality	17.09
Sample_T10C_k141_44274	22888	55	4	0	Low-quality	17.09
Sample_T4A_k141_99845	6409	17	0	0	Low-quality	17.09
Sample_C4A_k141_5475_fragment_2	7654	9	1	2	Low-quality	17.08
Sample_C8C_k141_1205	12200	12	1	1	Low-quality	17.08
Sample_C8C_k141_109540	7147	3	0	1	Low-quality	17.07
Sample_C10B_k141_3691	26590	38	8	2	Low-quality	17.06
Sample_T10A_k141_105803	6575	15	8	0	Low-quality	17.06
Sample_T18C_k141_66346	31443	34	1	8	Low-quality	17.05
Sample_C6A_k141_88842	6386	8	3	0	Low-quality	17.05
Sample_C7B_k141_171669	6206	10	7	0	Low-quality	17.05
Sample_T17C_k141_11498	5859	8	3	0	Low-quality	17.05
Sample_T13C_k141_118335	10798	7	0	2	Low-quality	17.04
Sample_T14B_k141_157294	6560	5	0	1	Low-quality	17.04
Sample_C4B_k141_215184	17835	15	0	12	Low-quality	17.03
Sample_T13A_k141_26599	19403	19	1	5	Low-quality	17.03
Sample_T16C_k141_65323	10171	10	6	0	Low-quality	17.03
Sample_C1B_k141_184696	11249	13	1	1	Low-quality	17.02
Sample_T2B_k141_170925	55550	56	2	30	Low-quality	17.02
Sample_T10A_k141_82739	7081	9	2	0	Low-quality	17.02
Sample_C12B_k141_11493	31364	35	1	5	Low-quality	17.01
Sample_C14A_k141_71153	24268	37	7	0	Low-quality	17.01
Sample_C4B_k141_147956	6821	8	4	0	Low-quality	17.01
Sample_C12A_k141_63768	5382	6	1	0	Low-quality	17
Sample_C12B_k141_174630	5901	6	3	0	Low-quality	17
Sample_T15A_k141_6098	25280	27	1	6	Low-quality	16.99
Sample_C4B_k141_21846	6792	16	2	2	Low-quality	16.99
Sample_T18C_k141_66724	5810	18	1	0	Low-quality	16.99

Sample_C14C_k141_81185	8773	7	6	0	Low-quality	16.98
Sample_T4C_k141_74358	6276	4	3	0	Low-quality	16.98
Sample_C1B_k141_568	14182	12	0	9	Low-quality	16.97
Sample_T13C_k141_145307	13969	12	0	0	Low-quality	16.97
Sample_C3B_k141_185278	7508	7	0	0	Low-quality	16.97
Sample_T5B_k141_69178	10986	13	6	2	Low-quality	16.97
Sample_C14C_k141_38886	27034	29	1	8	Low-quality	16.96
Sample_T15C_k141_62908	36758	34	0	6	Low-quality	16.96
Sample_C1B_k141_63991	6908	14	4	0	Low-quality	16.96
Sample_T2A_k141_63362	6493	6	0	6	Low-quality	16.95
Sample_T8B_k141_22305_fragment_1	13953	16	3	0	Low-quality	16.95
Sample_C13B_k141_30164	13634	17	2	2	Low-quality	16.94
Sample_C6B_k141_137707	10036	12	1	1	Low-quality	16.94
Sample_T2B_k141_175989	14660	12	0	3	Low-quality	16.94
Sample_C10C_k141_103111	9473	21	1	2	Low-quality	16.94
Sample_C8C_k141_83002	16945	15	1	3	Low-quality	16.93
Sample_C8B_k141_64906	6094	9	8	0	Low-quality	16.93
Sample_C8C_k141_123419	7713	7	2	0	Low-quality	16.93
Sample_C2A_k141_173436	8681	5	0	3	Low-quality	16.92
Sample_C9B_k141_25102	18234	20	0	6	Low-quality	16.92
Sample_T5B_k141_6145	16563	14	0	3	Low-quality	16.92
Sample_C10C_k141_12952	10272	22	0	0	Low-quality	16.92
Sample_T16B_k141_131139	6353	6	1	0	Low-quality	16.92
Sample_T17B_k141_3920	5719	8	8	0	Low-quality	16.92
Sample_T8C_k141_23475	10130	15	11	0	Low-quality	16.92
Sample_C8B_k141_4056	10126	10	0	4	Low-quality	16.91
Sample_C2C_k141_261419	92035	96	1	65	Low-quality	16.89
Sample_C6B_k141_38222	9918	13	0	4	Low-quality	16.89
Sample_T6A_k141_251962	12740	13	2	3	Low-quality	16.89
Sample_C10A_k141_176086	6440	15	1	0	Low-quality	16.89
Sample_C10A_k141_107200	6908	18	2	0	Low-quality	16.89
Sample_C3A_k141_158658	5583	8	3	0	Low-quality	16.89
Sample_C6C_k141_220214	7483	8	2	0	Low-quality	16.89
Sample_T10A_k141_173583	6088	8	5	0	Low-quality	16.89
Sample_C11C_k141_183937	8070	12	1	0	Low-quality	16.88
Sample_T8A_k141_22071	10231	18	4	3	Low-quality	16.88
Sample_T14A_k141_35856	28195	40	2	0	Low-quality	16.87
Sample_C10A_k141_15227	7708	10	4	0	Low-quality	16.87
Sample_T15A_k141_26025	11146	14	1	1	Low-quality	16.87

Sample_T6A_k141_25635	11152	12	1	1	Low-quality	16.87
Sample_T8B_k141_225604	6997	9	4	0	Low-quality	16.87
Sample_T6A_k141_49040	9388	7	0	5	Low-quality	16.86
Sample_T8A_k141_133350	18325	24	0	6	Low-quality	16.86
Sample_C2B_k141_111370	7074	8	1	0	Low-quality	16.86
Sample_C14B_k141_33535	20559	20	1	13	Low-quality	16.85
Sample_T4B_k141_40202	35038	28	0	21	Low-quality	16.85
Sample_T2A_k141_93136	6116	10	1	0	Low-quality	16.85
Sample_C8A_k141_20619	7074	13	2	0	Low-quality	16.84
Sample_C13C_k141_78326	5692	8	6	0	Low-quality	16.84
Sample_C4C_k141_70296	7984	9	2	0	Low-quality	16.82
Sample_T12B_k141_50416	15135	9	0	1	Low-quality	16.82
Sample_C13C_k141_34250	9729	9	1	1	Low-quality	16.81
Sample_C2A_k141_44214	13548	23	7	1	Low-quality	16.81
Sample_C6B_k141_10040	6872	10	6	0	Low-quality	16.8
Sample_C6C_k141_151481	5764	9	6	0	Low-quality	16.8
Sample_C11B_k141_245701	9675	8	0	1	Low-quality	16.79
Sample_T14A_k141_32934	13210	4	2	1	Low-quality	16.79
Sample_C10A_k141_168229	6586	8	1	0	Low-quality	16.79
Sample_T8C_k141_66841_fragment_2	18602	20	1	3	Low-quality	16.79
Sample_C3A_k141_153346	6288	8	2	0	Low-quality	16.78
Sample_T11B_k141_69093	6277	11	2	1	Low-quality	16.78
Sample_T13B_k141_53437	15849	20	7	2	Low-quality	16.78
Sample_C11A_k141_75900	12834	25	7	0	Low-quality	16.77
Sample_T2C_k141_110131_fragment_3	11479	18	3	3	Low-quality	16.77
Sample_T5A_k141_16270	26465	35	1	3	Low-quality	16.77
Sample_T1A_k141_31478	10917	11	0	3	Low-quality	16.76
Sample_T14C_k141_61904	7598	12	1	0	Low-quality	16.75
Sample_T2B_k141_69144	6487	5	0	1	Low-quality	16.75
Sample_T7A_k141_9149	62482	81	2	36	Low-quality	16.75
Sample_C4B_k141_213381	7566	11	0	1	Low-quality	16.75
Sample_T13B_k141_180753	6603	12	6	0	Low-quality	16.75
Sample_T7A_k141_205337	7024	8	7	0	Low-quality	16.75
Sample_T7C_k141_30451	9998	16	3	0	Low-quality	16.75
Sample_C1A_k141_185505	8167	13	1	0	Low-quality	16.74
Sample_T11B_k141_6794	13783	18	0	1	Low-quality	16.74
Sample_C10A_k141_87025_fragment_1	8316	16	3	0	Low-quality	16.74
Sample_C6A_k141_173314	8955	12	4	0	Low-quality	16.73
Sample_T15B_k141_92917	7051	13	2	0	Low-quality	16.73

Sample_T4A_k141_50160	8809	11	6	1	Low-quality	16.73
Sample_T8B_k141_237826	6291	11	5	0	Low-quality	16.73
Sample_T14B_k141_62348	6715	6	0	2	Low-quality	16.72
Sample_C10A_k141_33124	6842	14	2	0	Low-quality	16.72
Sample_T10B_k141_1703	10031	18	2	0	Low-quality	16.72
Sample_C10C_k141_40639	8890	7	0	2	Low-quality	16.71
Sample_T14C_k141_40561	7151	7	0	3	Low-quality	16.71
Sample_T18B_k141_112907	18850	14	0	6	Low-quality	16.71
Sample_C8C_k141_152233	15683	19	0	0	Low-quality	16.71
Sample_T6C_k141_79390	16032	33	3	0	Low-quality	16.71
Sample_C10A_k141_328877	9227	10	0	3	Low-quality	16.7
Sample_C13B_k141_65269	8867	11	0	4	Low-quality	16.7
Sample_C14C_k141_32320	7481	4	0	4	Low-quality	16.7
Sample_C3C_k141_58497	11035	12	1	3	Low-quality	16.7
Sample_T10A_k141_10271	5686	19	2	0	Low-quality	16.7
Sample_T4A_k141_75159	11776	15	5	0	Low-quality	16.7
Sample_C2A_k141_36585	8570	9	0	2	Low-quality	16.69
Sample_C7B_k141_93291	28828	33	1	18	Low-quality	16.69
Sample_C9A_k141_106366	11481	14	2	2	Low-quality	16.69
Sample_T8C_k141_100517	15021	13	0	1	Low-quality	16.69
Sample_C3A_k141_52154	8677	14	0	0	Low-quality	16.69
Sample_T10A_k141_116234	6918	14	7	0	Low-quality	16.69
Sample_T12B_k141_149857	10053	21	5	1	Low-quality	16.69
Sample_C11B_k141_26972	7838	14	2	0	Low-quality	16.68
Sample_T12A_k141_140120	6802	8	0	1	Low-quality	16.68
Sample_T8C_k141_34260	8268	12	9	0	Low-quality	16.68
Sample_C13B_k141_5908	5502	4	0	0	Low-quality	16.67
Sample_C5A_k141_158866	7562	5	2	0	Low-quality	16.67
Sample_T16A_k141_99132	9408	14	1	1	Low-quality	16.67
Sample_C5A_k141_225722	6731	11	4	0	Low-quality	16.66
Sample_T12A_k141_148303	6865	14	1	0	Low-quality	16.66
Sample_T18A_k141_196597	7746	13	6	0	Low-quality	16.66
Sample_C2B_k141_258176	6354	9	2	0	Low-quality	16.65
Sample_T17B_k141_18732	6534	9	7	0	Low-quality	16.65
Sample_C2A_k141_78255	14108	15	1	7	Low-quality	16.64
Sample_T1A_k141_13705	7720	15	11	0	Low-quality	16.64
Sample_T5A_k141_5385	7270	12	5	0	Low-quality	16.64
Sample_T14A_k141_31509	13691	13	0	1	Low-quality	16.63
Sample_T7C_k141_103207	7349	5	0	1	Low-quality	16.63

Sample_T13A_k141_98514	6249	11	0	0	Low-quality	16.63
Sample_C2C_k141_64883	6815	4	0	1	Low-quality	16.62
Sample_C3A_k141_227699	9859	12	0	4	Low-quality	16.62
Sample_T13A_k141_35010	10975	17	1	3	Low-quality	16.61
Sample_T7C_k141_147584	13233	27	2	0	Low-quality	16.61
Sample_C2C_k141_140964	6808	11	2	0	Low-quality	16.6
Sample_T2A_k141_29006	6721	8	2	0	Low-quality	16.6
Sample_T3B_k141_34064	27385	38	17	0	Low-quality	16.6
Sample_T8C_k141_10965	5627	15	0	0	Low-quality	16.6
Sample_C6B_k141_40491	9935	8	0	1	Low-quality	16.59
Sample_T8A_k141_245363	5960	4	0	4	Low-quality	16.59
Sample_C5C_k141_63562	7237	8	6	0	Low-quality	16.59
Sample_T18B_k141_21957	9853	14	6	0	Low-quality	16.59
Sample_C3C_k141_256176	14351	12	1	2	Low-quality	16.58
Sample_T11C_k141_91353	8388	11	0	1	Low-quality	16.58
Sample_C10A_k141_330508	6212	12	0	1	Low-quality	16.58
Sample_C13B_k141_17366_fragment_1	7757	15	1	0	Low-quality	16.58
Sample_C8B_k141_155031	6351	9	7	0	Low-quality	16.58
Sample_T15B_k141_61926_fragment_1	18791	19	2	3	Low-quality	16.58
Sample_C11C_k141_37202	33230	74	10	1	Low-quality	16.57
Sample_C10A_k141_233233	6083	8	4	0	Low-quality	16.57
Sample_C9C_k141_68049	5844	11	9	0	Low-quality	16.57
Sample_T14A_k141_184251	24602	46	5	2	Low-quality	16.57
Sample_T18A_k141_103607	14561	14	6	0	Low-quality	16.57
Sample_C14A_k141_81290	24397	41	3	2	Low-quality	16.56
Sample_C14A_k141_145238	6033	13	5	0	Low-quality	16.56
Sample_C4B_k141_64489	6553	12	2	1	Low-quality	16.56
Sample_T14B_k141_15911	6412	10	7	0	Low-quality	16.56
Sample_C5A_k141_216443	8730	10	1	1	Low-quality	16.55
Sample_C11B_k141_14247	9624	11	1	5	Low-quality	16.52
Sample_T12B_k141_24838	11006	19	0	3	Low-quality	16.52
Sample_T3C_k141_115172	8245	10	1	6	Low-quality	16.52
Sample_C8A_k141_1197	9498	14	9	0	Low-quality	16.52
Sample_T8A_k141_87606	15408	12	0	0	Low-quality	16.51
Sample_C14B_k141_34360	6281	13	4	0	Low-quality	16.51
Sample_C4B_k141_51669	23236	34	4	0	Low-quality	16.51
Sample_C14A_k141_27476	5605	5	5	0	Low-quality	16.5
Sample_T11B_k141_50942	26052	32	1	3	Low-quality	16.5
Sample_T13B_k141_143310	7389	11	7	0	Low-quality	16.5

Sample_T12A_k141_56953	6797	4	2	0	Low-quality	16.49
Sample_C11B_k141_11972	7125	19	4	0	Low-quality	16.48
Sample_C14A_k141_38367	6533	7	1	0	Low-quality	16.48
Sample_T13B_k141_176427	6189	11	1	0	Low-quality	16.48
Sample_T17B_k141_25850	7207	12	5	0	Low-quality	16.48
Sample_C11A_k141_119483	9669	21	2	3	Low-quality	16.47
Sample_C13A_k141_91243_fragment_1	11010	14	1	0	Low-quality	16.47
Sample_C14B_k141_82445	6906	8	4	0	Low-quality	16.47
Sample_C2A_k141_132817	6164	8	0	0	Low-quality	16.47
Sample_T11A_k141_97427	10200	7	2	0	Low-quality	16.47
Sample_T13A_k141_47800	7362	8	3	0	Low-quality	16.47
Sample_T12C_k141_120514	10369	1	1	0	Low-quality	16.46
Sample_T15C_k141_124546	14814	12	0	1	Low-quality	16.46
Sample_T10B_k141_71711	21783	49	3	0	Low-quality	16.46
Sample_T12C_k141_68792	24833	35	4	0	Low-quality	16.46
Sample_T16B_k141_75129	8726	16	13	0	Low-quality	16.46
Sample_C13B_k141_13872	8313	7	0	1	Low-quality	16.45
Sample_T12A_k141_3003_fragment_1	6183	14	1	0	Low-quality	16.45
Sample_T8C_k141_113929	5801	9	7	0	Low-quality	16.45
Sample_T16A_k141_26388	13343	19	1	2	Low-quality	16.44
Sample_C12A_k141_56966	6270	14	12	0	Low-quality	16.44
Sample_C13A_k141_110265	5876	8	1	0	Low-quality	16.44
Sample_C3A_k141_191943	8626	14	6	0	Low-quality	16.44
Sample_T10A_k141_169953	6373	7	7	0	Low-quality	16.44
Sample_C11C_k141_103975_fragment_1	6529	13	1	0	Low-quality	16.43
Sample_C5A_k141_249824	6592	10	1	0	Low-quality	16.43
Sample_T11A_k141_55543	13275	19	1	2	Low-quality	16.43
Sample_T15C_k141_103898	9475	17	4	0	Low-quality	16.43
Sample_C2A_k141_204374	13418	12	0	3	Low-quality	16.42
Sample_C5A_k141_267483	17911	15	5	3	Low-quality	16.42
Sample_C6B_k141_56575_fragment_5	8341	9	0	1	Low-quality	16.42
Sample_C13A_k141_73892	6200	3	0	0	Low-quality	16.41
Sample_C5A_k141_144326	7529	8	0	3	Low-quality	16.41
Sample_T10A_k141_123434	6073	4	0	1	Low-quality	16.41
Sample_T17B_k141_8563	9823	14	0	0	Low-quality	16.41
Sample_C11A_k141_6135	9188	16	3	0	Low-quality	16.41
Sample_C7A_k141_151597	8532	10	3	1	Low-quality	16.41
Sample_T18B_k141_122173	8177	5	0	3	Low-quality	16.4
Sample_T6C_k141_193359	7196	13	3	0	Low-quality	16.4

Sample_C4A_k141_92980	17414	24	3	1	Low-quality	16.39
Sample_C10B_k141_122599	6063	15	4	0	Low-quality	16.39
Sample_C10C_k141_208821_fragment_1	9308	12	1	1	Low-quality	16.39
Sample_C2C_k141_116602	17679	14	0	4	Low-quality	16.38
Sample_C5A_k141_195524	13294	18	1	1	Low-quality	16.38
Sample_T7A_k141_18054	5861	10	1	0	Low-quality	16.38
Sample_C12A_k141_17790	5184	8	1	0	Low-quality	16.38
Sample_C6A_k141_49942_fragment_4	11239	15	1	3	Low-quality	16.38
Sample_T16C_k141_30205	6648	12	4	1	Low-quality	16.38
Sample_C2C_k141_196939	25169	21	0	9	Low-quality	16.37
Sample_C10C_k141_212554	9552	20	8	0	Low-quality	16.37
Sample_C2B_k141_319578	6529	9	5	0	Low-quality	16.37
Sample_C12C_k141_191523	8088	8	0	0	Low-quality	16.36
Sample_C4A_k141_17020	23878	18	3	0	Low-quality	16.36
Sample_C4C_k141_226214	11240	17	1	0	Low-quality	16.36
Sample_T6C_k141_166236_fragment_1	7691	11	2	1	Low-quality	16.36
Sample_C10C_k141_163401	9786	14	0	5	Low-quality	16.35
Sample_C2B_k141_266641	30148	32	1	13	Low-quality	16.35
Sample_T6C_k141_128691	25512	23	1	13	Low-quality	16.35
Sample_T12B_k141_56579	6505	8	1	0	Low-quality	16.35
Sample_T2A_k141_102356	10807	14	1	0	Low-quality	16.35
Sample_C8C_k141_90659	15512	15	1	6	Low-quality	16.34
Sample_C9B_k141_1765	13073	11	0	5	Low-quality	16.34
Sample_T2C_k141_257876	16651	13	0	9	Low-quality	16.34
Sample_C10C_k141_229824_fragment_1	7378	10	9	0	Low-quality	16.34
Sample_C1B_k141_248498	10745	19	0	1	Low-quality	16.34
Sample_C2B_k141_244083	6039	15	6	0	Low-quality	16.34
Sample_T14A_k141_226585	10268	10	4	0	Low-quality	16.34
Sample_C10C_k141_125667	5968	12	0	1	Low-quality	16.33
Sample_C9B_k141_71906	8316	13	7	4	Low-quality	16.33
Sample_C12C_k141_194300	7688	11	8	0	Low-quality	16.33
Sample_C14A_k141_149550	7541	9	3	1	Low-quality	16.33
Sample_T13C_k141_59021	9014	14	0	2	Low-quality	16.32
Sample_C4B_k141_26717	6419	13	1	0	Low-quality	16.32
Sample_C8A_k141_40697_fragment_1	8931	18	13	0	Low-quality	16.32
Sample_T10A_k141_159915	6631	14	2	1	Low-quality	16.32
Sample_T4B_k141_108898	17773	33	0	1	Low-quality	16.32
Sample_C10C_k141_30566	25339	28	1	18	Low-quality	16.31
Sample_T16C_k141_44967	6853	6	0	5	Low-quality	16.31

Sample_C14C_k141_134749	6284	10	1	0	Low-quality	16.31
Sample_C4B_k141_247211	9683	18	3	1	Low-quality	16.31
Sample_T13B_k141_25369	5953	6	2	0	Low-quality	16.31
Sample_T2B_k141_18786	8945	12	5	0	Low-quality	16.31
Sample_C7A_k141_87654	22737	31	2	0	Low-quality	16.3
Sample_T16B_k141_69078	5382	5	0	1	Low-quality	16.3
Sample_C12B_k141_136872	8859	12	0	0	Low-quality	16.3
Sample_C12C_k141_80043	6859	11	4	0	Low-quality	16.3
Sample_T2B_k141_2841	22699	19	3	0	Low-quality	16.3
Sample_T8B_k141_280043	5722	7	0	0	Low-quality	16.3
Sample_T10A_k141_151650	8162	12	1	0	Low-quality	16.29
Sample_T1C_k141_41187	6846	7	0	5	Low-quality	16.29
Sample_C11C_k141_56981	7529	13	3	0	Low-quality	16.29
Sample_C7A_k141_64610	21717	27	6	1	Low-quality	16.29
Sample_T16B_k141_165054	7590	17	5	0	Low-quality	16.29
Sample_C7A_k141_45557	9077	5	0	1	Low-quality	16.28
Sample_C2C_k141_302200	10753	16	1	1	Low-quality	16.27
Sample_C4B_k141_53448	9740	16	0	7	Low-quality	16.27
Sample_T10A_k141_65560	7655	9	1	0	Low-quality	16.27
Sample_C14A_k141_105462	6648	17	3	0	Low-quality	16.27
Sample_C1B_k141_29721	15054	24	2	0	Low-quality	16.27
Sample_T16A_k141_59913	5781	7	5	0	Low-quality	16.27
Sample_C3A_k141_152011	7369	10	2	0	Low-quality	16.25
Sample_C3A_k141_243496_fragment_1	7656	13	0	0	Low-quality	16.25
Sample_T13B_k141_140401	7541	7	3	0	Low-quality	16.25
Sample_T5C_k141_73538	14049	21	1	8	Low-quality	16.24
Sample_C8B_k141_36091	6837	6	0	0	Low-quality	16.24
Sample_C9C_k141_6119	5989	9	2	0	Low-quality	16.24
Sample_T10C_k141_74119_fragment_2	14057	9	1	0	Low-quality	16.24
Sample_T12A_k141_176828	15273	20	6	1	Low-quality	16.24
Sample_T13A_k141_97868	6317	22	2	0	Low-quality	16.24
Sample_T13B_k141_34680	6103	14	3	1	Low-quality	16.24
Sample_T2C_k141_146316	25739	48	7	1	Low-quality	16.24
Sample_T16C_k141_78461	7337	11	2	0	Low-quality	16.23
Sample_T16B_k141_43977	15799	15	0	4	Low-quality	16.22
Sample_C14C_k141_130692	7582	11	7	0	Low-quality	16.22
Sample_T8C_k141_142821	8476	10	5	0	Low-quality	16.22
Sample_C12A_k141_208085	9706	13	0	4	Low-quality	16.21
Sample_T10A_k141_25335	7528	6	0	4	Low-quality	16.21

Sample_T2C_k141_138635	14397	9	0	2	Low-quality	16.21
Sample_T8C_k141_56828	12589	17	2	2	Low-quality	16.21
Sample_C8A_k141_180127	6590	8	3	0	Low-quality	16.21
Sample_T15A_k141_66721_fragment_3	10712	12	1	0	Low-quality	16.21
Sample_C1B_k141_144553	40197	51	1	29	Low-quality	16.2
Sample_C2B_k141_17854	9700	8	0	1	Low-quality	16.2
Sample_T2A_k141_206198	6096	15	4	0	Low-quality	16.2
Sample_T2C_k141_62173	9849	9	6	0	Low-quality	16.2
Sample_C13C_k141_81958	7941	11	2	1	Low-quality	16.19
Sample_C9A_k141_34724	7072	18	7	0	Low-quality	16.19
Sample_T2B_k141_251806	13136	12	1	2	Low-quality	16.19
Sample_T12A_k141_331466	7746	13	8	0	Low-quality	16.19
Sample_C9A_k141_67686	9688	13	0	5	Low-quality	16.18
Sample_C7B_k141_129819	10120	8	4	0	Low-quality	16.18
Sample_T10B_k141_136899	13216	14	0	4	Low-quality	16.17
Sample_T15C_k141_44107	7008	13	1	0	Low-quality	16.17
Sample_T6A_k141_162353	14214	7	0	3	Low-quality	16.17
Sample_C12A_k141_5434	6698	12	5	0	Low-quality	16.17
Sample_T8C_k141_137774	9671	16	1	0	Low-quality	16.17
Sample_T13C_k141_29134	8068	16	1	7	Low-quality	16.16
Sample_C8B_k141_4736	6381	16	4	0	Low-quality	16.16
Sample_T11C_k141_16263	10682	13	1	0	Low-quality	16.16
Sample_T18B_k141_90801	8668	14	3	0	Low-quality	16.16
Sample_T1B_k141_2900	13303	20	1	1	Low-quality	16.16
Sample_C2A_k141_66104	14064	24	0	11	Low-quality	16.15
Sample_C10A_k141_20080_fragment_1	7221	10	6	0	Low-quality	16.15
Sample_C3A_k141_228725	7347	10	10	0	Low-quality	16.15
Sample_T10B_k141_62461	9505	25	1	0	Low-quality	16.15
Sample_T15C_k141_523	6047	3	0	1	Low-quality	16.14
Sample_T12A_k141_117442	6530	14	3	0	Low-quality	16.14
Sample_T13B_k141_25208_fragment_1	26262	46	1	0	Low-quality	16.14
Sample_C3C_k141_181228	10455	11	0	3	Low-quality	16.13
Sample_T6B_k141_127604	8989	10	0	3	Low-quality	16.12
Sample_T11C_k141_25127	5847	7	2	0	Low-quality	16.11
Sample_T16C_k141_34385	9721	21	0	1	Low-quality	16.11
Sample_T6C_k141_132139	14703	18	2	3	Low-quality	16.11
Sample_C1C_k141_142374	18436	23	2	3	Low-quality	16.11
Sample_C6C_k141_129426_fragment_1	22796	25	2	3	Low-quality	16.11
Sample_C9A_k141_32816	6746	11	7	0	Low-quality	16.11

Sample_C8B_k141_48201	10637	15	1	4	Low-quality	16.1
Sample_T14A_k141_17393	18489	18	0	4	Low-quality	16.1
Sample_C5A_k141_119239	8406	8	0	2	Low-quality	16.09
Sample_C12A_k141_195850	6380	7	1	0	Low-quality	16.09
Sample_C13A_k141_10261_fragment_1	12370	18	5	0	Low-quality	16.09
Sample_C10C_k141_211203	8195	12	1	2	Low-quality	16.08
Sample_C2C_k141_56494	10697	14	12	0	Low-quality	16.08
Sample_C13A_k141_15197	9623	13	1	0	Low-quality	16.07
Sample_C9C_k141_78554	12767	10	0	3	Low-quality	16.07
Sample_T4B_k141_197573	7801	7	0	3	Low-quality	16.07
Sample_C2A_k141_327237	8261	7	0	2	Low-quality	16.06
Sample_T6B_k141_20135	7685	8	0	0	Low-quality	16.06
Sample_T8C_k141_119254	19317	26	0	7	Low-quality	16.06
Sample_C4B_k141_192741	6242	8	4	0	Low-quality	16.06
Sample_C6B_k141_145311	54491	40	1	19	Low-quality	16.05
Sample_T6A_k141_173429	8935	10	0	7	Low-quality	16.05
Sample_T8A_k141_159003	7685	16	1	1	Low-quality	16.05
Sample_C3A_k141_97458	8447	7	1	0	Low-quality	16.05
Sample_C5C_k141_34111	6710	15	0	0	Low-quality	16.05
Sample_C9C_k141_69491	5659	6	2	0	Low-quality	16.05
Sample_T2A_k141_30990	34787	62	2	2	Low-quality	16.04
Sample_T3B_k141_22369	5907	6	5	0	Low-quality	16.04
Sample_C6A_k141_71918	20567	36	3	1	Low-quality	16.03
Sample_T13A_k141_111853	21336	28	5	1	Low-quality	16.03
Sample_T6B_k141_16201	8210	10	1	0	Low-quality	16.03
Sample_T17B_k141_30927	13888	13	1	9	Low-quality	16.02
Sample_C13A_k141_26536	5917	9	5	0	Low-quality	16.02
Sample_C4C_k141_46548_fragment_1	23149	14	3	0	Low-quality	16.02
Sample_C9C_k141_58569	5649	11	4	0	Low-quality	16.02
Sample_T18A_k141_124119	9203	19	1	0	Low-quality	16.02
Sample_C6B_k141_36892	7437	5	0	2	Low-quality	16.01
Sample_T14A_k141_151235	13083	13	0	6	Low-quality	16.01
Sample_C13A_k141_17592	5545	12	0	2	Low-quality	16.01
Sample_T8B_k141_2126	13182	19	6	2	Low-quality	16.01
Sample_C1B_k141_105566	5957	10	6	0	Low-quality	16
Sample_T12B_k141_108472	17972	37	2	0	Low-quality	16
Sample_C2B_k141_55014	6650	12	2	0	Low-quality	15.99
Sample_C9B_k141_47930	7345	14	3	0	Low-quality	15.99
Sample_T2A_k141_213042	6078	14	2	0	Low-quality	15.99

Sample_T10A_k141_152403	7257	10	5	0	Low-quality	15.98
Sample_T5C_k141_123890	7465	12	10	1	Low-quality	15.98
Sample_T8B_k141_124716	7199	10	3	0	Low-quality	15.98
Sample_T5B_k141_26083	8721	10	0	0	Low-quality	15.97
Sample_C10A_k141_140709	6393	20	3	0	Low-quality	15.97
Sample_C7B_k141_52813	12899	12	5	0	Low-quality	15.97
Sample_C5A_k141_44513	28148	62	3	0	Low-quality	15.96
Sample_T18A_k141_12717	6951	12	9	0	Low-quality	15.96
Sample_T14B_k141_181954	9910	15	0	1	Low-quality	15.95
Sample_C9C_k141_20464	5958	11	1	1	Low-quality	15.93
Sample_C10A_k141_310588	9530	11	0	4	Low-quality	15.92
Sample_T4B_k141_26271	13412	17	0	2	Low-quality	15.92
Sample_C3A_k141_224789_fragment_1	9131	12	5	0	Low-quality	15.92
Sample_C5C_k141_13973	7286	16	13	0	Low-quality	15.92
Sample_C7A_k141_28378	7517	12	7	0	Low-quality	15.92
Sample_T7B_k141_67145	13111	14	2	1	Low-quality	15.92
Sample_C11B_k141_79385	30788	27	0	15	Low-quality	15.91
Sample_C2A_k141_71088	8016	12	0	5	Low-quality	15.91
Sample_C4B_k141_31238	28806	67	1	2	Low-quality	15.91
Sample_T2C_k141_176887	6671	6	0	4	Low-quality	15.91
Sample_C3A_k141_134810_fragment_1	8015	9	0	1	Low-quality	15.91
Sample_T14B_k141_145600	6627	6	0	2	Low-quality	15.9
Sample_C6B_k141_74446	6622	7	2	0	Low-quality	15.9
Sample_T17B_k141_29999	15056	18	0	3	Low-quality	15.89
Sample_T6B_k141_183408	12987	15	0	9	Low-quality	15.89
Sample_C2A_k141_86065	8979	17	6	0	Low-quality	15.89
Sample_C2C_k141_52194	6602	5	2	0	Low-quality	15.89
Sample_C4B_k141_186895	14181	9	1	4	Low-quality	15.88
Sample_C14A_k141_78749	16383	31	5	0	Low-quality	15.88
Sample_T2B_k141_293600	6774	4	0	1	Low-quality	15.87
Sample_T2C_k141_3837	7981	11	0	2	Low-quality	15.87
Sample_C2B_k141_328861	7845	19	5	0	Low-quality	15.87
Sample_C8A_k141_69653	11200	19	2	0	Low-quality	15.87
Sample_T10B_k141_67975	13753	19	1	0	Low-quality	15.87
Sample_T4B_k141_166188	6547	6	4	0	Low-quality	15.87
Sample_T5A_k141_25117_fragment_1	12799	20	5	0	Low-quality	15.87
Sample_T12C_k141_97465_fragment_1	18356	14	5	1	Low-quality	15.85
Sample_T17B_k141_572	11552	15	1	3	Low-quality	15.85
Sample_T2A_k141_221984	6496	7	0	0	Low-quality	15.85

Sample_T1C_k141_33910	7364	6	0	1	Low-quality	15.84
Sample_C8B_k141_141468_fragment_1	7152	13	2	0	Low-quality	15.84
Sample_C11A_k141_31710_fragment_1	16830	17	6	2	Low-quality	15.83
Sample_C3A_k141_112103	7121	11	1	1	Low-quality	15.83
Sample_C8C_k141_144590	7071	13	9	0	Low-quality	15.83
Sample_T4B_k141_325167	6934	10	5	0	Low-quality	15.83
Sample_T11C_k141_160780	14955	13	2	2	Low-quality	15.82
Sample_T4B_k141_252993	12782	14	1	2	Low-quality	15.82
Sample_C13B_k141_115500	6083	4	4	0	Low-quality	15.82
Sample_C3B_k141_49149	9059	21	3	1	Low-quality	15.82
Sample_C4C_k141_39028	9787	19	3	0	Low-quality	15.82
Sample_T8B_k141_11836	6627	13	4	0	Low-quality	15.82
Sample_C4A_k141_239055	39233	36	1	27	Low-quality	15.81
Sample_C5A_k141_121549	9762	11	1	1	Low-quality	15.81
Sample_C12A_k141_63017_fragment_1	5196	11	1	0	Low-quality	15.81
Sample_C12C_k141_70177	5723	11	4	0	Low-quality	15.81
Sample_T2C_k141_145922	6833	13	0	0	Low-quality	15.81
Sample_C4C_k141_72803	9495	13	3	0	Low-quality	15.8
Sample_C3C_k141_262843	8418	12	3	1	Low-quality	15.8
Sample_T12A_k141_289	12560	15	0	6	Low-quality	15.79
Sample_C12C_k141_203210	6634	13	0	0	Low-quality	15.79
Sample_C2A_k141_136967	6386	13	4	0	Low-quality	15.79
Sample_C2B_k141_208969	6451	15	4	0	Low-quality	15.79
Sample_C4C_k141_231642	8455	8	0	0	Low-quality	15.78
Sample_C13B_k141_112080	5158	5	1	0	Low-quality	15.78
Sample_T5B_k141_35780	5962	15	1	0	Low-quality	15.78
Sample_T8C_k141_92303_fragment_1	8268	12	2	0	Low-quality	15.78
Sample_T3A_k141_83507	9719	2	1	0	Low-quality	15.77
Sample_C12B_k141_53391	9317	10	1	0	Low-quality	15.77
Sample_C3B_k141_247065	11014	15	9	0	Low-quality	15.77
Sample_T6C_k141_63555	7989	8	0	1	Low-quality	15.76
Sample_C4C_k141_179425	9724	25	3	0	Low-quality	15.76
Sample_C8B_k141_26043_fragment_1	12118	18	5	0	Low-quality	15.76
Sample_T12C_k141_174053	12048	26	5	0	Low-quality	15.76
Sample_C13C_k141_106716_fragment_1	12109	19	5	0	Low-quality	15.75
Sample_C8A_k141_39318	5609	9	6	0	Low-quality	15.75
Sample_T6B_k141_150084	5478	11	9	0	Low-quality	15.75
Sample_C14A_k141_148262	15677	14	0	12	Low-quality	15.74
Sample_T1B_k141_29738	10402	7	1	0	Low-quality	15.74

Sample_C10A_k141_231577	6232	13	2	0	Low-quality	15.74
Sample_T13B_k141_176519	9476	12	1	0	Low-quality	15.74
Sample_T7A_k141_101719	6105	8	6	0	Low-quality	15.74
Sample_C3C_k141_87416	6026	3	0	1	Low-quality	15.73
Sample_T2B_k141_202672	86131	79	1	49	Low-quality	15.73
Sample_T2C_k141_145610	6278	10	6	0	Low-quality	15.73
Sample_C12A_k141_78021	6520	6	0	0	Low-quality	15.72
Sample_C6B_k141_27293	13768	12	1	6	Low-quality	15.72
Sample_C3A_k141_244175	7798	13	7	0	Low-quality	15.72
Sample_C2C_k141_241126	12555	11	0	8	Low-quality	15.71
Sample_T14C_k141_75373	7759	8	0	1	Low-quality	15.71
Sample_C11C_k141_1737	7168	10	1	0	Low-quality	15.71
Sample_T8A_k141_197847	5872	9	3	0	Low-quality	15.71
Sample_C12B_k141_64794	10374	13	1	1	Low-quality	15.7
Sample_C14C_k141_115200	18716	16	0	11	Low-quality	15.69
Sample_C1B_k141_268958	6593	6	0	5	Low-quality	15.69
Sample_C6B_k141_204263	16974	18	0	5	Low-quality	15.69
Sample_T14A_k141_45613	8176	9	0	3	Low-quality	15.69
Sample_C11B_k141_248356	5303	7	4	1	Low-quality	15.69
Sample_C14A_k141_188902	6036	7	3	0	Low-quality	15.69
Sample_C1B_k141_237107	26571	18	5	0	Low-quality	15.69
Sample_C8B_k141_98610	8104	7	0	0	Low-quality	15.69
Sample_T15A_k141_13406	6337	11	5	0	Low-quality	15.69
Sample_T8B_k141_254615	22975	13	2	0	Low-quality	15.69
Sample_T10A_k141_54003	6401	6	5	0	Low-quality	15.68
Sample_T10B_k141_171402_fragment_3	19724	38	1	0	Low-quality	15.68
Sample_T5B_k141_8595	15461	15	0	3	Low-quality	15.67
Sample_C3B_k141_221887	5556	8	3	0	Low-quality	15.67
Sample_C4C_k141_29219_fragment_1	8391	10	1	1	Low-quality	15.67
Sample_C7C_k141_12109	7009	12	5	0	Low-quality	15.67
Sample_T16A_k141_121136	8386	15	4	0	Low-quality	15.67
Sample_C12A_k141_26346	16450	15	0	3	Low-quality	15.66
Sample_C3C_k141_41715	5903	9	1	0	Low-quality	15.66
Sample_T13B_k141_135489	9375	9	0	4	Low-quality	15.66
Sample_C13A_k141_105997	9311	8	1	0	Low-quality	15.66
Sample_T16B_k141_6100	7631	6	1	0	Low-quality	15.64
Sample_T17C_k141_10361	16178	13	0	6	Low-quality	15.64
Sample_C3A_k141_144222	6252	5	2	0	Low-quality	15.64
Sample_C6C_k141_143501	6359	10	7	0	Low-quality	15.64

Sample_T2B_k141_47776	5655	9	3	0	Low-quality	15.64
Sample_C3B_k141_231204	85592	73	1	56	Low-quality	15.63
Sample_T18A_k141_61984	12121	17	0	1	Low-quality	15.63
Sample_T4C_k141_125196	16142	21	0	2	Low-quality	15.63
Sample_T5C_k141_157440	5132	12	1	0	Low-quality	15.62
Sample_C10A_k141_304233	6086	6	3	0	Low-quality	15.62
Sample_C2B_k141_224171	5478	8	7	0	Low-quality	15.62
Sample_T17C_k141_38590	5089	9	6	0	Low-quality	15.62
Sample_C8C_k141_18350	6959	4	0	1	Low-quality	15.61
Sample_T11C_k141_62358	23228	21	1	2	Low-quality	15.61
Sample_T15C_k141_127622	8137	10	0	1	Low-quality	15.61
Sample_C12A_k141_93126	5941	14	3	0	Low-quality	15.61
Sample_C8A_k141_102239	6071	15	1	1	Low-quality	15.61
Sample_C6B_k141_111235	9007	7	0	1	Low-quality	15.6
Sample_T14A_k141_231441	27009	22	0	0	Low-quality	15.6
Sample_C3B_k141_70088	15124	12	0	3	Low-quality	15.57
Sample_T1C_k141_38145	5377	12	0	1	Low-quality	15.57
Sample_T4B_k141_199453	24297	26	1	16	Low-quality	15.57
Sample_T14A_k141_10706	23866	17	3	1	Low-quality	15.57
Sample_C13A_k141_72318	8615	14	3	0	Low-quality	15.56
Sample_C8A_k141_168147	9718	16	2	1	Low-quality	15.56
Sample_C11B_k141_107231	7239	10	5	0	Low-quality	15.55
Sample_T15C_k141_84566	7251	6	1	0	Low-quality	15.55
Sample_T6B_k141_64632	6478	16	3	0	Low-quality	15.55
Sample_T7B_k141_25521	11089	11	2	1	Low-quality	15.55
Sample_T10A_k141_182660	6159	14	0	1	Low-quality	15.54
Sample_T15C_k141_42852	6618	9	0	0	Low-quality	15.54
Sample_T8B_k141_173565	6546	4	0	1	Low-quality	15.54
Sample_C11C_k141_181521	9354	9	1	0	Low-quality	15.54
Sample_C7B_k141_48220_fragment_1	10916	7	1	2	Low-quality	15.54
Sample_T10C_k141_21313	9428	17	1	0	Low-quality	15.54
Sample_C6B_k141_47527	11301	16	2	4	Low-quality	15.53
Sample_T17B_k141_981	7458	8	0	0	Low-quality	15.53
Sample_T1B_k141_42986	7573	8	0	6	Low-quality	15.52
Sample_T8B_k141_228722	12539	18	1	5	Low-quality	15.52
Sample_T12A_k141_336984	12830	20	1	1	Low-quality	15.51
Sample_T5B_k141_25847	8835	6	0	2	Low-quality	15.51
Sample_C13C_k141_15947	9643	13	1	4	Low-quality	15.51
Sample_T2C_k141_38494	6950	16	1	0	Low-quality	15.51

Sample_T3C_k141_60020	8985	18	0	1	Low-quality	15.5
Sample_T6A_k141_135669	8072	8	0	1	Low-quality	15.5
Sample_C6B_k141_115141	6971	13	10	0	Low-quality	15.5
Sample_T3C_k141_13139	6775	7	4	0	Low-quality	15.5
Sample_C4B_k141_251661	7742	8	0	6	Low-quality	15.49
Sample_C9A_k141_43118	6285	12	4	0	Low-quality	15.49
Sample_T11C_k141_139768_fragment_1	6970	9	3	0	Low-quality	15.49
Sample_T8A_k141_63107_fragment_2	7617	10	1	0	Low-quality	15.49
Sample_C1A_k141_59159	6137	5	0	3	Low-quality	15.48
Sample_C11B_k141_212947	5242	9	4	0	Low-quality	15.48
Sample_C13C_k141_15556_fragment_1	16166	21	1	2	Low-quality	15.48
Sample_T3C_k141_104084	8614	11	0	6	Low-quality	15.47
Sample_C4C_k141_111037	7338	13	10	0	Low-quality	15.47
Sample_T5B_k141_69502	5829	11	4	0	Low-quality	15.47
Sample_T6C_k141_114070	5537	11	2	1	Low-quality	15.47
Sample_C5B_k141_45369_fragment_1	8574	12	4	0	Low-quality	15.46
Sample_T16B_k141_59190	31613	27	1	21	Low-quality	15.45
Sample_C4C_k141_218378	16672	13	0	8	Low-quality	15.44
Sample_C10C_k141_33834	5608	13	1	0	Low-quality	15.44
Sample_C14A_k141_226659	5272	14	1	0	Low-quality	15.44
Sample_T11A_k141_137627	6493	10	6	0	Low-quality	15.44
Sample_C12A_k141_6047	6081	7	3	0	Low-quality	15.43
Sample_C7B_k141_112122_fragment_1	31514	30	2	6	Low-quality	15.43
Sample_C13C_k141_82798	6232	12	2	0	Low-quality	15.42
Sample_C3C_k141_202546	8565	8	0	0	Low-quality	15.4
Sample_T18A_k141_182024	9437	17	1	1	Low-quality	15.4
Sample_T7A_k141_149248	13860	9	0	2	Low-quality	15.4
Sample_T7A_k141_173421	5943	10	6	0	Low-quality	15.4
Sample_C7B_k141_137082	8581	4	0	1	Low-quality	15.39
Sample_C11B_k141_69260	13354	24	0	0	Low-quality	15.39
Sample_C4C_k141_150692	6480	15	1	0	Low-quality	15.39
Sample_C4C_k141_134329	5178	8	1	1	Low-quality	15.39
Sample_C11A_k141_38577	24519	28	1	7	Low-quality	15.38
Sample_C1B_k141_46721	5530	5	4	0	Low-quality	15.38
Sample_C4A_k141_61097_fragment_1	8246	16	5	0	Low-quality	15.38
Sample_C6C_k141_85462	15613	17	0	0	Low-quality	15.38
Sample_C4C_k141_173520	6484	8	2	0	Low-quality	15.37
Sample_T14A_k141_199005	17560	18	1	2	Low-quality	15.37
Sample_T14A_k141_145429	8396	10	0	1	Low-quality	15.37

Sample_C13B_k141_62948	6119	4	3	0	Low-quality	15.37
Sample_T14B_k141_216644	7287	13	0	0	Low-quality	15.37
Sample_T14A_k141_173473	17392	18	0	1	Low-quality	15.36
Sample_T14A_k141_46864	12176	29	0	0	Low-quality	15.36
Sample_C12A_k141_148391	6438	15	0	0	Low-quality	15.36
Sample_C10C_k141_158844	6043	8	6	0	Low-quality	15.35
Sample_T6C_k141_149582	11534	20	5	3	Low-quality	15.35
Sample_T11C_k141_81239	10825	10	1	1	Low-quality	15.34
Sample_C12A_k141_102645	9091	13	1	0	Low-quality	15.34
Sample_C12C_k141_209914	7525	9	6	0	Low-quality	15.34
Sample_T10A_k141_43994	6960	5	5	0	Low-quality	15.34
Sample_T17B_k141_23128	6888	13	11	0	Low-quality	15.34
Sample_T3B_k141_169523_fragment_1	8147	12	1	0	Low-quality	15.34
Sample_T5C_k141_11332	6760	8	0	0	Low-quality	15.34
Sample_T10B_k141_142819	6580	9	3	0	Low-quality	15.33
Sample_T13A_k141_85783	6284	9	1	0	Low-quality	15.33
Sample_C4B_k141_30984	5506	4	0	2	Low-quality	15.32
Sample_C11B_k141_113991	9964	16	2	0	Low-quality	15.32
Sample_C3B_k141_261363	12460	24	5	2	Low-quality	15.32
Sample_T17B_k141_16905	5777	8	1	0	Low-quality	15.32
Sample_C7B_k141_37016	8431	8	0	0	Low-quality	15.31
Sample_T14B_k141_198620	5766	9	0	1	Low-quality	15.31
Sample_T4B_k141_151343	6678	7	1	0	Low-quality	15.31
Sample_T3A_k141_19539	5917	7	0	1	Low-quality	15.3
Sample_T2C_k141_220989	5860	5	2	0	Low-quality	15.3
Sample_T6A_k141_289478	5715	7	5	0	Low-quality	15.3
Sample_C5C_k141_30770	8650	7	0	1	Low-quality	15.29
Sample_T7B_k141_46292	11375	11	1	1	Low-quality	15.29
Sample_C11B_k141_175152	12489	11	0	0	Low-quality	15.28
Sample_C5B_k141_29574	6458	6	0	4	Low-quality	15.28
Sample_T4B_k141_310379	13219	15	1	6	Low-quality	15.28
Sample_T2B_k141_276195	6789	9	2	0	Low-quality	15.28
Sample_T4B_k141_203822_fragment_2	6209	13	1	0	Low-quality	15.28
Sample_C1A_k141_232230	6916	6	1	0	Low-quality	15.27
Sample_T5C_k141_53383	6412	7	0	2	Low-quality	15.26
Sample_T7A_k141_160440	8511	8	0	2	Low-quality	15.26
Sample_C10C_k141_206712	5988	9	7	0	Low-quality	15.26
Sample_C11C_k141_181618	7215	12	3	0	Low-quality	15.26
Sample_C4A_k141_129596	6995	11	0	0	Low-quality	15.26

Sample_T14B_k141_112034	5690	5	2	0	Low-quality	15.26
Sample_C3A_k141_97008	5761	13	1	0	Low-quality	15.25
Sample_T10A_k141_39456	12753	20	3	0	Low-quality	15.25
Sample_T15B_k141_130761	19128	30	3	2	Low-quality	15.25
Sample_T2C_k141_35257	5944	13	0	0	Low-quality	15.25
Sample_C13B_k141_55146	9165	8	0	7	Low-quality	15.24
Sample_T12B_k141_19934	5837	3	0	2	Low-quality	15.24
Sample_C11B_k141_107576	6861	10	3	0	Low-quality	15.24
Sample_C4B_k141_192872	7528	11	6	0	Low-quality	15.24
Sample_C8C_k141_30868	7228	9	2	0	Low-quality	15.24
Sample_C12A_k141_94180	15847	16	0	14	Low-quality	15.23
Sample_T3B_k141_118461	27725	28	0	8	Low-quality	15.23
Sample_T17A_k141_9916	10057	12	1	4	Low-quality	15.22
Sample_T18B_k141_72733	8112	11	0	4	Low-quality	15.22
Sample_T12A_k141_114818_fragment_1	8255	15	6	0	Low-quality	15.22
Sample_T14B_k141_46048	5489	8	6	0	Low-quality	15.22
Sample_C4C_k141_91170	7559	9	0	1	Low-quality	15.21
Sample_T3C_k141_118955	8831	15	1	1	Low-quality	15.21
Sample_T5A_k141_43740	9881	11	0	4	Low-quality	15.21
Sample_C1C_k141_96568	5372	13	5	0	Low-quality	15.21
Sample_C4B_k141_85126	26846	28	9	0	Low-quality	15.21
Sample_C6B_k141_55444	6103	12	6	0	Low-quality	15.21
Sample_T11A_k141_91692	5521	8	0	0	Low-quality	15.21
Sample_T2B_k141_84752	7909	10	0	0	Low-quality	15.21
Sample_C14B_k141_43758	6988	5	0	2	Low-quality	15.2
Sample_C12B_k141_59773	5397	16	3	0	Low-quality	15.2
Sample_C5B_k141_63112	7019	13	1	3	Low-quality	15.19
Sample_T12C_k141_20474	34858	38	0	17	Low-quality	15.19
Sample_T8B_k141_247644	10222	10	0	3	Low-quality	15.19
Sample_C11C_k141_171090	6823	7	3	0	Low-quality	15.19
Sample_C12A_k141_84618	6497	6	1	0	Low-quality	15.19
Sample_C1B_k141_280191	6401	14	0	0	Low-quality	15.19
Sample_T13C_k141_1007	8095	16	4	0	Low-quality	15.19
Sample_C1C_k141_81971	5946	6	0	5	Low-quality	15.18
Sample_C9A_k141_86594	18034	19	0	4	Low-quality	15.18
Sample_T13C_k141_70710	9789	10	0	5	Low-quality	15.18
Sample_T5C_k141_178039	19322	22	1	2	Low-quality	15.18
Sample_T17B_k141_27126	16950	17	1	2	Low-quality	15.18
Sample_C10B_k141_123720	6129	9	4	0	Low-quality	15.17

Sample_C2B_k141_23723	6709	10	1	0	Low-quality	15.17
Sample_T12A_k141_168391	5957	8	4	0	Low-quality	15.17
Sample_T12A_k141_177138	7260	6	0	0	Low-quality	15.17
Sample_C4B_k141_153722	5041	5	0	4	Low-quality	15.16
Sample_C7B_k141_954	7046	5	0	2	Low-quality	15.16
Sample_C2C_k141_22864	11232	7	0	1	Low-quality	15.16
Sample_T13C_k141_60933	15355	13	0	4	Low-quality	15.15
Sample_T4B_k141_87099	8829	6	0	4	Low-quality	15.15
Sample_T12A_k141_306861	7512	11	2	0	Low-quality	15.15
Sample_T1A_k141_17514	7019	14	9	0	Low-quality	15.15
Sample_T2B_k141_152962	23346	21	6	0	Low-quality	15.15
Sample_T6A_k141_121640	12376	13	1	2	Low-quality	15.14
Sample_T10A_k141_49333	5712	8	3	0	Low-quality	15.14
Sample_C3C_k141_255973	8544	13	0	2	Low-quality	15.13
Sample_C6B_k141_41110	12633	9	1	0	Low-quality	15.13
Sample_T8A_k141_7828	10680	11	0	5	Low-quality	15.13
Sample_C8C_k141_105740	34756	24	0	6	Low-quality	15.12
Sample_C14C_k141_121524_fragment_2	11755	10	0	2	Low-quality	15.12
Sample_C8B_k141_176678	5862	9	5	0	Low-quality	15.12
Sample_C14A_k141_165729	5819	9	4	0	Low-quality	15.11
Sample_C6B_k141_47723	31053	32	6	1	Low-quality	15.11
Sample_C6B_k141_24381	6966	13	10	0	Low-quality	15.11
Sample_T7A_k141_227445	6610	8	1	0	Low-quality	15.11
Sample_C7B_k141_172143	17825	12	6	0	Low-quality	15.1
Sample_C8C_k141_81541	5128	8	5	0	Low-quality	15.1
Sample_T1B_k141_7553_fragment_1	8862	13	1	4	Low-quality	15.1
Sample_T5B_k141_65716	7521	11	9	0	Low-quality	15.1
Sample_T3A_k141_10420	6391	14	2	1	Low-quality	15.09
Sample_C10C_k141_191045	7079	14	0	2	Low-quality	15.08
Sample_C4C_k141_195992	12239	12	1	2	Low-quality	15.08
Sample_T8A_k141_13344	8338	10	0	5	Low-quality	15.08
Sample_C5A_k141_13749	6331	6	3	0	Low-quality	15.08
Sample_T16A_k141_77433	6209	12	6	0	Low-quality	15.08
Sample_T17A_k141_36902	5156	8	5	0	Low-quality	15.08
Sample_T7A_k141_162320	5472	7	0	0	Low-quality	15.08
Sample_C14A_k141_29917	5953	11	1	0	Low-quality	15.07
Sample_C8C_k141_74996	9550	8	0	2	Low-quality	15.07
Sample_T10B_k141_161657	11356	17	2	2	Low-quality	15.07
Sample_T10A_k141_62178	9018	11	1	1	Low-quality	15.06

Sample_T3A_k141_33004	7532	7	0	1	Low-quality	15.06
Sample_C1B_k141_245563_fragment_1	9954	10	1	0	Low-quality	15.06
Sample_C4C_k141_153145	10384	19	5	0	Low-quality	15.06
Sample_C3A_k141_61914	6411	11	1	0	Low-quality	15.05
Sample_T4B_k141_309032	6916	10	4	0	Low-quality	15.05
Sample_T8C_k141_145410	6062	7	6	0	Low-quality	15.05
Sample_T5C_k141_165751	5590	6	0	0	Low-quality	15.04
Sample_T18B_k141_22638_fragment_1	9295	14	3	2	Low-quality	15.04
Sample_T5B_k141_67786_fragment_1	5615	8	2	0	Low-quality	15.04
Sample_C4B_k141_104153	25563	23	0	17	Low-quality	15.03
Sample_C6B_k141_178349	10241	11	0	5	Low-quality	15.03
Sample_T2C_k141_161732	24223	31	5	1	Low-quality	15.03
Sample_C9A_k141_63494	26453	68	3	0	Low-quality	15.02
Sample_T6C_k141_55848	8475	9	0	0	Low-quality	15.02
Sample_T6C_k141_159214	9927	14	1	3	Low-quality	15.02
Sample_C2B_k141_223927	9114	20	0	0	Low-quality	15.02
Sample_C2C_k141_101305	9386	12	10	0	Low-quality	15.02
Sample_C3C_k141_178390	24561	36	1	1	Low-quality	15.02
Sample_T7A_k141_89374	11348	24	2	1	Low-quality	15.02
Sample_T11A_k141_109526	36917	42	1	9	Low-quality	15.01
Sample_C10A_k141_243771	20701	26	2	0	Low-quality	15.01
Sample_C11B_k141_81996	7228	10	8	0	Low-quality	15.01
Sample_C10C_k141_230037	7915	9	1	0	Low-quality	15
Sample_T5C_k141_34285_fragment_3	27173	29	1	2	Low-quality	15
Sample_T7C_k141_33260	6112	15	3	0	Low-quality	15
Sample_C14A_k141_215720	8251	11	4	0	Low-quality	14.99
Sample_C3B_k141_121621	7182	13	4	1	Low-quality	14.99
Sample_C6A_k141_235135	7908	9	1	0	Low-quality	14.99
Sample_T4C_k141_102729_fragment_1	13788	10	0	0	Low-quality	14.99
Sample_C12A_k141_65422	5307	5	0	4	Low-quality	14.98
Sample_C1A_k141_105096	12242	16	0	5	Low-quality	14.98
Sample_C3B_k141_219662	10953	5	0	0	Low-quality	14.98
Sample_T16A_k141_5140	5792	6	0	0	Low-quality	14.98
Sample_C12C_k141_78861	7075	10	3	0	Low-quality	14.98
Sample_C4B_k141_4299	14793	22	5	3	Low-quality	14.98
Sample_C4B_k141_126537	8960	12	0	4	Low-quality	14.97
Sample_C8A_k141_11062	6679	7	0	1	Low-quality	14.97
Sample_T2A_k141_28529	72383	86	5	23	Low-quality	14.97
Sample_T8B_k141_100346	17774	20	0	8	Low-quality	14.97

Sample_C3A_k141_50071	6017	7	1	0	Low-quality	14.97
Sample_T11A_k141_27220	5764	7	3	0	Low-quality	14.97
Sample_C3C_k141_193988	5654	4	0	0	Low-quality	14.96
Sample_C5A_k141_235085	6248	8	2	0	Low-quality	14.96
Sample_C10A_k141_76415	7772	18	1	1	Low-quality	14.96
Sample_C13C_k141_54749	5004	13	2	0	Low-quality	14.96
Sample_C4A_k141_27917	8875	14	2	0	Low-quality	14.96
Sample_T4C_k141_60992_fragment_1	9460	21	1	0	Low-quality	14.96
Sample_T5B_k141_55685	13910	28	7	2	Low-quality	14.96
Sample_C11A_k141_155756	5783	7	0	1	Low-quality	14.95
Sample_C1C_k141_103074	9878	12	1	5	Low-quality	14.95
Sample_C5B_k141_44539	8952	10	1	0	Low-quality	14.95
Sample_C11C_k141_107334	5907	16	1	0	Low-quality	14.95
Sample_C12C_k141_124427	7290	7	0	1	Low-quality	14.95
Sample_C12C_k141_77237	25723	29	7	7	Low-quality	14.95
Sample_T5A_k141_48849_fragment_1	12486	15	1	0	Low-quality	14.95
Sample_C11B_k141_56828	7230	4	0	0	Low-quality	14.94
Sample_C11A_k141_107177	9693	20	1	0	Low-quality	14.94
Sample_T7B_k141_146367	8589	21	2	0	Low-quality	14.94
Sample_T7B_k141_25623	6706	6	3	0	Low-quality	14.94
Sample_C10A_k141_104201	9099	4	3	0	Low-quality	14.93
Sample_C4C_k141_111609	7231	11	0	0	Low-quality	14.93
Sample_C12C_k141_84439	82190	72	3	38	Low-quality	14.92
Sample_C13C_k141_49297	8266	9	0	0	Low-quality	14.92
Sample_T10A_k141_71864_fragment_1	14080	24	3	2	Low-quality	14.92
Sample_T2C_k141_212206	25528	38	3	1	Low-quality	14.92
Sample_C11C_k141_40156	7532	8	0	1	Low-quality	14.91
Sample_T11A_k141_56954	24173	28	1	1	Low-quality	14.91
Sample_T5B_k141_14706	5671	6	0	3	Low-quality	14.91
Sample_C8C_k141_128977	7827	14	3	0	Low-quality	14.91
Sample_T12B_k141_143800	5448	10	5	0	Low-quality	14.91
Sample_C6B_k141_128906	5380	7	3	0	Low-quality	14.9
Sample_C7C_k141_22716	6711	5	1	0	Low-quality	14.9
Sample_T13C_k141_121429	7645	13	10	0	Low-quality	14.9
Sample_T2B_k141_270585	7363	13	4	0	Low-quality	14.9
Sample_T7A_k141_101426	5901	8	8	0	Low-quality	14.9
Sample_C1A_k141_244194	36943	31	1	25	Low-quality	14.89
Sample_C1A_k141_91503	7491	10	0	3	Low-quality	14.89
Sample_T3C_k141_89671	12028	16	1	6	Low-quality	14.89

Sample_C1B_k141_181824	6963	10	4	0	Low-quality	14.89
Sample_C5C_k141_96003	5523	6	2	0	Low-quality	14.89
Sample_T4B_k141_139138	5551	11	4	0	Low-quality	14.89
Sample_T7B_k141_105733	36907	58	3	1	Low-quality	14.89
Sample_C13B_k141_77748	10815	15	0	1	Low-quality	14.88
Sample_T14C_k141_122784	11609	15	5	3	Low-quality	14.88
Sample_T4B_k141_126526	16244	24	3	0	Low-quality	14.88
Sample_T14B_k141_130361	7377	11	1	1	Low-quality	14.87
Sample_T5C_k141_127914	8629	16	1	4	Low-quality	14.87
Sample_C13C_k141_3757	5334	13	0	0	Low-quality	14.87
Sample_C4A_k141_174186	6813	8	7	0	Low-quality	14.87
Sample_T18B_k141_124700_fragment_1	10102	9	3	2	Low-quality	14.87
Sample_C2B_k141_115915	12147	10	0	2	Low-quality	14.86
Sample_C4C_k141_164152	6248	12	1	0	Low-quality	14.86
Sample_C2B_k141_264910	7209	9	3	0	Low-quality	14.86
Sample_C6A_k141_170308	7169	15	2	0	Low-quality	14.86
Sample_C13C_k141_93990	6240	6	0	4	Low-quality	14.85
Sample_C10C_k141_29561	5924	6	6	0	Low-quality	14.85
Sample_C2B_k141_291720	6072	5	4	0	Low-quality	14.85
Sample_T10A_k141_94465	6347	15	2	0	Low-quality	14.85
Sample_C10B_k141_103062	11356	5	0	2	Low-quality	14.84
Sample_C6A_k141_174816	25910	26	0	20	Low-quality	14.84
Sample_C3B_k141_94068	8542	9	2	0	Low-quality	14.84
Sample_C7A_k141_110616	19856	40	4	2	Low-quality	14.84
Sample_T7C_k141_137892	6633	9	1	0	Low-quality	14.84
Sample_C3A_k141_10342	16042	13	0	3	Low-quality	14.83
Sample_T13C_k141_10501	36516	25	1	3	Low-quality	14.83
Sample_T7C_k141_120121	9959	13	0	1	Low-quality	14.83
Sample_T13C_k141_41666	6583	11	0	0	Low-quality	14.83
Sample_C2A_k141_229512	7506	8	0	1	Low-quality	14.82
Sample_T17B_k141_33001	5782	12	7	0	Low-quality	14.82
Sample_T4B_k141_236385	13329	12	0	1	Low-quality	14.81
Sample_C14A_k141_147494	5779	17	3	0	Low-quality	14.81
Sample_C6B_k141_154531	5251	10	0	0	Low-quality	14.81
Sample_C8B_k141_185707	6123	21	2	0	Low-quality	14.81
Sample_T15A_k141_99876_fragment_1	9395	8	0	0	Low-quality	14.81
Sample_C12C_k141_62688	5056	8	4	0	Low-quality	14.8
Sample_C13C_k141_45312	14097	10	0	3	Low-quality	14.79
Sample_T11C_k141_34433	7544	7	0	1	Low-quality	14.79

Sample_T13B_k141_148656	17346	11	0	8	Low-quality	14.79
Sample_T4B_k141_228664	22856	30	1	14	Low-quality	14.79
Sample_C13A_k141_75346	8341	14	6	0	Low-quality	14.79
Sample_T8C_k141_6397	6181	15	4	0	Low-quality	14.79
Sample_C14C_k141_49926	5652	7	3	0	Low-quality	14.78
Sample_C3C_k141_122797	6320	3	0	1	Low-quality	14.77
Sample_T14B_k141_3628	9113	7	0	2	Low-quality	14.77
Sample_C10A_k141_102956	9147	13	2	0	Low-quality	14.76
Sample_C1A_k141_55052	9534	29	1	0	Low-quality	14.76
Sample_C3A_k141_23681	5936	4	1	0	Low-quality	14.76
Sample_T15B_k141_26532	5939	11	1	0	Low-quality	14.76
Sample_C2A_k141_31107	21935	17	1	14	Low-quality	14.75
Sample_T17C_k141_9634	15125	13	0	6	Low-quality	14.75
Sample_T12B_k141_113091	5279	10	0	0	Low-quality	14.75
Sample_T10A_k141_64446	5030	15	1	0	Low-quality	14.74
Sample_T6B_k141_184642	27176	30	1	3	Low-quality	14.74
Sample_T3B_k141_94699	45696	45	3	10	Low-quality	14.73
Sample_C12A_k141_98022	5206	8	2	0	Low-quality	14.73
Sample_T10B_k141_70164	8823	16	5	0	Low-quality	14.73
Sample_T12C_k141_110557	6495	14	3	0	Low-quality	14.73
Sample_C4B_k141_212714	6388	7	1	0	Low-quality	14.72
Sample_T2B_k141_136450	6713	5	3	0	Low-quality	14.72
Sample_T8C_k141_26813	5699	9	5	0	Low-quality	14.72
Sample_C3A_k141_124641	7895	3	0	1	Low-quality	14.71
Sample_T12A_k141_254416	9100	4	1	0	Low-quality	14.71
Sample_T8B_k141_85912	12787	15	1	5	Low-quality	14.71
Sample_C13C_k141_32849	5745	12	0	0	Low-quality	14.71
Sample_T12A_k141_306557	5894	11	5	0	Low-quality	14.71
Sample_T12C_k141_35120	7579	15	2	0	Low-quality	14.71
Sample_T15C_k141_51012	6124	9	1	0	Low-quality	14.71
Sample_T4B_k141_371216	5104	6	2	0	Low-quality	14.71
Sample_T10B_k141_21819	5057	10	0	0	Low-quality	14.7
Sample_T12C_k141_19598	5945	15	4	0	Low-quality	14.7
Sample_T3A_k141_72011	8796	12	0	0	Low-quality	14.69
Sample_C12A_k141_106032	5319	11	0	0	Low-quality	14.69
Sample_C14A_k141_170656	22337	24	6	0	Low-quality	14.69
Sample_C1A_k141_4146	7116	10	0	0	Low-quality	14.68
Sample_C2B_k141_285390	5424	11	2	0	Low-quality	14.68
Sample_T12A_k141_57935	6883	12	11	0	Low-quality	14.68

Sample_T11A_k141_13352	8203	11	4	1	Low-quality	14.67
Sample_T5B_k141_6173	6313	7	0	1	Low-quality	14.67
Sample_C10A_k141_122320	8075	17	2	1	Low-quality	14.67
Sample_T10A_k141_170874	5887	9	0	0	Low-quality	14.67
Sample_T10B_k141_115460	5729	4	0	1	Low-quality	14.66
Sample_C12C_k141_3559	5204	9	6	0	Low-quality	14.66
Sample_T5B_k141_3994	8284	11	2	2	Low-quality	14.66
Sample_C1C_k141_123982	11173	5	0	1	Low-quality	14.65
Sample_T4B_k141_271543	18063	15	1	9	Low-quality	14.65
Sample_C8A_k141_130878	5214	6	5	0	Low-quality	14.65
Sample_C8A_k141_81549	12212	10	1	0	Low-quality	14.65
Sample_C5A_k141_272863	14554	9	0	5	Low-quality	14.64
Sample_T2A_k141_193824	6806	7	0	0	Low-quality	14.64
Sample_C7B_k141_120087	9066	19	1	0	Low-quality	14.64
Sample_T13B_k141_181652	5836	12	1	0	Low-quality	14.64
Sample_C14A_k141_487	11161	5	0	1	Low-quality	14.63
Sample_C3A_k141_224722	11959	11	0	5	Low-quality	14.63
Sample_T8B_k141_31944	5092	8	0	1	Low-quality	14.62
Sample_C12B_k141_24598	7998	16	8	0	Low-quality	14.62
Sample_C4C_k141_5010	5265	8	6	0	Low-quality	14.62
Sample_T6B_k141_198785	11022	18	1	0	Low-quality	14.62
Sample_C3B_k141_260493	26184	34	1	1	Low-quality	14.61
Sample_T17C_k141_32668	6026	12	7	0	Low-quality	14.61
Sample_T2A_k141_257691	6374	7	1	0	Low-quality	14.61
Sample_T4B_k141_228890	6057	14	1	0	Low-quality	14.61
Sample_C10B_k141_84557	5719	11	1	2	Low-quality	14.6
Sample_C10A_k141_208124	5435	9	7	0	Low-quality	14.6
Sample_C6B_k141_80064	6631	10	1	1	Low-quality	14.6
Sample_T13C_k141_67362_fragment_3	22808	23	0	3	Low-quality	14.6
Sample_T17B_k141_8093	5134	8	3	0	Low-quality	14.6
Sample_T7B_k141_154099	6747	10	5	0	Low-quality	14.6
Sample_T14A_k141_188911	17682	23	2	2	Low-quality	14.59
Sample_C12B_k141_155455	6903	11	2	0	Low-quality	14.59
Sample_C2B_k141_303645	5085	5	1	0	Low-quality	14.59
Sample_C3A_k141_82265	6192	14	3	0	Low-quality	14.59
Sample_C5B_k141_24207	10577	9	5	0	Low-quality	14.59
Sample_T8A_k141_111019	9642	11	1	2	Low-quality	14.59
Sample_C4C_k141_165701	27735	18	0	14	Low-quality	14.58
Sample_T1A_k141_39020	6768	12	7	0	Low-quality	14.58

Sample_T11C_k141_17497	16550	19	0	4	Low-quality	14.57
Sample_C2A_k141_128674	6694	9	8	0	Low-quality	14.57
Sample_C3A_k141_27174_fragment_1	5967	8	5	0	Low-quality	14.57
Sample_T16C_k141_69405	9542	9	0	1	Low-quality	14.57
Sample_T17A_k141_40467	15485	18	0	6	Low-quality	14.56
Sample_C10A_k141_341591	7663	9	1	0	Low-quality	14.56
Sample_C2A_k141_241275	6232	10	0	0	Low-quality	14.56
Sample_C3C_k141_69058	9325	11	1	0	Low-quality	14.56
Sample_T15A_k141_66422	10474	6	0	3	Low-quality	14.55
Sample_T5C_k141_79892	23225	19	1	5	Low-quality	14.55
Sample_T6C_k141_107535	5612	4	4	0	Low-quality	14.55
Sample_C11A_k141_9988	40088	39	0	35	Low-quality	14.54
Sample_T4B_k141_179871	47633	49	1	30	Low-quality	14.54
Sample_C10A_k141_94978_fragment_1	11090	20	2	0	Low-quality	14.54
Sample_C2C_k141_45008	7655	15	4	0	Low-quality	14.54
Sample_C4B_k141_210716	9739	11	4	1	Low-quality	14.54
Sample_C8B_k141_31515	6764	14	3	0	Low-quality	14.54
Sample_C3A_k141_95205	8700	7	0	1	Low-quality	14.53
Sample_T3B_k141_174893	11879	15	0	2	Low-quality	14.53
Sample_T11B_k141_175	8041	9	0	4	Low-quality	14.52
Sample_C3B_k141_155820	5598	9	0	0	Low-quality	14.52
Sample_T5B_k141_29217	5950	5	3	0	Low-quality	14.52
Sample_C4C_k141_89048	11609	14	2	1	Low-quality	14.51
Sample_T14A_k141_236222	7601	11	1	1	Low-quality	14.51
Sample_C14C_k141_100925_fragment_1	12303	10	1	1	Low-quality	14.5
Sample_C9C_k141_62486_fragment_1	12421	14	10	0	Low-quality	14.5
Sample_C10B_k141_87857	9576	12	1	4	Low-quality	14.49
Sample_C4B_k141_28711	15625	13	0	5	Low-quality	14.49
Sample_T8C_k141_44120	7528	10	0	2	Low-quality	14.49
Sample_C11A_k141_90997	8415	7	5	1	Low-quality	14.49
Sample_C14A_k141_80890	6817	8	1	0	Low-quality	14.49
Sample_C10C_k141_133172	8789	21	1	0	Low-quality	14.48
Sample_T17B_k141_4241	11165	13	2	1	Low-quality	14.48
Sample_T5A_k141_23965	14355	12	3	0	Low-quality	14.48
Sample_T8A_k141_138630	6910	11	8	2	Low-quality	14.48
Sample_T14A_k141_198608	7643	6	0	3	Low-quality	14.47
Sample_T14B_k141_185734	8661	12	0	1	Low-quality	14.47
Sample_C6B_k141_70922	8799	16	3	0	Low-quality	14.47
Sample_T5C_k141_16578	8517	20	2	0	Low-quality	14.47

Sample_C14C_k141_115757	8537	11	0	0	Low-quality	14.46
Sample_C5B_k141_9814	10068	11	1	3	Low-quality	14.46
Sample_T11B_k141_6145	5650	4	0	0	Low-quality	14.46
Sample_T2C_k141_41572	8065	6	0	1	Low-quality	14.46
Sample_C12A_k141_44325_fragment_1	7681	12	1	1	Low-quality	14.46
Sample_C1C_k141_118933	5158	8	2	0	Low-quality	14.46
Sample_T4B_k141_198943	8337	7	2	0	Low-quality	14.46
Sample_T6A_k141_156912	21544	25	4	1	Low-quality	14.46
Sample_C2C_k141_105348	6538	5	0	0	Low-quality	14.45
Sample_T6C_k141_55572	25478	27	1	18	Low-quality	14.45
Sample_T13A_k141_71260	6742	9	4	0	Low-quality	14.45
Sample_C6B_k141_33931	11664	17	1	3	Low-quality	14.44
Sample_T5C_k141_49133	10850	2	1	1	Low-quality	14.44
Sample_T12A_k141_177152	7004	16	0	0	Low-quality	14.44
Sample_T7A_k141_64884	5511	13	2	1	Low-quality	14.44
Sample_T5B_k141_24569	10735	19	1	2	Low-quality	14.43
Sample_C4B_k141_119522	6294	9	1	0	Low-quality	14.43
Sample_T18B_k141_98613_fragment_1	11879	12	0	1	Low-quality	14.43
Sample_C6C_k141_33152	5098	11	4	0	Low-quality	14.42
Sample_C8B_k141_134379	6459	12	0	0	Low-quality	14.42
Sample_T1C_k141_28256	6377	7	1	0	Low-quality	14.42
Sample_T11B_k141_138210	10149	11	0	5	Low-quality	14.41
Sample_C6C_k141_221538	5294	12	3	0	Low-quality	14.41
Sample_T10A_k141_8255_fragment_2	12081	18	5	0	Low-quality	14.41
Sample_T3B_k141_166150	8985	11	1	0	Low-quality	14.41
Sample_T8C_k141_23778	6069	14	4	0	Low-quality	14.41
Sample_C10A_k141_94114	7025	8	1	0	Low-quality	14.4
Sample_C2B_k141_66885	13695	19	1	9	Low-quality	14.4
Sample_T4B_k141_211694	6053	5	0	5	Low-quality	14.4
Sample_T12A_k141_302204	5686	6	2	0	Low-quality	14.4
Sample_C12A_k141_152185	26021	21	1	9	Low-quality	14.39
Sample_C14C_k141_152419	5504	4	0	0	Low-quality	14.39
Sample_C2C_k141_283501	18820	18	2	4	Low-quality	14.39
Sample_C2B_k141_25835	5966	15	4	0	Low-quality	14.39
Sample_T2B_k141_34700	7659	14	7	1	Low-quality	14.39
Sample_T13B_k141_129107	65157	69	3	37	Low-quality	14.38
Sample_C8A_k141_170833	6161	10	6	0	Low-quality	14.38
Sample_T16C_k141_140803	5691	13	4	0	Low-quality	14.38
Sample_T8B_k141_98210	8670	13	6	0	Low-quality	14.38

Sample_C13A_k141_1510	9667	11	0	2	Low-quality	14.37
Sample_C5A_k141_194454	6039	6	0	6	Low-quality	14.37
Sample_T5B_k141_54449	6039	6	0	6	Low-quality	14.37
Sample_C10C_k141_224077	6633	19	4	0	Low-quality	14.37
Sample_C2C_k141_33162	24527	29	12	1	Low-quality	14.37
Sample_T16B_k141_156366	25373	56	7	1	Low-quality	14.37
Sample_T18A_k141_56701	5408	6	3	0	Low-quality	14.37
Sample_C2B_k141_328542	10253	12	0	0	Low-quality	14.36
Sample_C2C_k141_105395	11606	14	1	6	Low-quality	14.36
Sample_C4C_k141_863	8634	11	1	0	Low-quality	14.36
Sample_T4B_k141_88522	24575	15	0	9	Low-quality	14.35
Sample_C6B_k141_59023	8323	14	2	0	Low-quality	14.35
Sample_T10A_k141_3199	9160	15	1	0	Low-quality	14.35
Sample_T3A_k141_46477	5384	10	1	0	Low-quality	14.35
Sample_T6B_k141_166737	8710	20	0	0	Low-quality	14.35
Sample_C2A_k141_188264	11717	12	0	2	Low-quality	14.34
Sample_C2C_k141_302809	5441	1	0	0	Low-quality	14.34
Sample_T13C_k141_31614	8583	7	0	2	Low-quality	14.34
Sample_T1C_k141_16798	25035	23	0	17	Low-quality	14.34
Sample_T8B_k141_40441	6643	10	0	0	Low-quality	14.34
Sample_T7A_k141_162490	5336	11	0	0	Low-quality	14.34
Sample_C3C_k141_10966	8257	16	1	0	Low-quality	14.33
Sample_T10C_k141_3143	18702	35	0	4	Low-quality	14.33
Sample_T17B_k141_28523	14870	22	7	0	Low-quality	14.33
Sample_T6A_k141_224684	11171	4	0	2	Low-quality	14.32
Sample_C11A_k141_139528	6952	10	5	0	Low-quality	14.32
Sample_C1B_k141_188680	7595	15	0	0	Low-quality	14.32
Sample_T6B_k141_141411	9526	7	0	3	Low-quality	14.31
Sample_C2A_k141_171427_fragment_1	10751	13	2	0	Low-quality	14.31
Sample_T5A_k141_23932	7813	8	0	0	Low-quality	14.31
Sample_T7A_k141_117869_fragment_1	10708	20	1	2	Low-quality	14.31
Sample_C2B_k141_274808	5884	12	4	0	Low-quality	14.3
Sample_C7A_k141_118062_fragment_1	11687	10	0	5	Low-quality	14.3
Sample_C10B_k141_5747	29136	21	0	12	Low-quality	14.29
Sample_C1B_k141_210000	8556	10	0	3	Low-quality	14.29
Sample_C9B_k141_6622	6004	5	0	5	Low-quality	14.29
Sample_T1C_k141_32408	25384	28	3	10	Low-quality	14.29
Sample_C10A_k141_72659	8551	12	0	5	Low-quality	14.28
Sample_C10A_k141_17816	22630	26	1	5	Low-quality	14.28

Sample_T5B_k141_67982	8220	8	0	2	Low-quality	14.28
Sample_C10A_k141_314807	9512	10	0	0	Low-quality	14.28
Sample_C1C_k141_174704	6911	11	1	0	Low-quality	14.28
Sample_C9C_k141_36418	9871	14	2	2	Low-quality	14.28
Sample_T13B_k141_14369	6128	13	4	0	Low-quality	14.28
Sample_T15A_k141_71748	25941	39	2	1	Low-quality	14.28
Sample_T16B_k141_122983	6280	11	2	0	Low-quality	14.28
Sample_T3C_k141_15016	9421	15	4	1	Low-quality	14.28
Sample_T11C_k141_128415	8271	9	0	1	Low-quality	14.27
Sample_T2A_k141_63794	17997	26	2	3	Low-quality	14.27
Sample_C9B_k141_94372	6318	3	3	0	Low-quality	14.26
Sample_T15B_k141_147714	17882	30	2	1	Low-quality	14.26
Sample_C13C_k141_61718_fragment_1	5561	11	5	0	Low-quality	14.26
Sample_C5C_k141_81641	7051	9	2	0	Low-quality	14.26
Sample_C6B_k141_79691	7076	20	0	0	Low-quality	14.26
Sample_T10A_k141_83761_fragment_1	6392	13	0	0	Low-quality	14.26
Sample_T11B_k141_73666	6164	8	0	0	Low-quality	14.26
Sample_T6C_k141_58195	12837	11	0	3	Low-quality	14.26
Sample_C12A_k141_35487	5245	7	1	1	Low-quality	14.25
Sample_C13A_k141_107703	23045	25	2	3	Low-quality	14.25
Sample_C9A_k141_69730	5300	9	3	0	Low-quality	14.25
Sample_C10C_k141_24408	5621	9	4	0	Low-quality	14.24
Sample_T18A_k141_30592	11421	12	1	5	Low-quality	14.24
Sample_C7A_k141_79942	11894	16	1	2	Low-quality	14.24
Sample_T3C_k141_108631	6181	7	0	0	Low-quality	14.24
Sample_T8C_k141_60646	7444	14	11	0	Low-quality	14.24
Sample_C10A_k141_33806	5238	6	2	0	Low-quality	14.23
Sample_C3B_k141_152498	6683	11	3	0	Low-quality	14.23
Sample_T4B_k141_145521	24899	34	8	2	Low-quality	14.23
Sample_C4A_k141_143754	5234	10	4	0	Low-quality	14.22
Sample_T3B_k141_99308	7075	11	8	0	Low-quality	14.22
Sample_T6A_k141_242453	5531	7	3	0	Low-quality	14.22
Sample_C13A_k141_29190	7859	10	0	2	Low-quality	14.21
Sample_C1C_k141_16061	6368	11	3	1	Low-quality	14.21
Sample_T12A_k141_9092	5833	12	1	0	Low-quality	14.21
Sample_T8C_k141_77304_fragment_2	23266	21	2	2	Low-quality	14.21
Sample_T13A_k141_126240	8696	10	0	2	Low-quality	14.2
Sample_C1B_k141_232682	11985	14	3	0	Low-quality	14.2
Sample_T12A_k141_161714	34626	36	10	0	Low-quality	14.2

Sample_T2A_k141_235261	6395	13	7	0	Low-quality	14.2
Sample_T5C_k141_8938	5627	9	4	0	Low-quality	14.2
Sample_T6A_k141_79318	5422	7	3	0	Low-quality	14.2
Sample_C1A_k141_202205	16659	16	0	14	Low-quality	14.19
Sample_C9B_k141_2967	10004	12	0	4	Low-quality	14.19
Sample_T11B_k141_153944	9376	12	1	2	Low-quality	14.19
Sample_T18B_k141_123882	11387	11	1	6	Low-quality	14.19
Sample_T4B_k141_300134	21359	19	0	13	Low-quality	14.19
Sample_C10A_k141_318946	8476	17	4	0	Low-quality	14.19
Sample_C2A_k141_14214	5773	9	1	0	Low-quality	14.19
Sample_T14C_k141_148171	9457	18	0	0	Low-quality	14.19
Sample_T14C_k141_27283	12282	5	1	1	Low-quality	14.18
Sample_C11A_k141_113301	8360	10	1	1	Low-quality	14.18
Sample_C2B_k141_131161_fragment_2	6687	11	3	0	Low-quality	14.17
Sample_C3C_k141_209580_fragment_1	6061	9	2	0	Low-quality	14.17
Sample_C3C_k141_138678	8988	5	0	0	Low-quality	14.16
Sample_T13B_k141_44019	8968	9	0	2	Low-quality	14.16
Sample_T8C_k141_89343	8462	8	0	1	Low-quality	14.16
Sample_C10C_k141_72499	5802	10	6	0	Low-quality	14.16
Sample_C6A_k141_179444_fragment_1	5476	11	3	0	Low-quality	14.16
Sample_T2C_k141_136669	12611	13	1	2	Low-quality	14.16
Sample_T7A_k141_6530	9353	7	1	1	Low-quality	14.15
Sample_T8A_k141_177202	7054	8	3	1	Low-quality	14.15
Sample_T10A_k141_179421	6000	7	1	0	Low-quality	14.15
Sample_C3C_k141_219246	5072	4	0	1	Low-quality	14.14
Sample_T2C_k141_68129	5497	9	1	0	Low-quality	14.14
Sample_T3A_k141_72140	15279	13	1	2	Low-quality	14.14
Sample_C11A_k141_164904	6613	7	4	0	Low-quality	14.14
Sample_C11C_k141_63683	11406	13	6	1	Low-quality	14.14
Sample_C1C_k141_112981	5904	5	1	0	Low-quality	14.14
Sample_C4C_k141_161327	7571	11	8	0	Low-quality	14.14
Sample_T17A_k141_35878	11547	13	0	8	Low-quality	14.13
Sample_C13C_k141_91412	7169	7	4	1	Low-quality	14.13
Sample_T3A_k141_94733	15614	7	1	2	Low-quality	14.13
Sample_T4B_k141_222020	6456	17	4	0	Low-quality	14.13
Sample_T6A_k141_215277	19741	26	1	1	Low-quality	14.13
Sample_T8C_k141_114363	8179	14	4	0	Low-quality	14.13
Sample_T12A_k141_298385	12706	9	0	3	Low-quality	14.12
Sample_T16B_k141_136035	5286	3	2	0	Low-quality	14.12

Sample_C12B_k141_93771	7160	18	1	0	Low-quality	14.12
Sample_T15B_k141_54448	5936	9	1	0	Low-quality	14.12
Sample_T8C_k141_93852	8446	8	0	2	Low-quality	14.11
Sample_C3B_k141_58320	6645	9	2	0	Low-quality	14.11
Sample_T16C_k141_83059	20407	34	4	1	Low-quality	14.11
Sample_T2B_k141_161310	5552	9	3	0	Low-quality	14.11
Sample_C4C_k141_140009	9044	8	0	2	Low-quality	14.1
Sample_C8A_k141_40642	7384	13	9	0	Low-quality	14.1
Sample_T8B_k141_197289	10052	12	0	4	Low-quality	14.09
Sample_C12A_k141_197202	7761	13	0	1	Low-quality	14.09
Sample_T14A_k141_242540	9822	9	0	0	Low-quality	14.09
Sample_T15B_k141_31545	5626	8	4	0	Low-quality	14.09
Sample_T3C_k141_53849	8948	11	0	0	Low-quality	14.09
Sample_T4C_k141_41143	5510	11	4	0	Low-quality	14.09
Sample_T8B_k141_264147	9722	17	0	1	Low-quality	14.09
Sample_C11B_k141_11231	50394	60	1	8	Low-quality	14.08
Sample_C12C_k141_117141_fragment_2	12215	19	4	0	Low-quality	14.08
Sample_C1A_k141_166182	6287	6	0	3	Low-quality	14.08
Sample_C3A_k141_158056	5538	7	3	0	Low-quality	14.08
Sample_T5A_k141_53720	6817	9	4	0	Low-quality	14.08
Sample_C11B_k141_46037	8131	8	1	2	Low-quality	14.07
Sample_C13B_k141_102741	29785	30	0	25	Low-quality	14.07
Sample_T3C_k141_71950	8303	10	0	4	Low-quality	14.07
Sample_T5C_k141_49726	7771	7	0	3	Low-quality	14.07
Sample_C2C_k141_199336	6859	10	1	1	Low-quality	14.07
Sample_T8C_k141_181843	8147	13	3	0	Low-quality	14.07
Sample_C6B_k141_108863	36232	27	0	23	Low-quality	14.06
Sample_C7A_k141_23153	44081	51	0	48	Low-quality	14.06
Sample_C11A_k141_77542	6650	14	3	0	Low-quality	14.06
Sample_C4A_k141_12768	6424	13	2	1	Low-quality	14.06
Sample_T7A_k141_121869	5337	11	6	0	Low-quality	14.06
Sample_T2C_k141_159051	10212	12	0	0	Low-quality	14.05
Sample_T5C_k141_187924	20906	22	1	1	Low-quality	14.05
Sample_T8A_k141_239617	7589	10	0	2	Low-quality	14.05
Sample_C10C_k141_111861	5274	7	1	0	Low-quality	14.05
Sample_C2A_k141_61106	5100	10	2	0	Low-quality	14.05
Sample_T8B_k141_279031	5783	7	2	0	Low-quality	14.05
Sample_C7B_k141_33025	5156	5	3	0	Low-quality	14.04
Sample_T6C_k141_123816	8480	5	5	0	Low-quality	14.04

Sample_T8A_k141_29152	28242	23	1	20	Low-quality	14.03
Sample_T5B_k141_33778	10295	7	0	1	Low-quality	14.03
Sample_C5A_k141_207025	19115	17	1	4	Low-quality	14.02
Sample_C8B_k141_107391	17772	18	2	3	Low-quality	14.02
Sample_T8B_k141_184328	5213	9	3	0	Low-quality	14.02
Sample_T8C_k141_13681	6100	12	10	0	Low-quality	14.02
Sample_C5A_k141_103087	5034	7	2	0	Low-quality	14.01
Sample_C7B_k141_94891	14961	12	0	2	Low-quality	14.01
Sample_C11B_k141_155583	7388	11	7	0	Low-quality	14.01
Sample_C1B_k141_180002	5004	7	2	0	Low-quality	14.01
Sample_C5A_k141_114905	13926	34	0	0	Low-quality	14.01
Sample_T11C_k141_59493	7658	14	3	1	Low-quality	14.01
Sample_T2C_k141_79285_fragment_1	5768	9	1	1	Low-quality	14.01
Sample_T4B_k141_353532	8242	13	5	0	Low-quality	14.01
Sample_C8A_k141_90447	5239	4	0	0	Low-quality	14
Sample_T2B_k141_135483	10508	14	0	2	Low-quality	14
Sample_C3A_k141_147631	5873	6	1	0	Low-quality	14
Sample_T14B_k141_166002	6812	9	5	0	Low-quality	14
Sample_T6B_k141_23846	5118	6	0	0	Low-quality	14
Sample_T4B_k141_364271	6333	16	1	0	Low-quality	13.99
Sample_C10B_k141_69579	27068	30	3	7	Low-quality	13.98
Sample_C12A_k141_39643	5722	8	0	4	Low-quality	13.98
Sample_C2A_k141_111618	7922	9	0	4	Low-quality	13.98
Sample_C4A_k141_36939	7676	11	0	2	Low-quality	13.98
Sample_C10A_k141_149075	5471	6	1	0	Low-quality	13.98
Sample_C11C_k141_116425	6582	14	1	0	Low-quality	13.98
Sample_C8C_k141_58955	5549	4	3	0	Low-quality	13.98
Sample_C11C_k141_50327	14584	9	1	2	Low-quality	13.97
Sample_C11C_k141_27560	7779	13	5	0	Low-quality	13.97
Sample_T11C_k141_117404	14005	8	0	2	Low-quality	13.96
Sample_C14A_k141_156111	5638	8	2	0	Low-quality	13.96
Sample_C1B_k141_220176	7313	6	1	0	Low-quality	13.96
Sample_C3C_k141_164546	5507	8	4	0	Low-quality	13.96
Sample_T15B_k141_25901	7563	13	6	0	Low-quality	13.96
Sample_T5A_k141_518	10646	14	9	0	Low-quality	13.96
Sample_C2C_k141_258082	6206	10	0	0	Low-quality	13.95
Sample_T12A_k141_170042	5458	9	3	0	Low-quality	13.95
Sample_C2B_k141_108339	12114	16	1	1	Low-quality	13.94
Sample_T4B_k141_98274	9392	11	3	1	Low-quality	13.94

Sample_T2C_k141_95653	8125	13	3	1	Low-quality	13.93
Sample_C1A_k141_228041	21951	19	0	4	Low-quality	13.93
Sample_C2B_k141_166686	5964	12	4	0	Low-quality	13.93
Sample_C8A_k141_876	5183	5	4	0	Low-quality	13.93
Sample_T13B_k141_102896_fragment_2	9859	10	0	3	Low-quality	13.93
Sample_T2A_k141_182864	6330	7	3	0	Low-quality	13.93
Sample_C5A_k141_117409_fragment_1	15043	15	0	2	Low-quality	13.92
Sample_T5C_k141_129467	7018	8	0	1	Low-quality	13.91
Sample_C12B_k141_17304	8882	4	0	3	Low-quality	13.89
Sample_C3A_k141_55911	25607	32	1	3	Low-quality	13.89
Sample_C8A_k141_48721	11351	12	0	4	Low-quality	13.89
Sample_T6A_k141_91918	8511	6	1	3	Low-quality	13.89
Sample_T14B_k141_73419	5652	7	2	0	Low-quality	13.89
Sample_T16B_k141_29981	9801	12	2	0	Low-quality	13.89
Sample_T16C_k141_82823	6711	9	2	1	Low-quality	13.89
Sample_T2A_k141_117570	6250	6	0	0	Low-quality	13.89
Sample_C8B_k141_111397	10654	8	0	2	Low-quality	13.88
Sample_C9C_k141_16662	8310	7	0	2	Low-quality	13.88
Sample_T12C_k141_140627	9350	11	0	2	Low-quality	13.88
Sample_T7C_k141_5410	5062	1	0	0	Low-quality	13.88
Sample_C10A_k141_338266	5539	13	2	0	Low-quality	13.88
Sample_C12B_k141_43952	5106	7	0	0	Low-quality	13.88
Sample_T10B_k141_86560	6670	10	3	0	Low-quality	13.88
Sample_T5B_k141_28080	5494	14	2	0	Low-quality	13.88
Sample_C10A_k141_35956	5820	8	6	0	Low-quality	13.87
Sample_C10A_k141_230136	8357	16	6	0	Low-quality	13.87
Sample_C2C_k141_155023	5666	7	5	0	Low-quality	13.87
Sample_C3A_k141_218002	6329	12	2	1	Low-quality	13.87
Sample_C8B_k141_49611	8472	15	4	0	Low-quality	13.87
Sample_T3B_k141_5160	5388	7	3	0	Low-quality	13.87
Sample_T8B_k141_17988	8225	17	2	0	Low-quality	13.87
Sample_C3B_k141_1373	6462	12	1	0	Low-quality	13.86
Sample_C12B_k141_95712	11409	16	3	1	Low-quality	13.86
Sample_T7A_k141_70996	5363	12	4	0	Low-quality	13.86
Sample_C2A_k141_143611	11192	12	1	2	Low-quality	13.85
Sample_T12B_k141_3913	10923	10	3	0	Low-quality	13.85
Sample_T13C_k141_28974	13451	8	0	4	Low-quality	13.85
Sample_C12A_k141_137097	5483	5	2	0	Low-quality	13.85
Sample_T12C_k141_113230	6301	13	9	0	Low-quality	13.85

Sample_T13C_k141_145199	5168	7	0	0	Low-quality	13.85
Sample_C8C_k141_59145	10604	6	0	2	Low-quality	13.84
Sample_C9C_k141_71966	5524	9	3	0	Low-quality	13.84
Sample_T12A_k141_139429	9291	11	7	0	Low-quality	13.84
Sample_T14C_k141_156787	7413	9	5	0	Low-quality	13.84
Sample_C4C_k141_239283	7756	7	0	2	Low-quality	13.83
Sample_T15C_k141_40461	42850	38	0	25	Low-quality	13.83
Sample_T18C_k141_62244	25495	29	1	6	Low-quality	13.83
Sample_C11B_k141_182845	6961	5	5	0	Low-quality	13.83
Sample_T15C_k141_137453	10049	9	0	2	Low-quality	13.82
Sample_C12B_k141_124224	6278	6	4	1	Low-quality	13.82
Sample_C13A_k141_46210	6758	7	6	0	Low-quality	13.82
Sample_C12A_k141_85303	15235	16	1	3	Low-quality	13.81
Sample_C6C_k141_209923	8216	11	1	0	Low-quality	13.81
Sample_T15C_k141_15120	7512	8	3	1	Low-quality	13.81
Sample_T17B_k141_37008	6498	4	0	0	Low-quality	13.81
Sample_T3B_k141_148724_fragment_2	18943	15	3	3	Low-quality	13.81
Sample_T4C_k141_94307	5866	7	2	0	Low-quality	13.81
Sample_T4B_k141_21961	7256	6	0	5	Low-quality	13.8
Sample_C11B_k141_42496	6606	13	6	0	Low-quality	13.8
Sample_T2A_k141_117499	5208	9	5	0	Low-quality	13.8
Sample_T8B_k141_79679	5609	11	4	0	Low-quality	13.8
Sample_T4A_k141_63078	9246	12	0	1	Low-quality	13.79
Sample_T5C_k141_125650	7692	5	0	1	Low-quality	13.79
Sample_C8C_k141_152058	9527	12	2	0	Low-quality	13.79
Sample_C8C_k141_156904	6215	6	1	0	Low-quality	13.79
Sample_T8C_k141_59432	5194	11	3	1	Low-quality	13.79
Sample_C7A_k141_149207	14726	14	1	3	Low-quality	13.78
Sample_C10A_k141_184269	20968	25	4	0	Low-quality	13.78
Sample_C9C_k141_39925	6476	12	10	0	Low-quality	13.78
Sample_T13C_k141_20938_fragment_2	23796	49	4	1	Low-quality	13.78
Sample_T14C_k141_94402	11348	11	7	1	Low-quality	13.78
Sample_C7A_k141_10776	6924	12	0	2	Low-quality	13.77
Sample_C12B_k141_50594	5025	5	3	0	Low-quality	13.77
Sample_T12B_k141_46304	5058	6	4	0	Low-quality	13.77
Sample_T3A_k141_47067	5330	8	7	0	Low-quality	13.77
Sample_C11A_k141_41323	11527	21	5	0	Low-quality	13.76
Sample_C11C_k141_182613	6158	8	4	0	Low-quality	13.76
Sample_C13C_k141_91124	7194	16	10	0	Low-quality	13.76

Sample_C9A_k141_79769	7667	6	0	2	Low-quality	13.75
Sample_T14A_k141_11356	8551	10	6	0	Low-quality	13.75
Sample_T4B_k141_30558	5832	6	1	0	Low-quality	13.75
Sample_C10B_k141_49175	6386	6	0	0	Low-quality	13.74
Sample_T10C_k141_28855	5142	7	0	1	Low-quality	13.74
Sample_T4A_k141_2283	5117	4	0	1	Low-quality	13.74
Sample_T4B_k141_273686	9799	7	0	3	Low-quality	13.74
Sample_C2B_k141_171774	16658	18	2	3	Low-quality	13.74
Sample_T2C_k141_149409	11757	15	2	0	Low-quality	13.74
Sample_C3A_k141_156553	7976	15	1	0	Low-quality	13.73
Sample_C10C_k141_178350	7923	12	1	0	Low-quality	13.73
Sample_T2C_k141_164200	5768	6	0	5	Low-quality	13.72
Sample_C1C_k141_128527	6183	9	5	0	Low-quality	13.72
Sample_C3A_k141_240795	5195	6	1	0	Low-quality	13.72
Sample_C7A_k141_3632	7549	14	0	1	Low-quality	13.72
Sample_T14C_k141_34686	6181	5	3	0	Low-quality	13.72
Sample_T18B_k141_49185	5454	14	3	0	Low-quality	13.72
Sample_C2A_k141_189766	22708	25	1	3	Low-quality	13.71
Sample_T12A_k141_287878	19992	19	0	17	Low-quality	13.71
Sample_T1A_k141_11672	5412	6	3	0	Low-quality	13.71
Sample_T5B_k141_42650_fragment_1	11208	9	1	3	Low-quality	13.71
Sample_T16C_k141_84223	11208	13	1	2	Low-quality	13.7
Sample_C9C_k141_27731	8112	10	6	0	Low-quality	13.7
Sample_T7A_k141_97889	7558	9	1	1	Low-quality	13.7
Sample_C9A_k141_50346	5207	5	0	4	Low-quality	13.69
Sample_C10A_k141_119836_fragment_2	9139	11	1	2	Low-quality	13.69
Sample_T14C_k141_28734	7977	11	0	1	Low-quality	13.68
Sample_C1A_k141_79849	5516	6	4	0	Low-quality	13.68
Sample_C8C_k141_109917	24459	41	3	1	Low-quality	13.68
Sample_C5A_k141_226784	8649	14	0	3	Low-quality	13.67
Sample_C14C_k141_146328	7277	11	3	0	Low-quality	13.67
Sample_C3A_k141_84987	5476	6	1	0	Low-quality	13.67
Sample_T4C_k141_123035_fragment_1	7123	12	1	2	Low-quality	13.67
Sample_T8C_k141_199639	11176	12	0	3	Low-quality	13.67
Sample_C4A_k141_162939	5906	7	1	0	Low-quality	13.66
Sample_T17A_k141_6614	5006	6	3	0	Low-quality	13.66
Sample_C13B_k141_100179	25174	25	1	13	Low-quality	13.65
Sample_C4A_k141_124393	27062	28	0	23	Low-quality	13.65
Sample_T10A_k141_86893	15451	11	0	2	Low-quality	13.65

Sample_T7A_k141_112990	11077	13	1	4	Low-quality	13.65
Sample_C10C_k141_151024_fragment_1	6072	11	5	0	Low-quality	13.65
Sample_C12A_k141_133917	5439	7	1	0	Low-quality	13.65
Sample_C1B_k141_116441	5320	13	4	0	Low-quality	13.65
Sample_T18A_k141_182878	8327	10	2	0	Low-quality	13.65
Sample_T12C_k141_98478	21188	23	1	8	Low-quality	13.64
Sample_T5C_k141_127924	36562	37	2	10	Low-quality	13.64
Sample_C2B_k141_69153	11076	20	1	0	Low-quality	13.64
Sample_T11B_k141_150235	6343	6	0	1	Low-quality	13.64
Sample_T12A_k141_146050	7586	13	0	3	Low-quality	13.63
Sample_T15A_k141_163446	28355	25	0	21	Low-quality	13.63
Sample_T7C_k141_45564	33243	37	4	0	Low-quality	13.63
Sample_C14A_k141_24500	14306	14	0	10	Low-quality	13.62
Sample_C3A_k141_58545	25116	24	1	4	Low-quality	13.62
Sample_T10A_k141_139244	5854	7	5	0	Low-quality	13.62
Sample_T14C_k141_39965	5222	7	1	0	Low-quality	13.62
Sample_T2A_k141_55812	23662	25	11	1	Low-quality	13.62
Sample_C6C_k141_80893	24879	34	1	19	Low-quality	13.61
Sample_C1C_k141_78361	5977	6	1	0	Low-quality	13.61
Sample_C8B_k141_36444	7839	11	4	0	Low-quality	13.61
Sample_T15C_k141_61432	24655	21	1	2	Low-quality	13.6
Sample_C11C_k141_113226	5738	10	1	0	Low-quality	13.6
Sample_T13A_k141_62510	8536	19	4	0	Low-quality	13.6
Sample_T6A_k141_49233	5317	10	4	0	Low-quality	13.6
Sample_C5A_k141_23603	6769	6	0	1	Low-quality	13.59
Sample_T12B_k141_62225	5588	4	0	1	Low-quality	13.59
Sample_T15C_k141_131505	8138	11	0	2	Low-quality	13.59
Sample_T5B_k141_67032	5713	5	0	4	Low-quality	13.59
Sample_T17B_k141_36498	6007	8	1	0	Low-quality	13.59
Sample_T2B_k141_117536	5216	14	0	0	Low-quality	13.59
Sample_T3A_k141_54097	6570	10	2	1	Low-quality	13.59
Sample_T8B_k141_43883	8138	12	0	0	Low-quality	13.59
Sample_T13A_k141_15799	5496	6	0	2	Low-quality	13.58
Sample_T16C_k141_125827	8367	13	0	2	Low-quality	13.58
Sample_C10B_k141_132855	5205	12	2	0	Low-quality	13.58
Sample_C8B_k141_23653	5465	9	1	0	Low-quality	13.58
Sample_C8C_k141_64448	5390	10	1	0	Low-quality	13.58
Sample_T17A_k141_22436_fragment_1	8237	15	6	2	Low-quality	13.58
Sample_T3A_k141_2166	8023	12	2	0	Low-quality	13.58

Sample_T8A_k141_246105	6367	10	5	0	Low-quality	13.58
Sample_T7A_k141_139281	10022	14	0	4	Low-quality	13.57
Sample_C3A_k141_97457	13739	10	0	3	Low-quality	13.56
Sample_C3B_k141_69381	8136	19	1	0	Low-quality	13.56
Sample_C4B_k141_181012	9693	10	1	1	Low-quality	13.56
Sample_C4C_k141_195647	7284	16	4	0	Low-quality	13.56
Sample_C12A_k141_208292	5157	3	0	3	Low-quality	13.55
Sample_C13C_k141_92866	8954	12	1	2	Low-quality	13.55
Sample_C2C_k141_21443	5165	5	0	3	Low-quality	13.55
Sample_T16A_k141_104074	5392	10	2	0	Low-quality	13.55
Sample_C4A_k141_5728	8871	9	0	2	Low-quality	13.54
Sample_T14A_k141_26374	12187	10	0	1	Low-quality	13.54
Sample_T12A_k141_278863	21799	28	2	0	Low-quality	13.54
Sample_T13B_k141_11631	7884	11	3	0	Low-quality	13.54
Sample_C11C_k141_9337	6038	5	0	3	Low-quality	13.53
Sample_C3B_k141_98683	17747	24	0	2	Low-quality	13.53
Sample_T11B_k141_68272	5060	3	3	0	Low-quality	13.53
Sample_C8A_k141_39034	5526	11	0	0	Low-quality	13.53
Sample_C14C_k141_102264	10924	15	1	0	Low-quality	13.52
Sample_C8B_k141_88840	8551	4	0	1	Low-quality	13.52
Sample_T12A_k141_35649	17632	16	0	12	Low-quality	13.52
Sample_C14B_k141_100279	12820	7	2	1	Low-quality	13.52
Sample_T12A_k141_142022	5015	4	1	0	Low-quality	13.52
Sample_T16B_k141_169709	5817	10	6	0	Low-quality	13.52
Sample_T8A_k141_90244	10365	16	3	0	Low-quality	13.52
Sample_T13C_k141_40687	5607	6	0	0	Low-quality	13.51
Sample_C7A_k141_11144	7239	8	0	0	Low-quality	13.51
Sample_T13B_k141_101355	8946	14	1	1	Low-quality	13.51
Sample_C1C_k141_62216	8082	11	0	2	Low-quality	13.5
Sample_C8C_k141_92956_fragment_1	11681	16	1	3	Low-quality	13.5
Sample_T1B_k141_16456	7606	9	1	0	Low-quality	13.5
Sample_T7B_k141_64192	14963	14	1	3	Low-quality	13.5
Sample_C10C_k141_216480	6417	6	0	2	Low-quality	13.49
Sample_T10C_k141_55412	8830	9	0	8	Low-quality	13.49
Sample_C2C_k141_147957	9144	9	0	1	Low-quality	13.49
Sample_C3A_k141_199074	15834	34	8	0	Low-quality	13.49
Sample_T4C_k141_115461	6378	7	4	0	Low-quality	13.49
Sample_T7A_k141_208447	7426	14	6	0	Low-quality	13.49
Sample_C12C_k141_65024	5502	8	2	1	Low-quality	13.48

Sample_T1A_k141_41539	6577	6	1	0	Low-quality	13.48
Sample_T14B_k141_79537	6789	13	2	1	Low-quality	13.48
Sample_T7B_k141_79113	5288	10	2	0	Low-quality	13.48
Sample_C3A_k141_229529	7639	13	1	1	Low-quality	13.47
Sample_C5A_k141_76967	21016	22	1	9	Low-quality	13.47
Sample_C7A_k141_8948	11012	11	0	4	Low-quality	13.47
Sample_C7B_k141_165184	16221	13	0	3	Low-quality	13.47
Sample_C9A_k141_108781	23721	45	2	0	Low-quality	13.46
Sample_C2B_k141_3896	12414	29	3	1	Low-quality	13.46
Sample_T11C_k141_102088	9020	9	4	0	Low-quality	13.46
Sample_C11A_k141_165905	6693	12	8	0	Low-quality	13.45
Sample_T13B_k141_170622	8130	5	1	0	Low-quality	13.45
Sample_C10B_k141_85504	20772	20	1	3	Low-quality	13.44
Sample_C12B_k141_105683	6532	4	0	0	Low-quality	13.44
Sample_C10A_k141_326784	7280	10	5	0	Low-quality	13.44
Sample_C5A_k141_222348_fragment_1	10246	15	2	2	Low-quality	13.44
Sample_C8B_k141_171442	5467	9	0	2	Low-quality	13.44
Sample_T12A_k141_322514	5558	6	2	0	Low-quality	13.44
Sample_T2B_k141_123244	6120	8	2	0	Low-quality	13.44
Sample_T7C_k141_38774	6095	18	1	0	Low-quality	13.44
Sample_C13A_k141_27745	5446	4	0	0	Low-quality	13.43
Sample_C10C_k141_62357_fragment_1	5255	12	2	0	Low-quality	13.43
Sample_C10A_k141_108322	13119	16	0	1	Low-quality	13.42
Sample_C2B_k141_29515	12048	10	0	2	Low-quality	13.42
Sample_T12C_k141_35737	21773	28	1	2	Low-quality	13.42
Sample_T13B_k141_19965	22652	20	1	12	Low-quality	13.42
Sample_C12A_k141_18613_fragment_1	13110	19	7	0	Low-quality	13.41
Sample_C3A_k141_237739_fragment_1	19590	36	2	6	Low-quality	13.41
Sample_T11C_k141_133246	6228	8	0	1	Low-quality	13.4
Sample_T13C_k141_73410	14001	9	0	2	Low-quality	13.4
Sample_C10C_k141_113715	8658	12	3	0	Low-quality	13.4
Sample_C13A_k141_6904	6351	8	1	0	Low-quality	13.4
Sample_T13B_k141_162641	6995	10	3	0	Low-quality	13.4
Sample_T2B_k141_104539	14468	12	0	3	Low-quality	13.39
Sample_T3A_k141_31246	10941	9	0	2	Low-quality	13.39
Sample_T7A_k141_42305	9260	11	0	8	Low-quality	13.39
Sample_C3A_k141_123700	5757	7	2	0	Low-quality	13.39
Sample_C8B_k141_112738	7407	9	0	0	Low-quality	13.39
Sample_T12A_k141_49933	5490	9	3	0	Low-quality	13.39

Sample_T2C_k141_36596	6440	7	0	1	Low-quality	13.39
Sample_T5C_k141_48552	5377	6	5	0	Low-quality	13.39
Sample_T6A_k141_39048	5458	6	4	0	Low-quality	13.39
Sample_C14C_k141_121617	24964	25	1	19	Low-quality	13.38
Sample_C11A_k141_124311	9131	12	4	0	Low-quality	13.38
Sample_C12C_k141_77888	10057	6	0	2	Low-quality	13.38
Sample_T10B_k141_87631	5320	10	7	0	Low-quality	13.38
Sample_C10A_k141_29158	6291	4	1	0	Low-quality	13.36
Sample_C3B_k141_69898	5342	8	2	1	Low-quality	13.36
Sample_T17B_k141_34999	6732	13	6	0	Low-quality	13.36
Sample_C10C_k141_44016	6836	7	0	2	Low-quality	13.35
Sample_T11A_k141_51228	17557	18	0	11	Low-quality	13.35
Sample_T13B_k141_7057	23309	17	0	14	Low-quality	13.35
Sample_C4A_k141_85877	6287	6	4	0	Low-quality	13.35
Sample_T10A_k141_90042	6217	9	3	0	Low-quality	13.35
Sample_T17C_k141_18542	9083	15	2	2	Low-quality	13.35
Sample_T7C_k141_129183	5045	9	0	0	Low-quality	13.35
Sample_C7B_k141_122169	5180	14	4	0	Low-quality	13.34
Sample_T12C_k141_80583	5762	7	1	3	Low-quality	13.34
Sample_T16C_k141_78287	22864	20	1	14	Low-quality	13.34
Sample_C6B_k141_173477	5477	5	4	0	Low-quality	13.34
Sample_C8C_k141_25075	10475	12	0	1	Low-quality	13.34
Sample_T4A_k141_88554	7058	9	2	0	Low-quality	13.34
Sample_C10A_k141_220096	10896	11	0	3	Low-quality	13.33
Sample_C10C_k141_179322	23930	28	2	8	Low-quality	13.33
Sample_T11B_k141_5735	10071	15	0	2	Low-quality	13.33
Sample_T11B_k141_11038	5602	6	0	6	Low-quality	13.33
Sample_T12B_k141_119108	35512	36	1	6	Low-quality	13.33
Sample_C8A_k141_590	6972	12	3	1	Low-quality	13.33
Sample_C9B_k141_48821	6224	12	7	0	Low-quality	13.33
Sample_T10B_k141_154907	19523	32	2	0	Low-quality	13.33
Sample_T12C_k141_54641	9253	12	2	0	Low-quality	13.33
Sample_T13B_k141_105919	6106	7	4	0	Low-quality	13.33
Sample_T6C_k141_120056	5583	8	4	0	Low-quality	13.33
Sample_C12C_k141_154416	9205	8	1	3	Low-quality	13.32
Sample_T15C_k141_136455	23251	15	0	14	Low-quality	13.32
Sample_T7A_k141_23559	7286	6	0	2	Low-quality	13.32
Sample_C14C_k141_135486	6937	17	10	0	Low-quality	13.32
Sample_C6B_k141_149611	8996	7	0	3	Low-quality	13.31

Sample_T17C_k141_35453	7766	8	0	0	Low-quality	13.31
Sample_C2A_k141_255418	5566	14	3	0	Low-quality	13.31
Sample_C10A_k141_184869	10788	11	1	0	Low-quality	13.3
Sample_C13A_k141_42779	23645	23	0	5	Low-quality	13.3
Sample_C2B_k141_116757	10866	10	0	3	Low-quality	13.3
Sample_C3B_k141_54060	6829	10	0	1	Low-quality	13.3
Sample_T5A_k141_58756	6589	5	0	4	Low-quality	13.3
Sample_T5A_k141_56759	7597	13	1	0	Low-quality	13.3
Sample_T8C_k141_160613	12992	27	4	1	Low-quality	13.3
Sample_C5B_k141_62658	9622	13	0	0	Low-quality	13.29
Sample_T2A_k141_225039	5971	11	5	0	Low-quality	13.29
Sample_C3A_k141_96387	5154	9	2	0	Low-quality	13.28
Sample_T11C_k141_129368_fragment_1	23790	30	1	1	Low-quality	13.28
Sample_T12B_k141_150190	26653	51	3	0	Low-quality	13.28
Sample_C12A_k141_86960	26143	26	0	4	Low-quality	13.27
Sample_T4B_k141_344965	5586	11	0	1	Low-quality	13.27
Sample_T6C_k141_78999	7106	11	3	0	Low-quality	13.27
Sample_C11A_k141_28496	6252	6	3	0	Low-quality	13.27
Sample_C13A_k141_65468	8841	18	3	1	Low-quality	13.27
Sample_T12A_k141_342305	5426	8	1	0	Low-quality	13.27
Sample_T5A_k141_37513	5419	9	4	0	Low-quality	13.27
Sample_C3C_k141_52052	18818	18	1	6	Low-quality	13.26
Sample_C14C_k141_172657	22006	27	8	12	Low-quality	13.25
Sample_T16C_k141_75755	8360	13	1	3	Low-quality	13.25
Sample_T6A_k141_61369	20996	18	1	14	Low-quality	13.25
Sample_C1C_k141_3533	6209	8	2	0	Low-quality	13.25
Sample_C2A_k141_290854	5511	5	3	0	Low-quality	13.25
Sample_T12A_k141_293079	5340	6	3	0	Low-quality	13.25
Sample_T7C_k141_92425	5421	9	1	1	Low-quality	13.24
Sample_C5C_k141_31231	5571	7	4	0	Low-quality	13.24
Sample_C9C_k141_41028	5847	11	3	1	Low-quality	13.24
Sample_T10B_k141_82794	6054	5	3	0	Low-quality	13.24
Sample_T18B_k141_148871_fragment_2	16188	13	5	3	Low-quality	13.24
Sample_C13A_k141_95391	28009	26	0	23	Low-quality	13.23
Sample_C7C_k141_36149	5592	10	0	0	Low-quality	13.23
Sample_T2A_k141_139879	6118	8	4	1	Low-quality	13.23
Sample_T8A_k141_78110	27250	22	3	1	Low-quality	13.23
Sample_T11C_k141_156079	5224	6	0	2	Low-quality	13.22
Sample_T12A_k141_324395	6932	9	3	0	Low-quality	13.22

Sample_C8B_k141_93241	9889	10	0	2	Low-quality	13.22
Sample_C1C_k141_83872	8479	8	0	3	Low-quality	13.21
Sample_C8C_k141_50242	15687	18	0	10	Low-quality	13.21
Sample_T12B_k141_9616	5619	5	0	3	Low-quality	13.21
Sample_C5C_k141_63636	5573	4	0	1	Low-quality	13.2
Sample_C9A_k141_85303	5119	5	0	0	Low-quality	13.2
Sample_C14A_k141_204073	5252	11	8	0	Low-quality	13.2
Sample_T12A_k141_3359	9094	17	1	0	Low-quality	13.2
Sample_C11A_k141_69209	23457	19	1	5	Low-quality	13.19
Sample_C1A_k141_204627	20781	28	2	4	Low-quality	13.19
Sample_C6B_k141_195510	6855	5	0	1	Low-quality	13.19
Sample_T11B_k141_1631	13197	10	0	1	Low-quality	13.19
Sample_C10C_k141_60226	5886	8	5	0	Low-quality	13.19
Sample_C6C_k141_51735	8397	9	1	1	Low-quality	13.19
Sample_C7A_k141_29863	5288	3	2	0	Low-quality	13.18
Sample_C9B_k141_60031	6662	3	3	0	Low-quality	13.18
Sample_T3C_k141_90523	21044	16	1	3	Low-quality	13.18
Sample_T8B_k141_202983	5673	8	1	0	Low-quality	13.18
Sample_C3A_k141_182316	5635	7	2	0	Low-quality	13.17
Sample_C3B_k141_169504	14287	26	2	0	Low-quality	13.17
Sample_T6C_k141_252564	5537	6	2	0	Low-quality	13.17
Sample_T7A_k141_147131	5373	5	4	0	Low-quality	13.17
Sample_T7B_k141_111763	5105	4	3	0	Low-quality	13.17
Sample_C1B_k141_105453	5527	8	0	3	Low-quality	13.16
Sample_T12A_k141_313941	21626	27	1	10	Low-quality	13.16
Sample_C10A_k141_65535	7449	10	5	0	Low-quality	13.16
Sample_C3B_k141_124360	7602	14	1	0	Low-quality	13.16
Sample_C6A_k141_170982	5301	7	4	0	Low-quality	13.16
Sample_T17C_k141_28710	6561	10	5	0	Low-quality	13.16
Sample_C6B_k141_60869	35022	40	1	13	Low-quality	13.15
Sample_C1C_k141_74268	5511	6	0	0	Low-quality	13.15
Sample_C6B_k141_112398	6082	8	3	0	Low-quality	13.15
Sample_C5A_k141_55955	6036	3	1	0	Low-quality	13.14
Sample_C12A_k141_175042	5495	5	4	0	Low-quality	13.14
Sample_T13B_k141_23493	8682	10	1	0	Low-quality	13.14
Sample_C3A_k141_148617	7458	12	0	0	Low-quality	13.13
Sample_C12B_k141_154224	5035	8	2	0	Low-quality	13.12
Sample_C3C_k141_87556_fragment_1	5031	9	3	0	Low-quality	13.12
Sample_C4C_k141_82219	5724	12	2	0	Low-quality	13.12

Sample_C3A_k141_87219	5550	8	4	0	Low-quality	13.11
Sample_T14B_k141_8490	5509	9	2	0	Low-quality	13.11
Sample_C10B_k141_121716	18485	13	0	11	Low-quality	13.1
Sample_C12A_k141_45037	5779	7	3	0	Low-quality	13.1
Sample_C13A_k141_59448	6300	17	2	0	Low-quality	13.1
Sample_C5C_k141_87822	6782	12	1	1	Low-quality	13.1
Sample_T12C_k141_98858	5614	8	3	0	Low-quality	13.09
Sample_T17B_k141_35466	11886	15	3	0	Low-quality	13.09
Sample_T5A_k141_38901	5498	14	3	0	Low-quality	13.09
Sample_T8C_k141_23350	5471	15	10	0	Low-quality	13.09
Sample_C10C_k141_114242	5323	6	5	0	Low-quality	13.08
Sample_C4A_k141_18991	5659	7	3	0	Low-quality	13.08
Sample_T12A_k141_207339	5506	10	5	0	Low-quality	13.08
Sample_T13A_k141_62908	6986	19	5	0	Low-quality	13.08
Sample_T13C_k141_95203	6461	11	8	0	Low-quality	13.08
Sample_T7B_k141_46514_fragment_1	32488	39	2	7	Low-quality	13.08
Sample_C9A_k141_92547	6375	9	0	2	Low-quality	13.07
Sample_T4B_k141_90152	5690	8	0	1	Low-quality	13.07
Sample_C12C_k141_77367	8749	15	1	1	Low-quality	13.07
Sample_T17A_k141_25834	5555	8	2	0	Low-quality	13.07
Sample_C4B_k141_256453	8384	8	0	3	Low-quality	13.06
Sample_T10C_k141_13609	12922	13	0	2	Low-quality	13.06
Sample_T1A_k141_37697	11756	11	0	2	Low-quality	13.06
Sample_C5C_k141_104550	5129	9	0	0	Low-quality	13.06
Sample_T15C_k141_57537	5419	4	0	0	Low-quality	13.06
Sample_C6A_k141_201280	27994	34	2	16	Low-quality	13.05
Sample_C6B_k141_96533	10669	11	1	2	Low-quality	13.05
Sample_T10C_k141_35498	5485	5	0	5	Low-quality	13.05
Sample_T6C_k141_142712	8898	10	1	1	Low-quality	13.05
Sample_T8A_k141_164214	31693	38	1	7	Low-quality	13.05
Sample_T4B_k141_307454	28724	27	3	11	Low-quality	13.04
Sample_T8B_k141_60627	23369	18	1	2	Low-quality	13.04
Sample_T10B_k141_143544	6990	11	5	0	Low-quality	13.04
Sample_T7A_k141_53502	11224	10	0	0	Low-quality	13.04
Sample_T8B_k141_114065	5404	7	5	0	Low-quality	13.03
Sample_T8B_k141_70896	6548	13	2	0	Low-quality	13.03
Sample_C13A_k141_21856	13284	12	2	1	Low-quality	13.02
Sample_C14C_k141_86844	7557	13	2	0	Low-quality	13.02
Sample_C3A_k141_62205	5849	10	1	0	Low-quality	13.02

Sample_T11B_k141_138014	9746	12	0	1	Low-quality	13.02
Sample_T15A_k141_3165	10640	10	0	0	Low-quality	13.02
Sample_T4C_k141_104534	5080	7	6	0	Low-quality	13.02
Sample_T6A_k141_113283	5115	8	4	0	Low-quality	13.02
Sample_C11B_k141_218471	6042	4	0	4	Low-quality	13.01
Sample_C7B_k141_24018	20111	19	1	6	Low-quality	13.01
Sample_T13A_k141_28826	8140	7	0	2	Low-quality	13.01
Sample_T5B_k141_8398	28112	41	1	8	Low-quality	13.01
Sample_C5A_k141_233468	8318	18	2	0	Low-quality	13.01
Sample_C9B_k141_97192	12665	7	0	1	Low-quality	13
Sample_C11A_k141_146679	8441	9	1	0	Low-quality	13
Sample_C13B_k141_108074	8680	12	1	0	Low-quality	12.99
Sample_T10B_k141_145155	16433	35	2	0	Low-quality	12.99
Sample_T15C_k141_953_fragment_1	10496	20	1	0	Low-quality	12.99
Sample_C2B_k141_24136	10738	13	1	1	Low-quality	12.98
Sample_T17A_k141_36714	7771	11	0	3	Low-quality	12.98
Sample_T7B_k141_24671	14046	10	0	2	Low-quality	12.98
Sample_C12C_k141_147728	6530	8	3	0	Low-quality	12.98
Sample_T12C_k141_184303	9954	21	0	0	Low-quality	12.98
Sample_C5C_k141_43169	9783	10	0	7	Low-quality	12.97
Sample_C1C_k141_82486	6954	8	6	0	Low-quality	12.97
Sample_C12B_k141_105565	7566	7	0	0	Low-quality	12.96
Sample_T6B_k141_35191	6305	4	0	0	Low-quality	12.96
Sample_C2A_k141_264067	5187	12	1	0	Low-quality	12.96
Sample_T17C_k141_22872	5948	13	3	0	Low-quality	12.96
Sample_T5C_k141_177071	10155	6	0	2	Low-quality	12.96
Sample_C2C_k141_235783	8199	8	0	0	Low-quality	12.95
Sample_T16B_k141_149012	13630	13	3	3	Low-quality	12.95
Sample_T2C_k141_259097	9034	10	0	1	Low-quality	12.95
Sample_C4C_k141_121393	18829	22	1	3	Low-quality	12.95
Sample_T6B_k141_201490	10364	14	3	2	Low-quality	12.95
Sample_T7A_k141_204143	5829	12	3	1	Low-quality	12.95
Sample_T8A_k141_215528	5922	5	4	0	Low-quality	12.95
Sample_C10A_k141_156516	10212	11	0	2	Low-quality	12.94
Sample_C10B_k141_19064	11634	12	0	3	Low-quality	12.93
Sample_C6C_k141_1115	7739	11	0	1	Low-quality	12.93
Sample_T2B_k141_281945	25593	20	1	4	Low-quality	12.93
Sample_T7B_k141_37732	6001	18	2	1	Low-quality	12.93
Sample_C9B_k141_26991	5808	11	3	0	Low-quality	12.93

Sample_T8B_k141_76826	6947	18	3	0	Low-quality	12.93
Sample_T4B_k141_133111	5534	6	0	1	Low-quality	12.92
Sample_T6C_k141_34132	7058	7	0	1	Low-quality	12.92
Sample_C3C_k141_256576	7103	8	1	2	Low-quality	12.91
Sample_C4B_k141_161389	15797	13	0	9	Low-quality	12.91
Sample_T6A_k141_68526	8533	8	1	3	Low-quality	12.91
Sample_C11C_k141_174042	21011	48	4	0	Low-quality	12.91
Sample_C12C_k141_62338	12055	6	0	3	Low-quality	12.91
Sample_T17B_k141_27230	11637	16	3	0	Low-quality	12.91
Sample_T5B_k141_19476	6045	6	4	0	Low-quality	12.91
Sample_T7C_k141_13617	7681	19	1	0	Low-quality	12.91
Sample_T8B_k141_209530	6710	10	5	0	Low-quality	12.91
Sample_T12A_k141_82425	5141	6	4	0	Low-quality	12.9
Sample_T2A_k141_229847	11598	9	0	2	Low-quality	12.89
Sample_C4C_k141_107741	5429	4	2	0	Low-quality	12.89
Sample_C10C_k141_95529	6130	3	3	0	Low-quality	12.88
Sample_C5A_k141_145369	5499	5	0	0	Low-quality	12.88
Sample_C10C_k141_106642	6314	9	3	0	Low-quality	12.87
Sample_T16B_k141_106259	7698	9	0	0	Low-quality	12.86
Sample_C12B_k141_92644	6067	11	4	0	Low-quality	12.86
Sample_T15B_k141_146491	6343	8	7	0	Low-quality	12.86
Sample_T6A_k141_119455	9494	8	0	2	Low-quality	12.86
Sample_T6A_k141_14426	13337	15	3	1	Low-quality	12.86
Sample_T18C_k141_48840	23689	23	1	2	Low-quality	12.85
Sample_T3C_k141_49516	5205	13	3	1	Low-quality	12.85
Sample_T7B_k141_120888	17842	21	1	4	Low-quality	12.85
Sample_C6B_k141_14819	8487	9	1	0	Low-quality	12.84
Sample_T12B_k141_128771	5480	10	5	1	Low-quality	12.84
Sample_T3B_k141_70974_fragment_1	9110	12	0	0	Low-quality	12.84
Sample_T12A_k141_69858	5554	4	0	0	Low-quality	12.83
Sample_C2A_k141_178972	10498	15	3	1	Low-quality	12.83
Sample_C6C_k141_209882	6784	9	1	0	Low-quality	12.83
Sample_T16C_k141_133185	12628	13	0	7	Low-quality	12.82
Sample_T8C_k141_91111	8003	10	0	2	Low-quality	12.82
Sample_T7A_k141_148334	5037	7	0	0	Low-quality	12.82
Sample_C14C_k141_127229	15774	17	0	6	Low-quality	12.81
Sample_T14A_k141_132488	13861	10	0	2	Low-quality	12.81
Sample_T3A_k141_45552	5886	4	0	0	Low-quality	12.81
Sample_T12B_k141_1775	5491	7	5	0	Low-quality	12.81

Sample_T4B_k141_120955	7402	13	5	0	Low-quality	12.81
Sample_C2B_k141_324561	5380	5	0	4	Low-quality	12.8
Sample_C2B_k141_50857	9550	10	0	3	Low-quality	12.8
Sample_T2A_k141_94493	9825	5	0	2	Low-quality	12.8
Sample_C2C_k141_187859	5577	10	3	0	Low-quality	12.8
Sample_C3C_k141_259376	5445	11	1	0	Low-quality	12.8
Sample_C9A_k141_105938	5747	8	5	0	Low-quality	12.8
Sample_T18B_k141_5737	7895	19	0	1	Low-quality	12.8
Sample_T5B_k141_66019	5840	6	4	0	Low-quality	12.8
Sample_T8C_k141_141896	14228	21	3	3	Low-quality	12.8
Sample_T8C_k141_195169	5070	9	5	0	Low-quality	12.8
Sample_C1B_k141_124546	24541	7	0	4	Low-quality	12.79
Sample_C5B_k141_13231	5144	8	3	0	Low-quality	12.79
Sample_C9A_k141_117340	6086	8	6	0	Low-quality	12.79
Sample_C1C_k141_10084	5397	3	0	2	Low-quality	12.78
Sample_T14B_k141_214475	9470	12	0	3	Low-quality	12.78
Sample_T14B_k141_109461	13357	9	1	6	Low-quality	12.78
Sample_C2B_k141_312995	10222	7	0	2	Low-quality	12.78
Sample_C4B_k141_258313	6021	10	4	0	Low-quality	12.78
Sample_T13C_k141_101220	6716	6	0	0	Low-quality	12.78
Sample_T1A_k141_12594	10885	16	2	1	Low-quality	12.78
Sample_C12B_k141_182604	8464	10	0	2	Low-quality	12.77
Sample_C14C_k141_66068	19282	16	1	7	Low-quality	12.77
Sample_C4B_k141_137265	5347	11	4	0	Low-quality	12.77
Sample_T5C_k141_171399	7675	12	7	0	Low-quality	12.77
Sample_C13A_k141_93836	5786	9	1	0	Low-quality	12.76
Sample_C7A_k141_132881	9330	11	1	2	Low-quality	12.76
Sample_T15A_k141_78626	8435	5	1	0	Low-quality	12.76
Sample_T16B_k141_46782	37212	35	0	22	Low-quality	12.76
Sample_C10A_k141_163828	5055	10	3	0	Low-quality	12.76
Sample_C2A_k141_234194	5032	10	2	0	Low-quality	12.76
Sample_T15C_k141_76470	6039	5	3	1	Low-quality	12.76
Sample_T6B_k141_61198	5588	11	8	0	Low-quality	12.76
Sample_T4C_k141_3792	9716	9	1	3	Low-quality	12.75
Sample_C3C_k141_255986	8405	8	0	0	Low-quality	12.75
Sample_T11B_k141_25664	6075	6	0	0	Low-quality	12.75
Sample_T15B_k141_85956	6105	11	0	1	Low-quality	12.75
Sample_T17A_k141_40130_fragment_1	7256	8	1	0	Low-quality	12.75
Sample_T13A_k141_104999	5534	7	0	0	Low-quality	12.74

Sample_C14A_k141_84796	5963	5	3	0	Low-quality	12.74
Sample_C14B_k141_70564	6898	10	3	1	Low-quality	12.74
Sample_T7C_k141_56737	5141	6	5	0	Low-quality	12.74
Sample_T10B_k141_81790	5783	15	2	0	Low-quality	12.73
Sample_C5C_k141_88255	20943	25	5	0	Low-quality	12.73
Sample_C9A_k141_2953	5703	9	2	1	Low-quality	12.73
Sample_C10A_k141_137540	23767	27	1	13	Low-quality	12.72
Sample_T2B_k141_180106	8863	10	0	6	Low-quality	12.72
Sample_T3B_k141_139885	5386	11	3	0	Low-quality	12.72
Sample_T5B_k141_57250	10394	7	0	1	Low-quality	12.72
Sample_T11B_k141_102864	5252	8	0	0	Low-quality	12.72
Sample_C1B_k141_218449	6685	5	0	2	Low-quality	12.71
Sample_T15A_k141_180983	11633	17	1	1	Low-quality	12.71
Sample_T8A_k141_17170	33091	35	2	3	Low-quality	12.71
Sample_T5B_k141_43132	5086	11	7	0	Low-quality	12.71
Sample_C3A_k141_59861	5133	4	0	0	Low-quality	12.7
Sample_T10A_k141_31556	10028	20	1	1	Low-quality	12.7
Sample_T2C_k141_12206	5902	6	0	1	Low-quality	12.7
Sample_T4B_k141_272036	10261	10	1	0	Low-quality	12.7
Sample_C1C_k141_106900	5901	8	5	0	Low-quality	12.7
Sample_C6C_k141_13834	5323	9	2	0	Low-quality	12.7
Sample_T14A_k141_196750	7200	18	3	0	Low-quality	12.7
Sample_T18A_k141_60866	7705	16	0	0	Low-quality	12.7
Sample_C1A_k141_131415	8591	8	0	2	Low-quality	12.69
Sample_C7B_k141_104782	22749	18	1	7	Low-quality	12.69
Sample_T8A_k141_208312	6500	6	0	4	Low-quality	12.69
Sample_C9A_k141_3067	6760	7	0	0	Low-quality	12.69
Sample_T7A_k141_171228	7349	11	0	0	Low-quality	12.68
Sample_T8B_k141_150414	5517	10	5	0	Low-quality	12.68
Sample_T8C_k141_206113_fragment_1	11750	10	2	0	Low-quality	12.68
Sample_T10B_k141_132510	41658	37	2	6	Low-quality	12.67
Sample_C11C_k141_79576	5042	8	2	0	Low-quality	12.67
Sample_C1B_k141_45542	5254	5	3	0	Low-quality	12.67
Sample_C1B_k141_88351	5720	8	2	0	Low-quality	12.67
Sample_C3A_k141_143240	10176	14	6	3	Low-quality	12.67
Sample_T1C_k141_31033	8370	8	5	0	Low-quality	12.67
Sample_T2A_k141_130757	5723	7	2	1	Low-quality	12.67
Sample_T10B_k141_169188	5351	7	0	1	Low-quality	12.66
Sample_T12A_k141_65013	9449	8	0	4	Low-quality	12.66

Sample_C1A_k141_164987	5530	7	1	0	Low-quality	12.66
Sample_T12A_k141_298713	5112	7	0	1	Low-quality	12.66
Sample_T8C_k141_14481	5177	8	1	0	Low-quality	12.66
Sample_C4C_k141_177204	7804	15	1	6	Low-quality	12.65
Sample_T15C_k141_127248	17681	14	1	5	Low-quality	12.65
Sample_C10B_k141_106873	6412	10	1	0	Low-quality	12.65
Sample_T12A_k141_88338	7145	10	2	0	Low-quality	12.65
Sample_T8B_k141_90197	6008	9	4	0	Low-quality	12.65
Sample_C7A_k141_28324	6088	6	0	1	Low-quality	12.64
Sample_C8B_k141_67143	19637	22	1	9	Low-quality	12.64
Sample_T10C_k141_24089	9086	12	0	10	Low-quality	12.64
Sample_C11B_k141_143440	6433	7	5	0	Low-quality	12.64
Sample_C3A_k141_193592	6262	12	8	0	Low-quality	12.64
Sample_T6B_k141_3242	6919	8	2	0	Low-quality	12.64
Sample_T13B_k141_118425	5296	5	0	1	Low-quality	12.63
Sample_T18B_k141_136712	5868	8	5	0	Low-quality	12.63
Sample_T17B_k141_28789	5302	6	0	3	Low-quality	12.62
Sample_T18C_k141_44941	6204	8	0	2	Low-quality	12.62
Sample_T2A_k141_81441	6105	17	2	0	Low-quality	12.62
Sample_C12B_k141_141650	5145	13	1	0	Low-quality	12.62
Sample_T15C_k141_119783	17805	23	2	1	Low-quality	12.62
Sample_T4C_k141_100027	5295	13	0	0	Low-quality	12.62
Sample_T2C_k141_129217	7279	15	1	0	Low-quality	12.61
Sample_T16A_k141_122399	11338	11	1	1	Low-quality	12.6
Sample_C12B_k141_35526	5230	8	1	0	Low-quality	12.6
Sample_C6B_k141_155612	5739	7	5	0	Low-quality	12.6
Sample_T16C_k141_99945	5220	8	5	0	Low-quality	12.6
Sample_T2B_k141_306361	5419	12	5	0	Low-quality	12.6
Sample_T3A_k141_41153	13866	11	0	7	Low-quality	12.59
Sample_T7A_k141_273	7580	9	0	1	Low-quality	12.59
Sample_T7C_k141_56903	10286	8	0	0	Low-quality	12.59
Sample_T12B_k141_21695	5662	7	2	0	Low-quality	12.59
Sample_C2C_k141_303064	10213	11	1	1	Low-quality	12.58
Sample_T15B_k141_66300	20665	24	1	13	Low-quality	12.58
Sample_T3C_k141_6925	8450	10	1	1	Low-quality	12.58
Sample_T8B_k141_155389	13819	17	1	5	Low-quality	12.58
Sample_C2B_k141_228326	5520	10	2	0	Low-quality	12.58
Sample_T1B_k141_5100_fragment_1	5719	8	1	0	Low-quality	12.58
Sample_C10C_k141_40157	5071	9	0	2	Low-quality	12.57

Sample_C2A_k141_132276	19525	22	1	11	Low-quality	12.57
Sample_T14A_k141_81721	25082	14	1	10	Low-quality	12.57
Sample_C7C_k141_13886	22319	32	0	20	Low-quality	12.56
Sample_C14C_k141_24465	6273	8	0	1	Low-quality	12.56
Sample_T12B_k141_31752	7383	9	0	0	Low-quality	12.55
Sample_T4C_k141_114567	5274	5	0	5	Low-quality	12.55
Sample_C8C_k141_153704	8200	5	1	1	Low-quality	12.55
Sample_T13C_k141_138429	7506	8	0	2	Low-quality	12.54
Sample_C5A_k141_77775	5686	10	3	0	Low-quality	12.54
Sample_T15B_k141_45724_fragment_1	5478	8	1	0	Low-quality	12.54
Sample_C5A_k141_214533	5612	6	3	1	Low-quality	12.53
Sample_T2B_k141_233582	5371	11	5	0	Low-quality	12.53
Sample_C13B_k141_81884	9100	8	1	1	Low-quality	12.52
Sample_C11B_k141_109668	5773	7	6	0	Low-quality	12.52
Sample_C2B_k141_257001	5744	7	1	0	Low-quality	12.52
Sample_C4B_k141_183186	6980	10	3	0	Low-quality	12.52
Sample_C4B_k141_248176	38370	53	3	6	Low-quality	12.52
Sample_T6C_k141_135132	11260	10	0	1	Low-quality	12.51
Sample_C12B_k141_118977	5281	6	1	0	Low-quality	12.51
Sample_T2A_k141_89190	6412	15	2	1	Low-quality	12.51
Sample_T4C_k141_89857	5221	11	2	0	Low-quality	12.51
Sample_C8C_k141_91467	5207	4	0	0	Low-quality	12.49
Sample_C13C_k141_57259_fragment_1	15560	19	1	4	Low-quality	12.49
Sample_T15B_k141_135722	7758	10	3	1	Low-quality	12.49
Sample_T1B_k141_18138	17453	32	1	1	Low-quality	12.49
Sample_C10A_k141_153533	25074	26	0	12	Low-quality	12.48
Sample_C5C_k141_27393	6748	9	0	0	Low-quality	12.48
Sample_T15C_k141_79589	7921	11	3	0	Low-quality	12.48
Sample_T6A_k141_309979	13886	16	1	0	Low-quality	12.48
Sample_C9A_k141_38389	17343	19	3	4	Low-quality	12.48
Sample_T2C_k141_190808	5113	6	1	0	Low-quality	12.48
Sample_T8A_k141_255203	7639	10	1	0	Low-quality	12.48
Sample_T2C_k141_142551	6939	7	0	2	Low-quality	12.47
Sample_C11C_k141_140792	9757	10	5	0	Low-quality	12.47
Sample_C2B_k141_271141	5188	7	2	0	Low-quality	12.47
Sample_C3A_k141_80109	10267	10	0	0	Low-quality	12.47
Sample_T4B_k141_243337	7642	5	3	0	Low-quality	12.47
Sample_C4B_k141_90130	7877	8	0	4	Low-quality	12.46
Sample_C1A_k141_144556	6685	11	2	0	Low-quality	12.46

Sample_C11B_k141_49977	8925	11	1	1	Low-quality	12.45
Sample_T8A_k141_241831	21925	17	1	12	Low-quality	12.45
Sample_C9A_k141_93237	5200	6	5	0	Low-quality	12.45
Sample_T11B_k141_61085	5313	8	1	0	Low-quality	12.45
Sample_T2A_k141_84555	11092	7	0	0	Low-quality	12.45
Sample_T4B_k141_6326	7165	11	7	0	Low-quality	12.45
Sample_C13B_k141_85274	5418	8	0	0	Low-quality	12.44
Sample_C7A_k141_121484	7504	17	0	0	Low-quality	12.44
Sample_T12C_k141_137898	13421	15	0	2	Low-quality	12.44
Sample_C2A_k141_307785	13383	18	2	0	Low-quality	12.44
Sample_C7C_k141_74241	14684	22	4	2	Low-quality	12.44
Sample_T11A_k141_37008_fragment_1	10167	10	0	2	Low-quality	12.44
Sample_T5C_k141_76235	5491	7	1	0	Low-quality	12.44
Sample_C6B_k141_72428	7615	8	0	1	Low-quality	12.43
Sample_T6A_k141_36218	24685	32	1	2	Low-quality	12.43
Sample_T7C_k141_3459_fragment_1	6932	9	2	1	Low-quality	12.43
Sample_T7C_k141_40024	5589	8	2	0	Low-quality	12.43
Sample_C2B_k141_102662	7664	8	1	5	Low-quality	12.42
Sample_T10A_k141_27259	8800	6	1	0	Low-quality	12.42
Sample_T10B_k141_25616	8463	19	1	0	Low-quality	12.42
Sample_T4B_k141_271246	19959	31	0	5	Low-quality	12.42
Sample_C4C_k141_71407	5053	5	0	0	Low-quality	12.42
Sample_T15C_k141_50739	28980	35	2	2	Low-quality	12.42
Sample_C11B_k141_127403	5374	4	0	1	Low-quality	12.41
Sample_C13C_k141_61341	5937	6	3	2	Low-quality	12.41
Sample_C2A_k141_178508	5281	11	1	4	Low-quality	12.41
Sample_T17B_k141_22415	7428	8	0	2	Low-quality	12.41
Sample_T8A_k141_27953	5699	6	0	1	Low-quality	12.41
Sample_T16A_k141_90898	5417	7	7	0	Low-quality	12.41
Sample_T5A_k141_5424	8694	8	0	3	Low-quality	12.4
Sample_C10A_k141_110413	5475	12	2	0	Low-quality	12.4
Sample_T17C_k141_27921_fragment_2	7358	10	3	0	Low-quality	12.4
Sample_T7B_k141_3587	9775	27	2	0	Low-quality	12.4
Sample_C6B_k141_56424	5953	7	0	1	Low-quality	12.39
Sample_T12A_k141_175927	5604	6	2	0	Low-quality	12.39
Sample_T12A_k141_90913	6448	13	3	1	Low-quality	12.39
Sample_T5C_k141_153859	12793	6	0	0	Low-quality	12.39
Sample_C10A_k141_206374	6893	8	0	7	Low-quality	12.38
Sample_T11A_k141_124414	9084	8	0	4	Low-quality	12.38

Sample_T12A_k141_109192	5055	12	1	0	Low-quality	12.38
Sample_T12C_k141_184228	30068	39	1	5	Low-quality	12.38
Sample_T17B_k141_600	10802	16	0	7	Low-quality	12.38
Sample_C9C_k141_17925_fragment_1	5502	9	0	2	Low-quality	12.38
Sample_C2A_k141_205035	5314	2	0	1	Low-quality	12.37
Sample_C5A_k141_223309	6230	3	0	1	Low-quality	12.37
Sample_C6A_k141_45091	44589	55	2	3	Low-quality	12.37
Sample_C6B_k141_211963	5645	7	2	0	Low-quality	12.37
Sample_T10B_k141_18856	19604	19	1	16	Low-quality	12.37
Sample_T14A_k141_66462	9396	9	0	1	Low-quality	12.37
Sample_C11C_k141_169291	5645	8	4	0	Low-quality	12.37
Sample_T8C_k141_135103	5413	10	9	0	Low-quality	12.37
Sample_C11C_k141_58185	5574	5	0	0	Low-quality	12.36
Sample_T5B_k141_23021	5760	5	0	1	Low-quality	12.36
Sample_T1C_k141_51844	15559	17	1	0	Low-quality	12.36
Sample_T3B_k141_147585	5868	5	3	0	Low-quality	12.36
Sample_C2C_k141_33439	13679	15	1	5	Low-quality	12.35
Sample_T14B_k141_139211	19877	17	1	3	Low-quality	12.35
Sample_T5C_k141_8112	12878	10	0	4	Low-quality	12.35
Sample_C12A_k141_171351	7489	16	6	0	Low-quality	12.35
Sample_C14A_k141_205824	5066	7	3	0	Low-quality	12.35
Sample_C6B_k141_163045	7621	7	1	0	Low-quality	12.35
Sample_T16C_k141_134263	6726	10	3	2	Low-quality	12.35
Sample_T5B_k141_53535	5727	6	4	0	Low-quality	12.35
Sample_C3B_k141_114697	7047	2	1	0	Low-quality	12.34
Sample_C4A_k141_22686	5212	8	1	0	Low-quality	12.34
Sample_T7B_k141_123121	5543	8	2	0	Low-quality	12.34
Sample_C4A_k141_228577	5164	5	3	0	Low-quality	12.33
Sample_C12A_k141_96657	22791	20	1	3	Low-quality	12.32
Sample_C1B_k141_94806	8019	12	0	5	Low-quality	12.32
Sample_T8B_k141_146842	7563	12	1	1	Low-quality	12.32
Sample_C5A_k141_61982	29915	37	1	1	Low-quality	12.32
Sample_C9A_k141_89568	5388	8	0	2	Low-quality	12.32
Sample_T15B_k141_116912	5498	6	0	2	Low-quality	12.32
Sample_C5A_k141_192815	26527	36	14	0	Low-quality	12.31
Sample_T14A_k141_32584	18255	27	4	1	Low-quality	12.31
Sample_T15A_k141_159601	7226	10	4	1	Low-quality	12.31
Sample_T3B_k141_40896	15281	19	1	0	Low-quality	12.31
Sample_C10A_k141_334639	37758	34	0	23	Low-quality	12.3

Sample_C3A_k141_78687	13276	15	0	3	Low-quality	12.3
Sample_T18A_k141_51492	7364	14	0	4	Low-quality	12.3
Sample_C3A_k141_97307	13402	12	3	0	Low-quality	12.3
Sample_T10A_k141_7943	11505	17	2	1	Low-quality	12.3
Sample_T11C_k141_23506	5159	6	2	0	Low-quality	12.3
Sample_T14B_k141_134546_fragment_1	6985	8	0	1	Low-quality	12.3
Sample_T2A_k141_55903	9528	21	4	2	Low-quality	12.3
Sample_C11B_k141_223041	21228	18	1	11	Low-quality	12.29
Sample_C1C_k141_30901	8217	11	1	1	Low-quality	12.29
Sample_T12A_k141_130686	5161	9	3	0	Low-quality	12.29
Sample_T13A_k141_50414	7264	15	5	0	Low-quality	12.29
Sample_T2A_k141_278454	5042	6	2	0	Low-quality	12.29
Sample_C10A_k141_265356	6380	8	6	0	Low-quality	12.28
Sample_C9A_k141_133319	7735	8	0	2	Low-quality	12.28
Sample_C6B_k141_51447	9911	12	1	3	Low-quality	12.27
Sample_T15C_k141_50365	10031	8	0	6	Low-quality	12.27
Sample_C1C_k141_152034	5123	11	2	0	Low-quality	12.27
Sample_C7A_k141_30262	7237	7	0	1	Low-quality	12.27
Sample_T11B_k141_68357_fragment_1	43079	43	1	4	Low-quality	12.26
Sample_C6C_k141_79306	9783	3	2	0	Low-quality	12.25
Sample_T16B_k141_121107	5148	6	0	5	Low-quality	12.25
Sample_T3C_k141_94824	8713	12	1	4	Low-quality	12.25
Sample_T18C_k141_16596_fragment_1	11108	10	1	3	Low-quality	12.25
Sample_C10A_k141_162301	24353	26	1	20	Low-quality	12.24
Sample_C2B_k141_315419	6585	6	0	1	Low-quality	12.24
Sample_C5A_k141_59540	5144	5	0	4	Low-quality	12.24
Sample_C8C_k141_24092	21374	20	0	14	Low-quality	12.24
Sample_C9A_k141_5391	8973	5	1	0	Low-quality	12.24
Sample_T10A_k141_82251	7327	11	1	0	Low-quality	12.24
Sample_T12A_k141_164191	7908	8	0	0	Low-quality	12.24
Sample_C6C_k141_208142	5524	8	3	0	Low-quality	12.24
Sample_C9B_k141_89678	5345	6	0	0	Low-quality	12.24
Sample_T2A_k141_16276	6854	8	4	0	Low-quality	12.24
Sample_T2C_k141_27905	7274	7	1	0	Low-quality	12.24
Sample_C11A_k141_66302	50805	49	2	19	Low-quality	12.23
Sample_C1B_k141_86156	5618	3	0	0	Low-quality	12.23
Sample_C1C_k141_39314_fragment_1	10069	14	0	0	Low-quality	12.23
Sample_T12B_k141_156422	6535	7	0	3	Low-quality	12.22
Sample_T2B_k141_230555	9371	6	0	2	Low-quality	12.22

Sample_C2A_k141_188957	5147	7	0	2	Low-quality	12.22
Sample_T10B_k141_119983	16608	36	2	2	Low-quality	12.22
Sample_T11B_k141_9695	11546	12	1	0	Low-quality	12.22
Sample_T2B_k141_272267_fragment_1	5226	8	1	0	Low-quality	12.22
Sample_C12B_k141_141807	41284	40	0	24	Low-quality	12.21
Sample_T11B_k141_127548	13030	10	0	3	Low-quality	12.21
Sample_T14A_k141_55797	17830	18	1	9	Low-quality	12.21
Sample_C1A_k141_245143	21193	22	9	1	Low-quality	12.21
Sample_C4A_k141_158185	5019	8	6	0	Low-quality	12.21
Sample_C5A_k141_77752	5559	8	2	0	Low-quality	12.21
Sample_T6B_k141_48630	9648	17	3	3	Low-quality	12.19
Sample_C11C_k141_143327	6578	10	1	0	Low-quality	12.19
Sample_C4A_k141_5937	6974	6	0	0	Low-quality	12.18
Sample_C4B_k141_121884	7896	7	0	3	Low-quality	12.18
Sample_T7C_k141_17886	7705	12	0	1	Low-quality	12.18
Sample_C10A_k141_109817	5559	7	3	0	Low-quality	12.18
Sample_C1A_k141_53342	7888	3	0	0	Low-quality	12.17
Sample_C4B_k141_137174	5421	11	2	0	Low-quality	12.17
Sample_T12C_k141_126605	5993	11	2	0	Low-quality	12.17
Sample_C10A_k141_183710	18496	16	2	2	Low-quality	12.16
Sample_T10A_k141_3719	5936	6	0	6	Low-quality	12.16
Sample_C11A_k141_183091	6810	10	4	0	Low-quality	12.16
Sample_C4A_k141_239466	12917	20	2	0	Low-quality	12.16
Sample_T15A_k141_162049	7136	9	1	0	Low-quality	12.16
Sample_C3C_k141_265122	7637	8	0	4	Low-quality	12.15
Sample_T2C_k141_20339	9524	9	0	5	Low-quality	12.15
Sample_C10A_k141_46134	5349	8	0	1	Low-quality	12.15
Sample_T10C_k141_46482	5252	16	2	0	Low-quality	12.15
Sample_T12B_k141_3477	5355	10	3	0	Low-quality	12.15
Sample_C10A_k141_77249	18726	23	0	8	Low-quality	12.14
Sample_C14A_k141_47861	25499	26	0	18	Low-quality	12.14
Sample_C6B_k141_122143	43322	50	2	9	Low-quality	12.14
Sample_T12A_k141_185194	76453	88	6	38	Low-quality	12.14
Sample_T11B_k141_159376	7742	13	3	0	Low-quality	12.14
Sample_C8C_k141_91180	34055	32	1	8	Low-quality	12.13
Sample_C11C_k141_98252	7200	10	3	0	Low-quality	12.13
Sample_C8B_k141_188247_fragment_1	7672	13	1	1	Low-quality	12.13
Sample_T13B_k141_10213	6172	8	6	0	Low-quality	12.13
Sample_T2A_k141_134447	6750	10	8	1	Low-quality	12.13

Sample_T6A_k141_303319	5292	7	2	0	Low-quality	12.13
Sample_T7C_k141_2997	5505	16	3	0	Low-quality	12.13
Sample_C13C_k141_59163	5092	4	0	4	Low-quality	12.12
Sample_C10B_k141_69286	5617	7	5	0	Low-quality	12.12
Sample_C3C_k141_264409	6983	12	4	0	Low-quality	12.12
Sample_C12C_k141_12140	8006	11	1	1	Low-quality	12.11
Sample_C1C_k141_133627	19897	22	1	3	Low-quality	12.11
Sample_T11C_k141_29118	7248	11	0	3	Low-quality	12.11
Sample_T17A_k141_12589	5404	6	5	0	Low-quality	12.11
Sample_C5A_k141_31734	6349	7	0	1	Low-quality	12.1
Sample_T17A_k141_12724	7130	14	3	0	Low-quality	12.1
Sample_T2C_k141_37038	6105	6	0	2	Low-quality	12.1
Sample_C2A_k141_50751	6061	10	0	1	Low-quality	12.09
Sample_C6C_k141_116139	18681	17	1	14	Low-quality	12.09
Sample_C3B_k141_185869	6961	13	1	1	Low-quality	12.08
Sample_T17A_k141_22422_fragment_1	7343	13	8	0	Low-quality	12.07
Sample_C3B_k141_273161	5147	3	0	1	Low-quality	12.06
Sample_C5A_k141_30031	10747	11	1	0	Low-quality	12.06
Sample_T12C_k141_152988	5551	11	4	0	Low-quality	12.06
Sample_T13A_k141_131495	8664	9	0	0	Low-quality	12.06
Sample_T8C_k141_134738	9737	12	1	3	Low-quality	12.05
Sample_T11A_k141_50701	19519	22	1	5	Low-quality	12.04
Sample_T15A_k141_53733	5928	8	0	2	Low-quality	12.04
Sample_T15C_k141_124465	7274	8	0	1	Low-quality	12.04
Sample_C1B_k141_97636_fragment_1	5248	10	1	0	Low-quality	12.04
Sample_C4A_k141_242623	7803	9	0	1	Low-quality	12.04
Sample_T10B_k141_73985	5368	6	4	0	Low-quality	12.04
Sample_T15B_k141_67843	7713	8	1	0	Low-quality	12.04
Sample_T3A_k141_88677	8197	10	3	0	Low-quality	12.04
Sample_C5A_k141_247847	5868	7	1	0	Low-quality	12.03
Sample_T3A_k141_111513	7205	9	0	4	Low-quality	12.03
Sample_C4B_k141_175250	5314	10	5	0	Low-quality	12.03
Sample_T15A_k141_13911	5870	9	0	0	Low-quality	12.03
Sample_T17A_k141_19270	5826	10	1	0	Low-quality	12.03
Sample_C8B_k141_36057	7344	11	8	0	Low-quality	12.01
Sample_T16B_k141_122879	7296	11	4	1	Low-quality	12.01
Sample_T8C_k141_205693	5443	10	7	0	Low-quality	12.01
Sample_C2C_k141_214396	11385	7	0	1	Low-quality	12
Sample_T8B_k141_52433	16735	20	2	4	Low-quality	12

Sample_C13C_k141_70765	6630	7	0	1	Low-quality	12
Sample_C6B_k141_140136	24634	31	3	1	Low-quality	12
Sample_T12C_k141_179315	7663	10	0	0	Low-quality	12
Sample_T13A_k141_84227	5672	11	9	0	Low-quality	12
Sample_T16C_k141_95211	5082	6	3	0	Low-quality	12
Sample_T6C_k141_51445	5104	7	0	1	Low-quality	12
Sample_C3B_k141_147786	22108	29	1	4	Low-quality	11.99
Sample_T12A_k141_15329	5574	4	0	2	Low-quality	11.99
Sample_C13A_k141_93849	5482	8	6	0	Low-quality	11.99
Sample_C13A_k141_18027	9683	15	1	3	Low-quality	11.98
Sample_C11A_k141_32717	10999	12	7	2	Low-quality	11.98
Sample_C4C_k141_201286	14932	10	1	2	Low-quality	11.98
Sample_T14C_k141_156172	7920	5	1	0	Low-quality	11.98
Sample_T18A_k141_198084	5590	6	1	1	Low-quality	11.98
Sample_C13B_k141_28436	7168	7	0	4	Low-quality	11.97
Sample_C3A_k141_156369	5081	6	0	1	Low-quality	11.97
Sample_C3A_k141_237820	10787	11	5	0	Low-quality	11.97
Sample_C4B_k141_204748	5951	5	0	1	Low-quality	11.97
Sample_C12C_k141_197834	7905	13	1	2	Low-quality	11.96
Sample_T8B_k141_20554	20875	18	0	14	Low-quality	11.96
Sample_C11B_k141_104504	5078	10	8	0	Low-quality	11.96
Sample_C3C_k141_113427	6934	11	1	0	Low-quality	11.96
Sample_T4B_k141_191944_fragment_1	12356	17	3	2	Low-quality	11.96
Sample_C10C_k141_210406	13426	16	0	5	Low-quality	11.95
Sample_C1C_k141_82711	5021	4	0	4	Low-quality	11.95
Sample_T17B_k141_1245	7153	10	0	1	Low-quality	11.95
Sample_T4B_k141_236869	20830	15	1	1	Low-quality	11.95
Sample_T5A_k141_3539	11232	13	1	1	Low-quality	11.95
Sample_C1A_k141_6776	7548	7	0	1	Low-quality	11.94
Sample_C4B_k141_81612	19998	20	0	9	Low-quality	11.94
Sample_C5A_k141_49848	5480	7	0	1	Low-quality	11.94
Sample_C11C_k141_162787	5517	11	1	0	Low-quality	11.94
Sample_C5B_k141_55648	8458	12	0	1	Low-quality	11.94
Sample_T2B_k141_197394	5780	9	4	0	Low-quality	11.94
Sample_C12C_k141_59068	6448	8	1	0	Low-quality	11.93
Sample_C7A_k141_57208	5506	5	0	0	Low-quality	11.93
Sample_C11C_k141_6169	5372	11	1	0	Low-quality	11.93
Sample_C3B_k141_57327	5604	7	0	1	Low-quality	11.93
Sample_C7C_k141_51554	10726	10	0	1	Low-quality	11.92

Sample_T11A_k141_43416	17736	20	1	3	Low-quality	11.92
Sample_C10C_k141_9063	7566	13	2	1	Low-quality	11.92
Sample_C3A_k141_250775	10045	17	3	0	Low-quality	11.92
Sample_C14C_k141_103883	5273	6	1	0	Low-quality	11.91
Sample_T13A_k141_115737	5676	12	9	0	Low-quality	11.91
Sample_T7B_k141_77613_fragment_1	8068	8	0	1	Low-quality	11.91
Sample_C12A_k141_171773	6238	9	1	1	Low-quality	11.89
Sample_C3B_k141_150069	36089	27	0	18	Low-quality	11.89
Sample_T18C_k141_38532_fragment_1	8104	9	0	1	Low-quality	11.89
Sample_T4B_k141_234978	6718	15	3	0	Low-quality	11.89
Sample_C11A_k141_114003	6092	7	0	4	Low-quality	11.88
Sample_C6B_k141_191115	5796	5	0	4	Low-quality	11.88
Sample_C11A_k141_155839	6651	8	2	0	Low-quality	11.88
Sample_C2C_k141_12579	8310	10	2	0	Low-quality	11.88
Sample_C3A_k141_54248	5619	6	1	0	Low-quality	11.88
Sample_C4C_k141_40682	19162	23	12	0	Low-quality	11.88
Sample_C12A_k141_149495	10906	22	0	1	Low-quality	11.87
Sample_T6C_k141_186867	6078	7	3	0	Low-quality	11.87
Sample_C12C_k141_59458	17632	26	1	3	Low-quality	11.85
Sample_T12C_k141_119959	7646	6	0	2	Low-quality	11.85
Sample_T4B_k141_87691	9681	10	0	3	Low-quality	11.85
Sample_T7C_k141_84906	7093	11	0	2	Low-quality	11.85
Sample_C1A_k141_193842	8725	11	3	0	Low-quality	11.85
Sample_C3A_k141_31277	7447	7	6	0	Low-quality	11.85
Sample_T10B_k141_44113	5791	10	2	0	Low-quality	11.85
Sample_T6C_k141_34363_fragment_2	17140	21	2	2	Low-quality	11.85
Sample_C9C_k141_24927	9514	7	2	2	Low-quality	11.84
Sample_T18B_k141_50561	5951	7	3	1	Low-quality	11.84
Sample_C9A_k141_22464	14402	12	2	5	Low-quality	11.83
Sample_T3A_k141_32599	7080	6	0	5	Low-quality	11.83
Sample_C14A_k141_187847	5573	12	2	0	Low-quality	11.83
Sample_C3A_k141_152489	5414	6	4	0	Low-quality	11.83
Sample_C3A_k141_238421	5340	6	3	0	Low-quality	11.83
Sample_C6C_k141_136035	7125	9	7	0	Low-quality	11.83
Sample_C7A_k141_177374	10649	9	0	1	Low-quality	11.83
Sample_T13A_k141_104036	15815	36	3	2	Low-quality	11.83
Sample_T13B_k141_18544	7531	8	1	1	Low-quality	11.83
Sample_C5A_k141_98785	35953	34	0	22	Low-quality	11.82
Sample_C7B_k141_27024	12435	17	2	8	Low-quality	11.82

Sample_T3A_k141_62367	7707	5	0	1	Low-quality	11.82
Sample_C1B_k141_8402_fragment_2	20085	30	4	3	Low-quality	11.82
Sample_C3A_k141_123722	17600	25	3	3	Low-quality	11.82
Sample_T8B_k141_154582	5058	6	0	0	Low-quality	11.82
Sample_T17C_k141_32490	13988	20	0	4	Low-quality	11.81
Sample_C2B_k141_238252	6307	13	0	1	Low-quality	11.81
Sample_C12A_k141_96109	7067	12	0	1	Low-quality	11.8
Sample_C6B_k141_14339	17351	27	1	1	Low-quality	11.8
Sample_T13B_k141_45587	9641	9	0	2	Low-quality	11.8
Sample_T15A_k141_73342	7696	14	1	1	Low-quality	11.8
Sample_T4B_k141_143783	6189	10	3	1	Low-quality	11.8
Sample_T6A_k141_147688	5927	5	0	0	Low-quality	11.8
Sample_T13B_k141_15088	5591	5	0	0	Low-quality	11.79
Sample_C3A_k141_39284	8039	7	4	1	Low-quality	11.79
Sample_C6C_k141_201134	5166	11	4	1	Low-quality	11.79
Sample_C8C_k141_85816	9494	12	1	0	Low-quality	11.79
Sample_T3B_k141_171993	5049	4	3	0	Low-quality	11.79
Sample_T17B_k141_10189	12232	7	3	0	Low-quality	11.78
Sample_T7A_k141_59478	18803	17	1	4	Low-quality	11.77
Sample_T7B_k141_38701	5058	5	0	0	Low-quality	11.77
Sample_C14A_k141_119425	10784	15	2	1	Low-quality	11.77
Sample_T4B_k141_76905	9032	20	5	0	Low-quality	11.77
Sample_C3B_k141_256030	7149	10	1	4	Low-quality	11.76
Sample_C5A_k141_60228	12093	10	1	1	Low-quality	11.76
Sample_C6A_k141_80220	7774	7	1	0	Low-quality	11.76
Sample_C11A_k141_111687	6228	6	2	0	Low-quality	11.76
Sample_T7C_k141_141325	5917	5	3	0	Low-quality	11.76
Sample_C10B_k141_32846	11533	12	0	5	Low-quality	11.75
Sample_C12A_k141_30909	7009	3	1	0	Low-quality	11.75
Sample_C14A_k141_73128	91820	71	5	44	Low-quality	11.75
Sample_C3B_k141_104081	21655	18	1	14	Low-quality	11.75
Sample_T10C_k141_71657	9994	12	0	3	Low-quality	11.75
Sample_T3B_k141_179025	11696	9	1	1	Low-quality	11.75
Sample_T6A_k141_41966	32896	30	1	4	Low-quality	11.75
Sample_C4C_k141_18482	5623	12	1	0	Low-quality	11.75
Sample_T13B_k141_125626_fragment_2	8061	8	2	0	Low-quality	11.75
Sample_T3C_k141_78195_fragment_1	10282	8	0	2	Low-quality	11.75
Sample_C4C_k141_143829	5288	11	7	0	Low-quality	11.74
Sample_C10C_k141_227079	9590	9	0	2	Low-quality	11.73

Sample_C3A_k141_3505	6311	1	1	0	Low-quality	11.73
Sample_T12B_k141_55715	5034	5	0	1	Low-quality	11.73
Sample_T17A_k141_48237	5442	8	6	0	Low-quality	11.73
Sample_T5C_k141_26180	6338	6	1	1	Low-quality	11.73
Sample_T8B_k141_113959	6133	14	3	1	Low-quality	11.73
Sample_T8B_k141_234603	6780	11	1	0	Low-quality	11.73
Sample_C1B_k141_28658	5416	8	1	0	Low-quality	11.72
Sample_C2B_k141_185753	7306	9	0	2	Low-quality	11.72
Sample_T4A_k141_20739	6344	9	0	2	Low-quality	11.72
Sample_C2A_k141_166975	5368	9	5	0	Low-quality	11.72
Sample_C5C_k141_39087	5693	9	5	1	Low-quality	11.72
Sample_C5A_k141_67566	7704	16	0	0	Low-quality	11.71
Sample_T14B_k141_122383	9498	11	1	0	Low-quality	11.71
Sample_T13B_k141_151230_fragment_1	8622	9	1	2	Low-quality	11.71
Sample_T2B_k141_35797	6398	8	0	0	Low-quality	11.7
Sample_T3C_k141_36591	12687	17	0	2	Low-quality	11.7
Sample_C14A_k141_202321	7298	8	3	0	Low-quality	11.7
Sample_C1B_k141_49681	5022	6	0	1	Low-quality	11.7
Sample_T8C_k141_73155	6354	10	2	0	Low-quality	11.7
Sample_C3A_k141_246360	5571	12	8	0	Low-quality	11.69
Sample_T13A_k141_95843	5386	4	0	0	Low-quality	11.69
Sample_T7C_k141_41553	10359	12	1	5	Low-quality	11.68
Sample_T5C_k141_143883	10465	10	0	0	Low-quality	11.68
Sample_C13A_k141_35654	6855	14	1	3	Low-quality	11.67
Sample_T12A_k141_196859	23788	28	1	6	Low-quality	11.67
Sample_T1C_k141_35247	6808	8	1	0	Low-quality	11.67
Sample_T3A_k141_44587	6989	10	0	2	Low-quality	11.67
Sample_T7A_k141_138764	6989	10	0	0	Low-quality	11.67
Sample_C9A_k141_75609	20764	22	5	0	Low-quality	11.67
Sample_C7C_k141_29831	42542	34	1	25	Low-quality	11.66
Sample_C8C_k141_163434	10920	9	0	3	Low-quality	11.66
Sample_T8A_k141_33114	6276	5	0	2	Low-quality	11.66
Sample_C3A_k141_132942	17266	45	0	0	Low-quality	11.66
Sample_C8A_k141_56158	6828	5	0	1	Low-quality	11.65
Sample_C10A_k141_309893_fragment_1	5870	12	2	0	Low-quality	11.65
Sample_C7A_k141_128921	7579	13	3	0	Low-quality	11.65
Sample_T1B_k141_29569	7197	13	3	0	Low-quality	11.65
Sample_C7C_k141_78435	8249	10	0	5	Low-quality	11.64
Sample_T2B_k141_230729	7189	5	0	1	Low-quality	11.64

Sample_T4B_k141_255254	5808	5	0	1	Low-quality	11.64
Sample_C11A_k141_49021	9754	16	3	0	Low-quality	11.64
Sample_C13C_k141_54606	6881	14	2	0	Low-quality	11.64
Sample_T12B_k141_30885	30371	33	1	5	Low-quality	11.63
Sample_T15C_k141_20927	7194	8	0	0	Low-quality	11.63
Sample_T12C_k141_157589	5392	14	2	0	Low-quality	11.63
Sample_T12C_k141_89726	16877	19	3	0	Low-quality	11.63
Sample_T18A_k141_180511	5428	5	2	0	Low-quality	11.63
Sample_T5C_k141_95420	7046	11	2	0	Low-quality	11.63
Sample_C14A_k141_170588	8903	14	10	0	Low-quality	11.62
Sample_C8B_k141_50965	10459	7	0	0	Low-quality	11.62
Sample_C1C_k141_40792	9549	6	0	2	Low-quality	11.61
Sample_C6C_k141_24739	5989	9	1	0	Low-quality	11.61
Sample_T4B_k141_145770	21688	22	1	10	Low-quality	11.61
Sample_T5B_k141_43071	6949	12	0	4	Low-quality	11.61
Sample_T6B_k141_189373	32565	43	3	17	Low-quality	11.61
Sample_T14B_k141_117894_fragment_1	6986	8	0	1	Low-quality	11.61
Sample_T8A_k141_105161	6401	7	0	2	Low-quality	11.61
Sample_C9A_k141_36791	5610	8	2	0	Low-quality	11.6
Sample_T4A_k141_34841	5641	6	4	0	Low-quality	11.6
Sample_C11C_k141_179618	20513	28	1	9	Low-quality	11.59
Sample_C6A_k141_189899	7089	8	0	1	Low-quality	11.59
Sample_C6C_k141_81786	8266	10	0	0	Low-quality	11.59
Sample_T11B_k141_117644	5482	10	7	0	Low-quality	11.59
Sample_T16C_k141_109953	23567	15	1	1	Low-quality	11.59
Sample_T17C_k141_38985	12026	16	4	1	Low-quality	11.59
Sample_T12C_k141_2476	6685	6	0	2	Low-quality	11.58
Sample_T16C_k141_60809	21355	29	1	1	Low-quality	11.58
Sample_T3A_k141_97278	10132	9	0	2	Low-quality	11.58
Sample_C2B_k141_56850	6771	8	5	0	Low-quality	11.58
Sample_C4C_k141_100251	8437	10	4	0	Low-quality	11.58
Sample_C6B_k141_141039	5198	7	3	0	Low-quality	11.58
Sample_C6C_k141_212774	6642	12	4	0	Low-quality	11.57
Sample_T12B_k141_98349	15523	19	3	0	Low-quality	11.57
Sample_T12C_k141_103954	7745	8	0	3	Low-quality	11.56
Sample_T10B_k141_173978	7844	12	2	0	Low-quality	11.56
Sample_T12A_k141_184822	20726	20	1	0	Low-quality	11.56
Sample_T16A_k141_11448	5627	5	0	2	Low-quality	11.56
Sample_C3A_k141_173650	5518	5	0	0	Low-quality	11.55

Sample_C3C_k141_57856	8818	19	1	0	Low-quality	11.55
Sample_T12A_k141_177073	9204	14	0	0	Low-quality	11.55
Sample_T12C_k141_106911	5193	8	4	0	Low-quality	11.55
Sample_T8A_k141_210685	7205	5	1	0	Low-quality	11.55
Sample_C2C_k141_149711	12465	8	0	4	Low-quality	11.53
Sample_C6B_k141_7495	9436	6	0	3	Low-quality	11.53
Sample_T2B_k141_140760	72828	70	2	29	Low-quality	11.53
Sample_T4B_k141_6776	5179	10	1	0	Low-quality	11.53
Sample_C8C_k141_148755	15374	36	4	2	Low-quality	11.53
Sample_T5B_k141_3446	5615	11	8	0	Low-quality	11.53
Sample_C10B_k141_85008	8317	11	0	5	Low-quality	11.52
Sample_C11A_k141_141063	8709	10	1	4	Low-quality	11.52
Sample_C11B_k141_182318	9194	3	2	0	Low-quality	11.52
Sample_T18C_k141_117248	9416	8	0	2	Low-quality	11.52
Sample_C11C_k141_1970	5560	12	2	0	Low-quality	11.52
Sample_C2C_k141_18534	5291	6	2	0	Low-quality	11.52
Sample_C2C_k141_158342	5167	9	4	0	Low-quality	11.51
Sample_C5C_k141_114299	5474	6	6	0	Low-quality	11.51
Sample_T11A_k141_49138	15453	9	3	0	Low-quality	11.51
Sample_T12A_k141_190728	5195	4	2	0	Low-quality	11.51
Sample_T16C_k141_93095	6308	5	1	0	Low-quality	11.51
Sample_T4B_k141_229211	18366	23	6	0	Low-quality	11.51
Sample_C3B_k141_162059	5340	11	2	0	Low-quality	11.5
Sample_C9A_k141_118821	21196	32	1	3	Low-quality	11.5
Sample_T14A_k141_23204	7499	3	1	0	Low-quality	11.5
Sample_C5C_k141_71090_fragment_1	6014	10	1	1	Low-quality	11.5
Sample_C12A_k141_189882	5338	3	0	3	Low-quality	11.49
Sample_C6C_k141_61184	6399	7	0	6	Low-quality	11.49
Sample_T15C_k141_32383	11377	13	0	4	Low-quality	11.49
Sample_T16C_k141_71680	10338	8	0	0	Low-quality	11.49
Sample_C14C_k141_159243	32038	26	1	14	Low-quality	11.48
Sample_T14B_k141_147438	10781	11	0	1	Low-quality	11.48
Sample_C6C_k141_229527	5156	5	1	0	Low-quality	11.46
Sample_C8A_k141_171563	8806	4	0	2	Low-quality	11.46
Sample_T4B_k141_52694	43220	45	0	34	Low-quality	11.46
Sample_C3A_k141_119883	6113	11	2	0	Low-quality	11.46
Sample_C3B_k141_107806	6741	7	1	2	Low-quality	11.46
Sample_T10B_k141_129232	5157	9	3	0	Low-quality	11.46
Sample_T14A_k141_58078	12276	10	3	1	Low-quality	11.46

Sample_C3C_k141_83542	53367	28	1	20	Low-quality	11.45
Sample_T2A_k141_165820	5157	11	3	0	Low-quality	11.45
Sample_C10C_k141_80616	8340	13	0	2	Low-quality	11.44
Sample_T4B_k141_301974	6894	8	0	1	Low-quality	11.44
Sample_C10A_k141_179293	5485	7	7	0	Low-quality	11.44
Sample_C5A_k141_158628	5425	9	0	0	Low-quality	11.44
Sample_T18C_k141_21623	6270	8	0	1	Low-quality	11.44
Sample_C11A_k141_147637	7693	10	0	2	Low-quality	11.43
Sample_T2B_k141_51411	15196	16	0	6	Low-quality	11.43
Sample_T2C_k141_85224	7235	8	0	4	Low-quality	11.43
Sample_T3C_k141_37562	6389	14	1	2	Low-quality	11.43
Sample_C14A_k141_84665	5316	12	2	0	Low-quality	11.43
Sample_C4B_k141_222174	5350	7	2	0	Low-quality	11.43
Sample_C7B_k141_73824_fragment_1	7516	11	1	0	Low-quality	11.43
Sample_T12A_k141_54712	8385	10	4	0	Low-quality	11.43
Sample_T13C_k141_11992	5973	10	3	1	Low-quality	11.43
Sample_T4B_k141_314859	19536	36	3	2	Low-quality	11.43
Sample_C12C_k141_24107	8204	12	0	1	Low-quality	11.42
Sample_T16B_k141_23655	23771	25	0	15	Low-quality	11.42
Sample_C10A_k141_312173	15045	11	0	8	Low-quality	11.41
Sample_C2A_k141_34579	7334	7	0	2	Low-quality	11.41
Sample_C2B_k141_287594	62458	47	1	33	Low-quality	11.41
Sample_T5C_k141_12513	18040	13	1	5	Low-quality	11.41
Sample_C10A_k141_329324	8733	13	10	0	Low-quality	11.41
Sample_C14B_k141_21412	6503	11	7	0	Low-quality	11.41
Sample_T10A_k141_105262_fragment_1	8316	8	0	1	Low-quality	11.41
Sample_C3B_k141_180891	10472	14	2	0	Low-quality	11.4
Sample_C5A_k141_141370	5271	13	1	2	Low-quality	11.4
Sample_C2A_k141_315986	5020	5	0	1	Low-quality	11.39
Sample_C1A_k141_105984	8671	6	1	1	Low-quality	11.39
Sample_C10C_k141_169278	13880	17	1	3	Low-quality	11.38
Sample_C12C_k141_78174	11885	9	0	5	Low-quality	11.38
Sample_T12A_k141_138962	7020	14	1	6	Low-quality	11.38
Sample_T16B_k141_19258	7358	12	2	1	Low-quality	11.38
Sample_T5B_k141_66251	5586	6	3	3	Low-quality	11.38
Sample_T6C_k141_6872	6816	6	0	1	Low-quality	11.38
Sample_T7B_k141_118265	5724	5	0	1	Low-quality	11.38
Sample_C11A_k141_115965	20674	19	0	3	Low-quality	11.37
Sample_T11B_k141_5762	5333	14	1	0	Low-quality	11.37

Sample_T12B_k141_179665	9094	19	0	0	Low-quality	11.37
Sample_T15C_k141_119857	12279	13	1	3	Low-quality	11.37
Sample_C6B_k141_98118	6867	13	2	0	Low-quality	11.37
Sample_T12A_k141_135613	5053	4	4	0	Low-quality	11.37
Sample_T14B_k141_120464	5098	7	2	0	Low-quality	11.37
Sample_C6A_k141_111911	15490	18	1	5	Low-quality	11.36
Sample_C8B_k141_125232	7211	9	0	1	Low-quality	11.36
Sample_T16C_k141_76904	7509	7	1	0	Low-quality	11.36
Sample_C5A_k141_79481	20210	16	2	0	Low-quality	11.36
Sample_T3A_k141_50041	7117	13	3	1	Low-quality	11.36
Sample_C10C_k141_218852	8669	9	0	7	Low-quality	11.35
Sample_C2C_k141_103465	9426	2	0	0	Low-quality	11.35
Sample_T16B_k141_45519	20930	30	1	2	Low-quality	11.35
Sample_T2B_k141_161602	8498	4	0	1	Low-quality	11.35
Sample_T6C_k141_18307	5205	5	0	4	Low-quality	11.35
Sample_T7A_k141_14339	9343	9	0	1	Low-quality	11.35
Sample_C11A_k141_105356	7175	16	1	0	Low-quality	11.35
Sample_C13B_k141_55425	5202	9	0	0	Low-quality	11.35
Sample_C14A_k141_218970	5955	11	2	0	Low-quality	11.35
Sample_C5A_k141_61769	10608	12	2	1	Low-quality	11.35
Sample_C13A_k141_5982	7026	10	3	2	Low-quality	11.33
Sample_C11A_k141_155189_fragment_1	12964	15	2	1	Low-quality	11.33
Sample_C12B_k141_52503	10198	7	0	1	Low-quality	11.33
Sample_C14C_k141_170729	41327	36	1	29	Low-quality	11.32
Sample_C9B_k141_18171	5680	9	0	2	Low-quality	11.32
Sample_C2A_k141_309749	8459	11	1	2	Low-quality	11.32
Sample_C11A_k141_59903	6334	15	0	0	Low-quality	11.31
Sample_C2B_k141_302003	5236	6	0	1	Low-quality	11.31
Sample_T6B_k141_48207	36262	38	2	11	Low-quality	11.31
Sample_T5A_k141_7030_fragment_1	12095	18	3	0	Low-quality	11.31
Sample_C12A_k141_91532	11820	9	1	4	Low-quality	11.3
Sample_T12B_k141_106597	19182	15	1	2	Low-quality	11.3
Sample_T2B_k141_106248	7467	7	1	2	Low-quality	11.3
Sample_C4B_k141_186909	27905	33	5	0	Low-quality	11.3
Sample_C10A_k141_18377	6677	12	0	1	Low-quality	11.29
Sample_T15A_k141_37996	5788	9	0	0	Low-quality	11.29
Sample_T17A_k141_6100	17539	11	1	2	Low-quality	11.29
Sample_T14A_k141_113031_fragment_1	9519	12	1	3	Low-quality	11.29
Sample_T15A_k141_1097	6409	5	2	0	Low-quality	11.29

Sample_T2B_k141_64685	6690	8	1	0	Low-quality	11.29
Sample_T2B_k141_241067	5514	7	2	1	Low-quality	11.29
Sample_C12C_k141_154615	5034	10	5	0	Low-quality	11.28
Sample_T7A_k141_87662	9221	7	0	2	Low-quality	11.28
Sample_C13A_k141_26711	8786	20	4	0	Low-quality	11.28
Sample_C7B_k141_60729	16187	10	1	1	Low-quality	11.28
Sample_C9A_k141_68816	6261	7	0	1	Low-quality	11.28
Sample_C4A_k141_772	5490	5	0	1	Low-quality	11.27
Sample_C14A_k141_128620	6121	5	3	0	Low-quality	11.27
Sample_C14A_k141_227364	5426	8	6	0	Low-quality	11.27
Sample_C7A_k141_18527	6220	7	1	0	Low-quality	11.27
Sample_T12A_k141_24051	9854	24	1	1	Low-quality	11.27
Sample_T16C_k141_50106	7481	12	5	1	Low-quality	11.27
Sample_C10C_k141_140618	5036	8	1	0	Low-quality	11.26
Sample_C14A_k141_229234	6033	10	5	0	Low-quality	11.26
Sample_T6A_k141_308026	5654	8	0	0	Low-quality	11.26
Sample_T8B_k141_232753_fragment_1	7879	10	3	3	Low-quality	11.26
Sample_C1B_k141_144198	18485	19	1	11	Low-quality	11.25
Sample_T14B_k141_204915	11564	12	0	2	Low-quality	11.25
Sample_C2A_k141_199586	8399	9	1	1	Low-quality	11.25
Sample_T3B_k141_71080	18895	8	2	0	Low-quality	11.25
Sample_T7C_k141_9060	10376	10	0	1	Low-quality	11.25
Sample_T14B_k141_59977	7673	9	0	6	Low-quality	11.24
Sample_C3A_k141_230802	132643	132	1	72	Low-quality	11.23
Sample_C1B_k141_247155	5219	9	3	0	Low-quality	11.23
Sample_C2A_k141_160170	7841	11	5	0	Low-quality	11.23
Sample_T10C_k141_48717	7024	12	2	0	Low-quality	11.23
Sample_T8C_k141_124468	6317	6	0	0	Low-quality	11.23
Sample_C13B_k141_103000	5660	16	4	0	Low-quality	11.22
Sample_T6B_k141_41930	5534	6	0	1	Low-quality	11.22
Sample_T7A_k141_3917	11485	13	0	2	Low-quality	11.22
Sample_T8B_k141_52659	7544	10	0	1	Low-quality	11.22
Sample_C12A_k141_138608	6799	10	2	0	Low-quality	11.22
Sample_C14C_k141_33994	15646	24	3	0	Low-quality	11.22
Sample_C5A_k141_244323	5786	5	3	0	Low-quality	11.22
Sample_C4C_k141_83912	24915	28	1	3	Low-quality	11.21
Sample_T18C_k141_47171	31483	31	1	11	Low-quality	11.21
Sample_T5C_k141_182946	9159	12	0	2	Low-quality	11.21
Sample_C5A_k141_192702	11128	18	2	0	Low-quality	11.21

Sample_T4B_k141_87066	9983	9	1	1	Low-quality	11.21
Sample_T8A_k141_211994	7229	17	1	0	Low-quality	11.21
Sample_T8C_k141_187095	6770	11	3	0	Low-quality	11.21
Sample_C10C_k141_200246	9044	16	2	0	Low-quality	11.2
Sample_C9A_k141_70064	5410	8	4	0	Low-quality	11.2
Sample_T5C_k141_108995	7882	9	2	1	Low-quality	11.19
Sample_C6B_k141_31829	5433	7	1	0	Low-quality	11.19
Sample_C8A_k141_29787	5463	8	4	0	Low-quality	11.19
Sample_T4B_k141_45193	5067	6	3	0	Low-quality	11.19
Sample_T6A_k141_221271	6390	9	3	0	Low-quality	11.19
Sample_T5C_k141_60128	6759	13	5	0	Low-quality	11.18
Sample_T5B_k141_51068	6685	9	0	3	Low-quality	11.17
Sample_C11B_k141_101090	6206	7	1	0	Low-quality	11.17
Sample_C2B_k141_130990	5145	7	2	0	Low-quality	11.17
Sample_T6C_k141_64162	8192	1	0	0	Low-quality	11.16
Sample_C5A_k141_36130	5296	11	4	0	Low-quality	11.16
Sample_T11B_k141_31083	6239	15	1	0	Low-quality	11.16
Sample_T5C_k141_194944	6594	14	2	1	Low-quality	11.15
Sample_C13B_k141_12684	20681	22	0	22	Low-quality	11.14
Sample_C8B_k141_10298	6419	9	3	0	Low-quality	11.14
Sample_C2C_k141_312187	5944	9	4	0	Low-quality	11.14
Sample_C7B_k141_110433	6891	12	6	0	Low-quality	11.14
Sample_C8C_k141_113554	6092	6	0	1	Low-quality	11.14
Sample_T10B_k141_67047_fragment_3	27141	36	3	3	Low-quality	11.14
Sample_C7B_k141_43547	12528	15	1	3	Low-quality	11.13
Sample_T18B_k141_16433	18290	21	1	5	Low-quality	11.13
Sample_C3A_k141_211683	13871	17	1	0	Low-quality	11.13
Sample_C8A_k141_25654	5180	10	4	0	Low-quality	11.13
Sample_C9B_k141_90199_fragment_2	11963	11	0	1	Low-quality	11.13
Sample_T16B_k141_77013	5109	6	1	0	Low-quality	11.13
Sample_T6A_k141_4112	8647	18	6	0	Low-quality	11.13
Sample_T7A_k141_204613	12260	15	0	0	Low-quality	11.13
Sample_C3C_k141_168097	6421	17	1	0	Low-quality	11.12
Sample_T12B_k141_138427	6036	11	4	0	Low-quality	11.12
Sample_T8C_k141_98104	5070	8	5	0	Low-quality	11.12
Sample_T6B_k141_8859	17259	24	1	14	Low-quality	11.11
Sample_C14C_k141_8867	6831	6	0	3	Low-quality	11.11
Sample_T7C_k141_66668	8962	17	3	2	Low-quality	11.11
Sample_T14B_k141_122305	8174	6	0	3	Low-quality	11.1

Sample_C10A_k141_235579	5035	6	1	0	Low-quality	11.1
Sample_C12C_k141_55236	7338	9	1	0	Low-quality	11.1
Sample_C1B_k141_150416	5917	8	1	1	Low-quality	11.09
Sample_C4B_k141_150107	37538	28	0	15	Low-quality	11.09
Sample_T3B_k141_113862	6091	13	0	1	Low-quality	11.09
Sample_T18C_k141_108503	7328	9	1	0	Low-quality	11.09
Sample_T5C_k141_127285	6988	8	0	1	Low-quality	11.09
Sample_C10A_k141_190894	6104	10	2	0	Low-quality	11.08
Sample_T3B_k141_151271	17665	14	1	5	Low-quality	11.08
Sample_T14A_k141_42127	5650	10	4	0	Low-quality	11.08
Sample_T17A_k141_19747_fragment_2	5951	9	1	2	Low-quality	11.08
Sample_C11C_k141_138272	7689	11	0	1	Low-quality	11.07
Sample_C12B_k141_83772	9796	13	0	4	Low-quality	11.07
Sample_C4B_k141_186451	17110	18	1	11	Low-quality	11.07
Sample_T17C_k141_18942	6720	18	1	0	Low-quality	11.07
Sample_T7A_k141_197405	5796	7	1	1	Low-quality	11.07
Sample_T10B_k141_79810	10462	7	0	2	Low-quality	11.06
Sample_T6B_k141_159385	19504	25	1	10	Low-quality	11.06
Sample_T8A_k141_43194	6252	7	1	0	Low-quality	11.06
Sample_C11B_k141_128677	15207	13	0	8	Low-quality	11.05
Sample_C6C_k141_36710	6153	10	0	3	Low-quality	11.05
Sample_C9C_k141_67624	5091	7	0	0	Low-quality	11.05
Sample_T13A_k141_106582	5303	12	8	0	Low-quality	11.05
Sample_T15C_k141_63056	5534	6	0	0	Low-quality	11.05
Sample_T4B_k141_42854	20802	25	1	3	Low-quality	11.05
Sample_C10C_k141_70638	38792	35	1	21	Low-quality	11.04
Sample_C2C_k141_10710	6363	8	0	1	Low-quality	11.04
Sample_T15A_k141_72351	8788	5	1	0	Low-quality	11.04
Sample_C7A_k141_50669	5126	5	0	2	Low-quality	11.03
Sample_T14C_k141_83397	9426	12	0	6	Low-quality	11.03
Sample_T18B_k141_113074	7061	7	0	0	Low-quality	11.03
Sample_C4A_k141_123854	6385	10	4	0	Low-quality	11.03
Sample_C7B_k141_92720_fragment_1	9592	12	1	2	Low-quality	11.03
Sample_C1A_k141_231277	12101	7	1	4	Low-quality	11.02
Sample_T15B_k141_117522	6907	13	2	0	Low-quality	11.02
Sample_T3A_k141_88400	6231	6	1	1	Low-quality	11.02
Sample_C3C_k141_3884	7820	13	2	0	Low-quality	11.01
Sample_C1C_k141_59463	15109	14	2	6	Low-quality	11
Sample_C6C_k141_6506	24929	29	1	23	Low-quality	11

Sample_C12B_k141_68619	7735	11	0	0	Low-quality	11
Sample_C13C_k141_95394	6703	9	5	0	Low-quality	11
Sample_C1A_k141_263162	8459	14	1	0	Low-quality	11
Sample_C2A_k141_119701	5232	11	3	0	Low-quality	11
Sample_T1A_k141_46527	6775	9	9	0	Low-quality	11
Sample_T14A_k141_214212	5111	6	0	1	Low-quality	10.99
Sample_T2B_k141_109166	10811	5	0	1	Low-quality	10.99
Sample_C4C_k141_50937	22402	22	0	14	Low-quality	10.98
Sample_T4B_k141_105711	9706	4	0	4	Low-quality	10.98
Sample_C1B_k141_109345	12892	12	4	0	Low-quality	10.98
Sample_C3C_k141_3367	5402	6	0	0	Low-quality	10.98
Sample_T17B_k141_8485	7257	7	1	1	Low-quality	10.98
Sample_T7A_k141_102203	6622	8	5	0	Low-quality	10.98
Sample_C10A_k141_218671	7052	10	4	0	Low-quality	10.97
Sample_C4C_k141_4345	16801	20	0	10	Low-quality	10.97
Sample_C2B_k141_260344	5424	6	3	0	Low-quality	10.97
Sample_T14A_k141_60726	19416	25	8	1	Low-quality	10.97
Sample_T7B_k141_109701	5517	6	1	0	Low-quality	10.97
Sample_C6B_k141_217612	18741	20	1	12	Low-quality	10.96
Sample_T8A_k141_241781	10721	11	0	3	Low-quality	10.96
Sample_C9B_k141_54692	6319	10	5	1	Low-quality	10.96
Sample_T4C_k141_60573	8594	7	1	4	Low-quality	10.95
Sample_T6B_k141_63119	19789	27	1	1	Low-quality	10.95
Sample_C14A_k141_197335	6196	4	0	0	Low-quality	10.95
Sample_C3A_k141_213119	6638	8	7	0	Low-quality	10.95
Sample_C9A_k141_70860_fragment_1	5978	10	1	1	Low-quality	10.95
Sample_C8B_k141_126564	174385	173	3	130	Low-quality	10.94
Sample_T2C_k141_215745	5084	6	0	0	Low-quality	10.94
Sample_C8A_k141_80244	5719	12	7	1	Low-quality	10.94
Sample_T12B_k141_135477	6103	6	0	1	Low-quality	10.94
Sample_T12C_k141_149406	5511	7	0	0	Low-quality	10.94
Sample_T15A_k141_56409	5178	8	3	0	Low-quality	10.94
Sample_T15B_k141_16310_fragment_1	16898	17	1	2	Low-quality	10.94
Sample_T18C_k141_80709	9010	12	0	1	Low-quality	10.94
Sample_T8A_k141_109452	6103	10	0	0	Low-quality	10.93
Sample_C1A_k141_160808	6818	9	1	0	Low-quality	10.93
Sample_C6A_k141_36374	7214	10	1	0	Low-quality	10.92
Sample_T11B_k141_146659	9253	9	0	2	Low-quality	10.92
Sample_T7C_k141_72347	8238	6	0	1	Low-quality	10.92

Sample_C13B_k141_38952	6354	5	1	0	Low-quality	10.91
Sample_T17C_k141_6150	5749	10	0	0	Low-quality	10.91
Sample_C1B_k141_71236	14846	21	0	1	Low-quality	10.91
Sample_C8B_k141_81377	5705	11	5	0	Low-quality	10.91
Sample_T8A_k141_179071	8907	10	0	4	Low-quality	10.9
Sample_C6A_k141_224157	8167	13	4	1	Low-quality	10.9
Sample_T6B_k141_12917	7914	13	4	0	Low-quality	10.9
Sample_C4B_k141_132337	5165	7	6	0	Low-quality	10.89
Sample_T6A_k141_141137	5966	7	1	1	Low-quality	10.88
Sample_C11A_k141_23624	6707	10	3	0	Low-quality	10.88
Sample_C10A_k141_286443	5180	9	0	0	Low-quality	10.87
Sample_C11C_k141_105298	5981	6	1	0	Low-quality	10.87
Sample_C1A_k141_262670	5396	7	0	0	Low-quality	10.87
Sample_T6C_k141_101579	17134	19	1	12	Low-quality	10.87
Sample_C14A_k141_56079	7317	7	3	0	Low-quality	10.87
Sample_C1C_k141_138567	6405	6	0	0	Low-quality	10.87
Sample_T6A_k141_42310	12142	15	1	2	Low-quality	10.87
Sample_T12B_k141_26454	6633	6	0	2	Low-quality	10.86
Sample_T6C_k141_201251	7179	9	1	3	Low-quality	10.86
Sample_C3A_k141_122814	6791	13	8	0	Low-quality	10.86
Sample_C4B_k141_232	5622	12	2	0	Low-quality	10.86
Sample_T13B_k141_108011	5030	8	2	0	Low-quality	10.86
Sample_T7B_k141_109887	7176	11	1	0	Low-quality	10.86
Sample_C4A_k141_60278	18001	15	0	10	Low-quality	10.85
Sample_C4B_k141_111853	5307	4	0	3	Low-quality	10.85
Sample_C8B_k141_93043	8389	12	0	7	Low-quality	10.85
Sample_T18B_k141_47990	8900	8	2	2	Low-quality	10.85
Sample_T4A_k141_37789	22093	21	1	4	Low-quality	10.84
Sample_T7B_k141_118504	10736	12	3	0	Low-quality	10.84
Sample_T18C_k141_70536	7505	8	0	0	Low-quality	10.83
Sample_T8B_k141_133845	8908	10	4	1	Low-quality	10.83
Sample_C2B_k141_330272	6024	4	0	4	Low-quality	10.82
Sample_T11B_k141_2071	5150	6	0	2	Low-quality	10.82
Sample_T5B_k141_58422	11227	14	0	4	Low-quality	10.82
Sample_C1B_k141_178397	5805	8	2	0	Low-quality	10.82
Sample_C6C_k141_233948	5237	5	1	0	Low-quality	10.82
Sample_T14B_k141_144791	6062	11	2	0	Low-quality	10.82
Sample_C12B_k141_86779	20930	15	1	4	Low-quality	10.81
Sample_T13B_k141_19855	6472	11	0	1	Low-quality	10.81

Sample_C4C_k141_40129	5802	8	2	0	Low-quality	10.81
Sample_T12B_k141_122960_fragment_1	7388	10	2	0	Low-quality	10.81
Sample_C9C_k141_17553	7736	6	0	1	Low-quality	10.8
Sample_T15C_k141_45866	5399	7	1	0	Low-quality	10.8
Sample_T13B_k141_181728	7239	6	2	0	Low-quality	10.79
Sample_T7A_k141_49753	5329	12	4	1	Low-quality	10.79
Sample_T11C_k141_125662	8009	10	0	6	Low-quality	10.78
Sample_T6A_k141_12347	17355	42	0	0	Low-quality	10.78
Sample_C1B_k141_175648	6939	9	0	1	Low-quality	10.78
Sample_C1B_k141_214568	5588	4	0	0	Low-quality	10.78
Sample_C2A_k141_327944	16317	29	3	0	Low-quality	10.78
Sample_T14B_k141_193458	12333	13	2	0	Low-quality	10.78
Sample_C14C_k141_19357	11988	7	0	1	Low-quality	10.77
Sample_C13C_k141_4619	9466	7	4	1	Low-quality	10.77
Sample_C3A_k141_255007_fragment_1	11721	17	6	0	Low-quality	10.77
Sample_C3B_k141_249871	6209	17	1	0	Low-quality	10.77
Sample_C4B_k141_66851	8579	7	0	1	Low-quality	10.77
Sample_T12C_k141_39889	5472	8	0	1	Low-quality	10.77
Sample_C14A_k141_51942	11820	15	1	3	Low-quality	10.76
Sample_C7A_k141_8656	21945	23	1	1	Low-quality	10.76
Sample_C8B_k141_45123	19500	15	0	11	Low-quality	10.76
Sample_C9B_k141_75709	26468	31	1	3	Low-quality	10.76
Sample_T15A_k141_22825	6427	9	0	2	Low-quality	10.76
Sample_T18C_k141_142730	8790	14	0	1	Low-quality	10.76
Sample_C11C_k141_79030	10093	14	0	1	Low-quality	10.75
Sample_C6C_k141_183007	37051	32	0	25	Low-quality	10.75
Sample_T12A_k141_9243	22154	18	0	15	Low-quality	10.75
Sample_T12A_k141_329107	18761	23	0	16	Low-quality	10.75
Sample_C5A_k141_234515_fragment_1	7183	11	1	1	Low-quality	10.75
Sample_T14C_k141_51397	6520	7	0	2	Low-quality	10.75
Sample_C11B_k141_117528	5865	6	0	2	Low-quality	10.74
Sample_T15B_k141_127992	11671	7	0	1	Low-quality	10.74
Sample_C6B_k141_180369	9849	12	0	4	Low-quality	10.73
Sample_T2C_k141_61808	6535	11	1	2	Low-quality	10.73
Sample_C11C_k141_140085	10553	6	0	1	Low-quality	10.72
Sample_T10A_k141_96357	6418	10	0	1	Low-quality	10.72
Sample_C3A_k141_241766	7111	12	4	0	Low-quality	10.72
Sample_T7A_k141_120787	5522	4	0	2	Low-quality	10.71
Sample_T14C_k141_138	5574	12	1	0	Low-quality	10.71

Sample_C3B_k141_95206	6565	9	0	3	Low-quality	10.7
Sample_C6C_k141_172435	36183	32	1	18	Low-quality	10.7
Sample_T14A_k141_228429	18680	18	0	17	Low-quality	10.7
Sample_T1C_k141_59755	15953	21	1	0	Low-quality	10.7
Sample_C6C_k141_126949	10921	7	0	1	Low-quality	10.7
Sample_T11B_k141_62063	5198	4	1	0	Low-quality	10.7
Sample_C3A_k141_251233	13611	12	1	2	Low-quality	10.69
Sample_T1B_k141_1942	19807	17	0	14	Low-quality	10.69
Sample_C10B_k141_35500	6465	8	5	0	Low-quality	10.69
Sample_C2B_k141_316572_fragment_1	5946	10	0	0	Low-quality	10.69
Sample_T10B_k141_49163_fragment_1	14895	16	1	2	Low-quality	10.69
Sample_T12A_k141_219241	18972	17	0	12	Low-quality	10.68
Sample_T3C_k141_7304	9151	10	1	4	Low-quality	10.68
Sample_T6A_k141_233075	26602	33	1	5	Low-quality	10.68
Sample_T15A_k141_101689	5906	6	3	0	Low-quality	10.68
Sample_T5C_k141_25455	5036	7	3	0	Low-quality	10.68
Sample_T8B_k141_141326	5744	7	0	0	Low-quality	10.68
Sample_T12A_k141_107655	8050	3	0	1	Low-quality	10.67
Sample_T17B_k141_11804	5569	9	3	0	Low-quality	10.67
Sample_C7A_k141_177230	6271	7	0	4	Low-quality	10.66
Sample_C7C_k141_36730	5353	6	1	1	Low-quality	10.66
Sample_T12C_k141_164788	5582	10	0	2	Low-quality	10.66
Sample_T8B_k141_88917	21729	25	1	3	Low-quality	10.66
Sample_T8C_k141_51982	8641	9	0	2	Low-quality	10.66
Sample_C12B_k141_140393	5044	9	8	0	Low-quality	10.66
Sample_C4C_k141_81035	7837	17	8	0	Low-quality	10.66
Sample_C7C_k141_50273	6834	6	1	0	Low-quality	10.66
Sample_C8B_k141_194915	7427	21	2	0	Low-quality	10.66
Sample_T2B_k141_305057	5082	9	1	0	Low-quality	10.66
Sample_T12C_k141_52625	15602	19	2	5	Low-quality	10.65
Sample_C3A_k141_157929	6729	11	3	0	Low-quality	10.65
Sample_C4B_k141_25666	6558	15	2	1	Low-quality	10.65
Sample_T14C_k141_58241_fragment_1	5691	9	2	0	Low-quality	10.65
Sample_C6B_k141_59641	8225	8	0	6	Low-quality	10.64
Sample_T16B_k141_2819	6370	9	0	1	Low-quality	10.64
Sample_C12C_k141_55055	7669	6	0	4	Low-quality	10.64
Sample_C11C_k141_69846	12221	12	0	7	Low-quality	10.63
Sample_C3A_k141_26979	12293	17	1	1	Low-quality	10.63
Sample_C8A_k141_177341	8686	10	0	4	Low-quality	10.63

Sample_C3C_k141_74982	5539	12	3	0	Low-quality	10.63
Sample_T15B_k141_67951	5759	11	0	0	Low-quality	10.63
Sample_C9B_k141_38	6218	8	1	0	Low-quality	10.62
Sample_T16C_k141_114670	5270	7	0	0	Low-quality	10.62
Sample_T2C_k141_121839	6179	9	0	1	Low-quality	10.62
Sample_C8C_k141_97399	7967	22	1	0	Low-quality	10.62
Sample_T18A_k141_68180	6181	11	3	0	Low-quality	10.62
Sample_T2A_k141_57641	5155	10	8	0	Low-quality	10.62
Sample_C7B_k141_135738	5272	6	1	0	Low-quality	10.61
Sample_T8B_k141_215512	5031	12	1	0	Low-quality	10.61
Sample_T14C_k141_39973	12363	12	0	3	Low-quality	10.6
Sample_T17C_k141_37585	6347	7	0	3	Low-quality	10.6
Sample_C11A_k141_118592	5102	13	2	0	Low-quality	10.6
Sample_C2C_k141_44698	10937	6	0	6	Low-quality	10.59
Sample_C2C_k141_174499	14857	17	1	1	Low-quality	10.59
Sample_C5A_k141_88745	6343	7	0	2	Low-quality	10.59
Sample_T10A_k141_113146	16284	15	0	14	Low-quality	10.59
Sample_T8B_k141_203634	5288	7	1	0	Low-quality	10.59
Sample_T14A_k141_205260	8877	9	0	2	Low-quality	10.58
Sample_C2A_k141_98847	8981	13	4	0	Low-quality	10.58
Sample_C2C_k141_283766	10493	6	1	0	Low-quality	10.58
Sample_C5C_k141_5767	5625	9	6	0	Low-quality	10.58
Sample_C5B_k141_620	7794	7	0	1	Low-quality	10.57
Sample_T15B_k141_14553	5037	13	1	0	Low-quality	10.57
Sample_T2C_k141_124255	6147	14	2	0	Low-quality	10.57
Sample_C4C_k141_5105	6867	7	0	0	Low-quality	10.56
Sample_C4C_k141_193982	11397	10	1	2	Low-quality	10.56
Sample_T12C_k141_46424	6061	7	0	0	Low-quality	10.56
Sample_T2B_k141_68293	5915	10	3	0	Low-quality	10.56
Sample_T7C_k141_17824	6411	10	0	0	Low-quality	10.56
Sample_C12A_k141_193606	6970	7	1	0	Low-quality	10.55
Sample_C6C_k141_35930	9689	10	0	7	Low-quality	10.55
Sample_T11C_k141_152719	7816	12	3	1	Low-quality	10.55
Sample_T13C_k141_77708	6772	12	1	1	Low-quality	10.55
Sample_T18C_k141_128448	19458	25	1	2	Low-quality	10.55
Sample_T4B_k141_89392	10623	10	0	3	Low-quality	10.55
Sample_C3A_k141_170552	14164	8	2	1	Low-quality	10.55
Sample_T13B_k141_25618	5995	10	2	0	Low-quality	10.55
Sample_T17A_k141_14664	5221	9	1	0	Low-quality	10.55

Sample_T6C_k141_213304	6380	18	3	1	Low-quality	10.55
Sample_C4C_k141_60236	7132	7	0	4	Low-quality	10.54
Sample_C11A_k141_107131	5740	6	0	1	Low-quality	10.54
Sample_C2A_k141_162545	7965	13	1	1	Low-quality	10.54
Sample_C3C_k141_193735	6070	16	3	0	Low-quality	10.54
Sample_T5A_k141_6074	7762	10	3	2	Low-quality	10.54
Sample_C5C_k141_111749	5232	7	4	0	Low-quality	10.53
Sample_C7A_k141_787	8668	5	3	0	Low-quality	10.53
Sample_T3C_k141_72626	7260	7	0	0	Low-quality	10.53
Sample_T16C_k141_16622	21492	18	1	15	Low-quality	10.52
Sample_T3A_k141_54443	5757	7	0	1	Low-quality	10.52
Sample_T11B_k141_146996	6307	7	0	0	Low-quality	10.52
Sample_T12A_k141_156770	5701	4	2	0	Low-quality	10.52
Sample_T15A_k141_113721	6748	9	0	0	Low-quality	10.52
Sample_T16A_k141_134102	6261	8	0	0	Low-quality	10.52
Sample_T16B_k141_127735	5439	4	4	0	Low-quality	10.52
Sample_C5A_k141_234159	65812	65	0	50	Low-quality	10.51
Sample_C12A_k141_142220_fragment_1	15154	14	2	1	Low-quality	10.51
Sample_T12B_k141_138474	5631	12	5	0	Low-quality	10.51
Sample_T5A_k141_39330	9706	13	6	0	Low-quality	10.51
Sample_C9C_k141_49556	15274	22	0	3	Low-quality	10.5
Sample_T10A_k141_124599	6286	7	0	1	Low-quality	10.5
Sample_T11C_k141_81363	7191	11	0	1	Low-quality	10.5
Sample_T12A_k141_280954	20346	16	1	10	Low-quality	10.5
Sample_C10C_k141_183859	8844	12	0	0	Low-quality	10.5
Sample_T5A_k141_19383	6892	10	8	0	Low-quality	10.5
Sample_C10A_k141_297942	5160	4	0	0	Low-quality	10.49
Sample_C12A_k141_197491	7468	7	1	1	Low-quality	10.49
Sample_C2A_k141_217611	5036	12	1	0	Low-quality	10.49
Sample_T12A_k141_52221	10189	11	0	0	Low-quality	10.49
Sample_T12A_k141_338323	7461	15	5	0	Low-quality	10.49
Sample_T2B_k141_45058	6096	11	1	0	Low-quality	10.49
Sample_T10B_k141_32600	8572	2	1	0	Low-quality	10.48
Sample_C8C_k141_159076	5976	4	0	3	Low-quality	10.47
Sample_T18B_k141_4075	9226	13	1	4	Low-quality	10.47
Sample_T8C_k141_62371	6271	13	0	0	Low-quality	10.47
Sample_T3A_k141_49342	5499	11	1	0	Low-quality	10.47
Sample_T8C_k141_204428	5256	9	4	0	Low-quality	10.47
Sample_T16B_k141_131425	6701	8	1	5	Low-quality	10.46

Sample_T2A_k141_278784	5742	3	0	1	Low-quality	10.46
Sample_T8C_k141_115538	5223	11	1	2	Low-quality	10.46
Sample_C6C_k141_98755	6299	7	5	0	Low-quality	10.46
Sample_C8B_k141_112630	5515	11	0	0	Low-quality	10.46
Sample_T16C_k141_110290	6123	5	1	0	Low-quality	10.46
Sample_C4A_k141_65995	7945	12	0	1	Low-quality	10.45
Sample_T5A_k141_2767	8525	8	0	3	Low-quality	10.45
Sample_C1B_k141_108956	10536	4	3	0	Low-quality	10.45
Sample_C3A_k141_163207	5417	8	4	1	Low-quality	10.45
Sample_C7C_k141_27917	5610	8	1	0	Low-quality	10.45
Sample_T10B_k141_16625	5226	8	0	0	Low-quality	10.45
Sample_C4B_k141_163832	6472	5	0	1	Low-quality	10.44
Sample_C5A_k141_282766	14735	21	2	0	Low-quality	10.44
Sample_C7C_k141_83546	6561	7	4	0	Low-quality	10.44
Sample_C8B_k141_115961	7802	7	0	0	Low-quality	10.44
Sample_T14B_k141_141330	17986	26	5	1	Low-quality	10.44
Sample_C12A_k141_108115	20096	15	0	13	Low-quality	10.43
Sample_T16B_k141_87315	16570	26	0	0	Low-quality	10.43
Sample_T14B_k141_26743	5722	9	2	0	Low-quality	10.43
Sample_T8C_k141_77862	8273	15	1	0	Low-quality	10.43
Sample_C13A_k141_15570	18193	24	0	17	Low-quality	10.42
Sample_C5A_k141_266129	6738	9	0	1	Low-quality	10.42
Sample_T12C_k141_150122	5813	6	0	1	Low-quality	10.42
Sample_T2C_k141_117792	7987	11	1	1	Low-quality	10.42
Sample_C2C_k141_68794	11017	10	1	0	Low-quality	10.42
Sample_T6A_k141_147216	19043	46	1	0	Low-quality	10.42
Sample_C4C_k141_38566	7426	9	1	2	Low-quality	10.41
Sample_T12A_k141_46685	11336	10	0	5	Low-quality	10.41
Sample_T16B_k141_53057	7243	10	0	4	Low-quality	10.41
Sample_T2B_k141_151162	5342	3	2	0	Low-quality	10.41
Sample_C8C_k141_110770	8309	6	0	2	Low-quality	10.4
Sample_T14A_k141_180327	6017	7	0	1	Low-quality	10.4
Sample_T3C_k141_59509	39036	34	0	23	Low-quality	10.4
Sample_C6B_k141_203082	6222	12	0	3	Low-quality	10.39
Sample_T11B_k141_146506	6626	11	0	5	Low-quality	10.39
Sample_C2B_k141_318492	5445	6	3	0	Low-quality	10.39
Sample_T8B_k141_278685	5676	15	1	0	Low-quality	10.39
Sample_T17B_k141_32999	20832	19	2	2	Low-quality	10.38
Sample_T13B_k141_159368	8545	7	3	1	Low-quality	10.38

Sample_C7C_k141_44225	9064	13	0	2	Low-quality	10.37
Sample_T12B_k141_19977	8629	7	0	1	Low-quality	10.37
Sample_C11C_k141_13401	29084	24	1	6	Low-quality	10.36
Sample_C2B_k141_80952	5708	7	0	2	Low-quality	10.36
Sample_C4A_k141_15465	6776	10	2	0	Low-quality	10.36
Sample_T3B_k141_57977	7623	8	0	2	Low-quality	10.36
Sample_T6B_k141_148287	16013	18	1	4	Low-quality	10.36
Sample_C1A_k141_173110	9569	14	1	1	Low-quality	10.35
Sample_C6B_k141_196406	8900	10	3	3	Low-quality	10.35
Sample_C6B_k141_121585	8461	8	0	0	Low-quality	10.35
Sample_T13B_k141_97246	18074	16	0	12	Low-quality	10.35
Sample_T2B_k141_255639	6148	10	1	1	Low-quality	10.35
Sample_T4B_k141_95416	25143	35	1	6	Low-quality	10.35
Sample_C14A_k141_108148	5598	12	1	0	Low-quality	10.35
Sample_C4B_k141_51654	5792	6	3	0	Low-quality	10.35
Sample_T11C_k141_15278	6345	10	1	0	Low-quality	10.35
Sample_T16A_k141_84965	5347	5	2	0	Low-quality	10.35
Sample_C10A_k141_210664	14295	11	0	4	Low-quality	10.34
Sample_C2B_k141_138812	7771	3	0	1	Low-quality	10.34
Sample_C3A_k141_62053	14966	14	1	4	Low-quality	10.34
Sample_C5A_k141_43035	11605	10	0	4	Low-quality	10.34
Sample_C9B_k141_88014	22297	38	1	1	Low-quality	10.34
Sample_T14B_k141_88948	25091	28	1	7	Low-quality	10.34
Sample_C4A_k141_44749	5604	13	1	0	Low-quality	10.34
Sample_C6C_k141_70563	11342	13	1	3	Low-quality	10.33
Sample_T4B_k141_154996	9531	19	2	1	Low-quality	10.33
Sample_C1C_k141_114953	11815	17	2	0	Low-quality	10.32
Sample_T10B_k141_60492	7834	1	0	0	Low-quality	10.32
Sample_T14A_k141_10559	13317	8	0	6	Low-quality	10.32
Sample_T4B_k141_252660	8433	10	0	2	Low-quality	10.32
Sample_T8C_k141_179460_fragment_1	9793	11	1	1	Low-quality	10.32
Sample_T16B_k141_84288	5962	9	0	5	Low-quality	10.31
Sample_T7A_k141_39628	5359	6	0	1	Low-quality	10.31
Sample_C14B_k141_57344	15846	11	1	0	Low-quality	10.31
Sample_C4C_k141_26448	6867	3	2	1	Low-quality	10.3
Sample_T12C_k141_183637	6493	7	0	2	Low-quality	10.3
Sample_T7B_k141_35851	10009	4	0	1	Low-quality	10.3
Sample_C3A_k141_18533	6697	11	5	0	Low-quality	10.3
Sample_C3C_k141_223757	5093	8	1	0	Low-quality	10.3

Sample_T7A_k141_228001	5739	13	2	0	Low-quality	10.3
Sample_T8A_k141_21267	6162	15	1	0	Low-quality	10.3
Sample_C12C_k141_176472	5757	5	0	0	Low-quality	10.29
Sample_C9A_k141_100938	12544	15	2	3	Low-quality	10.29
Sample_T12C_k141_177185	8785	12	0	1	Low-quality	10.29
Sample_C3A_k141_78725_fragment_1	7655	10	1	1	Low-quality	10.29
Sample_T12A_k141_304198	5649	6	0	0	Low-quality	10.29
Sample_T12A_k141_4908	6599	9	0	0	Low-quality	10.29
Sample_C11C_k141_126420	5737	12	1	0	Low-quality	10.28
Sample_C13A_k141_88681	5029	11	1	0	Low-quality	10.28
Sample_T11A_k141_97773	5442	6	2	0	Low-quality	10.28
Sample_C6C_k141_151734	6900	11	1	6	Low-quality	10.27
Sample_T14B_k141_13996	16793	13	2	0	Low-quality	10.27
Sample_C1C_k141_169557	8514	5	0	0	Low-quality	10.26
Sample_C5B_k141_81890	15471	19	0	6	Low-quality	10.26
Sample_C9B_k141_64129	6071	10	5	0	Low-quality	10.26
Sample_C8A_k141_72717	6235	8	0	5	Low-quality	10.25
Sample_C13C_k141_91420	6173	6	1	1	Low-quality	10.25
Sample_C8B_k141_144874	7032	11	1	0	Low-quality	10.25
Sample_C2C_k141_129601	18875	25	1	3	Low-quality	10.24
Sample_C8A_k141_164890	5479	4	1	0	Low-quality	10.24
Sample_T3A_k141_97709	18599	25	0	15	Low-quality	10.24
Sample_T3C_k141_122165	5882	5	0	1	Low-quality	10.24
Sample_T7B_k141_49023	11062	7	0	2	Low-quality	10.23
Sample_C11B_k141_208442	5938	11	2	0	Low-quality	10.23
Sample_C12A_k141_7013	5453	5	0	0	Low-quality	10.23
Sample_C13C_k141_74614	10175	18	2	0	Low-quality	10.23
Sample_T16B_k141_46877	10032	17	0	0	Low-quality	10.23
Sample_T7A_k141_176390_fragment_1	6238	9	6	0	Low-quality	10.23
Sample_C14A_k141_111	6517	10	0	2	Low-quality	10.22
Sample_C1A_k141_139417	6753	5	1	1	Low-quality	10.22
Sample_T15A_k141_32393	10778	7	0	3	Low-quality	10.22
Sample_T3B_k141_131320	5584	5	0	2	Low-quality	10.22
Sample_C14A_k141_123368	5520	8	1	2	Low-quality	10.22
Sample_C4C_k141_18562	5504	12	3	0	Low-quality	10.22
Sample_C9A_k141_52107	6754	9	1	0	Low-quality	10.22
Sample_C11A_k141_178451	5177	7	4	0	Low-quality	10.21
Sample_T16A_k141_109149_fragment_1	8404	16	4	3	Low-quality	10.21
Sample_T1B_k141_14686	7390	8	0	0	Low-quality	10.21

Sample_T6C_k141_174993	5091	7	3	0	Low-quality	10.21
Sample_T6C_k141_86966	6177	13	2	0	Low-quality	10.21
Sample_C8A_k141_46673	14157	15	1	3	Low-quality	10.2
Sample_C10C_k141_191243	5975	10	2	0	Low-quality	10.2
Sample_C5A_k141_191685	6786	7	0	0	Low-quality	10.2
Sample_T13B_k141_50598	6933	5	3	0	Low-quality	10.19
Sample_C10A_k141_145155	5696	6	1	3	Low-quality	10.18
Sample_C11A_k141_13367	7971	8	0	1	Low-quality	10.18
Sample_C11A_k141_58407	17763	21	0	18	Low-quality	10.17
Sample_C14C_k141_48133	11537	13	0	7	Low-quality	10.17
Sample_C1B_k141_93401	7821	5	0	1	Low-quality	10.17
Sample_C1C_k141_24189	7301	7	0	2	Low-quality	10.17
Sample_T7C_k141_150429	5167	7	0	1	Low-quality	10.17
Sample_T8C_k141_8441	6441	3	0	0	Low-quality	10.17
Sample_C8B_k141_85609	5329	5	4	0	Low-quality	10.17
Sample_T11A_k141_13137	6989	9	0	2	Low-quality	10.17
Sample_C3B_k141_126532	6083	9	1	0	Low-quality	10.16
Sample_T11C_k141_43232	8303	10	0	3	Low-quality	10.16
Sample_C10B_k141_61725	6147	7	2	0	Low-quality	10.16
Sample_C5A_k141_221148	5801	10	2	0	Low-quality	10.16
Sample_C8B_k141_190220	5149	4	2	0	Low-quality	10.16
Sample_T10B_k141_16510	5474	12	0	0	Low-quality	10.16
Sample_T7C_k141_92633	13535	14	7	0	Low-quality	10.16
Sample_C2B_k141_116563	18723	26	1	2	Low-quality	10.15
Sample_C8A_k141_167281	7271	17	4	0	Low-quality	10.15
Sample_C8A_k141_157689	7966	6	2	0	Low-quality	10.15
Sample_T10A_k141_38803	6019	6	5	0	Low-quality	10.15
Sample_C1C_k141_93310	12433	14	0	7	Low-quality	10.14
Sample_C7C_k141_23479	17864	31	6	0	Low-quality	10.14
Sample_C11A_k141_84568	6489	7	1	0	Low-quality	10.13
Sample_C11B_k141_25843	6073	12	0	0	Low-quality	10.12
Sample_T18C_k141_131197	9631	20	5	2	Low-quality	10.12
Sample_T5A_k141_45003	9504	13	6	1	Low-quality	10.12
Sample_C10C_k141_53654	11815	16	0	3	Low-quality	10.11
Sample_C4C_k141_242780	15599	15	0	5	Low-quality	10.11
Sample_T12A_k141_252717	14254	23	3	8	Low-quality	10.11
Sample_T5B_k141_77059	10976	11	0	6	Low-quality	10.11
Sample_T16B_k141_80271	6218	6	2	2	Low-quality	10.11
Sample_T7C_k141_68380	24655	19	7	1	Low-quality	10.11

Sample_T7A_k141_218296	14022	14	1	2	Low-quality	10.1
Sample_C5C_k141_56830	5377	8	1	0	Low-quality	10.1
Sample_T2C_k141_42460	22368	25	1	7	Low-quality	10.09
Sample_C5C_k141_114608	5156	7	4	0	Low-quality	10.09
Sample_T16C_k141_135774	6247	14	0	0	Low-quality	10.09
Sample_T7A_k141_227182	6876	18	3	0	Low-quality	10.09
Sample_T16C_k141_87745	9791	9	0	8	Low-quality	10.08
Sample_C2B_k141_2315	5893	12	1	0	Low-quality	10.08
Sample_C2B_k141_158753	5403	8	0	0	Low-quality	10.08
Sample_T14A_k141_68811	6172	4	4	0	Low-quality	10.08
Sample_C3B_k141_184467	5690	10	0	0	Low-quality	10.07
Sample_C14A_k141_162772	10772	13	4	0	Low-quality	10.07
Sample_C5A_k141_31776	5301	6	0	0	Low-quality	10.07
Sample_C8A_k141_3930	5719	8	0	1	Low-quality	10.07
Sample_T7B_k141_62867	8125	10	1	3	Low-quality	10.06
Sample_T18B_k141_49297	8890	15	0	0	Low-quality	10.06
Sample_T8C_k141_52583	5092	7	4	0	Low-quality	10.06
Sample_T2A_k141_205334	17524	21	3	0	Low-quality	10.05
Sample_C11B_k141_23217	13433	10	0	6	Low-quality	10.04
Sample_C1B_k141_67391	10851	11	0	2	Low-quality	10.04
Sample_T3A_k141_55188	53805	55	3	16	Low-quality	10.04
Sample_T8C_k141_220767	15142	18	2	0	Low-quality	10.04
Sample_C5A_k141_205989	5684	8	0	1	Low-quality	10.03
Sample_C8B_k141_26442	6066	13	2	0	Low-quality	10.03
Sample_T10B_k141_181447	5379	9	4	0	Low-quality	10.03
Sample_T13C_k141_80106	6071	7	1	2	Low-quality	10.02
Sample_T8A_k141_9983	5820	7	1	0	Low-quality	10.02
Sample_T13B_k141_177176	54834	52	1	37	Low-quality	10.01
Sample_T14B_k141_164611	20057	15	1	8	Low-quality	10.01
Sample_T6A_k141_248735	5343	6	1	3	Low-quality	10.01
Sample_C3C_k141_122200	6854	9	2	1	Low-quality	10.01
Sample_C7C_k141_86355	11824	17	2	0	Low-quality	10.01
Sample_T13B_k141_64816	5985	7	0	0	Low-quality	10
Sample_T15C_k141_68529	7988	5	0	2	Low-quality	10
Sample_C13A_k141_108524	7557	18	1	0	Low-quality	10
Sample_C14B_k141_37982	9345	13	2	1	Low-quality	10
Sample_C8C_k141_141863	6237	6	0	1	Low-quality	10
Sample_C10A_k141_139185	5979	5	0	2	Low-quality	9.99
Sample_T6C_k141_99583	6604	8	1	3	Low-quality	9.99

Sample_C11B_k141_129032	5468	6	0	1	Low-quality	9.99
Sample_T11A_k141_38073_fragment_1	8975	12	2	0	Low-quality	9.99
Sample_T12A_k141_118421	5503	8	1	0	Low-quality	9.99
Sample_C10A_k141_38141	12265	13	0	5	Low-quality	9.98
Sample_T10B_k141_153423	5660	7	0	1	Low-quality	9.98
Sample_T14A_k141_101210	5347	7	0	0	Low-quality	9.98
Sample_T3B_k141_77536	11428	14	3	0	Low-quality	9.98
Sample_C6B_k141_189348	6615	8	1	0	Low-quality	9.98
Sample_T5A_k141_16823	9890	10	2	0	Low-quality	9.98
Sample_T8B_k141_21613	8389	16	3	1	Low-quality	9.98
Sample_C7C_k141_40749	47622	50	2	13	Low-quality	9.97
Sample_T8A_k141_13276	17398	12	0	11	Low-quality	9.97
Sample_C6B_k141_203914	6030	8	7	0	Low-quality	9.97
Sample_T5C_k141_181659	6404	8	0	1	Low-quality	9.97
Sample_C10A_k141_84287	20303	17	0	9	Low-quality	9.96
Sample_C10C_k141_71377	5961	4	0	1	Low-quality	9.96
Sample_T17C_k141_29200	14505	9	0	9	Low-quality	9.96
Sample_C10C_k141_143104	5713	9	7	0	Low-quality	9.96
Sample_C7A_k141_108618	8250	12	2	0	Low-quality	9.96
Sample_T8C_k141_16639	5687	11	10	0	Low-quality	9.96
Sample_C12A_k141_54384	7959	9	0	2	Low-quality	9.95
Sample_C1C_k141_161007	8131	7	0	2	Low-quality	9.95
Sample_C2C_k141_157729	12396	14	1	6	Low-quality	9.95
Sample_C6C_k141_44832	6574	7	1	0	Low-quality	9.95
Sample_T1B_k141_1283	5473	6	0	1	Low-quality	9.95
Sample_T8B_k141_96194	8128	8	0	4	Low-quality	9.95
Sample_C7C_k141_158	5599	5	1	0	Low-quality	9.95
Sample_C13A_k141_41684	6688	13	3	0	Low-quality	9.94
Sample_C1C_k141_156234	16956	15	1	9	Low-quality	9.93
Sample_C5A_k141_70987	18234	19	3	3	Low-quality	9.93
Sample_C2C_k141_79370	5789	9	6	0	Low-quality	9.93
Sample_C3C_k141_208173	6115	9	4	0	Low-quality	9.93
Sample_C7B_k141_114043	8157	12	0	2	Low-quality	9.93
Sample_C7B_k141_181030_fragment_3	7393	9	1	1	Low-quality	9.93
Sample_T2C_k141_36905	7586	6	2	1	Low-quality	9.92
Sample_C14A_k141_151024	8167	10	3	0	Low-quality	9.92
Sample_T6A_k141_158819	6782	12	2	0	Low-quality	9.92
Sample_T6A_k141_179110	5729	11	2	0	Low-quality	9.92
Sample_C3A_k141_236319	5932	6	0	2	Low-quality	9.91

Sample_C12B_k141_153744	7456	7	4	2	Low-quality	9.91
Sample_T11A_k141_76556	8826	9	1	0	Low-quality	9.91
Sample_T4C_k141_39810	9302	6	0	1	Low-quality	9.91
Sample_C2A_k141_59249	9615	19	5	0	Low-quality	9.9
Sample_C10B_k141_143758	6271	9	0	3	Low-quality	9.89
Sample_C6B_k141_30737	7054	8	0	1	Low-quality	9.89
Sample_T14C_k141_78417	17666	18	1	8	Low-quality	9.89
Sample_T6B_k141_6592	5411	8	0	0	Low-quality	9.89
Sample_T6C_k141_256659	10601	10	0	5	Low-quality	9.89
Sample_C10A_k141_8694	6086	9	8	0	Low-quality	9.89
Sample_C11A_k141_57236	12910	13	6	0	Low-quality	9.89
Sample_C11A_k141_62449	5337	6	1	1	Low-quality	9.89
Sample_C2B_k141_330937	8598	8	0	1	Low-quality	9.89
Sample_T10A_k141_2608	13219	22	0	1	Low-quality	9.89
Sample_T7A_k141_53918	6249	12	0	0	Low-quality	9.89
Sample_C1A_k141_118293	5402	8	0	2	Low-quality	9.88
Sample_T2B_k141_90114	20153	22	1	4	Low-quality	9.88
Sample_T14A_k141_237939	11129	15	2	0	Low-quality	9.88
Sample_C14A_k141_27706	64098	57	2	27	Low-quality	9.87
Sample_C7B_k141_165133	10664	8	0	4	Low-quality	9.87
Sample_T12C_k141_119668	17426	10	4	0	Low-quality	9.87
Sample_C9A_k141_68315	9501	7	0	4	Low-quality	9.86
Sample_T18B_k141_1106	7527	10	1	7	Low-quality	9.86
Sample_C3A_k141_27041	5965	8	3	0	Low-quality	9.86
Sample_C5C_k141_56739	13839	16	2	1	Low-quality	9.86
Sample_T13C_k141_2540	8120	5	3	0	Low-quality	9.86
Sample_T4B_k141_85772	17436	19	0	2	Low-quality	9.86
Sample_C3B_k141_226768	5157	6	1	0	Low-quality	9.85
Sample_C7A_k141_127808	5452	3	3	0	Low-quality	9.85
Sample_C13C_k141_34420	9703	4	0	0	Low-quality	9.85
Sample_C2A_k141_123757	6108	6	0	0	Low-quality	9.85
Sample_T2C_k141_2469	18148	23	1	3	Low-quality	9.84
Sample_T6B_k141_106450	6506	12	1	2	Low-quality	9.84
Sample_T6C_k141_181522	5945	7	5	0	Low-quality	9.84
Sample_T17B_k141_10892	6843	9	1	2	Low-quality	9.83
Sample_C12A_k141_22669_fragment_1	9728	17	2	0	Low-quality	9.83
Sample_C5A_k141_20626	5192	11	4	0	Low-quality	9.83
Sample_C11C_k141_68690	8408	7	0	2	Low-quality	9.82
Sample_T8B_k141_249323	5270	4	0	0	Low-quality	9.82

Sample_T12C_k141_32140	8773	14	1	1	Low-quality	9.82
Sample_T8A_k141_150662	5340	15	2	0	Low-quality	9.82
Sample_C6C_k141_187241	5882	7	1	1	Low-quality	9.81
Sample_T6C_k141_197839	15548	13	0	10	Low-quality	9.81
Sample_C12C_k141_211545	7370	6	1	1	Low-quality	9.81
Sample_T10B_k141_73214	5087	13	2	0	Low-quality	9.81
Sample_C10B_k141_87260	5870	8	0	2	Low-quality	9.8
Sample_C12A_k141_68614	10324	9	1	1	Low-quality	9.8
Sample_C1C_k141_42059	9580	8	0	2	Low-quality	9.8
Sample_C4B_k141_124923	5568	5	0	0	Low-quality	9.8
Sample_T11B_k141_50029	6546	12	0	2	Low-quality	9.8
Sample_T12A_k141_340670	14579	13	1	4	Low-quality	9.8
Sample_T8B_k141_76625	5254	5	0	2	Low-quality	9.8
Sample_T12C_k141_159188	7724	12	6	0	Low-quality	9.8
Sample_T1B_k141_9669	5244	9	3	0	Low-quality	9.8
Sample_C1B_k141_62840	6032	7	0	0	Low-quality	9.79
Sample_C7A_k141_83727	5864	8	0	2	Low-quality	9.79
Sample_T10A_k141_96625	7911	8	0	6	Low-quality	9.79
Sample_T12B_k141_164981	5712	11	1	0	Low-quality	9.79
Sample_T2C_k141_172591	7986	8	0	1	Low-quality	9.79
Sample_T13A_k141_42944	5841	10	3	0	Low-quality	9.79
Sample_T10A_k141_172908	5494	9	1	0	Low-quality	9.78
Sample_T15C_k141_84533_fragment_2	8689	12	3	0	Low-quality	9.78
Sample_T4C_k141_109405	5279	7	1	0	Low-quality	9.78
Sample_C3A_k141_253064	16600	15	2	8	Low-quality	9.77
Sample_T13B_k141_159968	6254	5	0	0	Low-quality	9.77
Sample_T15B_k141_17934	6882	9	0	2	Low-quality	9.77
Sample_T8A_k141_34842	6893	17	3	0	Low-quality	9.77
Sample_T3B_k141_100538	15467	10	1	6	Low-quality	9.76
Sample_T8A_k141_43688	6047	8	0	0	Low-quality	9.75
Sample_C10C_k141_184092	17007	17	0	13	Low-quality	9.74
Sample_C14C_k141_181012	9797	9	1	3	Low-quality	9.74
Sample_T2B_k141_3039	6116	15	3	0	Low-quality	9.74
Sample_C2C_k141_216874	10296	20	3	0	Low-quality	9.74
Sample_T17B_k141_21326_fragment_1	8047	10	4	3	Low-quality	9.74
Sample_T1C_k141_51054	6437	5	1	0	Low-quality	9.74
Sample_C11A_k141_90914	12388	11	1	3	Low-quality	9.73
Sample_C2B_k141_14286	5765	10	0	2	Low-quality	9.73
Sample_C2B_k141_230958	17759	21	1	9	Low-quality	9.72

Sample_C4A_k141_124140	12168	10	0	5	Low-quality	9.72
Sample_T12A_k141_112165	6855	5	0	2	Low-quality	9.72
Sample_T7A_k141_143918	11086	8	2	2	Low-quality	9.72
Sample_C10C_k141_49848	5791	11	4	0	Low-quality	9.72
Sample_T16C_k141_108639	5971	10	3	0	Low-quality	9.72
Sample_T8B_k141_22202	7514	14	1	0	Low-quality	9.72
Sample_C14C_k141_65900	14999	24	1	3	Low-quality	9.71
Sample_C3A_k141_62183	10484	11	3	2	Low-quality	9.71
Sample_C10A_k141_78431	6410	8	1	1	Low-quality	9.7
Sample_C11C_k141_161144	6412	7	1	2	Low-quality	9.7
Sample_C5A_k141_197996	7924	9	0	1	Low-quality	9.7
Sample_T10B_k141_183359	6908	10	0	3	Low-quality	9.7
Sample_T16C_k141_59823	6503	11	0	0	Low-quality	9.7
Sample_T16C_k141_109155	7764	4	0	2	Low-quality	9.7
Sample_C13A_k141_61176	6412	9	1	0	Low-quality	9.7
Sample_C8C_k141_157115	5176	10	0	0	Low-quality	9.7
Sample_T10B_k141_160054_fragment_1	7526	11	0	0	Low-quality	9.7
Sample_T13B_k141_52098	8794	6	0	0	Low-quality	9.7
Sample_T6C_k141_105509	5261	8	1	0	Low-quality	9.7
Sample_T18A_k141_145365	17861	22	1	2	Low-quality	9.69
Sample_C2B_k141_110923	10842	5	0	0	Low-quality	9.68
Sample_C7B_k141_155517	5136	5	0	2	Low-quality	9.68
Sample_T14A_k141_197479	5399	2	0	1	Low-quality	9.68
Sample_T15C_k141_123515	7736	4	0	2	Low-quality	9.68
Sample_T14B_k141_62566	5966	8	1	0	Low-quality	9.68
Sample_T13B_k141_152288	9922	5	0	3	Low-quality	9.67
Sample_T15C_k141_45291	6550	12	1	0	Low-quality	9.67
Sample_T17C_k141_17338	306980	245	3	193	Low-quality	9.67
Sample_T2A_k141_133634	7813	9	1	1	Low-quality	9.67
Sample_T7B_k141_16913	5390	5	0	2	Low-quality	9.67
Sample_C4A_k141_88257	14401	27	1	0	Low-quality	9.67
Sample_C4B_k141_183425	5187	6	0	1	Low-quality	9.67
Sample_T10A_k141_135151	8617	7	1	0	Low-quality	9.67
Sample_C12B_k141_141640	15923	23	2	2	Low-quality	9.66
Sample_C13A_k141_87100	23952	24	1	21	Low-quality	9.66
Sample_T13A_k141_40731	5291	10	0	0	Low-quality	9.66
Sample_C10A_k141_178754	5429	8	1	0	Low-quality	9.66
Sample_C12A_k141_150476	5680	6	0	0	Low-quality	9.66
Sample_C3C_k141_212365	5855	14	6	0	Low-quality	9.66

Sample_C4B_k141_220479	5749	6	0	2	Low-quality	9.65
Sample_T7A_k141_57862	6181	8	0	0	Low-quality	9.65
Sample_T10A_k141_80497	16011	21	5	0	Low-quality	9.65
Sample_T12A_k141_336416	9224	9	0	1	Low-quality	9.65
Sample_C2C_k141_25895	18489	26	2	14	Low-quality	9.64
Sample_C5A_k141_219525	23407	34	1	5	Low-quality	9.64
Sample_C11B_k141_210180	6269	18	3	0	Low-quality	9.64
Sample_C14C_k141_57842	5130	6	0	1	Low-quality	9.64
Sample_C8B_k141_9884	9191	11	1	0	Low-quality	9.64
Sample_T15A_k141_68932	19683	21	1	2	Low-quality	9.64
Sample_C7B_k141_103764	36305	30	0	23	Low-quality	9.63
Sample_C10C_k141_83499	6445	11	2	0	Low-quality	9.63
Sample_C10B_k141_138406	7702	9	2	0	Low-quality	9.62
Sample_C2C_k141_95606	19039	12	1	2	Low-quality	9.62
Sample_T10A_k141_140066	5362	1	0	1	Low-quality	9.62
Sample_C9A_k141_86482	5146	7	1	0	Low-quality	9.61
Sample_T12C_k141_52287	9297	7	0	2	Low-quality	9.61
Sample_C13C_k141_30358	9559	12	3	0	Low-quality	9.61
Sample_C8A_k141_65681	5298	14	1	0	Low-quality	9.61
Sample_T10A_k141_103079	14024	18	4	0	Low-quality	9.61
Sample_T2B_k141_104568	6037	11	2	1	Low-quality	9.61
Sample_C4C_k141_257160	6112	5	1	1	Low-quality	9.59
Sample_C8C_k141_36356	9111	4	0	2	Low-quality	9.59
Sample_T7C_k141_120013	9109	10	0	4	Low-quality	9.59
Sample_C7C_k141_1354	5922	12	0	0	Low-quality	9.59
Sample_T17C_k141_30473	8414	9	3	0	Low-quality	9.59
Sample_C4B_k141_240520	5737	8	0	4	Low-quality	9.58
Sample_C8C_k141_133788	12745	36	1	0	Low-quality	9.58
Sample_C9C_k141_79816	10375	8	0	2	Low-quality	9.58
Sample_T4B_k141_361740	28077	40	2	8	Low-quality	9.58
Sample_T7A_k141_70815	12898	15	1	0	Low-quality	9.58
Sample_C2A_k141_230134	15113	27	3	1	Low-quality	9.58
Sample_T10A_k141_32702	12810	22	1	1	Low-quality	9.58
Sample_T14A_k141_201944	5814	16	1	0	Low-quality	9.58
Sample_T14B_k141_7834	5282	7	1	1	Low-quality	9.58
Sample_T16C_k141_21773_fragment_2	5717	13	5	0	Low-quality	9.58
Sample_C6C_k141_92540	9182	9	0	8	Low-quality	9.57
Sample_T4B_k141_144535	14444	16	0	6	Low-quality	9.57
Sample_C9B_k141_98511	5796	15	1	0	Low-quality	9.57

Sample_T3A_k141_106966	6711	10	4	1	Low-quality	9.57
Sample_T6A_k141_92419	8608	4	0	1	Low-quality	9.56
Sample_C5A_k141_158444	5793	9	2	0	Low-quality	9.56
Sample_T14B_k141_98381	17616	20	1	7	Low-quality	9.55
Sample_T16C_k141_8950	5583	6	0	0	Low-quality	9.55
Sample_C12B_k141_166574	7881	6	0	3	Low-quality	9.54
Sample_C8A_k141_38777	5713	9	0	1	Low-quality	9.54
Sample_T13A_k141_103986	12738	37	0	0	Low-quality	9.54
Sample_T17C_k141_5619	9365	3	0	0	Low-quality	9.54
Sample_C4A_k141_12918	12389	15	1	1	Low-quality	9.54
Sample_C14C_k141_121455	5671	11	2	1	Low-quality	9.53
Sample_C6C_k141_189639	5862	4	1	0	Low-quality	9.53
Sample_T15A_k141_114975	10329	10	0	4	Low-quality	9.53
Sample_T3B_k141_76375	5706	8	0	0	Low-quality	9.53
Sample_C12A_k141_199268	5605	5	1	0	Low-quality	9.53
Sample_C4C_k141_40735	5142	6	3	0	Low-quality	9.53
Sample_T5B_k141_41187	8125	10	2	1	Low-quality	9.53
Sample_T11B_k141_95039	5659	5	1	1	Low-quality	9.52
Sample_T7A_k141_137613	8572	7	0	1	Low-quality	9.52
Sample_T12A_k141_254179	6356	14	0	0	Low-quality	9.52
Sample_T13B_k141_129858	5770	13	1	0	Low-quality	9.52
Sample_T6B_k141_106434	5545	9	1	1	Low-quality	9.52
Sample_C10A_k141_207624	6860	4	2	0	Low-quality	9.51
Sample_T2C_k141_232376	6144	15	3	0	Low-quality	9.51
Sample_C8C_k141_9368	6331	5	0	2	Low-quality	9.5
Sample_T17C_k141_28748	7673	11	1	7	Low-quality	9.5
Sample_T8A_k141_132646	6227	7	0	1	Low-quality	9.5
Sample_C8C_k141_77793	6399	10	3	0	Low-quality	9.5
Sample_C11A_k141_86275	10002	11	1	4	Low-quality	9.49
Sample_C5C_k141_22719	6079	11	1	4	Low-quality	9.49
Sample_T12C_k141_159943	5042	7	0	1	Low-quality	9.49
Sample_T12A_k141_22084	5277	4	0	0	Low-quality	9.49
Sample_T12C_k141_129798	10239	10	0	2	Low-quality	9.48
Sample_C1B_k141_32184	16516	26	3	0	Low-quality	9.48
Sample_T10B_k141_53348	5964	8	3	0	Low-quality	9.48
Sample_T14B_k141_129620	5554	7	1	0	Low-quality	9.48
Sample_C13B_k141_1492	9451	4	0	3	Low-quality	9.47
Sample_C7B_k141_101237	5584	7	0	1	Low-quality	9.47
Sample_T3C_k141_4393	8264	8	0	6	Low-quality	9.47

Sample_T7C_k141_156192	5888	3	1	0	Low-quality	9.47
Sample_C2C_k141_140027	10010	22	3	0	Low-quality	9.47
Sample_C6A_k141_178165	5329	6	2	0	Low-quality	9.47
Sample_C10A_k141_28229	9314	6	0	6	Low-quality	9.46
Sample_C8B_k141_75615	8689	11	0	3	Low-quality	9.46
Sample_T17B_k141_30810	13596	14	0	2	Low-quality	9.46
Sample_T18C_k141_26408	14996	14	1	9	Low-quality	9.46
Sample_C1C_k141_123738	8508	9	0	1	Low-quality	9.45
Sample_C8C_k141_142957	13595	17	1	4	Low-quality	9.45
Sample_T10A_k141_12854	7364	10	0	3	Low-quality	9.45
Sample_T14B_k141_128020	5656	10	0	0	Low-quality	9.45
Sample_T2C_k141_114572	6569	5	0	1	Low-quality	9.45
Sample_C2C_k141_94274	5720	6	2	0	Low-quality	9.45
Sample_C9A_k141_56765	10677	11	4	2	Low-quality	9.45
Sample_T8C_k141_48152	5658	12	0	0	Low-quality	9.45
Sample_C10A_k141_259274	8067	2	1	0	Low-quality	9.44
Sample_C8C_k141_22912	19366	21	0	3	Low-quality	9.44
Sample_C12A_k141_1733	5716	13	4	0	Low-quality	9.44
Sample_T6A_k141_38903	5045	8	7	1	Low-quality	9.44
Sample_C12A_k141_87213	8490	9	0	1	Low-quality	9.43
Sample_C2B_k141_1273	5480	5	0	0	Low-quality	9.43
Sample_T4C_k141_32797	5155	7	0	1	Low-quality	9.43
Sample_T5B_k141_8408	5647	5	0	0	Low-quality	9.43
Sample_T14B_k141_108909	5151	5	4	0	Low-quality	9.43
Sample_T16C_k141_14264	5672	10	2	0	Low-quality	9.43
Sample_T2B_k141_203015	5893	9	2	0	Low-quality	9.43
Sample_T4B_k141_106596	7274	16	2	1	Low-quality	9.43
Sample_C8A_k141_111071	7588	8	0	1	Low-quality	9.42
Sample_C9A_k141_57844	8129	8	0	0	Low-quality	9.42
Sample_T17A_k141_13717	18095	12	0	12	Low-quality	9.42
Sample_T12A_k141_233713	6045	6	0	0	Low-quality	9.42
Sample_T12C_k141_169484	15025	30	4	2	Low-quality	9.42
Sample_C3A_k141_186284	13268	34	1	0	Low-quality	9.41
Sample_C6A_k141_200958	5931	5	0	1	Low-quality	9.41
Sample_T10A_k141_113648	5630	10	0	1	Low-quality	9.4
Sample_T10C_k141_20846	8150	7	0	2	Low-quality	9.4
Sample_T16A_k141_84291	10058	9	0	2	Low-quality	9.4
Sample_T18B_k141_72443	10443	7	0	2	Low-quality	9.4
Sample_T4C_k141_13035	6210	5	1	0	Low-quality	9.4

Sample_T5B_k141_42449	5625	7	0	2	Low-quality	9.4
Sample_T13B_k141_139122	5099	6	1	0	Low-quality	9.4
Sample_T5C_k141_154410	10540	20	2	1	Low-quality	9.4
Sample_T6B_k141_140808	11716	12	1	0	Low-quality	9.4
Sample_C4B_k141_220423	5989	9	0	3	Low-quality	9.39
Sample_T12C_k141_126197	28629	22	2	5	Low-quality	9.39
Sample_C2A_k141_66633	5109	6	0	0	Low-quality	9.39
Sample_C8B_k141_158755	5581	12	2	0	Low-quality	9.39
Sample_T7C_k141_30410	7564	5	2	0	Low-quality	9.39
Sample_C12B_k141_184736	8232	7	0	0	Low-quality	9.38
Sample_T14A_k141_78120	5658	10	0	2	Low-quality	9.38
Sample_T1B_k141_11980	6901	4	0	2	Low-quality	9.38
Sample_T13B_k141_47354	5713	11	1	1	Low-quality	9.38
Sample_C3A_k141_133216	8283	7	0	3	Low-quality	9.37
Sample_T11C_k141_149497	19159	20	0	13	Low-quality	9.37
Sample_C13C_k141_78255_fragment_2	5689	10	1	1	Low-quality	9.37
Sample_T10B_k141_64883	7341	6	0	1	Low-quality	9.37
Sample_C14C_k141_178440	7653	6	0	4	Low-quality	9.36
Sample_T14B_k141_176547	5443	8	0	1	Low-quality	9.36
Sample_C5A_k141_198516	5917	14	2	0	Low-quality	9.36
Sample_T10C_k141_72894_fragment_1	10723	17	4	2	Low-quality	9.36
Sample_C10A_k141_8612	20845	33	3	8	Low-quality	9.35
Sample_T7A_k141_211563	13190	12	2	0	Low-quality	9.35
Sample_T5A_k141_29162	5018	5	1	0	Low-quality	9.35
Sample_C12C_k141_108114	12830	13	0	4	Low-quality	9.34
Sample_T17C_k141_24663	7635	6	0	2	Low-quality	9.34
Sample_C11B_k141_155260_fragment_1	8336	8	0	4	Low-quality	9.34
Sample_C14B_k141_67692	5826	9	1	0	Low-quality	9.34
Sample_C2A_k141_313356	5212	6	0	1	Low-quality	9.34
Sample_C7A_k141_147166	7689	12	4	1	Low-quality	9.34
Sample_C4B_k141_222072	7483	16	1	1	Low-quality	9.33
Sample_T16B_k141_163673	9307	7	0	6	Low-quality	9.33
Sample_C11A_k141_16147	6049	8	1	0	Low-quality	9.33
Sample_T17C_k141_13168	6599	5	0	4	Low-quality	9.32
Sample_C8B_k141_21123	5916	12	2	0	Low-quality	9.32
Sample_T10A_k141_116739	6657	9	2	0	Low-quality	9.32
Sample_T8A_k141_174520	5603	12	2	0	Low-quality	9.32
Sample_C8A_k141_139733	12913	13	1	2	Low-quality	9.3
Sample_T10A_k141_31056	9948	11	0	2	Low-quality	9.3

Sample_C3A_k141_229302	6289	4	1	0	Low-quality	9.3
Sample_T12C_k141_32835_fragment_1	10640	11	2	1	Low-quality	9.3
Sample_C4B_k141_82478	50846	34	1	24	Low-quality	9.29
Sample_C7B_k141_33638	7652	9	0	3	Low-quality	9.29
Sample_T16A_k141_109462	20031	32	2	5	Low-quality	9.29
Sample_T16B_k141_38140	5522	7	0	0	Low-quality	9.29
Sample_T2B_k141_201242	6999	13	4	1	Low-quality	9.29
Sample_T5A_k141_1889	7161	9	5	0	Low-quality	9.29
Sample_C1B_k141_141930	5558	9	0	5	Low-quality	9.28
Sample_C8B_k141_66753	5163	6	0	2	Low-quality	9.28
Sample_T16A_k141_45909	8161	14	0	1	Low-quality	9.28
Sample_T17B_k141_34351	9634	9	2	0	Low-quality	9.28
Sample_T6B_k141_83034	9515	9	0	3	Low-quality	9.28
Sample_T8A_k141_220100	5614	8	2	0	Low-quality	9.28
Sample_T4B_k141_93682	5096	11	0	0	Low-quality	9.27
Sample_T2B_k141_318815	5553	8	3	0	Low-quality	9.27
Sample_C10C_k141_105023	6310	9	2	0	Low-quality	9.26
Sample_T11C_k141_117233	11578	8	1	2	Low-quality	9.26
Sample_T14A_k141_6210	5122	5	0	3	Low-quality	9.26
Sample_C12A_k141_104002	24279	20	1	6	Low-quality	9.26
Sample_C2C_k141_62612	6032	9	3	0	Low-quality	9.26
Sample_T11C_k141_33389	6448	9	1	1	Low-quality	9.26
Sample_T18A_k141_177303	6048	8	0	0	Low-quality	9.26
Sample_T2C_k141_222653	5006	9	1	1	Low-quality	9.26
Sample_T5B_k141_22436	8515	16	1	1	Low-quality	9.26
Sample_C8B_k141_88130	9641	11	0	6	Low-quality	9.25
Sample_T11B_k141_42642	7017	2	0	1	Low-quality	9.25
Sample_T3C_k141_3148	7728	8	1	1	Low-quality	9.25
Sample_T6A_k141_17199	15452	15	1	2	Low-quality	9.24
Sample_C11B_k141_98534	8034	7	2	1	Low-quality	9.23
Sample_T2A_k141_186155	16679	15	0	10	Low-quality	9.23
Sample_T7B_k141_16596	9032	9	0	2	Low-quality	9.23
Sample_T1A_k141_10655	13458	6	1	1	Low-quality	9.22
Sample_C11C_k141_58593	7590	7	0	0	Low-quality	9.22
Sample_C4A_k141_16980	7219	15	4	0	Low-quality	9.22
Sample_C8B_k141_41361	6860	13	5	0	Low-quality	9.22
Sample_C10B_k141_67463	13447	13	1	10	Low-quality	9.21
Sample_C4B_k141_102546	50004	60	2	30	Low-quality	9.21
Sample_C6A_k141_198303	9817	7	0	6	Low-quality	9.21

Sample_C9A_k141_84059	6237	8	0	4	Low-quality	9.21
Sample_C9C_k141_95629	7586	12	0	1	Low-quality	9.21
Sample_T6A_k141_241203	8767	4	2	0	Low-quality	9.21
Sample_T7A_k141_220630	8291	10	0	1	Low-quality	9.21
Sample_T8B_k141_237348	15631	16	0	2	Low-quality	9.21
Sample_C3A_k141_247366	8308	6	0	1	Low-quality	9.2
Sample_C7B_k141_19454	9313	3	0	1	Low-quality	9.2
Sample_C9B_k141_50583	16685	28	3	1	Low-quality	9.2
Sample_T18A_k141_151180	6148	11	1	1	Low-quality	9.2
Sample_T4B_k141_55625	7009	13	2	0	Low-quality	9.2
Sample_C10A_k141_297254	7687	8	0	5	Low-quality	9.18
Sample_C10C_k141_34911	5524	6	0	1	Low-quality	9.18
Sample_C11B_k141_142777	5848	6	0	2	Low-quality	9.18
Sample_C2A_k141_174478	5326	9	1	0	Low-quality	9.18
Sample_T4B_k141_131072	33321	42	6	11	Low-quality	9.18
Sample_T11A_k141_88657	7059	8	1	0	Low-quality	9.18
Sample_C11A_k141_28273	5318	5	2	0	Low-quality	9.17
Sample_C4A_k141_244717	5723	10	2	0	Low-quality	9.16
Sample_C6B_k141_119904	6784	6	0	6	Low-quality	9.15
Sample_C4B_k141_139337	14637	18	5	1	Low-quality	9.15
Sample_T14B_k141_173812	12193	20	0	1	Low-quality	9.15
Sample_T16B_k141_98373	6723	11	3	0	Low-quality	9.15
Sample_T8C_k141_116765	10179	11	1	0	Low-quality	9.15
Sample_T12B_k141_54771	97220	86	2	47	Low-quality	9.14
Sample_C6B_k141_26453	18803	38	6	1	Low-quality	9.14
Sample_T11C_k141_8518	7529	7	0	1	Low-quality	9.14
Sample_T13B_k141_109379	5772	18	3	0	Low-quality	9.14
Sample_C4A_k141_72713	5581	7	0	0	Low-quality	9.13
Sample_C7B_k141_114249	18638	11	1	7	Low-quality	9.13
Sample_T17B_k141_19491	9473	8	4	0	Low-quality	9.13
Sample_C4C_k141_193780	5928	6	0	3	Low-quality	9.12
Sample_T1A_k141_623	9047	5	0	1	Low-quality	9.12
Sample_C11A_k141_38034	9354	9	1	2	Low-quality	9.12
Sample_C2B_k141_107879	7761	9	0	2	Low-quality	9.12
Sample_C9B_k141_76851	5942	7	0	1	Low-quality	9.11
Sample_T16A_k141_14541	18821	19	0	11	Low-quality	9.11
Sample_T4B_k141_50730	7826	10	0	1	Low-quality	9.11
Sample_T8B_k141_56708	35238	38	1	5	Low-quality	9.11
Sample_C14C_k141_94780	5545	18	0	0	Low-quality	9.11

Sample_T2A_k141_260030	15895	15	0	10	Low-quality	9.1
Sample_T6A_k141_280856	5019	4	0	1	Low-quality	9.1
Sample_T6C_k141_60027	17098	12	0	12	Low-quality	9.1
Sample_C13B_k141_85088	6084	11	1	0	Low-quality	9.1
Sample_T6C_k141_235133	5078	7	0	0	Low-quality	9.1
Sample_C10B_k141_149988	9132	8	0	5	Low-quality	9.09
Sample_C12C_k141_51675	5806	3	0	0	Low-quality	9.09
Sample_T6C_k141_1671	27920	31	1	3	Low-quality	9.09
Sample_C2A_k141_218216	5456	12	1	0	Low-quality	9.09
Sample_C3A_k141_15128	5555	14	0	0	Low-quality	9.09
Sample_C3C_k141_228095	5254	14	1	1	Low-quality	9.09
Sample_C8C_k141_110960	7699	4	0	1	Low-quality	9.09
Sample_T5C_k141_110416	10769	8	4	0	Low-quality	9.09
Sample_C7B_k141_171950	9412	10	3	2	Low-quality	9.08
Sample_C9B_k141_102913	18246	20	0	4	Low-quality	9.08
Sample_T8A_k141_164650	7455	10	3	3	Low-quality	9.08
Sample_T16B_k141_930	14266	19	1	1	Low-quality	9.07
Sample_T18A_k141_88043	12587	10	1	2	Low-quality	9.07
Sample_T4B_k141_343411	13077	15	1	4	Low-quality	9.07
Sample_T2B_k141_80828	8355	3	0	1	Low-quality	9.06
Sample_T3C_k141_118002	9227	13	0	0	Low-quality	9.06
Sample_T8B_k141_30098	17865	21	1	8	Low-quality	9.06
Sample_T3B_k141_115654	5806	6	1	0	Low-quality	9.06
Sample_T6A_k141_265735_fragment_1	6243	8	2	0	Low-quality	9.06
Sample_T13C_k141_105270	5758	8	0	3	Low-quality	9.05
Sample_C2B_k141_279337	5980	5	1	0	Low-quality	9.05
Sample_T5C_k141_26007_fragment_1	10801	14	5	1	Low-quality	9.05
Sample_T12B_k141_165820	12545	11	2	0	Low-quality	9.04
Sample_C3C_k141_119836	5770	11	1	0	Low-quality	9.04
Sample_C3C_k141_32764	5680	6	6	0	Low-quality	9.04
Sample_T10B_k141_14767	6928	11	3	0	Low-quality	9.04
Sample_T17B_k141_28276	8258	3	0	0	Low-quality	9.03
Sample_C3B_k141_271114_fragment_1	7621	8	1	1	Low-quality	9.03
Sample_C9C_k141_101034	5901	9	2	0	Low-quality	9.03
Sample_T12B_k141_64780	5184	6	0	0	Low-quality	9.03
Sample_C2C_k141_46792	9174	8	0	7	Low-quality	9.02
Sample_C5A_k141_283409	13058	9	1	0	Low-quality	9.02
Sample_C8B_k141_7161	5242	8	0	0	Low-quality	9.02
Sample_T15B_k141_102745	17813	22	0	7	Low-quality	9.02

Sample_T10A_k141_88644	5901	11	2	0	Low-quality	9.02
Sample_T11C_k141_13539	5832	6	1	1	Low-quality	9.02
Sample_T12C_k141_179177	6938	14	0	1	Low-quality	9.02
Sample_T17B_k141_2590	9437	8	0	0	Low-quality	9.02
Sample_T3B_k141_63133	5958	4	1	0	Low-quality	9.02
Sample_C14C_k141_64992	16202	15	1	10	Low-quality	9.01
Sample_T12C_k141_146600	7138	13	0	0	Low-quality	9.01
Sample_C10A_k141_306813	5721	3	1	0	Low-quality	9
Sample_C3B_k141_1366	10400	5	1	0	Low-quality	9
Sample_T12C_k141_92676	6356	5	0	0	Low-quality	9
Sample_C4B_k141_123916	5759	6	0	1	Low-quality	9
Sample_C10A_k141_205288	7050	15	1	2	Low-quality	8.99
Sample_T8B_k141_110361	5383	5	0	1	Low-quality	8.99
Sample_T12B_k141_140111	14515	31	2	0	Low-quality	8.99
Sample_T13C_k141_5600	9241	4	0	0	Low-quality	8.99
Sample_C2C_k141_262169	5451	11	3	0	Low-quality	8.98
Sample_T15B_k141_19233	11253	26	5	0	Low-quality	8.98
Sample_C9C_k141_101018	5926	3	1	0	Low-quality	8.97
Sample_T6B_k141_23494	5368	10	0	0	Low-quality	8.97
Sample_T16C_k141_26082	5175	7	1	0	Low-quality	8.97
Sample_C12C_k141_133691	5919	4	0	1	Low-quality	8.96
Sample_T18C_k141_34503	17920	15	0	6	Low-quality	8.96
Sample_C1A_k141_152927	5598	11	3	0	Low-quality	8.96
Sample_T3B_k141_141450	5288	6	2	0	Low-quality	8.96
Sample_C10B_k141_84859	5360	9	0	2	Low-quality	8.95
Sample_C4B_k141_106332	16507	14	1	5	Low-quality	8.95
Sample_C7B_k141_29420	6558	2	0	0	Low-quality	8.95
Sample_T1B_k141_35823	15630	10	0	6	Low-quality	8.95
Sample_T6B_k141_34210	5357	4	0	0	Low-quality	8.95
Sample_C10A_k141_108036	5741	5	2	0	Low-quality	8.95
Sample_T12C_k141_40244	6862	13	2	0	Low-quality	8.95
Sample_T4B_k141_241145	5382	4	3	0	Low-quality	8.95
Sample_T7B_k141_28598_fragment_1	7779	13	1	0	Low-quality	8.95
Sample_T15C_k141_138217	7542	12	1	1	Low-quality	8.94
Sample_T6A_k141_281865	14173	17	1	9	Low-quality	8.94
Sample_T8B_k141_14163	7358	7	0	1	Low-quality	8.94
Sample_C3A_k141_158466	5454	7	4	0	Low-quality	8.94
Sample_T11A_k141_64904	6234	12	9	1	Low-quality	8.94
Sample_T2B_k141_202627	5739	7	0	1	Low-quality	8.94

Sample_T6B_k141_46306	10216	12	2	1	Low-quality	8.94
Sample_C10A_k141_292779	5728	8	3	0	Low-quality	8.93
Sample_C5C_k141_2815	13804	16	1	1	Low-quality	8.93
Sample_C9C_k141_16077	6092	11	7	0	Low-quality	8.93
Sample_T11C_k141_120014_fragment_1	9851	8	0	2	Low-quality	8.93
Sample_C6C_k141_238149	8136	12	2	3	Low-quality	8.92
Sample_T15A_k141_11300	17970	18	0	10	Low-quality	8.92
Sample_C13C_k141_28268	6673	11	2	0	Low-quality	8.92
Sample_C1B_k141_222937	5617	8	2	2	Low-quality	8.92
Sample_C13A_k141_32918	8018	8	0	0	Low-quality	8.91
Sample_C3C_k141_196058	16929	13	2	3	Low-quality	8.91
Sample_T7C_k141_33935	5338	5	3	0	Low-quality	8.91
Sample_C7A_k141_111951	7287	13	0	0	Low-quality	8.9
Sample_C8A_k141_8913	19254	19	1	6	Low-quality	8.9
Sample_T13C_k141_67262	5853	6	1	0	Low-quality	8.9
Sample_T4B_k141_4856	7807	7	0	1	Low-quality	8.9
Sample_C2A_k141_308344	14907	15	0	5	Low-quality	8.89
Sample_C6B_k141_129356	30186	30	1	12	Low-quality	8.89
Sample_T16B_k141_172706	7377	8	1	2	Low-quality	8.89
Sample_T17B_k141_13475	18811	14	0	14	Low-quality	8.89
Sample_T18A_k141_109428	10161	11	0	7	Low-quality	8.89
Sample_C7C_k141_77498	11237	12	3	0	Low-quality	8.88
Sample_C8B_k141_181588	5210	2	0	1	Low-quality	8.88
Sample_T10C_k141_20315	8907	9	0	6	Low-quality	8.88
Sample_T14A_k141_121286	10071	10	1	2	Low-quality	8.88
Sample_T6C_k141_177372	7572	6	0	0	Low-quality	8.88
Sample_C10B_k141_51946	7322	6	0	2	Low-quality	8.88
Sample_T3A_k141_86369	7307	5	2	0	Low-quality	8.88
Sample_T7A_k141_133681	5503	4	3	0	Low-quality	8.88
Sample_T8B_k141_174190	14284	20	4	1	Low-quality	8.88
Sample_T13C_k141_33885	8000	17	2	2	Low-quality	8.87
Sample_T7C_k141_75199	5368	10	1	0	Low-quality	8.87
Sample_T8B_k141_75868	6799	7	1	0	Low-quality	8.87
Sample_C10A_k141_299120	6107	10	0	0	Low-quality	8.87
Sample_C11B_k141_216022	7078	16	1	0	Low-quality	8.87
Sample_T14A_k141_120740	6175	13	1	0	Low-quality	8.87
Sample_C4B_k141_216126	5279	4	1	0	Low-quality	8.86
Sample_C7C_k141_12300	5037	5	0	0	Low-quality	8.86
Sample_T3A_k141_66783	5910	6	3	1	Low-quality	8.86

Sample_C10A_k141_23319	7972	10	1	0	Low-quality	8.86
Sample_C5A_k141_149957	10971	10	0	3	Low-quality	8.85
Sample_T14C_k141_112575	5736	5	0	0	Low-quality	8.85
Sample_T18C_k141_115834	15443	16	0	15	Low-quality	8.85
Sample_T8B_k141_117205	14131	11	1	1	Low-quality	8.85
Sample_T8A_k141_105945	5051	9	1	0	Low-quality	8.85
Sample_T3C_k141_6315	5081	7	2	1	Low-quality	8.84
Sample_C3A_k141_209113	9009	4	3	1	Low-quality	8.84
Sample_C3B_k141_177050	5079	14	1	0	Low-quality	8.84
Sample_C5B_k141_74362	6345	9	1	1	Low-quality	8.83
Sample_C6B_k141_27350	5808	8	0	3	Low-quality	8.82
Sample_T14A_k141_226120	31008	18	1	10	Low-quality	8.82
Sample_C1C_k141_64860	5014	11	1	0	Low-quality	8.82
Sample_T10A_k141_139950	5169	8	1	1	Low-quality	8.82
Sample_T1C_k141_16562	10874	10	0	7	Low-quality	8.81
Sample_T3B_k141_75144	8022	9	0	1	Low-quality	8.81
Sample_C7C_k141_104824	15538	29	1	0	Low-quality	8.81
Sample_T13B_k141_133814	12878	17	1	2	Low-quality	8.81
Sample_C10A_k141_7832	8196	12	2	3	Low-quality	8.8
Sample_T10A_k141_108223	6560	9	3	0	Low-quality	8.8
Sample_T15A_k141_87145	8140	4	0	2	Low-quality	8.79
Sample_C11A_k141_20707	15204	29	2	0	Low-quality	8.79
Sample_C11A_k141_171521	18580	14	0	14	Low-quality	8.78
Sample_C11A_k141_4510	7521	7	0	3	Low-quality	8.78
Sample_T13A_k141_16093	6422	8	1	1	Low-quality	8.78
Sample_T12A_k141_292868	6584	6	0	1	Low-quality	8.78
Sample_T15B_k141_48567	5215	9	2	1	Low-quality	8.78
Sample_T2A_k141_83045	5233	6	0	0	Low-quality	8.78
Sample_C4A_k141_23964	14002	11	1	4	Low-quality	8.77
Sample_T17A_k141_13855	24664	34	2	8	Low-quality	8.77
Sample_T6C_k141_174440	16929	17	0	14	Low-quality	8.77
Sample_T10C_k141_88437	11623	17	1	1	Low-quality	8.77
Sample_T13B_k141_154195	16800	18	0	11	Low-quality	8.76
Sample_T13B_k141_160143	8137	8	0	0	Low-quality	8.76
Sample_T8C_k141_152216	5232	6	0	0	Low-quality	8.76
Sample_C1A_k141_164238	10215	13	0	1	Low-quality	8.75
Sample_C8B_k141_12953	14191	16	1	1	Low-quality	8.75
Sample_T10A_k141_168288	5835	5	0	2	Low-quality	8.75
Sample_T13B_k141_150466	17054	14	0	10	Low-quality	8.75

Sample_T13C_k141_130072	7199	9	1	1	Low-quality	8.75
Sample_C11A_k141_56783	7151	5	1	1	Low-quality	8.75
Sample_C11A_k141_93982	6943	6	1	1	Low-quality	8.75
Sample_T14A_k141_149749	5778	4	1	0	Low-quality	8.74
Sample_T14B_k141_194170	6422	5	0	1	Low-quality	8.74
Sample_T16B_k141_53597	7841	10	0	1	Low-quality	8.74
Sample_T8A_k141_118392	17737	16	0	15	Low-quality	8.74
Sample_C10A_k141_121551	13349	18	4	1	Low-quality	8.74
Sample_T8B_k141_198717	6745	12	2	0	Low-quality	8.74
Sample_C12A_k141_11787	7135	6	0	1	Low-quality	8.73
Sample_T13C_k141_10602	5624	5	3	0	Low-quality	8.73
Sample_T7B_k141_89106	10759	7	4	0	Low-quality	8.73
Sample_C13A_k141_43371	8966	14	0	1	Low-quality	8.72
Sample_C6B_k141_51858	6323	6	0	1	Low-quality	8.72
Sample_T16C_k141_30626	13148	12	0	6	Low-quality	8.72
Sample_T18B_k141_60669	6267	4	0	1	Low-quality	8.72
Sample_T2A_k141_93902	5664	8	0	5	Low-quality	8.72
Sample_C8B_k141_200563	8307	16	0	0	Low-quality	8.71
Sample_T8C_k141_58353	5662	8	5	0	Low-quality	8.71
Sample_C10A_k141_201710	5316	5	0	2	Low-quality	8.7
Sample_T15C_k141_34145	5211	7	0	3	Low-quality	8.7
Sample_T15C_k141_145847	5927	6	0	1	Low-quality	8.7
Sample_T8C_k141_34331	6082	7	0	0	Low-quality	8.7
Sample_C5B_k141_57904	6356	8	2	0	Low-quality	8.7
Sample_T15A_k141_181038	7577	9	5	0	Low-quality	8.7
Sample_T3B_k141_125199	6585	13	4	0	Low-quality	8.7
Sample_T3B_k141_32922	7806	8	0	0	Low-quality	8.7
Sample_T5C_k141_185756	17495	15	0	10	Low-quality	8.69
Sample_T6C_k141_237040	5202	9	0	0	Low-quality	8.69
Sample_C3B_k141_138127	7820	7	5	0	Low-quality	8.69
Sample_T5C_k141_176355	5285	11	0	0	Low-quality	8.69
Sample_T11B_k141_44898	8232	7	0	1	Low-quality	8.68
Sample_T1A_k141_43605	5547	6	0	3	Low-quality	8.68
Sample_C10A_k141_331464	8033	11	5	0	Low-quality	8.68
Sample_C9A_k141_9136	16787	41	1	0	Low-quality	8.68
Sample_T18B_k141_96264	5301	5	4	0	Low-quality	8.68
Sample_T6C_k141_50760	7806	6	0	2	Low-quality	8.67
Sample_C12A_k141_146695	5489	6	1	0	Low-quality	8.66
Sample_T4B_k141_373638	5034	6	0	0	Low-quality	8.66

Sample_T6A_k141_255933	5911	9	0	0	Low-quality	8.66
Sample_C12C_k141_19865	7630	10	0	1	Low-quality	8.66
Sample_C7C_k141_85872	15218	32	8	1	Low-quality	8.66
Sample_C9A_k141_93442	18675	40	5	1	Low-quality	8.66
Sample_T3B_k141_179430	7505	6	0	2	Low-quality	8.66
Sample_C7A_k141_10395	6986	11	1	4	Low-quality	8.65
Sample_T2C_k141_23562	5184	5	0	1	Low-quality	8.65
Sample_T4B_k141_173859	7894	11	0	2	Low-quality	8.65
Sample_C8B_k141_39790	5716	4	1	0	Low-quality	8.65
Sample_T2B_k141_154751	7118	7	2	0	Low-quality	8.65
Sample_C13B_k141_85765	17098	15	2	2	Low-quality	8.64
Sample_C10A_k141_166757	5590	8	0	0	Low-quality	8.64
Sample_C14C_k141_72702	5240	9	0	0	Low-quality	8.64
Sample_C9A_k141_94829	8870	6	0	3	Low-quality	8.64
Sample_C6B_k141_35619	17280	17	0	12	Low-quality	8.63
Sample_T16B_k141_96697	6379	6	3	1	Low-quality	8.63
Sample_T18C_k141_7235	9324	11	0	3	Low-quality	8.63
Sample_C8A_k141_172273	6376	6	0	1	Low-quality	8.63
Sample_T3C_k141_21324	8893	10	0	1	Low-quality	8.63
Sample_T13C_k141_89493	15901	23	1	3	Low-quality	8.62
Sample_T7A_k141_209631	5386	5	0	2	Low-quality	8.62
Sample_C4B_k141_245582	7098	5	3	0	Low-quality	8.62
Sample_T4C_k141_117375	17208	17	0	5	Low-quality	8.61
Sample_C2A_k141_2031	12377	18	3	2	Low-quality	8.61
Sample_T4A_k141_101352	8746	13	0	2	Low-quality	8.6
Sample_C4C_k141_122204	7110	4	0	2	Low-quality	8.59
Sample_C7B_k141_178128	6575	2	0	0	Low-quality	8.59
Sample_T15B_k141_95308	13279	18	1	4	Low-quality	8.59
Sample_T3A_k141_131847	7017	6	0	0	Low-quality	8.59
Sample_T17B_k141_12512	5338	9	5	2	Low-quality	8.59
Sample_T4C_k141_70551	5130	14	2	0	Low-quality	8.58
Sample_C10C_k141_39768	5847	6	0	1	Low-quality	8.57
Sample_C2C_k141_28891	7684	8	0	7	Low-quality	8.57
Sample_C3B_k141_61217	10707	16	2	3	Low-quality	8.57
Sample_C6C_k141_235023	8212	7	0	0	Low-quality	8.57
Sample_C4A_k141_162070	12417	18	3	0	Low-quality	8.57
Sample_T10A_k141_93682	6925	12	4	1	Low-quality	8.57
Sample_T16A_k141_71724	5174	8	4	0	Low-quality	8.57
Sample_T2B_k141_131348	27152	25	0	17	Low-quality	8.56

Sample_T2C_k141_118712	14107	21	2	1	Low-quality	8.56
Sample_T6A_k141_226212	10558	11	0	6	Low-quality	8.56
Sample_C2C_k141_52457	5277	6	0	1	Low-quality	8.56
Sample_C4C_k141_7104	5164	8	0	0	Low-quality	8.56
Sample_C9C_k141_98817	6543	16	3	0	Low-quality	8.56
Sample_T14B_k141_4021	10325	10	0	0	Low-quality	8.56
Sample_T16B_k141_71420	6146	6	1	1	Low-quality	8.56
Sample_C1B_k141_207166	5357	7	1	1	Low-quality	8.55
Sample_C1C_k141_19369	5229	5	0	1	Low-quality	8.55
Sample_T8A_k141_79855	5779	8	0	1	Low-quality	8.55
Sample_C14A_k141_167324	7087	7	2	0	Low-quality	8.55
Sample_T7A_k141_181044	5137	11	3	0	Low-quality	8.55
Sample_T8C_k141_160762	5558	5	0	1	Low-quality	8.55
Sample_T4B_k141_22317	17349	15	0	12	Low-quality	8.54
Sample_T5C_k141_19830	5641	4	1	0	Low-quality	8.54
Sample_T2B_k141_293479_fragment_2	12707	13	1	2	Low-quality	8.54
Sample_T11B_k141_78321	23948	23	1	10	Low-quality	8.53
Sample_T4B_k141_342552	12982	16	0	10	Low-quality	8.53
Sample_C2B_k141_221394	5171	8	1	0	Low-quality	8.53
Sample_C1A_k141_183893	5112	4	0	2	Low-quality	8.52
Sample_C4A_k141_25008	5006	3	2	0	Low-quality	8.52
Sample_T17C_k141_1635	7593	8	0	2	Low-quality	8.52
Sample_T18C_k141_173	7082	6	1	1	Low-quality	8.52
Sample_C4C_k141_15444	12273	5	3	0	Low-quality	8.52
Sample_T18A_k141_192387	5101	4	0	0	Low-quality	8.52
Sample_T11C_k141_2220	11421	8	0	1	Low-quality	8.51
Sample_T12C_k141_166859	6226	5	0	2	Low-quality	8.51
Sample_T1A_k141_6655	15683	22	1	1	Low-quality	8.51
Sample_C9A_k141_11736	15161	25	2	0	Low-quality	8.51
Sample_T10A_k141_99607	14559	29	1	1	Low-quality	8.51
Sample_T14A_k141_158293	19576	22	3	4	Low-quality	8.5
Sample_C8B_k141_81341	5345	12	4	0	Low-quality	8.5
Sample_C9C_k141_58896	20913	22	1	4	Low-quality	8.5
Sample_C3B_k141_58293	15667	12	0	9	Low-quality	8.49
Sample_C5A_k141_213084	46487	39	1	32	Low-quality	8.49
Sample_T11A_k141_20233	13503	20	2	0	Low-quality	8.49
Sample_T8C_k141_26079	5082	7	0	0	Low-quality	8.49
Sample_C2C_k141_302734	8973	11	4	0	Low-quality	8.49
Sample_C4B_k141_219158	5205	7	5	0	Low-quality	8.49

Sample_T12B_k141_135570	5263	7	7	0	Low-quality	8.49
Sample_T6C_k141_268882	5168	5	2	1	Low-quality	8.49
Sample_C5C_k141_16832	23810	20	1	6	Low-quality	8.48
Sample_C8B_k141_16798	5303	3	0	1	Low-quality	8.48
Sample_T17B_k141_13037	11307	12	0	3	Low-quality	8.48
Sample_T8A_k141_109889	5612	8	2	0	Low-quality	8.48
Sample_T8B_k141_22591	6049	13	2	0	Low-quality	8.48
Sample_C2A_k141_290688	6681	10	0	1	Low-quality	8.47
Sample_C3B_k141_179438	8562	7	0	6	Low-quality	8.47
Sample_T16B_k141_30710	13165	14	1	7	Low-quality	8.47
Sample_T2B_k141_148875	7888	8	0	5	Low-quality	8.47
Sample_C2A_k141_129222	10927	12	3	0	Low-quality	8.47
Sample_C7A_k141_48527	5029	6	0	2	Low-quality	8.46
Sample_T14A_k141_150501	5221	5	3	0	Low-quality	8.46
Sample_C13A_k141_46223	16540	15	0	7	Low-quality	8.45
Sample_C13B_k141_10641	8628	3	0	3	Low-quality	8.45
Sample_C1C_k141_55249	14190	13	1	10	Low-quality	8.44
Sample_T3B_k141_57241	14963	12	0	12	Low-quality	8.44
Sample_C12A_k141_68090	6741	9	0	2	Low-quality	8.44
Sample_C7A_k141_100230_fragment_3	13046	11	1	0	Low-quality	8.44
Sample_T11C_k141_98723	5579	5	1	0	Low-quality	8.44
Sample_T13B_k141_17416	13937	15	2	1	Low-quality	8.44
Sample_C7C_k141_11781	5050	6	0	0	Low-quality	8.43
Sample_C11C_k141_12061	9901	13	1	0	Low-quality	8.43
Sample_T11A_k141_70207	7911	13	0	1	Low-quality	8.42
Sample_C1C_k141_106163	6101	5	4	0	Low-quality	8.42
Sample_T3C_k141_136429	5009	6	0	1	Low-quality	8.42
Sample_C10A_k141_263461	5555	6	1	0	Low-quality	8.41
Sample_C10A_k141_263492	5555	6	1	0	Low-quality	8.41
Sample_C2B_k141_106528	19868	23	1	17	Low-quality	8.41
Sample_C6C_k141_122984	9090	9	0	6	Low-quality	8.41
Sample_T10A_k141_23526	7896	8	0	1	Low-quality	8.41
Sample_T2C_k141_218929	15073	17	1	2	Low-quality	8.41
Sample_C7C_k141_21403	5273	15	1	0	Low-quality	8.41
Sample_T7A_k141_14810	5253	6	0	0	Low-quality	8.41
Sample_C10C_k141_107874	5439	6	0	1	Low-quality	8.4
Sample_C3A_k141_35793	12503	12	1	3	Low-quality	8.4
Sample_C4C_k141_197054	11252	14	1	0	Low-quality	8.4
Sample_C4B_k141_18137	5182	15	2	0	Low-quality	8.4

Sample_T15C_k141_84017_fragment_1	12490	16	1	0	Low-quality	8.4
Sample_C12C_k141_40617	8811	9	0	1	Low-quality	8.39
Sample_T17A_k141_27235	5016	6	0	0	Low-quality	8.38
Sample_T8B_k141_86582	23526	21	1	3	Low-quality	8.38
Sample_C10C_k141_128493	7708	7	0	1	Low-quality	8.37
Sample_C5A_k141_248137	5747	7	0	4	Low-quality	8.37
Sample_T1B_k141_26780	13023	12	0	4	Low-quality	8.37
Sample_T4B_k141_94946	12167	10	0	6	Low-quality	8.37
Sample_C4B_k141_73109	5347	6	1	0	Low-quality	8.37
Sample_T18B_k141_17474	6139	10	2	0	Low-quality	8.37
Sample_C5A_k141_83705	9031	6	0	2	Low-quality	8.36
Sample_T15B_k141_1119	14981	11	1	2	Low-quality	8.36
Sample_T7A_k141_127718	8354	4	0	0	Low-quality	8.36
Sample_C10C_k141_71544	5053	10	5	0	Low-quality	8.36
Sample_C4C_k141_189732	5133	12	2	0	Low-quality	8.36
Sample_T15A_k141_128747	14700	27	0	1	Low-quality	8.36
Sample_T18C_k141_33277	10653	4	0	2	Low-quality	8.35
Sample_T10B_k141_24411	8828	6	0	5	Low-quality	8.33
Sample_T11A_k141_140597	20087	7	0	1	Low-quality	8.33
Sample_T11B_k141_97475	6619	7	0	2	Low-quality	8.33
Sample_T6C_k141_86740	14968	20	2	4	Low-quality	8.33
Sample_C12C_k141_140873	12454	18	2	2	Low-quality	8.33
Sample_C6C_k141_159739	7027	12	1	1	Low-quality	8.33
Sample_T14A_k141_125638	5141	8	6	0	Low-quality	8.33
Sample_T5A_k141_21291	8254	10	3	0	Low-quality	8.33
Sample_T8B_k141_83321	5226	9	5	0	Low-quality	8.33
Sample_C2B_k141_262211	6705	4	0	0	Low-quality	8.32
Sample_C7A_k141_68409	15385	15	0	8	Low-quality	8.32
Sample_C8C_k141_106212	12776	13	0	2	Low-quality	8.32
Sample_T8A_k141_222110	18480	19	0	11	Low-quality	8.32
Sample_C13A_k141_12642	13340	11	0	0	Low-quality	8.32
Sample_T18C_k141_149179	7422	13	0	0	Low-quality	8.32
Sample_T10C_k141_81264	6718	8	1	3	Low-quality	8.31
Sample_T15C_k141_65960	14222	14	1	3	Low-quality	8.31
Sample_T1A_k141_12609	10987	12	0	6	Low-quality	8.31
Sample_T2A_k141_127212	6179	10	0	0	Low-quality	8.31
Sample_T6A_k141_28762	10854	11	0	1	Low-quality	8.31
Sample_C12B_k141_81744	5765	6	1	1	Low-quality	8.31
Sample_C14A_k141_138562	5085	7	3	0	Low-quality	8.31

Sample_T10C_k141_24312	11074	16	1	0	Low-quality	8.31
Sample_C2C_k141_175987	6786	8	0	2	Low-quality	8.3
Sample_C3B_k141_34617	8967	9	0	2	Low-quality	8.3
Sample_C5A_k141_190276	5486	11	1	0	Low-quality	8.3
Sample_T8A_k141_144721	5106	12	1	0	Low-quality	8.29
Sample_C1B_k141_19613	8441	11	1	0	Low-quality	8.29
Sample_C3A_k141_155460	7301	15	6	0	Low-quality	8.29
Sample_C9A_k141_69445	5553	12	5	0	Low-quality	8.29
Sample_C11A_k141_75010	5058	6	2	0	Low-quality	8.28
Sample_C6B_k141_98391	6096	4	0	2	Low-quality	8.28
Sample_T10B_k141_141346	5426	12	0	0	Low-quality	8.28
Sample_C5B_k141_63669	5197	10	6	0	Low-quality	8.28
Sample_T12B_k141_156462	13183	37	1	0	Low-quality	8.28
Sample_T8B_k141_194816	5365	5	1	1	Low-quality	8.28
Sample_T14B_k141_114735	13850	16	1	10	Low-quality	8.27
Sample_T15A_k141_56481	5427	5	0	2	Low-quality	8.27
Sample_T12B_k141_18340	12630	12	1	0	Low-quality	8.27
Sample_T7A_k141_5907_fragment_1	13068	14	2	2	Low-quality	8.27
Sample_C12C_k141_199639	11791	9	1	0	Low-quality	8.26
Sample_C3B_k141_261387	9774	10	0	2	Low-quality	8.26
Sample_C9A_k141_109854	12047	10	0	7	Low-quality	8.26
Sample_T5C_k141_30104	12292	13	1	0	Low-quality	8.26
Sample_T13B_k141_125565	7991	8	0	2	Low-quality	8.25
Sample_T11B_k141_33689	5295	5	1	0	Low-quality	8.25
Sample_C2A_k141_203628	5527	11	2	0	Low-quality	8.24
Sample_C12B_k141_30452	5290	5	0	1	Low-quality	8.23
Sample_C3A_k141_259455	29062	39	1	7	Low-quality	8.23
Sample_T10A_k141_111211	14363	14	0	11	Low-quality	8.23
Sample_T10C_k141_3842	17207	21	4	11	Low-quality	8.23
Sample_T12C_k141_37617	13124	18	2	10	Low-quality	8.23
Sample_C11C_k141_73804	7172	8	2	0	Low-quality	8.23
Sample_C2C_k141_113432	13047	11	4	0	Low-quality	8.23
Sample_T14A_k141_60261	5358	13	1	0	Low-quality	8.23
Sample_T3A_k141_44350	5093	9	1	0	Low-quality	8.23
Sample_C12C_k141_81038	5054	10	1	0	Low-quality	8.22
Sample_C10A_k141_87332	5261	3	0	0	Low-quality	8.21
Sample_T12A_k141_116293	10197	6	0	6	Low-quality	8.21
Sample_C10C_k141_59481	5527	9	2	0	Low-quality	8.21
Sample_T4C_k141_38333	6874	5	1	1	Low-quality	8.21

Sample_C9B_k141_25509	8477	6	0	3	Low-quality	8.2
Sample_T2C_k141_39262	5888	5	0	0	Low-quality	8.2
Sample_T13B_k141_129233	8611	11	0	3	Low-quality	8.19
Sample_T12A_k141_204056	6585	11	3	0	Low-quality	8.19
Sample_T18A_k141_112245	7686	11	0	2	Low-quality	8.18
Sample_C1C_k141_36289	9037	18	1	0	Low-quality	8.18
Sample_C9A_k141_13645	5336	8	1	1	Low-quality	8.18
Sample_T5B_k141_60165	16188	15	3	6	Low-quality	8.17
Sample_T12B_k141_54776_fragment_1	9943	9	0	1	Low-quality	8.17
Sample_T2B_k141_114784	12476	16	2	0	Low-quality	8.17
Sample_C3B_k141_94315	16651	16	1	12	Low-quality	8.16
Sample_T8B_k141_154844	5144	8	1	0	Low-quality	8.16
Sample_C3C_k141_65863	20051	23	1	2	Low-quality	8.15
Sample_C4A_k141_110591	6385	3	0	2	Low-quality	8.15
Sample_T6A_k141_185970	7825	8	0	0	Low-quality	8.15
Sample_C4C_k141_196580	5551	8	1	0	Low-quality	8.15
Sample_C7C_k141_55736	8771	4	0	0	Low-quality	8.15
Sample_T7A_k141_15840_fragment_1	6586	10	1	0	Low-quality	8.15
Sample_C3C_k141_205715	11745	9	0	7	Low-quality	8.14
Sample_T2A_k141_24205	14219	17	0	13	Low-quality	8.14
Sample_C1B_k141_208153	14116	15	6	1	Low-quality	8.14
Sample_C1C_k141_52777	11333	9	0	0	Low-quality	8.14
Sample_C4B_k141_186454	6968	12	3	0	Low-quality	8.14
Sample_C4B_k141_158038	5506	8	3	1	Low-quality	8.14
Sample_T4B_k141_285130	6403	9	6	0	Low-quality	8.14
Sample_C11C_k141_82781	7314	5	0	0	Low-quality	8.13
Sample_T14A_k141_57215	21805	18	2	4	Low-quality	8.13
Sample_T4B_k141_177623	16725	18	0	11	Low-quality	8.13
Sample_C8B_k141_148506	5886	4	0	1	Low-quality	8.12
Sample_T10A_k141_59703	20160	17	1	9	Low-quality	8.12
Sample_T12B_k141_145642	10287	13	1	0	Low-quality	8.12
Sample_T14B_k141_67269	6930	8	0	2	Low-quality	8.12
Sample_T10A_k141_153470	6267	7	2	0	Low-quality	8.12
Sample_T8B_k141_64638	5364	6	1	0	Low-quality	8.12
Sample_C3C_k141_257348	5269	10	0	0	Low-quality	8.11
Sample_T14A_k141_181059	8103	10	0	1	Low-quality	8.11
Sample_T17A_k141_15621	8208	9	0	2	Low-quality	8.11
Sample_T12C_k141_193338	13527	17	5	0	Low-quality	8.11
Sample_T14A_k141_237847	7227	7	1	0	Low-quality	8.11

Sample_T5C_k141_153867	5233	9	2	0	Low-quality	8.11
Sample_C9C_k141_92756	6381	7	3	0	Low-quality	8.1
Sample_T13A_k141_59892	8811	10	0	2	Low-quality	8.1
Sample_T14A_k141_127158	5283	13	2	0	Low-quality	8.1
Sample_T8C_k141_61879	6445	11	4	0	Low-quality	8.1
Sample_T10A_k141_145232	5007	2	1	0	Low-quality	8.09
Sample_C12C_k141_44371	7628	11	1	0	Low-quality	8.09
Sample_C9A_k141_97258	5317	6	3	0	Low-quality	8.09
Sample_T15B_k141_94586	7010	15	1	0	Low-quality	8.09
Sample_T6A_k141_37631	12404	22	0	1	Low-quality	8.09
Sample_T2C_k141_169200	8842	12	0	0	Low-quality	8.08
Sample_T11C_k141_30217_fragment_1	14902	19	3	0	Low-quality	8.08
Sample_T11C_k141_134623	6656	12	5	0	Low-quality	8.08
Sample_T16C_k141_95543	5338	5	1	0	Low-quality	8.08
Sample_T4B_k141_68831	6350	11	1	0	Low-quality	8.08
Sample_C6A_k141_170876	5899	9	2	1	Low-quality	8.07
Sample_C6B_k141_93648	5334	7	1	0	Low-quality	8.07
Sample_T16C_k141_120889	14304	15	0	2	Low-quality	8.07
Sample_C10B_k141_21688	5152	10	3	1	Low-quality	8.07
Sample_C1A_k141_245960	6594	9	0	0	Low-quality	8.07
Sample_T1A_k141_18883	11182	19	1	5	Low-quality	8.06
Sample_T4B_k141_242811	6320	10	0	0	Low-quality	8.06
Sample_C11A_k141_58104	9503	13	0	2	Low-quality	8.06
Sample_C2C_k141_151011	7112	4	1	0	Low-quality	8.06
Sample_C3A_k141_50538	9220	16	2	0	Low-quality	8.05
Sample_T6B_k141_186943	7233	4	0	1	Low-quality	8.04
Sample_C3A_k141_108820	5336	8	3	1	Low-quality	8.04
Sample_T4C_k141_78229	5006	7	0	0	Low-quality	8.04
Sample_C3B_k141_89941	6320	8	0	2	Low-quality	8.03
Sample_C5C_k141_14968	7057	8	0	1	Low-quality	8.03
Sample_T10A_k141_282	11884	11	0	10	Low-quality	8.03
Sample_T11B_k141_118029	8693	8	0	2	Low-quality	8.03
Sample_T2A_k141_146423	5322	3	0	1	Low-quality	8.03
Sample_C3A_k141_171928	8494	6	5	0	Low-quality	8.03
Sample_T5A_k141_46271	7417	18	0	0	Low-quality	8.03
Sample_T12A_k141_147427	5490	6	0	1	Low-quality	8.02
Sample_T2B_k141_859	5900	1	0	0	Low-quality	8.02
Sample_T4B_k141_270086	13454	10	1	3	Low-quality	8.02
Sample_C11B_k141_40819	8795	12	3	0	Low-quality	8.02

Sample_C1B_k141_203659	6653	13	2	1	Low-quality	8.02
Sample_T5C_k141_81188_fragment_2	8181	13	2	0	Low-quality	8.02
Sample_C14B_k141_1042	15442	27	1	3	Low-quality	8.01
Sample_T8B_k141_62505	8653	9	0	2	Low-quality	8.01
Sample_T2B_k141_106348	5010	15	1	0	Low-quality	8.01
Sample_C10B_k141_116676	16058	12	0	9	Low-quality	8
Sample_C5A_k141_210417	5976	6	0	2	Low-quality	8
Sample_T6C_k141_31206	5171	7	0	3	Low-quality	8
Sample_T7C_k141_4626	6537	8	0	2	Low-quality	8
Sample_T18A_k141_67911	6124	8	1	0	Low-quality	8
Sample_T8A_k141_234211	5023	6	0	0	Low-quality	8
Sample_C10A_k141_27270	22906	21	0	17	Low-quality	7.99
Sample_C14C_k141_34954_fragment_1	7314	15	3	0	Low-quality	7.98
Sample_T17C_k141_34741	7512	10	2	0	Low-quality	7.98
Sample_T6C_k141_76772	15655	16	0	9	Low-quality	7.97
Sample_T11C_k141_90916	8201	11	0	6	Low-quality	7.96
Sample_T7C_k141_3377	7993	3	0	1	Low-quality	7.96
Sample_C2B_k141_315	6475	12	3	0	Low-quality	7.96
Sample_C13A_k141_27850	30589	32	2	5	Low-quality	7.95
Sample_T11B_k141_161940	6470	4	0	0	Low-quality	7.95
Sample_T14C_k141_6727	5678	2	0	0	Low-quality	7.95
Sample_C7C_k141_83029	13193	20	2	0	Low-quality	7.95
Sample_C1C_k141_159207	44188	38	2	22	Low-quality	7.94
Sample_T16B_k141_109381	7970	9	0	2	Low-quality	7.94
Sample_T5B_k141_74483	12675	10	1	2	Low-quality	7.94
Sample_T12B_k141_160141	8158	10	2	0	Low-quality	7.94
Sample_T1C_k141_2733	5940	8	0	0	Low-quality	7.94
Sample_C11C_k141_68805	7104	9	2	0	Low-quality	7.93
Sample_C2A_k141_25958_fragment_1	24337	27	1	3	Low-quality	7.93
Sample_C2B_k141_286900_fragment_1	14462	14	2	0	Low-quality	7.93
Sample_T6B_k141_100636	12549	11	1	9	Low-quality	7.92
Sample_C12B_k141_23095	5237	5	1	0	Low-quality	7.92
Sample_T12C_k141_107891	10105	13	0	1	Low-quality	7.92
Sample_T8B_k141_257898	6764	8	6	1	Low-quality	7.92
Sample_C1C_k141_175418	6639	10	1	0	Low-quality	7.91
Sample_C7A_k141_146846	9598	9	0	4	Low-quality	7.91
Sample_T10A_k141_60037	5164	8	0	1	Low-quality	7.91
Sample_T5A_k141_12062	7842	6	0	0	Low-quality	7.91
Sample_C2A_k141_26876	6369	3	1	0	Low-quality	7.9

Sample_T7C_k141_82578	14573	23	1	2	Low-quality	7.9
Sample_T1C_k141_17208	7555	8	0	1	Low-quality	7.9
Sample_T13C_k141_3112	6451	6	0	1	Low-quality	7.89
Sample_C4C_k141_116964	8384	5	0	0	Low-quality	7.89
Sample_T6B_k141_22983	5417	6	2	0	Low-quality	7.89
Sample_T7A_k141_217624	6434	7	3	0	Low-quality	7.89
Sample_C14C_k141_175338	13939	11	1	3	Low-quality	7.88
Sample_C4A_k141_11730	5814	12	1	1	Low-quality	7.88
Sample_C7C_k141_11760	9090	11	0	4	Low-quality	7.88
Sample_T6A_k141_255022	19548	12	1	5	Low-quality	7.88
Sample_T8C_k141_138117	98063	94	1	48	Low-quality	7.88
Sample_C5C_k141_90344	13343	18	6	1	Low-quality	7.88
Sample_C7A_k141_156327	5153	7	2	0	Low-quality	7.88
Sample_C9B_k141_99404	6497	14	2	0	Low-quality	7.88
Sample_T5B_k141_51863	6518	16	1	1	Low-quality	7.88
Sample_C9A_k141_111256	9599	8	1	0	Low-quality	7.87
Sample_T16B_k141_35686	5382	6	0	1	Low-quality	7.87
Sample_C11B_k141_25533	6932	16	4	0	Low-quality	7.87
Sample_C3A_k141_112057	6816	11	1	0	Low-quality	7.87
Sample_C5A_k141_147213	7197	12	1	0	Low-quality	7.87
Sample_C5A_k141_71509	5482	7	1	0	Low-quality	7.87
Sample_T8C_k141_113637	8511	17	2	0	Low-quality	7.87
Sample_C4A_k141_117910	10706	15	1	3	Low-quality	7.85
Sample_T14C_k141_55274	5095	7	0	2	Low-quality	7.85
Sample_C10C_k141_56636	8357	11	3	0	Low-quality	7.85
Sample_C6A_k141_5088	42945	38	1	23	Low-quality	7.84
Sample_T15A_k141_30995	8474	5	0	3	Low-quality	7.84
Sample_T13C_k141_122601_fragment_1	5831	7	1	0	Low-quality	7.84
Sample_C1A_k141_3031	7290	15	0	0	Low-quality	7.83
Sample_C5C_k141_44377	15952	10	0	6	Low-quality	7.83
Sample_T13B_k141_114712	16218	17	0	15	Low-quality	7.83
Sample_T8A_k141_117605	19029	26	1	4	Low-quality	7.83
Sample_T12C_k141_85543	5449	3	3	0	Low-quality	7.82
Sample_T3B_k141_70283	10583	13	0	5	Low-quality	7.82
Sample_C7B_k141_148562	13222	7	2	0	Low-quality	7.82
Sample_T12A_k141_2940	5166	7	1	0	Low-quality	7.82
Sample_T14B_k141_189176	12912	12	3	0	Low-quality	7.82
Sample_T14C_k141_88954_fragment_2	10576	11	3	3	Low-quality	7.82
Sample_C11A_k141_52506	13636	16	0	15	Low-quality	7.81

Sample_C5C_k141_78821	9034	5	1	0	Low-quality	7.81
Sample_T14C_k141_165977	6339	7	1	1	Low-quality	7.81
Sample_T2A_k141_42578	8441	5	0	1	Low-quality	7.81
Sample_C14C_k141_65456	8104	10	0	1	Low-quality	7.81
Sample_T13C_k141_50008	5159	5	1	0	Low-quality	7.81
Sample_T10A_k141_771	12814	14	1	2	Low-quality	7.8
Sample_T8A_k141_84246	8430	8	0	3	Low-quality	7.8
Sample_C4B_k141_164452	13443	9	0	8	Low-quality	7.79
Sample_T12C_k141_91562	6179	7	2	0	Low-quality	7.79
Sample_T15B_k141_150441	5211	13	3	0	Low-quality	7.78
Sample_C10A_k141_281549	5810	8	2	0	Low-quality	7.78
Sample_C5A_k141_4333	11957	8	3	0	Low-quality	7.78
Sample_T18A_k141_185372	5144	4	1	0	Low-quality	7.78
Sample_T6A_k141_88374	8532	8	0	1	Low-quality	7.77
Sample_T5B_k141_79907	6344	7	0	1	Low-quality	7.76
Sample_T12A_k141_34500	19074	22	3	0	Low-quality	7.76
Sample_T14A_k141_1061	6856	12	2	0	Low-quality	7.76
Sample_C11B_k141_104541	8378	5	0	4	Low-quality	7.75
Sample_C9B_k141_5307	6922	7	1	0	Low-quality	7.75
Sample_C3A_k141_242518	5559	7	2	0	Low-quality	7.75
Sample_T13C_k141_33316	13891	12	1	1	Low-quality	7.75
Sample_C10C_k141_120966	7367	9	1	7	Low-quality	7.74
Sample_C3A_k141_26135	11512	14	1	3	Low-quality	7.74
Sample_T16C_k141_55176	8138	6	0	4	Low-quality	7.74
Sample_C10A_k141_145842	12576	31	2	0	Low-quality	7.74
Sample_C10A_k141_189830	12486	10	1	3	Low-quality	7.74
Sample_C6A_k141_137884	7080	13	1	0	Low-quality	7.74
Sample_T2B_k141_113897	5747	8	1	1	Low-quality	7.74
Sample_T6A_k141_1371	14495	12	0	0	Low-quality	7.74
Sample_T7C_k141_60610	10312	9	4	0	Low-quality	7.74
Sample_C7A_k141_113438	8917	9	0	3	Low-quality	7.73
Sample_T13C_k141_78060	9142	12	0	7	Low-quality	7.72
Sample_C4B_k141_29012	6353	8	4	0	Low-quality	7.72
Sample_C9C_k141_54237	9140	6	0	2	Low-quality	7.71
Sample_C13C_k141_103110	5311	10	3	1	Low-quality	7.71
Sample_C3A_k141_59429	5468	12	0	0	Low-quality	7.71
Sample_C3C_k141_215733	6474	10	1	1	Low-quality	7.71
Sample_T14A_k141_94423	8514	6	0	0	Low-quality	7.71
Sample_C10C_k141_88353	11037	16	1	0	Low-quality	7.7

Sample_C2A_k141_142482	12252	13	1	1	Low-quality	7.7
Sample_C8B_k141_64545	21639	21	1	4	Low-quality	7.7
Sample_T15A_k141_41110	5518	3	0	2	Low-quality	7.7
Sample_T17C_k141_11852	7462	6	3	1	Low-quality	7.7
Sample_T8B_k141_23085_fragment_1	12591	14	1	0	Low-quality	7.7
Sample_C12A_k141_56971	14175	20	1	2	Low-quality	7.69
Sample_C6B_k141_21685	6118	3	0	2	Low-quality	7.69
Sample_C6C_k141_178879	15682	17	1	3	Low-quality	7.69
Sample_C8C_k141_87973	29214	29	1	8	Low-quality	7.69
Sample_C5A_k141_92609	7621	11	2	0	Low-quality	7.69
Sample_T13C_k141_31111	5276	11	1	1	Low-quality	7.69
Sample_C10C_k141_111256	5261	3	0	1	Low-quality	7.68
Sample_C12A_k141_150237	9629	9	1	1	Low-quality	7.68
Sample_T3C_k141_109718	10334	13	1	6	Low-quality	7.68
Sample_C11C_k141_130278	13069	17	2	0	Low-quality	7.68
Sample_C5C_k141_15272	13111	15	6	0	Low-quality	7.68
Sample_T6A_k141_107604	6268	2	0	0	Low-quality	7.67
Sample_T8B_k141_46522	13387	10	0	8	Low-quality	7.67
Sample_C3A_k141_210139_fragment_1	7977	9	0	3	Low-quality	7.67
Sample_T7B_k141_17961	5007	5	1	0	Low-quality	7.67
Sample_T8C_k141_112840	5020	4	1	1	Low-quality	7.67
Sample_T6C_k141_265908	15165	5	0	5	Low-quality	7.66
Sample_C6B_k141_100316	5426	7	0	1	Low-quality	7.66
Sample_C10A_k141_178001	6447	8	0	1	Low-quality	7.65
Sample_T12A_k141_272447	29496	26	1	22	Low-quality	7.65
Sample_T2B_k141_191436	14113	13	1	6	Low-quality	7.65
Sample_C12B_k141_78892	5728	11	4	0	Low-quality	7.64
Sample_T12A_k141_266587	6654	7	1	1	Low-quality	7.64
Sample_T2A_k141_134189	14714	13	1	0	Low-quality	7.63
Sample_C2B_k141_346634_fragment_1	13358	14	2	0	Low-quality	7.63
Sample_T10B_k141_54751	5538	10	1	0	Low-quality	7.62
Sample_T7A_k141_107525	10759	14	3	2	Low-quality	7.62
Sample_T3C_k141_141744	5095	7	1	0	Low-quality	7.62
Sample_C5C_k141_117086	5229	4	0	0	Low-quality	7.61
Sample_T15B_k141_78782	13286	11	0	9	Low-quality	7.61
Sample_C10A_k141_111454	8883	9	0	1	Low-quality	7.61
Sample_T12A_k141_234285	18791	23	2	0	Low-quality	7.61
Sample_C6A_k141_27947	10606	13	3	0	Low-quality	7.6
Sample_C10A_k141_151514	15239	13	0	12	Low-quality	7.59

Sample_C12C_k141_27955	5019	6	1	0	Low-quality	7.59
Sample_T6A_k141_281282	27689	18	1	7	Low-quality	7.59
Sample_C11B_k141_174665_fragment_1	11091	14	2	0	Low-quality	7.59
Sample_C11B_k141_189433	5218	8	1	1	Low-quality	7.59
Sample_T12C_k141_17771	11793	24	2	0	Low-quality	7.59
Sample_T14C_k141_33441	5321	13	2	0	Low-quality	7.58
Sample_T7B_k141_40996	13575	12	1	2	Low-quality	7.57
Sample_C2A_k141_61829	6119	7	1	0	Low-quality	7.57
Sample_C2C_k141_213376	8002	13	0	0	Low-quality	7.57
Sample_T8B_k141_206257	8280	10	0	0	Low-quality	7.57
Sample_T15B_k141_11774	8424	8	0	1	Low-quality	7.56
Sample_C10C_k141_125284	8172	7	0	1	Low-quality	7.55
Sample_T12A_k141_59352	6071	11	4	0	Low-quality	7.55
Sample_T12B_k141_160291	6353	7	0	1	Low-quality	7.55
Sample_T2C_k141_199969	15664	16	1	6	Low-quality	7.55
Sample_C12A_k141_122094_fragment_1	13926	11	1	1	Low-quality	7.55
Sample_T6B_k141_121138	12250	13	2	3	Low-quality	7.54
Sample_T16B_k141_100928	8363	13	2	0	Low-quality	7.54
Sample_C3A_k141_178011	5245	7	1	0	Low-quality	7.53
Sample_T7A_k141_110899	6040	11	0	0	Low-quality	7.53
Sample_T10A_k141_93517	6292	10	1	1	Low-quality	7.52
Sample_C11C_k141_97048	324869	288	5	194	Low-quality	7.51
Sample_C13A_k141_56282	6514	16	0	0	Low-quality	7.51
Sample_T11C_k141_40076	8482	8	1	1	Low-quality	7.51
Sample_C10C_k141_122236	5868	8	0	1	Low-quality	7.51
Sample_T11B_k141_33974	5891	12	1	0	Low-quality	7.51
Sample_T4A_k141_100934	6097	7	0	0	Low-quality	7.51
Sample_C1A_k141_112999	6052	6	0	5	Low-quality	7.5
Sample_C5A_k141_160487	7795	8	0	3	Low-quality	7.5
Sample_T8B_k141_147743	12711	14	3	0	Low-quality	7.5
Sample_T16B_k141_140886	5811	8	1	0	Low-quality	7.5
Sample_C12A_k141_179657	5092	4	0	0	Low-quality	7.49
Sample_T12C_k141_168682	5173	7	0	1	Low-quality	7.49
Sample_T7A_k141_64629	10809	13	0	3	Low-quality	7.49
Sample_C12C_k141_230919	5090	3	0	0	Low-quality	7.48
Sample_T12A_k141_322541	10388	14	1	2	Low-quality	7.48
Sample_T12A_k141_23142	12355	11	1	8	Low-quality	7.48
Sample_T18C_k141_125829	44704	48	3	12	Low-quality	7.48
Sample_T3B_k141_62883	7402	19	2	0	Low-quality	7.48

Sample_C11A_k141_25666	6805	5	0	0	Low-quality	7.47
Sample_C4B_k141_14685	59348	36	2	18	Low-quality	7.47
Sample_C5C_k141_44780	9272	6	0	6	Low-quality	7.47
Sample_C2A_k141_322888	5014	9	4	0	Low-quality	7.47
Sample_T8A_k141_134778	6099	7	0	2	Low-quality	7.46
Sample_C4A_k141_139392	5192	7	1	0	Low-quality	7.46
Sample_C11C_k141_154534	7068	8	0	3	Low-quality	7.45
Sample_C8B_k141_62134	15176	12	0	11	Low-quality	7.45
Sample_T14C_k141_139799	6281	8	0	1	Low-quality	7.45
Sample_C2A_k141_302918	8138	9	1	3	Low-quality	7.44
Sample_C2A_k141_190195	9272	11	1	2	Low-quality	7.44
Sample_C2C_k141_274429	5044	6	1	1	Low-quality	7.44
Sample_C4B_k141_228018	9958	21	5	0	Low-quality	7.44
Sample_T6B_k141_30951	7057	5	0	1	Low-quality	7.44
Sample_C13A_k141_110311	8344	7	0	2	Low-quality	7.43
Sample_C3A_k141_134950	6074	15	1	0	Low-quality	7.42
Sample_C6B_k141_19815	5103	7	0	2	Low-quality	7.42
Sample_C8B_k141_45762	8021	12	1	0	Low-quality	7.42
Sample_T12C_k141_114077	5675	14	0	1	Low-quality	7.41
Sample_C3A_k141_29701	11806	13	1	1	Low-quality	7.41
Sample_C3A_k141_101248	8090	8	4	0	Low-quality	7.41
Sample_C6C_k141_51555	5796	1	0	0	Low-quality	7.4
Sample_T5B_k141_80046	10571	4	0	0	Low-quality	7.4
Sample_T7C_k141_14842	8668	7	0	2	Low-quality	7.4
Sample_C3B_k141_221841_fragment_1	5784	10	3	1	Low-quality	7.4
Sample_C4A_k141_249519	10768	17	9	0	Low-quality	7.4
Sample_C4C_k141_22297	5290	3	2	0	Low-quality	7.39
Sample_T15B_k141_57933	14454	19	1	2	Low-quality	7.39
Sample_T2A_k141_148064	7839	6	0	2	Low-quality	7.39
Sample_T5B_k141_63357	10846	10	0	1	Low-quality	7.39
Sample_T6B_k141_107159_fragment_1	12868	13	1	0	Low-quality	7.39
Sample_T12B_k141_160665	5733	4	0	1	Low-quality	7.38
Sample_T4B_k141_198602	9816	7	0	2	Low-quality	7.38
Sample_C5B_k141_13262	5360	13	3	0	Low-quality	7.38
Sample_T12C_k141_140787	5809	11	1	0	Low-quality	7.38
Sample_T4B_k141_333361	6128	10	0	2	Low-quality	7.38
Sample_T2A_k141_57000	5243	3	0	1	Low-quality	7.37
Sample_T8B_k141_164729	7090	3	0	1	Low-quality	7.37
Sample_C2C_k141_188713	19041	28	2	2	Low-quality	7.36

Sample_T8A_k141_207221	6559	6	1	1	Low-quality	7.36
Sample_T12C_k141_1334	10060	21	2	2	Low-quality	7.35
Sample_C9C_k141_8128	5037	6	1	0	Low-quality	7.35
Sample_C8B_k141_57317	7027	12	0	0	Low-quality	7.34
Sample_T3A_k141_6685	23016	24	0	12	Low-quality	7.34
Sample_T4B_k141_22214	7788	5	0	4	Low-quality	7.34
Sample_T8C_k141_151288	5933	9	1	3	Low-quality	7.34
Sample_C10A_k141_314862	9129	13	1	2	Low-quality	7.33
Sample_C7A_k141_160812	5140	11	1	0	Low-quality	7.33
Sample_C6B_k141_70503	37631	32	0	28	Low-quality	7.32
Sample_T15C_k141_48890	6237	7	1	1	Low-quality	7.32
Sample_C11A_k141_110782	13483	15	1	8	Low-quality	7.31
Sample_T12A_k141_147854	6219	6	0	4	Low-quality	7.31
Sample_T12C_k141_178352	5760	13	2	1	Low-quality	7.3
Sample_T3A_k141_40783	5007	7	1	1	Low-quality	7.3
Sample_T12B_k141_105171	5312	3	0	0	Low-quality	7.29
Sample_T17C_k141_7955	11272	15	1	2	Low-quality	7.29
Sample_C7B_k141_103	8631	11	9	0	Low-quality	7.29
Sample_T7C_k141_106935	5939	10	2	0	Low-quality	7.29
Sample_C14C_k141_105429	23584	23	0	22	Low-quality	7.28
Sample_C2B_k141_177466	5672	8	1	0	Low-quality	7.28
Sample_C3B_k141_170991	9642	8	0	1	Low-quality	7.28
Sample_C9A_k141_87889	14674	13	1	9	Low-quality	7.28
Sample_T10C_k141_10486	5789	2	0	0	Low-quality	7.28
Sample_T7A_k141_168483	20206	16	1	5	Low-quality	7.28
Sample_C9B_k141_43376	6561	9	3	1	Low-quality	7.28
Sample_C4C_k141_202056	14813	15	1	5	Low-quality	7.27
Sample_T13B_k141_162515	5444	2	0	0	Low-quality	7.27
Sample_T6A_k141_176531	10321	14	9	0	Low-quality	7.27
Sample_C11B_k141_165051	14952	13	1	12	Low-quality	7.26
Sample_C8C_k141_110149	7905	20	2	0	Low-quality	7.26
Sample_T4A_k141_90320	7491	4	0	4	Low-quality	7.26
Sample_C5A_k141_182428	15626	35	2	1	Low-quality	7.26
Sample_T7A_k141_65487	5053	9	0	0	Low-quality	7.26
Sample_C10A_k141_79488	77709	64	0	54	Low-quality	7.25
Sample_T1B_k141_870	7273	7	1	0	Low-quality	7.25
Sample_T15B_k141_35284	10885	13	0	4	Low-quality	7.25
Sample_T8B_k141_181596	11723	23	5	0	Low-quality	7.25
Sample_C12C_k141_230539	5081	5	0	1	Low-quality	7.24

Sample_C1B_k141_3520	8898	8	0	3	Low-quality	7.24
Sample_C2C_k141_177025	8327	4	0	0	Low-quality	7.24
Sample_C7B_k141_169761	12652	14	2	0	Low-quality	7.24
Sample_C1C_k141_167669	5300	5	0	3	Low-quality	7.23
Sample_C6B_k141_116724	14712	9	3	1	Low-quality	7.23
Sample_T8B_k141_114307	22525	25	0	10	Low-quality	7.23
Sample_C8B_k141_197333	8974	6	0	1	Low-quality	7.23
Sample_T8A_k141_155447	14589	34	6	0	Low-quality	7.23
Sample_C6C_k141_32955	22846	26	0	17	Low-quality	7.22
Sample_C8A_k141_22509	5170	7	2	0	Low-quality	7.22
Sample_T15C_k141_146213	10327	7	1	1	Low-quality	7.22
Sample_T8B_k141_29359	9198	9	0	6	Low-quality	7.22
Sample_T12A_k141_1696	36410	39	0	36	Low-quality	7.21
Sample_T14A_k141_23389	7228	6	1	1	Low-quality	7.21
Sample_T8B_k141_84608	7778	6	0	1	Low-quality	7.21
Sample_C10A_k141_219521	6001	8	3	0	Low-quality	7.21
Sample_C10A_k141_178773	7620	5	0	3	Low-quality	7.2
Sample_C10C_k141_111606	5259	6	0	4	Low-quality	7.2
Sample_C4C_k141_68298	20813	18	0	13	Low-quality	7.2
Sample_C10A_k141_253756	5460	4	2	0	Low-quality	7.2
Sample_C8C_k141_13151	8967	10	1	2	Low-quality	7.2
Sample_T12A_k141_188875	7119	8	3	0	Low-quality	7.2
Sample_T12B_k141_52459	5724	15	1	2	Low-quality	7.2
Sample_T8C_k141_3356	6279	15	3	0	Low-quality	7.2
Sample_C11A_k141_21814	6546	3	0	1	Low-quality	7.19
Sample_C1C_k141_100050	7317	3	0	0	Low-quality	7.19
Sample_C5A_k141_155995	7159	5	1	1	Low-quality	7.19
Sample_T2C_k141_174169	5605	10	0	1	Low-quality	7.19
Sample_T8B_k141_130520	12366	15	1	10	Low-quality	7.19
Sample_C6A_k141_70817	7607	4	0	4	Low-quality	7.18
Sample_C8B_k141_47249	11835	17	2	2	Low-quality	7.18
Sample_T12C_k141_167333	17431	22	0	4	Low-quality	7.18
Sample_T14B_k141_63242	12870	18	1	0	Low-quality	7.18
Sample_C13A_k141_39594	5260	5	0	1	Low-quality	7.17
Sample_C4B_k141_57752	8431	7	0	6	Low-quality	7.17
Sample_C5C_k141_24926	24471	22	0	21	Low-quality	7.17
Sample_T11C_k141_65088	10079	14	0	2	Low-quality	7.17
Sample_C9B_k141_53597	8498	11	1	0	Low-quality	7.16
Sample_C13A_k141_81664	7751	7	0	1	Low-quality	7.15

Sample_C13B_k141_115622	33351	32	1	8	Low-quality	7.15
Sample_C13C_k141_78744	6637	1	0	0	Low-quality	7.15
Sample_T16B_k141_161155	8116	10	0	4	Low-quality	7.15
Sample_C6A_k141_200247	6204	8	1	0	Low-quality	7.14
Sample_C8B_k141_173747	5878	10	0	2	Low-quality	7.14
Sample_T10A_k141_112365	8798	11	1	2	Low-quality	7.14
Sample_T12C_k141_37281	12666	16	2	0	Low-quality	7.14
Sample_C1C_k141_194741	7581	8	1	0	Low-quality	7.14
Sample_T6B_k141_91748	5288	6	2	0	Low-quality	7.14
Sample_C14A_k141_77993	5870	7	0	0	Low-quality	7.13
Sample_C5C_k141_113106	7152	2	0	1	Low-quality	7.13
Sample_C6B_k141_64726	17892	13	0	11	Low-quality	7.13
Sample_C6C_k141_111057	5773	9	0	0	Low-quality	7.13
Sample_T12A_k141_65786	15394	9	0	6	Low-quality	7.13
Sample_T15A_k141_44739	11305	19	2	2	Low-quality	7.13
Sample_T4A_k141_2085	6352	4	0	1	Low-quality	7.13
Sample_C1A_k141_84203	11037	11	0	8	Low-quality	7.12
Sample_C2C_k141_207035	7991	4	1	0	Low-quality	7.12
Sample_T1A_k141_16690	7540	4	0	1	Low-quality	7.12
Sample_T15B_k141_9337	9255	9	1	0	Low-quality	7.12
Sample_T10A_k141_92783	9217	16	1	0	Low-quality	7.11
Sample_T4A_k141_15075	6804	6	0	0	Low-quality	7.11
Sample_C11A_k141_90342	12582	13	1	0	Low-quality	7.11
Sample_C4A_k141_125997	6202	8	1	1	Low-quality	7.11
Sample_T2C_k141_43480	8838	17	3	1	Low-quality	7.11
Sample_T10A_k141_56154	5746	2	0	0	Low-quality	7.1
Sample_T12C_k141_19155	88111	85	1	39	Low-quality	7.1
Sample_T15C_k141_145370	6393	5	0	1	Low-quality	7.1
Sample_T5C_k141_177649	7021	8	1	1	Low-quality	7.1
Sample_T17A_k141_20330	13576	16	2	0	Low-quality	7.1
Sample_C10B_k141_52852	7411	5	0	4	Low-quality	7.09
Sample_T12A_k141_185956	5751	7	1	1	Low-quality	7.09
Sample_T5B_k141_71599	8394	5	0	2	Low-quality	7.09
Sample_T6A_k141_64430	15008	11	0	9	Low-quality	7.09
Sample_T6C_k141_55060	5837	7	0	0	Low-quality	7.09
Sample_C12A_k141_131347	10267	24	0	0	Low-quality	7.08
Sample_T2A_k141_99787	18893	14	0	9	Low-quality	7.08
Sample_C11B_k141_72942	12044	25	1	1	Low-quality	7.08
Sample_C12B_k141_199794	18633	22	2	0	Low-quality	7.08

Sample_T12B_k141_63180	5577	5	3	0	Low-quality	7.08
Sample_T15A_k141_65896	9182	12	1	0	Low-quality	7.08
Sample_C6B_k141_209765_fragment_2	5664	8	3	0	Low-quality	7.07
Sample_T11B_k141_132895	9439	11	0	3	Low-quality	7.07
Sample_T14A_k141_142638	10716	12	2	0	Low-quality	7.06
Sample_T18C_k141_2890	5701	7	1	3	Low-quality	7.06
Sample_T16B_k141_1818	6433	7	0	0	Low-quality	7.06
Sample_C3A_k141_60909	6661	2	0	1	Low-quality	7.05
Sample_T11B_k141_70250	5775	9	1	0	Low-quality	7.05
Sample_T12C_k141_8444	5525	6	1	0	Low-quality	7.05
Sample_T11A_k141_5902	32376	36	2	8	Low-quality	7.04
Sample_C1A_k141_142201	5749	4	0	2	Low-quality	7.03
Sample_C2C_k141_139019	11196	9	0	2	Low-quality	7.03
Sample_T11B_k141_102040	5444	8	0	1	Low-quality	7.03
Sample_T15C_k141_63904	16160	16	1	13	Low-quality	7.03
Sample_T4B_k141_75625	7054	9	1	1	Low-quality	7.03
Sample_C1B_k141_29602	5399	8	5	0	Low-quality	7.03
Sample_T16B_k141_72743	11890	8	2	0	Low-quality	7.03
Sample_T4A_k141_120864	6260	8	1	1	Low-quality	7.03
Sample_C6B_k141_70693	9940	6	2	2	Low-quality	7.02
Sample_C7B_k141_15009	7067	11	0	0	Low-quality	7.02
Sample_T10B_k141_36799	5140	5	0	2	Low-quality	7.02
Sample_T6A_k141_33559	6670	7	0	0	Low-quality	7.02
Sample_T4B_k141_127323	13148	18	1	1	Low-quality	7.01
Sample_T4C_k141_45627	5595	4	0	2	Low-quality	7.01
Sample_T8B_k141_134182	9056	6	1	3	Low-quality	7.01
Sample_C10C_k141_27860	5076	6	1	1	Low-quality	7.01
Sample_T6C_k141_15713_fragment_1	10828	12	1	1	Low-quality	7.01
Sample_C1B_k141_138202	5854	4	0	4	Low-quality	7
Sample_C8C_k141_21573	5654	6	1	2	Low-quality	7
Sample_T2B_k141_177257	5760	7	0	3	Low-quality	7
Sample_T6A_k141_195259	13016	15	1	1	Low-quality	7
Sample_C4B_k141_81483	9212	4	0	1	Low-quality	6.99
Sample_C6A_k141_2522	6511	7	1	1	Low-quality	6.99
Sample_C13B_k141_104154	5491	5	0	0	Low-quality	6.98
Sample_T5A_k141_13165	5707	5	0	1	Low-quality	6.98
Sample_T8C_k141_14603	5357	10	6	0	Low-quality	6.98
Sample_C1B_k141_181242	12642	12	0	8	Low-quality	6.97
Sample_T12C_k141_73335	7126	4	0	2	Low-quality	6.97

Sample_T18A_k141_167993	5778	1	0	0	Low-quality	6.97
Sample_T2A_k141_148160	10126	12	0	8	Low-quality	6.97
Sample_C11A_k141_146117	7698	10	1	1	Low-quality	6.96
Sample_C12C_k141_4395	26772	30	5	3	Low-quality	6.96
Sample_C2C_k141_173334_fragment_6	8593	10	1	2	Low-quality	6.96
Sample_C3A_k141_235397	7592	12	5	1	Low-quality	6.96
Sample_T15B_k141_84104	9009	8	1	2	Low-quality	6.96
Sample_T18C_k141_43172_fragment_1	10868	10	1	2	Low-quality	6.96
Sample_T2A_k141_61392	5692	11	0	2	Low-quality	6.96
Sample_T4B_k141_144875	5051	11	3	0	Low-quality	6.96
Sample_T7B_k141_34547	5332	6	0	1	Low-quality	6.96
Sample_C11B_k141_20625	7259	6	0	1	Low-quality	6.95
Sample_C2B_k141_336923	5736	5	2	0	Low-quality	6.95
Sample_C3B_k141_82820	10346	11	1	1	Low-quality	6.95
Sample_C4A_k141_18189	6437	12	2	0	Low-quality	6.95
Sample_T2B_k141_264721	14565	14	5	1	Low-quality	6.95
Sample_T4B_k141_313107	6237	6	1	0	Low-quality	6.95
Sample_C12C_k141_164487	11172	14	1	0	Low-quality	6.94
Sample_T2B_k141_142089	5680	7	1	0	Low-quality	6.94
Sample_T4B_k141_136439	8450	11	0	0	Low-quality	6.94
Sample_C10A_k141_23642	11738	13	7	0	Low-quality	6.94
Sample_C1A_k141_257264	5562	8	0	0	Low-quality	6.94
Sample_C3A_k141_159382	5357	4	3	0	Low-quality	6.94
Sample_T12C_k141_17556	7754	12	1	1	Low-quality	6.94
Sample_C13A_k141_78644	5806	3	0	1	Low-quality	6.93
Sample_T12C_k141_85962	5387	14	1	1	Low-quality	6.93
Sample_T5B_k141_34874	10876	10	1	3	Low-quality	6.93
Sample_T6A_k141_282179	9045	13	0	0	Low-quality	6.92
Sample_C10A_k141_231651	11205	10	1	1	Low-quality	6.92
Sample_C4A_k141_49703	6866	8	3	0	Low-quality	6.92
Sample_C5A_k141_270013_fragment_3	10301	19	1	0	Low-quality	6.92
Sample_C8A_k141_165448	5427	3	0	1	Low-quality	6.91
Sample_T6A_k141_23881	12496	19	1	0	Low-quality	6.91
Sample_C3A_k141_55667	5903	9	4	0	Low-quality	6.91
Sample_C4B_k141_137767	9985	10	0	1	Low-quality	6.9
Sample_C8A_k141_134119	13201	10	1	8	Low-quality	6.9
Sample_T10A_k141_117669	12720	15	1	1	Low-quality	6.9
Sample_T3C_k141_58859	12218	15	1	3	Low-quality	6.9
Sample_T12A_k141_109080	10965	13	1	2	Low-quality	6.89

Sample_T12B_k141_25912	9582	13	0	0	Low-quality	6.88
Sample_C5A_k141_36268	14917	36	7	0	Low-quality	6.88
Sample_T12A_k141_61043	10942	17	4	1	Low-quality	6.88
Sample_T4B_k141_90670	5252	6	1	0	Low-quality	6.88
Sample_T5C_k141_49268	5537	6	0	1	Low-quality	6.88
Sample_C1B_k141_256029	10919	8	0	4	Low-quality	6.87
Sample_T15C_k141_9818	5909	10	1	2	Low-quality	6.87
Sample_C2A_k141_79694	5413	8	3	1	Low-quality	6.86
Sample_T10B_k141_118795	8758	5	0	0	Low-quality	6.86
Sample_T11C_k141_147293	5180	8	1	0	Low-quality	6.86
Sample_C9A_k141_99657	7384	6	0	1	Low-quality	6.86
Sample_C2A_k141_232351	27122	38	2	13	Low-quality	6.84
Sample_T17B_k141_26831	10175	13	1	1	Low-quality	6.84
Sample_C14A_k141_48922	7069	13	0	5	Low-quality	6.83
Sample_C9A_k141_100985	7381	4	0	2	Low-quality	6.83
Sample_T4A_k141_89181	5569	8	2	0	Low-quality	6.83
Sample_C9B_k141_56920	5064	11	3	0	Low-quality	6.82
Sample_T15A_k141_153001	7550	6	0	1	Low-quality	6.82
Sample_T8A_k141_194733	37368	29	1	23	Low-quality	6.82
Sample_C10A_k141_51439	8102	10	1	2	Low-quality	6.82
Sample_C5A_k141_191507	11850	22	1	0	Low-quality	6.81
Sample_T13B_k141_132022	7449	6	1	2	Low-quality	6.81
Sample_T2B_k141_60377	84662	43	1	36	Low-quality	6.81
Sample_T3B_k141_15629	9919	9	0	7	Low-quality	6.81
Sample_T18B_k141_53163	5604	4	2	0	Low-quality	6.81
Sample_T4A_k141_34904	5635	1	0	0	Low-quality	6.8
Sample_C4A_k141_148632	5225	7	2	0	Low-quality	6.8
Sample_T6C_k141_58677	8702	15	0	1	Low-quality	6.8
Sample_T3B_k141_32482	24225	28	1	5	Low-quality	6.79
Sample_T7A_k141_107469	13420	20	5	0	Low-quality	6.79
Sample_C14C_k141_121699	6397	10	4	2	Low-quality	6.79
Sample_T14A_k141_217533	10089	13	1	3	Low-quality	6.78
Sample_T15B_k141_8873	7054	7	0	2	Low-quality	6.78
Sample_C10C_k141_6713	19754	24	0	15	Low-quality	6.77
Sample_C2C_k141_2056	9161	8	0	6	Low-quality	6.77
Sample_C9C_k141_42316	5645	6	0	1	Low-quality	6.77
Sample_T11C_k141_21283	5439	5	0	2	Low-quality	6.77
Sample_T4A_k141_88535	6467	7	0	0	Low-quality	6.77
Sample_T6A_k141_252908	17164	15	0	9	Low-quality	6.77

Sample_T12A_k141_161993	5429	9	2	0	Low-quality	6.77
Sample_C8B_k141_159568	6740	3	1	0	Low-quality	6.76
Sample_T5C_k141_9333	7594	10	0	2	Low-quality	6.76
Sample_C11A_k141_170200	5557	4	0	0	Low-quality	6.75
Sample_C13A_k141_47160	13173	12	0	7	Low-quality	6.75
Sample_C3B_k141_72612	7803	4	1	0	Low-quality	6.75
Sample_C6C_k141_44926	7563	8	0	5	Low-quality	6.75
Sample_T12C_k141_96525	6804	19	0	0	Low-quality	6.75
Sample_T14B_k141_180151	7328	8	0	3	Low-quality	6.75
Sample_T2A_k141_256867	38229	39	1	23	Low-quality	6.75
Sample_T2C_k141_29716	11561	17	2	1	Low-quality	6.75
Sample_T8B_k141_11305	26655	33	0	18	Low-quality	6.75
Sample_C11B_k141_115085	7006	10	1	0	Low-quality	6.75
Sample_C8A_k141_139368	18966	17	1	0	Low-quality	6.75
Sample_C8B_k141_72340	7358	4	0	2	Low-quality	6.74
Sample_T12B_k141_88081	7785	7	0	2	Low-quality	6.74
Sample_T5B_k141_59186	7076	8	0	1	Low-quality	6.74
Sample_T5C_k141_167382	25186	21	0	12	Low-quality	6.74
Sample_C5A_k141_115592	6962	6	0	1	Low-quality	6.74
Sample_C10C_k141_217421	13719	16	1	2	Low-quality	6.73
Sample_T15C_k141_84184	6132	4	0	1	Low-quality	6.73
Sample_C10A_k141_132397	5632	6	1	0	Low-quality	6.73
Sample_C11B_k141_192238	6527	14	3	0	Low-quality	6.73
Sample_C7B_k141_80302	7948	13	2	0	Low-quality	6.73
Sample_T16A_k141_42085	11011	10	1	0	Low-quality	6.73
Sample_T17B_k141_26354	7453	8	0	1	Low-quality	6.73
Sample_T15B_k141_140925	7191	1	1	0	Low-quality	6.72
Sample_C14C_k141_130046	8977	11	1	0	Low-quality	6.72
Sample_T12C_k141_67882	11700	13	2	0	Low-quality	6.72
Sample_T14B_k141_142070	7077	6	0	0	Low-quality	6.72
Sample_T7A_k141_124597	7407	8	2	0	Low-quality	6.72
Sample_T13B_k141_132893	5419	8	1	3	Low-quality	6.71
Sample_T15A_k141_80063	9746	12	2	3	Low-quality	6.71
Sample_T1C_k141_4051	5673	4	0	1	Low-quality	6.71
Sample_T3B_k141_99348	7387	11	8	2	Low-quality	6.71
Sample_T12A_k141_334885	9938	11	2	3	Low-quality	6.7
Sample_T2B_k141_103479	8847	9	0	0	Low-quality	6.69
Sample_C10C_k141_39041	6786	8	0	3	Low-quality	6.68
Sample_T10C_k141_27680	7176	9	0	3	Low-quality	6.68

Sample_T11B_k141_126622	11658	10	0	7	Low-quality	6.68
Sample_T12B_k141_100889	9934	12	1	2	Low-quality	6.68
Sample_C1B_k141_8784	9825	15	0	0	Low-quality	6.68
Sample_C4A_k141_178607	5195	9	3	3	Low-quality	6.68
Sample_T6A_k141_223501	13224	10	1	1	Low-quality	6.68
Sample_C1C_k141_190484	7020	10	0	3	Low-quality	6.67
Sample_C3A_k141_152461	8357	7	0	0	Low-quality	6.67
Sample_C6A_k141_31762	6262	9	0	3	Low-quality	6.67
Sample_T15B_k141_91904	7518	9	0	2	Low-quality	6.67
Sample_C6B_k141_94056	12405	11	1	2	Low-quality	6.67
Sample_C12C_k141_205011	12284	18	1	1	Low-quality	6.66
Sample_C11B_k141_102591	6914	11	5	0	Low-quality	6.66
Sample_C14C_k141_152807	9904	21	1	1	Low-quality	6.66
Sample_T2C_k141_213065	7545	10	3	1	Low-quality	6.66
Sample_C12B_k141_224	7463	6	0	2	Low-quality	6.65
Sample_C7B_k141_82055	6254	3	1	0	Low-quality	6.65
Sample_T11B_k141_117208	9286	7	0	5	Low-quality	6.65
Sample_T12A_k141_175946	5431	5	0	2	Low-quality	6.65
Sample_T8B_k141_159869	6449	12	3	0	Low-quality	6.65
Sample_C13A_k141_46634	9732	15	2	2	Low-quality	6.64
Sample_T6A_k141_126695	15758	12	0	8	Low-quality	6.64
Sample_T6A_k141_181894	7771	8	1	1	Low-quality	6.64
Sample_C4B_k141_189510	11082	12	1	0	Low-quality	6.64
Sample_C13B_k141_102254	5458	8	0	1	Low-quality	6.63
Sample_T11C_k141_91302	5758	3	2	0	Low-quality	6.63
Sample_T15C_k141_64917	6818	8	0	2	Low-quality	6.63
Sample_T2B_k141_47040	8168	14	1	0	Low-quality	6.63
Sample_T6A_k141_275421	11990	20	0	1	Low-quality	6.63
Sample_C2C_k141_96791	5043	11	0	0	Low-quality	6.63
Sample_T12B_k141_113679	5175	9	1	0	Low-quality	6.63
Sample_C4B_k141_132263	5280	7	0	5	Low-quality	6.62
Sample_C9A_k141_68844	5003	5	0	0	Low-quality	6.62
Sample_T12C_k141_31355	5241	10	2	0	Low-quality	6.62
Sample_T2C_k141_168371	10375	19	1	1	Low-quality	6.62
Sample_C1B_k141_87521	5949	10	1	1	Low-quality	6.61
Sample_C13A_k141_19287	5720	10	0	0	Low-quality	6.61
Sample_T15A_k141_135509	7176	6	0	2	Low-quality	6.61
Sample_C10C_k141_230038	10687	13	1	1	Low-quality	6.6
Sample_T16B_k141_53309	5485	7	0	1	Low-quality	6.6

Sample_T10A_k141_113537	11416	15	8	0	Low-quality	6.6
Sample_C14A_k141_106030	6364	7	1	1	Low-quality	6.59
Sample_C14A_k141_215538	12560	18	2	3	Low-quality	6.59
Sample_C9A_k141_36588	6781	7	0	1	Low-quality	6.59
Sample_T14A_k141_191733	5425	5	3	0	Low-quality	6.59
Sample_C10A_k141_275423	9795	9	1	2	Low-quality	6.58
Sample_C10B_k141_67266	6666	7	0	3	Low-quality	6.58
Sample_C1C_k141_62396	103781	91	1	45	Low-quality	6.58
Sample_C2A_k141_200085	7269	6	0	3	Low-quality	6.58
Sample_C2B_k141_217137	8490	4	0	2	Low-quality	6.58
Sample_T12A_k141_221962	6609	8	0	2	Low-quality	6.58
Sample_T16B_k141_54670	5381	5	0	4	Low-quality	6.58
Sample_C9A_k141_67744_fragment_1	13304	8	3	2	Low-quality	6.58
Sample_T13B_k141_128053	7243	9	1	0	Low-quality	6.58
Sample_T15A_k141_104342	8281	10	1	0	Low-quality	6.58
Sample_T8B_k141_164901	9837	8	0	0	Low-quality	6.58
Sample_C10C_k141_219250	33066	23	2	4	Low-quality	6.57
Sample_C1C_k141_157524	6753	5	0	5	Low-quality	6.57
Sample_T2B_k141_114544	18336	15	1	7	Low-quality	6.57
Sample_T3A_k141_102706	5361	11	0	0	Low-quality	6.57
Sample_T4B_k141_21477	6222	3	0	2	Low-quality	6.57
Sample_T5C_k141_42773	8817	9	0	6	Low-quality	6.57
Sample_C1B_k141_113448	14236	10	0	8	Low-quality	6.56
Sample_C2B_k141_255689	5329	6	0	1	Low-quality	6.56
Sample_C4C_k141_221817	9473	8	0	4	Low-quality	6.56
Sample_T2C_k141_256233	5144	6	1	1	Low-quality	6.56
Sample_C2A_k141_180698	5746	10	0	1	Low-quality	6.56
Sample_C6C_k141_39201	11436	13	0	12	Low-quality	6.55
Sample_T11C_k141_22069	10274	15	1	2	Low-quality	6.55
Sample_T4C_k141_52161	13277	11	0	10	Low-quality	6.54
Sample_T11C_k141_78280	5389	6	1	0	Low-quality	6.54
Sample_T16A_k141_10778	5683	8	1	0	Low-quality	6.54
Sample_C11A_k141_51126	5196	4	0	4	Low-quality	6.53
Sample_C9B_k141_95167	35778	27	1	19	Low-quality	6.53
Sample_C10A_k141_35429	6250	7	3	1	Low-quality	6.53
Sample_T10A_k141_7907	10688	21	1	0	Low-quality	6.53
Sample_C10A_k141_288974	7088	3	0	1	Low-quality	6.52
Sample_C13B_k141_17030	6280	7	1	2	Low-quality	6.52
Sample_C1C_k141_44626	7239	7	0	5	Low-quality	6.52

Sample_C10C_k141_185438	10323	12	1	1	Low-quality	6.51
Sample_C3A_k141_34460	5311	13	2	0	Low-quality	6.51
Sample_C8C_k141_104241	8667	10	4	0	Low-quality	6.51
Sample_T3C_k141_88389_fragment_1	23010	25	3	8	Low-quality	6.51
Sample_C11B_k141_177837	5311	5	0	2	Low-quality	6.5
Sample_C1C_k141_142261	10706	12	1	3	Low-quality	6.5
Sample_C2A_k141_170442	5516	3	0	0	Low-quality	6.5
Sample_C2A_k141_163017	11243	12	0	4	Low-quality	6.5
Sample_C8B_k141_73150	13129	15	0	11	Low-quality	6.5
Sample_T2A_k141_132288	5183	8	0	5	Low-quality	6.5
Sample_C6C_k141_96341	11844	15	2	0	Low-quality	6.5
Sample_T12A_k141_147203	5277	11	1	0	Low-quality	6.5
Sample_T2C_k141_209992_fragment_1	5516	8	2	1	Low-quality	6.5
Sample_C5A_k141_267066	10998	8	0	3	Low-quality	6.49
Sample_C8B_k141_85651	5969	6	0	1	Low-quality	6.49
Sample_T16B_k141_588	11328	10	0	7	Low-quality	6.49
Sample_T4A_k141_88325	15219	16	0	11	Low-quality	6.49
Sample_C13B_k141_9273	22821	28	1	0	Low-quality	6.49
Sample_C2A_k141_130670	6356	9	0	2	Low-quality	6.48
Sample_C4B_k141_55259	7342	10	0	1	Low-quality	6.48
Sample_T3C_k141_92659	10316	16	1	3	Low-quality	6.48
Sample_T5C_k141_22533	80333	73	1	25	Low-quality	6.48
Sample_T12A_k141_26845	6339	7	0	0	Low-quality	6.48
Sample_C6B_k141_48144	8533	10	3	4	Low-quality	6.46
Sample_C6A_k141_203777	8913	7	2	0	Low-quality	6.46
Sample_C11C_k141_68428	6022	7	1	1	Low-quality	6.45
Sample_T1C_k141_66419	12408	15	0	9	Low-quality	6.45
Sample_C10A_k141_125906	12143	8	0	7	Low-quality	6.44
Sample_T11A_k141_51679	9144	6	0	3	Low-quality	6.44
Sample_T4A_k141_113682	8411	6	0	4	Low-quality	6.44
Sample_C2A_k141_121824	6961	6	0	2	Low-quality	6.43
Sample_C4B_k141_94901	8040	7	0	7	Low-quality	6.43
Sample_C4B_k141_130269	5402	5	1	0	Low-quality	6.43
Sample_C5A_k141_124454	6343	3	0	1	Low-quality	6.43
Sample_T11C_k141_79037	5876	5	0	1	Low-quality	6.43
Sample_T6C_k141_220494	5095	2	0	0	Low-quality	6.42
Sample_T6C_k141_88902	11479	11	0	10	Low-quality	6.42
Sample_C2B_k141_165686	5795	4	3	0	Low-quality	6.42
Sample_C1C_k141_52253	11186	8	0	6	Low-quality	6.41

Sample_T2B_k141_318367	10980	10	1	1	Low-quality	6.41
Sample_T6C_k141_19294	6455	4	0	2	Low-quality	6.41
Sample_C3A_k141_47200	12418	20	1	1	Low-quality	6.41
Sample_T10B_k141_185420	5046	9	3	0	Low-quality	6.41
Sample_T13C_k141_105911	13900	9	0	7	Low-quality	6.4
Sample_T6B_k141_17612	11718	20	2	0	Low-quality	6.4
Sample_C1A_k141_117973	5285	10	3	0	Low-quality	6.4
Sample_C4C_k141_107080	5244	6	0	1	Low-quality	6.39
Sample_T2A_k141_35570	7164	7	2	0	Low-quality	6.39
Sample_T8C_k141_77304_fragment_3	6596	9	3	0	Low-quality	6.39
Sample_T8C_k141_66708_fragment_1	13639	17	3	0	Low-quality	6.39
Sample_C7A_k141_17461	9828	10	1	1	Low-quality	6.38
Sample_T11A_k141_61940	6062	5	0	2	Low-quality	6.38
Sample_T12A_k141_279094	5718	8	0	3	Low-quality	6.38
Sample_T12B_k141_14037	9968	19	1	0	Low-quality	6.38
Sample_T7B_k141_32539	5627	8	0	0	Low-quality	6.38
Sample_C7A_k141_128476	9059	6	0	4	Low-quality	6.37
Sample_T13A_k141_587	8501	7	0	4	Low-quality	6.37
Sample_T17B_k141_36719	8399	7	1	0	Low-quality	6.37
Sample_T6C_k141_210287	11118	11	0	8	Low-quality	6.37
Sample_T6B_k141_398	8684	18	3	2	Low-quality	6.37
Sample_C10A_k141_137735	13184	12	0	7	Low-quality	6.36
Sample_C6B_k141_176984	11110	12	0	11	Low-quality	6.36
Sample_C11B_k141_9298	6604	13	4	0	Low-quality	6.36
Sample_T10B_k141_92357_fragment_1	11823	18	1	4	Low-quality	6.36
Sample_C6B_k141_124950	12385	14	0	12	Low-quality	6.35
Sample_C6C_k141_210220	7899	9	0	5	Low-quality	6.35
Sample_T4B_k141_312618	5142	6	0	1	Low-quality	6.35
Sample_T8C_k141_41560	5169	10	3	0	Low-quality	6.35
Sample_C11B_k141_13839	6756	4	0	0	Low-quality	6.34
Sample_C1B_k141_29464	11413	14	0	6	Low-quality	6.34
Sample_T12B_k141_88953	8776	21	0	1	Low-quality	6.34
Sample_T4B_k141_229710	16293	13	2	3	Low-quality	6.34
Sample_T10A_k141_81928	5911	12	0	0	Low-quality	6.34
Sample_T5A_k141_50981	5972	8	5	0	Low-quality	6.34
Sample_C1B_k141_72928	6867	6	0	1	Low-quality	6.33
Sample_C2C_k141_191719	5481	6	1	0	Low-quality	6.33
Sample_C4C_k141_140024	5316	10	1	0	Low-quality	6.33
Sample_T13C_k141_28080	9778	21	1	1	Low-quality	6.33

Sample_T1A_k141_29226	5504	10	1	0	Low-quality	6.33
Sample_T14C_k141_150497	5163	6	0	1	Low-quality	6.32
Sample_T2C_k141_99493	9151	4	1	0	Low-quality	6.32
Sample_T8B_k141_232599	5167	7	0	4	Low-quality	6.32
Sample_C4C_k141_120851	8750	8	1	1	Low-quality	6.32
Sample_C12A_k141_94139	13595	17	1	3	Low-quality	6.31
Sample_C13B_k141_38612	5156	6	0	2	Low-quality	6.31
Sample_C8A_k141_57548	6585	8	0	1	Low-quality	6.31
Sample_T16A_k141_100048	5972	4	0	3	Low-quality	6.31
Sample_T7A_k141_159995	6828	4	0	0	Low-quality	6.31
Sample_T7B_k141_42039	6196	13	0	0	Low-quality	6.31
Sample_T17C_k141_17232	7353	10	1	1	Low-quality	6.31
Sample_T10B_k141_85435	11000	8	0	7	Low-quality	6.3
Sample_C13B_k141_95070	9890	17	2	3	Low-quality	6.3
Sample_C13B_k141_79811	11534	12	1	1	Low-quality	6.3
Sample_C2C_k141_268223	6928	8	2	1	Low-quality	6.3
Sample_C4B_k141_233372	5771	13	2	0	Low-quality	6.3
Sample_T6A_k141_287364	5717	8	1	0	Low-quality	6.3
Sample_T17A_k141_1503	11597	19	1	1	Low-quality	6.29
Sample_T5C_k141_191772	7354	6	0	0	Low-quality	6.29
Sample_C5A_k141_249981	6637	6	0	1	Low-quality	6.29
Sample_T12A_k141_332037	6317	9	2	0	Low-quality	6.29
Sample_T4B_k141_99501	5800	9	3	0	Low-quality	6.29
Sample_C3B_k141_167758	5539	5	1	3	Low-quality	6.28
Sample_C4A_k141_201955	7171	7	0	3	Low-quality	6.28
Sample_C9A_k141_126315	8075	12	8	2	Low-quality	6.28
Sample_T2C_k141_257120	6813	16	0	0	Low-quality	6.28
Sample_T12C_k141_34644	77967	70	1	49	Low-quality	6.27
Sample_T7C_k141_37289	6051	7	0	0	Low-quality	6.27
Sample_C10B_k141_74730	9097	7	0	7	Low-quality	6.26
Sample_C3B_k141_90408	12849	8	1	7	Low-quality	6.26
Sample_T14A_k141_69567	12479	18	2	4	Low-quality	6.26
Sample_C11B_k141_229644	7752	8	0	3	Low-quality	6.26
Sample_T2B_k141_185497	8908	10	2	0	Low-quality	6.26
Sample_C5A_k141_58642	10905	12	0	10	Low-quality	6.25
Sample_T8B_k141_198806	7288	6	0	6	Low-quality	6.25
Sample_C10A_k141_244689	6185	6	0	1	Low-quality	6.25
Sample_T5A_k141_13676_fragment_1	9048	11	1	2	Low-quality	6.25
Sample_C11B_k141_240360	6787	6	0	1	Low-quality	6.24

Sample_T11B_k141_58768	5415	3	2	0	Low-quality	6.24
Sample_T12A_k141_145491	11300	9	0	5	Low-quality	6.24
Sample_T15B_k141_76912	6061	4	1	0	Low-quality	6.24
Sample_T15A_k141_149440	9929	13	0	0	Low-quality	6.24
Sample_T5B_k141_25775_fragment_1	6690	8	1	1	Low-quality	6.24
Sample_C11A_k141_151367	5093	6	0	5	Low-quality	6.23
Sample_T15B_k141_66621	9622	13	1	2	Low-quality	6.23
Sample_C1C_k141_34463	13162	6	1	5	Low-quality	6.22
Sample_C5C_k141_75603	5940	4	0	0	Low-quality	6.22
Sample_C11B_k141_183091	8199	7	0	5	Low-quality	6.21
Sample_C14C_k141_19193	5979	6	1	2	Low-quality	6.21
Sample_C7B_k141_71739	7331	13	0	0	Low-quality	6.21
Sample_C8B_k141_64155	5587	4	0	0	Low-quality	6.21
Sample_T14B_k141_174776	10834	10	0	8	Low-quality	6.21
Sample_T16B_k141_73809	6668	7	0	2	Low-quality	6.21
Sample_T2A_k141_142353	5410	1	0	0	Low-quality	6.21
Sample_T8C_k141_115326	5620	2	0	0	Low-quality	6.21
Sample_C14C_k141_2328	9258	8	0	5	Low-quality	6.2
Sample_C2A_k141_96958	5076	6	0	1	Low-quality	6.2
Sample_C4C_k141_133468	6452	17	1	1	Low-quality	6.2
Sample_T2B_k141_26673	10804	11	1	0	Low-quality	6.2
Sample_C14A_k141_192406	6060	4	0	2	Low-quality	6.19
Sample_C2A_k141_174544	28842	23	1	6	Low-quality	6.19
Sample_C3A_k141_202157	6693	5	0	2	Low-quality	6.19
Sample_T7C_k141_70918	7325	9	0	2	Low-quality	6.19
Sample_C13C_k141_99277	10499	24	1	2	Low-quality	6.19
Sample_C2C_k141_175511	6547	11	8	0	Low-quality	6.19
Sample_T8B_k141_221528	10170	15	1	2	Low-quality	6.19
Sample_C12B_k141_181024	9283	9	0	6	Low-quality	6.18
Sample_C14C_k141_99224	12226	14	0	7	Low-quality	6.18
Sample_C4A_k141_45912	6513	5	0	4	Low-quality	6.18
Sample_C5A_k141_290048	10789	10	0	7	Low-quality	6.18
Sample_C7C_k141_8699	10636	12	2	0	Low-quality	6.18
Sample_C9A_k141_94711	5002	10	3	1	Low-quality	6.18
Sample_C2C_k141_262535	38400	38	0	26	Low-quality	6.17
Sample_T14B_k141_159228	5671	9	0	0	Low-quality	6.17
Sample_T12B_k141_127068	10114	24	1	1	Low-quality	6.17
Sample_T14A_k141_117960_fragment_2	8589	10	1	1	Low-quality	6.17
Sample_T18B_k141_124637	5081	12	5	0	Low-quality	6.17

Sample_T7A_k141_65287	6793	8	0	0	Low-quality	6.17
Sample_C13A_k141_71510	7118	4	1	0	Low-quality	6.16
Sample_C6B_k141_95572	12746	12	0	11	Low-quality	6.16
Sample_C9C_k141_73102	10831	13	3	0	Low-quality	6.16
Sample_C12C_k141_239813	9652	20	1	0	Low-quality	6.15
Sample_C9B_k141_108077	5912	10	5	0	Low-quality	6.15
Sample_C14C_k141_82553	15025	14	0	13	Low-quality	6.14
Sample_C1C_k141_104806	5919	5	0	1	Low-quality	6.14
Sample_C1C_k141_149246	7786	9	2	1	Low-quality	6.14
Sample_C4B_k141_143864	5334	3	2	0	Low-quality	6.14
Sample_T13C_k141_98234_fragment_1	17258	20	2	3	Low-quality	6.14
Sample_C3C_k141_111631	5652	4	0	3	Low-quality	6.13
Sample_C4B_k141_263786	7083	4	1	0	Low-quality	6.13
Sample_T6A_k141_282385	63163	44	2	22	Low-quality	6.13
Sample_T15C_k141_58649	5776	5	0	0	Low-quality	6.12
Sample_T8A_k141_128874	11134	11	0	6	Low-quality	6.12
Sample_T8B_k141_253335	6223	9	0	4	Low-quality	6.12
Sample_C7A_k141_103978	5038	8	1	1	Low-quality	6.12
Sample_T10A_k141_58072	5099	10	1	0	Low-quality	6.12
Sample_C14C_k141_10373	5090	11	0	0	Low-quality	6.11
Sample_C5A_k141_92118	13161	27	0	0	Low-quality	6.11
Sample_C5A_k141_15628	6141	2	0	0	Low-quality	6.11
Sample_C6A_k141_212089	6265	5	1	0	Low-quality	6.11
Sample_C5A_k141_39464	8417	8	2	0	Low-quality	6.11
Sample_T8A_k141_201176	19073	25	3	3	Low-quality	6.11
Sample_T18A_k141_124155	10350	13	1	1	Low-quality	6.1
Sample_C4A_k141_115866	10551	15	5	1	Low-quality	6.1
Sample_T7B_k141_150153	8794	10	1	1	Low-quality	6.1
Sample_C8B_k141_88620	10039	8	0	5	Low-quality	6.09
Sample_T2C_k141_66801	9740	17	2	1	Low-quality	6.09
Sample_T3A_k141_53421	5142	8	0	2	Low-quality	6.09
Sample_C14B_k141_35211	5977	7	0	0	Low-quality	6.09
Sample_C2B_k141_5737	5885	7	0	2	Low-quality	6.09
Sample_T14A_k141_140233	9548	7	1	3	Low-quality	6.08
Sample_C5A_k141_39500	8389	7	2	0	Low-quality	6.08
Sample_T10A_k141_62288	11074	7	0	1	Low-quality	6.08
Sample_C11A_k141_179996	13168	7	0	4	Low-quality	6.07
Sample_C2C_k141_33618	6711	9	1	1	Low-quality	6.07
Sample_T14A_k141_149074	12151	11	1	9	Low-quality	6.07

Sample_T2B_k141_13023	10727	11	0	4	Low-quality	6.07
Sample_T12A_k141_83286_fragment_1	9823	10	1	0	Low-quality	6.07
Sample_T13A_k141_113285	8228	11	2	0	Low-quality	6.07
Sample_C7B_k141_150332	9364	13	1	2	Low-quality	6.06
Sample_T12C_k141_157507	6540	6	0	2	Low-quality	6.05
Sample_T6A_k141_7255	6522	6	0	1	Low-quality	6.05
Sample_T5B_k141_5727_fragment_1	5094	9	4	2	Low-quality	6.05
Sample_C7B_k141_6395	6182	7	0	1	Low-quality	6.04
Sample_T13B_k141_110511	19742	21	2	5	Low-quality	6.04
Sample_T12A_k141_16471	18087	18	0	7	Low-quality	6.03
Sample_T2A_k141_168032	8646	4	0	0	Low-quality	6.03
Sample_C7C_k141_78560	10736	12	5	0	Low-quality	6.03
Sample_T12A_k141_1632	10508	11	0	10	Low-quality	6.02
Sample_T2B_k141_58433	9520	9	0	6	Low-quality	6.02
Sample_T2C_k141_144989	9375	7	2	0	Low-quality	6.02
Sample_C12B_k141_169143	15845	10	5	0	Low-quality	6.02
Sample_C11A_k141_17511	10372	17	0	1	Low-quality	6.01
Sample_C5C_k141_76253	9344	12	1	1	Low-quality	6.01
Sample_C6A_k141_192889	10669	11	0	8	Low-quality	6.01
Sample_T6B_k141_107903	32564	33	1	17	Low-quality	6.01
Sample_C5A_k141_177046	9850	16	1	4	Low-quality	6
Sample_T3A_k141_3639	9232	7	0	6	Low-quality	6
Sample_T8B_k141_187984	10722	9	1	5	Low-quality	6
Sample_C4B_k141_15284	6045	12	1	1	Low-quality	6
Sample_C5B_k141_74833	5467	7	1	1	Low-quality	6
Sample_C12A_k141_67589	7725	10	2	0	Low-quality	5.99
Sample_T11B_k141_81934	5567	1	0	0	Low-quality	5.99
Sample_C13C_k141_61616	5771	14	4	1	Low-quality	5.99
Sample_T12B_k141_47626	10145	17	4	1	Low-quality	5.99
Sample_C11A_k141_7904	5183	9	1	3	Low-quality	5.98
Sample_C3B_k141_133189	11139	12	0	11	Low-quality	5.98
Sample_T4B_k141_12455_fragment_1	8900	10	1	0	Low-quality	5.98
Sample_T8B_k141_127467	9678	15	6	1	Low-quality	5.98
Sample_C10B_k141_89847	10998	19	1	1	Low-quality	5.97
Sample_C13C_k141_802	5421	4	0	2	Low-quality	5.97
Sample_C1C_k141_116706	11555	13	1	1	Low-quality	5.97
Sample_T11B_k141_9102	19205	18	2	7	Low-quality	5.97
Sample_T17B_k141_10351	8802	18	0	17	Low-quality	5.97
Sample_C11B_k141_32068	8788	9	0	0	Low-quality	5.97

Sample_C3A_k141_14719	6518	8	3	0	Low-quality	5.97
Sample_C5A_k141_122242	11613	15	2	0	Low-quality	5.97
Sample_T7A_k141_138664	6527	7	0	1	Low-quality	5.97
Sample_C10A_k141_171684	5511	5	0	3	Low-quality	5.96
Sample_C12A_k141_8505	5712	4	0	0	Low-quality	5.96
Sample_T3C_k141_109966	5368	4	0	1	Low-quality	5.96
Sample_T6A_k141_200456	5337	7	0	1	Low-quality	5.96
Sample_T6A_k141_262169	5164	4	2	0	Low-quality	5.95
Sample_C3B_k141_195083	8583	9	1	0	Low-quality	5.95
Sample_C3C_k141_130343	6105	10	0	1	Low-quality	5.95
Sample_T5A_k141_34504_fragment_1	14735	19	1	1	Low-quality	5.95
Sample_C5A_k141_118682	5488	6	0	1	Low-quality	5.94
Sample_T5B_k141_31501	6863	4	1	0	Low-quality	5.94
Sample_T16C_k141_137041	10245	18	3	0	Low-quality	5.94
Sample_C2C_k141_133078	5792	8	0	1	Low-quality	5.93
Sample_C6C_k141_226895	10359	11	0	10	Low-quality	5.93
Sample_T14C_k141_21946	17474	20	0	8	Low-quality	5.93
Sample_T4C_k141_102685	10933	16	1	0	Low-quality	5.93
Sample_T8A_k141_132137	6324	7	1	0	Low-quality	5.93
Sample_C2C_k141_146620	5663	3	0	0	Low-quality	5.92
Sample_T14B_k141_109044	6371	5	0	3	Low-quality	5.92
Sample_T18B_k141_122170	6846	4	1	0	Low-quality	5.92
Sample_T4C_k141_40881	8805	14	1	1	Low-quality	5.92
Sample_C4B_k141_238529	10625	21	1	0	Low-quality	5.92
Sample_C11B_k141_41172	5594	4	0	2	Low-quality	5.91
Sample_T12A_k141_183614	5613	7	0	2	Low-quality	5.91
Sample_T12C_k141_139971	9197	19	1	0	Low-quality	5.91
Sample_T12C_k141_30216	6109	4	0	1	Low-quality	5.91
Sample_T16C_k141_45138	5783	10	0	2	Low-quality	5.91
Sample_T7B_k141_84573	5363	8	0	1	Low-quality	5.91
Sample_T14C_k141_165290	6259	12	1	0	Low-quality	5.91
Sample_T14A_k141_195534	9118	15	1	1	Low-quality	5.9
Sample_T16A_k141_32814	15586	12	1	4	Low-quality	5.9
Sample_C11B_k141_234660	6210	11	2	0	Low-quality	5.9
Sample_C5C_k141_18105	7769	8	0	3	Low-quality	5.9
Sample_T12C_k141_18322	5565	10	1	0	Low-quality	5.9
Sample_T17B_k141_24988	9457	14	3	2	Low-quality	5.9
Sample_C4A_k141_164157	10933	32	0	0	Low-quality	5.89
Sample_C14A_k141_34616	10449	11	4	0	Low-quality	5.89

Sample_T3B_k141_33436	12767	18	1	0	Low-quality	5.88
Sample_T12A_k141_85598	9508	7	2	2	Low-quality	5.88
Sample_T12C_k141_104826	10125	13	3	1	Low-quality	5.88
Sample_T16B_k141_124446	10638	12	3	0	Low-quality	5.88
Sample_C11C_k141_186211	9961	13	1	1	Low-quality	5.87
Sample_T6A_k141_300796	8969	11	1	0	Low-quality	5.86
Sample_C4C_k141_212056	6229	10	2	0	Low-quality	5.86
Sample_C6C_k141_55527	8602	8	0	0	Low-quality	5.86
Sample_C8B_k141_159072	5457	10	1	1	Low-quality	5.86
Sample_T13B_k141_175430	8858	12	2	0	Low-quality	5.85
Sample_C10C_k141_43508	10202	13	0	10	Low-quality	5.84
Sample_C10C_k141_122755	5145	7	5	0	Low-quality	5.84
Sample_C4B_k141_58815	8138	10	0	4	Low-quality	5.83
Sample_T4B_k141_280465	8115	6	0	5	Low-quality	5.83
Sample_C6A_k141_128017	9898	16	1	0	Low-quality	5.83
Sample_C7B_k141_38643	6167	9	0	0	Low-quality	5.83
Sample_C7B_k141_50291	5549	4	0	0	Low-quality	5.82
Sample_T18C_k141_44566	6019	1	0	0	Low-quality	5.82
Sample_C4A_k141_113210	8623	8	0	1	Low-quality	5.82
Sample_C4B_k141_147348	9713	24	0	0	Low-quality	5.82
Sample_C4B_k141_112958_fragment_3	9047	9	1	1	Low-quality	5.82
Sample_T18B_k141_115071_fragment_1	8074	11	1	1	Low-quality	5.82
Sample_T5B_k141_67803	6406	12	1	0	Low-quality	5.82
Sample_C4C_k141_187112	6222	6	0	1	Low-quality	5.81
Sample_T10A_k141_106934	8015	7	0	7	Low-quality	5.81
Sample_T14B_k141_113479	7440	6	0	2	Low-quality	5.81
Sample_T8B_k141_40715	17965	13	0	9	Low-quality	5.81
Sample_C1B_k141_89579	6410	6	0	1	Low-quality	5.81
Sample_C4A_k141_166498	8438	9	6	0	Low-quality	5.81
Sample_C6B_k141_95257	5489	7	2	0	Low-quality	5.81
Sample_T16B_k141_85130	10275	29	1	0	Low-quality	5.81
Sample_C3A_k141_40306	6270	11	3	0	Low-quality	5.8
Sample_T12A_k141_313643	5492	5	1	0	Low-quality	5.8
Sample_T14A_k141_233342	5131	7	2	1	Low-quality	5.8
Sample_C6B_k141_104251	5815	3	0	3	Low-quality	5.79
Sample_T3C_k141_108704	11813	11	0	10	Low-quality	5.79
Sample_T4B_k141_106131	31661	57	1	0	Low-quality	5.79
Sample_T7C_k141_27307	5306	8	1	1	Low-quality	5.79
Sample_C3C_k141_72993	8383	13	1	1	Low-quality	5.79

Sample_C9A_k141_55652	5814	11	2	0	Low-quality	5.79
Sample_C8B_k141_201605	5502	7	1	0	Low-quality	5.78
Sample_T1A_k141_9188	6986	7	1	2	Low-quality	5.78
Sample_C9C_k141_12568	10271	17	2	0	Low-quality	5.78
Sample_T16C_k141_125926	8180	11	0	0	Low-quality	5.78
Sample_C14C_k141_3451	13274	18	2	7	Low-quality	5.77
Sample_C6C_k141_71112	16427	12	0	8	Low-quality	5.77
Sample_C9C_k141_62570	10637	19	1	2	Low-quality	5.77
Sample_T12A_k141_1120	5054	10	1	1	Low-quality	5.77
Sample_T6A_k141_179861	8262	17	3	0	Low-quality	5.77
Sample_T6A_k141_62339	8578	8	1	0	Low-quality	5.76
Sample_T7A_k141_193254	5292	8	2	2	Low-quality	5.76
Sample_T10A_k141_118966	9257	8	0	5	Low-quality	5.75
Sample_T13C_k141_108117	7712	3	0	0	Low-quality	5.75
Sample_T10A_k141_135733	7991	14	0	0	Low-quality	5.75
Sample_T14A_k141_180713	5507	6	0	1	Low-quality	5.75
Sample_T2B_k141_141437	5394	9	1	0	Low-quality	5.75
Sample_C13B_k141_68826	7971	8	1	0	Low-quality	5.74
Sample_C8B_k141_42594	5815	7	2	1	Low-quality	5.74
Sample_T10A_k141_151322	7837	8	0	8	Low-quality	5.73
Sample_T14A_k141_123914	7701	7	0	6	Low-quality	5.73
Sample_T6A_k141_100167	7230	7	0	6	Low-quality	5.73
Sample_T6A_k141_185932	6215	6	0	3	Low-quality	5.73
Sample_C6A_k141_64571	5549	5	0	2	Low-quality	5.72
Sample_T15A_k141_40256	9432	12	1	3	Low-quality	5.72
Sample_T6A_k141_147478	9857	15	1	0	Low-quality	5.72
Sample_T12C_k141_172006	8121	6	0	3	Low-quality	5.71
Sample_C10A_k141_243930	5144	17	1	0	Low-quality	5.71
Sample_C4B_k141_162656	5597	3	0	1	Low-quality	5.7
Sample_T6A_k141_43688	9401	18	2	2	Low-quality	5.7
Sample_T8A_k141_193928	18467	14	0	13	Low-quality	5.7
Sample_C10A_k141_183279	10117	11	0	7	Low-quality	5.69
Sample_T11A_k141_35213	9925	12	0	11	Low-quality	5.69
Sample_T12A_k141_60003	11070	11	0	8	Low-quality	5.69
Sample_T14A_k141_39648	5030	5	1	0	Low-quality	5.69
Sample_T4B_k141_227592	7791	10	1	0	Low-quality	5.69
Sample_C1A_k141_259433	5224	2	0	0	Low-quality	5.68
Sample_T4A_k141_78792	7899	8	0	0	Low-quality	5.68
Sample_T7C_k141_159017	8763	13	1	0	Low-quality	5.68

Sample_T8B_k141_158759	9432	8	4	1	Low-quality	5.68
Sample_C1C_k141_43461	8089	8	0	5	Low-quality	5.67
Sample_T6C_k141_93093	5492	5	0	2	Low-quality	5.67
Sample_T7A_k141_168659	5675	2	0	0	Low-quality	5.67
Sample_T8B_k141_55742	7709	8	1	1	Low-quality	5.67
Sample_T7A_k141_84034	9670	11	1	4	Low-quality	5.66
Sample_T7B_k141_80654	5660	5	0	2	Low-quality	5.66
Sample_C11A_k141_2151	8193	14	0	0	Low-quality	5.66
Sample_T6A_k141_88389	5813	17	1	0	Low-quality	5.66
Sample_T8C_k141_211713	8479	10	1	0	Low-quality	5.66
Sample_C12C_k141_180549	5607	6	1	1	Low-quality	5.65
Sample_C13A_k141_66674	7199	9	1	1	Low-quality	5.65
Sample_C1C_k141_4907	8551	5	0	5	Low-quality	5.65
Sample_C7A_k141_91787	12304	7	0	3	Low-quality	5.65
Sample_T11C_k141_131401	6348	10	1	1	Low-quality	5.65
Sample_T16B_k141_43015	7385	9	0	2	Low-quality	5.65
Sample_C1B_k141_175950	11523	10	0	9	Low-quality	5.64
Sample_C5C_k141_112500	7326	9	0	3	Low-quality	5.64
Sample_T12A_k141_94068	5295	6	0	2	Low-quality	5.64
Sample_T17B_k141_28813	9844	11	0	8	Low-quality	5.64
Sample_C2A_k141_81299_fragment_1	11477	12	1	3	Low-quality	5.64
Sample_C6C_k141_195783	5819	10	0	1	Low-quality	5.64
Sample_C9A_k141_103803	7832	13	1	0	Low-quality	5.64
Sample_C10A_k141_151787	16521	23	3	2	Low-quality	5.63
Sample_C10C_k141_865	17832	17	0	11	Low-quality	5.63
Sample_C2C_k141_74746	70059	66	1	43	Low-quality	5.63
Sample_T2A_k141_11158	13381	7	1	2	Low-quality	5.63
Sample_T5C_k141_153862	7816	9	1	5	Low-quality	5.63
Sample_C11B_k141_60355	8868	13	1	0	Low-quality	5.63
Sample_T10A_k141_50105	7529	19	4	0	Low-quality	5.63
Sample_T2A_k141_213677	9558	9	0	1	Low-quality	5.63
Sample_T1B_k141_10439	5339	4	0	2	Low-quality	5.62
Sample_T14A_k141_207808	7859	10	0	1	Low-quality	5.61
Sample_T8A_k141_244128	5645	10	0	2	Low-quality	5.61
Sample_T8C_k141_176517	5324	3	0	0	Low-quality	5.61
Sample_C1A_k141_66798	5538	9	1	1	Low-quality	5.6
Sample_C6B_k141_54856	11525	24	1	0	Low-quality	5.6
Sample_T7A_k141_53443_fragment_1	6975	13	1	1	Low-quality	5.6
Sample_C2C_k141_121257	40563	36	3	12	Low-quality	5.59

Sample_T13C_k141_96503	5945	8	1	2	Low-quality	5.59
Sample_T14B_k141_133615	8522	10	1	3	Low-quality	5.59
Sample_C2A_k141_74994_fragment_1	8313	8	1	0	Low-quality	5.59
Sample_C7B_k141_128760	5251	8	0	2	Low-quality	5.59
Sample_C2B_k141_102882_fragment_1	8492	8	1	0	Low-quality	5.58
Sample_T8B_k141_77602	5690	4	0	0	Low-quality	5.58
Sample_C3A_k141_121633	12937	14	1	2	Low-quality	5.57
Sample_T14A_k141_22954	9719	8	0	7	Low-quality	5.57
Sample_C2B_k141_265687	10248	12	1	0	Low-quality	5.56
Sample_C6B_k141_138401	5850	5	0	2	Low-quality	5.56
Sample_T15A_k141_117992	11318	11	0	10	Low-quality	5.56
Sample_T5A_k141_9006	5172	7	1	0	Low-quality	5.56
Sample_C10C_k141_146886	21075	24	1	6	Low-quality	5.55
Sample_C2B_k141_152664	7347	9	0	4	Low-quality	5.55
Sample_T16C_k141_40880	6503	3	0	3	Low-quality	5.55
Sample_T10A_k141_57061	11294	24	2	2	Low-quality	5.54
Sample_T2A_k141_136546	9646	21	1	0	Low-quality	5.54
Sample_T8B_k141_67698	8647	10	0	5	Low-quality	5.54
Sample_C2C_k141_116597	8341	12	2	1	Low-quality	5.53
Sample_T18C_k141_108467	5252	4	0	2	Low-quality	5.53
Sample_T13C_k141_141773	8326	11	1	0	Low-quality	5.53
Sample_T16B_k141_173465	9767	26	6	0	Low-quality	5.53
Sample_T8B_k141_41265	9544	8	5	0	Low-quality	5.53
Sample_C11B_k141_59209	11259	11	1	1	Low-quality	5.52
Sample_C4A_k141_6337	18830	19	0	12	Low-quality	5.52
Sample_C6C_k141_77628	6006	6	0	3	Low-quality	5.52
Sample_C2C_k141_9824	5833	10	0	0	Low-quality	5.52
Sample_C2C_k141_25044	10423	9	0	8	Low-quality	5.51
Sample_T14A_k141_1994	150220	160	6	117	Low-quality	5.51
Sample_T3C_k141_89119	5608	2	0	0	Low-quality	5.51
Sample_T5C_k141_97075	8164	10	1	1	Low-quality	5.51
Sample_T8B_k141_75205	9600	8	1	0	Low-quality	5.51
Sample_C11B_k141_133662	8184	15	1	4	Low-quality	5.5
Sample_C2B_k141_242729	5624	6	0	0	Low-quality	5.5
Sample_T14C_k141_120426	11217	16	1	2	Low-quality	5.5
Sample_T16C_k141_68974	8025	7	0	2	Low-quality	5.5
Sample_T2C_k141_10321	6012	11	0	2	Low-quality	5.5
Sample_T5B_k141_28373	13343	19	2	1	Low-quality	5.5
Sample_C10A_k141_227248	8596	6	0	1	Low-quality	5.49

Sample_C13A_k141_38330	7514	7	0	1	Low-quality	5.49
Sample_C13A_k141_35022	5154	10	0	0	Low-quality	5.49
Sample_T1B_k141_4696	12647	14	1	4	Low-quality	5.49
Sample_C7C_k141_2697_fragment_1	7243	11	1	0	Low-quality	5.49
Sample_C11B_k141_222377	15283	16	0	9	Low-quality	5.48
Sample_C1A_k141_80477	5113	14	0	0	Low-quality	5.48
Sample_C6C_k141_78302	6018	7	1	3	Low-quality	5.48
Sample_T10B_k141_110229	7273	11	3	0	Low-quality	5.48
Sample_C2C_k141_176455	9689	7	1	0	Low-quality	5.47
Sample_C1B_k141_82783	9525	9	0	7	Low-quality	5.46
Sample_T18C_k141_48293	11384	6	1	1	Low-quality	5.46
Sample_T4A_k141_61851	5128	7	0	1	Low-quality	5.46
Sample_C14A_k141_172055	8204	9	0	0	Low-quality	5.45
Sample_C3A_k141_86237	7379	8	0	3	Low-quality	5.45
Sample_C6C_k141_28747	10792	9	0	8	Low-quality	5.45
Sample_T16A_k141_29075	8098	6	1	0	Low-quality	5.45
Sample_T16C_k141_91814	5317	8	0	0	Low-quality	5.45
Sample_T2C_k141_178950	7807	9	2	0	Low-quality	5.45
Sample_T4B_k141_19299	9341	7	0	5	Low-quality	5.45
Sample_T7C_k141_153389	7378	23	0	0	Low-quality	5.45
Sample_T8C_k141_199381	5406	8	0	2	Low-quality	5.45
Sample_C2C_k141_254578	5860	5	0	1	Low-quality	5.44
Sample_C6C_k141_100174	5882	3	0	2	Low-quality	5.44
Sample_T10B_k141_69438	5006	1	0	0	Low-quality	5.44
Sample_C13A_k141_114109	5711	11	3	0	Low-quality	5.44
Sample_T8C_k141_98421	7181	13	1	0	Low-quality	5.44
Sample_C2A_k141_287083	5541	5	0	3	Low-quality	5.43
Sample_T13C_k141_44390	8412	8	0	7	Low-quality	5.43
Sample_T16C_k141_90138_fragment_1	8445	8	1	0	Low-quality	5.43
Sample_T4B_k141_18156_fragment_2	12148	10	0	3	Low-quality	5.43
Sample_T6B_k141_159948	9994	15	1	1	Low-quality	5.42
Sample_C5A_k141_144955	9044	16	3	1	Low-quality	5.42
Sample_T14A_k141_9461	5182	8	0	1	Low-quality	5.42
Sample_C4B_k141_131879	8846	15	1	1	Low-quality	5.41
Sample_C7A_k141_2823	8062	9	1	1	Low-quality	5.41
Sample_C1B_k141_32558	16579	12	0	5	Low-quality	5.4
Sample_C3C_k141_183908	5919	6	1	0	Low-quality	5.4
Sample_C5C_k141_67365	10756	9	0	4	Low-quality	5.4
Sample_T13A_k141_7897	7776	9	0	5	Low-quality	5.4

Sample_T10C_k141_15422	7798	6	1	0	Low-quality	5.39
Sample_C4B_k141_224127	9584	11	1	2	Low-quality	5.38
Sample_T8C_k141_73206	6107	1	0	0	Low-quality	5.38
Sample_C10C_k141_141388	7989	6	0	0	Low-quality	5.38
Sample_C4A_k141_242061	5751	7	0	0	Low-quality	5.38
Sample_T17B_k141_37562_fragment_1	13403	19	3	3	Low-quality	5.38
Sample_C10B_k141_156709	12532	11	1	2	Low-quality	5.37
Sample_C11C_k141_35605	6354	6	4	0	Low-quality	5.37
Sample_C5B_k141_7067	5048	7	0	2	Low-quality	5.37
Sample_T12C_k141_192995	5321	4	0	1	Low-quality	5.37
Sample_T6A_k141_164744	9517	7	1	0	Low-quality	5.37
Sample_C11C_k141_171162	6330	11	1	3	Low-quality	5.36
Sample_T13C_k141_20688	9333	18	0	0	Low-quality	5.36
Sample_T1A_k141_8340	11347	7	0	5	Low-quality	5.36
Sample_C14C_k141_22447	7073	13	1	1	Low-quality	5.36
Sample_C3A_k141_187859	7967	14	1	0	Low-quality	5.36
Sample_T10A_k141_24532	7138	9	0	0	Low-quality	5.36
Sample_C2C_k141_298718	8749	17	1	1	Low-quality	5.35
Sample_C8B_k141_22966	5467	1	0	0	Low-quality	5.35
Sample_T1B_k141_44016	5580	4	0	3	Low-quality	5.35
Sample_T12A_k141_258185	12433	12	1	3	Low-quality	5.35
Sample_C10A_k141_183754	10921	12	0	5	Low-quality	5.34
Sample_T12A_k141_284258	5588	7	1	2	Low-quality	5.34
Sample_T14A_k141_84534_fragment_1	13157	17	1	3	Low-quality	5.34
Sample_T8C_k141_24438	6096	17	3	0	Low-quality	5.34
Sample_C1C_k141_64199	9485	9	0	5	Low-quality	5.33
Sample_C5A_k141_30111	8837	8	0	1	Low-quality	5.33
Sample_T8C_k141_141537	5587	6	0	2	Low-quality	5.33
Sample_C14B_k141_65840	29149	29	1	22	Low-quality	5.32
Sample_C1A_k141_92769	5197	5	0	2	Low-quality	5.32
Sample_C8B_k141_96504	5075	2	0	0	Low-quality	5.32
Sample_T15A_k141_111156	6307	5	0	1	Low-quality	5.32
Sample_T2A_k141_97736	9141	10	0	5	Low-quality	5.32
Sample_T7A_k141_108676	5857	4	0	0	Low-quality	5.32
Sample_T7B_k141_17167	11255	7	0	6	Low-quality	5.32
Sample_T8A_k141_170000	5215	6	0	1	Low-quality	5.32
Sample_C11A_k141_36733	7785	14	2	0	Low-quality	5.32
Sample_C7A_k141_52339	10634	6	1	1	Low-quality	5.32
Sample_T2B_k141_82213	11444	33	3	0	Low-quality	5.32

Sample_C14A_k141_48881	7489	8	1	2	Low-quality	5.31
Sample_C5A_k141_74247	5553	5	0	1	Low-quality	5.31
Sample_C8A_k141_5800	5527	3	0	3	Low-quality	5.31
Sample_T12A_k141_305296	5816	5	1	0	Low-quality	5.31
Sample_C9A_k141_67602	6903	8	0	6	Low-quality	5.3
Sample_C8B_k141_45336	10816	9	1	0	Low-quality	5.3
Sample_C12C_k141_173767	9481	11	1	2	Low-quality	5.29
Sample_T11A_k141_82503	6999	7	1	4	Low-quality	5.29
Sample_T1C_k141_8968	6950	11	0	3	Low-quality	5.29
Sample_C3A_k141_225541_fragment_1	9746	21	1	0	Low-quality	5.29
Sample_T12C_k141_117751	8325	14	2	1	Low-quality	5.29
Sample_C2A_k141_168205	7590	8	0	3	Low-quality	5.28
Sample_C7B_k141_1969	10760	11	0	7	Low-quality	5.28
Sample_T13B_k141_61673	8007	7	0	5	Low-quality	5.28
Sample_C2C_k141_180355	16191	28	1	5	Low-quality	5.27
Sample_T12A_k141_271985	5428	5	0	3	Low-quality	5.26
Sample_C1C_k141_98868_fragment_1	10466	10	0	6	Low-quality	5.26
Sample_T12C_k141_180632	9662	18	9	0	Low-quality	5.26
Sample_C1B_k141_240801	9160	10	0	8	Low-quality	5.25
Sample_C7B_k141_162230	6178	8	3	0	Low-quality	5.25
Sample_T13C_k141_117170	7788	7	0	2	Low-quality	5.25
Sample_T2A_k141_248693	9469	15	4	0	Low-quality	5.25
Sample_C6C_k141_27830	7779	8	0	5	Low-quality	5.24
Sample_C7B_k141_114229	5700	5	0	1	Low-quality	5.24
Sample_C7B_k141_156458	8710	3	0	0	Low-quality	5.24
Sample_T3C_k141_29146	9655	16	1	1	Low-quality	5.24
Sample_C11C_k141_80286	7082	7	1	2	Low-quality	5.24
Sample_C6C_k141_223951_fragment_1	10894	9	1	1	Low-quality	5.24
Sample_C3A_k141_78997	6527	11	1	1	Low-quality	5.22
Sample_C6A_k141_38191	7222	3	0	0	Low-quality	5.22
Sample_T18A_k141_49583	5648	4	0	3	Low-quality	5.22
Sample_T10A_k141_134276	7883	10	1	1	Low-quality	5.22
Sample_T12C_k141_91197	7406	10	1	1	Low-quality	5.22
Sample_C5B_k141_43314	7405	5	0	2	Low-quality	5.21
Sample_C9A_k141_71117	5274	1	0	0	Low-quality	5.2
Sample_T2A_k141_264988	17077	17	2	3	Low-quality	5.2
Sample_C10B_k141_154532	8415	9	0	0	Low-quality	5.2
Sample_C11C_k141_80721	6051	7	0	2	Low-quality	5.2
Sample_T13A_k141_85491	9066	10	0	7	Low-quality	5.19

Sample_C7A_k141_3971	8003	16	2	0	Low-quality	5.19
Sample_T8C_k141_135305	12036	15	1	5	Low-quality	5.18
Sample_C1B_k141_102639	5149	8	1	0	Low-quality	5.18
Sample_T13C_k141_112206	7446	17	2	0	Low-quality	5.18
Sample_C2C_k141_70283	5466	10	2	0	Low-quality	5.17
Sample_C4A_k141_92103	14507	14	1	2	Low-quality	5.17
Sample_C5B_k141_9437	7256	6	0	2	Low-quality	5.17
Sample_T1B_k141_29778	10279	15	2	3	Low-quality	5.17
Sample_T14A_k141_231518	9011	9	0	6	Low-quality	5.16
Sample_T4C_k141_235	8663	6	1	3	Low-quality	5.16
Sample_C12B_k141_193765	5359	9	1	0	Low-quality	5.16
Sample_C5A_k141_244952_fragment_2	19956	23	1	1	Low-quality	5.16
Sample_T12B_k141_99835	7666	10	1	0	Low-quality	5.16
Sample_C12B_k141_183891	5606	6	0	1	Low-quality	5.15
Sample_C3B_k141_205250	8989	11	0	10	Low-quality	5.15
Sample_C13A_k141_112979	9500	13	1	0	Low-quality	5.15
Sample_T14A_k141_152365	6357	7	1	0	Low-quality	5.15
Sample_T6A_k141_192447	8747	17	2	0	Low-quality	5.15
Sample_C1B_k141_3675	21099	27	3	14	Low-quality	5.14
Sample_C11C_k141_108016	6067	12	0	0	Low-quality	5.14
Sample_T6C_k141_103809_fragment_1	9980	17	2	1	Low-quality	5.14
Sample_C12C_k141_113537	5864	7	1	0	Low-quality	5.13
Sample_C2C_k141_91230	9536	9	0	4	Low-quality	5.13
Sample_C3C_k141_120752	6728	9	2	0	Low-quality	5.13
Sample_C5B_k141_25786	11137	22	2	0	Low-quality	5.13
Sample_C13A_k141_85632	9113	9	0	2	Low-quality	5.12
Sample_C6C_k141_143963	10224	8	0	7	Low-quality	5.12
Sample_T15A_k141_159271	5143	5	0	2	Low-quality	5.12
Sample_C1C_k141_123258	5253	8	2	1	Low-quality	5.12
Sample_T11A_k141_90097	6055	7	0	0	Low-quality	5.12
Sample_T6A_k141_205290	8344	13	1	0	Low-quality	5.11
Sample_T8B_k141_128773	5636	7	1	0	Low-quality	5.11
Sample_C2A_k141_59804	11077	10	0	7	Low-quality	5.1
Sample_T16C_k141_146211	5010	9	0	2	Low-quality	5.1
Sample_T7C_k141_34619	5602	3	0	2	Low-quality	5.1
Sample_T8B_k141_48554	5509	4	0	2	Low-quality	5.1
Sample_C12A_k141_192104_fragment_1	11343	9	1	1	Low-quality	5.1
Sample_C5C_k141_37106	6129	5	3	0	Low-quality	5.1
Sample_C5C_k141_8043	10820	9	1	2	Low-quality	5.1

Sample_C10C_k141_91561	12478	13	0	1	Low-quality	5.09
Sample_C12B_k141_124689	15795	12	0	8	Low-quality	5.09
Sample_T11B_k141_115373	9267	12	0	2	Low-quality	5.09
Sample_T2B_k141_11504	5339	3	0	0	Low-quality	5.09
Sample_T2C_k141_250546	7566	8	1	2	Low-quality	5.09
Sample_T5B_k141_77755	38719	33	3	13	Low-quality	5.09
Sample_T3B_k141_135419	5231	5	2	1	Low-quality	5.09
Sample_C11A_k141_56480	9361	13	1	0	Low-quality	5.08
Sample_C2A_k141_32557	8389	14	2	2	Low-quality	5.08
Sample_C7B_k141_66104	6028	8	0	1	Low-quality	5.08
Sample_T11B_k141_52208	7672	10	0	9	Low-quality	5.08
Sample_C1C_k141_143582	14209	16	4	0	Low-quality	5.08
Sample_C11A_k141_170528	10931	14	1	2	Low-quality	5.07
Sample_C9C_k141_82222	7840	11	1	1	Low-quality	5.07
Sample_T11A_k141_48628	7804	7	0	7	Low-quality	5.07
Sample_T7A_k141_115292	17442	18	0	15	Low-quality	5.07
Sample_C2A_k141_131494	6354	6	4	0	Low-quality	5.07
Sample_T12C_k141_145262	9130	15	0	0	Low-quality	5.07
Sample_T7C_k141_16687	6725	7	1	0	Low-quality	5.07
Sample_C10C_k141_177214	12455	16	1	0	Low-quality	5.06
Sample_T5C_k141_83567	8053	11	1	1	Low-quality	5.06
Sample_T8B_k141_14599	7180	8	1	1	Low-quality	5.06
Sample_C10B_k141_30386	15797	15	0	10	Low-quality	5.05
Sample_C4B_k141_201591	10147	16	2	2	Low-quality	5.05
Sample_T4A_k141_32960	5107	5	0	3	Low-quality	5.05
Sample_T13C_k141_33720	7928	10	2	0	Low-quality	5.05
Sample_T2A_k141_177704	6669	5	0	2	Low-quality	5.04
Sample_T4B_k141_142980	15689	12	0	5	Low-quality	5.04
Sample_C11B_k141_195618	12191	11	0	7	Low-quality	5.03
Sample_C1C_k141_164189	9060	7	0	5	Low-quality	5.03
Sample_T14B_k141_113716	7905	13	2	0	Low-quality	5.03
Sample_C1C_k141_66975	9723	10	0	6	Low-quality	5.02
Sample_C3C_k141_103934	7576	19	0	0	Low-quality	5.02
Sample_C4C_k141_147400	5459	4	0	3	Low-quality	5.02
Sample_C9A_k141_40220	10031	16	2	3	Low-quality	5.02
Sample_T7A_k141_214706	5048	10	2	1	Low-quality	5.02
Sample_C2C_k141_247517	8263	7	1	2	Low-quality	5.01
Sample_T10B_k141_169496	7931	10	1	0	Low-quality	5.01
Sample_T12B_k141_138572	5288	7	1	1	Low-quality	5.01

Sample_T14B_k141_164289	5138	4	1	1	Low-quality	5.01
Sample_T15C_k141_79037	6951	10	1	1	Low-quality	5.01
Sample_T16B_k141_114778	5913	13	6	2	Low-quality	5.01
Sample_C7C_k141_81360	7200	4	0	3	Low-quality	5
Sample_T4B_k141_80497	5330	4	0	3	Low-quality	5
Sample_C2A_k141_28996	6292	8	7	0	Low-quality	5
Sample_C5C_k141_14130	6582	14	2	0	Low-quality	5
Sample_T16C_k141_44026	7232	13	5	0	Low-quality	5
Sample_T2B_k141_62478	9962	9	0	7	Low-quality	4.99
Sample_T2C_k141_54296	7103	7	0	2	Low-quality	4.99
Sample_T8A_k141_218692	18194	22	1	4	Low-quality	4.99
Sample_C1B_k141_37330	5702	6	0	0	Low-quality	4.99
Sample_C7A_k141_62226	8168	9	1	0	Low-quality	4.99
Sample_T12A_k141_298566	9170	5	0	1	Low-quality	4.99
Sample_T8B_k141_197838	7708	10	0	0	Low-quality	4.99
Sample_C2C_k141_36035	9603	9	0	4	Low-quality	4.98
Sample_C4C_k141_4039	10156	10	1	2	Low-quality	4.98
Sample_C8C_k141_32349	5073	6	0	4	Low-quality	4.98
Sample_T13A_k141_42700	19258	9	0	7	Low-quality	4.98
Sample_C1C_k141_50145	6070	8	1	3	Low-quality	4.98
Sample_C5A_k141_163747	8314	14	6	0	Low-quality	4.98
Sample_C12C_k141_4753	9561	12	0	11	Low-quality	4.97
Sample_C8C_k141_93247	8430	8	1	2	Low-quality	4.97
Sample_T6A_k141_34802	7297	4	0	4	Low-quality	4.97
Sample_C9A_k141_76016	10718	8	2	0	Low-quality	4.97
Sample_C2C_k141_181642	8179	14	2	1	Low-quality	4.96
Sample_C6C_k141_198299	5163	6	0	2	Low-quality	4.96
Sample_T11A_k141_150156	6656	4	0	4	Low-quality	4.96
Sample_T15A_k141_148149	5050	4	0	1	Low-quality	4.96
Sample_C1B_k141_10437	12976	15	1	2	Low-quality	4.95
Sample_C8B_k141_121146	6358	6	0	1	Low-quality	4.95
Sample_T4C_k141_44364	12023	14	1	5	Low-quality	4.95
Sample_C4B_k141_190773	8962	10	3	1	Low-quality	4.95
Sample_T11B_k141_330	9871	10	1	2	Low-quality	4.94
Sample_T10B_k141_110258	6562	9	2	0	Low-quality	4.94
Sample_C11B_k141_16009	8608	14	1	7	Low-quality	4.93
Sample_C3A_k141_170279	15619	12	0	9	Low-quality	4.93
Sample_T1C_k141_56687	6529	5	0	3	Low-quality	4.93
Sample_T3A_k141_72839	8610	8	0	8	Low-quality	4.93

Sample_T16B_k141_89676	5514	6	2	0	Low-quality	4.93
Sample_T6A_k141_241901	7264	14	2	0	Low-quality	4.93
Sample_C6A_k141_70421	6505	7	0	2	Low-quality	4.92
Sample_T3A_k141_100217	6322	2	1	1	Low-quality	4.92
Sample_T4B_k141_368332	6882	6	0	3	Low-quality	4.92
Sample_T2B_k141_254994	7323	9	1	0	Low-quality	4.92
Sample_T6A_k141_190643	6988	9	4	0	Low-quality	4.92
Sample_C10C_k141_34277	6526	4	0	3	Low-quality	4.91
Sample_T12B_k141_118634	7910	15	1	0	Low-quality	4.91
Sample_T2C_k141_45384	6538	14	0	1	Low-quality	4.91
Sample_T12C_k141_84296	7405	7	3	0	Low-quality	4.91
Sample_C6A_k141_72673	7242	10	1	0	Low-quality	4.9
Sample_C2C_k141_115823	5176	12	1	0	Low-quality	4.9
Sample_C5A_k141_167315	8743	10	1	1	Low-quality	4.9
Sample_C8C_k141_26799	6265	10	1	0	Low-quality	4.9
Sample_T14A_k141_16830	7700	3	0	0	Low-quality	4.89
Sample_C2C_k141_237179	9985	6	1	5	Low-quality	4.88
Sample_T4B_k141_274493	10331	6	0	6	Low-quality	4.88
Sample_C5A_k141_256944	22697	15	1	4	Low-quality	4.87
Sample_C7B_k141_60774	13673	15	1	2	Low-quality	4.87
Sample_T2B_k141_240426	6793	3	2	0	Low-quality	4.87
Sample_T4B_k141_160528	7026	9	0	2	Low-quality	4.87
Sample_T5C_k141_70640	5939	11	0	0	Low-quality	4.87
Sample_T6C_k141_169328	5016	5	1	1	Low-quality	4.87
Sample_T4B_k141_140448	5192	9	1	0	Low-quality	4.87
Sample_T10B_k141_64143	40184	36	2	18	Low-quality	4.86
Sample_T4B_k141_156068	5757	7	0	3	Low-quality	4.86
Sample_T7B_k141_45726	12161	9	0	0	Low-quality	4.86
Sample_C2A_k141_320256	11847	6	1	1	Low-quality	4.86
Sample_C3C_k141_220821	6744	10	1	2	Low-quality	4.86
Sample_C8A_k141_41853	18798	19	1	2	Low-quality	4.86
Sample_C8B_k141_108728	8732	7	1	0	Low-quality	4.86
Sample_C12A_k141_90826	5986	4	0	2	Low-quality	4.85
Sample_C14C_k141_132936	5265	4	0	1	Low-quality	4.85
Sample_C4A_k141_121880	6289	3	0	3	Low-quality	4.84
Sample_T7A_k141_225270	12201	14	1	4	Low-quality	4.84
Sample_C12A_k141_197083	7675	11	1	1	Low-quality	4.83
Sample_C5A_k141_263643	9689	8	1	6	Low-quality	4.83
Sample_T11C_k141_84192	6968	4	0	3	Low-quality	4.83

Sample_T14B_k141_168203	8231	15	0	1	Low-quality	4.83
Sample_T16C_k141_144131	6640	7	0	6	Low-quality	4.83
Sample_T2A_k141_26430	18245	13	0	9	Low-quality	4.83
Sample_T4B_k141_200343	9486	11	0	9	Low-quality	4.83
Sample_C4B_k141_59057	10118	11	2	3	Low-quality	4.83
Sample_C6C_k141_84103	6051	6	0	4	Low-quality	4.82
Sample_T10A_k141_137741	9763	7	0	7	Low-quality	4.81
Sample_C11A_k141_33414	8534	11	2	0	Low-quality	4.81
Sample_T8A_k141_29476	5416	7	1	0	Low-quality	4.81
Sample_C5A_k141_55388	44079	45	2	29	Low-quality	4.8
Sample_T5A_k141_21260	9154	7	1	1	Low-quality	4.8
Sample_C2A_k141_73669	5360	12	1	0	Low-quality	4.8
Sample_T6A_k141_156378	6698	13	1	0	Low-quality	4.8
Sample_T12A_k141_44442	13185	10	1	9	Low-quality	4.79
Sample_T13C_k141_65697	5816	3	0	2	Low-quality	4.79
Sample_T16B_k141_156270	6238	7	0	3	Low-quality	4.79
Sample_T11B_k141_144021	7107	12	1	0	Low-quality	4.78
Sample_C12A_k141_71464	8480	7	0	3	Low-quality	4.77
Sample_C6A_k141_34074	8322	8	0	5	Low-quality	4.77
Sample_T11C_k141_86406	7514	7	0	3	Low-quality	4.77
Sample_T12A_k141_23940	9135	13	0	10	Low-quality	4.77
Sample_T12C_k141_25653	7917	7	0	1	Low-quality	4.77
Sample_T3A_k141_26624	7557	9	1	0	Low-quality	4.77
Sample_C14C_k141_60642	19027	17	0	13	Low-quality	4.76
Sample_C1B_k141_170473	8841	3	0	0	Low-quality	4.76
Sample_C1B_k141_212325	15559	12	0	8	Low-quality	4.76
Sample_T4C_k141_34881	5126	4	0	2	Low-quality	4.76
Sample_T6A_k141_19976	13382	12	1	1	Low-quality	4.76
Sample_C11C_k141_78570	8005	11	1	0	Low-quality	4.76
Sample_C1B_k141_114474	10257	28	0	0	Low-quality	4.76
Sample_C4C_k141_43150	6651	13	1	0	Low-quality	4.76
Sample_T11B_k141_162440_fragment_1	13642	11	1	3	Low-quality	4.76
Sample_C5A_k141_63912	10244	31	0	2	Low-quality	4.75
Sample_T12A_k141_10251	8579	22	0	0	Low-quality	4.75
Sample_T10A_k141_101876	6331	11	0	0	Low-quality	4.74
Sample_T15C_k141_16418	17301	14	1	4	Low-quality	4.74
Sample_T2A_k141_70520	8878	10	0	8	Low-quality	4.74
Sample_C14C_k141_68435	6345	14	0	0	Low-quality	4.74
Sample_C11B_k141_98996	6839	13	2	0	Low-quality	4.73

Sample_T2B_k141_23568	6800	11	0	2	Low-quality	4.73
Sample_T7C_k141_144755	6392	10	1	1	Low-quality	4.73
Sample_C7B_k141_79271	5451	7	0	5	Low-quality	4.72
Sample_T11C_k141_1411	9337	6	0	5	Low-quality	4.72
Sample_T6A_k141_64633	6406	10	0	1	Low-quality	4.72
Sample_C1B_k141_101430	5185	4	1	0	Low-quality	4.72
Sample_T10B_k141_128500	6923	9	0	0	Low-quality	4.72
Sample_T11B_k141_45149	6447	6	3	0	Low-quality	4.72
Sample_C1B_k141_89879	7002	8	1	0	Low-quality	4.71
Sample_C4A_k141_69042	17818	13	0	12	Low-quality	4.71
Sample_C9A_k141_3791	8324	6	4	0	Low-quality	4.71
Sample_C6C_k141_30119	9266	8	0	4	Low-quality	4.7
Sample_C2B_k141_52283	5618	6	0	3	Low-quality	4.7
Sample_C1C_k141_51984	7383	10	1	0	Low-quality	4.68
Sample_C4A_k141_145098	7663	4	1	0	Low-quality	4.68
Sample_C6A_k141_62352	8600	7	0	4	Low-quality	4.68
Sample_T1B_k141_5213	28350	24	2	13	Low-quality	4.68
Sample_C13A_k141_105164	6498	9	4	0	Low-quality	4.68
Sample_C4B_k141_263145_fragment_1	6643	9	0	0	Low-quality	4.68
Sample_C3B_k141_103999	6891	3	0	0	Low-quality	4.67
Sample_T2C_k141_242723	7796	16	1	1	Low-quality	4.67
Sample_T5C_k141_82882	5807	16	0	1	Low-quality	4.67
Sample_C14A_k141_169244	8533	8	0	3	Low-quality	4.66
Sample_T10A_k141_43083	5378	5	0	2	Low-quality	4.66
Sample_C1B_k141_247131	8493	9	2	0	Low-quality	4.66
Sample_C5A_k141_230663	5319	8	2	0	Low-quality	4.66
Sample_T18B_k141_126272	7140	10	1	0	Low-quality	4.66
Sample_C10A_k141_118316	6010	5	0	4	Low-quality	4.65
Sample_C11B_k141_153551	8041	8	0	8	Low-quality	4.65
Sample_C4C_k141_4228	5030	5	0	2	Low-quality	4.65
Sample_T10B_k141_7307	5739	4	0	2	Low-quality	4.65
Sample_T11B_k141_137594	5963	5	0	3	Low-quality	4.65
Sample_T16B_k141_89776	18267	13	0	8	Low-quality	4.65
Sample_T2B_k141_88943	5023	4	0	1	Low-quality	4.65
Sample_C9C_k141_58451	7403	9	1	1	Low-quality	4.65
Sample_C6C_k141_65928	6691	4	0	3	Low-quality	4.64
Sample_T12A_k141_139305	5043	2	0	0	Low-quality	4.64
Sample_C7B_k141_162929	6068	7	0	0	Low-quality	4.64
Sample_C11C_k141_76724	5469	11	0	0	Low-quality	4.63

Sample_C5C_k141_5093	5109	5	0	4	Low-quality	4.63
Sample_T12B_k141_175052	5424	6	1	1	Low-quality	4.63
Sample_T16C_k141_113711	11109	9	2	2	Low-quality	4.63
Sample_T4C_k141_106742	8085	5	0	5	Low-quality	4.63
Sample_T7C_k141_35955	12847	17	1	6	Low-quality	4.63
Sample_C3B_k141_173497	7159	8	1	0	Low-quality	4.63
Sample_C4A_k141_137047	8429	16	1	0	Low-quality	4.63
Sample_T12A_k141_203302	6429	9	0	0	Low-quality	4.63
Sample_T5C_k141_178052	7311	12	1	0	Low-quality	4.63
Sample_C10B_k141_136740	8057	10	0	6	Low-quality	4.62
Sample_C1A_k141_68035	8066	9	0	7	Low-quality	4.62
Sample_T13B_k141_10045	7371	8	1	0	Low-quality	4.62
Sample_T5B_k141_1121	5839	7	0	4	Low-quality	4.62
Sample_T5C_k141_157524	7761	12	1	0	Low-quality	4.62
Sample_T8C_k141_183032	8524	8	1	2	Low-quality	4.62
Sample_C14C_k141_183450	9105	19	2	0	Low-quality	4.62
Sample_C5A_k141_145242	5858	6	1	2	Low-quality	4.61
Sample_T14A_k141_121534	12897	16	1	2	Low-quality	4.61
Sample_T7B_k141_6227	15198	13	0	5	Low-quality	4.61
Sample_C14A_k141_80158	5894	4	1	0	Low-quality	4.61
Sample_C8B_k141_69846	8931	11	1	0	Low-quality	4.61
Sample_T12B_k141_100268	6583	12	0	0	Low-quality	4.61
Sample_T16B_k141_79928	6670	9	1	0	Low-quality	4.61
Sample_T2B_k141_143487	5975	7	0	1	Low-quality	4.61
Sample_C9A_k141_117308	9920	29	0	0	Low-quality	4.6
Sample_T5C_k141_52245	7774	9	1	1	Low-quality	4.6
Sample_T4C_k141_67782	10064	16	4	2	Low-quality	4.6
Sample_T8B_k141_210340	7441	10	0	0	Low-quality	4.6
Sample_C2A_k141_228941	8959	10	0	7	Low-quality	4.59
Sample_C5A_k141_189546	11368	11	1	1	Low-quality	4.59
Sample_C7C_k141_103002	5514	3	0	3	Low-quality	4.59
Sample_C9A_k141_56791	9884	16	1	0	Low-quality	4.59
Sample_T2B_k141_104298	6157	2	1	0	Low-quality	4.59
Sample_T2B_k141_125223	6654	3	0	3	Low-quality	4.59
Sample_T2C_k141_8300	129239	94	2	51	Low-quality	4.59
Sample_T18B_k141_21109	7415	12	5	0	Low-quality	4.59
Sample_T7C_k141_150291	6401	15	1	0	Low-quality	4.59
Sample_C4A_k141_38283	18187	19	0	13	Low-quality	4.58
Sample_C7B_k141_63530	5424	7	0	0	Low-quality	4.58

Sample_T15B_k141_139865	6601	12	0	1	Low-quality	4.57
Sample_C12C_k141_47087_fragment_1	8705	8	0	4	Low-quality	4.57
Sample_T17B_k141_13728	8251	8	0	3	Low-quality	4.56
Sample_C1C_k141_88118_fragment_1	12795	15	2	2	Low-quality	4.56
Sample_T10A_k141_121885_fragment_2	14017	11	1	4	Low-quality	4.56
Sample_T15A_k141_104587	16306	18	1	5	Low-quality	4.55
Sample_T17B_k141_31941	7939	11	0	10	Low-quality	4.55
Sample_T6A_k141_235829	6451	13	1	1	Low-quality	4.55
Sample_C5C_k141_16818	8112	12	1	0	Low-quality	4.54
Sample_T5A_k141_37643	7931	7	0	1	Low-quality	4.54
Sample_C2C_k141_70293	9778	8	1	1	Low-quality	4.54
Sample_T4C_k141_48237	10794	8	0	8	Low-quality	4.53
Sample_C5A_k141_106327	5143	5	2	1	Low-quality	4.53
Sample_C7C_k141_99874	5319	5	0	0	Low-quality	4.53
Sample_C3B_k141_31889	8342	12	1	1	Low-quality	4.52
Sample_T11C_k141_57147	11864	9	1	3	Low-quality	4.52
Sample_T8B_k141_157238	6339	9	1	2	Low-quality	4.52
Sample_C3B_k141_118491	6978	11	1	0	Low-quality	4.52
Sample_T2A_k141_85291	6124	8	0	4	Low-quality	4.51
Sample_T14B_k141_94973	5748	6	0	1	Low-quality	4.51
Sample_T13A_k141_112681	6342	9	0	3	Low-quality	4.5
Sample_C11B_k141_233130	6511	10	1	0	Low-quality	4.5
Sample_C8C_k141_72978	6723	14	0	0	Low-quality	4.5
Sample_T13B_k141_167377	5009	9	1	0	Low-quality	4.5
Sample_T6A_k141_122868	6310	5	2	0	Low-quality	4.5
Sample_C3A_k141_122807	7840	8	0	7	Low-quality	4.49
Sample_C6C_k141_110943	9576	10	0	9	Low-quality	4.49
Sample_C11A_k141_166329	6241	13	5	2	Low-quality	4.49
Sample_C6C_k141_67169	52923	52	1	11	Low-quality	4.48
Sample_T8B_k141_70161	18150	20	2	15	Low-quality	4.48
Sample_T14A_k141_73643	5394	5	0	3	Low-quality	4.47
Sample_T8A_k141_58721	7115	6	1	0	Low-quality	4.47
Sample_C2B_k141_47156	7517	8	0	8	Low-quality	4.46
Sample_T11A_k141_97157	5285	6	0	2	Low-quality	4.46
Sample_T13C_k141_87861	5795	4	0	2	Low-quality	4.46
Sample_T16B_k141_6649	5006	4	0	3	Low-quality	4.46
Sample_T17C_k141_34609	14869	8	0	7	Low-quality	4.46
Sample_T8A_k141_93747	9075	7	0	7	Low-quality	4.46
Sample_C3A_k141_178110	8557	3	1	1	Low-quality	4.45

Sample_C5A_k141_82623	5745	8	0	2	Low-quality	4.45
Sample_C6C_k141_202956	9262	10	0	8	Low-quality	4.45
Sample_T12A_k141_338250	9416	4	0	2	Low-quality	4.45
Sample_T6A_k141_250733	13345	8	0	8	Low-quality	4.45
Sample_T6A_k141_47632	9238	9	1	1	Low-quality	4.45
Sample_T8A_k141_221060	6132	7	0	3	Low-quality	4.45
Sample_C5A_k141_63262	6196	8	1	1	Low-quality	4.45
Sample_T7A_k141_200871	6583	7	0	2	Low-quality	4.45
Sample_C4B_k141_215856	6452	10	8	0	Low-quality	4.44
Sample_T12B_k141_63148	7452	6	4	0	Low-quality	4.44
Sample_T13A_k141_112242	6414	8	0	2	Low-quality	4.44
Sample_T13B_k141_122578	7542	15	4	0	Low-quality	4.44
Sample_C10C_k141_29460	10412	9	0	9	Low-quality	4.43
Sample_C10A_k141_185871	7709	7	0	5	Low-quality	4.42
Sample_C10C_k141_222190	7824	13	1	2	Low-quality	4.42
Sample_T7A_k141_93271	8868	11	0	5	Low-quality	4.42
Sample_C4C_k141_237857	6598	7	1	0	Low-quality	4.42
Sample_C9B_k141_103905	5537	9	3	0	Low-quality	4.42
Sample_T10B_k141_93312	5158	6	1	1	Low-quality	4.42
Sample_C14B_k141_97154	5272	6	1	1	Low-quality	4.41
Sample_C9C_k141_34654	6672	5	1	1	Low-quality	4.41
Sample_T5C_k141_139262	6087	6	1	1	Low-quality	4.41
Sample_C4A_k141_211017	5084	3	1	0	Low-quality	4.4
Sample_T16C_k141_10645	6209	6	1	1	Low-quality	4.4
Sample_C5C_k141_50694	6197	10	0	7	Low-quality	4.39
Sample_C8C_k141_76245	8156	9	2	2	Low-quality	4.39
Sample_T11B_k141_42427	7696	7	0	7	Low-quality	4.39
Sample_C4C_k141_7667	7143	7	1	0	Low-quality	4.39
Sample_C9A_k141_54653	15236	13	0	13	Low-quality	4.38
Sample_T16B_k141_93000	16054	15	0	15	Low-quality	4.38
Sample_T4A_k141_93261	9024	9	0	8	Low-quality	4.38
Sample_T6A_k141_49051	7801	8	0	4	Low-quality	4.38
Sample_C2A_k141_117484	10164	11	1	1	Low-quality	4.38
Sample_T6A_k141_204876	7310	14	4	1	Low-quality	4.38
Sample_T15B_k141_58174	6321	11	2	0	Low-quality	4.37
Sample_T5C_k141_60305	5450	9	1	2	Low-quality	4.37
Sample_T8B_k141_212577	7550	17	2	0	Low-quality	4.37
Sample_C7A_k141_54249	5761	5	0	3	Low-quality	4.36
Sample_T12A_k141_139863	7840	6	0	6	Low-quality	4.36

Sample_T3A_k141_8805	5888	10	0	3	Low-quality	4.36
Sample_C1C_k141_133544	7176	7	3	0	Low-quality	4.36
Sample_C2A_k141_68428	5113	4	0	0	Low-quality	4.36
Sample_C12A_k141_136166	5684	5	0	2	Low-quality	4.35
Sample_T16C_k141_20482	6751	17	0	0	Low-quality	4.35
Sample_C2A_k141_171194	6543	7	1	2	Low-quality	4.35
Sample_T10B_k141_25840	7678	5	0	2	Low-quality	4.34
Sample_C1C_k141_76996	6483	8	0	0	Low-quality	4.34
Sample_T3C_k141_100321	6061	6	2	0	Low-quality	4.34
Sample_T7B_k141_148968	8897	16	4	0	Low-quality	4.34
Sample_C10A_k141_89353	7656	12	1	5	Low-quality	4.33
Sample_C4B_k141_82708	9343	11	1	4	Low-quality	4.33
Sample_C9B_k141_71088	8566	6	0	5	Low-quality	4.33
Sample_C13A_k141_54984	7927	4	1	0	Low-quality	4.33
Sample_C11A_k141_127640	5313	7	0	6	Low-quality	4.32
Sample_C14A_k141_4171	5718	7	1	0	Low-quality	4.32
Sample_T15B_k141_2946	8427	9	1	5	Low-quality	4.32
Sample_C4C_k141_77584	6217	3	0	0	Low-quality	4.31
Sample_T10A_k141_18784	8090	10	1	1	Low-quality	4.31
Sample_T10A_k141_25159	5350	4	0	3	Low-quality	4.31
Sample_C13A_k141_99524	7711	8	1	0	Low-quality	4.3
Sample_T11A_k141_144162	12040	13	0	10	Low-quality	4.3
Sample_T18C_k141_45843	9055	8	0	5	Low-quality	4.3
Sample_T8A_k141_98979	8529	5	0	4	Low-quality	4.3
Sample_C13B_k141_44665	6675	4	1	0	Low-quality	4.3
Sample_C3C_k141_191923	6314	10	0	0	Low-quality	4.3
Sample_T12A_k141_315153	6282	6	0	4	Low-quality	4.29
Sample_T4B_k141_193530	5535	5	0	3	Low-quality	4.29
Sample_T5A_k141_45864	23438	33	1	2	Low-quality	4.29
Sample_C12C_k141_15272	6477	15	5	0	Low-quality	4.29
Sample_T3B_k141_133781	5014	8	2	2	Low-quality	4.29
Sample_T5B_k141_20267	5097	8	5	1	Low-quality	4.29
Sample_C11B_k141_82516	8127	8	1	5	Low-quality	4.28
Sample_T11C_k141_73103	5849	8	1	1	Low-quality	4.27
Sample_T1C_k141_15471	6692	8	0	2	Low-quality	4.27
Sample_T18A_k141_95099	5813	8	5	0	Low-quality	4.27
Sample_T6A_k141_283214	6675	16	1	0	Low-quality	4.27
Sample_C13B_k141_16962	9794	11	1	8	Low-quality	4.26
Sample_C8C_k141_62435	8126	3	0	2	Low-quality	4.26

Sample_C8C_k141_122801	7437	8	0	6	Low-quality	4.26
Sample_T12B_k141_152418	7445	8	0	8	Low-quality	4.26
Sample_T16C_k141_21288	7849	15	1	1	Low-quality	4.26
Sample_T17A_k141_13730	8501	8	1	5	Low-quality	4.26
Sample_T15A_k141_180325	8278	13	2	0	Low-quality	4.26
Sample_C10B_k141_156413	10078	7	0	5	Low-quality	4.25
Sample_T4C_k141_22189	7419	6	0	2	Low-quality	4.25
Sample_T6A_k141_249769	6669	11	1	1	Low-quality	4.25
Sample_T8B_k141_69271	6316	7	1	0	Low-quality	4.25
Sample_C2B_k141_85945	5215	4	0	1	Low-quality	4.24
Sample_C4B_k141_116525	6245	8	1	0	Low-quality	4.24
Sample_T4B_k141_372018	5537	6	1	0	Low-quality	4.24
Sample_C10C_k141_72974	14835	22	1	0	Low-quality	4.24
Sample_C11A_k141_164138	7279	12	2	0	Low-quality	4.24
Sample_T15B_k141_63607	6148	8	4	0	Low-quality	4.24
Sample_C10A_k141_98728	6490	19	0	0	Low-quality	4.23
Sample_T4B_k141_76587	8621	6	0	3	Low-quality	4.23
Sample_T10B_k141_81519	6711	9	1	0	Low-quality	4.22
Sample_T12A_k141_158653	6503	6	0	0	Low-quality	4.22
Sample_T17A_k141_38261	5904	6	0	2	Low-quality	4.22
Sample_T18C_k141_63865	5306	3	0	2	Low-quality	4.22
Sample_T7C_k141_5164	5615	10	4	0	Low-quality	4.22
Sample_C10C_k141_142237	5377	4	0	1	Low-quality	4.21
Sample_T12A_k141_17438	5563	10	0	7	Low-quality	4.21
Sample_C10C_k141_182156_fragment_1	8588	10	0	5	Low-quality	4.21
Sample_T12C_k141_105932	7412	11	3	0	Low-quality	4.21
Sample_T2B_k141_24146	5786	5	3	0	Low-quality	4.21
Sample_T6A_k141_104132	6991	11	2	0	Low-quality	4.21
Sample_C4B_k141_188597	16202	11	0	8	Low-quality	4.2
Sample_C9C_k141_79495	7739	15	1	1	Low-quality	4.2
Sample_C11A_k141_101372_fragment_1	8354	11	3	0	Low-quality	4.2
Sample_C11A_k141_36399	7215	7	3	0	Low-quality	4.2
Sample_C8B_k141_76867	9198	10	1	2	Low-quality	4.2
Sample_T12B_k141_140938	6128	9	6	2	Low-quality	4.2
Sample_T14A_k141_223478	7113	5	3	0	Low-quality	4.2
Sample_T12B_k141_24541	6239	16	1	0	Low-quality	4.19
Sample_C11C_k141_4862_fragment_4	8137	15	2	1	Low-quality	4.19
Sample_C1C_k141_95607	6784	6	1	0	Low-quality	4.19
Sample_C10A_k141_36246	8744	6	1	5	Low-quality	4.18

Sample_C12A_k141_214071	7496	11	1	0	Low-quality	4.18
Sample_C12B_k141_7881	9008	14	1	1	Low-quality	4.18
Sample_C13B_k141_24576	6336	6	0	3	Low-quality	4.18
Sample_T16A_k141_62382	8368	11	1	3	Low-quality	4.18
Sample_T16C_k141_62517	8679	8	1	1	Low-quality	4.18
Sample_C12A_k141_40573	11973	9	0	4	Low-quality	4.18
Sample_T10B_k141_78208	6723	13	2	0	Low-quality	4.18
Sample_C1B_k141_244691	6848	6	0	3	Low-quality	4.17
Sample_C4C_k141_169314	6105	7	0	5	Low-quality	4.17
Sample_C9B_k141_102397_fragment_1	14648	19	1	3	Low-quality	4.17
Sample_T11B_k141_97811	6604	10	1	0	Low-quality	4.17
Sample_T8B_k141_217183	5641	6	1	0	Low-quality	4.17
Sample_T4C_k141_125092	5130	3	0	2	Low-quality	4.16
Sample_T5C_k141_149358	7446	9	0	1	Low-quality	4.16
Sample_T14B_k141_9823	6317	11	0	0	Low-quality	4.16
Sample_T2C_k141_6989	5356	6	0	1	Low-quality	4.16
Sample_C12A_k141_200001	7390	7	0	4	Low-quality	4.15
Sample_T12C_k141_45260	6343	3	3	0	Low-quality	4.15
Sample_T13B_k141_21931	8986	9	1	0	Low-quality	4.15
Sample_T14C_k141_38682	5565	2	0	0	Low-quality	4.15
Sample_C14C_k141_160133	8269	8	2	1	Low-quality	4.14
Sample_T13C_k141_64372	5946	7	0	1	Low-quality	4.14
Sample_T17A_k141_25119	7380	9	0	0	Low-quality	4.14
Sample_T13B_k141_21392	5568	5	0	4	Low-quality	4.13
Sample_T2A_k141_5291	7214	5	0	3	Low-quality	4.13
Sample_T8B_k141_33642	6346	7	0	0	Low-quality	4.13
Sample_C11B_k141_43325	5673	10	2	1	Low-quality	4.13
Sample_T12C_k141_160747	5086	9	1	0	Low-quality	4.13
Sample_T2C_k141_1249	6642	21	2	0	Low-quality	4.13
Sample_C14C_k141_78608	5377	10	1	3	Low-quality	4.12
Sample_C9A_k141_127385	7180	5	1	0	Low-quality	4.12
Sample_C12A_k141_60183	8355	6	0	6	Low-quality	4.11
Sample_C6B_k141_116636	26527	29	2	14	Low-quality	4.11
Sample_T10A_k141_130082	6924	10	1	0	Low-quality	4.11
Sample_T10B_k141_175239	7172	8	0	4	Low-quality	4.11
Sample_T14B_k141_183638	6431	10	1	0	Low-quality	4.11
Sample_T4A_k141_108043	7830	7	0	4	Low-quality	4.11
Sample_C14A_k141_90415	6288	8	2	0	Low-quality	4.11
Sample_C14C_k141_141015	5726	6	0	0	Low-quality	4.11

Sample_C3C_k141_20696	7200	5	1	1	Low-quality	4.1
Sample_C9A_k141_18359	6322	6	0	4	Low-quality	4.1
Sample_C5A_k141_170011	8774	7	2	0	Low-quality	4.1
Sample_C8B_k141_89959	6576	4	2	0	Low-quality	4.1
Sample_T12B_k141_69054	5750	12	3	0	Low-quality	4.1
Sample_T5C_k141_82668	6530	7	0	0	Low-quality	4.1
Sample_C13B_k141_40400	6027	7	0	5	Low-quality	4.09
Sample_T11A_k141_38515	12770	7	0	5	Low-quality	4.09
Sample_T18A_k141_96307	10741	9	1	2	Low-quality	4.09
Sample_T3C_k141_110678	11606	9	0	7	Low-quality	4.09
Sample_C5A_k141_62321	7522	5	0	1	Low-quality	4.08
Sample_C7C_k141_3141	8799	13	1	3	Low-quality	4.08
Sample_T13A_k141_81727	5438	15	0	0	Low-quality	4.08
Sample_T6B_k141_86642	26045	34	3	12	Low-quality	4.08
Sample_T6B_k141_154872	7093	14	0	0	Low-quality	4.08
Sample_C4B_k141_20212	5535	6	4	0	Low-quality	4.08
Sample_C11B_k141_54264	9704	7	0	6	Low-quality	4.07
Sample_C9B_k141_102006	8215	14	0	1	Low-quality	4.07
Sample_T14A_k141_59361	6726	4	0	2	Low-quality	4.07
Sample_T16B_k141_160900	5098	3	0	0	Low-quality	4.07
Sample_C1B_k141_8651	6327	9	1	0	Low-quality	4.07
Sample_C3C_k141_235853	6549	20	3	0	Low-quality	4.07
Sample_T12A_k141_130024	7696	8	2	0	Low-quality	4.07
Sample_C1B_k141_205067	7546	7	1	3	Low-quality	4.06
Sample_C5A_k141_4832	7494	13	1	1	Low-quality	4.06
Sample_C11C_k141_6817	19889	25	2	4	Low-quality	4.06
Sample_C12C_k141_87094	5630	4	0	1	Low-quality	4.05
Sample_T12A_k141_286093	6250	5	0	0	Low-quality	4.05
Sample_C4B_k141_263174	5730	14	3	0	Low-quality	4.05
Sample_T1C_k141_49007	6907	9	1	0	Low-quality	4.05
Sample_C3A_k141_99835	5854	7	0	3	Low-quality	4.04
Sample_C11A_k141_151715	6887	10	3	0	Low-quality	4.04
Sample_T10B_k141_15889	7087	8	1	0	Low-quality	4.03
Sample_T13A_k141_490	6179	10	0	8	Low-quality	4.03
Sample_T3A_k141_102850	6333	11	1	1	Low-quality	4.03
Sample_T8B_k141_172638	6521	4	1	0	Low-quality	4.03
Sample_C2A_k141_72172	5821	7	0	2	Low-quality	4.02
Sample_C5A_k141_39023	5375	6	1	0	Low-quality	4.02
Sample_C7C_k141_7069	7289	14	0	0	Low-quality	4.02

Sample_T16B_k141_78198	14342	14	1	2	Low-quality	4.01
Sample_T4B_k141_40459	5800	7	0	3	Low-quality	4.01
Sample_C1B_k141_41482	6249	7	1	0	Low-quality	4.01
Sample_C10A_k141_245005	12110	15	0	7	Low-quality	4
Sample_C1C_k141_14742	7882	6	0	3	Low-quality	4
Sample_T15A_k141_132634	6975	6	0	5	Low-quality	4
Sample_T3B_k141_69710	6462	10	0	0	Low-quality	4
Sample_C8B_k141_135763	12875	9	0	8	Low-quality	3.99
Sample_T12A_k141_162605	6958	6	0	3	Low-quality	3.99
Sample_T17B_k141_28646	5278	8	1	1	Low-quality	3.99
Sample_T8B_k141_232756	7553	5	0	2	Low-quality	3.99
Sample_C5A_k141_227406	8607	12	2	0	Low-quality	3.99
Sample_C6C_k141_197047	6541	8	1	1	Low-quality	3.98
Sample_T10A_k141_34470	7328	6	0	1	Low-quality	3.98
Sample_T15B_k141_33337	6228	5	0	3	Low-quality	3.98
Sample_T4C_k141_100791	8042	5	0	4	Low-quality	3.98
Sample_C2B_k141_236824	6435	4	1	0	Low-quality	3.98
Sample_T16B_k141_132707	6047	7	0	1	Low-quality	3.98
Sample_T6A_k141_228596	5641	7	3	0	Low-quality	3.98
Sample_C6B_k141_46876	5329	4	0	2	Low-quality	3.97
Sample_C8A_k141_61290	7312	16	1	1	Low-quality	3.97
Sample_C9B_k141_67666	7985	8	0	4	Low-quality	3.97
Sample_T13B_k141_12627	11906	12	0	8	Low-quality	3.97
Sample_T7C_k141_96582	7994	8	1	5	Low-quality	3.97
Sample_T11C_k141_69900	5776	9	1	2	Low-quality	3.97
Sample_T10A_k141_108045	7935	5	0	3	Low-quality	3.96
Sample_C10A_k141_330743	9868	13	1	1	Low-quality	3.95
Sample_C6C_k141_156545	6888	6	0	4	Low-quality	3.95
Sample_C9A_k141_69673	6259	8	1	0	Low-quality	3.95
Sample_C12A_k141_84435	5157	5	0	3	Low-quality	3.94
Sample_C4A_k141_240508	6875	8	0	7	Low-quality	3.94
Sample_C4B_k141_141810	7854	10	0	3	Low-quality	3.94
Sample_C8B_k141_88031	7166	5	0	4	Low-quality	3.94
Sample_T3C_k141_38330	5602	2	0	2	Low-quality	3.94
Sample_T8B_k141_99862	8656	6	2	1	Low-quality	3.94
Sample_T17C_k141_33361_fragment_1	9235	8	0	1	Low-quality	3.94
Sample_C3C_k141_123655	6522	10	1	0	Low-quality	3.93
Sample_T8B_k141_139208	7313	10	1	0	Low-quality	3.93
Sample_T8C_k141_5453	5605	4	0	4	Low-quality	3.93

Sample_C3A_k141_112851	5505	7	0	1	Low-quality	3.93
Sample_T5C_k141_74581	6738	9	0	0	Low-quality	3.93
Sample_T14B_k141_179811	5565	7	1	2	Low-quality	3.92
Sample_T7A_k141_217126	9956	6	0	6	Low-quality	3.92
Sample_T10B_k141_177460	6410	7	1	0	Low-quality	3.92
Sample_T18B_k141_33232	6241	6	0	0	Low-quality	3.92
Sample_T18C_k141_29166	5679	7	0	3	Low-quality	3.91
Sample_C1B_k141_94907	6270	6	2	0	Low-quality	3.91
Sample_C5C_k141_107675	7194	16	1	1	Low-quality	3.9
Sample_T10C_k141_28190	5630	13	0	0	Low-quality	3.9
Sample_T11C_k141_683	5939	9	0	7	Low-quality	3.9
Sample_T14B_k141_3622	5454	3	0	3	Low-quality	3.9
Sample_T3B_k141_68558	6857	12	3	0	Low-quality	3.9
Sample_T8B_k141_18819	5636	8	1	0	Low-quality	3.9
Sample_C2A_k141_100711	6498	7	0	3	Low-quality	3.89
Sample_C8C_k141_15412	5489	8	1	1	Low-quality	3.89
Sample_T13C_k141_79158	9275	10	1	3	Low-quality	3.89
Sample_T16C_k141_5571	7739	9	0	6	Low-quality	3.89
Sample_T8A_k141_41538	14786	16	1	4	Low-quality	3.89
Sample_T10B_k141_68533	6140	6	5	0	Low-quality	3.89
Sample_C10A_k141_148135	6773	7	0	3	Low-quality	3.88
Sample_C12A_k141_196675	6053	6	0	0	Low-quality	3.88
Sample_T11C_k141_95455	45783	48	1	18	Low-quality	3.88
Sample_T4B_k141_238839	13517	7	1	1	Low-quality	3.88
Sample_T6C_k141_79357	5109	7	1	1	Low-quality	3.88
Sample_C4C_k141_260149	6453	11	5	1	Low-quality	3.88
Sample_C8C_k141_98370	5861	8	2	1	Low-quality	3.88
Sample_C14C_k141_186044	6265	17	1	0	Low-quality	3.87
Sample_T15A_k141_127520	5868	10	0	7	Low-quality	3.87
Sample_T16B_k141_70643	7103	6	1	1	Low-quality	3.87
Sample_C14C_k141_80822	23293	26	0	23	Low-quality	3.86
Sample_C7A_k141_127893	18165	15	2	9	Low-quality	3.86
Sample_T8B_k141_209163	6150	7	1	0	Low-quality	3.86
Sample_C14C_k141_81036	8118	6	0	3	Low-quality	3.86
Sample_C7C_k141_29524	6795	20	1	0	Low-quality	3.86
Sample_C9A_k141_125829	5385	4	1	0	Low-quality	3.86
Sample_T12C_k141_113039	6225	8	1	0	Low-quality	3.86
Sample_T17A_k141_8180	7095	14	1	1	Low-quality	3.85
Sample_T14B_k141_97343_fragment_1	14862	9	1	1	Low-quality	3.85

Sample_C10A_k141_54823	7730	6	1	4	Low-quality	3.84
Sample_C11A_k141_37733	6645	13	2	1	Low-quality	3.84
Sample_C14B_k141_90266	6419	11	3	0	Low-quality	3.84
Sample_C9A_k141_68509	5614	15	0	0	Low-quality	3.84
Sample_T8B_k141_96873	6212	16	1	0	Low-quality	3.84
Sample_C13B_k141_104717	9000	8	1	6	Low-quality	3.83
Sample_T13B_k141_114993	6261	6	0	5	Low-quality	3.83
Sample_T16C_k141_44868	5134	2	0	0	Low-quality	3.83
Sample_T8B_k141_118240	6720	23	0	0	Low-quality	3.83
Sample_C11A_k141_98741	6305	12	1	0	Low-quality	3.83
Sample_C2C_k141_116480	7536	10	0	7	Low-quality	3.82
Sample_C9B_k141_16612	5672	6	0	3	Low-quality	3.82
Sample_T6A_k141_261955	5034	6	0	1	Low-quality	3.82
Sample_C1C_k141_92572	9389	7	1	0	Low-quality	3.82
Sample_C7B_k141_26545	11742	10	1	1	Low-quality	3.82
Sample_T12C_k141_2728	6746	9	2	0	Low-quality	3.82
Sample_C12A_k141_1734	7839	10	0	2	Low-quality	3.81
Sample_C12C_k141_77771	45035	49	1	16	Low-quality	3.81
Sample_T15B_k141_114176	7092	11	1	2	Low-quality	3.81
Sample_T16C_k141_5606	8041	6	0	5	Low-quality	3.81
Sample_C4C_k141_233928	6100	7	0	0	Low-quality	3.81
Sample_T3A_k141_45634	6127	6	2	0	Low-quality	3.81
Sample_T7C_k141_155457	10426	13	2	0	Low-quality	3.81
Sample_T6C_k141_232341	8520	9	2	2	Low-quality	3.8
Sample_T3B_k141_172399	5382	7	0	0	Low-quality	3.79
Sample_T8B_k141_188619	6135	4	2	0	Low-quality	3.79
Sample_C12B_k141_122734	5434	7	0	2	Low-quality	3.78
Sample_C4C_k141_161543	5325	7	0	4	Low-quality	3.78
Sample_T14A_k141_241184	6192	3	1	0	Low-quality	3.78
Sample_C1B_k141_127300	7348	6	0	0	Low-quality	3.78
Sample_C7C_k141_77076	7699	8	0	6	Low-quality	3.77
Sample_T15A_k141_111657	6170	7	0	0	Low-quality	3.77
Sample_T6C_k141_90723	7452	6	0	2	Low-quality	3.77
Sample_T4B_k141_20368	5192	6	0	0	Low-quality	3.77
Sample_T6A_k141_237759	6481	13	3	1	Low-quality	3.76
Sample_C5A_k141_93339	6860	8	2	0	Low-quality	3.76
Sample_C8C_k141_23090	5503	9	2	0	Low-quality	3.76
Sample_C4A_k141_189570	5583	15	1	0	Low-quality	3.75
Sample_T16B_k141_110464	7434	6	0	5	Low-quality	3.75

Sample_T2B_k141_43918	6529	5	0	3	Low-quality	3.75
Sample_C7C_k141_93684	11154	10	1	1	Low-quality	3.75
Sample_T10A_k141_98298	5495	7	1	0	Low-quality	3.75
Sample_T12B_k141_106282	6675	6	1	0	Low-quality	3.75
Sample_C5A_k141_136591	5411	10	1	2	Low-quality	3.74
Sample_T6C_k141_253343	7461	6	1	4	Low-quality	3.74
Sample_C11A_k141_133147	6120	9	1	0	Low-quality	3.74
Sample_T12B_k141_142883	6228	10	2	0	Low-quality	3.74
Sample_T3B_k141_5538	6530	5	0	0	Low-quality	3.73
Sample_C13A_k141_8196	13763	17	3	3	Low-quality	3.73
Sample_T12B_k141_100157	8266	13	2	0	Low-quality	3.73
Sample_T14A_k141_62438	6494	11	2	0	Low-quality	3.73
Sample_T17B_k141_31746	5046	6	1	1	Low-quality	3.72
Sample_T8A_k141_213074	6486	8	0	6	Low-quality	3.72
Sample_C4B_k141_253915	5061	9	3	0	Low-quality	3.72
Sample_C5A_k141_76364	6533	10	3	0	Low-quality	3.72
Sample_C8C_k141_70110	5416	9	1	0	Low-quality	3.72
Sample_C3C_k141_173884	6644	6	1	0	Low-quality	3.71
Sample_C6B_k141_34354	51473	50	2	30	Low-quality	3.71
Sample_T5C_k141_102445	5524	11	1	1	Low-quality	3.71
Sample_C1C_k141_164011	5063	9	0	0	Low-quality	3.71
Sample_C8B_k141_89364	5859	9	1	0	Low-quality	3.71
Sample_T10A_k141_120012	6104	8	5	0	Low-quality	3.71
Sample_T13B_k141_168336	8092	5	1	1	Low-quality	3.71
Sample_T18B_k141_12831	6371	9	6	0	Low-quality	3.71
Sample_C3C_k141_88762	5799	9	1	0	Low-quality	3.7
Sample_C5A_k141_45432	6488	21	2	0	Low-quality	3.7
Sample_T3A_k141_105578	7019	7	0	3	Low-quality	3.7
Sample_C8C_k141_4758	41280	35	2	8	Low-quality	3.69
Sample_T13B_k141_15385	12132	5	0	3	Low-quality	3.69
Sample_T13C_k141_29534	12171	15	2	2	Low-quality	3.69
Sample_T13B_k141_95296	8017	11	1	0	Low-quality	3.69
Sample_T6A_k141_82739	5242	10	0	0	Low-quality	3.69
Sample_T13B_k141_69259	13432	14	1	3	Low-quality	3.68
Sample_T8C_k141_93140	7209	8	1	1	Low-quality	3.68
Sample_C5A_k141_231615	9530	10	1	1	Low-quality	3.67
Sample_T14B_k141_167511	6771	12	1	1	Low-quality	3.67
Sample_C3A_k141_172635	6840	4	0	0	Low-quality	3.67
Sample_T14B_k141_90415	5540	9	1	0	Low-quality	3.67

Sample_C4A_k141_71692	8116	8	1	2	Low-quality	3.66
Sample_C4B_k141_100760	5472	3	2	0	Low-quality	3.66
Sample_C5C_k141_43683	5311	10	0	0	Low-quality	3.65
Sample_T2A_k141_95882	19768	25	1	11	Low-quality	3.65
Sample_C4C_k141_91365	6046	6	4	0	Low-quality	3.65
Sample_T11C_k141_80485	5526	6	0	0	Low-quality	3.65
Sample_T12A_k141_237438	6435	11	4	0	Low-quality	3.65
Sample_T12C_k141_124337	6428	14	0	0	Low-quality	3.65
Sample_C3B_k141_116172	13792	15	0	7	Low-quality	3.64
Sample_T8A_k141_173837	7394	7	0	6	Low-quality	3.64
Sample_C9A_k141_79938	5730	19	5	0	Low-quality	3.64
Sample_C14C_k141_113988	5571	8	0	8	Low-quality	3.63
Sample_T12A_k141_40202	7880	4	0	3	Low-quality	3.63
Sample_T2C_k141_161977	7511	9	0	8	Low-quality	3.63
Sample_T4A_k141_28018	5343	8	0	1	Low-quality	3.63
Sample_T5C_k141_174082_fragment_1	6766	8	0	1	Low-quality	3.63
Sample_C11A_k141_45348	6284	7	0	1	Low-quality	3.62
Sample_C11C_k141_134640	6682	10	1	0	Low-quality	3.62
Sample_C7A_k141_101755	5074	10	0	0	Low-quality	3.62
Sample_T11B_k141_34489	5515	5	0	1	Low-quality	3.62
Sample_T12C_k141_67447	5733	5	0	1	Low-quality	3.62
Sample_C10A_k141_135688	5543	12	1	0	Low-quality	3.62
Sample_C2B_k141_250728	7056	8	2	1	Low-quality	3.62
Sample_T2B_k141_238661	5629	4	0	4	Low-quality	3.61
Sample_T6A_k141_253206	13520	10	0	9	Low-quality	3.61
Sample_C10A_k141_199144	6191	9	5	0	Low-quality	3.61
Sample_C14B_k141_79155	5725	9	0	0	Low-quality	3.61
Sample_C1C_k141_158628	5815	7	0	0	Low-quality	3.61
Sample_T17B_k141_37921	6472	8	1	0	Low-quality	3.61
Sample_C2C_k141_196957	7985	8	0	8	Low-quality	3.6
Sample_C9A_k141_134072	9034	8	1	3	Low-quality	3.6
Sample_T12C_k141_60320	5539	4	0	0	Low-quality	3.6
Sample_T12B_k141_91169	5675	10	1	0	Low-quality	3.6
Sample_C6B_k141_59656	12798	20	1	1	Low-quality	3.59
Sample_T13A_k141_46432	6623	12	1	1	Low-quality	3.59
Sample_T16B_k141_86504	6265	6	0	5	Low-quality	3.59
Sample_T6C_k141_13811	6392	4	0	2	Low-quality	3.59
Sample_C11A_k141_94974	8396	9	2	2	Low-quality	3.58
Sample_C9A_k141_122959	7707	15	2	0	Low-quality	3.58

Sample_T11A_k141_48091	7715	7	0	1	Low-quality	3.58
Sample_T8B_k141_79470	9683	15	2	0	Low-quality	3.58
Sample_T12C_k141_80836	6784	8	1	0	Low-quality	3.57
Sample_C1A_k141_54026	6014	8	3	0	Low-quality	3.57
Sample_C11C_k141_109804	11580	9	0	7	Low-quality	3.56
Sample_C2A_k141_170733	7040	7	1	5	Low-quality	3.56
Sample_C5A_k141_122388	5496	9	1	0	Low-quality	3.56
Sample_T10A_k141_100598	16593	23	1	2	Low-quality	3.56
Sample_T5C_k141_48133	5660	7	1	0	Low-quality	3.56
Sample_C10A_k141_275544	5806	9	1	0	Low-quality	3.56
Sample_C8C_k141_172474	5158	7	0	1	Low-quality	3.56
Sample_T12B_k141_30835	6665	10	4	0	Low-quality	3.56
Sample_T16B_k141_173701	10871	19	1	0	Low-quality	3.56
Sample_T1C_k141_58293	6728	5	1	1	Low-quality	3.56
Sample_T6A_k141_118478	5052	8	1	1	Low-quality	3.56
Sample_C11B_k141_174115	5135	10	0	0	Low-quality	3.55
Sample_C2A_k141_298496	5066	4	0	1	Low-quality	3.55
Sample_C6C_k141_156651	6204	8	0	7	Low-quality	3.55
Sample_T3B_k141_48996	5343	3	0	0	Low-quality	3.55
Sample_T4C_k141_53513	7080	6	1	4	Low-quality	3.55
Sample_T15A_k141_148030	8640	13	3	0	Low-quality	3.55
Sample_C11B_k141_82556	19264	12	1	3	Low-quality	3.54
Sample_C4A_k141_98645	5060	8	0	1	Low-quality	3.54
Sample_C4C_k141_240105	7439	6	1	2	Low-quality	3.54
Sample_T12A_k141_75485	5122	7	0	1	Low-quality	3.54
Sample_T13C_k141_15348	6519	8	1	0	Low-quality	3.54
Sample_T4B_k141_133661	5795	10	0	0	Low-quality	3.54
Sample_T5B_k141_63502	6382	6	0	1	Low-quality	3.54
Sample_T6B_k141_136506	5098	4	1	0	Low-quality	3.54
Sample_C10C_k141_75400	6399	5	0	4	Low-quality	3.53
Sample_C14A_k141_202635	7331	5	1	1	Low-quality	3.53
Sample_T2B_k141_157719	7598	10	5	0	Low-quality	3.53
Sample_C11B_k141_8932	8282	8	1	1	Low-quality	3.52
Sample_C11B_k141_233730	8594	6	1	2	Low-quality	3.51
Sample_C5B_k141_64990	5301	8	0	6	Low-quality	3.51
Sample_C6C_k141_110234	9387	10	1	3	Low-quality	3.51
Sample_C9A_k141_61723	6355	8	0	1	Low-quality	3.51
Sample_T10B_k141_81440	6735	3	2	0	Low-quality	3.51
Sample_T15B_k141_14615	6947	5	1	0	Low-quality	3.51

Sample_C5A_k141_11546	5327	10	0	0	Low-quality	3.51
Sample_T2B_k141_276261	12342	10	1	2	Low-quality	3.51
Sample_T2C_k141_40266	7375	13	3	2	Low-quality	3.51
Sample_T11C_k141_132888	6347	11	0	0	Low-quality	3.5
Sample_T12B_k141_144599	7552	5	1	3	Low-quality	3.5
Sample_T17C_k141_3191	8628	10	1	2	Low-quality	3.5
Sample_T13A_k141_82371	7605	15	1	1	Low-quality	3.5
Sample_C1C_k141_178946	5496	7	1	0	Low-quality	3.49
Sample_C4C_k141_46800	10728	13	1	1	Low-quality	3.49
Sample_T10A_k141_86098	6091	6	0	5	Low-quality	3.49
Sample_C10A_k141_217249	5020	4	2	0	Low-quality	3.49
Sample_C7C_k141_61097	6074	6	0	2	Low-quality	3.48
Sample_T15C_k141_90270	6069	4	0	4	Low-quality	3.48
Sample_T18A_k141_7173	5527	9	0	7	Low-quality	3.48
Sample_T4C_k141_11589	6122	7	0	7	Low-quality	3.48
Sample_T6A_k141_81448	12411	8	1	4	Low-quality	3.48
Sample_C6C_k141_156639	12242	22	2	4	Low-quality	3.48
Sample_C9A_k141_130914	6151	15	1	0	Low-quality	3.48
Sample_C7A_k141_521	7460	7	1	5	Low-quality	3.47
Sample_T10A_k141_97895	6351	8	0	3	Low-quality	3.47
Sample_T11B_k141_85837	6742	11	2	2	Low-quality	3.47
Sample_C12B_k141_55585	7008	7	1	1	Low-quality	3.47
Sample_C1B_k141_120627	7474	10	3	1	Low-quality	3.47
Sample_C5A_k141_200047_fragment_1	7468	12	1	0	Low-quality	3.47
Sample_C3B_k141_185860	6045	5	0	4	Low-quality	3.46
Sample_T10A_k141_139676	11524	16	1	0	Low-quality	3.46
Sample_T14A_k141_19430	6036	6	0	4	Low-quality	3.46
Sample_T3A_k141_120783	5236	7	0	3	Low-quality	3.46
Sample_T7A_k141_205849	5374	5	0	0	Low-quality	3.46
Sample_C4B_k141_65903	6231	9	2	0	Low-quality	3.46
Sample_C5A_k141_15840	6009	7	1	0	Low-quality	3.46
Sample_T2A_k141_35946	5055	4	1	0	Low-quality	3.46
Sample_T6A_k141_151832	5584	5	2	0	Low-quality	3.46
Sample_C10C_k141_77055	7182	8	0	7	Low-quality	3.45
Sample_C3B_k141_166197	5337	7	0	7	Low-quality	3.45
Sample_C5A_k141_147705	5146	3	2	0	Low-quality	3.45
Sample_T12C_k141_152833	12365	13	1	1	Low-quality	3.45
Sample_T2B_k141_131843	5945	9	1	0	Low-quality	3.45
Sample_C12A_k141_125739	5723	6	3	0	Low-quality	3.45

Sample_T14A_k141_4001	7394	14	1	0	Low-quality	3.45
Sample_T3A_k141_3259_fragment_2	6112	9	1	2	Low-quality	3.45
Sample_T8C_k141_73126	5313	7	6	0	Low-quality	3.45
Sample_C2B_k141_314136	10469	10	0	6	Low-quality	3.44
Sample_T4B_k141_167398	5406	7	0	3	Low-quality	3.44
Sample_C9B_k141_28231	5342	10	1	0	Low-quality	3.44
Sample_C6C_k141_59167	7374	8	2	2	Low-quality	3.43
Sample_C10A_k141_83210	7982	8	1	6	Low-quality	3.42
Sample_C2B_k141_278487	5966	7	0	7	Low-quality	3.42
Sample_T2A_k141_191831	15053	15	2	3	Low-quality	3.42
Sample_C6C_k141_47406	5642	5	0	4	Low-quality	3.41
Sample_C5A_k141_154312	7564	7	1	0	Low-quality	3.41
Sample_T12B_k141_153954_fragment_1	11976	17	1	1	Low-quality	3.41
Sample_T3C_k141_45620	6616	8	3	0	Low-quality	3.41
Sample_T10C_k141_37035	8980	8	0	7	Low-quality	3.4
Sample_T11B_k141_90466	5930	6	0	4	Low-quality	3.4
Sample_T7B_k141_155596	5937	5	0	4	Low-quality	3.4
Sample_C12C_k141_123418	5112	6	1	1	Low-quality	3.4
Sample_C2B_k141_54238	5457	4	0	3	Low-quality	3.39
Sample_T12C_k141_38701	7902	8	0	7	Low-quality	3.39
Sample_C3C_k141_14539	5726	4	2	0	Low-quality	3.39
Sample_C14C_k141_41383	6229	9	1	0	Low-quality	3.38
Sample_T16B_k141_20306	6254	2	0	2	Low-quality	3.38
Sample_T18B_k141_21167	5068	6	1	0	Low-quality	3.38
Sample_T1C_k141_54683	6364	7	0	3	Low-quality	3.38
Sample_C11A_k141_133583	5493	10	1	0	Low-quality	3.38
Sample_T4C_k141_72002	5575	6	1	0	Low-quality	3.38
Sample_T8B_k141_214298	5884	6	4	0	Low-quality	3.38
Sample_C6A_k141_83796	6215	5	1	2	Low-quality	3.37
Sample_T11C_k141_23146	5191	5	0	2	Low-quality	3.37
Sample_T8C_k141_162173	7326	3	0	3	Low-quality	3.37
Sample_C7A_k141_161478	8301	13	1	0	Low-quality	3.37
Sample_C3C_k141_158652	5942	7	0	7	Low-quality	3.36
Sample_T12A_k141_205719	6917	7	0	4	Low-quality	3.36
Sample_C9A_k141_29991	5893	12	5	1	Low-quality	3.36
Sample_C10A_k141_125201	9506	6	0	4	Low-quality	3.35
Sample_C7B_k141_107812	6781	5	0	5	Low-quality	3.35
Sample_T17A_k141_40775	5850	5	0	3	Low-quality	3.35
Sample_C10A_k141_73352	9783	6	1	1	Low-quality	3.35

Sample_C5A_k141_87429	5724	5	1	0	Low-quality	3.35
Sample_C7A_k141_176599	5298	8	0	1	Low-quality	3.35
Sample_C3C_k141_82423	5132	6	0	1	Low-quality	3.34
Sample_C6C_k141_66532	5832	5	0	5	Low-quality	3.34
Sample_C9B_k141_35316	9382	12	1	3	Low-quality	3.34
Sample_T6A_k141_256360	5562	15	2	0	Low-quality	3.34
Sample_C12A_k141_173304	5845	7	1	0	Low-quality	3.34
Sample_C4B_k141_48517	5453	6	5	0	Low-quality	3.34
Sample_T15C_k141_3521	5486	10	1	0	Low-quality	3.34
Sample_T7B_k141_149941	6753	12	3	0	Low-quality	3.34
Sample_C4B_k141_31342	6702	8	1	1	Low-quality	3.33
Sample_T12A_k141_85320	5573	6	1	4	Low-quality	3.33
Sample_T14A_k141_220936	5791	8	0	1	Low-quality	3.33
Sample_T15B_k141_83747	11112	9	1	3	Low-quality	3.33
Sample_T15C_k141_57145	13248	12	3	4	Low-quality	3.33
Sample_T4B_k141_46105	7342	9	1	1	Low-quality	3.33
Sample_T8A_k141_200472	5242	6	0	0	Low-quality	3.33
Sample_C10B_k141_51126	11847	12	1	1	Low-quality	3.32
Sample_C8C_k141_18488	123911	106	2	60	Low-quality	3.31
Sample_C13C_k141_56112_fragment_1	7589	10	0	0	Low-quality	3.31
Sample_C5A_k141_53040	5822	14	1	0	Low-quality	3.31
Sample_C10C_k141_51916	5107	11	1	0	Low-quality	3.3
Sample_T1A_k141_46047	5071	9	0	7	Low-quality	3.3
Sample_C10A_k141_49839	5110	5	1	1	Low-quality	3.29
Sample_C11A_k141_65171	6201	9	1	1	Low-quality	3.29
Sample_C5B_k141_63073	10298	10	0	5	Low-quality	3.29
Sample_T3B_k141_112368	8886	6	0	6	Low-quality	3.29
Sample_T4C_k141_107469	5317	6	0	4	Low-quality	3.29
Sample_C11B_k141_149104	5498	13	0	0	Low-quality	3.29
Sample_C13A_k141_120291	5890	9	1	1	Low-quality	3.29
Sample_T5C_k141_159631	6470	7	0	2	Low-quality	3.29
Sample_C4B_k141_13480	39156	33	1	28	Low-quality	3.28
Sample_C8C_k141_22866	6511	10	1	0	Low-quality	3.28
Sample_T5C_k141_80515	5465	3	0	3	Low-quality	3.27
Sample_C11C_k141_119552_fragment_1	6346	10	1	1	Low-quality	3.27
Sample_C1C_k141_178997	5496	7	0	0	Low-quality	3.27
Sample_C5A_k141_202970	5508	7	2	0	Low-quality	3.27
Sample_C6C_k141_61306_fragment_1	6352	8	1	1	Low-quality	3.27
Sample_C5B_k141_46104	5052	4	0	1	Low-quality	3.26

Sample_C8A_k141_3804	5872	18	0	0	Low-quality	3.26
Sample_T4B_k141_341949	6402	8	1	2	Low-quality	3.26
Sample_C2C_k141_125318	7233	10	1	0	Low-quality	3.26
Sample_C10A_k141_153243	10459	8	0	8	Low-quality	3.25
Sample_C14A_k141_149225	21589	19	2	5	Low-quality	3.25
Sample_C3C_k141_50182	7048	4	0	4	Low-quality	3.25
Sample_T15A_k141_169253	5986	12	1	1	Low-quality	3.25
Sample_T15B_k141_93249	5539	18	1	0	Low-quality	3.25
Sample_T3B_k141_186474	7058	4	0	4	Low-quality	3.25
Sample_T10A_k141_184003	5499	15	3	0	Low-quality	3.25
Sample_T16A_k141_81481	5553	8	1	1	Low-quality	3.25
Sample_C10A_k141_330534	15141	11	0	7	Low-quality	3.24
Sample_C13B_k141_22678	5652	6	0	5	Low-quality	3.24
Sample_C11A_k141_113471	5526	6	1	0	Low-quality	3.24
Sample_T11A_k141_143443	5961	9	3	0	Low-quality	3.24
Sample_T14B_k141_153239	5059	7	4	1	Low-quality	3.24
Sample_C13A_k141_15814	5960	12	1	1	Low-quality	3.23
Sample_T11B_k141_135777	5753	10	0	0	Low-quality	3.23
Sample_T12A_k141_244490	5376	3	0	1	Low-quality	3.22
Sample_C1C_k141_155758	8547	12	1	0	Low-quality	3.21
Sample_T15C_k141_133586_fragment_2	8119	11	3	0	Low-quality	3.21
Sample_C4A_k141_104790	5803	6	2	2	Low-quality	3.2
Sample_C5A_k141_163505	5644	1	0	0	Low-quality	3.2
Sample_T12A_k141_163094	7348	15	2	0	Low-quality	3.2
Sample_C2A_k141_287122	6476	7	0	6	Low-quality	3.19
Sample_C3C_k141_14176	5004	9	1	0	Low-quality	3.19
Sample_T12C_k141_126413	5844	6	2	1	Low-quality	3.19
Sample_T12C_k141_140457	5633	10	2	0	Low-quality	3.19
Sample_T6A_k141_67977	5280	9	0	1	Low-quality	3.19
Sample_C3A_k141_215233	6048	7	1	2	Low-quality	3.18
Sample_C3C_k141_81437	6428	6	0	4	Low-quality	3.18
Sample_T1A_k141_29695	7499	5	1	1	Low-quality	3.18
Sample_T3B_k141_165874	149138	134	4	96	Low-quality	3.18
Sample_T8B_k141_258109	5153	9	0	0	Low-quality	3.18
Sample_T14B_k141_109428	5139	6	1	1	Low-quality	3.18
Sample_C10A_k141_107424	8904	10	1	1	Low-quality	3.17
Sample_C12B_k141_187080_fragment_1	8253	8	1	0	Low-quality	3.17
Sample_T10A_k141_72234	7082	11	1	0	Low-quality	3.17
Sample_C10A_k141_103705	12505	11	0	10	Low-quality	3.16

Sample_C1A_k141_118427	5103	7	1	1	Low-quality	3.16
Sample_C4C_k141_252345	5733	9	0	0	Low-quality	3.16
Sample_T14C_k141_102579	5817	12	1	1	Low-quality	3.16
Sample_T2B_k141_279743	6010	13	1	1	Low-quality	3.16
Sample_T12B_k141_18173	5560	14	0	0	Low-quality	3.16
Sample_T14A_k141_170640	5338	11	0	0	Low-quality	3.16
Sample_C2C_k141_248215	7902	11	0	6	Low-quality	3.15
Sample_T10C_k141_31379	7696	4	1	2	Low-quality	3.15
Sample_T12B_k141_34123	6849	6	0	4	Low-quality	3.15
Sample_T8B_k141_5697	9604	8	0	6	Low-quality	3.15
Sample_C11A_k141_173504	5411	14	4	0	Low-quality	3.15
Sample_C9A_k141_44030	6092	8	1	0	Low-quality	3.15
Sample_C1C_k141_122099	5564	8	0	1	Low-quality	3.14
Sample_T14B_k141_46729	5713	6	0	0	Low-quality	3.14
Sample_T15A_k141_173282	5414	8	0	0	Low-quality	3.14
Sample_C13C_k141_726	9527	15	2	1	Low-quality	3.14
Sample_C3C_k141_72865	5460	11	0	0	Low-quality	3.14
Sample_T7C_k141_119540	5815	5	0	4	Low-quality	3.13
Sample_T17B_k141_3741_fragment_1	5652	7	1	0	Low-quality	3.13
Sample_C1B_k141_249054	6728	11	0	0	Low-quality	3.12
Sample_C3C_k141_66935	5777	8	0	0	Low-quality	3.12
Sample_T5C_k141_82735	6026	10	0	0	Low-quality	3.12
Sample_C11A_k141_3049	5366	17	0	0	Low-quality	3.12
Sample_T8C_k141_91124	5591	6	1	0	Low-quality	3.12
Sample_C4B_k141_254401	6118	4	0	2	Low-quality	3.11
Sample_T11C_k141_107698	5737	10	1	0	Low-quality	3.11
Sample_T14A_k141_36301	26243	29	3	21	Low-quality	3.11
Sample_T16B_k141_6069	5729	6	1	0	Low-quality	3.11
Sample_T18C_k141_73432	5944	4	1	1	Low-quality	3.11
Sample_T6A_k141_88441	5650	14	1	0	Low-quality	3.11
Sample_T12C_k141_118656	5216	8	0	0	Low-quality	3.11
Sample_C5A_k141_171773	6472	8	2	1	Low-quality	3.1
Sample_T12A_k141_163109	6234	5	1	3	Low-quality	3.1
Sample_T13B_k141_79301	5257	4	1	0	Low-quality	3.1
Sample_T13C_k141_71239	5404	6	0	2	Low-quality	3.1
Sample_C10A_k141_276581	5464	4	0	3	Low-quality	3.09
Sample_C5A_k141_236691	5386	11	6	0	Low-quality	3.09
Sample_T4B_k141_45178	13822	13	2	4	Low-quality	3.09
Sample_T7B_k141_145754	6366	12	3	1	Low-quality	3.09

Sample_C4B_k141_244083	5593	2	0	2	Low-quality	3.07
Sample_C6B_k141_28190	38235	30	1	21	Low-quality	3.07
Sample_T12C_k141_83838	5981	20	0	0	Low-quality	3.07
Sample_T5C_k141_195079	7463	9	1	0	Low-quality	3.07
Sample_T6B_k141_87896	5018	6	0	0	Low-quality	3.07
Sample_C9A_k141_27898	5145	14	3	0	Low-quality	3.07
Sample_T14A_k141_176846	6292	10	1	2	Low-quality	3.07
Sample_T12A_k141_239118	5335	5	0	4	Low-quality	3.06
Sample_T16B_k141_154784	5372	8	0	0	Low-quality	3.06
Sample_C5A_k141_190823	6599	8	2	0	Low-quality	3.06
Sample_T12A_k141_92650	7879	9	2	0	Low-quality	3.06
Sample_T8B_k141_268548	6790	7	1	0	Low-quality	3.06
Sample_C10C_k141_17834	5591	4	0	4	Low-quality	3.05
Sample_C6A_k141_190674	8601	6	0	5	Low-quality	3.05
Sample_T10B_k141_131369	5208	3	0	0	Low-quality	3.05
Sample_T6C_k141_186808	9358	9	1	5	Low-quality	3.05
Sample_T7A_k141_93107	5349	7	1	0	Low-quality	3.05
Sample_C14C_k141_122589	5151	4	0	4	Low-quality	3.04
Sample_T2B_k141_293980	5315	2	0	2	Low-quality	3.04
Sample_T4C_k141_44053	9692	10	0	8	Low-quality	3.04
Sample_T5C_k141_31401	6095	7	0	0	Low-quality	3.04
Sample_T7C_k141_62615	8328	6	2	0	Low-quality	3.04
Sample_C6C_k141_80399	5553	12	1	0	Low-quality	3.03
Sample_T3B_k141_171391	5372	6	0	0	Low-quality	3.03
Sample_T6A_k141_265916	10725	16	1	0	Low-quality	3.03
Sample_C11B_k141_8456	10262	11	1	5	Low-quality	3.02
Sample_C7C_k141_52876	6152	5	1	3	Low-quality	3.02
Sample_T11B_k141_135748	5469	13	0	0	Low-quality	3.02
Sample_T12B_k141_34042	5669	9	3	4	Low-quality	3.02
Sample_T17C_k141_17838	16679	17	0	6	Low-quality	3.02
Sample_T12A_k141_341666	5176	5	1	0	Low-quality	3.02
Sample_T6C_k141_270248	6784	8	1	1	Low-quality	3.01
Sample_T12B_k141_114233	5595	9	1	0	Low-quality	3.01
Sample_C10C_k141_33779	7511	6	0	6	Low-quality	3
Sample_C11A_k141_22549	5539	7	1	0	Low-quality	3
Sample_C4B_k141_82472	10279	7	0	5	Low-quality	3
Sample_T8A_k141_122737	5435	2	0	0	Low-quality	3
Sample_T8B_k141_17029	5336	7	0	0	Low-quality	3
Sample_T8A_k141_225013	6690	11	1	0	Low-quality	3

Sample_C2C_k141_192940	5337	6	1	0	Low-quality	2.99
Sample_C9A_k141_124437	5772	4	0	3	Low-quality	2.99
Sample_C4C_k141_9992	5094	12	2	0	Low-quality	2.99
Sample_C2B_k141_279693	8383	7	1	4	Low-quality	2.98
Sample_C2C_k141_177757	5693	4	0	2	Low-quality	2.98
Sample_T16B_k141_115812	5493	9	1	0	Low-quality	2.98
Sample_C10A_k141_250804	5555	5	3	0	Low-quality	2.98
Sample_C5B_k141_50246	6480	4	2	0	Low-quality	2.98
Sample_C8A_k141_117608	9819	11	0	9	Low-quality	2.97
Sample_T10B_k141_106211	6821	8	0	2	Low-quality	2.97
Sample_T12A_k141_20105	10907	8	1	2	Low-quality	2.97
Sample_T6A_k141_166633	9371	8	0	6	Low-quality	2.97
Sample_C5A_k141_194326	6401	10	3	0	Low-quality	2.97
Sample_C5C_k141_38302	6905	12	1	0	Low-quality	2.97
Sample_T12C_k141_25490	5393	12	1	0	Low-quality	2.97
Sample_T4B_k141_271980	6976	14	3	0	Low-quality	2.97
Sample_C3B_k141_56785	5191	5	0	3	Low-quality	2.96
Sample_C4A_k141_136909	11069	10	0	7	Low-quality	2.96
Sample_C6C_k141_205170	125574	128	2	85	Low-quality	2.96
Sample_T10B_k141_99390	7726	5	1	0	Low-quality	2.96
Sample_T5B_k141_43512	6059	9	1	2	Low-quality	2.96
Sample_C4B_k141_229637	7395	4	0	3	Low-quality	2.95
Sample_T13C_k141_121350	5151	7	0	7	Low-quality	2.95
Sample_T7C_k141_64215	8078	7	0	0	Low-quality	2.95
Sample_C1B_k141_98228	6190	9	1	1	Low-quality	2.95
Sample_T12C_k141_44483_fragment_1	6480	8	2	1	Low-quality	2.95
Sample_T6A_k141_231477	5393	7	0	0	Low-quality	2.95
Sample_C12A_k141_91229	6333	6	0	4	Low-quality	2.94
Sample_T10A_k141_55479	5127	4	0	3	Low-quality	2.94
Sample_T10B_k141_163043	6579	7	0	4	Low-quality	2.94
Sample_T6A_k141_296813	8128	7	1	1	Low-quality	2.94
Sample_C11A_k141_152690	5149	14	0	0	Low-quality	2.94
Sample_C9C_k141_33723	6539	4	1	0	Low-quality	2.94
Sample_T7C_k141_122521	7167	7	2	0	Low-quality	2.94
Sample_C10C_k141_97821	5118	3	0	3	Low-quality	2.93
Sample_C8B_k141_90468	5143	14	0	1	Low-quality	2.93
Sample_C12A_k141_26161	5100	6	0	5	Low-quality	2.92
Sample_C12A_k141_19083	5383	11	1	0	Low-quality	2.92
Sample_T13B_k141_181867	8753	7	0	5	Low-quality	2.92

Sample_T4B_k141_215669	5792	5	0	1	Low-quality	2.92
Sample_T5C_k141_21654	64069	66	1	43	Low-quality	2.92
Sample_T6A_k141_250286	5220	4	1	3	Low-quality	2.92
Sample_T7A_k141_15705	9244	9	0	3	Low-quality	2.92
Sample_C10C_k141_56941	5289	2	0	2	Low-quality	2.91
Sample_C13B_k141_79828	5335	3	1	0	Low-quality	2.91
Sample_C9A_k141_83674	5217	4	0	0	Low-quality	2.91
Sample_T7A_k141_83269	5040	10	2	1	Low-quality	2.91
Sample_C4C_k141_159606	15718	13	1	2	Low-quality	2.9
Sample_C12B_k141_176296	7641	6	0	0	Low-quality	2.9
Sample_T8A_k141_106329	5209	14	1	0	Low-quality	2.9
Sample_T2B_k141_225763	6223	9	0	1	Low-quality	2.89
Sample_C2C_k141_284980	5004	7	1	0	Low-quality	2.89
Sample_C6A_k141_42940	5188	6	1	0	Low-quality	2.89
Sample_C9B_k141_69323	5217	10	4	0	Low-quality	2.89
Sample_C5A_k141_121681	7666	5	0	4	Low-quality	2.88
Sample_T12C_k141_61405	15566	12	0	8	Low-quality	2.88
Sample_T13B_k141_167003	9635	10	1	4	Low-quality	2.88
Sample_C10A_k141_62441	6235	8	1	1	Low-quality	2.88
Sample_C6B_k141_99372	5385	9	2	1	Low-quality	2.88
Sample_C2B_k141_116733	5727	6	0	3	Low-quality	2.87
Sample_T16C_k141_3052	11181	12	0	10	Low-quality	2.87
Sample_T2A_k141_184833	5298	11	1	1	Low-quality	2.87
Sample_C9A_k141_127058	5066	14	1	0	Low-quality	2.87
Sample_T7C_k141_82525	7864	12	0	0	Low-quality	2.87
Sample_C4C_k141_166281	5682	7	0	7	Low-quality	2.86
Sample_C8C_k141_172922	7404	6	2	1	Low-quality	2.86
Sample_T3C_k141_119871	5395	4	0	2	Low-quality	2.86
Sample_T4C_k141_65573	5278	2	0	2	Low-quality	2.86
Sample_T5A_k141_4734_fragment_1	5493	8	1	0	Low-quality	2.86
Sample_T18A_k141_170328	5459	5	0	4	Low-quality	2.85
Sample_T3B_k141_104453	5027	1	1	0	Low-quality	2.85
Sample_C12C_k141_194614	6166	8	4	2	Low-quality	2.84
Sample_C9C_k141_13049	5164	7	0	0	Low-quality	2.84
Sample_T12B_k141_56435	6523	7	0	4	Low-quality	2.84
Sample_T8A_k141_85143	9700	9	0	9	Low-quality	2.84
Sample_T10A_k141_85968_fragment_2	10442	9	1	1	Low-quality	2.84
Sample_T2B_k141_296194	5354	8	1	1	Low-quality	2.84
Sample_C10C_k141_62411	7014	4	1	1	Low-quality	2.83

Sample_T18A_k141_203106	6927	6	2	1	Low-quality	2.83
Sample_T13B_k141_104759	5650	7	1	1	Low-quality	2.82
Sample_T18A_k141_43716	40131	35	3	12	Low-quality	2.82
Sample_T8C_k141_71812	5193	8	1	0	Low-quality	2.82
Sample_C4C_k141_15448	6299	8	1	1	Low-quality	2.82
Sample_C8B_k141_44559	5725	5	0	1	Low-quality	2.82
Sample_C14C_k141_112597	5181	6	1	0	Low-quality	2.81
Sample_C14B_k141_81028	5039	5	1	0	Low-quality	2.81
Sample_C2B_k141_104013	5500	11	3	0	Low-quality	2.81
Sample_C11A_k141_19625	5373	19	0	1	Low-quality	2.8
Sample_C2B_k141_304154	6494	9	1	0	Low-quality	2.8
Sample_C6B_k141_129737	8116	11	0	8	Low-quality	2.8
Sample_C11A_k141_10952_fragment_2	12902	8	1	3	Low-quality	2.8
Sample_C14B_k141_47701	6034	21	1	0	Low-quality	2.8
Sample_T12B_k141_29091	5102	9	3	0	Low-quality	2.8
Sample_T8C_k141_51709_fragment_1	8292	10	2	2	Low-quality	2.8
Sample_C2C_k141_98561	16570	10	0	5	Low-quality	2.79
Sample_C5A_k141_17380	5314	8	0	3	Low-quality	2.79
Sample_C8C_k141_149864	7900	3	0	2	Low-quality	2.79
Sample_T17B_k141_34813	6438	6	0	5	Low-quality	2.79
Sample_T1A_k141_15297	5222	3	0	2	Low-quality	2.79
Sample_T1C_k141_8405	9350	9	1	2	Low-quality	2.79
Sample_C9A_k141_13147	6022	8	0	0	Low-quality	2.79
Sample_C12A_k141_14249	15467	16	0	13	Low-quality	2.78
Sample_C4B_k141_212150	11859	11	0	10	Low-quality	2.78
Sample_C5A_k141_243307	5731	6	0	1	Low-quality	2.77
Sample_C5A_k141_172205	5453	5	0	4	Low-quality	2.77
Sample_T15A_k141_113647	6404	7	1	0	Low-quality	2.76
Sample_C1B_k141_200943	5917	14	0	0	Low-quality	2.75
Sample_T16C_k141_44312	5448	5	0	2	Low-quality	2.75
Sample_T7C_k141_118844	5202	8	2	0	Low-quality	2.75
Sample_C8B_k141_88018	10584	7	0	7	Low-quality	2.74
Sample_C1C_k141_194740	5141	7	1	0	Low-quality	2.74
Sample_C5A_k141_226693	10402	15	1	0	Low-quality	2.74
Sample_T5C_k141_155766	6370	13	1	2	Low-quality	2.74
Sample_T7A_k141_100555	5376	14	1	1	Low-quality	2.74
Sample_T4B_k141_184277	14781	14	1	3	Low-quality	2.73
Sample_T8B_k141_159445	6961	8	1	1	Low-quality	2.73
Sample_T14B_k141_158921_fragment_1	6937	9	1	1	Low-quality	2.73

Sample_C1B_k141_234223	5383	4	0	3	Low-quality	2.72
Sample_C2B_k141_286836	5811	5	0	4	Low-quality	2.72
Sample_T10A_k141_160109_fragment_2	7255	9	2	0	Low-quality	2.72
Sample_T2B_k141_108574	5853	15	2	0	Low-quality	2.72
Sample_T8C_k141_26230	5416	8	1	1	Low-quality	2.72
Sample_C14B_k141_79233	5831	12	1	0	Low-quality	2.71
Sample_C4A_k141_94187	7054	11	1	2	Low-quality	2.71
Sample_T13C_k141_9694	8573	5	0	5	Low-quality	2.7
Sample_T14A_k141_191715	5745	9	1	1	Low-quality	2.7
Sample_C10B_k141_68599	6260	10	1	1	Low-quality	2.69
Sample_C2A_k141_217478	5535	12	0	0	Low-quality	2.69
Sample_C3C_k141_173618	38822	40	2	22	Low-quality	2.69
Sample_C5C_k141_621	6403	3	0	0	Low-quality	2.69
Sample_T15C_k141_77894	7527	10	1	3	Low-quality	2.69
Sample_C11C_k141_35766_fragment_2	5463	8	1	0	Low-quality	2.69
Sample_C6C_k141_66048	8397	5	0	5	Low-quality	2.68
Sample_C9C_k141_70313	6569	4	0	2	Low-quality	2.68
Sample_T6C_k141_19782	10063	13	0	9	Low-quality	2.68
Sample_T3B_k141_72029	5329	5	1	1	Low-quality	2.68
Sample_T4B_k141_151471	5893	6	1	1	Low-quality	2.67
Sample_C3C_k141_220683	7935	10	1	2	Low-quality	2.66
Sample_T10B_k141_138535	6597	7	1	1	Low-quality	2.66
Sample_T10B_k141_55113	6330	7	1	1	Low-quality	2.66
Sample_C5C_k141_109398	5875	5	1	0	Low-quality	2.65
Sample_T14A_k141_137289	5340	5	0	2	Low-quality	2.65
Sample_T16B_k141_111387	14154	11	0	6	Low-quality	2.65
Sample_T7B_k141_8507	10368	3	0	3	Low-quality	2.65
Sample_T10A_k141_149207	7177	6	0	5	Low-quality	2.64
Sample_T15B_k141_94170	9760	4	0	4	Low-quality	2.64
Sample_T16A_k141_34584	11640	8	1	2	Low-quality	2.64
Sample_T4B_k141_130155	8325	9	0	5	Low-quality	2.64
Sample_T8A_k141_231923	7616	2	0	2	Low-quality	2.64
Sample_C11B_k141_104041	6474	9	1	1	Low-quality	2.64
Sample_C1A_k141_69003	5978	4	0	2	Low-quality	2.63
Sample_C2A_k141_185488	31077	33	1	8	Low-quality	2.63
Sample_T6C_k141_114158	15632	15	0	12	Low-quality	2.63
Sample_C8A_k141_174191	6536	5	1	0	Low-quality	2.63
Sample_C12A_k141_130430	7519	9	1	4	Low-quality	2.62
Sample_C4B_k141_79607	5126	10	1	2	Low-quality	2.62

Sample_T4C_k141_35604	5261	2	0	2	Low-quality	2.62
Sample_T2C_k141_196565	6081	11	1	1	Low-quality	2.62
Sample_T6A_k141_217899	5573	4	1	1	Low-quality	2.62
Sample_C1A_k141_7490	5642	13	2	0	Low-quality	2.61
Sample_C6B_k141_2232	6303	10	1	2	Low-quality	2.61
Sample_C8C_k141_175724_fragment_1	20489	10	1	5	Low-quality	2.61
Sample_C8C_k141_99339	10283	6	0	6	Low-quality	2.6
Sample_T11A_k141_65498	5501	5	0	5	Low-quality	2.6
Sample_T6B_k141_151908	14360	14	0	13	Low-quality	2.6
Sample_T14A_k141_7604	6940	11	1	1	Low-quality	2.6
Sample_C2B_k141_16798	5122	6	0	4	Low-quality	2.59
Sample_C7B_k141_103950	6780	8	1	1	Low-quality	2.59
Sample_T15C_k141_91760	5577	7	0	0	Low-quality	2.59
Sample_T16B_k141_26315	5084	7	2	1	Low-quality	2.59
Sample_T8B_k141_209775	5340	8	0	6	Low-quality	2.59
Sample_C10C_k141_61336	5224	3	0	2	Low-quality	2.58
Sample_C2B_k141_346221	5136	6	0	2	Low-quality	2.58
Sample_T12A_k141_5948	7798	7	0	4	Low-quality	2.57
Sample_T3B_k141_6359	5054	11	1	0	Low-quality	2.57
Sample_C1C_k141_25763	8501	3	0	3	Low-quality	2.56
Sample_T4A_k141_31648	8861	4	0	3	Low-quality	2.56
Sample_T7C_k141_111714	5229	7	0	4	Low-quality	2.56
Sample_T11A_k141_56197	9729	13	1	1	Low-quality	2.56
Sample_C9A_k141_94804	6165	4	0	3	Low-quality	2.55
Sample_T3C_k141_34605	5283	2	0	0	Low-quality	2.55
Sample_C9A_k141_66339	5498	10	1	0	Low-quality	2.55
Sample_C10A_k141_320607	6139	5	0	1	Low-quality	2.54
Sample_C2C_k141_79646	9012	7	1	1	Low-quality	2.54
Sample_C14C_k141_171848	5178	6	0	6	Low-quality	2.53
Sample_T16B_k141_163722	5936	8	0	8	Low-quality	2.53
Sample_T4B_k141_32754	5250	3	0	2	Low-quality	2.52
Sample_T10A_k141_166717	5345	7	0	1	Low-quality	2.52
Sample_T2B_k141_55675	7655	8	0	4	Low-quality	2.51
Sample_T3A_k141_89742	7019	5	1	2	Low-quality	2.51
Sample_T6B_k141_155694	8181	7	0	2	Low-quality	2.51
Sample_C4A_k141_141681	5979	8	2	1	Low-quality	2.51
Sample_C11A_k141_69438	7094	4	0	3	Low-quality	2.5
Sample_C13B_k141_62702	9772	8	0	3	Low-quality	2.5
Sample_T10B_k141_85200	8592	4	0	4	Low-quality	2.5

Sample_T12A_k141_58747	15505	16	1	9	Low-quality	2.5
Sample_T4B_k141_217066	6066	8	1	0	Low-quality	2.5
Sample_C3B_k141_149099	9621	14	1	3	Low-quality	2.49
Sample_T12A_k141_195946	6198	4	1	1	Low-quality	2.49
Sample_C2A_k141_263823_fragment_1	7012	8	1	1	Low-quality	2.48
Sample_T16A_k141_72088	9559	8	1	1	Low-quality	2.48
Sample_T2B_k141_52615	5856	7	0	5	Low-quality	2.47
Sample_T4B_k141_11962	44736	45	2	27	Low-quality	2.47
Sample_T16B_k141_63407	6149	4	1	2	Low-quality	2.46
Sample_T2C_k141_275389	5320	6	0	0	Low-quality	2.46
Sample_C1B_k141_37848	5290	10	2	0	Low-quality	2.46
Sample_T15C_k141_89499	5020	8	1	0	Low-quality	2.46
Sample_C4A_k141_127654	18072	17	0	16	Low-quality	2.45
Sample_T13A_k141_78917	9053	5	0	2	Low-quality	2.45
Sample_T2B_k141_110597	5606	6	0	3	Low-quality	2.45
Sample_T8B_k141_157715	5094	4	0	3	Low-quality	2.45
Sample_C9A_k141_90870	9323	9	1	1	Low-quality	2.45
Sample_T11A_k141_62976	5712	6	1	0	Low-quality	2.45
Sample_T12A_k141_74912	5968	6	0	0	Low-quality	2.45
Sample_T8B_k141_206959	6181	6	2	1	Low-quality	2.45
Sample_C10C_k141_158133	5558	7	0	6	Low-quality	2.44
Sample_T16B_k141_127113	5329	5	0	2	Low-quality	2.44
Sample_C9B_k141_16981	10059	7	1	2	Low-quality	2.43
Sample_T4C_k141_99774	5280	5	0	1	Low-quality	2.43
Sample_C6C_k141_21026	5067	7	4	0	Low-quality	2.43
Sample_C8B_k141_84747	6068	9	1	0	Low-quality	2.43
Sample_T16C_k141_64856	6733	10	1	0	Low-quality	2.43
Sample_T3C_k141_141870	7113	9	1	1	Low-quality	2.43
Sample_T4B_k141_164967	6429	5	1	2	Low-quality	2.42
Sample_T12A_k141_116036	6419	8	2	2	Low-quality	2.42
Sample_C8C_k141_56169	5025	11	2	0	Low-quality	2.41
Sample_C13C_k141_68295	5813	4	0	3	Low-quality	2.4
Sample_C8C_k141_31194	6180	6	1	1	Low-quality	2.4
Sample_C2B_k141_8038	8555	6	0	6	Low-quality	2.39
Sample_C8B_k141_696	9367	1	0	1	Low-quality	2.39
Sample_T7A_k141_5103	11104	7	1	2	Low-quality	2.39
Sample_C7B_k141_95298_fragment_1	7912	8	0	3	Low-quality	2.39
Sample_C5C_k141_17321	5052	6	0	6	Low-quality	2.38
Sample_C7C_k141_29668	5766	4	0	4	Low-quality	2.38

Sample_T3B_k141_125972	5350	4	0	2	Low-quality	2.38
Sample_C4C_k141_15550_fragment_1	8013	10	2	1	Low-quality	2.38
Sample_T2C_k141_179845	7307	8	2	0	Low-quality	2.38
Sample_C1B_k141_41731	7081	5	0	5	Low-quality	2.37
Sample_C6B_k141_47114	8651	11	1	2	Low-quality	2.37
Sample_T3A_k141_69683	6899	5	0	3	Low-quality	2.37
Sample_C4B_k141_245658	5023	5	1	2	Low-quality	2.37
Sample_C12A_k141_25635	5621	5	1	3	Low-quality	2.36
Sample_T11B_k141_51240	9306	4	0	4	Low-quality	2.36
Sample_T4C_k141_82657_fragment_2	7766	8	0	3	Low-quality	2.36
Sample_C3C_k141_27196	7435	7	1	1	Low-quality	2.35
Sample_T2B_k141_15264	10369	11	2	2	Low-quality	2.35
Sample_T15A_k141_118890	10901	10	0	5	Low-quality	2.34
Sample_T7C_k141_88908	8520	6	0	5	Low-quality	2.34
Sample_C5C_k141_84669	7482	9	1	1	Low-quality	2.34
Sample_T11C_k141_1851	11585	9	1	2	Low-quality	2.34
Sample_T11C_k141_59004	15575	16	0	8	Low-quality	2.33
Sample_T13C_k141_80074	5642	5	0	3	Low-quality	2.33
Sample_T15B_k141_16614	12414	8	0	8	Low-quality	2.33
Sample_C11C_k141_27828	5067	3	0	1	Low-quality	2.32
Sample_C7C_k141_2051	5773	11	1	0	Low-quality	2.32
Sample_T3B_k141_13675	6153	7	1	1	Low-quality	2.32
Sample_C12B_k141_95025	6991	3	0	2	Low-quality	2.31
Sample_C13A_k141_24844	8751	3	0	1	Low-quality	2.31
Sample_T12C_k141_89235	5807	5	0	3	Low-quality	2.31
Sample_C5A_k141_236109	5532	4	1	1	Low-quality	2.3
Sample_C8C_k141_3996	5372	4	0	1	Low-quality	2.3
Sample_T8C_k141_217583	5106	6	1	0	Low-quality	2.3
Sample_C7A_k141_31221	8155	11	1	2	Low-quality	2.29
Sample_C8C_k141_164455	12931	13	0	7	Low-quality	2.29
Sample_T3A_k141_3052	6446	8	1	2	Low-quality	2.29
Sample_C12C_k141_169140	7998	3	0	2	Low-quality	2.28
Sample_C2A_k141_91105	5258	6	0	2	Low-quality	2.28
Sample_C2C_k141_85818	11412	9	1	3	Low-quality	2.28
Sample_T3B_k141_123620	10639	13	1	6	Low-quality	2.28
Sample_T17C_k141_13816	5125	7	1	0	Low-quality	2.28
Sample_C1C_k141_61592	16068	14	0	9	Low-quality	2.27
Sample_C2C_k141_29230	5060	3	0	0	Low-quality	2.27
Sample_C3A_k141_81706	5746	3	0	3	Low-quality	2.27

Sample_T8A_k141_114891	11778	10	2	2	Low-quality	2.27
Sample_T12B_k141_134668	8913	13	2	2	Low-quality	2.26
Sample_T4B_k141_286239	6657	7	1	1	Low-quality	2.26
Sample_C8C_k141_167685	7517	8	0	5	Low-quality	2.25
Sample_C4C_k141_101887_fragment_1	7742	8	0	3	Low-quality	2.25
Sample_C6C_k141_223972	5612	5	1	0	Low-quality	2.25
Sample_T4C_k141_53436	5117	7	1	1	Low-quality	2.25
Sample_T4B_k141_133042	6386	5	1	2	Low-quality	2.23
Sample_C2C_k141_283117	8148	9	1	0	Low-quality	2.23
Sample_C6B_k141_201576	8119	10	1	3	Low-quality	2.22
Sample_C4C_k141_29802	6667	9	1	2	Low-quality	2.21
Sample_T10B_k141_104514	5345	4	0	4	Low-quality	2.21
Sample_T7A_k141_55719	8546	10	1	0	Low-quality	2.21
Sample_C1B_k141_51334	5399	4	1	1	Low-quality	2.21
Sample_T6A_k141_85219	5537	10	1	1	Low-quality	2.21
Sample_T4B_k141_61008	11967	13	1	4	Low-quality	2.2
Sample_C2C_k141_35060	8351	9	1	2	Low-quality	2.19
Sample_C10B_k141_17263	5400	5	0	2	Low-quality	2.18
Sample_T16B_k141_57881	5449	6	1	3	Low-quality	2.18
Sample_C2B_k141_199785	5467	6	0	3	Low-quality	2.17
Sample_T10A_k141_93902	13523	9	1	4	Low-quality	2.17
Sample_T7A_k141_8060	7942	10	0	6	Low-quality	2.17
Sample_C13C_k141_94486	5175	3	0	1	Low-quality	2.16
Sample_C4C_k141_245509	6600	2	1	1	Low-quality	2.16
Sample_T18B_k141_1223	7708	6	0	6	Low-quality	2.16
Sample_T18C_k141_8953	8148	9	0	4	Low-quality	2.16
Sample_C10C_k141_187570	7801	3	0	2	Low-quality	2.15
Sample_T13A_k141_28398	8194	3	0	3	Low-quality	2.15
Sample_C3A_k141_120285	5849	5	0	3	Low-quality	2.14
Sample_C3A_k141_75544	7532	6	1	1	Low-quality	2.14
Sample_T12C_k141_31051	7133	7	1	1	Low-quality	2.14
Sample_C5C_k141_47727	6099	5	0	4	Low-quality	2.13
Sample_T3B_k141_81720	5984	8	1	1	Low-quality	2.13
Sample_T14B_k141_8505	12869	8	1	3	Low-quality	2.13
Sample_T16C_k141_28651	5365	7	2	1	Low-quality	2.13
Sample_C5A_k141_90678	6786	8	1	3	Low-quality	2.12
Sample_T3A_k141_22509	10911	7	0	6	Low-quality	2.12
Sample_T4B_k141_230038	12330	9	0	9	Low-quality	2.12
Sample_T7B_k141_91806	7212	6	1	0	Low-quality	2.12

Sample_T14A_k141_101805	12451	9	0	4	Low-quality	2.11
Sample_T8B_k141_100894	22656	24	0	16	Low-quality	2.11
Sample_T6C_k141_202827	5535	5	1	1	Low-quality	2.11
Sample_C1C_k141_137205	7999	5	0	5	Low-quality	2.1
Sample_C12A_k141_60038	7356	4	1	2	Low-quality	2.09
Sample_C7B_k141_174906	8950	10	1	3	Low-quality	2.09
Sample_T7A_k141_79011	6910	10	1	3	Low-quality	2.09
Sample_T7A_k141_217348	8904	8	0	7	Low-quality	2.09
Sample_C11B_k141_234217_fragment_1	86628	105	5	10	Low-quality	2.09
Sample_C8A_k141_85004	7768	6	0	5	Low-quality	2.08
Sample_T6A_k141_280828	5021	3	0	3	Low-quality	2.08
Sample_C1C_k141_46149	5393	5	1	1	Low-quality	2.07
Sample_T12A_k141_216029	8466	2	0	2	Low-quality	2.07
Sample_T15A_k141_95189	5270	4	0	3	Low-quality	2.07
Sample_T7C_k141_125172	5675	12	1	0	Low-quality	2.07
Sample_T15B_k141_16530	6321	6	1	1	Low-quality	2.06
Sample_C9C_k141_73096	6120	6	1	2	Low-quality	2.06
Sample_C10A_k141_214664	7796	9	1	0	Low-quality	2.05
Sample_C12C_k141_164888	5875	6	1	1	Low-quality	2.05
Sample_C13B_k141_53754	12870	11	0	10	Low-quality	2.04
Sample_T7B_k141_95208	5136	5	0	2	Low-quality	2.04
Sample_C12C_k141_154286	9847	6	1	1	Low-quality	2.04
Sample_T8B_k141_58674_fragment_2	10331	8	0	3	Low-quality	2.03
Sample_C12A_k141_44061	5458	10	1	1	Low-quality	2.02
Sample_T7C_k141_118578	5529	4	1	0	Low-quality	2.02
Sample_T4B_k141_35792	7118	7	0	6	Low-quality	2.01
Sample_C10C_k141_117438	8629	5	1	2	Low-quality	2
Sample_C14C_k141_12679	5012	2	0	1	Low-quality	2
Sample_C7C_k141_42571	5594	9	1	0	Low-quality	2
Sample_T12A_k141_231466	7823	9	0	8	Low-quality	1.99
Sample_C11B_k141_16878	12941	13	0	11	Low-quality	1.98
Sample_T2B_k141_257093	7713	2	0	2	Low-quality	1.97
Sample_T7A_k141_92125	12657	12	0	11	Low-quality	1.97
Sample_C7B_k141_134443	5589	8	1	1	Low-quality	1.97
Sample_C4B_k141_200743	5336	6	0	6	Low-quality	1.96
Sample_C1B_k141_189580	6786	3	0	3	Low-quality	1.95
Sample_T12C_k141_113	5290	13	1	1	Low-quality	1.95
Sample_C5A_k141_265815	6075	8	0	4	Low-quality	1.94
Sample_T12A_k141_150400	7681	7	0	5	Low-quality	1.94

Sample_T8A_k141_93408	8071	8	0	5	Low-quality	1.94
Sample_C10A_k141_37689	5353	7	1	1	Low-quality	1.94
Sample_T5A_k141_36469	7358	9	1	0	Low-quality	1.94
Sample_T12C_k141_86232	12031	12	2	3	Low-quality	1.93
Sample_T5C_k141_126186	5999	9	1	2	Low-quality	1.93
Sample_T6C_k141_174234	10271	10	1	7	Low-quality	1.93
Sample_T7C_k141_70825	5280	6	0	1	Low-quality	1.93
Sample_C7C_k141_44463	7135	7	1	1	Low-quality	1.93
Sample_T18B_k141_81939	6365	5	0	5	Low-quality	1.92
Sample_C13B_k141_17059	5474	6	0	4	Low-quality	1.91
Sample_C2B_k141_194878	7305	6	1	1	Low-quality	1.91
Sample_C8A_k141_15738	8842	8	0	6	Low-quality	1.91
Sample_T12A_k141_267619	6709	7	0	5	Low-quality	1.91
Sample_T7C_k141_47254	5224	2	1	0	Low-quality	1.91
Sample_C3A_k141_187900	7694	5	3	1	Low-quality	1.91
Sample_T15A_k141_113728	7930	6	0	4	Low-quality	1.9
Sample_T8C_k141_208459	11366	9	0	7	Low-quality	1.9
Sample_T10B_k141_34991_fragment_1	7085	9	1	2	Low-quality	1.9
Sample_T7C_k141_121644	5199	7	1	0	Low-quality	1.9
Sample_C2C_k141_313565	19267	23	0	17	Low-quality	1.89
Sample_C2B_k141_148411	20278	22	0	12	Low-quality	1.88
Sample_T13B_k141_111942	6644	14	1	1	Low-quality	1.88
Sample_C13B_k141_88463	7613	9	1	1	Low-quality	1.88
Sample_C1B_k141_87857	5331	4	0	2	Low-quality	1.86
Sample_C3A_k141_146060	5271	5	1	1	Low-quality	1.85
Sample_C5C_k141_60212	9088	8	0	7	Low-quality	1.85
Sample_C8A_k141_78102	11596	14	1	2	Low-quality	1.85
Sample_T11C_k141_108253	10042	13	1	3	Low-quality	1.85
Sample_T12A_k141_41004	7019	8	1	1	Low-quality	1.85
Sample_T2A_k141_145827	6449	8	0	6	Low-quality	1.84
Sample_C6A_k141_56367	11519	13	0	6	Low-quality	1.83
Sample_T2A_k141_196495	7347	9	1	4	Low-quality	1.83
Sample_C10A_k141_2955	5871	6	1	1	Low-quality	1.83
Sample_C4C_k141_35025	6668	10	1	2	Low-quality	1.83
Sample_C14C_k141_33861	5623	3	0	2	Low-quality	1.81
Sample_T10A_k141_50674	46705	47	3	17	Low-quality	1.81
Sample_C13A_k141_100745	5569	9	1	0	Low-quality	1.81
Sample_C7B_k141_16129	5137	5	0	4	Low-quality	1.8
Sample_T12A_k141_319396	6996	7	0	1	Low-quality	1.8

Sample_T2C_k141_29482_fragment_2	7220	9	2	1	Low-quality	1.8
Sample_T7A_k141_55769	6763	7	1	2	Low-quality	1.8
Sample_T6C_k141_16285	9419	9	0	8	Low-quality	1.79
Sample_T8A_k141_184694	9902	7	0	4	Low-quality	1.77
Sample_T12B_k141_83548	6825	14	2	0	Low-quality	1.77
Sample_T8B_k141_262934	6960	6	0	5	Low-quality	1.76
Sample_C5B_k141_87393	5833	4	1	1	Low-quality	1.76
Sample_C4A_k141_81385	16272	14	0	11	Low-quality	1.75
Sample_C4B_k141_90943	7388	12	2	4	Low-quality	1.75
Sample_C9B_k141_97483	6059	7	0	5	Low-quality	1.75
Sample_T10B_k141_111978	7172	3	0	3	Low-quality	1.75
Sample_T18A_k141_103187	5935	6	1	1	Low-quality	1.75
Sample_T4B_k141_195981	6669	4	1	2	Low-quality	1.75
Sample_T4B_k141_39115	10137	7	1	2	Low-quality	1.75
Sample_T10A_k141_97846	5078	5	1	0	Low-quality	1.73
Sample_T2A_k141_198565	5234	6	1	2	Low-quality	1.72
Sample_T4A_k141_99489	5852	6	0	4	Low-quality	1.72
Sample_T4C_k141_119295	6686	3	0	2	Low-quality	1.72
Sample_C5A_k141_95888	5380	6	1	1	Low-quality	1.71
Sample_T12B_k141_77429	5172	9	1	1	Low-quality	1.71
Sample_C12A_k141_116911	5269	6	0	3	Low-quality	1.7
Sample_C7B_k141_123400	5862	4	1	1	Low-quality	1.7
Sample_T12A_k141_60871	6328	3	0	3	Low-quality	1.69
Sample_T18A_k141_96666	10444	9	0	8	Low-quality	1.69
Sample_T10A_k141_141750	7819	13	1	0	Low-quality	1.68
Sample_T3B_k141_157662_fragment_1	6657	8	1	2	Low-quality	1.68
Sample_C10A_k141_121384	9956	11	0	6	Low-quality	1.67
Sample_T10B_k141_176819	5671	3	0	3	Low-quality	1.67
Sample_C6C_k141_227662	5033	4	0	3	Low-quality	1.66
Sample_T4A_k141_63681	9673	6	0	4	Low-quality	1.66
Sample_C1A_k141_52884	10055	10	0	4	Low-quality	1.65
Sample_C3A_k141_175217	5525	7	1	1	Low-quality	1.64
Sample_C6A_k141_112274	7983	10	2	2	Low-quality	1.64
Sample_C3A_k141_206350	5031	6	0	2	Low-quality	1.62
Sample_C10C_k141_133701	6234	12	1	3	Low-quality	1.61
Sample_T11A_k141_22229	9563	8	0	8	Low-quality	1.61
Sample_C2B_k141_51984	5770	9	2	1	Low-quality	1.61
Sample_C2A_k141_89167	10046	9	0	6	Low-quality	1.6
Sample_C4A_k141_44097	15285	14	2	4	Low-quality	1.6

Sample_T5B_k141_73923	5585	2	0	2	Low-quality	1.6
Sample_T6C_k141_61556	14308	13	1	8	Low-quality	1.6
Sample_T8B_k141_262156	8866	8	0	7	Low-quality	1.6
Sample_T14A_k141_153614	10385	11	0	1	Low-quality	1.6
Sample_T11B_k141_117554	6067	3	0	2	Low-quality	1.59
Sample_T7B_k141_96043	5743	4	1	2	Low-quality	1.59
Sample_C10A_k141_82654	5015	6	1	2	Low-quality	1.58
Sample_C1B_k141_151243	9012	7	0	6	Low-quality	1.58
Sample_T4A_k141_87294	10258	11	0	2	Low-quality	1.58
Sample_C3C_k141_197675	7256	7	1	3	Low-quality	1.57
Sample_C5C_k141_7588	9693	7	0	5	Low-quality	1.56
Sample_T18C_k141_138120	7595	9	1	1	Low-quality	1.56
Sample_C1A_k141_37096	5716	5	0	4	Low-quality	1.55
Sample_C6C_k141_27079	10404	7	0	6	Low-quality	1.55
Sample_C7B_k141_8471	8952	9	1	3	Low-quality	1.55
Sample_C9A_k141_12410	5987	7	1	1	Low-quality	1.55
Sample_T16B_k141_27694	8997	7	0	6	Low-quality	1.55
Sample_T2B_k141_64064	5807	5	0	2	Low-quality	1.55
Sample_C2A_k141_81543	8024	4	1	1	Low-quality	1.54
Sample_C5C_k141_48749	9655	10	0	8	Low-quality	1.53
Sample_T8A_k141_62321	9089	12	0	3	Low-quality	1.52
Sample_T3A_k141_35755	6450	4	0	4	Low-quality	1.51
Sample_C2B_k141_72518	15432	14	0	7	Low-quality	1.5
Sample_T4B_k141_319484	8707	7	0	4	Low-quality	1.5
Sample_C2C_k141_34821	6173	10	1	1	Low-quality	1.5
Sample_T14A_k141_32844	5388	10	2	3	Low-quality	1.5
Sample_C4B_k141_201150	11867	10	1	4	Low-quality	1.49
Sample_C10A_k141_87102	5167	8	0	5	Low-quality	1.48
Sample_C12C_k141_213299	6249	6	0	2	Low-quality	1.48
Sample_C1C_k141_137920	5594	4	0	3	Low-quality	1.48
Sample_C4B_k141_1137	5719	10	1	1	Low-quality	1.48
Sample_C9B_k141_101776	5576	7	1	2	Low-quality	1.47
Sample_T11A_k141_86721	11134	10	1	3	Low-quality	1.46
Sample_T14A_k141_76174	5549	8	1	0	Low-quality	1.46
Sample_T15C_k141_93618	15923	9	0	7	Low-quality	1.46
Sample_C12C_k141_220176	5074	3	0	2	Low-quality	1.45
Sample_T11B_k141_52588	8337	12	0	5	Low-quality	1.45
Sample_T8B_k141_83959	15782	10	0	7	Low-quality	1.45
Sample_T10A_k141_90795	7797	17	1	0	Low-quality	1.44

Sample_T15A_k141_6984	6437	7	1	2	Low-quality	1.44
Sample_C12C_k141_201883	5221	7	1	0	Low-quality	1.43
Sample_C5A_k141_139944	5859	6	1	2	Low-quality	1.42
Sample_T6A_k141_307791	9244	9	0	6	Low-quality	1.42
Sample_T7A_k141_183892	5306	3	0	2	Low-quality	1.42
Sample_T8C_k141_169673_fragment_3	15906	18	3	4	Low-quality	1.42
Sample_T12A_k141_292890	5438	7	0	1	Low-quality	1.41
Sample_T12C_k141_106402	5196	6	1	2	Low-quality	1.41
Sample_T2A_k141_6748	6783	7	1	2	Low-quality	1.41
Sample_T2B_k141_41365	15267	8	0	7	Low-quality	1.41
Sample_T3A_k141_43414	5121	4	1	1	Low-quality	1.41
Sample_T4B_k141_85689	10844	10	1	2	Low-quality	1.4
Sample_T8A_k141_47646	9049	9	0	6	Low-quality	1.4
Sample_C9A_k141_108509	5259	5	0	4	Low-quality	1.39
Sample_T10A_k141_138493	7382	9	1	1	Low-quality	1.39
Sample_T15C_k141_124621	88314	85	2	59	Low-quality	1.39
Sample_C2A_k141_103049	6232	7	0	2	Low-quality	1.39
Sample_T12B_k141_161296	5740	7	1	1	Low-quality	1.38
Sample_T15C_k141_41558	5767	6	0	3	Low-quality	1.37
Sample_T6A_k141_309357	8386	10	0	7	Low-quality	1.37
Sample_C11A_k141_133993	12226	22	1	0	Low-quality	1.37
Sample_T5C_k141_2114	8073	8	0	6	Low-quality	1.36
Sample_C4C_k141_102949	10670	11	2	3	Low-quality	1.35
Sample_T12A_k141_37875	10488	12	2	4	Low-quality	1.34
Sample_T2C_k141_70301	13488	10	2	4	Low-quality	1.34
Sample_T6C_k141_219506	7964	6	0	4	Low-quality	1.34
Sample_C12A_k141_89618	7560	4	0	3	Low-quality	1.33
Sample_T2B_k141_112812	6127	5	1	1	Low-quality	1.31
Sample_C2B_k141_326608	5498	3	1	1	Low-quality	1.3
Sample_T16A_k141_131651	7710	9	0	3	Low-quality	1.3
Sample_T6A_k141_113823	12626	28	2	0	Low-quality	1.3
Sample_T6A_k141_307229	5188	6	0	2	Low-quality	1.3
Sample_T14B_k141_31480	15332	19	1	0	Low-quality	1.29
Sample_C6B_k141_4493	10348	8	1	4	Low-quality	1.28
Sample_C6B_k141_22188	7526	6	0	6	Low-quality	1.26
Sample_T6A_k141_235154	11192	8	1	5	Low-quality	1.26
Sample_T6A_k141_147572	14324	17	2	4	Low-quality	1.26
Sample_T15B_k141_121854	5677	4	0	3	Low-quality	1.25
Sample_C1B_k141_141007	5053	6	1	2	Low-quality	1.25

Sample_C3A_k141_104684	5107	3	1	1	Low-quality	1.24
Sample_C4A_k141_142083	5675	10	1	2	Low-quality	1.24
Sample_T4B_k141_142256	8026	7	0	5	Low-quality	1.24
Sample_C1C_k141_36549	12751	13	0	8	Low-quality	1.23
Sample_C2A_k141_29737	6862	7	1	1	Low-quality	1.23
Sample_C2B_k141_109043	5117	5	0	3	Low-quality	1.21
Sample_T12C_k141_20149	7262	8	0	5	Low-quality	1.21
Sample_C6B_k141_155631	7623	8	0	7	Low-quality	1.2
Sample_T2C_k141_124212	5567	5	0	2	Low-quality	1.2
Sample_T12A_k141_59345	5386	2	0	2	Low-quality	1.19
Sample_T18B_k141_27335	5035	7	1	2	Low-quality	1.19
Sample_T1B_k141_14355	14784	18	1	12	Low-quality	1.19
Sample_T3B_k141_22186	7709	8	1	2	Low-quality	1.19
Sample_C8C_k141_40645	6432	7	1	1	Low-quality	1.18
Sample_T8A_k141_79124	7902	7	0	1	Low-quality	1.18
Sample_C8C_k141_132560	5204	8	1	3	Low-quality	1.17
Sample_C2A_k141_144780	6837	5	0	5	Low-quality	1.16
Sample_T11B_k141_34257	7645	7	0	3	Low-quality	1.15
Sample_C6B_k141_98936	5125	4	1	1	Low-quality	1.14
Sample_T16C_k141_116833	12937	5	1	1	Low-quality	1.14
Sample_C7B_k141_5114	7000	7	1	4	Low-quality	1.12
Sample_T18C_k141_34117	6343	5	0	5	Low-quality	1.12
Sample_C4A_k141_76323	5140	8	1	1	Low-quality	1.11
Sample_C2B_k141_206567	7696	7	0	5	Low-quality	1.1
Sample_C7C_k141_50198	7074	10	1	6	Low-quality	1.1
Sample_C4C_k141_190799	7248	6	1	1	Low-quality	1.1
Sample_C2C_k141_312894	13452	10	1	4	Low-quality	1.07
Sample_C3A_k141_102192	11771	8	0	5	Low-quality	1.07
Sample_C13B_k141_33730	6260	6	0	5	Low-quality	1.06
Sample_C8C_k141_153283	6139	4	1	1	Low-quality	1.04
Sample_C4A_k141_45903	8115	9	1	7	Low-quality	1.02
Sample_T8B_k141_86554	5660	4	0	4	Low-quality	1.01
Sample_T7A_k141_200064	5695	8	1	2	Low-quality	1.01
Sample_C5A_k141_142459	5874	5	1	2	Low-quality	1
Sample_C6B_k141_123115	5931	6	0	5	Low-quality	0.99
Sample_T2A_k141_181076	5567	4	0	4	Low-quality	0.99
Sample_C10A_k141_25625	5650	6	0	2	Low-quality	0.98
Sample_C2A_k141_1095	5808	6	0	3	Low-quality	0.98
Sample_T8B_k141_212946	5515	7	2	0	Low-quality	0.97

Sample_C3B_k141_184582	7575	7	1	4	Low-quality	0.96
Sample_T4B_k141_124014	5686	5	0	5	Low-quality	0.96
Sample_C1C_k141_107599	5101	5	1	1	Low-quality	0.96
Sample_T14B_k141_62853	5216	6	0	0	Low-quality	0.95
Sample_T8A_k141_212637	6117	5	0	5	Low-quality	0.95
Sample_C6C_k141_66600	9482	9	2	6	Low-quality	0.94
Sample_C11A_k141_112070	5105	5	1	1	Low-quality	0.93
Sample_C13A_k141_52919	11124	11	1	3	Low-quality	0.93
Sample_T12B_k141_158154	5053	7	1	1	Low-quality	0.92
Sample_T7C_k141_73413	5085	5	0	4	Low-quality	0.91
Sample_T1A_k141_32918	7603	6	0	5	Low-quality	0.9
Sample_T13B_k141_147572	5338	5	2	2	Low-quality	0.89
Sample_C6C_k141_59052	5120	6	0	5	Low-quality	0.87
Sample_C13B_k141_29257	6145	6	1	2	Low-quality	0.86
Sample_T16C_k141_87515	5043	5	0	4	Low-quality	0.86
Sample_C4C_k141_131743_fragment_1	8431	10	1	3	Low-quality	0.86
Sample_C10C_k141_144982	5131	5	0	4	Low-quality	0.85
Sample_T12C_k141_120103	6325	6	0	6	Low-quality	0.85
Sample_T3C_k141_59835	6263	4	1	2	Low-quality	0.85
Sample_C14C_k141_168668	5898	6	1	3	Low-quality	0.85
Sample_T11B_k141_155728	5678	6	2	2	Low-quality	0.84
Sample_T4A_k141_93873	7545	7	0	5	Low-quality	0.84
Sample_T10B_k141_148657	5610	5	0	4	Low-quality	0.83
Sample_T4B_k141_171706	5373	4	1	3	Low-quality	0.82
Sample_T6C_k141_173026	13359	7	1	6	Low-quality	0.81
Sample_C8B_k141_101615	5256	5	1	1	Low-quality	0.81
Sample_T12B_k141_39984	7518	8	1	2	Low-quality	0.8
Sample_C3C_k141_67924	5041	7	1	2	Low-quality	0.79
Sample_T10A_k141_41888	6825	8	1	3	Low-quality	0.79
Sample_T11B_k141_66026	5497	4	0	4	Low-quality	0.79
Sample_C8B_k141_36151	5001	6	1	3	Low-quality	0.79
Sample_C12C_k141_40630	5141	5	0	2	Low-quality	0.77
Sample_C14C_k141_175312	5285	5	1	2	Low-quality	0.76
Sample_C1C_k141_26847	6176	7	1	4	Low-quality	0.75
Sample_C9B_k141_18544	5322	4	1	1	Low-quality	0.75
Sample_T8B_k141_178700	5665	8	1	2	Low-quality	0.75
Sample_C10A_k141_150048	6151	7	1	2	Low-quality	0.74
Sample_C6C_k141_171246	7968	4	0	3	Low-quality	0.73
Sample_C3A_k141_92293	6092	2	1	1	Low-quality	0.71

Sample_C6C_k141_47593	7456	9	1	2	Low-quality	0.71
Sample_T4B_k141_66549	7678	4	0	2	Low-quality	0.71
Sample_C7C_k141_40251	7648	3	0	3	Low-quality	0.7
Sample_T2B_k141_302888	7539	3	0	2	Low-quality	0.69
Sample_C10C_k141_60746	6796	7	0	6	Low-quality	0.67
Sample_C3B_k141_119708	5216	5	1	2	Low-quality	0.67
Sample_C14C_k141_21768	7833	6	1	1	Low-quality	0.66
Sample_T11B_k141_43310_fragment_2	7388	10	1	7	Low-quality	0.66
Sample_C5A_k141_165478	6763	5	1	2	Low-quality	0.65
Sample_C4A_k141_184357	6743	9	2	3	Low-quality	0.65
Sample_C13A_k141_48430	8779	8	0	7	Low-quality	0.63
Sample_T17B_k141_26500	5633	7	2	1	Low-quality	0.63
Sample_T15C_k141_132838	6635	7	1	1	Low-quality	0.63
Sample_C1B_k141_94046	9495	7	1	2	Low-quality	0.62
Sample_C7B_k141_129831	6350	9	2	4	Low-quality	0.62
Sample_T8A_k141_219164	8169	9	1	1	Low-quality	0.62
Sample_T8B_k141_184088	6506	3	0	2	Low-quality	0.6
Sample_C4B_k141_1460	12335	7	1	2	Low-quality	0.59
Sample_T2B_k141_62523	6290	4	0	4	Low-quality	0.58
Sample_C4A_k141_159982	7039	8	1	2	Low-quality	0.57
Sample_T2C_k141_158636	5739	5	0	4	Low-quality	0.56
Sample_T4C_k141_29015	12537	10	1	7	Low-quality	0.56
Sample_C10C_k141_80143	5879	4	0	3	Low-quality	0.55
Sample_C9C_k141_52641	5566	7	1	2	Low-quality	0.55
Sample_T4B_k141_45727	5535	3	0	3	Low-quality	0.55
Sample_C8C_k141_116362	5483	5	0	4	Low-quality	0.54
Sample_T2C_k141_193719	5720	4	0	3	Low-quality	0.53
Sample_C4C_k141_16866	5628	3	0	3	Low-quality	0.52
Sample_C8B_k141_48937	5728	4	0	3	Low-quality	0.52
Sample_C12A_k141_101593	5484	3	0	3	Low-quality	0.51
Sample_T4A_k141_6061	5512	2	0	1	Low-quality	0.51
Sample_C10C_k141_173707	5492	5	0	2	Low-quality	0.5
Sample_C5B_k141_31374	5322	9	1	2	Low-quality	0.49
Sample_T7C_k141_67815	5362	2	0	2	Low-quality	0.49
Sample_T5C_k141_19844	5187	2	0	2	Low-quality	0.48
Sample_T16B_k141_11412	9008	9	1	6	Low-quality	0.46
Sample_T13A_k141_56835_fragment_1	11848	8	1	2	Low-quality	0.43
Sample_C2C_k141_285921	5936	7	1	2	Low-quality	0.42
Sample_C10C_k141_224224	10122	10	1	6	Low-quality	0.41

Sample_T12A_k141_7663	5355	6	1	5	Low-quality	0.4
Sample_C8B_k141_126344	11217	7	1	4	Low-quality	0.38
Sample_C4A_k141_249461	5810	4	1	2	Low-quality	0.35
Sample_C1B_k141_79771	7139	10	1	0	Low-quality	0.33
Sample_C11B_k141_119973	5986	3	1	1	Low-quality	0.32
Sample_T10C_k141_25443	6491	8	1	3	Low-quality	0.31
Sample_T8B_k141_116604	5796	11	1	1	Low-quality	0.29
Sample_C10B_k141_56519	8521	5	1	2	Low-quality	0.26
Sample_T14B_k141_215540	9593	6	1	3	Low-quality	0.26
Sample_T16B_k141_110035	5780	5	1	3	Low-quality	0.24
Sample_T16C_k141_60848	5673	9	1	4	Low-quality	0.24
Sample_T10A_k141_50534	6099	5	1	4	Low-quality	0.23
Sample_T7B_k141_11894	6055	7	1	2	Low-quality	0.19
Sample_C10A_k141_196034	10607	10	1	5	Low-quality	0.18
Sample_T4C_k141_15597	6663	5	1	2	Low-quality	0.12
Sample_C1A_k141_44306	5553	2	1	1	Low-quality	0.11
Sample_C2B_k141_210313	6218	6	1	2	Low-quality	0.11
Sample_T7A_k141_11156	6920	5	1	2	Low-quality	0.09
